# Supplementary material for: Prediction of Burkholderia pseudomallei DsbA substrates identifies potential virulence factors and vaccine targets
Source: PLoS One. 2020 Nov 20;15(11):e0241306. doi: 10.1371/journal.pone.0241306 (PMC7678975; doi:10.1371/journal.pone.0241306)
Supplement: S1 Data — (DOCX) [file pone.0241306.s001.docx]

S1 Data

Accession numbers for disease related genomes of *B. pseudomallei* used in this analysis

GCA_000410895.1

GCA_000452945.1

GCA_000452965.1

GCA_000452985.1

GCA_000453005.1

GCA_000511895.1

GCA_000511915.1

GCA_000520895.1

GCA_000521645.1

GCA_000714875.1

GCA_000932075.1

GCA_000932085.1

GCA_000932095.1

GCA_000932105.1

GCA_000932145.1

GCA_000953095.1

GCA_000954175.1

GCA_000959305.1

GCA_000961535.1

GCA_000981285.2

GCA_001182265.1

GCA_001182285.1

GCA_001182305.1

GCA_001182325.1

GCA_001191815.1

GCA_001192415.1

GCA_001192815.1

GCA_001193035.1

GCA_001193375.1

GCA_001193615.1

GCA_001193655.1

GCA_001194765.1

GCA_001194905.1

GCA_001195465.1

GCA_001196125.1

GCA_001196775.1

GCA_001197015.1

GCA_001197235.1

GCA_001197775.1

GCA_001197795.1

GCA_001197955.1

GCA_001199035.1

GCA_001199195.1

GCA_001199695.1

GCA_001199735.1

GCA_001199815.1

GCA_001199855.1

GCA_001200615.1

GCA_001200935.1

GCA_001202135.1

GCA_001202415.1

GCA_001202895.1

GCA_001203375.1

GCA_001203735.1

GCA_001204155.1

GCA_001204275.1

GCA_001204455.1

GCA_001204515.1

GCA_001205635.1

GCA_001205935.1

GCA_001206455.1

GCA_001206495.1

GCA_001207035.1

GCA_001207055.1

GCA_001207675.1

GCA_001207785.1

GCA_001208265.1

GCA_001208545.1

GCA_001209045.1

GCA_001209405.1

GCA_001209505.1

GCA_001209525.1

GCA_001210025.1

GCA_001210645.1

GCA_001210705.1

GCA_001210965.1

GCA_001211245.1

GCA_001211305.1

GCA_001211345.1

GCA_001211385.1

GCA_001211405.1

GCA_001211505.1

GCA_001211565.1

GCA_001211705.1

GCA_001211865.1

GCA_001212025.1

GCA_001212065.1

GCA_001212185.1

GCA_001212265.1

GCA_001212325.1

GCA_001212405.1

GCA_001212465.1

GCA_001212705.1

GCA_001233045.1

GCA_001262275.1

GCA_001262315.1

GCA_001262355.1

GCA_001270725.1

GCA_001275585.1

GCA_001277975.1

GCA_001320065.1

GCA_001320105.1

GCA_001320145.1

GCA_001320185.1

GCA_001320215.1

GCA_001320255.1

GCA_001320285.1

GCA_001320325.1

GCA_001320365.1

GCA_001320395.1

GCA_001320425.1

GCA_001320465.1

GCA_001320505.1

GCA_001320545.1

GCA_001320585.1

GCA_001320615.1

GCA_001320645.1

GCA_001320685.1

GCA_001320725.1

GCA_001320755.1

GCA_001320795.1

GCA_001320825.1

GCA_001320865.1

GCA_001320905.1

GCA_001320945.1

GCA_001320985.1

GCA_001321015.1

GCA_001321045.1

GCA_001321065.1

GCA_001321085.1

GCA_001321105.1

GCA_001321125.1

GCA_001321145.1

GCA_001321165.1

GCA_001321185.1

GCA_001321205.1

GCA_001321225.1

GCA_001321245.1

GCA_001321265.1

GCA_001321285.1

GCA_001321305.1

GCA_001321325.1

GCA_001321345.1

GCA_001321365.1

GCA_001321385.1

GCA_001321405.1

GCA_001321425.1

GCA_001321445.1

GCA_001321465.1

GCA_001321485.1

GCA_001321505.1

GCA_001321525.1

GCA_001321545.1

GCA_001321565.1

GCA_001321585.1

GCA_001321605.1

GCA_001321625.1

GCA_001326895.1

GCA_001326915.1

GCA_001326935.1

GCA_001326955.1

GCA_001326975.1

GCA_001326995.1

GCA_001327015.1

GCA_001327035.1

GCA_001327075.1

GCA_001327095.1

GCA_001327115.1

GCA_001327135.1

GCA_001327155.1

GCA_001327175.1

GCA_001327195.1

GCA_001327275.1

GCA_001327315.1

GCA_001327395.1

GCA_001327415.1

GCA_001327455.1

GCA_001327515.1

GCA_001327535.1

GCA_001327575.1

GCA_001885195.1

GCA_001887555.1

GCA_001887575.1

GCA_001905265.1

GCA_001974745.1

GCA_001975065.1

GCA_001975085.1

GCA_001975105.1

GCA_001976165.1

GCA_001976175.1

GCA_001976185.1

GCA_001976195.1

GCA_001976245.1

GCA_001976255.1

GCA_001976265.1

GCA_001976275.1

GCA_001976325.1

GCA_001976335.1

GCA_001976345.1

GCA_001976385.1

GCA_001976395.1

GCA_001976405.1

GCA_001976415.1

GCA_001976465.1

GCA_001976475.1

GCA_001976485.1

GCA_001976495.1

GCA_001976545.1

GCA_001976565.1

GCA_001976575.1

GCA_001976585.1

GCA_001976625.1

GCA_001976645.1

GCA_001976655.1

GCA_001976675.1

GCA_001976685.1

GCA_001976725.1

GCA_001976735.1

GCA_001976755.1

GCA_001976785.1

GCA_001976805.1

GCA_001976815.1

GCA_001976825.1

GCA_001976865.1

GCA_001976885.1

GCA_001976895.1

GCA_001976905.1

GCA_001976925.1

GCA_001976965.1

GCA_001976975.1

GCA_001977005.1

GCA_001977015.1

GCA_001977045.1

GCA_001977055.1

GCA_001977085.1

GCA_001977095.1

GCA_001977125.1

GCA_001977135.1

GCA_001977165.1

GCA_001977185.1

GCA_001977195.1

GCA_001977225.1

GCA_001977245.1

GCA_001977265.1

GCA_001977275.1

GCA_001977285.1

GCA_001977325.1

GCA_001977345.1

GCA_001977365.1

GCA_001977375.1

GCA_001977385.1

GCA_001977425.1

GCA_001977435.1

GCA_001977465.1

GCA_001977475.1

GCA_001977495.1

GCA_001977525.1

GCA_001977545.1

GCA_001977565.1

GCA_001977575.1

GCA_001977605.1

GCA_001977615.1

GCA_001977645.1

GCA_001977655.1

GCA_001977675.1

GCA_001977695.1

GCA_001977725.1

GCA_001977735.1

GCA_001977745.1

GCA_001977785.1

GCA_001977805.1

GCA_001977815.1

GCA_001977825.1

GCA_001977865.1

GCA_001977875.1

GCA_001977885.1

GCA_001977915.1

GCA_001977945.1

GCA_001977955.1

GCA_001977975.1

GCA_001978005.1

GCA_001978015.1

GCA_001978045.1

GCA_001978055.1

GCA_001978085.1

GCA_001978105.1

GCA_001978115.1

GCA_001978125.1

GCA_001978165.1

GCA_001978175.1

GCA_001978185.1

GCA_001978205.1

GCA_001978245.1

GCA_001978265.1

GCA_001978285.1

GCA_001978295.1

GCA_001978325.1

GCA_001978345.1

GCA_001978365.1

GCA_001978385.1

GCA_001978405.1

GCA_001978415.1

GCA_001978445.1

GCA_001978455.1

GCA_001978485.1

GCA_001978505.1

GCA_001978515.1

GCA_001978525.1

GCA_001978565.1

GCA_001978585.1

GCA_001978605.1

GCA_001978615.1

GCA_001978635.1

GCA_001978665.1

GCA_001978675.1

GCA_001978705.1

GCA_001978725.1

GCA_001978745.1

GCA_001978765.1

GCA_001978785.1

GCA_001978795.1

GCA_001978825.1

GCA_001978835.1

GCA_001978865.1

GCA_001978875.1

GCA_001978905.1

GCA_001978925.1

GCA_001978935.1

GCA_001978965.1

GCA_001978985.1

GCA_001978995.1

GCA_001979015.1

GCA_001979045.1

GCA_001979065.1

GCA_001979085.1

GCA_001979105.1

GCA_001979115.1

GCA_001979135.1

GCA_001979165.1

GCA_001979175.1

GCA_001979195.1

GCA_001979215.1

GCA_001979245.1

GCA_001979255.1

GCA_001979275.1

GCA_001979285.1

GCA_001979325.1

GCA_001979335.1

GCA_001979345.1

GCA_001979385.1

GCA_001979405.1

GCA_001979415.1

GCA_001979435.1

GCA_001979455.1

GCA_001979485.1

GCA_001979495.1

GCA_001979505.1

GCA_001979545.1

GCA_001979565.1

GCA_001979585.1

GCA_001979595.1

GCA_001979615.1

GCA_001979645.1

GCA_001979665.1

GCA_001979675.1

GCA_001979695.1

GCA_001979725.1

GCA_001979745.1

GCA_001979755.1

GCA_001979765.1

GCA_001979805.1

GCA_001979815.1

GCA_001979835.1

GCA_001979865.1

GCA_001979885.1

GCA_001979895.1

GCA_001979905.1

GCA_001979945.1

GCA_001979965.1

GCA_001979975.1

GCA_001979995.1

GCA_001980025.1

GCA_001980045.1

GCA_001980065.1

GCA_001980075.1

GCA_001980105.1

GCA_001980125.1

GCA_001980145.1

GCA_001980155.1

GCA_001980175.1

GCA_001980205.1

GCA_001980215.1

GCA_001980245.1

GCA_001980265.1

GCA_001980275.1

GCA_001980305.1

GCA_001980315.1

GCA_001980335.1

GCA_001980365.1

GCA_001980385.1

GCA_001980395.1

GCA_001980425.1

GCA_001980435.1

GCA_001980465.1

GCA_001980485.1

GCA_001980495.1

GCA_001980515.1

GCA_001980545.1

GCA_001980565.1

GCA_001980585.1

GCA_001980605.1

GCA_001980625.1

GCA_001980645.1

GCA_001980655.1

GCA_001980675.1

GCA_001980695.1

GCA_001980725.1

GCA_001980735.1

GCA_001980755.1

GCA_001980775.1

GCA_001980805.1

GCA_001980815.1

GCA_001980835.1

GCA_001980845.1

GCA_001980885.1

GCA_001980895.1

GCA_001980905.1

GCA_001980915.1

GCA_001980965.1

GCA_001980985.1

GCA_001980995.1

GCA_001981025.1

GCA_001981045.1

GCA_001981055.1

GCA_001981085.1

GCA_001981105.1

GCA_001981125.1

GCA_001981135.1

GCA_001981165.1

GCA_001981185.1

GCA_001981195.1

GCA_002110925.1

GCA_002110945.1

GCA_002110965.1

GCA_002110985.1

GCA_002111005.1

GCA_002111025.1

GCA_002111045.1

GCA_002111065.1

GCA_002111085.1

GCA_002111105.1

GCA_002111125.1

GCA_002111165.1

GCA_002111185.1

GCA_002111205.1

GCA_002111225.1

GCA_002111245.1

GCA_002111265.1

GCA_002111285.1

GCA_002111305.1

GCA_002111325.1

GCA_002111345.1

GCA_002111365.1

GCA_002111385.1

GCA_002113945.1

GCA_002115385.1

GCA_002245325.2

GCA_002843645.1

GCA_002860065.1

GCA_002900605.1

GCA_002900625.1

GCA_002900645.1

GCA_002900665.1

GCA_002920945.1

GCA_002920995.1

GCA_002921005.1

GCA_002921015.1

GCA_002921055.1

GCA_002921075.1

GCA_002921105.1

GCA_003268455.1

GCA_003268465.1

GCA_003546995.3

GCA_003547015.1

GCA_003547035.1

GCA_003547055.1

GCA_003583425.1

GCA_003583435.1

GCA_003584055.1

GCA_003584065.1

GCA_004323015.1

GCA_004323035.1

GCA_004348055.1

GCA_004348075.1

GCA_004360045.1

GCA_004360055.1

GCA_004360065.1

GCA_004360075.1

GCA_004360125.1

GCA_004360205.1

GCA_004367665.1

GCA_004367685.1

GCA_004367705.1

GCA_004367725.1

GCA_004526325.1

GCA_005853645.1

GCA_005862325.1

GCA_006542565.1

GCA_006542585.1

GCA_007995115.1

GCA_900006245.1

GCA_900446265.1
