# Supplementary material for: Prediction of Burkholderia pseudomallei DsbA substrates identifies potential virulence factors and vaccine targets
Source: PLoS One. 2020 Nov 20;15(11):e0241306. doi: 10.1371/journal.pone.0241306 (PMC7678975; doi:10.1371/journal.pone.0241306)
Supplement: S2 Data — (DOCX) [file pone.0241306.s002.docx]

S2 Data

Core genome (4,498 gene products) of disease related *B. pseudomallei* (fasta format)

>WP_183176400.1 glutamate-1-semialdehyde 2,1-aminomutase [Burkholderia pseudomallei]

MLDERHRGTPARRTAAAAPKPVARVSGTISRIDTRDASRGASPLARTLSQVQFMSNNQTLFERAQRTIPG

GVNSPVRAFRSVGGTPRFVARAQGAYFWDADGKRYIDYIGSWGPMIVGHVHPDVLAAVQRVLADGFSFGA

PTEAEIEIAEEICKLVPSIEQVRMVSSGTEATMSALRLARGFTGRSRIVKFEGCYHGHADSLLVKAGSGL

LTFGNPTSAGVPADVAKHTTVLEYNNVAALEEAFAAFGGEIAAVIVEPVAGNMNLVRGTPEFLNALRALT

AKHGAVLIFDEVMCGFRVALGGAQQHYGITPDLTCLGKVIGGGMPAAAFGGRGDIMSHLAPLGGVYQAGT

LSGNPVAVAAGLATLRLIQAPGFHDALADKTRRLADGLAAEARAAGVPFSADAIGGMFGLYFTEQVPASF

ADVTKSDIERFNRFFHLMLDAGVYFAPSAYEAGFVSSAHDDATLDATLDAARRAFAALRA

>WP_183163289.1 tetratricopeptide repeat protein [Burkholderia pseudomallei]

MNDRHFDDACDGACTARRPGRAARASRARSMGSMGGGIGAQRAGRVRRAMPVALLLAALLAGCASHGVFQ

PRPVLSQRGTVGDGDLSVAESALAAGDAELAATLFERALKADPRSLPAQVGLGDAMYQTGELARAGVLYA

QAAAAAPDDPRAQLGLARVALRERHLDDALARYAKLNAARPDNIAAAAGLGTVLDLLGRHDDAQRVYRAA

LARHPDAHALKTDLGLSLVLANRAREGANVLLDIAGLPNAPAQAREDLALAYGMLGNDDAAKRILVHDLP

AASAEDNLRFYRNVRERRAAGAGGGGGAPGPAGAAGATNAANAANAAAGPTMTTAANAPRAAHAAAAPQA

PYAGRAIADAGLAPVRVEVVK

>WP_055315119.1 hypothetical protein [Burkholderia pseudomallei]

MPFPCSCSPLSHVAASRRLLLAGAFCACALAAAPARAQEFALFGGRLAGGGGRSYAWAFDYQEGLGRYAA

AGFAWYNEGHIPNHHRDGQAVQLWGRLPLRENRFVISAGVGPYRYFDTVAATEGRGYSNSHGWGMLMSVR

AAYYTSHRWLAQLQVNRTQVFSGPNTTSLMFGIGYQLDAPDTPGPRDRAAGRAGRVTANEVTAMLGETIL

NSRRSPTALGGSLEYRRGIAKYVDWTATYLYEGAKQSVRRNGIASQVWLTRAFLDDKIALSAGAGAYLTL

NERDIRGRPGEGDGRVSGIVSISASYRFDDRWLARVTWNRVVTRYDRDTDVIQAGLGYRF

>WP_024428965.1 cytochrome P450/oxidoreductase [Burkholderia pseudomallei]

MTTHSASTPPALPAAQCPFRPVSDDADAHPAGCPASAGAAAFDPFDDAYQQDPPEYVRWAREREPVFYSP

KLGYWVVARYDDIKTIFRDNRTFSPSIALEKITPTGPDANAALASYGYAMGRTLVNEDEPAHMARRRLLI

EPFTPDALRAHEPMVRRLAREYVDRFVDDGRADLVDQMLWEVPLTVALHFLGVPEADMDTLRAYSIAHTV

NTWGRPRPDEQVAVAHAVGQFWQYAGKVLEQMREDPSGPGWMQFGIRMQREHPDIVTDSYLHSMMMAGIV

AAHETTANATANALKLLLEHPRAWRELCDDPALIPNAVDECLRHSGSIVAWRRIATRDATVGGVAIPAGA

KLLIVTSSANRDERRFADPDLFDIHRDNPADHLTFGYGSHQCMGKHLARMEMQIFIDELSRRLPHMRLAE

QRFTYVPNTSFRGPEHLWVEWAPAENPQRRGHGARRCALAVRIDESNARRAGRALRVDSVMPVAERVLRV

RLVAPDGAPLPRWSAGAHIDVECGDTGLSRQYSLCGDPAERGVYEIAVLREPASRGGSAWLHERLRPGMR

VKIRGPRNHFRLDGHAARAILIAGGIGITPIGAIAREARARGIDYALHYCGCSRRTMAFVDELAALHGER

LRLHVSDEGTRHDFSALDVAAGTQVYACGPARMLDALAACAAHWPAHALRVEHFAAAAPRVDPRTDQPFE

VELKDSGLTLTVPANRTLLATLRAANVDVQSDCEEGLCGSCEVRVLAGEVDHRDSVLSADERALNTRMMA

CCSRARHGRLVLEL

>WP_004554725.1 MULTISPECIES: isocyanide synthase family protein [pseudomallei group]

MKCDRNDEIALAILGEILGIHRRYPEYTTDSDIRHEIEQIHAVQLPRIRAFVDAARPVEFVLPAFPSKSP

NPNKVLGRLPDMAEQLSLSFLNDLCERISGFYAPGAKLTICSDGRVFGDLIRVDDRDITAYQHALGQLIA

ALRADRLSTYNLENFEAFAQRAANFDDMRRRLVDEFADPIEAIRHRLMADEEGTLLYRAMTRFMFEDGFT

PDYRGSKAALQKDSKTRALGVIQRSWAWGALLATRFPDAIRLSIHPQPAASLKIGVHMMPTRDNWLTPWH

GVAVDLGGQFALMKRRDVELLGGRVVMRGGRPSHYAIERWRVAGDALAPLAGGGARPRADAGGALVISGE

AV

>WP_004546977.1 ATP-dependent zinc metalloprotease FtsH [Burkholderia pseudomallei]

MNNNMFSKAAVWLVIALVLFTVFKQFDKPRVQEGVSYSQFMDDAKNGKVKNVVVQGRNLTVTPADGQKYQ

IVSPGDIWMVGDLMKYGVQVSGKADDEPNALVSALYYLGPTLLIIVFWFYMMRQMQGGGKGGAFSFGKSR

ARLIDENNNAVNFSDVAGCDEAKEEVSELVDFLRDPQKFQKLGGRIPRGVLLVGPPGTGKTLLARAIAGE

AKVPFFSISGSDFVEMFVGVGAARVRDMFEQAKKHAPCIVFIDEIDAVGRHRGAGMGGGNDEREQTLNQM

LVEMDGFEANSGVIVIAATNRSDVLDKALLRPGRFDRQVYVGLPDIRGREQIMRVHLRKVPIANDVDAAV

IARGTPGFSGADLANLVNEAALFAARRGKRIVEMQDFEDAKDKIFMGPERKSAVIREEAKRATAYHESGH

AVIAKLLPKADPVHKVTIIPRGRALGVTWQLPEHDNETYSKDYLLDRLAILFGGRVAEELFLNLVSTGAS

DDFNKATQTARAMVARFGMTDALGPMVYVDDENDGGPFGRGFTRTISEATQQKVDAEIRRVLDDQYGLAR

RLLEENRDKVEAMTAALMEWETIDADQINDIMEGRPPRSPKSVPPAGDPSSGGSSGAEVKPGNATAPA

>WP_004194622.1 MULTISPECIES: HD domain-containing protein [Burkholderia]

MALTLDDIRMLFDRHGDIAYSGEPVTQREHALQSAQLAEQAGANDALIAAALLHDLGHLLNRRGETPTAH

GIDDLHQYCVLPFLRPLFPDAVLEPIRLHVDAKRCLCAIDASYYARLSADSVRSLHLQGGVFSDAEAEAF

LKRPYAPDAIRVRRWDDLAKMAGKPTPGLDHYMRAVARAAIAK

>WP_004186989.1 MULTISPECIES: dipeptidase [Burkholderia]

MSTLHQDSIIIDGLNISKFERSVFEDMQKGGVTAANCTVSVWENFTKTVDNIALMKKQIRENGELLTLVR

TTDDILRAKREGRTGVILGFQNAHAFEDNLGYVEAFADMGVRVVQLCYNTQNLVGTGCYERDGGLSDFGR

EVITEMNRVGIMVDLSHVGGNTSSEAIAFSKKPVCYSHCLPSGLKEHPRNKSDAQLKEIADAGGFVGVTM

FAPFLKRGIDATIDDYIEAIGYVVNLIGEDAVGIGTDFTQGYSVDFFDWLTHDKGRYRRLTNFGKVVNPE

GIRTIGEFPNLTAAMERAGWKASRIRKIMGENWVRVFKEVWGA

>WP_004186237.1 MULTISPECIES: acyl-CoA desaturase [Burkholderia]

MLNSLLDFLSNGLLRFSWWQIVLFTLAVTHVTIVGVTVYLHRCQAHRALDLHPIMSHFFRAWLWMTTGML

TGQWAAIHRKHHAKCETEEDPHSPQTRGIWKVLLEGAELYRAEAKNEETMRKYGHGTPNDWLERNVYSKY

PILGVSLMMVIDVALFGLVGLTVWAVQMVWIPFWAAGVVNGLGHFWGYRNFNAADASTNLFPWGIVIGGE

ELHNNHHTFATSAKLSNKWYEFDIGWMYIRIMSAFGLAKVKKIAPTPRLAARKTVLDQETLQAVLSNRYE

VMARYAKTLKRAYRQELAHLKELGAREKYQLMRGARKWFHKDEAGLDEPQKRMLPEIFANSQKLHTFFQL

RAELTAIWERSNASREQLLTQLQDWCHRAEQSGIKALQEFATRLRRYA

>WP_183158388.1 helix-turn-helix domain-containing protein [Burkholderia pseudomallei]

MDARYPGDSNYRFAQILCFNAETYFRGASPCTRKYRARRSRFAVRAPQARASEPRAAVPDARFASPRRFP

LEWCPSATGAQAAATGAAARHGMKLASRGWRAPRARVPTTSKHPPACAAPSTRTERPRPPPAVSFHRNRG

STVAKLDHRNQSRYWHSPGISGVDLLLADFTTHDYAPHVHDSLVVAVTEVGGSVFKSRGQTRLAEPNAVL

VFNPCEPHSGRMGGSSRWRYRSFYLAEAGLARVLTLLGMAQPRFFTSNVLDDPQLVEQFLTLHRAMDEQD

DLLRQQELLVSSFGTLFSRHGLQAGLGAGPGFGTKAGLPALKPALDLMNDCFDHALTLEQIAGAAGLTSF

QLITAFNRVIGLTPHAYLNQLRLRAALRELQAGHSLADAALTSGFYDQSALCNHFKRTFGMTPMQYTRAL

APGKRALAPIGI

>WP_183155473.1 alpha/beta fold hydrolase [Burkholderia pseudomallei]

MFAGGHGALLTGARRAVRGAGRRYAAAVRRAPARSSTKQHEAARSSTKQHEAARSGTRRHKAAQGNTEQH

EATGSSTKRCRIGMRGGRLPGPRTARRHSARHAVPHGAAFPDNPEGGLIMSHPINARRRRLLGTTLAGIS

LVDLGLGGFAHAQSAPRATSAAGVARAAGGASFDTIHQIDAGVLNVGYADVGPKGGPAVILLHGWPYDIH

SFAEVAPLLAAAGYRVIVPYLRGYGTTRFLSADTPRNGQQAVTAVDIIALMDALKIDRAVLGGFDWGART

AGIIAAAWPQRCHALVSVSGYLIGSQEANRKPLAPKAELAWWYQFYFATERGAAGYAANRDDFNRLIWQL

ASPRWRFDDETFARSAASFRNPDHVAIVVHNYRWRLGLAQGEPQYDALEKRLAAAPAIAAPTITLEGDAN

GAPHPEPAAYAKKFTGKYRHRDIAGGIGHNLPQEAPKAFADAILQAAHL

>WP_182910307.1 hexose kinase, partial [Burkholderia pseudomallei]

GKRQAASGKRQAASGKRQAASGKRQAASGKRQAASGKRQAASSKQQAASSKQQAASSKQQAASSKQQAAS

SSRSSPGLMVRRRIERKHCGACPPRSTREPRTRASRCVVFPSRIPTLKPNVTMPEIVTLTPNPAIDVATC

VERVTDTRKLRCGPARRDPGGGGINVARVLTRLGADCSAVYLAGGGTGLALRGLLADEGVRAHGIDIAGE

TRENFSVLETSTGREFRFVLPGPALAAHEWPRCVEALGRLADASRYLVMSGSLPPGMPDDCYARLARRAS

ARGVRTVVDTSGPALAAALDAGVYLVKPSLGELRALTGLPLEDDGARLAAARAIVAGGRVQIVALTLGDA

GALVVSRDDAVRLPGVKVAVRSAIGAGDSFVAGLVAALNRGANVADAARHALAAASASLLSTGTALGTKE

DIARIYRELPGTIDA

>WP_076823316.1 acyl-CoA synthetase [Burkholderia pseudomallei]

MHRNGIRKNDRRRTMLPHAAHYAELVERFAWRVPAHYNIGVDACDKWADGSGRLALIHEHAHGVVARYTF

DELRSSSNRLANSFARAGVKRGDRIGILLAQGPETAIAHLAAYKLGAIAVPLFTLFGADALEFRLGDSGA

VALVTDRAGYEKIAPLHASLPSLATIYCIDGAPDLAEPGVLAFDAALAAESDTFRPADTSADDPALIIYT

SGTTGKPKGTLHAHRVLLGHLPGVEMSQNLFPARARLFWTPADWAWIGGLLDVLLPSLHHGVPVLARRFE

KFGGAAAFDLLARHGVTHAFLPPTALKLMRAAVARPRERYALALESVASGGESLGAELVSWGRDAFGVTI

NEFYGQTECNVVLSSCSALFEPRAGTIGKAAPGHRVAIVDDAGNALPPGVTGNIGVRAPDPVMFIGYWRR

PEATREKFAGDFLLTGDLGIADADGFIRFVGRNDDVITSAGYRIGPGPIEDCLLEHPAVRMAAVVGVPDA

VRTEIVKAFVVLNAGYEGSAALARELQTHVKTRLAAHEYPRALAFVDSLPMTATGKIIRRALRDI

>WP_004530656.1 molybdopterin oxidoreductase family protein [Burkholderia pseudomallei]

MQRANMDDRARSGGEPREVKTTTCYMCACRCGIRVHLRNGEVRYIDGNPDHPLNQGVICAKGASGIMKQY

SPARLTQPLMRKAGAERGSAQFEPVSWDVAFSVLEQRLAHLRATDPKRFALFTGRDQMQALTGLFAKQYG

TPNYAAHGGFCSANMAAGMIYTVGGSFWEFGGPDLDRAKLFFMIGTAEDHHSNPLKIAISKFKRAGGRFV

AINPVRTGYAAIADEWVPIRPGTDGALFMAMIRELIETGGYDRDFVTRYTNAAELLDMRAEADTFGLFVR

DASRPERNPLFPQNHLWWDLGSGRAVAHHTRGATPALDGRYALDDGTPVAPSFALLRERVAECTPQWAER

ITGIPAATIRRLAHEMADVARDHKITLPIRWTDAWGETHDTVTGNPVAFHAMRGLAAHSNGFQSIRALAV

LMSLLGTIDRPGGFRHKSPYPRAVPPSAKPPNGPDAVRPNTPLAAGPLGWPAAPEDLFVDEQGGPVRIDK

AFSWEYPLAVHGLMHSVITNAWRGDPYPIDTLMIFMANMAWNSSMNTVEVRRMLADRHDNGDYKIPFIVV

CDAFQSEMTAFADLILPDTTYLERHDAMSMLDRPISEFDGPVDSVRIPVVPPTGECKPFQEVLIELASRL

KLPAFTNADGTRKFRDYPDFVINYQTAPDSGVGFLIGWRGEDGGDALVGAPNPRQWDEYEKHGCVFHYTL

PDTLQYMRGCNGPYLKWAVEKGFRKYDAPIVIHLYSDVLQKFRLAAQGRTRGRQPPEHLRARIARHFDPL

PFWYEPLELGATDLQRYPLAAVTQRPMAMYHSWDSQNAWLRQIHGENALFVNPKVARDAGIDDGGWIYVE

SQWGKVRCRARYSEVVEPGTVWTWNAIGKAAGAWNLGPDANESQRAFLLNHVITDELPGEGAHAPRISNS

DPITGQAAWYDVRVRIYPAEADADHTLPQFAPMPALPGVTGAVRRIVQTYFAGRGEFAARLRDAAKRR

>WP_004524503.1 transporter substrate-binding domain-containing protein [Burkholderia pseudomallei]

MKMNQTLLALACCAGLAHAQTAPQPGAPASRLDDVLARGTLRVCTTGDYKPYTYRREDGAFEGIDIDMAA

SLAKSLGVKTAFVKTTWPTLTDDFVAKCDIAVGGISTTLERQKRVFFTQPYVSDGKTPIVRCADAERYQT

IAQIDQPQTRVIVNPGGTNERFAKQHLTRAKLTVFPDNVTIFKQILAGNADVMVTDASETLLQQKLNPGL

CSVHPDKPFQFGEKAYMVPRGDVVFQQYVDQWLHLARETGELRAISDKWLK

>WP_004523737.1 MULTISPECIES: superinfection immunity protein [Burkholderia]

MQMEILIQIVGSVVAIALYFLPAIVADRRHRDDKLTIALFNALFGWTGIGWLLTLYWSLQPNATDDFVSD

VRLKRRALSMKTFSTGLVERVQRRIAAQEQWADKQGTR

>WP_004522169.1 D-glycero-alpha-D-manno-heptose-1,7-bisphosphate 7-phosphatase [Burkholderia pseudomallei]

MKNRALFLDRDGVINRDDGYVFEIEKFVFLDGIFELAGAAKALGYLSIVVTNQAGIGRGYYSEDDFFRLS

DWMKGVFATEGAPIDGVYFCPTHPEHGIGRYKVESRFRKPNPGMILAAQHDFDLDLGASLLVGDKESDIQ

AGSTAGVGTTLLICDRDASRVATTASAVVRNPRDVIPFLTGPGPDAGSF

>WP_004190574.1 MULTISPECIES: DedA family protein/thiosulfate sulfurtransferase GlpE [Burkholderia]

MLHDLVARFGPLIVFVNVLAAAIGLPVPAMPTLVLFGAMATLHPDALGAQLAPVLALAVLAALIGDTVWY

VAGRHFGGRALKTLCKLSLSRDSCVKKTERFFGRWGVRVLAVARFIPGLSLISVPMAGALGTRYRVFVGY

DGLGALLWAGCGVAIGFAFAKQIDWLFAGANQLGRAVLVVIVALLAVYTAVRWMRRRALIRQLANARIDV

DELERLLRAEPAPVVFDARSPEHRKLDPYAIPGAQFADERDLRDIVAHYPATQKFVIYCSCPNEVSAALM

AQRLKQAGFADALALRGGLDAWRDTGRQLIELAPQPPSEAPVLAAAPKTA

>WP_004525408.1 MULTISPECIES: LysR family transcriptional regulator [Burkholderia]

MTIDAHNLNDLMYFSQVVEHGGFSAAERVLGISKSRLSRRLTELEATLGVRLLQRSTRKLALTEAGQLFY

QHCQAMLSEAQAAMNAVQQLRSAPRGTVRVSVPVTVSQTMLSRLLPEFLRRYPEVRVQIRVTNRVIDLFE

DSIDVALRVRSEPPQSANIVVRPLWRTEQMLVGAPSLLQQNAPPLAPDDLAGFDTLDTPSGDGRHVFNLI

APDGTRHTHEHEPRLVTADLMTIREAVLDGVGIAALPESMYGNALRAGQLSPVMPGWTLPVPQLYAVFVS

RQGMPPAVRAFVDYLVEKLDNERYEEPGCPERGAKDTQTKISI

>WP_004198488.1 MULTISPECIES: bestrophin family protein [Burkholderia]

MIVRPREHGFRMLFVWNGSVLKSILPQLALMSAVSIVALLTNGRILGEKVPLNPTPFTLAGLALAIFAAF

RNNASYDRYWEARKLWGGVLSAARALTSQALGYDALADGASFARATAGFVYALKHQLRGTDPTHDLRRRL

PADWLDPVLAAQFRPVAILHALRGRLAGRHRDGALTDAQLWMLDAQLNELAAKLAGCERIASTPIPFPYH

VLLHRTVYAYCVMLPFGLVDSIGIATPFVSVFVSYTLIALDAIAGEIAEPFGDGPNHLALDALTRQIERS

LFELAGLPLPDEMRAGPNHRLS

>WP_004194086.1 MULTISPECIES: nucleotidyltransferase family protein [Burkholderia]

MREAIILAGGFGTRLRTVVSDVPKPMAPIAGRPFLEILLTRLSEKKFSRVVLSVGFMAEKIMSHFGDRFA

GIDLAYSVESDPLGTGGALKATLPYCEGDHAFVFNGDTYLDLEVDELDDGWQTGGFPTIVARQVPDTGRY

GRLVVDGGRVTGFAEKGVSGPGLINAGCYVLPKDILAGETAEAFSFETDFMSSAVQSRRFDVFVTRGQFI

DIGVPEDFYRAQDELSGICK

>WP_004525522.1 MULTISPECIES: peroxiredoxin [Burkholderia]

MPIINSPIKPFKATAYHNGDFVQVSDETLKGKWSVVVFYPADFTFVCPTELGDLAERYAEFQKLGVEIYA

VSTDTHFTHKAWHDTSDTIAKIKYPMIGDPTLAISRNFDVLIEEEGLALRGTFVINPQGEIKLCEIHDNG

IGRDAGELLRKVQAAQYVAAHPGEVCPAKWTPGADTLTPSLDLIGKI

>WP_004186345.1 MULTISPECIES: peroxiredoxin [Burkholderia]

MSLRLGDIAPDFEQDSSLGRIKFHEWLGNSWGVLFSHPADYTPVCTTELGLTAKLKGEFEKRNVKVIALS

VDSVESHKGWIDDINETQATSVGFPIIADGDRKVSELYDMIHPNANETLTVRSLFVIDPNKKVRLIITYP

ASTGRNFDEVLRVIDSLQLTDHYKVATPGNWKDGDDVVIVPSLQDPDELKQRFPKGFKAVRPYLRLTPQP

NK

>WP_180986282.1 4a-hydroxytetrahydrobiopterin dehydratase [Burkholderia pseudomallei]

MTNNYGRMADTARRRANGRARAAIGRGTKMIHKLTSEERKTQLESLHHWTAVPGRDAIQRSLRFADFNEA

FGFMTRVAIKAQEMNHHPEWFNVYNRVDVTLSTHDANGLTERDIKLAHFIDEVGKHAKAA

>WP_180980633.1 glutamate-5-semialdehyde dehydrogenase [Burkholderia pseudomallei]

MASLTMDIDQYMTDVGRRARRASREIARASTAAKNAALEAVARAIGRDADALKAANARDVARAKDKGHDA

AFVDRLTLSDKALKTMIEGLRQVATLSDPIGEMSNLKYRPSGIQVGQMRVPLGVIGIIYESRPNVTIDAA

ALCLKSGNATILRGGSEALESNTALAKLIGEGLAAAGLPQDAVQVVGTADRAAVGKLITMTEYVDVIVPR

GGKSLIERLINEARVPMIKHLDGICHVYVDDRASVAKALAVCDNAKTHRYGTCNTMETLLVARGIAPAVL

TPLGRLYREKGVELRVDADARAVLEAAGVGPLVDATDEDWRTEYLAPVLAIKIVDGIDAAIEHINEYGSH

HTDAIVTEDHDRAMRFLREVDSASVMVNASTRFADGFEFGLGAEIGISNDKLHARGPVGLEGLTSLKYVV

LGHGEGRE

>WP_152768855.1 MULTISPECIES: glutamine ABC transporter permease GlnP [Burkholderia]

MPRRSERHRELNVQFDWSAIWAALPDLMDGVRLTVFIAFFGLAGGFLVGMIAGMFRAYGPAVLNVLAQVY

IELIRGTPIVVQVMFLYFALPLLAHVRIDGLSAAVVAITLNSGAYLAEVVRGALLSIPKGLTEAGLAMGL

SMPRVLVKIVGPLAFRRLIAPLGNQCIVSLKDTSLFIVIGVGELTRKGQEIIAGNFQAVEIWTAVAAIYL

VLTGVMTLTLRFVEKRMRIL

>WP_080109811.1 hypothetical protein [Burkholderia pseudomallei]

MRASPRASGDRSPQRALAVDRAHGARAFVTCGALNSYNRRLFRRAARRDESRRRRATGKHHEKNARRGFG

EMNNQNTMNKFNNGPAESRKKTDARRRSAGARGATSAARTAHAQRDAKRSPDGVATGRGVTSAMPDGSVQ

LGRPAASQPRVEVATEAASASGDPSDPSRSSGARRSPHALDAAGSAAAPCAAEPPRSAAPGAARMPRVHR

AFASLQTHYKKLDTAQKLYLNCALALLLYGVTYPLHAWWNFVGLALLSLAFWFAGMVCDALTLYKRIYAT

HIGKGLLLLAFTGFTNFAIALANQRINAFAGVDPSKFPHTEIFVAILSIPFIALLILMMLYTASVLFLPV

YGVFFIAMDAKAKAFFSAGLMKQDDSEYPVITGIFRFVSFFVFAGVVLFFAKRMLGDYDRFMTSTAERSV

YQLEMYEKSPCDVPAGARVAFLDDGNIVYALRVGAELKFFPQRCKRSGQ

>WP_076904040.1 hypothetical protein [Burkholderia pseudomallei]

MQRCVTTCHGRPRGPHVRARAGGAIVRYSPPAASRRITTGAAVFASFEPTHTGFVAEIDGCRCSIEGAPS

PIAERIDWRWTIAQPTPENPDGSDPYQYEVLATGETVTPLQAEQQIVAWLEAHPPEDA

>WP_076804406.1 acid phosphatase [Burkholderia pseudomallei]

MRASPVRAPTRGGPSNQPEDRGAEVTDKHPPSTDLPEDPDRRRVLGGIAALGASVALAGCETAVPAAPRS

AAELRLDAALRREVRNIVVIYAENRSFANLFGDFPGVQQPLGAVSPERYAQLDRDGKTPLATLPKIWGGL

VPQAQVVDGKRYMIGEHDIVGLPNAPFVIPDAQGKPLPNGVITRDLWHRFYQNQMQINGGRNDQFAAWAD

SGGLVMGHYRNSAETLRLWNLARGYTLCDNFFMAAFGGSWLNHIFLISAQAPLYPDVHQSPAKHLVSVVE

GDDPAGTRLKLAADSPASALDGPPKFENDGLFTPDGYAVNTMAPPYQPSSVRPPVEGNPAYADPSNPRVL

PPQHYATIGDRLSEKGVDWAWYGGAWQYALEHRDTGSVPDFQYHHQPFNYFASYAPGTEARRRHLRDAGL

GDDPSTNHLIADIDAGRLPTVTFYKPQGNLNMHAGYADVASGDRHIATVIEHIMRGPQWAHTVVILTVDE

NGGWWDPVSPPKGDRWGPGSRIPAMVISPFAKKGYVDHTLYDTNSILRFISRVHGLAPLDGVLARERAFA

ARGAQPPGDLTEALDLG

>WP_073699448.1 MULTISPECIES: DUF2827 domain-containing protein [pseudomallei group]

MVIDSIRSSSTGPRDAPGKRLAVGISLFARDGQAIWENGIHQNIAFLAMMLKQSDRIGPVYFLNGGDAKA

LPAGLDLGGLDVPLVQPRDVSHALDVVIEMGAQLPVDWLKHMKALGKKLVVCFVGHTYSGLTETPMFNRP

SGHIFNGGPFDEVWILPKSEKIDAPLLRTLTRAPVHVVPHIWSPYFIERRARELEAAGLRFGYAPGRAKW

RLSILEPNISVVKTCHYPMLAADEFHRAQPDAVEHLFVVNSLHMKEHPTFVHFANSLDLVREHKATFEPR

IDLPSFLAQHGDAVISHQWENPQNYLYYDVLYGGYPLVHNSPMLGDAGYYYPEFDSAAGGRALQHAWRHH

DAHLDDYRRHADALLQGVSIDNRRNLDAYVARLFA

>WP_073699289.1 MULTISPECIES: EAL domain-containing protein [pseudomallei group]

MNGTSSMREQALGMNDGGGQAAELRPALDVWPILLMVSVLVCVVIVSLAVVSFLRVYIGGESTWSKSQKD

AVIYLIRYAETGDERAYKLYEAAIDKPYRLSLARAALQRKPPDRASAREAIIASGIDPTDATAAALLLPA

LSWVAKINYALQCWMQADVELAEFRSVASQLHALVSSGHRDDARVKALEYRAWEINERVSGLPDRFSKAF

SDEFRSSVALLLVCYFVASMFLMYLALVWARKAALQRLSIQYALDRSEAFAEAALSSVAEAVITVGLAKN

VDFINNAAEQLLGYPAAACVGAPVSSVLMLLDKETGHAVDIVDEFWAREERTPIKREVHLLRRDHSKIVV

QTTISEMGDRVHGHIGYVLILRNMTREQQFLESLAWQATHDVLTGLVNRVEFERRLEIALADTASGKERA

DSMLLMLDLDRFKEINDTCGHAAGDAMLREVTQRFQSCLDDDDVLARLGGDEFGVLLPHGAGSWPSKNKA

ERLRRSLEDFVFLWEGERFTVSVSIGVLELCKAPRNLEMAVKLADIACYIAKERGRNRIQLADPSDLQQA

RHVGDVQWSRRIKTALENDGFRLYVQPIVHTRPDSRTPERAEVLLRMMDSAGRDISPAAFLPAAERYGLM

GLIDRWVIRTVFRKLSALPAREYHEYNVNLSGASISDERFLEFVIAELLSSGLEPSVICFEITETIAVKN

LELASRFMGELRTIGCRFALDDFGAGMSSFRYLRSLPIDYLKIDGEFVSNMMSDRVSYGVVSAINEVAHS

MRCSTVAEHVESEVELAMLRELGIDFCQGYFFAQPSPWREGGY

>WP_050020039.1 bifunctional enoyl-CoA hydratase/phosphate acetyltransferase [Burkholderia pseudomallei]

MCSRNKECRVDREILQNRPFDSLAIGDSASLVRTASRDDIDLFAAMSGDVNPAHLDAAFAAHDLFGHVVV

HGMWTGALVSAVLGTKLPGPGTIYLDQTLEFRHPVAPGDTITATVTVAEKRPEKRIVLLDTRCTNQDGHV

VLRGTATVIAPDTPITWERVPGPEVAVRRHDRYETFVRDAREQPPLRTAIVHPCSPDALRAAIDARDANL

IEPILIGPQAKIRAIADAAGVDLAGVPIEAVEHSHAAAARAVEMGGAGEVAALMKGSLHTDELLAAVIGP

ASRLRTERRISHVYAMDVPAYPKPLLITDAAINIAPTLEQKRDICQNAVDLLHVLGVERPRVAVLAAVET

VNPKMPSTLDAAALTVMAARGQIAGAIVDGPLAFDNAVSAVAAMSKGIASPVAGDADVLLVPDLEAGNML

AKQLMYFAGADAAGLVLGARLPIILTSRADSLRVRIASAALAKLVAERTRAGALAS

>WP_024428957.1 MULTISPECIES: uracil-DNA glycosylase [Burkholderia]

MAWTEAALEELGLAPAWVRRDAARAADVNEAAAHAAGEAAVAAVARGAQPASPDAPREPARRIARDSGRD

GGGDGGQDGGQDGASGDAGSASPNDAAHAMARVAGAEASAADAVADEHAALARQAGDGRGQTRQAAAESG

ARAAADAPAPAAAPASRTRDATIARGASPDEPGVAGVVGVVGVADEPSVAGGARRARTSGGGADVPARAL

DWTDAAEATRPAAAPAPAALTGGDAGAAASDEDMSWFDLEPVHEPVLPDVAARPAATTPSVAELGWDELR

ARVADCERCRLCEKRTNTVFGVGDEHADWMLVGEAPGENEDKQGEPFVGQAGKLLDNMLRALALKRGENV

YIANVIKCRPPGNRNPEPDEVARCEPYLQRQVALVKPKLIVALGRFAAQTLLKTDGSIASMRGRVHQYEG

VPVIVTYHPAYLLRSLQDKAKAWSDLCLANDTYRSAAPAADPP

>WP_024428537.1 hypothetical protein [Burkholderia pseudomallei]

MGLLFEQSRLERVRTWAMANKQRFRPNGFGRQFALLQEFPDVPDAIWDIKRDVVAHFGLHDLPQEPLYRD

LCGVITEGGAVHPHRDSNQGMLVHTRFNVMVSRPDGGGVPMIDGMLVDVPEGGIFRVDAGLLTHSCTPVI

GARPRIILSFGFLSPLGRFSGLPFCISTP

>WP_023360175.1 protein phosphatase CheZ [Burkholderia pseudomallei]

MNEPIDMAVAGANAGERLADAADLASDRILARIGQLTRTLRDSMRELGLDKHVEKAAEVVPDARDRLRYV

VAMTEQAAERVLTAIEVAKPVQERLQQEAAALDARWAKWYETPIERAEVRGLMDDTRAFLRALPDATGAT

NAQLLEIMLAQDFQDLTGQVIKKIMDMVYLIEQQLLNVLVENIAPERREQFAATAAALAAEQAGGAMTPE

SLLNGPQINPEGKTDVVQDQAQVDDLLASLGF

>WP_023359800.1 MULTISPECIES: hypothetical protein [Burkholderia]

MMQPALSRARRNIAPRPAVIDRAKAAIRSLRPIAFAYLADRAHGG

>WP_020850699.1 MULTISPECIES: DUF2957 domain-containing protein [pseudomallei group]

MSDAFSKGVAMAIAVAPLFAACSGGGGGTPAPIAVPQCSGSSCGVQGPPSSTAANTSLCPADANIGSSTY

LGGAGGGEIVSLNINATTMTYTLKWLESPVPLATGTVTPTRAGTTITGSVAHPPAGTLPTAEQTRCAFVL

LPGSGTAPATNSTYSTAADFNQANPPMILIGFGVAGGGIPGATIQYSGLTIIPGVLQNIGQVPQRHFDFY

PFLGFANTTTDLSKLPGTYNALVYHTVPSGNYAAKAIASNETFDANGACTSTSASGCMTTGNPWTASGNG

YFNSTQAPQILPQTQLPLIGATGKSAVAHMVLGQLNGATVPVVVRTGNVNLGTPPLHTDAQVDDESGIAV

LGLAQAIASGGIDGGYAGADSNFKYTATVIKGTTGTFVNPSTQQAETGFTLDYGQSTPGLLGVTTTDTSA

PGFVIASGGLYAALVQGTVNGGITQSSAIAGQTPSAPYFGVGAQVSK

>WP_017881046.1 MULTISPECIES: PHB depolymerase family esterase [Burkholderia]

MTKSLTKFWLGGVKRMLHMPNARTSASALKAAQKMTADWPFAETMAAAAAASVASVAASADTVPPAARES

RVRPRAAAWAGGEWIRADHPLPPAFGRFVKHLEYGLYVPSGAKTLGLPLVVMLHGCKQDMNQFSQGTRMN

LLADRYGFAVLYPEQSLSAHAHGCWHWYEDTVHGGRGEAQAVVALVDALVAERGFDASRVYAAGMSAGAG

LVSLLALHFPRRFAAVALHSGPAFGDAHSGITAMDVMRRGLYRDPAAVVDALVEPGAHPGMPAFIVHGDD

DRVVVPKNADELAVQFLRLNGLADADGEPAGVERFETRAADVRTLDYRRDGESVVRLCRVHGLAHAWAGG

DDSVPFHSAAGPDASELIWSFFASRERAAVAS

>WP_017880699.1 MULTISPECIES: oligosaccharide flippase family protein [Burkholderia]

MLRRSKTAPAVGRPGHLAHVAAPRAMLKSRLSNPDVAKAVANLAWLGLERLTQIGVAIVVSGLLARYFGP

DTFGKWQYANTLLLVLAPLTWVCGAEILVPTIVQRTGAQLGAVLGSAFALRFTVSVAALALTWAAIALGA

FDPLVGAMLAGLAVTLALREPFVGIVNAWLQSMTYSKPQLLASIAAALAKMALVWLLVRAAAAPARFGWL

WALEAAAIAAALIWYYRARNGGTLGWRVERPLVREFATTGTVFWLGLVCMYLFLKLDRLMLERYVSFAEL

GRYAAAQQLNENWITLALMLAQTIAPAFVYRVQNAPQLRRNVWRLIGMTAALMIAGALVLDLLAGFIIRK

VFGPGFETSVDVFRWAVWLSVPAGIEAIGNLVVLKYQAKFVLLAKWSAALALAALVNLIAIPALGLYGAL

AGLAAGYLAAAAVNFHYIRLKLRP

>WP_011205743.1 pilus assembly protein [Burkholderia pseudomallei]

MNRFSLPPRPSRMRGAVAVEFAIVMIPLVLLATGVAEFGRAIYQYEALTKATRDAARYLSTYLPTDPAYP

LAQAQCLAVYGSTTCGSTGSELAPGLATSMVVVCDAAHAPDCSDSSDPAQFANVPTYDTNNGSPDPASLA

GSMNLVEVKIKGYQYRPIPAFPGLPNLSFGNIVTVMRQVS

>WP_011205699.1 NAD(P)H-binding protein [Burkholderia pseudomallei]

MANLDMALVVGAAGGIGGEMVRALRGAGWRVRALARSAPPAGDGAADIDWRLGDASNPHDVAEAARGCAA

IVHAVNPPGYRRWSEWVLPMLENTIAAAKRERATIVLPGTVYNYGPDAGGLLREDSPQHPLTRKGAIRVE

MERRLRDATEAGARVLIVRAGDFFGPGARNNWFSQGLVKPGRPVRTVRAPGRAGVGHQWAYLPDVAQAMV

ELLARRDALDAFSCFHLAGHWDADGTRMSAAIRAAAARRSGVEPRVAAFPWWLVTLASPFVETLREMREM

RYLWREAVRLDNARLVAALGREPHTPLEAAVETTLDALGCLNGRASRAAPSVR

>WP_011205572.1 MHS family MFS transporter [Burkholderia pseudomallei]

MRRNALTRGIPPSPPARQPRRAAGAAFVGTMIEWYDFYIYANAAALVFGELFFPSHDPFTSTMASFATFA

VGFFARPLGGLFFGHLGDRIGRKKALMATLALMGAATVCVGLLPTYERVGLLAPVLLVLLRIVQGIAVGG

EWGGAVLMAGEHAPYGRRTFFASFAQLGSPAGLILSLVAFRAVASMEHGAFMSWGWRLPFLASIALLAVG

VFVRLKVDESPEFAHEKAMSRTVARPLAEVLRTSRAMLLFCLCANTIGIAGLYFTNTFMIAFTTQHVGVT

KSLILDCLFIVAIIQFVTQPIAAWLGGKLGDARFLKLAALFAMASPYPMFALVQTGRLAPMVIGIAIATV

FLAGFYSVIAGFVSGAFPTRVRYSAISISYQMCGAIAGGLTPLVGTWLAHRFAGQWWPLAVFYTCLAALS

LGGVIALDARRHRRADVADAVLTR

>WP_011205321.1 MULTISPECIES: hypothetical protein [pseudomallei group]

MADSFNRRRRRHMSYVALEVLTEDANRYSLPELIGVGGVSPDVPHICEMLLADAQWPTIQAYLDRQELPY

KFARPSTGRRVGRNNPCW

>WP_011205271.1 porphobilinogen synthase [Burkholderia pseudomallei]

MSQKFTEGNCKVSSVTYPSHSAMSIHPLHRPRRMRRDDFSRRLMRENILTTNDLIYPVFVVEGTNVRQPV

PSMPGVERVSIDLLMGVAEQCVELGVPVLSLFPAIEPSLKTPDGREAANPEGLIPRAVRELKRRFPELGV

LTDVALDPYTSHGQDGVLDEAGYVLNDETLEILVEQARAQAEAGVDIVAPSDMMDGRIGAVREMLEREGH

IYTRIMAYSAKYASAFYGPFRDAVGSASNLGKGNKMTYQMDPANSDEALREVRLDIDEGADMVMVKPGMP

YLDIVRRVKDEFRYPTYVYQVSGEYAMLKAAAQNGWLDHDKVVLESLLAFKRAGADGILTYFALDAARLL

RAQK

>WP_011205259.1 MULTISPECIES: hypothetical protein [pseudomallei group]

MGHLAINWRKAMQELNQQEIDLVGGATVGGGLIGGLTGLIGAYGGALYDLGANLTGDLVGFATGVVQIGE

TLVGGTLSNIGSIFTGTSH

>WP_011205243.1 outer membrane protein assembly factor BamE [Burkholderia pseudomallei]

MIPRSLLQLQRSRVRSTVIAAVAVVGLAACSSYDSVTQRIAQSITPYRITVVQGNFVSQEKASQLQVGMS

REQVRALLGTPLLTDMFHADRWDYLFYFKRGSTSVVQQRDLVVNFAGDRLAGWSGAENLPSELDLLADID

GDRRGKKARAAAAARAASAPEAAAAAPEAMQAAEQNDANAQAARAANRATAQVPGQSGAASRFAPSAQMA

PNAPTPGGLPPGAAPAIQPQFQFHRPPPPPAQGGANQPVGPQGADALPNQPLTAPASGAQGTGG

>WP_011205218.1 KpsF/GutQ family sugar-phosphate isomerase [Burkholderia pseudomallei]

MRRCAPQYSPPFSMAHDGSQMNHHNYLDSARQVFDIESRALASLSARVGDSFGDAVDAILRSSGRVVVCG

MGKSGIIGRKIAATFASTGTPSFFMHPGEAYHGDLGMVTSADTFLAISYSGETDEVIKLIPFLKSNRNYL

VALTGNARSTLAQAAHSHLDAGVEQEACPLQLAPTSSTTAALAMGDALAVTLMKARGFRPENFARFHPGG

SLGRRLLSKVDDEMTVDGLPFVDERAPAIDVLQAMTRGRLGLAIVRRETGFGIVTDGDVRRAIEAYGDTL

FRRAASDLMSADPAMVPLGTRVEDALLMMEARRINALLVFDGEDVVGVFKK

>WP_011205177.1 MULTISPECIES: hypothetical protein [pseudomallei group]

MLRRIAMSKIRAMLIGAAVAAMATSAFAQTGGVATQGSAGGSADVLGQKAGASAQLNAGGDVSAASPEAA

ENAVKHSAKKHVKHAKAQAKAKADEAAELGADAKTKASGAIEGAAGTAGNAVGGVTNAAGNVVGGATNAV

GGVAGAAGGLAKGVAGGANGSVSVNGGMKAGAGE

>WP_011205167.1 Fe-S cluster assembly ATPase SufC [Burkholderia pseudomallei]

MTNEGIAMNRSEPLLQVFDLSVEVAKRRVLQSVSLAVPAGALVVLMGANGSGKSTLGMTLAGHPAYRATH

GHVRFGGQDLLAMSVQERARAGLFLSFQAPPDIPGVKNNLFVRTALNAVREARGDTALDALDFLGDARAA

AAQVGLSDAMLNRSMNEGFSGGERKRNELLQLALLRPRLAMLDEIDSGMDVDGVRAAVALIGRLREQGTA

FVIVSHYLQMIEALAPDTVLLLDRGRIAESGDLALARDIAAKGFARSDALAQA

>WP_011205090.1 MULTISPECIES: hypothetical protein [pseudomallei group]

MDESATMERNRRRRRAMFPTLSPEAIEALKWIDQFGSGRPLPVGFRLALEELLNDGFVYQSGPDRVDITD

DGKAYLSEAYD

>WP_011205083.1 MULTISPECIES: ABC transporter ATP-binding protein [pseudomallei group]

MRRMAAEPSTLFSNPADAHACATSGKLVRVDRVTLEYRHRERVVRATQQVSFDVYGADRFVLLGPSGCGK

STLLKAIAGFVAPVEGAITLAGEPVRGPGADRIVVFQEFDQLAPWKTVRENVAFALRAARRLSRRDAAER

SRAALDKVGLAAFADAYPHTLSGGMKQRVAIARALAMEPRVLLMDEPFAALDALTRRRMQHELLRLWDDA

RFTLLFVTHSIEEALVIGNRVLLLSPHPGRVRAELNSHHFDERSVGRADFQRTAERIQRLLFDESGARA

>WP_011204944.1 MULTISPECIES: DNA-deoxyinosine glycosylase [pseudomallei group]

MMLRGFPPVASPGTHTLILGSFPGEASLAAAQYYAHPRNQFWRLLGAVLGEPQLHALPYDARLARVLAHG

FGIWDVLAACHREGSLDAAIRHAQPNDFASLREVAPKLTKVCFNGKTAGRFEPTIRAAGFDTLVLPSSSP

ANAMLSFEQKLVFWQRIVG

>WP_011204906.1 purine permease [Burkholderia pseudomallei]

MLLLRFVNAHNVEPPSKQNHGVRQMQSNTVHPCDEVLPSGKLLTLGLQHVLVMYAGAVAVPLIVGGALKL

PKDQIAFLISADLFACGIATLIQTLGVWLFGIRLPVIMGCTFAAVGPMIAIGTNPGLGILDIFGSTIAAG

AIGIVLAPMIGKLLRFFPPVVVGTVISVIGLSLMEVGINWAAGGVGNPNYGDPVYLGLSFIVLALILAIN

KFGRGFVANISVLLGMIAGFAIAFAAGRVNTDGVAAAPWVGFVAPFHFGLPHFDPLSIATMVIVMFVTFI

ESTGMFLAVGDMVERPVDQETLVRGLRVDGLGTLIGGIFNSFPHTSFSQNVGLIGVTGVKSRFVCATGGM

ILVLLGLFPKMAQLVASVPPFVLGGAGIVMFGMVAANGVKVLSKVDFVRNHHNLFIVAVSVGLGLVPVVS

PNFFSKLPAAFAPILHSGILLASVSAVVLNIVFNGMKGEKDARCDIRRAGRDFDGQPADLH

>WP_011204861.1 hydrolase [Burkholderia pseudomallei]

MENAMQIHEVRIPIGKVELNGLLAAPEQASGIVVFAHGSGSSRLSPRNQEVAAVLQRAGLATLLFDLLTL

EEQRRDAVTAEYRFAISFLARRLVSALDWLRERPDVGALPVGLFGASTGAAAALIAANARGRVVRAVVSR

GGRPDLAGDALPRVRVPTLLVVGERDDEVLRLNRVAAGWLIGESKLVVVPGATHLFEEPGTLDEVARVAA

DWFVAHLGEGRPSPEGARR

>WP_011204837.1 hypothetical protein [Burkholderia pseudomallei]

METAMGFYGPEPFERATATYVWLGLRVPGALIVEVEGNAPRYTTGIQLVRDPRFVGGLKIDVMGWTGPLS

SGTQSYKVRHTFQGVFHPTIVVHGSNKTEVVEVKQIPHEEADAFLQALDAA

>WP_011204812.1 MULTISPECIES: nuclear transport factor 2 family protein [pseudomallei group]

MQSRSMAMVAKGIDAIRALERDRFRAMVDGDGEALDALLSDKVCYVHTNGKRETKQQFIDAITAGRRRYR

QIEIQSQDVLPVGDATYVVAGRALIEMETNNGGLVFSIAYTAVQTHESGRWRLLAWQATRCATDA

>WP_011204175.1 MULTISPECIES: DUF2844 domain-containing protein [pseudomallei group]

MQSLNSRRNGVRSSRFRRLAATAAGFMCAWLAAPAHAGLGGAPMTPPAADTAATVRSIQRSIRAAGGAST

AAVGYTVRETTLGSGTVVREYVSTAGTVFALSWQGPVAPNLSDLLGAYFPQYEAGVKASRAAHGPRAPAA

IDTGGLVIHTGGHMGSYSGQAWLPQALPAGFSTNDIQ

>WP_011203979.1 MULTISPECIES: peptidoglycan DD-metalloendopeptidase family protein [pseudomallei group]

MSMLRAMQNNRSKVPLSLAQRAICMAAFALLGACATRLDQAPVVDRSGSLGTTAATQPPAPLPPAPPGYY

RVKPGDTLYRIALENGQNYRDIAAWNNLTNPNQIEVDQLLRVSPTAGGATAAAPAAGAVVTPPLSSGPSN

GAAAVPPIYGSGSTASAAPSAPAASASSEPSAPAANGNVSFAWPVRGALLNTFDDSKNKGINIGGPAGEA

VKAAADGRVVYAGNGLRGYGNLIIIKHDATYLTAYAHNRALMVKEGDAVTKGQKIAEMGNSDSDRVMLHF

EVRRQGKPVDPLKYLPPQ

>WP_011203961.1 MULTISPECIES: alkaline phosphatase family protein [pseudomallei group]

MHAGGPQRSPLVANSLSRIEHIVHLMLENRSFDQMLGFLYTDDGNRSPTGQPFDGLTGNESNPDDLGRPV

GVYRIRATDPHPYLMPGADPGEGFQNTNYQLFCDDHPAPGAVPTNQGFVVNFKSAIATDQSRHDKDALPG

TTPEQIMGMYTPELLPVLSGLAKGYAVCDRWFASAPTMTMPNRAFALAATSQGHLDDHVKIFTCPSIFGR

LSDQGVDWAIFGYNRDPLTRHDFPDTQNADDSHFGHFRDFQARAASGTLPAFTFLEPSWDASGNSQHPNY

DVAAGEQLIHDVYYALRNGPGWNSTLFIVTYDEHGGNYDHVAPPSGATPPGDGTVGEFGFDFTRFGVRVP

AVLVSPLIAAGTVFRSAAGTIDHTSVLKTIGERFGTAPLTARDRAAPSLGDALTLASPRAASDDPLAGVT

VPVSRVSHPNAAMPSKLDKLQAARIAALPLRNEKGYYEEADTPLASSAELSNFIRDRGAAWSQHRQRQQQ

RRQQQQRQQPRSKPRR

>WP_009941446.1 helix-turn-helix domain-containing protein [Burkholderia pseudomallei]

MKKPLRGTGRPGVAILLPPRLLGGYVFLTQELLLLAGTMKARSLDVVNSRLFDIAILSHDGRPVPTIGGL

DVPATAALSDADAHEVVIVPAQFMPDAQIGALERVFIDWLERRYAAGALVVGLNAAPLLAKAGLLDGRGA

TGLPSERTLFARHFPAVRYTPSKPLVVDGRLITVSGINPAVDACAYVIDYCFGAGVSQRLLRVALTQSLP

SYEHMAVWAAQYKRHGDAPVLAVQDSVERALAHPPTLAELAARAAMSERTLSRRFAAATGLTLRRYVAAL

RVELAAFLLRTSRMTLEHIADECGFASASALSHAFLAGQGCSPIQYRNRHRPDARREPAGGGGTAAGSGE

GAAS

>WP_004556827.1 MULTISPECIES: pyridoxal phosphate-dependent aminotransferase [Burkholderia]

MNAPHDMPTTPTLLSRLPEVGTTIFTVMSALAAEKGAVNLGQGFPDFDCDPRIVDAVAHAMRDGRNQYPP

MAGVAPLREAIADKIERLYGRRYDAATEITVTAGATQALLTAILCAVHPGDEVIVIEPTYDSYLPSIELA

GATPVFVTLNAPDYAIPFDKLAAAITPKTRAILINTPHNPTGTVWREADMRALEDIVRGTGLLIVSDEVY

EHMVYDGARHESVARYPELAARSIIVSSFGKTFHVTGWKIGYVAAPAPLTAEFRKVHQFNVFTVNTPMQF

GLADYLRDPEPYLTLPAFYQQKRDFFRAGLANTRFALLPCAGTYFQCVDYSAISDLPEAEFAKWLTTEIG

VAAIPVSAFYHEPHESGVVRFCFAKREDTLATALERLARL

>WP_004553805.1 MULTISPECIES: fatty acid desaturase family protein [pseudomallei group]

MSSGIAPPHSVNYRKKYATEAHALSAIRPWRCAWVIARQWIGIAIAFALPIALVARLTGGTSLAHAFAAL

GAPQRLGVAAALGAAYVYLACKQHALGIVMHDATHFRLFESRRVNELVGNWLCAFPIGMVTSCYRRSHLP

HHLFTNKPNDPYWARLVEDAHYAFPMSRAAFGRILLGDVFGANLRAWWPTLRSWTGWSSVLDNREKLLTP

SERRQFVAFWIGALALAAYFGVLSYFLLLWVLPMFTLSLAFIRLRVIAEHDLEKAGHELERTRHVDGGWF

ERLAIAPLNINYHVAHHLFPSVPLYNLPKMHALLMQEPAFREHAQLWRSYLGRKHGMVRSLLT

>WP_004553287.1 MULTISPECIES: hypothetical protein [Burkholderia]

MMKFLTREIVVLAALDWALLAGCAPVQAPPSSSSAPPASSCCGPITASGQTLRHTLDASDVEHLWLPRQH

VDWSTGKPDPTAEGFKSHCSAFAAAMGARLDVYMLRPPEHSQILLANAQAAWLASDSGRAAGWRELHEAY

EAQAAANRGELVVAAFQSADPKMPGHMAIIRPSLKSNVQLADEGPEIIQAGAVNRLDWNVRDGFARHPGA

WPNGIKYFAHVVPAK

>WP_004552876.1 phenylalanine--tRNA ligase subunit alpha [Burkholderia pseudomallei]

MGSMDLDQIVADAQQSFEGAADITTLENEKARFLGKSGALTELLKGLGKLDPETRKTEGARINVAKQQVE

AALNARRQALADALLNQRLAAEAIDVTLPGRGAGAGSLHPVMRTWERVEQIFRSIGFDVADGPEIETDWY

NFTALNSPENHPARSMQDTFYVDGKDADGRPLLLRTHTSPMQVRYARMNRPPIKVIAPGRTYRVDSDATH

SPMFNQVEGLWIDENVSFADLKGVYTDFLKKFFERDDILVRFRPSYFPFTEPSAEIDMMFEHGKNAGKWL

EISGSGQVHPTVIRNMGLDPERYIGFAFGSGLERLTMLRYGVQDLRLFFENDLRFLRQFA

>WP_004552494.1 MULTISPECIES: 3-oxoacid CoA-transferase subunit A [Burkholderia]

MVNKIFDSLQSAVADVHDGATIMIGGFGTAGMPAELIDALIAQGARDLTIVNNNAGNGETGLAALLKARR

VRKIICSFPRQADSQVFDALYRAGEIELELVPQGNLAERIRAAGAGIGGFFSPTGYGTKLAEGKETRVID

GKHYVFETPIHADFALVKAYRGDRWGNLVYRKTARNFGPVMAMAAKTSIVQVSQVVPLGALNPEHIVTPG

IFVQRIVEVPQAAHAAELAAERAASAA

>WP_004552435.1 MULTISPECIES: hypothetical protein [pseudomallei group]

MTTFRIEAACLPIFVADARGEQVSVGGFAVDDAAAGKRPPAGDGLQRLAQIHRRDLEGFAIFGNRAARDH

EPLFR

>WP_004552394.1 MULTISPECIES: hypothetical protein [pseudomallei group]

MMRFNSTVFLIDPTPRRADGEYMAHARISANRSDGREYDVCRSGDLAGFDLRADAIAHARRWAETWLEDH

FG

>WP_004552355.1 MULTISPECIES: hypothetical protein [Burkholderia]

MTIDNPIEISSVMLAFAALGAIIIGGLLTAMHLKRKYHPNLIGALIGALLCFLLIEALPAIT

>WP_004552260.1 MULTISPECIES: BON domain-containing protein [Burkholderia]

MKEMQSRVPRYTQPHSPHHAAKRFGRLATAVLIALAAACLALPQAAAAAGDGTGGAGAATGGANGSANGS

ANGPNDMTGANTANRATDMNGASSSHSTSAGTKLRDTAITTKVKAALLATNDLSSGDIHVKTRRGAVQLA

GTVPDERQRTLAVDVTRQVDGVKTVRDKLTVQPK

>WP_004552236.1 Flp family type IVb pilin [Burkholderia pseudomallei]

MRTQSFSRKSPRAHGIGARPARAFMRWLRDESAVSAIEYALIASLIAIVIIGAVQVVGTNLQSVFSTVAS

DV

>WP_004551759.1 MULTISPECIES: acyl carrier protein [pseudomallei group]

MSQPVSEPTRVLDPASAHSASIELWLIERIACYLQRAPHEIRPDTPLDEIGLDSVYALTLCGEIEEVLHL

TVEPTIAWDYPTVAEIARELDARLAQRSAQGEAHAQ

>WP_004551542.1 hypothetical protein [Burkholderia pseudomallei]

MTETIVVRMAHSDDYPNILALQKANQIGNLTEAERKNGFLSAEIPEERIGALASDTGIVVAYDQAEFAGF

FCISRLASWRGNAIIDALVASFEQSEEYASHRISETMCLFGPMCLNQVARGKGILEKMIGLAIESAKTQF

ENAISFIAVDNARSVNAVAKLGYRPVRRFTSGEREYHALIAEFA

>WP_004551270.1 MULTISPECIES: hypothetical protein [Burkholderia]

MENAFNERGVMVTRNGLSAAGQIFALREIRGVEVRTVRKNKLVPCAISLTGAAAAIAGGALGSSALLVAG

VMLVVVGYLAWTTQDVTHRLIVDMPDGKREAIMSVDREFVERVAHAVDAARAAGAST

>WP_004551182.1 hypothetical protein [Burkholderia pseudomallei]

MTNRSESPQPSDPRDAMLRDALRAQSFEEIKARTAEAVRKRPSDTRERWLLFQLLCIDGAWERALKQLQT

WAGLEPQGEARAQLHRGLIRCEMFRADVFAGKRTPGFIDAQPAWVDTLLQANAKLGAGDVAAADTLREAA

FHCAPVTRGESAEMGSFAWLTDSDTRLGPIFEIAVAGGYRWIPFEQLKSITFTPAGTLTDLVWRPVTALM

LDATVLRGYAPTRYSGSENGATAIRLASETTWRDIGTTNVVALGQRTWTTDQGDWGMLQIGGCRFTGESD

DAAS

>WP_004551068.1 methyl-accepting chemotaxis protein [Burkholderia pseudomallei]

MSPFMLSSIRSRILVACLAIVIGSLVINTALNYFVANRYNRESISQNLSAVLTGHEAGIADWVASKTQMI

VSVEDAAISPDPIPALKQIAAAGGFTNVYVGYADKTAKFSDPTGIPPDYDPTGRPWYKQAAQAGKPVVTP

PYVDVGTGKLVVAFAAPIMRDGALKGVVSGDVAMDSVIANVKAIHPTPGSFGMLVDRSGHIVAHADSKLT

LKPVTDLSDDLSLDALAAASADENAAPIDAHVAGAAKLMRARAVPGTDWLTVVALDKSDATAGMHSLLLV

SIGTLVALAAVAALIVGAITGVAFRGLARIRDAMESIGSGTGDLTQRLPDSGRDEVAQIARSFNAFVSKL

QEVMRVIRDASESVRHAAGEIASGNHDLSRRTESAAASLQQTAASIEEITSTVTQSAGAARQANDIATNA

ASVASRGGTVVSDVVSTMHEIEGASGKIADIIGVIDGIAFQTNILALNAAVEAARAGEEGRGFAVVAGEV

RSLAQRSAQAAKEIKALIDSSVTSVSTGATLVQQAGQTMSDIVGTVSNVTTIMREISNAADEQTRGIQEV

NRAVAQLDEMVQQNAALVEQSAAAASALQTQAVELADAVGQFKVA

>WP_004550756.1 MULTISPECIES: hypothetical protein [pseudomallei group]

MTASAAKPPRSPGSRRFAREAGSARPRDGTTLLHPVFQAQPCGRRTMEILPGRKRIGPATGGRRADQREV

ACAGWPLLRRNVELCRDPALP

>WP_004550683.1 MULTISPECIES: NAD(P)H-binding protein [Burkholderia]

MTQGTLKIALFGATGMIGSRIAAEAARRGHQVTALSRNPAASGANVQAKAADLFDPASIAAALAGQDVVA

SAYGPKQEEASKVVAVAKALVDGARKAGVKRVVVVGGAGTLEVAPGKQLVDTEGFPDAYKAVALAHRDAY

GYLSTVQDLDWTFFSPAALIAPGERTGRFRTGAGRLIVDEQGNSKISAEDYAIAFVDEIEQGRFIRQAAT

AAY

>WP_004550550.1 ShlB/FhaC/HecB family hemolysin secretion/activation protein [Burkholderia pseudomallei]

MKSRHDAWMLLLALVAAAGAAHAQSRSGGNPLEALPQINTPQKPSVTVQVAPQEVQVQALLARHLTPSSF

QVEGVKSIPFEEISQRFTPLVGKDITIGQLIETANGVTKLYQERGYALSFAFVPAQTFEGGVVRVTVVEG

YVANLKITGRPGAMEPKVRAIAAHIMADRPLRRATFERYVNTFGLLPGVTVKANVPPPQNTDGATTLELN

VDRKPFNVSAGLNTNNPGLQGLFTVTENGLTSLGEQMSISALFPKGPNNQTYVSFNGAVPIGSNGLVTRL

DASHYRGNPSVDQTVLPNVQRTVINDKLGLSASYPLMLSNQRSLLGTVSGYASHSEDRYQNQSTGATIGM

RSQVRVLQMQFDYTSVQPKQVQKLSFNVAKAFDILGASKSGFTNLPGVIATNPASTTFVRTGATFVQTNE

WPFKIGSTVQLTGQYSPDSLPSTEQISFGAQRFALGYQPGETSGDSGWGASLELNRAFAPGFTYLKNITP

YIVYDMARVYLHSGTPVPRRLSSAGFGVRLTDSRFYNLDVSIAKPVGDAPIESASRSPRVNASFSYQLY

>WP_004546703.1 MULTISPECIES: hypothetical protein [Burkholderia]

MGGSISKSSDAVSTASIPATQSHADTDTASKASSHSAQRRPSSGAFEGLMPLAQKGTVGELQNFAAAAFA

ATTGASPALVDAAHHNPAMQTLREQPMTQENVTQLCQMETDAARKGDPANWQNRLAASSVADVARHAVAQ

TVALNVSLGRDGPIKTQEEGGWNVHEHVIGVHGS

>WP_004545318.1 MULTISPECIES: transcriptional regulator [pseudomallei group]

MVVFLAAVELHNLAMPTHEEKAAFTERLKFALLRSPEKVTGATELALHFNLRHHGEHPVSPQTAHKWLTG

RTIPTPDKLHTLAEWLRVDLHWLHYGPPPSAARTTPQPLPRDEKYPPTPETIELASKIEALSPHHRYLVQ

ELIEQFYGDTSKR

>WP_004544493.1 MULTISPECIES: gamma-glutamylcyclotransferase [pseudomallei group]

MARLKQGRKMRYVFCYGTLRAGEINDIGRAAATHGIAAPRLVGAVAVAGRLYDFGNYPGMVAGGGRDLVW

GDVYAIDERLVPVLDEIEEVYPGVEGLFVRQRASVELGGRRYDCLYYPVGAHAIADKPRIESGDWVQYRR

ARTA

>WP_004543833.1 MULTISPECIES: FHA domain-containing protein [pseudomallei group]

MALLENGLSGEASLLRARHVLGRDPQRCDTVIADPYVSRIHASICWTAGRWELHDHGRNGTFVSGRFVGE

GECVVLRDGDLIQFGSAGSVRWRARELGEPVDMLWPLRAPARPIALDRTHALPGAAFTVSRSAQGDWLCN

DTTPARVLHDGDAVICGEFAWQLVLAHRGVTAALPRATQVATLPQRIDFTVSRDEEHVTATLHTRGGAVD

LGARAHHYCLVTLARARFADAQAGYDTASRGWIELDVLARMLGLDESHINVQIHRARTQFLPLLSPGSAE

LVERRRGGVRFGALAFRVVRGDRLECQSADADASAPLPDPLARGAATFVSPAALG

>WP_004541157.1 alpha/beta hydrolase [Burkholderia pseudomallei]

MTRRSRSYNNETTIRENKTMSFEEFAPFRVTAQDVDIFGVKGGAGPPILLLHGHPQTHMIWHRVAATLAR

HFTVIATDLRGYGASGKPPSDARHAPYSKRAMAADQVAVMRHFGFPRFYVCAHDRGARVAHRMALDHPDA

VERMLLLDIAPTLAMYERTDRAFATAYFHWFFLIQPEPLPETLIGGQSDAYVEAVMGNRSAGLAPFSPEA

LHAYREALRQPGAVHAMCEDYRASATIDLDHDRADLERGHKLACPLRVLWGEQGVVARCFEPLDEWRRVA

RDVSGRALACGHYIPEEAPVALIDEIVSFFEAREAA

>WP_004539597.1 MULTISPECIES: hypothetical protein [pseudomallei group]

MSNENETARLRASPPGYASMTRHHTHAGIPRTLKIVGYCGLAAIALALFVAMCAILKDSALDREAARQLQ

NGNVELHGGADVEPGSTNDAAQARTPG

>WP_004538328.1 MULTISPECIES: nodulation factor ABC transporter ATP-binding protein NodI [Burkholderia]

MSVAPIDFQQVEKRYDDKLVVDGLSFHVQPGECFGLLGPNGAGKTTTLKMLLGITHPDAGSISLCGEPVP

SRARHARQRVGVVPQFDNLDPDFTVRENLLVFARYFGLTAHAARALVPPLLEFAKLESKADAKVGELSGG

MKRRLTLARALVNDPDVLVLDEPTTGLDPQARHLMWERLRSLLARGKTILLTTHFMEEAERLCHRLCVIE

EGRKIAEGAPRMLIEAEIGCDVIEIYGPDPVQLRDELAPFAERTEISGETLFCYVDNPEPIHARLKGRTG

LRYLHRPANLEDVFLRLTGREMLD

>WP_004536963.1 MULTISPECIES: alpha/beta hydrolase [Burkholderia]

MSTQHLKDAPHAAARGRRDGLDMTWLDPLRLGPRAARIARGWAARSRPDIRFCDVPAATIRARVAGTRAS

GRPTIVLVCDPPSVVEHFDPLISLVAPHARIVCFEPPGFGFSVPNRHFTFSFDDYRAAIEAMLADLNEGP

YLLAFSCVWAHIALQIAAAQPALVGRLLLWQSPTWDEQVAWARHVDARNLLATPFVGQCACAFAARKIGA

GWFRQAMAKDRYREFTPMLDTSFDHGAFCCLASLWQRFYSGTPRAVQVTQPTLLTWGAADRTHRHSDKWS

IARQVPHAIRHPGFEHAGHSPELEESSAFSQLLLSWVDAKDPR

>WP_004536208.1 MULTISPECIES: hypothetical protein [pseudomallei group]

MPPTTLLSTNDAAQGSAETNMSSSDLIQPRQVLVSIASATATYTALSPHVPGFLHVVAQGEAPLHVGNIH

LAHDPGFVGGVLLEVVGIAGRPSPTVKQYRTPAYSFESCYHREIFIRGANGIIPVKVGLFRREAAQAAEA

ALDAD

>WP_004534826.1 MULTISPECIES: immunity 52 family protein [Burkholderia]

MEINTMFRDTTLSSRDYEEMLARESRLVDLLSTKSPTMARANWRVTGDTLEEANSYPAFEADGAPSRPAL

AVLTERGRGNKHGVSHAAIWNAATSAEEGASISCHVSDAKVLPDTLSVSLRVPGCYATADDFADVIKAIV

AAFHPAVIEASPDGYFEKQVFDDKPGVGWMLYLPKIITQQQVPEAHALIPVPEAGKRQTGTIIVSVTDAP

FSVDNPEHVAIANRIEIRLVDQDLLPAYVDI

>WP_004534573.1 MULTISPECIES: ATP-grasp domain-containing protein [Burkholderia]

MAATFIVKTFVFIESNTTGTGRLCLQKALLRGFDVLFVTSRPQLYPFLQEEMVVPLVADTADPQRIADAL

APYAGIAGIFSTSEYYIETAATVATRLGLPAADPEAIRTCRDKGRLHRRLRDAGVGVADTEIVSERTQLR

DLAHGATYPRVLKPAFGSGSVGVRLVRTPAEMLAHGERMLDARGNERGIALARQVLVQSFVDGPEFSVEV

VGLGAEHGHAVLGVTGKHLGPLPHFVEAGHDFPAPIAAAQRDAIVAETLRALDAVGHRFGPAHVECRVSG

GKVVVIEINPRLAGGMIPQAIEWATGVDVLGAMIDLHAGTPPDLGPRRRGHAAIRFVLPARSGELRALSF

EPDERFAGVRTRFMPLKQLGQRIEPAGDFRDRLALVIASAADPDALAHALEDVDRCVTVAIGDAGAAGEG

AGAGRLRRTLHPEALAIVRKPAPRAERLAELDAFAAIDEAHLLMLVDAGICDRTRAATVLAELARQRDAK

FAAIADAIAPRGTYALYEQLLIERVGIDAGGAVHTARSRNDINACVAKLRAREWFDTCGGKLWRVRAAIV

DKAQHTLDWPLPTYSQYQAAQPGSFGYYLWSVETALRRDQAALERLDEELAVCPLGAGAGAGTDFPIRPG

VSAALLGFARSFDSALDAVASRDLVLHFLAAIAIASTTLSRLAHDLQLWTMRETDFLALPDELSGGSSLM

PQKKNPYLLEIVKGKLAHVAGALNAAVFASQRTPFSNSVEIGTEMLAPCADAVQAFGESCDLLRLMVSGV

TGDPAKMRAAAEAGLVSATQVANALVRETDISFHAAHRQIGALITQALDAHEDPAAALDALVRQPGASID

EAAARLAYGGGPGAAGAGLARSRALLRQSAERLWRRRAAWHAAHARRRGCVADLLAAAAA

>WP_004534273.1 MULTISPECIES: porin [pseudomallei group]

MEEMMKKVMLAAVLATAGVAAHAQSSVTLYGRLDAGIEYMNGLPSANGGSTSRWRAESGNWGTSLWGLKG

SEDIGGGNKIVFQLEGSFDTMNGNGPGAGSIWNRWATIGISNDTYGTLLLGRELAIANGVWDFDPFGQSS

WSTASLVRGRNWNKTSNNISYQSPKFYGFDVYGQYALSNATNWNGNGSTSQGRSAGLQLTYTTSLFQIRG

LYDEARDPANGKLDDVFNYSREYFAGVNVFLGQFKLQAAYQASHADGGPVVNNGITTTQQVWGGVTWQAT

PAAALIAAVYHVNANHGGGNANIFTVGGSYNLSKRTLLDVQVATVRNSKTANFGLNANPAGTDISTGNPK

FGGSQTGVYAGIQHLF

>WP_004533804.1 fimbria/pilus periplasmic chaperone [Burkholderia pseudomallei]

MRLWKMCVAVAAACCLASTLSQAAIVITGTRVVYPESSREVNVRLNNVEQSPVLVQAWIDDGNAGASPDE

IKVPFVLMPPVFRVEPKKGQTLRVMYTGDDLPKDRESVYWLNVLEIPPKPTVEAEQNLLQLAFRTRIKLF

FRPSALEDGNAAQARDQLKWKIVRNEKGVSVLRAENRSPYYISISQATAKSGDKSVQFDPGMVPPFGSKE

FVSKHDISTLSTHAEIAYKLLNDYGAEVAGSATAE

>WP_004532148.1 PHP domain-containing protein [Burkholderia pseudomallei]

MPTMNADLHCHSNVSDGQLAPADVARRAHAGGVTLWALTDHDEVGGQRAAREAAEALGMRYLHGVEISVT

WASRTVHIVGLNIDPRNPALIDGLHRTRNGRAARAVAIGDALAALGIEAAYEGALAYVSNPDLISRTHFA

RFLVDKGYAASMSDVFDRYLGDGKPGYVAHRWAKLSDAVAWIRQAGGEAVVAHPGRYAYTSVEFDAFFGE

FIELGGVAIEVVTGSHTPDQYREYADVARRFGFEASRGSDFHAPGEGRTEFGSLPPLPSDLKPVWERWL

>WP_004532089.1 DNA repair protein RecO [Burkholderia pseudomallei]

MGTNDGTSDVGMTDEADADPQPFAAPPATGAPAADKPARKPRRAAPRTSEFRIAEQPAFVLHSYPYRETS

LVIDVLTRDHGRIALVAKGAKRPHSALRGVLQTFQPLSLSWSGKSELRTLTGAEWVGGMLPLAGDALLCG

FYANELLVKFCAREDPHPPLFQHYLVTLTRLAHGEPPVQVLRSFERVLLRETGYAMTLKRTVARRAVEPD

KLYVFDPQRGVRDAGSDAPSHWPVIAGQTLLDMEEDDYHRAQTVAQSKTLMRFLLNTYLGGTPLATRQIL

IDLQNL

>WP_004531206.1 MULTISPECIES: outer membrane protein assembly factor BamC [Burkholderia]

MTDLRLTKRFAALLATGAFVAGCSSPSPTKIDYKSDSKSKEVSLAVPPNMLDETPDQRSLPPQGGATSLS

SLQQVQQAAPPTDSVAPAVPGMHIQRDGTESWLVIDDKAPAQIWPQVRRFWQEQGFLLVVEQRDKGVMET

DWNETHPQINDGLIRGVISKAMGNSYVTAERNKYRTRLDAAPNGGTYVFISQKGMREALTGANNDSSKWE

PKPNDPGLETEYLKRLMAVIAQNDARVKAAGVPITDDAAPSRAKASSNKSKEAAAAIAAKNVASASTSST

PSGASVPDVPPEVTLAEPYDRSWLHVGLALDRANFTVDDRDRTKGLYYVRYVDPKDLSVEEQGFWSQLFH

GKKEKKAKQYLVNVKAVTEGETRIAVVDDKGNVDASAPARQIMSLLVNQLR

>WP_004531127.1 hypothetical protein [Burkholderia pseudomallei]

MLELSAEQVAGLAQIDERGFVERVRQDLVKENPAFADDDSLSSRLWTAYRAARALGIERDENVVAFLRLE

AYAPGFYEKPATKAWLTRPGRSADARFHDYLRVIKWRIEHPDGGLEHGGIGISGNRSGGGGAWADLGARW

RRLVGRRDGGGNGESVG

>WP_004531123.1 MULTISPECIES: hypothetical protein [Burkholderia]

MRKPQMLRMIVSPTTDTTMDLLLIATGLSVIGIGALVAFARRVDPLSGRLHGRLRKR

>WP_004531019.1 bacteriohopanetetrol glucosamine biosynthesis glycosyltransferase HpnI [Burkholderia pseudomallei]

MSVEGRAMKALAWLTGAVLALLAAELISFGDIGRFVTLLAALLAALCALAAAFGCVYTLVAAALTHRFFA

RAPREPHACPPVTIVKPLHGVERTLFANLASFCEQRYDGPIQFLFGVHDRDDPALRAVDALRTAFPRAHV

TIVADARLYGPNRKIANLVNMLPAAAHDVLIFADSDVSVGPDYVRHIVGELGEPGVGLVTCVYRGRPDPG

FWPRVEALVTSHQFLPGVVTGLALKLARPCFGQTIAMRRAMLDAIGGLAQFAHHLAEDHAIGEAVRARGA

RVVVPPFAVEHGCVETRVAQLVEHELRWSRTIRAVDPRGHLGSLLTHPLALALLAGVLSSGAAWAWPLVP

AALVARVAAKRIVDRATKRPVRDLWLLPLADLIAFGIFVASFSSSRVIWRGFSFDVDRDGRLCPAPEKRP

NA

>WP_004530923.1 MULTISPECIES: hypothetical protein [Burkholderia]

MSSDIVESRIVLVPVTRAYATYTSFSLHLLGTFHVYAEGEKPLNIRNPHLTPQMWYPPGGLAVVGVVGRP

RLGPITSYRTPVYSYQGQYQDTIYVQGANGIVTVKVRLLKDEEAESFLDTLDAAA

>WP_004530855.1 MULTISPECIES: 2-dehydropantoate 2-reductase [Burkholderia]

MATIEMTALRIAMLGAGAMGSLFGGLLAECGHDVTLVDVNAAHVDAIARDGLRLDTDRGERRVARLAALR

PDGVAAHAGMPPDLVIVFTKTLHTRAALAGARALFGPRTHALTLQNGLGNVETLAEYVPLERILVGVTTW

PADLAGPAHVRSHGAGWVRLMSADGAMRPIVQASAHALDRAGLNCAIDTGVWATIREKVGFNAALNTLCA

LTRGTVDALGAREDGPALALAIVAEVAAVARAKGVGADERRMRENVLHAIREHRGHRPSMLQDVLAGRRT

EIDAINGAVVAAAGELGVAVPHVRTLLQLVRLIDAQGG

>WP_004530793.1 hypothetical protein [Burkholderia pseudomallei]

MFQTSIISVVGVLVAIVLIFVVVTTTRTLSTVMSSVQAQTVAKNGYEAMADVLSIGQTGVTLNNVPMMRI

ELRVHHNGASWDVTIKQFIDLGNIPRAGERVRVMVDPADNGHVAYVGLAGAGR

>WP_004530453.1 MULTISPECIES: hypothetical protein [Burkholderia]

MIRAHPFGYPKHLDRRRVAQAGCPKRRKAVRRLHPPLAQPATALCRVPGYGALVQLWRRVRATLPGVVCF

EAMRPAFYRCVAAHAPARRRRSTRTAASRR

>WP_004530350.1 MULTISPECIES: O-succinylhomoserine sulfhydrylase [Burkholderia]

MSGLFVGLRIRRATRTAAMRLKTDERNMDDSLNFDTLAVRSGTLRSEFNEHSEALFLTSSFCYASAAEAA

ERFKHSEDYYTYSRFTNPTVTMFQDRLAALEGGEACIATASGMAAITSVVMAALQAGDHLVSSRSLFGST

LGLFGQIFAKFGIETTFVDPADLDAWRAAVRPETKMFFLETPSNPMTELADIEAVGRIAKAAHALFVVDN

CFCSPVLQQPLKLGADVVMHSATKFLDGQGRVLGGALVGSKAFIMEKVFPFVRTAGPTLSAFNAWVLLKG

METLSLRVNRQSENALEIARWLEAHPAVKRVFYPGLESHPQHALAKRQQKSGGSVVSFELNGDTPEQQRA

NAWRVIDGTKIVSITANLGDTRTTITHPATTTHSRIAPEAREAAGITEGLIRLAVGLEDPADIRDDLARG

LAG

>WP_004530218.1 MULTISPECIES: S8 family peptidase [Burkholderia]

MSMSILIRTASFKATVLCAALAGLVSAAQAETAAAPQVPGPADAVNQLIVKLRAVKTPPGATAAKAERAD

VQAVIDRVLAARNARAAGRAFGAAAASAPGNPDDPAAGIRIKRDMSGGATVLSLQRHVSLAQAEALARDF

AADGAIEYAEPDARMHPFVVPNDTRYSEQWGYFNPTAGANLPKAWDRTTGSARVVVAVIDTGYRPHADLA

ANLLPGYDFISDIPSANDGNGRDSDASDPGDWVSAQEDGDPSGPFYGCGASDSSWHGTHVAGTIGAVTNN

GVGVAGISWVGKVLPVRVLGKCGGMLSDIADGMRWAAGLPVPGAPSNPNPAKVLNLSLGGYGRTCSSTYQ

NAINEITSRGANVVVAAGNNGGSVSTTQPANCRGVIAVGAIDSRGVRASFSNTGAAVKISAPGVGILSTL

NAGKTSPGADSYASYSGTSMATPHVAGTVALMLAVNSTLSPSQILQRLQSSARPFSSGSSCSTSTCGAGL

LDAGNAVDAAAQ

>WP_004530215.1 MULTISPECIES: DUF4148 domain-containing protein [Burkholderia]

MKATFLTVALAAAFVSPAAFADAPAGKTRAQVYQELVEAKANGLDYVTDASYPEISPLYAPRFANKAKPQ

QPAHAVADTRSDAKAGSSGNLSEAAQDEHCVGPRTFCNPYAGS

>WP_004530193.1 FGGY-family carbohydrate kinase [Burkholderia pseudomallei]

MTEPCVLAIDLGTSGPKAAIISLDGRVVAAARDAVATLRMPDGGVEQDPLAVWRAVKRACGGALRAAGIA

SRDVLAVACASQYSSIVPVGADGAPVANMMLWLDRRGAPRARRGAADGPLRADSPLRQWRWLRVHGLPPV

EGGISLTHMRYLKHAKPDVYARTATFLEPMDYLNLCFTGRACANQCTAFMSLVVDNRRLNVACYDPRLVR

YSRIDASKLPELLPVDAIVGRVLPDVADELGLPRGTPVVAGLNDTQAGGIGAHAFAGEHAALSIGSSSVM

IAHVRFKRTDIRHAILSMPSPIPDTYFVMAENGIGGAALKHFIEQQVYADDPFGALPRDDCFARLQKAID

ATPPGSGGVMFMPWLAGSLAPHADASMRGGFVNLGLDATRSHLARAVLEGVAMNLRWLRGPVEAFAKRRF

SHFVFYGGGAESDAWSQIVADVLDAPVHRIEQPQYTTCVGVALLAFQRLGLLGFDDFASRVRIRGVCEPN

RANARVYAEMSAQFVEAFRRNRPIFRALQRNLRRPASAAANGLRPSG

>WP_004530105.1 MULTISPECIES: sulfite exporter TauE/SafE family protein [pseudomallei group]

MTAYAIVLLIGVVAGVVSGVIGTGASIMLLPPLVFYFGAKQAVPIMAIAAVLGNVSRAYVWRRDIDWTAF

FAYSIAAAPAAALGANTLWALNPQWVDCALGVFFLSMIPYRYIARRSTFALTAWQLAAAGAVVGYLTGIV

FSTGPLTLPIFSAYGLLKGGLLATEAAASLAVYASKLVAFGQLGGLPLDVAIKGALVGLSLSAGISVGKA

VTLRLSTNAFQRLLDLVMLSAGTTLLWGAIR

>WP_004530058.1 MULTISPECIES: choline ABC transporter substrate-binding protein [Burkholderia]

MTPGVTHFQMEQAMKRYESIARRLARRAAAASPAFAALAWCAAAAAATTTAAAAEPAACRDVRMAGPGWT

DIEATNALAGVVLKALGYRQSVSNLSVPITYQGLKKGQLDVFLGNWMPAQAPLVKPFVDARAIDVLHANL

SHAKFTLAVPDYVAAAGVHSFADLAKYAQRFGAKIYGIEPGAPANQNISRMLADKALGPANWQLVESSET

GMLTQVERAVRERQWIVFLGWEPHLMNTKFHLVYLSGGDAYFGPDYGGATVNTVARADFASQCANLARLF

RQMTFTVDLENGMIAAMLQGKRSAVDAAQHALRANPSLVEAWLDGVRTASGAPGLPAVRAALDAQ

>WP_004530043.1 DotU family type IV/VI secretion system protein [Burkholderia pseudomallei]

MSLLRNLSSTLRRVSMASNMLDRAAANPDLVTRVPTLSSLSSAAMTSSAVSTTGTTNTVASGGAAAGAPS

FAPPAADAFPQADGAPRNPAVLQFPVPGGAPAPADARVAAPVVYSAQGEQAAIMKAGLQQASWNNPFVSH

ALPAVLQLQRHLAAGPLNQAAIRTQLGLEVRLYRERLAASGCEWEQIRDASYLLCTYLDETVNDAAREHA

QVVYDGERSLLVEFHDDAWGGEDAFADLSRWMKTEPPPIPLLSFYELILSLGWQGRYRVLDRGDVLLQDL

RSQLHALIWHHVPPEPLGTELVAPAKRRRSWWTAGRAAAVALGVLVLAYGAISFWLDSQGRPIRNALAAW

MPPTRTINIAETLPPPLPQILTEGWLTAYKHPQGWLLVFKSDGAFDVGKANVRADFMHNIERLGLAFAPW

PGDLEVIGHTDSRPIRTSEFPDNQALSEARARNVADELRKTALPGGARAPENAVQRNIEYSGRGDAQPID

TAKTAAAYERNRRVDVLWKVIPDGAQQSGRSLNLQQPEKPGQVPMRPAMPEGVEIAPDGQLPYATSTTMP

ATRPTTEGRQP

>WP_004530038.1 MULTISPECIES: type VI secretion system contractile sheath large subunit [pseudomallei group]

MSMQQLESSAEKVVVDQNNVNEDLKDILRRSFRPRTNEAAEAVQNAVETLLTYARRSRVVVREDVAQTIE

QLVAELDKKISEQLTLVLHNKRFQSLEGAWRGLHYLVSNTDTSENLKIRYLNISKADLGKTLRRFKGVVW

DQSPIFKMIYEQEYGQFGGEPFGCLIGDFYFDHSMQDVSILTEMSKISAAAHAPFIAAAAPGLLQMDDWS

ELSNPRDVSKIFTATEYAFWRRLRESNDSRYLALTLPRFLARVPYGPKTQPVEEFGFEEKVDPNRAEDFC

WANSAYAMGANITRAFKTYGWCTKIRGVESGGAVEVLPKFVLPSQDREVDLHCPTEIAISDRREHELSES

GLMPLVYRKNSDTAAFIGAKTVHRPAIYEDDDATANSNLSSRLPYIFATCRFAHYLKCIVRDKIGSFKSA

EDTQRWLNDWLMNYVDGDPSISSEVTKSQRPLSAAEVVVDEIPENPGYYRAQFFLRPHFQLEGLTVSLRL

VSKLPSTKHEVTT

>WP_004529973.1 MULTISPECIES: copper oxidase [Burkholderia]

MKMVSRRTFLGGSGAALLGAALVSKAGAASLPEAPTMAKAATQPPLAPPNGRPYTPVVTLNGWTLPWRMK

NGWKEFHLIAEPVVRELAPGMNAHLWGYNGQTPGPTIEAVEGEKVRVFVTNRLPEHTTVHWHGMLLPCGM

DGVGGLTQPHIPPGKTFVYEFQLEKAGTFMYHPHADEMVQMAMGMMGTFIVHPKDRGAMPADRDFVFLMS

AYDIDPGSFTPRVNEMTDFNMWTWNARVFPGIDPLPVRAGDRVRIRFGNLTMTNHPIHLHGYAFEVAGTD

GGWIAPAARWPEVTVDVAVGQMRAIEFTANRPGDWAFHCHKSHHTMNAMGHQVPNLIGVPQQDLAKRINK

LVPDYMAMGSTGGAMGTMEMPLPENTLPMMTGTGPFGPLEMGGMFTVVKVRQGLGRNDYRDPGWFRHPKG

TVAYEYAGALPDD

>WP_004529705.1 MULTISPECIES: SDR family oxidoreductase [pseudomallei group]

MTTSPSPLPAVRAIVTGHTRGLGEALAEQLLRRDIAVLGLSRGRHPSLASHAPERLVEAELDLSEPARVQ

AWLGGDTLGAFVAGASRVLLFNNAGTVEPIGPLDTQDTAAIARAVGLNVATPLMLASALAKLAPDALERR

IAHISSGAARNAYAGWSVYCATKAALDHHARAVALDANRALRICSVAPGVVDTGMQATIRATSDERLPSR

ERFAQLKSSGALSTPDDAARQLIDYVLSDDFGSTPTADIRHLG

>WP_004529682.1 MULTISPECIES: LysR family transcriptional regulator [Burkholderia]

MRATRGASRRNGSSQDSGAQMSEQSKNSQAGHAPLAYPLAALTRALPTLAALQSFVAAAQLGSLSKAAAH

LCRTQGAVSRQIQQLEAHYRCALFVRQPTGLTLTADGDALFAVAADVLARLVRHARERDEAAAAVTVRVP

STFAIRWLLPRLPAIRRALGATQLCLSTSANDTPDFSEPDIDAIVARGDGRWPGVEAIPLFAETLAPMCG

PALAASLKSVDDLAHVTLLHPGRGRAEWRCWLDAVGAAHIDATRGPVFDTLELTLSAAAEGHGVAIGDPR

MAGDRLRAGTLTMPLRETAANGLSYYLVYPAQRATQPKIRALADVLVKLARAR

>WP_004529400.1 MULTISPECIES: GtrA family protein [Burkholderia]

MSAVADIGMRARIVRFGVSGAACTALHAAIAGVLMGAFAATAVQANAIAFVCATGASYLLNTLWSFSAPL

RWRNVARFVAVSVVGLMLTMAISHGVLALGLAAAWSIAAVVALVPPLTFAMHRLWTYR

>WP_004529359.1 MULTISPECIES: hypothetical protein [Burkholderia]

MRTTGSSGAMTLLTEFDQADGRELRSLRLVATEDGKSVLLIEVDERKPGIHREVRYEITPAELIAAIRSH

GAELPGETHGTVPLARTTAP

>WP_004529210.1 beta-glucosidase [Burkholderia pseudomallei]

MKRLAAVAMSVTQSISIPIHYPAATAALLLLLLTGCGGGGDQSKVNAAASPANNLVVPAPGTASPGTPAP

APGAPAPAETASVLPFFGVNGHYVDGGVYASVPLATQASHLAGLGMNVYRQDVYIPDHVDTLASTVIPGL

GSGITVLPMIQAHPWADPSLNGQPPTEASAYAYAYKLAAYAAKKLAGIPMVEFGNEYDIDSHNAPIQGDG

INVSDYDNSTFPIWRGALRGSLDGWRSVDTNRTTKLIANATSGALHFGFLDGLMTGTQPDGTTGHPKITP

DVIQWHWYSNGGDFENALGKTGRYNVLARLKDRYNLPIVVTEIGVNTDNSDTQIAAYIAKTIPELVAAKA

AYNVIGFNWYELYDDRSGAYGLLTNSAQEKPRYGLMRAAIAGAVPN

>WP_004529200.1 GGDEF domain-containing protein [Burkholderia pseudomallei]

MRKRTTSFATRFLQPWTQSLMSPTGVIVCGMLLLTLIWLFCARVLYQSHRDAYLHAVDNAHNLVLLLERD

IARNVELYDLSLQAVVDGVNDPRIMALDPAIRAKVLFDRAATGKYLGTISVMNEHGDIVLDSHFPHPPAV

ANFSYRDYFVYQRDHPKGGLYIGEPYAARLRHGAPTIALSRRITRADGSFGGVVVGTLGIDYFRSLLDGL

SVGPDGTAAVFETNGLMITRLPFDPKMVGRSIADSALYAHVREHDAGVFTGVASIDGVRRLYVYKRLQGL

PIVVNVSPAERHVFMQWRIRAQRLGVLMLVFGIAIVGGTALLARELRWRRHAEMRLQRLARTDALTGLGN

RRAFDENLRSEWARALRTGRPLSLLFVDIDQFKDYNDHYGHQAGDDVLREVGGCLAVNVRRAADDVARYG

GEEFVITLPDTDAKSAAAIAEYIRRAVYDLDIEHVRSPYARVTVSIGLVTSHEHIAHSDATLVKMADAAL

YQAKSTGRNRVCGAQHA

>WP_004529001.1 hypothetical protein [Burkholderia pseudomallei]

MRSSDAYRLGVDIGIDVRIDARNGANPFDVVARRRHARPDIQRNPFSHLPMAIRQSPVRACSRRATATRT

RFPAWPTPSPGCMRRDAVTGESRGMPRAHNATR

>WP_004528594.1 hypothetical protein [Burkholderia pseudomallei]

MTSLPSVKATPMQAISEQQARAIRGGGSAYANSAAYTVSLANQSITPVVNGLLNEYQTQAVNQFKMALGS

GS

>WP_004528106.1 MULTISPECIES: hypothetical protein [Burkholderia]

MGMLDDVQLKAVHASLAPVVERLSEASATLASSAVPLLGVPPATAPPAAVPVAAAPPAGVELATQLSAHA

APVALAAPLVSLSIPALSQPVRT

>WP_004528008.1 MULTISPECIES: multidrug transporter [Burkholderia]

MQEGNMSDPSRENPPTDAHASSLVTHDEPVPRGRRAPRPLWTLVPRAAGYGVVFFVIWMVLTIMFPNVFT

RSSERAVVNNEVTLVTSPVEGVVTEQHVTAGKPFDANQPLATVQNPNVDRALLIDLTGKKLDNQQREDAA

RAELAGDESQLASTEHDLQRYQSVAQKEHAATIRALEARLAVARAQVDQQEDIVNRNQAMQWAGAVSEAY

TSASRYQLSILSNAKAAAAAELEHAVANGDASRSKVYASATDGPAASLSQRGRLLGADIAQRKAEIAQFD

AYGQSVDKLIAAEQQRLDRLSRIEIRSGEPGVVEDVLAPPGTRVAAGATLIRASNCARSRVVAVFPRSLS

DDLLPGTHLNVRMDGVPAVLPASIAEVLPRASEGEQARYFVPFPPIEKNEIYVIAKLDEPLAPLSRRASA

RPDARCAMGRWARVSLDRGWLASNVSGLANVDPNWTAGARSALARGGQWLRDAGVQARRRLEDFATNAAR

RLGELAADGRRWLNERASAARRWLDDLKSAA

>WP_004527921.1 MULTISPECIES: hypothetical protein [Burkholderia]

MKIVTYLGFTVAATIISMSNLGAAFAYDDLPKPPPPPEGWVQPPKPSEGTVTRDVENQIAQRFTAATGGN

PNGLLSKQQAKAAGWGLVSDRFSDIDRAGTGYVRLDDVLRFMSERTPQRVMRMRNATKPNP

>WP_004527907.1 MULTISPECIES: cytochrome c biogenesis protein ResB [Burkholderia]

MSVTTSGLQSRTGPSAVRRAVELLSSMRFAIALLVVLSIASIIGTVLTQDDPYPNYVNQFGPFWADIFRS

LSLYTVYSAWWFMLILIFLVVSISLCVIRNAPKMLADAKSWKDKVREGSLRAFHHKAEFAVAGDRARATQ

TVAAFVAKAGYRHVVREAGGATLVAAKRGAMTKFGYIAAHLAIVVICVGGLLDSNLPIKFQMWMFGKSPV

NTSATISEIGAEHRLSASNPTFRGYAWVPEGQYVSTAILNQPTGSLIQDLPFSIQLEKFIVDYYSTGMPK

LFASDIVVVDRETGKRIPARVEVNKPFTYKGVSIYQSSFQDGGSLMEMTAYPMTGASAATFPFKGTIGNS

VPLAAAGADGETVEFSDFRAINVENVTDANGKTDARGVAANRSIKELFDERLGSGAKTSKPVELHNIGPS

VQYKVRGKDGQAREFNNYMLPVDMGGERVFLAGVRASPNDPFRYLRIPADSRDSVGEWMRLRAALEDPAV

RIEAARRFARHSLGTGDAALRDRLEDSAQRVLTLFAGADGSIGRGADGNPIGGFQSVATFIDRSVPKDEK

EKAAALLLRMLEGSMWEVWQIARERAGEPPVQQAQDTVRFVQNAINALSDSFLYGAPVYLQLDSFKQVQA

SVFQLTRAPGKNLVYLGSLLLVAGIFAMFYVRERRLWFWLKDTGAGVSVLMAMSTARKTFDFEKEFVQTR

DAAGAALGAPAFRAPDGAGTKDSTR

>WP_004527836.1 MULTISPECIES: type VI secretion system baseplate subunit TssF [Burkholderia]

MEELLPYYERELSFLRRYSLEFANRYPKIAARLAQTGEQCEDPHVERMIESFALLGARINKKLDDEYPEF

TEALVEVLYPHYLRPFPSCSIAQFGAPHAFAELTEPRIVERGTELKSRAIRGVQCRFRTAYDVTLAPIRL

ADARYGSVAVAPAAARLPGNATGVISIAFESTSPQLDLAALKLGKLRAHLHGEQSFVAALADCLFAHALA

CYVEPDRGGRWIALGKTPFEHAGFDETDALIDYPARSHPAYRLLTEYFAFPDKFNFVDFDLGALARRAGP

CRQLTLHIALRDVRGDSHVARLLDLLSASHFRLFCTPVVNLFRQHGEPIRVDHRAVSYPVIGDARRAFAY

EVYSIDSVHLVRQRADRETLVEFRPFYSLHHGEAAQAGHYWFARRNDDVARKSPGYETEISVVDVDFEPG

APQTDTLSLDLTCTNRDLPAALATGLDGGDLFIDGGEQPGPIALLRRPTPSARFERGHAAHWRLVSHLAL

NHVSLAEEGLAALKEALVLYDLRRSAVSTRHIDGIVGIEQKSAVQWLPGKPFATFVRGVEIRLAIDDEHF

VGSSLATFVRMIDAFFGLYVHLNSFVQLIVVSRRTGEEIMRCKPRSGESILA

>WP_004527663.1 MULTISPECIES: LTA synthase family protein [Burkholderia]

MASSMPITFVFAIALSFAADAIAIPRAAVRRPFLAGALHTASVLFVACVILALTRRPHFAAFLALALVAL

AGAVSNAKFSSLREPFVFTDLSLFSQLFSHPRLYLPFLSATTVVAMVLGSVALAAGFFLDPAVSAQTAWQ

AAMAALICFATGGLCAARLPLTLDPLDDQRRHGFFAVFVAYLLNGMRPATFRSVAGLTCAGPFASEDELP

GHPDVIVIQSESFFDPRPLSAAIDSSILGHFDRVCRESVEHGQLAVPAWGANTMRSEFAFLTGLPSSHLG

YARFYPYAFVRRISASLAGWFRRAGYRTTAIHPYYADFFGRDRVFPLLQFDRFFDIRAFGDAPRAGPYIS

DAAVLDQIVAVLDEKRTQPAFLFAMTMENHGPLHLEPVEAGEAARYHTLGDDATWRDLTAYLRHVANADA

MIGRLVAYLRQCRRDTILCFYGDHVPALSHVFERFGNIPDQSNYFIWRNFGEHAPRKQDRAVEELGTALL

RAMKTTGRQAVSTGASEITT

>WP_004527639.1 MULTISPECIES: DUF3318 domain-containing protein [pseudomallei group]

MSYSAPEQRFHTASQRRHMSAAQYRALRKELLILRSDVERLELAQAGAELRQSITHFKWLKLLVPGVSGG

SLGKSARNVNATLGMLVSQYPLLSSLASVVLAKPVRALLRASARPALKWGAVGFALWEGYQIWKQAKHDD

ASDAGARQRTRP

>WP_004527446.1 MULTISPECIES: LLM class flavin-dependent oxidoreductase [Burkholderia]

MIPFSVLDLAPIPAGADAAQALRHSVDLARHAERLGYRRYWLAEHHNMPGIASAATAVVIGHVAGATRTI

RVGSGGVMLPNHAPLVIAEQFGTLASLYPGRIDLGLGRAPGTDQTTARALRRDLIGSADSFPDDVVELQR

YFAAPAAGQRVRAVPGAGLDVPIWLLGSSLFSAQLAAMLGLPFAFASHFAPDYLMRALDVYRAQFRPSAA

LDKPYAMVGVNVFAADTDDDARRLFTSLQQQFLKLRRGTPGQLPPPVESLDALGATEQELANVAHALSFA

AVGSRDTVHERLRRLIAQTGADELIVAAQIFDHGARVRSYEIAAQVRDALRDEAGV

>WP_004527256.1 MULTISPECIES: NAD(P)-dependent oxidoreductase [Burkholderia]

MMGGNFNRYSNERKGEHVDVGFCGPGLMGAPMIRHLLTAGHRVHVWNRTRAKADALAGCGAQVVDTPAEL

AARAQTVMLCVLDAQAVGDVVFGASGVLAGDAATRRVRRIVDHSSIAPAATRAYAARAAALGVSWIDAPV

SGGVPGAEAGTLAVMAGGPQADVDAVRVLIGAYAARVTHLGDAGAGQTAKLCNQAIVTATVTAIAEAVGL

AQASGIDAARLAEALAGGWADSVLLQTFVPRMTRAGHPPIGALKTFQKDVDAIADAARDAGAVMPVASTV

QQVLRLGAAMGLAQADFAAFIDIVRPQAKRGA

>WP_004527249.1 penicillin-binding protein 1A [Burkholderia pseudomallei]

MLEARLVRRPSDMPIIKRPPSSRATNEHHYSLRRSPAGAYYTDDDDDDERAPRRTGRDGGSRTFGSRVAL

WFAGLFATLLVVGALIVGYALVVMGPQLPSLDALTNYQPKVPLRVYSADHVLLGEFGEERRSLVRFADIP

DVMKKAVLAIEDYRFYEHGGVDFVGILRAGVADLLHGGARQGASTITMQVARNFFLSSEKTYTRKIYEML

LAYKIEKALTKDQILELYMNQIYLGQRAYGFAAAARVYFGKDLKDVTLAEAAMLAGLPKAPSAYNPVVNP

KRAKVRQEYILKRMLEIGYITQQQYDQAVKEEIHVRTPGNQYAVHGEYVAEMVRQMMYAQYKDETYTRGL

TVTTTINAADQEAAYQAVRRGIMDYERRHGYRGPEGFVALPPAGDERDEAIDDALADHPDNGDLQSAVVL

AVSPTAVDVQFVGGATATINGAGLRFAAGALSARASAALKIKPGSIVRVMKDARSAWQIVQLPQVEGALV

AVAPQDGAIRSLVGGFDFNKSKFNHVTQAWRQPGSSFKPFIYSASLEKGLGPATIINDAPLYFPPSVPGG

QPWEPKDDDQPDGPMPMRTALQRSKNLVSIRILASIGTSYAQDYVTQRFGFDPAKTPPYLPMALGAGLVT

PLQLAAGYAVFANGGFKVDPYLIAEVDDARGQALQKAQPVVAGASATRTIDARNAYVMNSLLHTVATAGT

GAGTNALGRGDLQGKTGTTNEAKDGWFAGYQQSLVAVAWMGFDQPKSLGSREFGAQLALPIWVNYMRTAL

NGVPEQQMAMPDGLTTIDGELYYADRTPGAGFVASVDFNPAASPTVSANDALGSAGAAGLTPPPVTPEEK

RQIIDMFEGNKP

>WP_004527232.1 MULTISPECIES: class II histone deacetylase [Burkholderia]

MTKTAFFTDERTFWHTGGAHALFFPVGGWVQPPSSAGYAESPDSKRRLLSLVHASGLAAKLDMSSAPAAT

DDDLRRIHPAHYLDAFKRASDAGGGDLGELAPFGRGSYEIAALSAGLALAAVDAVLAERTANAFSLSRPP

GHHCLRDKPMGFCLLANIPIAIEAARAKHRVERVAVIDWDVHHGNGTQSIYYDDPNTLTISLHQDRCFPP

GYSGADERGAGAGAGSNVNVPLLAGAGDDAYRYAFERIVLPALDAFRPELVIVASGLDANAVDPLARMQL

HSDSYRYMTHALKQAAQRHCGGRLVIVHEGGYSEAYVPFCGHAIVEALAGMRTDVADPMLELAIAQQPGE

RFNAFQRQLIDEMATSFGY

>WP_004527158.1 MULTISPECIES: phosphoribosyltransferase [Burkholderia]

MTQNLTASITMSDPRNDDKNLWVGWDEYHRLIEMLALAVHDSGWKFDKILCLARGGLRVGDQLSRIYDLP

LAILATSSYREAAGTQQGDLDIAQYITMTRGELTGNVLLVDDLVDSGVTLARVQQHLKERYPAVTAVRSA

VLWYKGCSKVKPDYHVQYLPTNPWIHQPFEEWDTVRPHNLAAWIKRGNAQRDDASAA

>WP_004527120.1 MULTISPECIES: depolymerase [Burkholderia]

MPMRHAAARAVLAAAFTITLAAAPAGARAAPPLPALRADANRVSVSGLSSGAYMALQYQVAYSASVVGVG

VIAGGPYYCAAGSVANTDLCRGLVPNMVPDSGRLVAAAQGFAASGQIDPLANLQRARIYLFSGTKDTLVR

QSAVDATWSFFWLVGVPVTNIVYVADVPAGHAFVTPSAGNACDANAAPFISHCTVGQSGYDQAGALLDAI

YGPLAPPAATPTGRAIAFDQREFAPASSGLAAQGYAYVPRTCDANAGCKVHVVFHGCLQSAAVVRDMTTY

DNWADANGIVVLYPQVAKTSTPNDPQGCWDWFAYTGQNYAWKSGAQMRAVRAMIERVTSAP

>WP_004527043.1 MULTISPECIES: adenylyl-sulfate kinase [Burkholderia]

MDKLHAERNVEWQTTSVSRRDRESRQAHGALAVWLTGLPGAGKSTLAIEAERCLHQAGKRTYVLDGDNLR

HGLCRDLGFSADDRAENIRRAGEVARLFVDAGVIVLSAFISPFRFDRAKVRALFDAGDFIEVYCDCDPRI

CEIRDVKGLYRRARAGLISEFTGISSPYEPPRHPDVYVNTGKESLDACVDRIVRRVLHSLR

>WP_004526460.1 MULTISPECIES: triose-phosphate isomerase [Burkholderia]

MNTTPGFGASTTRQTMSKQRIKRVIGNWKMHGRLSGNEALLDEVAQGAQAVPEHVGIGVCVPFPYLAQAR

ARLHGGRVAWGAQDVSAHEQGAFTGEVAAGMVAEFGAAYAIVGHSERRAYHGESNEIVAAKAQRALAAGL

TPVVCVGETLAEREAGATEQVVGVQLDAVLAVLSQDEAVRIVVAYEPVWAIGTGRSATAEQAQQVHAFLR

GRLAAKGAERVSLLYGGSVKADNAAELFGQPDIDGGLIGGASLKSGDFLAICRAAQ

>WP_004526292.1 GNAT family acetyltransferase [Burkholderia pseudomallei]

MSAGMSTAIVSDDIAIRPFARADTDAALAVWRDAFPSYSDASTPHRDPRRAIELKLATQPELFFVATAGG

RVVGTVMAGYDGHRGWLYSLAVERGARRLGIGRALLAHAEAALAERGCPKVNLQVLPGNDDACRFYEALG

YHEEARISFGKRLATD

>WP_004526233.1 MULTISPECIES: zinc ABC transporter substrate-binding protein [Burkholderia]

MSLAAAPRMFASCSSRSPSAPLALRASPLLRAARALAAGVAALALASAAFAQNATLKIVAAENFYGDVAK

QIGGARVAVSSVLSNPDQDPHLFEASPKVARELQHADLVIYNGADYDPWMAKLLAASKNAKRATVVVAEL

VGKKAGDNPHLWYDPATMPAAARALAAALGSADPAHKAEYDANLAKFVASMKPIDAKVAGLRARYKGVPV

TATEPVFGYMADAIGLDMRNPRFQLATMNNTEASPADIAAFENDLKRRQVRVLIYNSQAVEPMTKRMLKL

AQDARVPTVSVTETQPAGKTFQQWMLAQLDALGAALGKRP

>WP_004525997.1 MULTISPECIES: D-alanyl-D-alanine carboxypeptidase [Burkholderia]

MRLSSLGLTSLASSTAIAVAARNVALGIVLPAALVSTIAVAQAKPAAKAKHAAAPAAAAAPTGAPATYVP

GAVPPPGVNARSWVLVDASSNQVLASGNADERVEPASLTKLMTAYLVFEALDAKKITMEQIVTPSEAVRR

VGRDESRMFIEANKPVSVHDLVYGMIIQSGNDAAIALAELVGGSEAQFVNMMNAEAQRLGMKNTHFADVN

GMPDPNHYTTAGDLAKLSTHLIRDYPDYYNIFSVKEFTYNNIKQPNRNRLLWLDPTVDGLKTGHTQAAGY

CLIASAKRALPGAADATRRLVTVMMGETKESDRVQDSMKMLNYGYTAFDSVRLYKASQPIDTPRVYKGKS

NNVQVGVKKDQFITVPRGLADKVKPEVALNAPLIAPLADGQVVGSVKLVADGKTVAEFPVVALQPVPEAG

LLGRIWDSILLMFSKKK

>WP_004525001.1 MULTISPECIES: ABC transporter ATP-binding protein [Burkholderia]

MTMAGLAIDARGLTKWFGEGEARTRALADVSVQARFGEMLLIVGPSGSGKTTLLSVMSGILRPDAGSVIV

DGVDLWAQDNDAIAEFRLNRIGFVFQDYHLFARLTTAENVAIPLILKRRDWNRALADAHEYLDVVGLRNR

ASLPPVKLSGGEQQRVAIARAIVSQPDILILDEPTASLDGDTGRTIIDFVKHKVLNDKRCIVIVTHDSRI

FDYADRILRMEDGKLTAIERGGGL

>WP_004524947.1 MULTISPECIES: hypothetical protein [Burkholderia]

MNTPQESFPVAPNAVYVWRGFRSPTLDYEQFAQFLGSVFVPACVLLQPPVGLRAYLPTMMPQAGKPAAVP

DQTALMFWATPASHDRAMKALAVRIYQNLHGDVYDMTRSKLPEVPVALDPHADALVAEQPYFLIDRAADW

MLGSAKHFVGARRANLRAADFLAAVHVWATSFQIEPPNGVDGALVCCGEDYVAAWIHGTYPQPRPCPALD

QLAALTTPVLRMAPRPLALPAGLWDDWSGLDLTRDTCINLQFPRPRTSRAAPREPEA

>WP_004524924.1 MULTISPECIES: hypothetical protein [Burkholderia]

MKPTEFKITVSGAALDGRQIAHASAQLNIALLTALVGENATQIKGPAWNVVLPNGGRIIVAEEAERLLRV

TEQERIGE

>WP_004524638.1 MULTISPECIES: outer membrane protein assembly factor BamE [Burkholderia]

MPRAKPRRFPMTMFRFRILPAAMLGAALALAGCDDRQSEQTVQRFKDFFNAIKPAPLFLKGLTPGVTTEA

EIRGQMGRPETERVYTDGSKRLEYPRGPMGNETYMVDIDASGRFVAATQVLTAANFAKIRPGMTQDEVRR

LLGKPTEVARYPLKPETVWSWRWLEDGVNQDAFFNVHFGPDGLVYTTSRSDILKGR

>WP_004524196.1 MULTISPECIES: LysR family transcriptional regulator [Burkholderia]

MSNIRSHVSRVLMDRLDWNLLRTYLTIMQERSISRAAARLHLTQPAVSQALKRLEDSLGRTLIQRRGAQF

QPTRAGEEVYRIATDIYGHMSRLDNELDDRGGELIGSVRLLCVSRVESPVYDEFLAEFRRTYPRVDLHVE

VMRSADILSSLLQKTATCGLALCRTPVDKIELRSFLRQRYAMFCGRHHRLFGRQALKIEDLLAENFVSFT

SDQIGDALAPLTVFRDQRGFTGRIVATSPSLDEIRRLVFAGYGIGCLPEHIVRDDLARQRLWRLPPEEGL

LDVEIFLMWNRERRMNAAENVFIDAFQRYVQRYPMAERLGMLH

>WP_004524090.1 MULTISPECIES: catalase [Burkholderia]

MISRHSMRWLSRALVAVSASGALAAHASTLTRDNGAPVGDNQNSQTAGANGPVLLQDGHLIQKLQRFDRE

RIPERVVHARGTGAHGVFVATRDISDLTRAKVFEPGTQTPVFVRFSSVIHGGTSPETLRDPRGFATKFYT

AEGNWDLVGNNLPVFFIRDAMKFPDMVHSLKPAPDTNIQDPDRFFDFFSHQPEATHMITRVYSDAGTPAS

YREMDGNSVHAYKFVNAHGGVTYVKFHWKSLQGQKNLTAAQAEAIQGKDFNHMTRDLIAAIDAGRYPKWD

LYVQTLKPDQLDQFAFDPLDATKVWPGVPEVKIGTMTLNRNPGNVFQETEQAAFAPSNLVPGIEPSEDRL

LQGRLFAYADTQLHRVGVNGAQLPVNRPRAPVANYNRDGAMNGGAARGTVNYEPGAQAALAADPAFAASR

APLAGSTQQARIAKTRNFDQAGAFYRSLSASERANLVANLAGDLKQVRNDGVKYTMLSYFQKADAEYGRK

VTAALGADQGRVDALTAKLAD

>WP_004524036.1 esterase family protein [Burkholderia pseudomallei]

MRASNHYAVMRRFVSPLAALLAAALTAAAHAAPASSIVTRTFRSPALHRDWSYTVYLPAGYNPEGARYPV

LYLLHGNAGNANDWITQGRLQLTADALIERRDIAPVVIVMPQGGTDWYVDRKEKMQSAFLDDLIPDVEAH

YAVSNQRAGRAIGGVSMGGYGALRFAFLEPERFCGAMLLSPAIYANEPPASSAARYVGVFGDRQFDPKVW

HELNYPALWRGYFAQPLRLRMFIAAGDDDLSIQAESSALYTSLRRAQNPAALRIVDGAHTWDVWRRLIGP

ALKYTLECVK

>WP_004523548.1 MULTISPECIES: type VI secretion system baseplate subunit TssG [Burkholderia]

MARDMTQAAVADTALSPQALARLRAEPWRYGFLALLRRIGADARIDPIGKARRPQAEPFRLGQQPSLAFA

PREIASVGDANGRLKVRLFGLGMLGPNGPLPIHVTEIARDREESRRDPTLGNFLDIFHHRYLTLLYRAWA

SAQAAAGLDRPDDERFSFYVASLAGQDLDEVGARPLPAHARLSASPHLVREARNADGLRMTLERYFGVPV

TLEENVFHWIAVDPLEHSRLGRPGDASTMAAGALLGELVPDRQHKFRLVFGPLDIDAYLRFTPRGEDLPR

LVEWVRAFVGYEFEWELELRIKPNGAPPAVMGGPHQLGWSGWLGRSPSGEPVTGMRFEPEHYAHGFARGT

ARDTRGMRGER

>WP_004523300.1 MULTISPECIES: NAD(P)-dependent oxidoreductase [Burkholderia]

MEAIMKLGFVGLGVMGQPMALNLARAGTELVVWNRTRERCEPLRAAGAQVADSAADVYRRARIVILMMAT

DAAIDAVLDRGKPAFASNVAQHTIVQMGTVSAEYSRGLEADIRAAGGRYVEAPVSGSRQPAEAGRLVAML

AGEPAAVEEVRALLAPMCREIVATGQVPSGLLMKLAVNTFLIAMVTGLAEAAHAARGFGLDMKQFQAVLD

AGPMASSVSRVKIDKLVNEDFEVQASIVDVFKNSRLATEAAHGAHLAAPLLEVCCELYRETEALGHGQAD

MAAVVHAIEARSVARYAGS

>WP_004523020.1 hypothetical protein [Burkholderia pseudomallei]

MNRAIRMRAAVAAAAFSASLPAGAAGPAIDPGRYLYVEGGSAHGVLTVKGNAFEIETIGGNCHTCALSGT

FDGRVGIARDGENVCRIAVSGGHGDLRLDTSGSDACRDFCGMRASFDGEYRRPGAACTDRARDVRTERSH

RQYAAHDYDAARTTLKALLAECGGFMGWIELDRAKSDLALTEYHRGDRAQCVAVLSDTIAYRAQQDHSDA

FGLPPCDADNYKSTGDAILHNLALCQAPAKR

>WP_004522995.1 MULTISPECIES: VOC family protein [Burkholderia]

MSATPRGALRPFHLAFPVTSLEHARRFYGGLLGCPEGRSSDHWVDFDFFGHQLVAHLAPGETGRSAVNPV

DGDDVPVRHFGVVLSMDEWHALADTLKAAGTRFVIEPHIRFKGEAGEQATMFFVDPCGNALEFKAFADIG

QLFAK

>WP_004522937.1 NAD(P)H-dependent oxidoreductase subunit E [Burkholderia pseudomallei]

MGHIMDSYYRHHVFFCLNQREKGAERPSCANCGSQEMQEYAKKRVKELGLAGAGKVRVNKAGCLDRCEEG

PVVVVYPEGTWYTYVDKNDIDEIVESHLRDGQVVERLRI

>WP_004522781.1 MULTISPECIES: ATP-grasp domain-containing protein [Burkholderia]

MKTFLFVEARPIECAGIGYIRALGYAPVLFTSMQSRNKVLFDDLDLNLFDAVHHVDTLDARAMRERIDAL

RLPVGAVLGCYDDVMIPASELADALGLPHPSLAGLRRAYGKERVRDTLRGRGYRQPAYQVLSAERAAPRP

DIGFPCVVKPLRDAGAYGVSLCANEADYRAAVERFRSGDGVSMLGSRHREFLAEAFVEGPFYGAELLHNG

GRWHVLGINRIFVSPRDSLCMTGISHPSDLPAADADAIADEIVEWVNLLGLAGGALNVEFILAETGPVLV

EINLRIAGARAVRQIALTTGIDMVEHLIDFVCGIDRPIAPREPARYGFVADAFVFAPAAGVVRGVAFAPN

GAHYVASGFRKLPLESARAAKNFGSVIGYVLAHGRTCDEAMRHARALADGVRVELG

>WP_004522679.1 MULTISPECIES: SGNH/GDSL hydrolase family protein [Burkholderia]

MNPLQRQQQPAGAARRRFWRGAQVALASAAFALLAACGGGDDNGSSQPSAGVNMQVVSFGDSLSDVGTYS

PQILIGFGGGRFTTNPGQVWTQDVAAYYGGTLTPAFEGGFGVPLQAAGGLGYAQGGSRVTQQPGIGHADA

SVANADYAQATTVPVATQVQQYLQQHGSFNANQIVLVNGGANDIFYQVQVAQAQGNTPAAQLAAAQQIGL

AAQQLAGVVQQIVAAGATHVFVSNVPDIGGTPLAASTGQQAALTQLSTIFNSTLVAALKALNVDPAKAVL

IDAFTWQDGIAANYQGNGFSVANTGTACNLQSMIAAATKAGVANPTAFGSSLFCSPQMYTVANADQTYMF

ADTVHPTTRLHALFAQYVEQQIAKTGVGK

>WP_004522594.1 MULTISPECIES: NAD-binding protein [Burkholderia]

MQCAHGGYNAFLRINRYIMIATRILRRPRVLIVGCGDVGMRCAAQLRARRENLRILALTSRRSRCVELRA

AGVVPVVGDLDARATLKRIARVAPVVLHLAPPQATGDVDRRTQALVAALASPRRPSRQLAPAYGRLRAWR

TAARSARPPFQASAIVPDALPRPVVVYASTSGVYGDCGGARVDETRAVRPANPRARRRVSAERQLRRATA

RGALSARIVRIPGIYAANRLPLARLEKGTPALVEADDVYTNHIHADDLASILLRAAVRGKPARVVHASDD

TELKMGDYFERVARAFGLRSPPRIARAEAERQLEPMLLSFMRESRRLANARMKRELRIALRYPSVDDFLR

TVSAPRPLK

>WP_004522359.1 D-glycero-beta-D-manno-heptose-7-phosphate kinase [Burkholderia pseudomallei]

MSSPSLPSADKRASLAATARAVSREQIAKSRVLVVGDVMLDRYWFGNVNRISPEAPVPVVHVQRQEERLG

GAANVARNAVTLGGQAGLLCVVGRDEPGERIVELLGESGVTPYLERDPSLPTTIKLRVLAQQQQLLRVDF

EASPTHEVLLAGLARFDALLPSYDVVLMSDYAKGGLTHVTTMIAKARAAGKPVLVDPKGADWARYRGASL

ITPNRAELREVVGRWTSEDDLRARVTKLREALAFDALLLTRSEEGMTLFSHEGELHVSALAREVYDVSGA

GDTVIATVATMLGAGLPLVEAVVLANRAAGIVVGKLGTATVEYDELFH

>WP_004522272.1 MULTISPECIES: urea ABC transporter permease subunit UrtB [Burkholderia]

MPMPLSRAARALAALAACAAFSFAAPRAALAVTAADVAALAGDDFDAKRAAIDRLAAGHDAAAAALLNAL

ANGDALATDAGRILIQHGDAARDALTNAPAQAGDAQPVMLNNLLRTKIANALSGLDLASPDIDTRRRAID

ALLKRPDAALKPMIDAARAKETDPVLKRRLDALWAIAALRDADPAKRLEAVRLVAARSDLDMIEQLRPLV

AKRPDGGDAEPDARVREAAQQGLGALYAIQRRGEIAGTLFAGLSLGSVLLLAALGLAITYGLIGVINMAH

GEFLMIGAYATYVVQTLVQRYLPGAFDWYPLAAIPVSFAAAAALGIVLERTVLRHLYGRPLETLLATFGV

SLILIQATRMIFGAQNVQVVNPSWMSGGVTVMQNLILPYNRLAILAFALVVVGIAWAVLTKTRLGLFVRA

VTQNRRMAACVGVKTARVDSYAFAFGAGIAGLGGCALSQIGNVGPDLGQSYIVDSFMAVVLGGVGQIAGT

VLGGFGLGLVSKAIEPFWGAVLAKIAVLVMIVLFIQKRPQGMFALKGRSAEA

>WP_004522075.1 4-hydroxy-tetrahydrodipicolinate reductase [Burkholderia pseudomallei]

MSSMKIAIAGASGRMGRMLIEAVLAAPDATLAGALDRTGSPQLGQDAGAFLGKQTGVALTDDIERVCAEA

DYLIDFTRPEGTLAHLDAALRHDVKLVIGTTGFSEPQKAQLRAAGGKIALVFSANMSVGVNVTMKLLEFA

AKQFAQGYDIEIIEAHHRHKVDAPSGTALMMGETIAAATGRTLDDCAVYGRHGVTGERDPSTIGFSAIRG

GDIVGDHTVLFAGIGERIEITHKSASRVSYAQGALRAARFLAGHQAGFFDMQDVLGLR

>WP_004521661.1 MULTISPECIES: Flp family type IVb pilin [Burkholderia]

MKTQGTRINMKQLMHRFFKEEAGVTAIEYGLIAGLIAVAIATTVGTVGTDLSALFSTIASKLPAA

>WP_004521599.1 MULTISPECIES: type II and III secretion system protein family protein [Burkholderia]

MWSVIDAIGKRRQAASASPRARAFAAARRAAAAALWWLALALGMAVAPRFARAADLGPVLAVPAGGGEMV

KLPAPAVAVFVADPDVADVHVPTPQAVFVLGKKAGTTTLFALGANNRTILRETVVVDVDTPSLQRILDAR

FPQLRLTLAGAPGSLMVSGRVPSAADADAVVQTLKPYLRQQEALVNRLTLARPIQVHLRVRITEVDRNIT

QQLGINWSALGASGNFVGGLFNGRTLFDTASKAFDLSPSGAFSVVGGFHTSRYSIDGVLDALDQEGLITM

LAEPNLTAISGQTASFLAGGEFPIPVAQDTTGAITIQFKPYGVSLDFTPTVLADNRISLKVRPEVSEIDP

TNSVTTGSIKVPALTVRRVDTTVELSSGQSFAIGGLLQSKSSDVLAELPGLARLPVLGKLFSSRNYLNDK

TEVVVIVTPYIVQPANPGELRDALDDVTRPSSDIEFVLQRSLGIDPLGGDAPRLAGPAGFVY

>WP_004521555.1 MULTISPECIES: hypothetical protein [pseudomallei group]

MTIGRWSDAARLQYPERVDLPGAAMKRFLSCVALVCFALPAAHAQTQTYHFEEGGTTPTGVTSQPASAAA

QARTQRAKPHRHPVRRNKHHRTHRRARSAQNDRFYSQP

>WP_004521511.1 MULTISPECIES: GNAT family N-acetyltransferase [Burkholderia]

MMSAFSLTHAHLDFGHEGTRMTTFPISTARLVLRPFKRGDLAAFTAYRNHPDVARYQSWASYTDADAKAF

FAEQQTLRFDTDGTWFQIAAEHQANGALVGDVAVHFFDEGRQAELGVTFDVAAQRQGFALEAVSRVIELL

FADLGKHRIVATVDALNVRAQRLLERQGFRREGHYRENIFFKGAWGDEYGYALLNREWRALRQAAAGGTQ

>WP_004521417.1 MULTISPECIES: cation:proton antiporter [Burkholderia]

MADWILQLCVVVVVAFTLGALAQRLGQGRVVGELLAGLLLGPSAFGALTVAGHGALIGPQTAALLSRLGE

LGLVFLMFQTGMHITWTTAHGRSSGLVATVVAGFGMALPFATGCAIAVAASGSFQPQASHLAYVLFCGIA

LSVSALPVMMRIIADARIAGRACATLSILAATLTDSLGWLMLAAVGTLATSGLSPASTVHAMGVLVAFVV

VSISLVRPIVLGVLDRTRRAGSGSAMLICALCYVLLSSWAAARLGFHGAFGALVAAANLAGRHDLRQLWD

ARFAGLADLVLIPLFFVSMGFQASFSALDTGAAWGWLAAFLVGGVATKFAGCYAGARLCGIDRAEATLVG

VLMNCRGTVELMVLAIGLQLRIIPASLYTVLLIATLTMTWLSAMSTHRLAKRRPLRSPPAGASDAPAGFR

ATQAQAAGGDARLPGLPEASRPSR

>WP_004521218.1 2-oxo-4-hydroxy-4-carboxy-5-ureidoimidazoline decarboxylase [Burkholderia pseudomallei]

MMKAMHYTLEQLNAMSTDAFVAALSGIFEHSPWVAEAAAAARPFGSIDALHGTMKDAVEAAGDARQLALI

NAHPELAGKAAVRGELTAESTREQSGAGLDRCTQAEFDRLQQLNRAYREKFGFPFILAVRGFDRHGIIAN

FEARVGHTRDEELRASLAQIYRIARFRLDDLIGA

>WP_004266412.1 MULTISPECIES: sulfate ABC transporter substrate-binding protein [Burkholderia]

MVKRNTGLAGGARRLIASLALGAAAALGALTPALADTTFLNVSYDPTRELYQDVNQAFGKEWKARTGETV

NFKQSHGGSGAQARSVLDGLQADVVTLALAYDIDALANKGLVSKDWQKRLPDNASPYTSTIVFLVRKGNP

KGIKDWDDLVKPGVSIVTPNPKTSGGARWNYLAAWAYAQHQPGGTAQTAKDFVTKLYRNAGVLDSGARGA

TTSFVQRGIGDVLIAWENEAFLSIKEFGADKFEIVVPSASILAEPPVAVVDKVVDKKGTRKLADAYLNFL

YSRQGQEIAARNYYRPRSRDVPAALTKQFPKLKLYTVDDTFGGWTQAQKTHFADGGVFDSIYKPQ

>WP_004266343.1 MULTISPECIES: H-NS histone family protein [pseudomallei group]

MVMATYKELLAQLDDLKQQAKRARALELPDVLVGLRKKIVEYGLSQKDLFPPRLGRPKKADALPKPRYRD

PDTGATWTGRGRAPAWIAGQDRERFRID

>WP_004206780.1 MULTISPECIES: aldo/keto reductase [pseudomallei group]

MALRSLGTSTIQVSPLVFGGNVFGWTADENTSFSLLDALADAGINFIDTADVYSAWVPGNQGGESETIIG

KWLKRSGKRDQVVIATKVGLLETRAGLSRENILKAADDSLRRLQTDYIDLYFSHRDLADTAPLEETLGAY

QTLIEQGKVRIIGASNYSGARLREAAELSRRTGLPAYQVIQPEYNLYDREAYERDLEPAATELRLGVVTY

YALASGFLSGKYRSEADLKKSARGRRVEQYLNPRGLRILAALDAVAAKHGSTQTSVALAWQMARPSVTAP

IASATSLEQLSALGAAIRLQLDAHDIRQLDDASAP

>WP_004206544.1 MULTISPECIES: DUF4148 domain-containing protein [pseudomallei group]

MEVHIMKTALTLKTAALALALAAPALSFAQPSSDTSITRAQVRDELIQLERAGYQPSKNRYPANLQAAEA

RIAPMNGGADMSGYGGTTGGAMQSGQRVSPTSGGWDTPYRHR

>WP_004206353.1 MULTISPECIES: isoprenylcysteine carboxylmethyltransferase family protein [Burkholderia]

MATTRRHATATALAAAARPCAAPHADSFLGILIEVFARVVAALALGVFAYAAYLQWRLDPSRITLILLAV

STTLTVGLSLFAEPPKKRDWSPLALFFSVGGTFYYLVFQLTASRQLLPEAAGAAIQLIGLFWQLFAKVSL

RKSFGILPANRGIVSRGAYRFVRHPMYLGYLIIDAGFLLTNFSARNLIAVALQIGLQLGRIRREERLLSE

DAAYRAYRRSVRYRVIPGLF

>WP_004204912.1 MULTISPECIES: type VI secretion system baseplate subunit TssE [Burkholderia]

MKRFEPSFLDKLFDDEPHVPAPAAMRQLSLEELKNTVARDVEAILNTRIALTEEDLAELPECQRSVLTYG

LNDFAGLSLASHYDRTFICKSIQQAIARHEPRLQQVQVAFELNEQSTNALYFAIQALLVVHPAEEPVTFD

AMLQPSTLQYSVTRSRAAKV

>WP_004202877.1 MULTISPECIES: MarR family transcriptional regulator [Burkholderia]

MTMSGLYDPEHIELESSLGYYLTKARQALVERLDRALGPLELTAQQISVILLLARGYARTPFELSRKLSY

DSGSMTRMLDRLEKKGFVVRARSESDRRVIELALTERGAHAARALPALIATELNAQLEGFSADELALLTD

LLRRFIANAPGAADAACAEPPPDQR

>WP_004202737.1 MULTISPECIES: GNAT family N-acetyltransferase [Burkholderia]

MNKPDLRPAESVGIAGPAPVLVRELASGDRQQLLTHFLALGEEDRLLRFGQAVPDHVIENYVRTMDFGRD

TVFGVFDHALELIGVGHLAYLPAEGDKRTAEFGVSVLERARGQGVGTKLFERAAIRSRNTHVTTLYMHCL

SRNATMMHIAKKSGMRIEYAYGEADAYLSLPPADHSTIIAEMMQEQAAVFDYALKRQARRATQIFESLLP

AGLTA

>WP_004202616.1 MULTISPECIES: enoyl-CoA hydratase/isomerase [pseudomallei group]

MDMLSRPADYRTIRVRYDGDICFLQLHRPDAQNAINNRMIAECMDVLDRCEHAAKIVVLQGLPDVFCFGA

DFSDIAEKPDALIDSDAIYGLWHRLATGPYITIAHVQGKVNAGGIGFVAASDIAICDENVPFSLSELLFG

LIPACVLPFLARRIGTPKAHYMTLMTQPVTAQQAFSWGLVDAIGANTDTLVRKHLLRLRCLNKASVARYK

SYAATLDDTLAAARPGAVRVSIEMFADPENRRKIARYVDTGKFPWEAD

>WP_004202011.1 MULTISPECIES: ferritin-like domain-containing protein [pseudomallei group]

MSSVPISSSEPLRCARSAALAALGESDPARKVALVAELHAALADGRAAVFPERELSAPARGVPGRPARPE

LVEPRRLERRSMRSPQGRAVLLHALAHIEFNAINLALDAVWRFARMPTAFYADWLKVAAEEAHHFSLLAA

RLAEFGHAYGDFPAHDGLWEMCERTAGDVLARMALVPRTLEARGLDASPPIRARLQQAGDHASAAILDVI

LRDEIGHVWIGNRWFRHLCDAAGLDPHPTYERLAGQYRAPRLRGPFNFDARRAAGFNDDELNALVAQDAD

PNG

>WP_004201791.1 MULTISPECIES: hypothetical protein [pseudomallei group]

MRIAACLARRRHSIAAALAASLAIACPYVTAAHENETYRIVDTRPEHVAARGRAERCARVDDGAFARQRD

HAPMPVPVATPRPLVDGTAVTLWDEIGPPAPMPVPTDARHTVRGDGANYTRQ

>WP_004201765.1 MULTISPECIES: phage tail assembly chaperone [pseudomallei group]

MERTMLSPHEFATLLLVKDAPDQADMDRDELDALLERQLVKLEALGSGRKYCVTEIGDAALRSIKLRYS

>WP_004201611.1 MULTISPECIES: VOC family protein [pseudomallei group]

MYKELAMTTANNESVIAWFDIPSVDFDRAIRFYETVLQTALQREVIGGVPTAVFVHPEAATGGAVVYDPQ

RLKPGADGTVVYLNANGPVTAALERAKRAGGIVQGAVIELPNNYGYIGYLIDTEGNRVGLHSLKCS

>WP_004201477.1 MULTISPECIES: NADH-quinone oxidoreductase subunit NuoN [pseudomallei group]

MQNAPMNVLLPDALVMAAIIVAWLNDTFVGAAGRRLTYLIAVVASAAAGVWFALQALDPQQYYFFSRMVV

VDSFASAMKAVVSIGFAVTLVYSRKYLEDRDLFRGDVFLLGMFSLLGQLVMISGNHFLTLYLGLELMSLS

LYAVIALRRDAAQSSEAAMKYYVLGALASGFVLYGISMLYGATGSLELNEVLKAVASGRINDAVLLFGVI

FIVAGVAFKLGAVPFHMWVPDVYQGAPTAMTLFVGGGPKVAAFAWGLRFLVMGLLPLAVDWQEMLVILAA

LSLIVGNLTGIVQRNIKRMLAYSAISNMGFVLLGLLAGIVNGNPSAAASAYSSAMFYTIVYLVTTLGSFG

VVMLLARRDFEAETLDDFKGLNKRSPVFAFVMMVMMFSLAGIPPTVGFYAKLAVLEATVNAGLTWLAVLA

VVTSLFGAFYYLRIVKLMYFDAPQDTTPIAGDACKRAILVLNGLAVVVLGIVPGPLMTICLQAISHTLPL

>WP_004200917.1 MULTISPECIES: diiron oxygenase [Burkholderia]

MIMDAYRSPFDDWHQAASVRSMPADAWLAGGAGAWIAPQAGGVLAGLPARDARRERAAGGLLIAHLSFTV

ALENTLISPVARDIAARALHADYDDGVVADALRVQCDEAYHALLAQELMTRVRAATGADKPRRTPGFLRH

VERLAATLPGVDAPLLRFCAAVVAETLITHTLRDDWRDEGLQPDVRTFLRRHYLDEARHSAYFSRLLTLL

WPQWPAGVRDALRPLWSGLIDAFLFADARIAAEALAAAGLTACEIDRAMSACGAPDAMQRRRAQSARQTL

HALRAAGALEAGESAAIALGNA

>WP_004200750.1 MULTISPECIES: acyl-CoA thioesterase [pseudomallei group]

MEASIMTDSPMQLPQKPPALRVVPQPSDANVHGDVFGGWIMAQVDIAGSIPASRRANGRVATVAVNSFLF

KQPVFVGDLLSFYASIVKTGNTSVTVDVEVYAQRMSLMSEIVKVTEATLTYVATDSDRRPRALPPLE

>WP_004200457.1 MULTISPECIES: hypothetical protein [Burkholderia]

MHLELVYAGPVDAAVALFAAAADGCDGGAAAQAAAAPAATPGDVIVAGAGRPAANFVAKPARIKRARIDV

VAAVDIGTLARRTAVPRAASPSRRAPSTSDAVRAIADTTRRAIPSEAFDVPQPMPASAREAGDGMRGGVG

GAKAGAWTLYDFKEAKHTVLKS

>WP_004200216.1 MULTISPECIES: DUF484 family protein [pseudomallei group]

MPKPERSAMNDREVADYLLANPEFFAQHAELLATIKLANPHGKAAVSLQERQMEMLREKNKHLERRLAEL

LRYGHENDGLAAKFNRWTARVIAERDPYALPRAIAGGLADVFDVPQTALRLWDVAETYAQADFARNVGEE

VRLFTNSLATPYCGANTGFEAAQWLTPVAAPPAADDGEAPAASGAAESVALIALRASAEGEGAGAFGLLV

LGSPDPRRFHDGMGTDFLTQIGTLASAALTRLLPH

>WP_004200135.1 MULTISPECIES: hypothetical protein [pseudomallei group]

MNQPARSPAVAGMLNKVPEVTLAFWIVKIMSTTVGETGADYLAVHVGLGAAVTGALTACLLVGALALQWR

ARAYVPWIYWLNIVLVSVAGTQITDALTDGLGVSLHVSTPLFAAALAATFALWYRSERTLSIHTIVTPRR

EGFYWAAILLTFALGTAAGDLATEALGLGFRLGIVAFGGAIALVAAAALGGMNRVLAFWLAYILTRPLGA

SLGDFLSQAQAYGGLGLGTIRTSAVFLAVIVALVAGLSVAAARAGRLGARGR

>WP_004200023.1 MULTISPECIES: chemotaxis response regulator protein-glutamate methylesterase [pseudomallei group]

MNAVQKKIKVLCVDDSALIRSLMTEIINSQPDMEVCATAPDPLVARELIKQHNPDVLTLDVEMPRMDGLD

FLEKLMRLRPMPVVMVSSLTERGSEITLRALELGAVDFVTKPRVGIRDGMLDYSEKLADKVRAASRARVR

QNPQPHAAAAAAAHGHAGAAAPLINNPLVSTEKLIIVGASTGGTEAIREVLTPLPPDAPAVLIAQHMPPG

FTRSFAQRLNGLCRISVKEAEHGERVLPGHAYIAPGHAHLLLARSGANYIAHLSDEPPVNRHRPSVDVLF

RSAAQHAGKNALGVILTGMGRDGAAGLLEMKKAGAYTFAQDEASCVVFGMPREAIAMGGVDDVAPLSDMS

RRIMARLASMGDRVQRV

>WP_004199963.1 MULTISPECIES: CidA/LrgA family protein [Burkholderia]

MSRISATAATATRPTTAAPARAGRVARIALQTAALGALWAAIDWTVRAFGVPVPSGVVGLAVLLALLLSG

RVAPGWVKDGANWLLSDMLLFFIPATVAAVQYGGLFKADGWRLALVVVAGTTFVMLSVAIAVDVAAGFER

RLAVMRVRAGRRRARA

>WP_004199890.1 MULTISPECIES: ClpXP protease specificity-enhancing factor [Burkholderia]

MQEISTKPYLLRALYEWCTDNGYTPHIAVRVDKSTRVPRQFVRDGEIVLNISFEATSQLQMGNEWIEFTA

RFSGKAHKLEVPVANVLAIYARENGQGMAFQVDVAAEMGEADGSDALAVAEEGDDAGARHASADGAAEAA

PEPGDAAEELPKSDGDGDGDGAKGSRPRLKIVK

>WP_004199876.1 MULTISPECIES: ABC transporter permease [pseudomallei group]

MTDMTLPTPLGTLTSLEDEERAAQRRLRRRRQLIVGLRIAVLVAVLGGWEIAARLKWIDPFFFSMPSLIA

AQIQDWFVNGTSQGPLLLQVWVTLEETIAGFLIGSVAGIFCGIVLGRNKLLADVFGLYIQIANSIPRVVL

GSVFVIALGLGMASKIALAVVMVFFVVFGNAFQGVREADRYLIANAQILGASRRQITTSVVIPSALSWIL

ASLHVSFGFALVGAVVGEFLGSKQGIGLLISTAQGAFNASGVFAAMIVLAVVALAADFLLTRLEKRLLKW

RPAAF

>WP_004199535.1 MULTISPECIES: OmpH family outer membrane protein [pseudomallei group]

MLTGKFSKRVMCALALAAALGATAARAQDVARIAAVNSDRILRESAPAKAAQTKLEAEFAKRDKDLQDMA

ARLKSMSDALDRNGPTMSANDRAQKQRDLAQLDTDFQRKQREFREDLNQRRNEELAAVLDKANKVIKQIA

EQQNYDLIVQEAVYVSPRIDITDKVLKALASPSSLSN

>WP_004198489.1 MULTISPECIES: hypothetical protein [pseudomallei group]

MPKKDRTMNHHTNQLHPSTSFAFVAASWAALLAGVCAYLLGLWNAGMQLNEKGYYFTVLAYGLFAAVSLQ

KSVRDRLEGVPVTGLYYGLAWASVILTIALLVIGLFNATLLLSEKGFYAMSFALALFGSVAVQKNTRDLK

AAGRGRAETEVAVDIAE

>WP_004198225.1 MULTISPECIES: DUF799 domain-containing protein [Burkholderia]

MFKFITARRLAAASVLALLAACAAPAKHVDYTAFKKSQPRAILLLPPQNDTSDVKATYGLLSQMTRPVAE

AGYYVVPVAVMEETFKQNGLTNAADIQTVSPAKLRDIFGADAALYTTVTQYGSVYTVLDSTTVVSASAKL

VDLRTGDLLWQGSGRATGKELGSNVDFSGFGLIATLAQAAVKQIAHTLSDDAVDVAGLTSGRMLSAGQPN

GLLYGPRSPKYGTD

>WP_004198203.1 MULTISPECIES: ATPase [Burkholderia]

MHQWHFDGSTELKQAMGNDIFLIGIDGGGTGTRAVLADARGRELAQGSAGPSGLALGIEAAWRSIEAASA

EACARAGVAFDWRHCVLGCGLAGVNHRAWLNAFRASAPLAALAIESDAYTTVVGAHGGAPGVIVALGTGS

IAAALDASGACRIAGGYGFPSGDEASGAWLGLRALSYAQQALDGRAPLDALARALVAHTGAPDRDALIVW

SCEANQTAYAQLAPIVLAHRDHPAAAHWIEQAGIEIGRMIDALDPAAALPVALCGGLAQALASAVPEPHG

ARLVAPQADSAHGALRLAEREAARLGLTAPR

>WP_004197835.1 MULTISPECIES: hypothetical protein [Burkholderia]

MQFGLLLSQLPHVDESRVHYDAMLSLKAAGEPASASFWQRLKALIS

>WP_004197715.1 MULTISPECIES: MFS transporter [Burkholderia]

MSSDRPATRLATRLAFLVAGFGVACWAPLVPFAKARLGVDDAVLGALLLCVGLGSVIAMLITGPLGTRYG

SRPIIVAGGVALAAILPLLSVANTTPALGFALLAFGAALGSLDVAMNVHAIEVEHAAARPLMSGFHALFS

VGGIAGSSVMTFLLSMRVGAFASTLLCSAPMLAAILVARPRLLRAARAAARPRFAMPRGVVPLLAMLAAI

TFLAEGALLDWSALLITDKGLVAAAQGGLGYLLFSIAMTAGRLGGDAIAARVGDRSTMFWGGLLAVAGFV

MLLAAPIAGVAMAGFLLIGLGASNIVPVLFRRAGAQQAMPSALAVTLITITGYAGHLAGPAGMGFVARSV

GLESAFWMLAALLGIVPLCARVVTANRT

>WP_004196851.1 MULTISPECIES: c-type cytochrome [Burkholderia]

MTIAKRVKRTMSAAAAAMAVVSCAMAAAPAAHADAGDGLKVARSNACMGCHAVDRKLVGPSFQQIAERYK

NDKQAEPKLAKKVKDGGSGVWGAIPMPAHPRMSDADVRSVVQWVLAGAPSK

>WP_004196094.1 MULTISPECIES: TetR/AcrR family transcriptional regulator [pseudomallei group]

MLDFPAMKPIRLTREQSKDLTRERLLSAAHAIFTKKGYVAASVEDIASAAGYTRGAFYSNFRSKAELLIE

LLKRDHEEAEADLQKIFESGGTREQMEAHALEYYSQFFRNNPAFLLWGEAKLQATRDAKFRARFNEFVKE

KRDRFTHYILTFAERVGTPLLLPADVLALGLMSLCDGVQSYHAADPRHVTGDAAQQVLAGFFARVVLARA

PD

>WP_004196054.1 MULTISPECIES: hypothetical protein [pseudomallei group]

MERCVMKTSITTTTEPRAVARGARLPGWMWFALLWMGGVAGAVTLGYAFKLFMYATLFAVK

>WP_004195923.1 MULTISPECIES: lipoprotein-releasing ABC transporter ATP-binding protein LolD [Burkholderia]

MNDRVFEQTMNQNHQDGGARECVLEARGVTKTFVQGGFNVQVLDNAQVSVRRGEKLAIVGASGSGKSTLL

HVLGGLDEPSAGQVSLLGKPFTQLAERERNELRNRALGFVYQFHHLLPEFTALDNVAMPLRIRRMSTEEA

RRHAREMLEQVGLGARAKHRPGELSGGERQRVAIARALVTKPACVLADEPTGNLDGSTADHVFHLMLELS

RTLDTSFVIVTHDPDLAARCDRILRLRDGVLHEEPAVPV

>WP_004195907.1 MULTISPECIES: tetratricopeptide repeat protein [Burkholderia]

MKPSRGRAPSAATLVATAAVSAALALLPGAAALAQKAPAPTDGTPEIDASITNRNWAQALAQLDARIASN

PRDVQAQFKRGTVLARLNRDDDAIKQFVELTQAYPELPEPYNNLAALYAKHGRYDDARSALVTATHANPN

YGLAYENLGDLYLRLAAESYKRAQSLGRTTGASAQRLSDLQKIIAPPKTRAAAAPAPAARDYSARAAANV

TTTTLPLSPTFQFGGPSGMLAMPPYAAPSQ

>WP_004195797.1 MULTISPECIES: DUF3717 domain-containing protein [Burkholderia]

MSDISIHELEAAINFWRARSPASGDELELCEEASALSKPYALLIVQRQSALQLEGLDPKARNAWETYVRL

NNGLEG

>WP_004195328.1 MULTISPECIES: ferritin-like domain-containing protein [Burkholderia]

MQPYEDAARSQRWSMPVDALMMGDYEYRETALTNLYHKAKAAQWDVNRDIDWNVTLDPDNPLGMPDGMLL

VFGTDLWHKLGAAERAEMRHHWQGWLLSQILHGEQAALICASKLASAEQSLPARLCAAAQIMDEARHVEA

YAKLVNEKISVRYPMSRSLEGLLKDTVTSSALDITNLGMQVLVEGIALSTFQTVVAFSADPFVKDLFARI

QRDEARHFAVGRLTLARVYSDMTSAEMKEREEFVSEGASVLYEHLCADDIWEPLGYSKQACAQMVRESEV

SRALRRSIFRRLVPTIREIGLLTPRVRRTFEQLDVIDYEDMPLALDM

>WP_004195111.1 MULTISPECIES: CNP1-like family protein [Burkholderia]

MKAIALAAASIAAAAALAGCAHSNTPSNKDDSEFVYLLDRQPQWTENKVDKLPPLPQTSDLLPFNVSQNT

PLKFFVDSKSLDVGTDGVVRYIVVVTSPAGARNVNYEGIRCDTYEWRQYAGLNADHSGWDRTIETDWQRI

ENGELNAYHAALYQDFFCANKMPAAKRPTIIENIRYNRTQLNQIR

>WP_004194461.1 MULTISPECIES: hypothetical protein [Burkholderia]

MRPARAGRAFRFAVSRFAVSRFAMPIFVVLFALAALIWGAIHMFEAIAARFGDAVAIGAATVAAVAIAAA

IARSVRRRRDIAPNAREDGWTHVLQRAWGDLRISATKGLLWLSQDGADGRYTLTDLSGCRAETLDGRWYL

VVAVRDDRRAEWKLPMDDKRDALRWARVLTLAKRQRL

>WP_004194433.1 MULTISPECIES: polyisoprenoid-binding protein [Burkholderia]

MNKQLMIAAGALAAALSFSAHAAPATYQFDPSHTYPSFEADHFGGLSVWRGKFDRSSGTVTLDRAAKTGT

VDVTTKVASIATGSQKLDEHLQTADFFDSAKYPDATYKGTIKFEGDRPAEVVGNLTLHGVTKPVTLKIDS

FKCMPHPMLKKEVCGVDAVGEFNRDDFGLDYGKQYGFKMKTKLLITAEAVKQ

>WP_004194201.1 MULTISPECIES: phosphatidylglycerophosphatase A [Burkholderia]

MPTDPTPRPADSADSASQPGATPAPASSPAPRRDSPQDPQRIARRRATVRFMFSHPVHIVSLGFGSGLAP

FMPGTFGTLFGWLTFVALNRYLTVPEWWALIVAGFVAGIWMTGFTAKKMGIADPGPAVWDEIVAIWLVML

LVTPATFVEQLWAFVVFRFFDMVKPPPIRYFDRNLKGGFGIMFDDLIAALMTLFVIALWRSFGVQ

>WP_004193758.1 MULTISPECIES: efflux RND transporter periplasmic adaptor subunit [pseudomallei group]

MNRSGSRAALLIGVALIAAACHRKEAAPSAPRPVVAVPAQADGAAAAVSLPGEIQPRYATPLSFRIAGKL

VERKVRLGDIVKKGQVVALLDTSDVARSAASAQAQLDAATHALTFAQQQRERDRAQARENLIAPAQLEQT

ENAYASARAQRDQAAQQLALAKNQLQYATLVADHAGYITAEQADTGQNVSAGQPVYQLAWSGDIDVVTDA

PESELPRLAPGRTARVTLPALPGRAFDARVREIAPAADPQSRTYRVKLTLAQPDAAVRLGMTANVALSPD

APSAANAQPRFTLPSTALFHAGNAPALWIVRKQDDTLELRRVDVVRYNERTVTVSGGIQPGERVVLQGVH

TVSAGEKVRAVPPLHPEDVAS

>WP_004193180.1 MULTISPECIES: RNA polymerase factor sigma-70 [Burkholderia]

MAEVLARPAPAKPLPILASNGAFAPAAAPVLRAPSRPAARTPGAREHGALVDVLVANRPMLVKLARGFVG

CASRAEDVVHDVFVKLVDFPNQDAIRQPVAYVTRMVRNASIDACRRQTLENTYHADEDDGLDVPSPELSP

EAALVVRDTLRHVYDALAQLPARSRAAFEMVRLREETLQSAARALNVSQTLVHFMVRDAERHCVACVDAS

ERGLACPAFCGARARTVKKCVRDSSIE

>WP_004193138.1 MULTISPECIES: hypothetical protein [Burkholderia]

MQTITMEQCKRIHGGDNLIDGTFNLLGGTFTINSSSLSTLLTNANTLVVNANNWLTNPKGTLSSTKK

>WP_004192981.1 MULTISPECIES: PAS domain-containing sensor histidine kinase [Burkholderia]

MVLKNLIKARAGHPERLSDDDRLVRSGLLTGLEALPTVVLVLDRRTLRIAFANPSAEAMLDISRRQLSQM

PWGEIFPNASELATTITAIGEERFHATHLDTVLDRPGREPLHVHAIVGFLEGAPEFVLVELFENERQSRT

DREERIHDLTAVNKQLIRNLAHEIKNPLGGIRGAAQLLEFELGERERDELREYTQVIIKESDRLQTLVDR

LLEPHRHPHIVGDVNIHEVCERVRAVMLAEFPRGLTIERDYDVSVPDLRGDKEQLIQALLNIVRNAAQAL

RERIAQGDARIELRTRVARKVTIAKRLYRLALDLRVIDNGPGIPDDIRDRIFYPLVSGRDDGSGLGLTLA

QTFIQQHDGMIEVDSRPGRTEFQILLPLDH

>WP_004192905.1 MULTISPECIES: hypothetical protein [Burkholderia]

MNNPLRLIARLLIPFATLAAAPAASADSQALDAALDCSSTGHAFVAPLVASGAVRSQPMHVEANSMNAFR

TNRSLSAYGFSVYVVLGYQANDPLFAHGDGEPIGDWAYGVVVRGSKQAVEAKVRAAGSQAVVKDAFPFLT

AIVCTSP

>WP_004192871.1 MULTISPECIES: SpoVR family protein [Burkholderia]

MTTRHLHNEARGYEPRRRSDEASGAAAHERDASPEAAAQHPEAPASGLKEARMNVADRKPLPCPSDWTFE

LIEEYDTHISQVAEQYELDIYPIQLELISAEQMMDAYASVGMPVNYRHWSFGKHFLSTEKSYRRGQMGLA

YEIVINSNPCIAYLMEENTMTMQALVIAHAAYGHNSFFKGNYLFRLWTDAHAIIDYLVYAKNYVAECEER

YGLDRVEELLDSCHALMNYGVDRYKRPQKPSLAKESELRREREAYLQSQVNELWRTLPAKKPEFADEEQE

ERYPPEPQENLLYFAEKNAPFLEPWEREVIRIVRKIGQYFYPQRQTQVMNEGWATFWHYTLLNTMYNQGK

LADGFMMEFLHSHSNVIYQPPVTKPYYSGINPYALGFSMMSDIRRICEAPTEEDHKWFPELAGSPWLPAL

HYAMRNFKDESFIAQYLSPHLIREMRLFSVLDDDMRDALEVSAIHDDSGYQYVRQALSRQYDIHHREPNI

QVWSVNTRGDRSLTLRHFMTDNRHLSNDSEEVLKHMARLWQFDVYLESVDETGTVRKRYECRYTAPEIRL

>WP_004192776.1 MULTISPECIES: MFS transporter [Burkholderia]

MAMSWTREQRNVTIAAYLGWTLDAFDFFLMVFVLKDIAAEFNTKIPAVAFAITLTLAARPIGALIFGRLA

DHFGRRPTLMINIACYSLLELASGFAPSLAALLVLRTLFGVAMGGEWGVGSALTMETVPPRARGAVSGLL

QAGYPSGYLLASVVFGLLYPYIGWRGMFMIGVLPALLVLYVRAKVPESPAWKQMEKRARPGLVATLKQNW

KLSIYAVVLMTAFNFFSHGTQDLYPTFLREQHHFDPHTVSWITIVLNIGAIVGGLTFGWLSERIGRRRAI

FIAAMIALPVLPLWAFSTGALALAAGAFLMQISVQGAWGVIPVHLNEISPDEIRATFPGFVYQLGNLLAS

GNATLQAQFAVDHGNNYGMALATVAGIVAVVICVLIVFSRERRGIDMTQTAAMSPTSG

>WP_004192187.1 MULTISPECIES: SdpI family protein [Burkholderia]

MLDRPETVSYLLASALFFALAVPLAAQRIAPNRFYGVRTRATLRNPALWYRRNRVFGVALMITSATFIVV

AEYCRLRGVRLPDAALLAAFVAEIAVPIAVCFAAGRPPETPETPSRRAGDGRRR

>WP_004192020.1 MULTISPECIES: DegQ family serine endoprotease [Burkholderia]

MMNPSLRTWLVAAAVTALTPLAAQSATAAPNVTTTPAATGAVPAARAGLPDFADLVERVGPAVVNIRTTA

NVPADTRGALPPGLDNGDMSEFFRRFFGIPLPQGPGGQKNAPSTPDAPDTEQNRGVGSGFILSPDGYVMT

NAHVVDDADTIYVTLTDKREFKAKLIGVDERTDVAIVKINASSLPTVAIGDSNRVRVGEWVVAIGSPFGL

DNTVTAGIVSAKGRNTGDYLPFIQTDVAVNPGNSGGPLINMQGEVIGINSQIYSRTGGFMGISFAIPIDE

AMRVAEQLKASGKVTRGRIAVAIGEVTKEVADSIGLPKAEGALVSSVEPGGPADKAGLQPGDIILKFNGR

PVEAASDLPRMVGDTKPGAKATVTVWRKGQSRDLPITIAEFPADKAAKADSRQAPQQKPRSSALGLTVSD

LSPEQLKTLKLRNGVQIDAVDGPASRAGLQRGDIVLRVGDVDITSAKQFVDVTSKLDPQRAVAVLVRRGE

NTQFIPIRPRQK

>WP_004191954.1 MULTISPECIES: Mth938-like domain-containing protein [Burkholderia]

MFRPFSTPRKTDLKLHQDPSNALNTVTGYGPDYVDINLQRHETSVIVLPGAPVVEWPVASFDALTPELFA

MLLEPQPEVVVFGSGARLRFPHPRLTAQLTAQRVGVETMDFQAACRTYNILMAEGRKVAAALLIER

>WP_004191793.1 MULTISPECIES: hypothetical protein [Burkholderia]

MNWHCYVARELAHLEVAIGVLEKARKEFVHHTAVCDPAYWRLRLDTIRERLDEDPTLERQMSELLARIDR

LEARNPRRESADFLA

>WP_004190826.1 MULTISPECIES: TOBE domain-containing protein [Burkholderia]

MTADSLSSRSDARAARESLALSGELWLHAGGQTLGGAARIALLAAIGETGSITRAAKAVGLSYKGAWDAI

DTMNNLAGEPLVLRATGGKGGGGTTLTPRATALIAAFRAIEREHRRFIDAASAAVEGFEVNWKLIGRIGM

KTSARNQLFGKVLAVKHGAVNDEVVLALPGEHTITAVVTHESVQELGLAPGVDACALVKASWVVLAVEDG

SPLRLSARNQLQGVVETVTRGAVNSEVLLALDGGMTLAAIVTNDSVDALGLAQGVSAVAAFKASSVILAV

NG

>WP_004190735.1 MULTISPECIES: hypothetical protein [Burkholderia]

MKRTIRKSWAALVPLLVAGSVCAAAPLCQTQKLGAHTSKMCVEQTPFKHDYYTLWVDDSPIFMLPDDYVE

KVALTHTVPEDGAIEFPLSKQGTPTVTISGGCAPVSETQGKGADAVNLETGRVCSFNWGKEPVVKDLRFS

FE

>WP_004190638.1 MULTISPECIES: OprD family porin [Burkholderia]

MTQHRSMKRVARAVVLAWGLPMLAGVSIAGAARADDAAPAAPAQAPLAATLAQQATTPNALVNADATQAV

LPQPPMSSQAKSKGFIADSHFDVLLRNYADVLDAKGGPHRHAWVQGVMANFESGYTGGLIGFGVDASIYA

ALKLDGGAGGGNMVHVAKGGGGSNQLAWAFPGIYDVKARISNTVIKYGLQAVDNPFMEQHDNRALPPTFL

GATIVSNEFKNVMLEAGSFTKTNARGHTTLTNLTTQYGGTRVDRFTYVGGTWDYSQTGSASFYAGQADDV

WRQYYGSVKQSYGSPQTVKWTGFGNFYSTHDTGDARQGKIDNNAYSLSLAAQHGPHELLLGYQQVLGDQF

FDYLNETNGIFLVDSMDVDYNAPHEKSLQLRYTFYGKEAGLPGFKAMVWGVTGWGADGSASAALDPTRSS

IYWKDGAPVQGRHHEFGFIPSYTIQSGKLKDTKITFIAMWHVGSAHYSDSTNQEYRLVVNVPVKVF

>WP_004190160.1 MULTISPECIES: hypothetical protein [Burkholderia]

MMTERAIRKIGQWAAGVTCALAAVLVFAQPPHGGGPGFGGGRGGVPAMAGGWRGAPGYLQAAPHGGEARI

GVNGYRQRWGLRPTPSYGRYAAQSPYRPISAEARPAPHASGPGNVPMRAGSIRADVARYNEERGGRPAPA

PRQDDSQRLPFFAPFSRN

>WP_004189918.1 MULTISPECIES: N-acetylmuramoyl-L-alanine amidase [Burkholderia]

MSRKMLIKPFRSIESAATATHNWRRRQILRAGASTLVLGLALPRLAHASSVLGVRVWPARDYTRVTIESD

QPLQNTQQLLQGPDRLVVDLNGLELDQALRDLVSKIAPNDPQIQSVRVGQYQPHVVRMVFDLKGSVKPQV

FTLPPVGTYKYRLVFDLYPAVAPDPLSDLIAQTERKEQQLNDTLRAQQAQPPTAALNGPTAAPPAATDNS

DAFFQRFAQNNPPAAHSARPAPANPPVAQASPPAAPPRPAVKPPPVIARRNDDDSGDTDTYAFTAPKSGR

GTVRLLTVAIDPGHGGEDPGAIGGGGTYEKHIALDIAKKLRAKIDAAPNMRAMMTRDADFFVPLNVRVQK

ARRVGADLFVSIHADAFTTPSARGSSVFALSDHGASSAAARWLANKENSSDLIGGINIKTQDVAVNRALF

DMSTTAQIRDSLRYGNYVLREVGGINKLHKGSVEQAGFAVLKAPDIPSILVETAFISNPDEERRLNDDAY

RDQMADAIFRGIKRYFSANPPLAKSRMT

>WP_004189582.1 MULTISPECIES: hypothetical protein [Burkholderia]

MNRELLTHIRADQLDGSIFDASCHAVTVGPVTVEFRTSAEPPSSTAELYISRQLVGGCTVNAAHPSGKFG

GSVGKLKAEVDLTLDVPGGKLDYKLTVCAPIIGGTSKSGTLGL

>WP_004189224.1 MULTISPECIES: hypothetical protein [Burkholderia]

MDERPTRIPPPEKVMSPDPEPVAVEFLAELPEHVRAFFDEQHRLCTPK

>WP_004189093.1 MULTISPECIES: DUF502 domain-containing protein [Burkholderia]

MMKKTTLKSVFLTGLLVLVPLAITLWVLGLIIGTMDQTLLLLPASWQPERLFGFRLPGIGAVLTLAFIFV

VGLATQNFIGQKLVTWWNAVVRHIPVVGPIYTSVKQVSDTLLSSSGNAFRKALLIEYPRRGSYTIAFLTG

TPGGDVVNHLKEEHVSVYVPTTPNPTSGFFLMVPKSEVVELDMSVDAALKYIVSMGVVAPPAPVAAPARR

PVEPPM

>WP_004189072.1 MULTISPECIES: IclR family transcriptional regulator [Burkholderia]

MTMEGIYFTMVNPHGGPRREPPPPHSSRHAVNKSASPPSDAPDDDLADDHDTGEEKVRSGIQSIEVGFRL

LDVLTGEPRAMMLRDLAQRAAMSPAKAHRYLVSFQRLGLVSQDPVSGRYELGGFALQMGLARLARVDGVK

LARIALTALRDQLDQTVGIAVWGNQGPTIVHWMESSHPAKASLKLGDVMPLLGSATGLVFAAYLPRGKTA

AMIERELADTRRAAHYTGPRTRAEVDAVLADVRAHRAARVEGMLLPTINAFGMPVFDAVGELALAIIALG

HEGAFDIRWGGEVDTALRTCAQKLSYELGYSEGARDA

>WP_004189020.1 MULTISPECIES: response regulator [Burkholderia]

MNGTEESTTPIAPDAPQPHAADHARSAGSACAGADGGAPAAPVILIVDDEPSILSALKRLLRTARYQVVT

AESGAAALDVLAVGEVDLIISDMRMPCMTGAEFLARAQTLHPDTMRILLTGYSEIDAVVSAINEGGVYRY

LNKPWDDHDLLLTVRQALEQRRLRQETARLFALTQKQNEELVAFSTELEAQVHARTEEIRQTVMFLEDAQ

RDLKRNFMTMIQVGANMIELRCGVMGGESRRIGELAKRLALALDMSELQAQDLYFAGLLHGIGKLSLPDE

LLRKSIDRMSAEESRLYHQHPLRAQMVLTPVTQLNHVAHIIRHQYERFNGRGTPDGLAGDDIPLGSRILA

IARDYEGLQRGGVVNQRLQSEQAIALIKSQAGLRYDRQVVDRFVALVKDLATLGCNTPYARITGGQLLEG

MRLADDLRTSRGVLLMTKGSVVSAHQVALVRRYEAQESTPFAILIQTTTLAEAAASAGREC

>WP_004188960.1 MULTISPECIES: SCP2 sterol-binding domain-containing protein [Burkholderia]

MTLAAKPFAAAVNHLLARESWARDRLIPYAGKTARLEISPVTLVLLVQPDGYLAAVEAHDARRFDVSIAL

SSGADGGAFDAAAAFLQGGQAAVMKHVKIDGDAEFATQIAKLAEHLRWEPEEDLSSVVGDAAAHRIATVV

RSAGARARRTGRNVLDSIAEYWLDENPQVVRKSALADFDAGLARARDTLARVEKRIERLEQKIDVRAGGS

SRRAQ

>WP_004188306.1 MULTISPECIES: GNAT family N-acetyltransferase [Burkholderia]

MLQMRPMTQTEFREYRERAARGYARDLIESGQSAPDEADARALACIDTLLPDGLLTDDQVLLTLSESADG

SVLGHLWYGVVAEGPHRSLFIYDLEIEPAFRRQGWATRVLQALEDDARELRVSEIGLSVFNHNAAALALY

RELGFAAVTTTFVKSIESP

>WP_004188208.1 MULTISPECIES: hypothetical protein [Burkholderia]

MDMHWIASGAVLLVMAVLSAYRDALKNEPINRSGLGRGGAPYSEEFE

>WP_004187391.1 MULTISPECIES: hypothetical protein [Burkholderia]

MSLIFALLQKIEDLFTSPHLPLDADYSYKARHAEVERVRKSHVSQVGLIRR

>WP_004186871.1 MULTISPECIES: DsbC family protein [Burkholderia]

MKKTIRIAALALAAATATLGCTAQADQSTDKLKAALQSRLGADAPIKSVTKSPIAGLYEVNLGSQIVYSD

ASGDYVLLGELVNTKTHKNLTAERLAEINKIDFASLPLSNAIKVVKGNGARKIAVFSDPNCPYCKKLETT

LQSVDNVTVYTFLYPVLSPDSTVKSKSIWCASDRVKSWQSWMLEHRAPTSAANCDTTALDKNLALGRGMN

VTGTPTVFLADGTRLPGAVSADELNQALAGVK

>WP_004186870.1 MULTISPECIES: MltA domain-containing protein [Burkholderia]

MSCRLGCFFLPEAVQTTMKTSRASASNDSSAKMSIGYWSSRSDMAVSINNGHCMGFSRRLAGWAAAVAAA

ALLAACVGSPVRQGARPAGAAIVPGQIAAARLTPVAWQQVPGWQDDSLIGATIALRQNCARLARQANWQR

ACAAAMRLDDLDVGSARTFFETYFTPFQFANNDGTLDGLVTGYYEPLLHGSRVRRGPYQYALYRWPAGYR

AGASMPARAQLMRSGALSGNELVWVDDPIEAFFLQVQGSGRVVLDDGTVMRVGYGGTNNQPYRSIGKWLL

DHGELGAGQATMQGIKAWARANPSRVDALLDTNPRFVFFREMPSQEDVPHGGADGPVGALGVPLTPERSI

AVDPSSIPLGTPVFLQTTRPMTNAPLNRLVFAQDVGTAIKGGVRADYFWGLGDDAGDQAGRMKQNGRMWL

LFPNS

>WP_004186572.1 MULTISPECIES: glutathione S-transferase [pseudomallei group]

MMKETSMLRLCGFPVSNYYNKVKFVLLEHDIPFEASIVSVPIKDPAFLADTPLGKVPYLMTEHGSLCESQ

AIIEYLAAIHPDKPIFPADPFAAAKAREITAVLELYLEWNVRELFPEAFFGGKVSDGTKAHVEKRMPRAL

DGFKRLAKFSPYVLGDTFCIADIAAFIHLPVVALATKAIYGRDFVVDAGIDWKAYVKRIEEARPAAKRVS

DERKAFVAGLAKPG

>WP_004186104.1 MULTISPECIES: DNA translocase FtsK [Burkholderia]

MAKAPYTAQAQALPHRMSRLLTEIRWILQVALLAFLLMALLSYSRRDPSWTHAAQVDHIANWAGRVGAWT

ADIMLLLFGLSAYWLIVLLGRRVAANYRRITRHDALPGEPEKPAGWLAEGFAFVLVLLASDGIEALRMWS

LKVQLPRAPGGVVGETVARGVAHALGFTGGTLALLIALAIGLSLYFRFSWLSVAERVGDAIINAFTLAKL

RREAERDRKLGEAAAVKREGKVEEERVRIEEHEPVTIVPPVVTPAKSERAEKERQQPLFTDLPGDSTLPA

IALLDPAPTSQETISADTLEFTSRLIEKKLKDFGVEVSVVAAYPGPVVTRYEIEPATGVKGSQIVNLSKD

LARSLSLVSIRVVETIPGKNFMALELPNQRRQTVRLSEILGSEVYADAPSMLTLGLGKDIGGKPVCADLA

KMPHLLVAGTTGSGKSVGINAMILSLLYKASAEQVRLILIDPKMLEMSVYEGIPHLLCPVVTDMRQAGHA

LNWTVAEMERRYKLMSKLGVRNLAGYNNKIEDAKKREEKIPNPFSLTPDDPEPLGRLPNIVVVIDELADL

MMVVGKKVEELIARIAQKARAAGIHLILATQRPSVDVITGLIKANVPTRIAFQVSSKIDSRTILDQMGAE

SLLGQGDMLYLPPGSGLPVRVHGAFVSDDEVHRVVEKLKEHGEPNYIEGLLEGGTIDGDEGSAAGTGEAN

GESDPLYDQAVEIVIKNRRASISLVQRHLRIGYNRAARLLEQMEQSGLVSAMSSSGNREILTPARDAE

>WP_004185992.1 MULTISPECIES: hypothetical protein [Burkholderia]

MGSTLNPDDEPREPDERSPSSGDNRTLGPSDSSDSGSDVAGARRRPFDVDTELDNHALETGDAELDSDTD

RSGTGERASADGDSTFDDSADIEPDRTERVPRTKRGDD

>WP_004185577.1 MULTISPECIES: carbohydrate ABC transporter permease [Burkholderia]

MQPKMTLSRAVIYAALILFALYFLFPIYVMLSTSFKDLDQLRTGNLLTPPSSWTVAPWAKAWGEACTGVR

CEGMKPFFFNSIKMVIPAVLISSLIGAFNGYVLTHWRFRGADALFTMLLVGCFIPFQVILLPMARLQGYF

GMANTIPGLVLVHVVYGIAFTTMFFRNFYVSVPAELVKAARIDGAGFFTIFTKILLPVSLPIFMVCLIWQ

FTQIWNDFLFGIVFSGVDSMPITVALNNLVNTSTGVKEYNVDMAGAIIAALPTLLVYVIAGRYFVRGLTA

GAVKG

>WP_004184782.1 MULTISPECIES: chemotaxis protein CheC [pseudomallei group]

MASMNDIALTEEHRDALQEISNIGMGRAGAALAKLLGAFVTLSVPDIKLVSARELLDELQRCERRADMPR

PVRQSFQSDICGEALVLFGSDGRRELKELMGYDDATDDIEDEALSDIASLLVGACVHSVFEQLDRRLTFL

RPTFVPPGSLASALGDERLGRWDVALLLEVHFTLEHGGFVAKLVMLLPDAAIHKMKAALEQFLDAL

>WP_180945708.1 hypothetical protein [Burkholderia pseudomallei]

MKRRTMRRACKRAVSAVSTGRGAVLVLVLMPVLVLVPVLVLVPVPVLVLVLVLTLSPSRGVSPAVARVGA

SSPSVHTRRVPRKSCRACAAASS

>WP_179165088.1 glycoside hydrolase family 92 protein [Burkholderia pseudomallei]

MAVDASSTAPLACEAQEPETIMNRFVRLGAAMAVACALAACGGDDGGPAVAASSFAAAGANGATAGVADT

AQADGAPRTGSLTQYVDPLIGTLASNSPNPVPAGQAGSVVPAAGLPSGMIQWAPDTNTTPAPADSKEPGS

PAGYYYDLNAIQGFSVTHMSGAGCAGNNGEFPVMPTTDATKLAPTFSHANETAKPGYYSVLLDSQIKVEL

TATLRTGFGRFAYPAGKPALLVIDATRTNTKTSTSGAITRVSANAISGSTVGGGFCGNSVPVPVYFYATF

DRPFAPASSISRGVAKLAFDSGATVRMKIGISYVSVDNAKANLDAENRTWDFDGVRALADAAWNDRLGAI

RVSSSDADALKKFYTAFYHALWAPSVFSDVNGQYIGFDKQVHTVAKGQAAQYSSFSGWDVYRSLIQLKAV

LFPRETSDMIQSLVNDADQCGAIPHWVNDNVEDGVMPGDAGSLMVSSAYAFGAREFDARGALAHMIRMAN

IPGTACAGVTTNGGRASYLQTGYITSGEWGIASSTLEYTSSDFAISRFALALGDTATQKMLLGRSAYWQN

LLNASLNPPLIAARQSNGAWIAETPGSTDNYVEGNAEQYTWMVPYDPAGLFAQLGGNQAVVPRLDKFFTV

LNAGMSLPNFYMGNEPTFEVPWLYNWAGSPSGTQRVVRQIMQTAFSTKPDGLPGNDDLGAVSGWYVWAAL

GLYPQVPGVAGFAIGSPQFDAIDVRLGSGRMLKIRAPGAPASGYVQSVAVNGRAQASPWIALDALDGGAV

MHFKMGGAPSQWGAGEAPPSFGVPVARDVADSFNNRGISADGATNADGQGADFDGSLFSYSANALAQAGV

RPGAPFTYGGASFVLGGASSLDNAVAVGQTVMLPPGSAGTSVVVLGASNNGPSAGVARVSFADGTSAQVT

LSFDDWTLNGGSTGATSAIAVTSAYRNAGNGQKDNVKTYIFAQKIPVPAGKVVTSVTLPRQVSAGKMHVF

GIGVAA

>WP_179165082.1 sigma 54-interacting transcriptional regulator [Burkholderia pseudomallei]

MFPVRRAMRAACIVARTSVPRTIAHASPRRRRGSLAERLPRRPVASDASVASVASVASVASVASVASVAS

IASVASVASVASVASVASVASVASVASVASVASVASVASVASVASVASPPSRPTRIGRTLRASAPTSNSA

RRARPDTFRTTSQLRCIVPDFRPPSDCAPPGHAAQRSASPADAAARQLVYLSRTPDTTLVAHLRARRWNV

HVARSANEAARRVKPNQPQAGIADLDGFAPRELPTLEAVLRQQQVGWIALAGDTRINDPDVRRLIRQYCF

DYMQGLPPHETIDYLVGHAYGMVALCDLDVTAGAAATGDEMVGACDAMQQLFRTIRKVAATDATVFISGE

SGTGKELTALAIHERSERRKAPFVAINCGAIPNHLLQSELFGYERGAFTGASQRKVGRVEAADGGTLFLD

EIGDMPLESQASMLRFLQEGKIERLGGHESIPVDVRIISATHVDLDAAMREGRFRDDLYHRLCVLKLDEP

PLRARGKDIEILAHHILHQFRSDGARRIHGFTSCAIEAMYNYHWPGNVRELINRIRRAIVMSDSRQLSAA

DLDLAPFAARQATTLAEARERAERRTIEASLLRHRNRLTEAAAELGVSRATLYRLMVSHGLRELSWGAQR

AGASDVDDEAGPA

>WP_178131487.1 3-keto-5-aminohexanoate cleavage protein [Burkholderia pseudomallei]

MTRRPSPPSIERSPNVASPRKVIITCAPTGAIHTPSMSPHLPVTPREIEEAAIAAAEAGAAILHLHARDP

ADGRPTQDPAVFAEFLPRIKARTDAVINITTGGSPHMTVAERLRPAHHFQPEVASLNMGSMNFGLYPMLE

RFREFRHPWEREHLEKSRDLVFKNTFADIETILASCGANGTRFEFECYDISHLYNLAHFVERGLAKPPFF

VQSVFGLLGGIGAHPEDLAHMRRTADRLFGADYVWSILGAGKHQIPLATIGAAQGANVRVGLEDSLWIAP

GRLAESSAAQVRKIRQVLEGLSLEIATPADARAMLALKGGGAVNF

>WP_004547468.1 MULTISPECIES: lytic murein transglycosylase B [pseudomallei group]

MIFHQPAAPASLAFRLRFPLAALSLAATLFASAAVAQTQPAAAVVAQEPAQSQSQSQSQSQLQLQPQPQP

QPQPQPSVQQGQTFEEEIIPQRYANNAKIDAFIADMVARHDFDANALHALFARVSYSATAAKLVMPAPSP

AVKNWRVYQSRFLDAVRVNAGVKFWRANQGTLQRASTEFGVPPEVIVGIIGVETIYGRYMGNFRTLDALT

TLAFDYPNTPNRDARQATFRKNLEDFLVWTRDSQLDPTGVLGSYTGAVGIPQFLPSSIRDYAVDYDGNGH

IDLRASQADAIGSVANYLKQHGWETGRPVVWNIAPDTGSQGVAQAAADGRPEPHWVLSQLLRAGLVLDEP

SVNIASEASTPVTVVDLPTPGRATEYKLGLQNFYVLTRYNRSFFYALAVYQLGERVKAQMEASGALTPSP

ADAATGSPAAQPPSE

>WP_004522643.1 MULTISPECIES: pyridoxamine 5'-phosphate oxidase [Burkholderia]

MTTLADLRTNYSRASLDAADVNPNPFVQFDVWFKEALDAQLPEPNTMTLATVDESGRPSARIVLIKGADE

RGFVFFTNYESRKGRELAHNPNAALLFYWIELERQVRVEGRIEKTSEEESDRYFASRPLGSRIGAWASEQ

SAVIESRALLEAREKEIGARFGENPPRPPHWGGYRLVPSSIEFWQGRPSRLHDRLLYTRDAASASGWKIA

RLAP

>WP_179104127.1 UDP-N-acetylmuramate:L-alanyl-gamma-D-glutamyl-meso-diaminopimelate ligase [Burkholderia pseudomallei]

MRGGASAALSMFSGNRRDVRPAADFNYAHAARSSMHIHILGICGTFMGGLAVLARAAGHTVTGCDAGVYP

PMSTQLEAQGIQLIEGYGAEQIDLKPDLFVIGNVVSRGNPLMEAILDRGLPYVSGPQWLGEHVLAGKWVL

AVAGTHGKTTTSSMLAWVLEDAGLNPGFLIGGVPLNFGVSARLTDSSFFVIEADEYDTAFFDKRSKFVHY

RPRTAILNNLEFDHADIFPDLAAIETQFHHLVRTVPGVGRLVTNGRDDALERVLSRGCWSDVERFGVDGG

WQALPVENGVPVDGRFAVYWRSERVGAVDWQVQGEHNRMNALAAIAAARHVGVPPAQAAAALAAFRNVKR

RMEVRGSVDGVTVYDDFAHHPTAIETTIAGLRTRIGRENTRILAVLEPRSNTMKLGVMKAQLPASLADAD

LVFGYGAPSGRDALGWSLPDALAPLGGKARAFDDLHALVKAVTASARPGDHVLVMSNGGFGGVHQKLLDA

LSARGDAAPARSGA

>WP_179102759.1 YadA-like family protein, partial [Burkholderia pseudomallei]

VASLSSSTSTGLSSANSAVASLSTSTSTGLSSANSNIGSLSTGLSTANSTVASLSSSTSTGLSSANSAVA

SLSTSASTGLSSANSNIDSLSTGLSTTNSTVASLSTSTVAGLNSLSTGLSTTNSNVASLSSSTSTGLSSA

NSAVVSLSTSASTGLSSANSNIGSLSTGLSTTNSTVASLSTSTVAGLNSLSTGLSTTNSNVASLSSSTST

GLSSANSAVASLSTSTSTGLSSANSNIGSLSTGLSTTNSTVASLSTSTVAGLNSLSTGLSTTNSNVASLS

SSTSTGLSSANSAVASLSTSASTGLSSANSNIGSLSTGLSTTNSTVASLSSSTSTGIGSLSTGVANSVQY

DSPAHTSITLGGASATSPVKITNLAAGANPSDAVNYEQLTSLSTSASTGLSSANSAITSLSTSTSTGIGS

LSTGLSTTNSNVASLSTSASTGLSSANSAITSLSTSTSTGIGSLSTGLSTTNSNVASLSTSASTGLSSAN

SAITSLSTSTSTGIGSLSTGLSTTNSNVASLSTSASTGLSSANSAITSLSTSTSTGIGSLSTGLSTTNSN

VASLSTSASTGLSSANSAITSLSTSTSTGIGSLSTGLSTTNSNLSSLSTSSSTGLSTANSNISSLSTGLN

SLSTAVNGGGTKYFHANSTQPDSQALGADSVAVGPAAIAAGASGIAIGNAANAAANGAVAIGQAAVAKGG

LAVSIGVSNTASGDGAVAIGDPNVATGTGAVALGADNSANGQGAVALGNANIATGTGSLAFGNTSTAAAA

GAVALGAGAIANNANDVALGSASVTAAANPVASALIAGQAYSLAGGAPASVVSVGAPGAERQITNVAAGR

ISATSTDAVNGSQMNAMTQALESLSTSTASALSTAQSGLGSLSTGLSSTQSSVSSLSTGLSTTSGNVASL

SSGLGTMQSGIASLSTGLSTTNSSLASLSTAVSGGGVRTSSLGDTSAGNGANASGGNGTAVGGAASASGT

DATALGQASNASGNHSTALGQASSASGSGSTAVGQGAGAPGDGASAFGQGALASGTDSTALGAHSTAAAP

NSAAIGANSVASAPNSVSFGSRGHERRLTNVAPGIDGTDAANMNQLWGVQSSVDQAARRAYSGVAAATAL

TMIPEVDPGKTIAVGIGAGSYQGYSASAIGVSVRFSDNLKAKLGVGISAQGSTYGAGVSYQW

>WP_179100823.1 M15 family metallopeptidase [Burkholderia pseudomallei]

MIAVVLVAYFAVAVLAAALLLLPAVRTTVFDSVAQFHSRISRRASDRAARAREHLAQSARMSRSTLSGVQ

NLLVRRRLLIATTTGILATPPLIAIALRGRQLFQYDDTLRVPDEKIAALLKGEQLVPPLPLPPEVFATRE

VEQVRPALKDASRDWNLLDTDFRTRLLLVYKIMREQHGYEMALLEGYRSPERQNRLAQMGSNVTNAAAFQ

SYHQYGLAADNAFLRDGKLVISEKDPWAMRGYQLYGQVAEEVGLTWGGRWKMMDLGHVEYHKPGFKLGRS

SAR

>WP_179100475.1 alpha,alpha-trehalase TreA [Burkholderia pseudomallei]

MPQPTVICAKCGKNRLPERRFSCESAAAAHDRQTRRHASGPRDQETVLPFISKTGGGDMVTPRHRPLHVE

NPFYRRLLNAPAWVALVAAAGIGCTSATLARADSSPAHASTAAAVASAASAPGAASIPPPPSQLYGDLFV

AVQTAQIFADQKTFVDSTPNADPATIVQLYQQQKGQPGFSLKAFVAQYFTPPSDESVTPPPNQTLREHID

WLWPKLTRTTTTAPPYSSLIALPKPYVVPGGRFREGYYWDTYFTMLGLQEAGREDLVDNMLDNFAYLIDT

VGHVPNGNRSYYVSRSQPPFFAYMVTLAAKAEGNRVYQKYLPALRKEYAYWMQGERTTPRGQATRNVVAM

PDGSVLNRYWDASDTPRDESYLEDVKTAQQASGRPAAEVWRDLRAAAESGWDFSSRWFGDNRTLATIRTT

AIVPVDLNSLMFNLETTIVKGCAVTRDFACVAEFAGRAGKRAVAINRYLWNRNGYYGDYDWKLGKPRDNL

SAAALYPLFAGVAWPERAKQTAKNVQKALLKPGGLATTTYDTAQQWDAPNGWAPLHWIALVGLRHYGEKS

LADDIGTRFLADVKGVYAAQGKLVEKYIVEGVGTGGGGGGEYPLQDGFGWTNGVTLKLLDLYGG

>WP_179100172.1 YadA-like family protein, partial [Burkholderia pseudomallei]

GESSTATGQGSQATGDNSTATGQDANASGESSTAMGQGSQATGSNSTATGQDAIASGESSTATGQGSQAT

GDNSTATGQDAIASGESSTAMGQGSQATGSNSTATGQDATASGESSTAMGQGSQATGSNSTATGQDATAS

GESSTATGQGSQATGSNSTATGQDATASGTSSTATGQGSQATGSNSTATGQDANASGESSTATGQGSQAT

GSSSTATGQDATASGGSSTATGQGSQATGSNSTATGQDATASGGSSTAMGQGSQATGDNSTATGQNAAAS

GESSTATGQGSQAAGSNSTANGAGAAATGHQSTALGAGAQAAGSQGVASGWNANASGTQGVAIGGNASAQ

GNQSIALGANASATGDHSIALGAGSQATGSNSVALGQGSIADRDNSVSVGSDGGERNVTNVANGWHDTDA

VNFRQLREVARYAYSGIAAATALAMIPDVDAGKTFSIGVGTGGYLGYQAVAVGASARLGQNLKVRVGAGI

SAASTTWGAGASYSW

>WP_179098602.1 hypothetical protein [Burkholderia pseudomallei]

MTSMAMKKTDLEKNKALKLTHAMKQTTSARFGKGAEDAAADRRERRRLEQAQGLVPFACKLNIELVARLN

ERAAAHPDGMTGLLTELLQRGLDETAK

>WP_179095309.1 phytochelatin synthase family protein, partial [Burkholderia pseudomallei]

AASSRASRYARARVARAALRSSNDPGAMTVNTRLAGAVLAALALIGPAAAQPLPLPAGLIDLGSLAGEQM

LAESGARSAYASLGSHFVTQKTQSYCGVASLVMVLNALRVPAPAAAQYPPFHYFTQDNVLGDATERIRPR

ALIERHGMTLDQLGALANALGASGDVRHASDVSVDVFRADAIAHLGRPGRYVLVNYLRSRLGQQTGGHIS

PLGAYDAAADRFLILDVSRYKYPPVWVTTADLYAAMNTPDADSGGRSRGYVLIDGVVAGADS

>WP_076899887.1 ABC transporter substrate-binding protein [Burkholderia pseudomallei]

MCRRRRAVARARIASRGVDSVDHVGSGGSGGSIRGIRGIRGIRGIRGIRGIRGIRDIRGIRGIRGIRGIR

GIRGIRGIRGIRGVRGVRGIRSVSGIRSISGIRSIGGTEAMTDRRTFVGGAAAFAAAWAAAPFGARAGIA

VDLDPRQAGRVRAERDPAVVDAARRYRWVDDRAFTVAIAPHAPPVATFATDARTVVGADPDYAQLVADAL

GRRLALVPVAWADWPLGLSAGKYDAVISNVGVTEQRKRKFDFTTYRLGLHGFYVRAASPIARIAAPRDVA

GLRIITASGTSQERILLEWDRRNVAQRLKPVDVLYFDDDAASRVALLSGRADAELNPNATLAYQAARDGR

IRCVGNVNAGWPLKADVAIATRKGSGLADALTLATNGLIRNGKYRQALARWGLLSEALDRSETNPAGLPS

F

>WP_038773682.1 GNAT family N-acetyltransferase [Burkholderia pseudomallei]

MATADTRMPPTSLTIECAAARAGALVRRVMRAFSPAGAPFIVSGILFVSDNAYTNAFGQPIGAPVPGWQP

RPAPPRTPAQGRFCRIEPVDPGRHADDLHAAYATAADGRDWTYMSAGPFADAESYRAYLRKAAETDDPLH

HAIVDLATGKAVGTFALMRADRANGAIEVGFVAYSPLLQKTCAGTEALFLLMRRVFDELGYRRFEWKCDS

LNAPSRAAALRYGFTYEGLFRQAVVYKGRSRDTAWYSIVDGEWPALRSAFEQWLAAGNFDAHGRQRQSLS

TLIAAARRR

>WP_004186734.1 bifunctional riboflavin kinase/FAD synthetase [Burkholderia pseudomallei]

MRHPAALSPTLSPAIVKVFRGLPNAESRAPCALTIGNFDGVHRGHQALLARVRAAADARGLPVCVMTFEP

HPREFFNPASAPPRIAMLRDKLEALREHGVDRVVVEHFNHTFASQSPEAFVERTLVHGLHTRWIMVGDDF

CYGARRAGDFASLKAAGERFGFEVEQMETLADADGARISSSGVRAALAAGDLDAAARALGHGYAISGHVA

HGLKLGRDLGFPTLNLPIAHKRPALSGIFVVRVHGLAPEPLPGVASLGVRPTVDDSGRVLLEVHLLDWHG

DAYGKLVRVEFLKKLRDEAKFVDLETLSRAIAQDVVDTRAYFAEHGRAPGSRATGFATSATDRIS

>WP_156606184.1 phospholipase D family protein [Burkholderia pseudomallei]

MVHRPARGSVDPRRRRHLECLRAVHRPAAPSIRRFVPTDARRSPNVPDRPALRGLAPRHPPRIVKAAARA

LVLCAALALSGCATHPPATTLERTVSHALPPDASTPLADALAVQARAHPGESGFVVLPRGDEALQMRIAV

ARAATKTLDIQYYIAAEDTTGKLLLGAALYAADRGVRVRMLVDALNFKDIDKLMAALDAHANLEVRVFNP

FGAPRLGMFARTANVFTRIDNFTRRMHNKAMISDNQIAIVGGRNLGDEYFNASPTLQFRDLDVLAAGPVT

RAVSASFDAYWSSALTYPLPALNRRRYDAKDLDAARDALRAHWRANATPYNAKPLNATPLAAQIARNELG

LVWASAEFTADSPEKIAAPDDSYKSPPMQRLFALTRDAQREFLVLSPYFVPHDAGVNALGRLTARGVRVA

ILTNSLAATDAIAVQAGYAPYRVPMLERGVELYEYKPDPGRSRIGMLGSRSRASLHAKAYVIDRKILVIG

SMNLDPRSAHLNTELALVIHSPRLANEIANLFDEVTKPTISYRVTLAPDTPGAAQTTGAGAPAWPLVWTE

IADGQVRTYSVDPNAGFYRNLLTGLCLLLPIDDQL

>WP_156605851.1 L-arabinose ABC transporter ATP-binding protein AraG [Burkholderia pseudomallei]

MAGNGGDVAAALRFDNIGKVFPGVRALDGISFDVQAGQVHGLMGENGAGKSTLLKILGGEYQPDSGSVLV

DGRAMRFPSAAASIAAGVAVIHQELQYVPDLTVAENLLLGRLPSALGWVRKRDAQRFVRERLAAMGVDLD

AQAKLRRLSIAQRQMVEICKALLRNARVIALDEPTSSLSHRETEVLFKLVDDLRRDGRALIYISHRMDEI

YRLCDACTIFRDGRQVASHASLANVPRETLVRQMVGREISDIYHYAPRALGDVRLSARALEGDALRAGAS

FDVRAGEIVGFFGLVGAGRSELMRVIYGAQRRTGGALTLDGEPLDIRSTRDAIRRGIVLCPEDRKEEGIV

AHASVAENINISCRRHGLRAGLFLDRKREAETADRFIKLLKIKTPNRRQKIRFLSGGNQQKAILARWLAE

PDLKVVILDEPTRGIDVGAKHEIYGVIYELAKRGCAIVMVSSELPEVLGVSDRIVVMREGRIAGELARGE

ANEEAVLNLALPQGATAHAA

>WP_024428867.1 TIGR02099 family protein [Burkholderia pseudomallei]

MSDRQDSAATTEAGPPQHHHPVLRRVFKGVLAIAIAAYFIAAAAFLGLRYLLLPRIDEYRPRIEAFVSQK

LHAELRIGRLAPHWSGMQPGVDISRLTIRGRDGRVALSVPHATAALSWRSLARFAPTLSSLVVDDPDLLT

ERRADGSLFVAGVAVPTTKTGADDTFSAWLLKQEAIVLRGGTLRWRDAQHDAPELALTGIRIAVLNDGIV

HRIALQAPANGTLFHGPLDFRARFVHQALAPIGKPSNWTGDAYLSTGPVDLPTLARYANIRVTAYAGRID

NAIWAHFGDGHLYRAGGELRGYDVALRVRPTQPRLDIPVARFGWDVAIDPKRDYTLHLSRLHAELGQPPL

ADGTPLSRALALHTLTARYRVPNVDEGQLLSVSGDRVDLGILAEFIRGLPLPARLRNELVRFDPRGLVAN

YAIEVERAKPASPEFVDEERRSGTAPIIRYRFQGDLQGISFEAQEPPPGLSPHGHPRAGIPGVENLWGHV

DADEKGGAARFDTVDAAVTVPGVFDEPRLAFDKLRGRASWTITPAPGERHARVDVAVPEFRVENADAAIA

VAGSYANPGHGRGSLDLKADFERAAVARITRYLPTSLSDHLRLYLGHALQAGQVTKGATIVAKGPLETFP

YEHDPKAGVFHIVAPFAGGRFEPTPQPPRTLANGTPNVWPALDGIDGVFELEQNRLRFDIDRARYKGVAL

AKVTGRIDDLGNPTHSPLVIEGRAHGPLADLLDYANHSAIAGMTGHIGNLVRAQGPATLALKLTIPQHVA

HPHVGVDGALGFAGNALEADGVPPVTRLRGNVRFTQYTASVDRLTARFLGGDVRARGALAENGRYAFDID

GRLSLDAARGLNLRGAAAAALERVVGDAPYRLAVRGAKGGLPDITANSDLTGVALEFPAPFAKPAGTPMP

FSFVLAPEPQASGKPLERADLALGPLAATYLLDVERGRPVRAVRGALGMNRMPDLPQEGVSAAIDVHELD

ADAWQAFAQGFGKSPAAREQPAASPVDLASFAPKRFALHFGTLKLLKRNWENVIVGASHIDDIWQANIAS

NQVSGYLSWAPGGGPTGAGVLSARLAKLVIPESAEHDLVGRAIDLPTPTHHAMPAIDLVVDQVVARGHDI

GRLEVDARNVDENGIPVWQLDKLELANPAAKLTATANWRTSRRALAHGADEEDAPRRTVFDFTLAIDDAG

ALLERVGLPRTVENGRGTLSGKVAWRGGPTAIDYPTLNGRLSLDLAHGQILKVDPGAAKLLGVLSLQGLA

RFLTLDFRDVIGKGLPFETITGTGRIDNGIARTQDFEMKTSPAKVTVTGSVDLAHETQDLNAHVAPKVSA

SAAAVGAAIINPFLGLGVLAANLALSQTLAHAFAMDYTITGSWAHPHIERVSGDRGKMGFAPAAVEH

>WP_011325029.1 hypothetical protein [Burkholderia pseudomallei]

MEAIVIEQVILGVFLVLPLLIVAVLYSDELWQEHRLQHPRDEHTPHIDWRHPWRILRRGH

>WP_004521780.1 SCO family protein [Burkholderia pseudomallei]

MNPPSLIRRRLVLAGAALVSLPACAAESREIAHRPWGAIEPAEAAPDIEVSLADGTRGRLVDLLRGKTSA

IQFVFTGCSATCSLQGAIFQSLQAQLARHPIGGAQLLSISIDPANDTAVAMTAWLRRFGAQPGWQAAIPA

SGDLARLARLYRDERNPADSHIDQAFISSRSARFVWKTDHLPTPESVHDALRYYASH

>WP_004528234.1 MULTISPECIES: lipid II flippase Amj family protein [pseudomallei group]

MDTQLWIICGLTFVIHVIATLAYAVRIAGVRTRRIAVSFSLFGIIALVSRTANSFQGPFIAKRVELDISR

HLGEGLLADFRLFLLSATVASVVGALLIPTFQRYFSRAVHHFQANRSVSRLLLRVFSRDGVGYIRGGARL

PSPRNVTQLATGTGVSWQVIVLNVVAMAIWTVGVFASLYAGYLKPELRVTCANLSSIINGFATVAMAVII

DPQVSVMTDDVIEGRLCENHFRRAITTLAGARVLGTLCAQAALVPAALVIVRVAELI

>WP_176370880.1 EamA family transporter, partial [Burkholderia pseudomallei]

MNLKNALLLIVLAALWGASFLFIRVGVAEFGVAPLMALRVGIGALFLVALMLTRYAPRELIALLRQHAWP

LFVVGLLNSAAPFCLFAFAELTLSAGVTSVINATTPLWGALVAYLWLKDSLSLPRALGLVIGFAGVLTLV

WDQVFSPHGANPASPATAALAAAAALGATLLYGIAANFTKRKLTGVDPLVSATGSMVGATILLLPFALAT

WPAAPVSAHAWGSVLALGIACTGVAYFIFFHLIAHIGPARAITVTFVIPVFGILWGALFLGERVSFAMLE

GCVIVLLGTALATGAIRRIPGVR

>WP_024428445.1 ABC transporter substrate-binding protein [Burkholderia pseudomallei]

MTVRRSLLPRSPLAAVRRACAAMLIPVASLALSLGASAAHAQDTAICYNCPPEWADWAAQIAAIKQRTGI

RVPFDNKNSGQAIAQLIAEQKSPVADVVYLGVSSAFQAKDKGVVAPYKPAHWSDIPANLKDPQGYWFAIH

SGTLGFFVNKDALDGKPVPRTWADLLKPEYKGMVGYLDPSSAFVGYAGAVAVNQALGGSFDDFRPALDWF

RKLKANQPIVPKQTAYARVLSGEIPILLDYDFDAYRAKYKDHANVEFVIPKEGTISVPYVMSLVKGALHE

ANGRKVLDFVLSDEGQKLWANAYLRPVRAQALGADVAAQFLPASEYARAKSVDFGKLAAGQQAFGKQYLQ

AMQ

>WP_011204170.1 MULTISPECIES: LEA type 2 family protein [pseudomallei group]

MAVPAPRFRGSSSMHAWYRFRPFVGLWFAALAVLLALGGCAALTGRDPVRVSVVGIEPLVGQGLEMRFDV

KLRLQNPNDAPIDYDGVALDLELNGRPFASGVSDVRGTVPRFGEQVLSVPVTVSAFAAARQAFGLADATV

SGKLPYVLRGKLAGGMFGSVRFTDSGTLSLPASGGYGGGY

>WP_004192119.1 MULTISPECIES: MEKHLA domain-containing protein [Burkholderia]

MSLSTDVAFFRLLADSYHRLVGKSLVPEGMSAAEGAAWLYEAAPFGMLAHDASADPVFVYGNRRAQAIFE

YDWDELTTLPSRLSAEPVERRERRSFLDRVARDGFVSDYRGVRVTKSGKRFWIEHATVWQLTDSAGHYRG

QAAMIPEVRPIGESA

>WP_024428781.1 PAAR/RHS domain-containing protein [Burkholderia pseudomallei]

MGEVQSSAPSDSQQKLAALAQRGSNADAVQTVSNMGLAINAAQVTAAGTSAWAAGTFQCFAGRVIAPLGG

AMLGGALAEALGADRPVTWVLDKMGLPAVAKPGKAPARVGHKIVHENAFIGALTGLLAGIAVGVAIAAAA

AAIVATGGAAAVAIAAAGPFVVGFVSGAVGGFVGAAVAKGIGHTGSVTGAIAHGSPNVSFEGAPVARVTD

PVTCSKDPGMPPPQIAQGSLTVSVNGLPLARIGHKITCSAVIQEGCTTISADETTGTFGKIDANVSLLEQ

LVLTATDVIMMRSATKEGGLLDGVLRELLGEPIDMATGDYADYRTDFTWPHVLPLTLSRAYAGRQPVEGL

LGDRWISNWSQRLRYRRPADGPATVTFFDADGQQLVYPVPHEPFNAINFWAPHYALHGSRARAVVFDERS

QQSLIFEPAHAEDDVARLTRIEDRNGNTIDFEYNALGRLCTVRHSGGMTLWVTCDSRGLLQSVSEQPGGE

GELVRYRFDGKRLTDVHSRFQGEFHFGYTDEGWLNHWRDSGATQVALRYDERGRVIATRTNTALYDDRFE

YDDEARLTTYIDALGHRHQRWFDAQNRLIRSRDPLGRVMCASYDENGWLASRTDPLGRVSTYRHDCRGRP

LQVTDAFGRVSRYGWNGAGQLVEQQDHNGKVQWHYSAEGNLVALRARSGETRFRYDARGLLVGRTDPDGA

VHAWRYDGAGRPERWTDPLGRHTYLEHDRYGRLIARIDAAGHRTTYGYERGPSNPRELLASITYPDGAIA

RFQYDSEGLLTEAVNPLGQRTRYAWGAFDLLASVTDPGQATTRYHRDGAARLIGVTNAAGQHWKFERDPA

GQLIAQTDWSGRRTRYVLNPMGQVTEKHLPDGVAIRYEYDSQDRLVALTGPRRRHVFAWTASGLLTRAEV

WTRNDEAGEWRRDDRLDLEYDDACRLVEESQHGRAVGYEYDPMGRTRSMATPSGRTLWQYDAAGQLGSME

SNGHRFHFDYDGLGLETLRRYTPTQAHVARHPQWVEPYSEGYAQQQDHDGRWRLTKQACATWAELRERGA

ARTRHYEWDAAGRCVGMHEARRGLPIAQDRWRYDARGQVVDAHYERTETRSGRERYEYDALGNVSTRQID

AGEAMTHVYHGDQLVSAGPSRYEYDARGRMIARTEGRDGFRPRTWRYQWDDFDQLEQVLTPEGERWRYRY

DAFGRRISKACLSTPKAGRPARIDYLWCGSRLIEAWRAYGERDGTQYEIQRWHYRPGTHSVLAQERLKYD

DKPDLQNSEWFALACDPNGAPHTLYSSDGRIMWSARRELWGRVADDPDRDTVRHAVREQLRTSLLTGDAF

DPPDCELRFPGQWADEESGLHYNFHRYYDPATGQYLSPDPLGLAGGLRTHAYVHDPLQWVDPLGLQGYQG

SGKPEFIGNRKLPQNDLPWIKYQKRVTGRPYEETWRVGDHNVNLDGKRAGYTVEAKWTGKNSAAWESSPY

NPEHEFYNESKILDQAGRLLEFNDASGGSGVRYAVSNAEAQQHFTQLFEQHFPTEMQDGTLSVWHVPGNG

M

>WP_004539109.1 Nramp family divalent metal transporter [Burkholderia pseudomallei]

MLSSTTMSRSAGDFATAGDASSRTVRAARDALEGRRKGVAAALPFVGPAVIASIGYMDPGNFATNIQAGA

AYGYELLWVVLAANVIAMVFQAMSAKLGIVTGRNLAELCREHFPRPVVWGMWATSEIAAMATDLAEFLGG

ALAFGLLLHLPLIAGMAATAVLTCAILTLEKRGFRPLEAAIAALVGVIGASYLGELLIAPQDWHAAAYHL

VVPQLRDGTALTLAVGIIGATIMPHTLYLHSGLTQRRTTPRDARERRLLLRFSNREVAVALGVAGFVNIA

MVMMASSAFHRAAPGMADIGDAYHTLIPLLGPAAGALFLVALFASGVSSSVVGTLAGQVVMQGFLRRRVP

IWVRRLVTIAPAFAIVALGCDVTRAMVLSQVVLSLVLPLPMAALLLLSSRRALLGEHALRAPTLVAASAA

AAAIVALNVYLIWAAFN

>WP_004534713.1 ABC transporter permease subunit [Burkholderia pseudomallei]

MHSTRRRTTDDRRARDAQAMSRRAAHLACALPVAARGAVISSSSTPPIILVDENVYIALRTPMTPPPKDA

AALARPAAAAGPHAPPAVRRARRAPLDWLAAAQWAVTLALCAFLIVPVAMSVLAGLTVNYFRGPSSGLTL

RWLGEVWAQYHGSVFLSLEVALATLAVTLVAGVPAGYALARSRSRVSRLIEEALVLPVALPGLASALALL

SVYGGFAAFRTSLWFIVVGHVVFTLPFMVRAVAAVAARADLRTLEEGAASLGASFATRFATIVLPNLRPG

IVAGALAVLTLSIGEFNLTWMLHTPDTKTLPVGLADTYASLRLEIGSAYTILFLLMTLPLLVAMQRLGVE

PPGARDRTRDRTCKRR

>WP_004523537.1 MULTISPECIES: LysR family transcriptional regulator [Burkholderia]

MDNLRRLDLNLLITLDALLSEHNVTRAAARLNLSQPSVSIHLAKLRDIFGDPLLLPGPRGMRPTARADEL

REPLRRALETLGRAVSPGAPFDPAASAHTWRVAASDYSESTVVLPALNGLRAAAPGTRLAVVETVPSRIA

KQAEQGDIDLAFHITECAPGNLRHRALFTERYVLVGRAGHPRLKRRPTLAQFCKLDHVIVSPDGGGFQGG

TDHALAKLGLARRVVLSVPHFLFMMSAVANSDLVAMMPSRLVRDSRALQVVEPPVDVPGFRMAMLWHERS

HRDPAHQWLREHIAASV

>WP_174905337.1 DUF1311 domain-containing protein [Burkholderia pseudomallei]

MAAAAFTMATPPRQAKPSQAKPSQAKPSRRRQCRRCYRYRHGIDIDSGNSNSNSNSNSNSNSNSNSNSNS

NSNSNSNSNSNSNSNSNSNSNSNSNSNSNSNSNSNSNGNGNGNSSRHRYHPHLRRTPYERLSNYPLEVPQ

GGSMSTNMKRLMTAALGAALAFGALSARAASFDCAHAANAAERAICGTPALGELDVRMAAYYEMLQNARP

ADEGMAYREFRDALRDEQQRWRQRTRDACGARIDCLTNAYTARIAALRGVAAERLVLRMTGGSAASAGAA

DATYAIEGESITLANGESVRPAAPGSAMKRVTTLVARSAVATIAGRPVEAVLLSDDPGGSGRFLYVATAQ

PGGGAPAVLLGDRVKPVSVSIERAATGGAVVVVEYLDRPEGAPFAQAPTIKIVRRFALEQGRLVEQRG

>WP_038799955.1 VirK family protein [Burkholderia pseudomallei]

MVRKNSIVSSLVLSVAITSGTSFANASSSEGAFLESAVMAGKDIHVILDLSRCIEHGTQIPGPAVRGSVR

PDTFMILSDHSIAFSNTHFTVPADNKPVQEFMKYRANGDGKVEFQTMVLDPINFSVLRKNQYDCEVNKGV

KFFW

>WP_174904625.1 hypothetical protein [Burkholderia pseudomallei]

MTALPDASHPSSRVAWPAVEPGFAAASVRAMPIDAAARGIARLCRAVARAVRQCVARIGRARDDCEDCED

SDAHDDRHDRGSLDARSEPAPMPRMTTRAATRASASRPTESKRSRLAIRRDRRRASQTAASIDRIASANP

SGSSPAPRRAGNTDLQRRSVTILALRAEAGAASDSELSEAAPKHGSLGQPAGGHDMRRALRHGDALAHLI

GIVQVVQIAGAVRATANVLVLRPPAGTLFESGIHACPGALQRPLAEAVKLAPAICSTFAAGRRRVAGGAT

HARWRTAAPRVPVRADRADDATAGVDARRMPATPGRAYCPSGRCGSRRAARASAIAKASASASGRRGSGD

ARDVGGPAASRIAANLLHARPASAGRVTAGAIGIIETKAAGNAGSRQDNHSPSPR

>WP_009970051.1 cytochrome c oxidase subunit II [Burkholderia pseudomallei]

MEILGKEAMKTIKRALTGVLACSALLLSGAALAVGDSPGGPRVNEINLQPPVTKIAEELYDLHTMMLILC

TVIFVGVFGVMFYSIFAHRKSKGHKAANFHESTTVEIIWTIVPFIIVVLMALPATKAVVAMKDTTNADLT

VKVTGYQWKWGYDYVKGPGEGISFLSTLSTPRTEVNGRQPISDTYLQEVDHPLVVPVNKKIRVITTANDV

VHSWYVPAFGVKQDAIPGFVRDTWFKAEKVGTYRGFCTELCGKEHAYMPVVVEVLSDDDYAKWVGTQKAK

LAAGAVDPNKVYTRAELMAHGEEVYKANCAACHQPNGKGVGAFPALDGGKIVNGPIAGHLEQVLKGKGAM

PSWASLSDLDIASVITYERNSWGNHKGDSLQPKQVADARNGKLPEDAQQADGGAAANAASGAAAQTQAQA

PALPAAIYFETGKSELPADAKDAIAAAAEYVKAHPDAKLALSGFTDKTGSADANAELAKRRAQVVRDALK

TAGVAEDRIILKKPETITGGADAKEARRVEIGPAA

>WP_004522912.1 phosphoenolpyruvate--protein phosphotransferase [Burkholderia pseudomallei]

MIKEVRVSFTLHGIPVSRGIAIGRAYLIAPAALDVAHYLIEAERIEAEIERFRTALGAVRRELDVLRADL

TDDTPTEVAAFIDVHAMILGDAMLVQETIDLIRTRRYNVEWALTEQLDVLAGHFDDIEDEYLRERKADIE

QVVERVLKALAGAPSAAQALDRAAGNGRDEMIVVAHDIAPADMMQFKTQSFQAFVTDLGGRTSHTAIVAR

SLGIPAAVGVQHASALIRQDDLIIVDGDQGIVIVDPAPIVLEEYSYRQSEKALEQRKLQRLKFSPAQTLC

GTKIDLLANIELPDDAKAAVDAGAVGVGLFRTEFLFMSKVRMPEEEEQFAAYKRAVELMHGMPVTIRTID

VGADKPLDVHDEGYETAPNPALGLRAIRWSLSEPQMFLTQLRAILRASAFGQVKILVPMLAHAQEIDQTL

DLINEAKRQLDAAGLAYDPNVRVGAMIEIPAAAIALPLFLKRVDFLSIGTNDLIQYTLAIDRADNAVAHL

YDPLHPAVLHLIAFTLREAKRAGVPVSVCGEMAGDPALTRLLLGMGLTEFSMHPSQLLVVKQEILRAHLK

ALEKPTADVLASFEPEEVQAALARLASAEPRADVAA

>WP_173671368.1 lipopolysaccharide heptosyltransferase I [Burkholderia pseudomallei]

MPATAGLFFSVQKILIVRVSSLGDVVHNMPVIADIRRRHPDAQIDWLVEEGFADLVRLVDGVRDVLPFSL

RRWRKRLSASQTWREIRAFRRRLAEERYDLVIDCQGLIKTAWVASWARGPLVGLGNRTDGAGYEWPVRFF

YDRRVPIAPRTHVVERSRQLVAAALGDPAPAPGEPIDFGLDTHGAARALAALDLNLPVPYVVFVHATSRA

DKQWPDEAWTGLGEALVRRGASLVLPWGSDAERATSERLAKAFGAAAIVPPKLSLPAVVGLVDGAAATVG

VDTGLVHIAAALKRPTVELYNFATAWRTGGYWSPNVVNLGTAGAPPSLSQAKDALASFGLL

>WP_004197996.1 MULTISPECIES: cytochrome c oxidase subunit I [Burkholderia]

MSSIGHDVAAGHAHDDHAHETPHGWRRWLFATNHKDIGTLYLLFSFIMFLSGGVMALAIRAELFEPGLQI

MRPEFFNQLTTMHGLIMVFGAIMPAFVGFANWMIPLQIGASDMAFARMNNFSFWLLPVAAVLLVGSFFSP

GGATAAGWTLYAPLSTQMGPGMDFAIFAVHIMGASSIMGGINIVVTILNMRAPGMTLMKMPMFAWTWLIT

AYLLIAVMPVLAGAITMVLFDRHFGTSFFNAAGGGDPVMYQHIFWFFGHPEVYIMILPAFGIVSQVIPAF

ARKPLFGYSSMVYATASIAILSFMVWAHHMFVTGMPVTGQLFFMYATMLIAVPTGVKVFNWLATMWRGSL

TFETPMLFAIGFLFVFTMGGFTGLMLAMAPLDIQYHGTYFVVAHFHYVLVAGSLFALFAGWYYWAPKWTG

WMYNETRGKIHFWASMIFFNVTFFPMHFVGLAGMPRRYADYPAQFTDFNQLATIGAFGFGLAQVYFLFAI

VLPAYRGGGELERASDKPWDGATGLEWTVPSPAPFHTFEHPPTVE

>WP_004547411.1 collagenase [Burkholderia pseudomallei]

MPMTEVFRKTRRWSAVAALSAFVGLAGAASANTQPMQPTQQKQARMPRLPQNLPVSPEQAEYNLPLSEQD

RAALTRPSPLKQPAKRGKRSAPGADCRDMSVMTQYRGAALADYIANLPDYECHYGLFSVDKTLAAQIFSA

ENVHAVASRFVQDIYRYDASNLILVNLLIYLRSAYYQYDVSGIANPIPNLAVWLRPYIKQSLEGAALYRE

NARAPSTANELMKLITNMKDEAFYLPTLKARIAFYTASATNPQAAAPLLQPSAAGGFTGLLTVFFYAHQR

SGAQPMLDSDATLPETLNRFVTANRASLSNTSAAYQLADAARETFRFLRYPAQKPRVKKMIQDMLASTSM

TGADSDLWLAAAEAVDYGDPGNCADYGTCDYKKRLTDAVLTHRYACNAGVRILAQDMTLPQLQSVCTSVA

QQDDYFHRMMKTGRKPVAGDRNDTIELVIFDDYANYRKYASVIYGISTDNGGMYLEGDPSAPGNQARFIA

HEASWLRPEFKVWNLEHEFTHYLDGRYDMAGDFAASTAKPTVWWIEGLAEYLSRKNDNQESIDAARTGAY

RFSDVLGTLYSSSDYVARAYRWGYMATRFMFERHRADVDTIVSRFRVGDYDGYANYVAYIGNRYDGEFVD

WARAATTAGEPPLPTKR

>WP_004541849.1 GspH/FimT family pseudopilin [Burkholderia pseudomallei]

MFSMYARSGTIFVCDTRRHAGAALGGAAKARAGACAGVSSGRPAGTASARRARGFTLLEMLVVLVIAGIL

VSVASLTLRRNPRTDLREEAQRIALLFETAGDEAQVRARPIAWRATEHGFRFDIRTGDGWRPLRDDVLRA

RDWDGGVTGAAIDYPGSDTHTDAVVFGTESIDVPVRVTLYSAVGSATIVGTGNGRYEVR

>WP_004528172.1 NAD(P)/FAD-dependent oxidoreductase [Burkholderia pseudomallei]

MNFDVVIIGGSFAGQAAALQLARARRGVLLIDAKRPRNRFARTSHGFLGQDGHAPSAINATAGRQLAAYP

TVQFDAGDAIDAAAVSDGFRVSLADGRQAQGRRLILATGVRDILPVLPGLQERWGVSVLHCPYCHGYELE

RRPMGVLASSEQAVHQAMLVSDWGPTTLFTQGAFVPTREQSAMLSARGVTVEQTPVAALVGAAPALEAVC

LADGRTAALCGLFIAPRTVPACDLPERLGCVFDDGPTGPYLSVDERQQTSVPGVFAAGDVARPMANATLA

AAAGVIAAAGAHHSLIYGAGERG

>WP_004522282.1 MULTISPECIES: S53 family peptidase [Burkholderia]

MKNNSSLSILIAAACIQAFAATASLAQGPAHPPSYVEGTRVPKGFARPPFHTNPARFSATTVSGLAPATV

RHAYGFDSIANQGDGMVVAIVDAYDDPKIESDLGVFSKNFSLPPCTTSNGCFKKLYASGSKPSPNAGWAL

EMSLDVEWVHAIAPKAKIVLVEAASNSFNDLMTAVDVAVGAGASVVSMSFGGSEFSSETSFDSHFGAPSN

VTFVASSGDSGNGTEYPAASPYVVAVGGTTLSADASGNYVGETAWSGSGGGVSAYELEPVGQTLWPIPYA

GQRGVPDVAYDANPNSGFAVYDSVTYQGQSGWFVVGGTSAGAPQWAALFAIANSMRTAAGKAKLAGAYNQ

LYTVGKTAYGSDYHDVTSGTNGSCGMICTASGGYDYVTGLGSPQALNLVQALVAQP

>WP_004197876.1 hypothetical protein [Burkholderia pseudomallei]

MKETNMSLAAQDLIELLRLAQGGAGALTLHLLREIAGNLIAFGVADCRGGC

>WP_004185781.1 MULTISPECIES: GspH/FimT family pseudopilin [pseudomallei group]

MRAERDRWATAGFVLVEVMAALMLVALAAMLTAPTLAGARMRDRVDARARVFGALLAYARGEAVRLGARV

VLCRSDATAKCIAAGRPCGGGAADWSCGWAVAAADGERGTRLLRRIAPDARVAVTGTTVDVVFTPPAGQV

IGGFRSFEFAPADASGAWRGERWRRCLRIAAGGRVRFAEGGCGAST

>WP_172848208.1 hypothetical protein [Burkholderia pseudomallei]

MISRPVSGAGARAPRRPGRTGTDMKARPMLEYALHPLEDRLVHVDELCRADPVPRGWARCPLCLEALYVV

QLRDRSHARRFAHLAGEFARCPLVNDALPNPLAVHVGPPLDEHGRQARATFFRHWQRHLHTIRQTASAFG

IARFMRAIELADVLKLWAWPTLAQRDIPYILLVLTEFIAAPRGERKQAAWSRFWFDASVQRVDDLRKPRG

VLPRLFRLRYRLPRMSKFPNARHLIDCQPVLMNDIGPSDAALGTPRADIAMFEAFAARFARRPAE

>WP_004531734.1 YadA-like family protein [Burkholderia pseudomallei]

MRGQLIAVSEFSRSNGKCSTTQVVTAAPGVAGRTAASGRSRPSWTKLGLMSLAVSAAMGCMATDAAAQVS

YAAGENAYAGPGGNTGPWAFYNPAFSAGTLLYGTAVGNYAYANGEGSSAYGDHATVKGRIGSAFGAYSEA

AGDGSTAIGASARALPDFSIAIGTNAQALKDTGQSIPGREDIGTIAIGAGALAQGDNSDPLHVSAPNAFG

GYSSATASGAVALGEGAASSGYYANALGSYSKASGAGAVAVGGGAQASAQGAVAIGGATSVDNATALSGY

ASASGVNAIAIGSGAQATGARSISIGTGNVVSGASSGAFGDPSTVTGTGSYSFGNNNTINSNNAFVLGNN

VTIGPGFDGSVALGSGTTVAAANPTGSATITTSSGGQLTLSGFAGANPTSVVSVGAPGAERQITNVAAGR

ITPTSTDAVNGSQLYAVASTIDNAVNGGGIKYFHANSTLADSTAAGTDSVAVGPAALAYGNDSIAEGTNA

TAGVSGNPAVAGDVALGSGAQATGGRSLALGANASVNTAGGVALGAGSVANRAAGTYTDPITGSSFTTAF

GAVSVGLEGSLRQITNVAAGTQATDAVNVGQLQGAIAQLNQTIQNITNGSNSGNTGNNGNNTGQTVSGQW

ITGNPSTYTPPVASGIGSTAAGSGSVASGANSVAIGDGASASGNNSVALGAHSVASAPNTVSVGSVGNER

TISNVAPGVNGTDAVNVNQLNSGIGNAVGQANQYTDQKVDHLRREMNGGVAAAMAVAGLPQPTAPGKSMV

AIAGSTWQGQQGFALGVSTISENGKWLYKGSLTTSTRGGTGAVLGAGYQW

>WP_172625310.1 MerR family transcriptional regulator [Burkholderia pseudomallei]

MLKSNSTTPADVAPDASMSNEYTVDELARVSDTTVRNVRAYQDRGLLAPPRKRGRVGIYDDTHVARLKVI

NHLLARGYTLSNIQDLIKAIDDGHDLRSILGLENAIGGRWSNERPQTFSLAQLIQMFGPQTPASLGRVAE

LGLLERRGVSFVAKSPALIEAAAAMVREGIPARELLDAISITRPYFDAIARVLVELVVRRLDRYDEGSLP

PPTDVPALVDAIWRLRPLSSVFVEGEMNRALEAASSAYLGGRVATILDKKLSSQAAREGEPELERVTAKG

APKKR

>WP_004526219.1 lipopolysaccharide heptosyltransferase II [Burkholderia pseudomallei]

MRRALVIAPNWIGDALMAQPLFALLKKLHPRIVIDAVAPAWVAPVLERMPEIHDVHATELAHGKLQMLHR

WQLASDLRELGYDAAYVLPNSLKSALIPWLAGIPLRIGYTGEHRYALLNVRHANPGKARETRAPMAQHYA

ALAYAPGAKLPESFQTLPPPRLDADLNETARVSARFNLDTRKPLIVFCPGAEYGPAKRWPPEHFAALAQS

VSQSFPYTQIVALGSPKDAAAAQAIAERAPNVRSLCGQTSLTEACALIARANAVVTNDSGLMHVAAALRR

PLVALYGSTDPRHTPPLSELAKVQWLHLECSPCFERECPLGHLKCLRELSPEQVFGDLRGMLVGQR

>WP_004522315.1 lipopolysaccharide heptosyltransferase I [Burkholderia pseudomallei]

MKRILIVKVTSLGDVVQTLPVVADLHRAFPGVQVDWAVDESCADVVRWNTGVSRVLCAPLRRFKKARSPA

DLKAIAASISELRAHRYDAVIDLHGVYKSAIISFLARARRRYGYRNQDLGELGAMFAYNGRFGPRPACDA

WHGMRVSAGQALGYEPQGRADYLLDVPPESREPHTAPLVALAEPGGPYALFFHATSNDDKRWPTDRWSAV

AREMRARGLRVLLPWGSEREREEAQRIAARAPGAVVLPRLTLADVARKIDRAALVIGVDTGFVHMAHALE

KPTVMIFCATSRQHLGVSGAPHSLSIGDEGATPGVDDVLGAIEHVYPTGAVEQPRRVAAM

>WP_172408686.1 sorbosone dehydrogenase family protein [Burkholderia pseudomallei]

MRCSLVALFRHVSSSPPALPRAAPAACLVLATIVTVATAPAASAALPIDELRVPPGFRVQVLADDVPTAR

EMAWSPRGILYVGSMNGRVHALVVRDGHVREHHVIASGLEMPVGVAYRSGALFVSAVSRILRLDRIDERL

AAPPKPVVVTNALPTDRHHGWKFIAFGPDGKLYVPTGAPCNICVADRDRYAMIGRMNADGSGYEVYARGV

RNTVGFAWHPATRELWFTDNGRDLMGDDRPDDKLNRAPRAGLDFGYPFCHGGDVLDPQFGRGHTCSSYAP

PVLKLGAHVAALGMRFYTGGMFPPEYRDNIFIAEHGSWNRSRKVGYRVVRVIASPDGRAAREETFVHGWL

RPDESVWGRPADVLPLPDGSLLVSDDYAGAIYRITYDATH

>WP_076851503.1 DNA mismatch repair protein MutS [Burkholderia pseudomallei]

MATQIDASSEAAAATAAAQHTPMMQQYLRIKSEHPDTLVFYRMGDFYELFFEDAEKAARLLDLTLTQRGA

SAGTPIKMAGVPHHAVEQYLAKLVKFGESAAICEQIGDPATSKGPVERKVVRVVTPGTLTDAALLSDKSD

VFLLALCVGHNKRGVASNIGLAWLNLASGALRLAELAPDQLGAALERIRPAEILAADGTIESVPAGMGAI

TRVPAWHFDIASGTQRLCDQLEVASLDGFGAQALTSANGAAGALLIYAAATQGQQLRHVRSLKVENESEY

IGLDPSTRRNLELTETLRGTESPTLYSLLDTCCTAMGSRLLRHWLHHPPRASVAAQARHQAIGALLDAPP

NAGLDSLRSALRQIADVERITGRLALLSARPRDLSSLRDTFAALPALRERVAEIASNAAALGRLEAALEP

PPGCLDLLTRAIAAEPAAMVRDGGVIARGYDAELDELRDISENCGQFLIDLETRERARTGISNLRVEYNK

VHGFYIEVTRGQTDKVPDDYRRRQTLKNAERYITPELKTFEDKALSAQERALARERALYDGVLQALLPHI

EGCQRVASGLAELDLLAAFAERARTLDWVAPEFTDEIGIEIDQGRHPVVEAQVEQFIANDCALNPERKLL

LITGPNMGGKSTFMRQTALIALMAYVGSYVPAKAARFGPIDRIFTRIGAADDLAGGRSTFMVEMTEAAAI

LNDATPHSLVLMDEIGRGTSTFDGLALAWAIARHLLSHNRCYTLFATHYFELTQLPAEFPHAANVHLSAV

EHGHGIVFLHAVEEGPANQSYGLQVAQLAGVPAPVIRAARKHLAHLEQQSAAQATPQLDLFAAPPVVDEP

ECNEPPAATPHPALERLLELDPDDLKPRDALDLLYELHTLARSGPADAQR

>WP_004525771.1 type II secretion system minor pseudopilin GspI [Burkholderia pseudomallei]

MMMRAPAPPRSPARSRGFTMIEVLVALAIIAVALAASIRAVGSMATGASDLHARLLAGWSADNALAQLRL

AHAWPDIGMQTFDCSQGNVALTCTQRVSSTPNPVFRRVEIAVSMNGRAGVLAQMVTVVANETSRPL

>WP_004524671.1 MULTISPECIES: putative zinc-binding protein [Burkholderia]

MNHETKTLPIVYSCSGCSNVAQLANHVAVRLDRGGDAEMSCIAGVGGDVPSLLKIAHSGRPILAIDGCPL

VCAKKSLERHGIAPDAHLQLGEHGVRKRFHEDFAPGDASRILAIAKAEAARLAGSRPAPEPELAQAGEDA

GAALERKR

>WP_004189458.1 MULTISPECIES: recombinase RecA [Burkholderia]

MEESKKGSGLTAEKSKALAAALAQIEKQFGKGSIMRLGDGEAVEDIQVVSTGSLGLDIALGVGGLPRGRV

VEIYGPESSGKTTLTLQVIAEMQKLGGTAAFIDAEHALDVQYASKLGVNVPELLISQPDTGEQALEIVDA

LVRSGSIDMIVIDSVAALVPKAEIEGEMGDALPGLQARLMSQALRKLTGTIKRTNCLVIFINQIRMKIGV

MFGNPETTTGGNALKFYSSVRLDIRRIGSIKKNDEVIGNETRVKVVKNKVSPPFREAIFDILYGEGISRQ

GEIIDLGVQAKIVDKAGAWYSYSGEKIGQGKDNAREFLRENPEIAREIENRIRESLGVAAMPQGAGSEAE

IMDEEE

>WP_004189176.1 MULTISPECIES: carbonate dehydratase [Burkholderia]

MNKNDHPLSHLFDNNDAWVKRKLADDPQYFSRLADQQAPEYLWIGCSDSRVPANQIIGLPPGEVFVHRNI

ANVVVHTDLNCLSVIQFAVDLLKVKHVMVVGHYGCSGVNAALHNRRVGLADNWLHHVQDVREKHAALLED

WPLGEARYRRLIELNAIEQVVNVCRTTIVNDAWARGQPLTVHALVYGVHDGRMRNLGMAVSHAEQLDATY

RRAVAALSASGAHSADNDVVAADAAQLAGAVDLIAQTIKETKHDGC

>WP_071810525.1 type 1 glutamine amidotransferase [Burkholderia pseudomallei]

MSENTSARADQSGSSSAATPPEHTGQAPSSSSVTDKPPAGATVHVGAFAAQIAAAQDIGDPGSAPSGAIA

EPGAPGIAAGGPGVEPAAVSAGPGAHAAAPSGATGAGASAAGAAAAGASAKAGSPPPGFGAPPDFEASRP

PPPGAATPAPPAYLKQSDTPWSVFGRIIAARARRLFDRAGQRITQRTLRIGVSARIFHPEVGAPGLRGKT

LQYLEESIAHWVMSRDVLVFMIPTVGHQGMLHPSNIRLRDYAKHLDGLLLQGGADVSPQTYAASDSRPEW

PGDRVRDMYELELLHEFVESGKPVLGVCRGCQLINVAFGGSLYQDIASDVPTAGAHVSEHYDQHRHSIRF

PDGSTLANMFPGRREAIVNSIHHQAIRDIGRDLNIEAVSAEDGIIEGIRYRRAPFVVGVQWHPEFHRAGG

PELLDCTPLLDTFLRAARETRL

>WP_024428656.1 type VI secretion system membrane subunit TssM [Burkholderia pseudomallei]

MQRFLNVLTHPRTLTIVGFAALAAVLFIAADALQIGLAWAAVALGVALALWLATKLWRRWRVRRANRQLG

DMLEQQAETGKMSAAVAEPAKRAELDVLRTRLADTVKTIKTSKIGQVSGGSALYELPWYIVIGNPAAGKS

SAVINSGLQFPFADKNSAVIHGIGGTRNCDWFFTTEGILLDTAGRYSVHEEDRSEWLGFLDLLKRYRPKA

PINGIIVTASIAELTGNRPEFAINLAKNLRQRVQELTEKLEVFAPVYVMFTKADLITGFTEFFSSNDRQE

YDRVWGATLPYEPDEKRDVVALFDQRFEELYDGLKEISVAQLSVSRGNKLSPGQLSFPLEFSTIKPALRS

FLATLFEDNPFQYKPIFRGFYFTSALQEGETNSAAAQRIAHRFGLDSQSLPKPHSAFSKNGFFLRDLFSK

VIFADRQTVRQFASPTKTRMRYATFFGFVAALALALGGWTWSTIGNQQLVSNVQADLDNVTRLQQNRNDL

QSRLQAMDILEDRIEQLEQFRRDKPLSVSLGLYQGNRLEQHLLTEYYNGVRQILLAPVSDNLASFLKDVN

AHPDQLAPMTRAPESGATPAGPMPVSTHPAAPGAAPSPAVAQGRQQGGLYNDASPTNVEDAYNALKTYLM

LSDKRHVEQAHLTDQVARFWRGWLETNRGNMPRDEMIKSAERMITFYLSRVSDNDWPMIEANLALVDQTR

ENLRRVVRGMPARQRVYEEIKARASTRFAPMTIARIVGEGNTGLVAGSYAIPGTFTRDAWFQYVQPAIRD

AATKELQAKDWVLNTASADDLTLEGSPEQIQKTLVAMYKTEYAQHWQKFMQGIAVQGFGNFTQAVDAMNK

LGDPQDSPIRKVLETAYDQTSWDNPSLANATIKKAQTGVVNWVKQWFTRQPGGQIAANVDINGNPADVPM

GPIGQEFVGLGRIVATHDGTSMLKGYMDTLSKVRTRFNVIKNQGDPGPGARQLMQQTLDGNGSELADSLK

FVDEQMLTGLTDSQRKSLRPLLVRPLMQAFAVVIQPASVEVNKVWNAQVYQPFQNSLANKYPFAASAKVE

AGAGEIAQVFGPDGSIAKFVGTTLGPLAVRRGDTLSARTWGDMGLALTPDFTGGFARWVAPLAGGAATSA

AASSEPQTVFQVLPQPSSGTTEYTIAIDGQQLRYRNTPPQWTNFVWPNPQGSPGATLSATTFDGRTLQLV

NEPGRYGLEKLINSAQRKRRPDGTFDLTWTQGSVSVSVTMRIISTSQPTGGGDQPQQQSLRGLRLPSSVA

DASAGGAANATAQPGSGAAAAQVAVTAASASNAQGAQ

>WP_004540541.1 MULTISPECIES: HU family DNA-binding protein [Burkholderia]

MATSAKKVAKKAAAPAKKVAAKKAAPAKKVVAKKAVAKAPAAPTPLKDKFTKASLATHIAERAAVEVKAV

KAVLAALENVVLGSIHKKGAGEFTLPGLLKITAQAVPAKKKRFGKDPFTGAERWFPAKPASVRVKARALK

KLKDAAA

>WP_004535338.1 D-(-)-3-hydroxybutyrate oligomer hydrolase [Burkholderia pseudomallei]

MTAIRGGSRRAPGLALALLGGVLLGACHGDENAQVNALPGFVSGSVRKTAYDGASDDLLTAGLGKTGLGS

DTRPGFANPAQPSAAELRRLAIYSNYRALVDITPNGGYGRFWGPNVDLAGNDTLGEGKIAGTEYLAYSDD

GSGRKNVTLLVQVPASFDPANPCIVTATASGSRGVYGAIAAAGEWGLKRGCAVAYNDKGGGNGAHEIGTG

VVTLIDGTLATASSAGSSSLFTASESSSTLAAFNSAFPNRYAYKHAHSQQNPEQDWGRVTLQAVEFAYWA

LNEQFGPVVDGTRHGIRYRPGDITTIAASVSNGGGSALAAAEQDTRGWITAVVVGEPQINVRMTPGVTVE

QGGAPVPSFGRPLADYATLANLLQPCAAAAVAATGAPYLSALPMGVTQSIRTQRCATLAAAGLVSGADTA

SQASDALAQLHAAGYLADSDLLQAPMWDSQAMPAIAVTYANAYTRSRVTDNLCNFSFATTNPVTGAVAAP

AVSPMTNLFGAGNGVPPTNGINLVFNGASGGVDHRLATPDASFAGAFCLRQLWAANQLGIGTNVDAVRVA

ANLQHKPAIIVHGRSDALVPVNHASRAYVAQNSATEGRASQLSFYEVTNGQHFDAFLSVPGFDTRFVPVH

YYDEQALNLMWNHLKSGAPLPPSQVIRTVPRGGVPGAAPALSTANLPPIVQSPGANAIAVNAGVIDVPL

>WP_004531854.1 UDP-N-acetylglucosamine 1-carboxyvinyltransferase [Burkholderia pseudomallei]

MQVTVNEHDAVERVATATPAGNREAHAHGTDKLAIEGGRRLAGEIAVSGAKNAALPILCAGLLSAEPVRL

DNVPDLKDVRTTLALLGQMGMREETDGARVVLDASRVDNPVAPYELVKTMRASILVLGPLLARFGYAKVS

LPGGCAIGARPVDQHIKGLQAMGAEIHIEHGYIEARAKRLSGARIVTDMITVTGTENLLMAATLADGETV

IENAAREPEVTDLAHLLVAMGAKIDGIGTDRLVIQGVERLHGATHAVIPDRIEAGTFLCAVAAAGGDVTL

TGMRAHILDAVIDKLREAGATIDEGVDTLRVRMDGRPSAVAIRTSEYPAFPTDMQAQFMALNAVAQGVAQ

VTETIFENRFMHVQELNRLGANIAVDGNTALVTGVPKLSGASVMATDLRASASLVIAGLCAQGETLVERI

YHLDRGYDRMETKLTAVGANVRRISGSEA

>WP_004529645.1 type VI secretion system membrane subunit TssM [Burkholderia pseudomallei]

MSYLKRFLRFVFSWQMLACIAVLLVCAAVWFVGPLLAFDELRPLAGVVVRVAVIVLLVALLAFWLLRWPL

SPIGVAALCLLIWHAGPLFAFGDHRPFGPAWVRVLIVAAMLFCYAVYGLYRLWQAVRTNDALLKRILEPS

AGKPDAAAQANIRAVSVAVSKAIGQLKRLRGGAFGWRRLLESGRYLYELPWYMVVGAPGAGKSAAIARSG

LKFPLADQMEASSERARGGTANCEWWFANEAVLIDTAGRYVRHEVPGDEEATVANGAEWKGFLGLLRKHR

PRAPVNGVVLSVSVEELVGRTPAERTAHAATLRARLGELHQELGIHFPVYVIVTKLDLLPGFPEYFQSLT

AEGRTQIWGFTLPYDAENRRSAVGALREHCADELKRLEMRIDAGLNNRLLEEYENDRRKRLYALPQEFRS

LSAALSDMLALVFLDSRYDDAQLQNTLRGVYFTSAEQTDQVLAADRETILQRLKRQLGRMLGGDAGAQTR

DSGAVSGSRGYFLRDVFQHVIVPEAHLVRPNVRWEVRFRLMRWAGHLLAVALLVWLASALTVSFDNNRGY

LDAISEKTAALAARVNAYNKAPKPAQIGGVLDGARDLPQHGNLDLDAPGASFRYGLYVAPGIVDASDATY

RSLLRRSLLPQIVRRVENALSAQIDAKHADEVYRTLTVYLMLDDAARHDAKAVKDWVMRDWERSDSAAEM

GGRNLMARHLDALFVDARPFEPSGHRNAALVQRARLFLNANPAPRRLYERAMAAIEKEAPENFTLARAVG

LQGAGIFRLADGSRFQRGMPGLYTYDGYHQVFSVRLPEFLARAQSDDAWVMGNAGPAARWGDAIRNTQAV

AGRSSLADDIRRQYLTDYGNYWQQYLADIRPASSGEDGAGGTLAFDLATLRALAAPDSPLVRLARAVVRE

TSLSVVDAREDASLTDAALSALGRRSGAAKEVADGAQKLAARRPEQRLEKEVVDNRFAALREVVTGQADT

GSGPAMTDVPVAAGGRALQLDAILTLINEQYTRLVVADNALSSQSMPPALDIGTTLQMEAEKLPAPLRTV

LGGIATQAADKVGREVGSLLAMQVDSSVGNACRAAVDGKYPFARSAQEVDIEDFNRLFAAGGLFDAFFQK

ALAPHVDTHSKPWRYKALNPGMPPIRGPSLEPFERAAAIRDVFFREPGAKRMAWKMDAKVASIDPEITEL

IVDVDGQSQRYVHGPVLPFSVNWPGPRGGAIAEITAKPRIRPDTSTIATTGPWALFRLIERGRLTGTTSA

SRLMLDFDFDGRHAALELRTNGQMNPLTSGLLTHFRCPGSVG

>WP_004527586.1 lipid IV(A) 3-deoxy-D-manno-octulosonic acid transferase [Burkholderia pseudomallei]

MLRAIYRGLWWLVAPLAVLRLVWRSRKERGYREHIGERFGFGPGRALARDVDEATPIVWVHAVSVGETRA

AQPLIDALLRARPDAHVLLTHMTPSGRATGEQVFGERVSRCYLPYDLPRAVRRFLRAWRPSLGLVMETEV

WPTLIDECRRADVPLVLTNARMSARSFGRAAKFGAAARDVFGGFSRVLAQSPADAQRLTSLGARNVAVLG

NLKFDMTTPPELAARGRAWRAAIGERPVWVAASTRDGEEALVLDAFVSLKTPGALLILVPRHPQRFAEVA

ALVERRGLRHVRRTEWAHDAAAAAAGRPAASALPADVSVLLGDSMGELGAYYAAADVAFIGGSLLPLGGQ

NLIEACAVGVPVLIGPHVFNFTQATADAVTAGACAQVRDPADLACTLDALFADHARRTAMGAAGAAFAAR

HRGATARTVDVLNALLPAPRVAPAANDEGDAGGLDAGAALD

>WP_004525554.1 MULTISPECIES: type VI secretion system membrane subunit TssM [Burkholderia]

MIRTSLRVFAAILIAILIWWVGPLFAFGIYHPLGPVWVREILVALVLIWGFWPTLARLWARLAMSPRQVK

VAPKAKQLDFVDKHLRTLDQQLKERWRKEPRGRWKRWVGALTREHRTMLPWYLVLGSEGSGKSSLVAKAV

SVSGSLQDRVLGSDATYGRGDDLNFRITREAVWFDVGGRWSLRAGADEAEFDAWRKLLRGMRRLRRGAPI

SGVVLCVDGLEMIDAPLDARKRLADSVRARLEEMREAFGQQVQVYVALNGLDRLDGAVSTLSLLEASKWV

KGVGFSLPDDGAHADAARADASWQHALQGLQQRVQQQVLYSAPAATEVSMNHAQLRFVETLSRLQKALVA

WLHVALAPGEPHTAARLRGVWLGSMAELAEPHPAGAGGVGSAELPVPSRPLSELWTPLIRQVALERDAVR

PSGPKSWRGRLGEALRWGAVPLVALSLLLWFGWGYVTERDYLDGVWAQFTEAKRLAQAEASYGNDGGSTL

IEIANQMRYAQLQAEDAAQGMATPYFEHGLVAETARETYYRHLQKMLMPELYNEVRRTLVSQVDGSPGDI

YQTLKVYLMLCRPARRSADDVVRWLDGRWDALSGGQYSDDDRRSLLAHVRTLMSLKEVPATPEDANLVRS

ARAKAAQIPLVTRVLQHIHAQGLPQQVNDISLSRAAGFEASMSLRMRSNVPSTDTAVSGWFTRAGYTDVF

LPRLQKSARAMLEEESWVLRDETLSGNSFQIDGLVQKLADSARNQFLQDYISAWQNFLNDVTVRGVTGLD

DASQLAAAMMEAQSPLANLLRFAARETTLTGASDEGNIDSWIDRQKYRFEKGRRQVVGELSGQHYRTVLL

PEHVVEEHFQAIRQLAAQLNRNNTIANNPLSRLFEPLYRQLGLVNGALQAGQVLPAQYDAFSRLKETAAR

QPEPVRGIMLDLVSSGSTMTTRESGALLNRGAAGATKMVCDQGFTGRYPMRRGAQADAGVEDFERLFSAQ

GLMATYFRDHLAAYVDTSAKPWQALRSNGGPNGMVSQSVLNSYETAERIRGAMLDDSGHLRVSTVLRFID

MDSQLSEAQLSVAGQTVRFAHGVTSPHRVDWTNQNTQLAIKLQLKSVDGRMTTLQFDGPWALFRFFDAGQ

AVGGTGTADRRERLYQTSLGTVRIEWQALTLPSPIWSGILQSFRCPS

>WP_004524806.1 type VI secretion system membrane subunit TssM [Burkholderia pseudomallei]

MSHAVARIVRPLPSRDIWTFAGLVVLACFVWLAGPLFAFAEFRPFESGAVRAATIVALFVAWGARIAWRG

WRAGQLNAQLLNQLREAAPRPAATGDPAQAQLDELRSRFDEAATLLKKVRFGEADGARKGLPRWLEQMSR

QYLYQLPWYVFIGAPGSGKTTALVNSGLSFPLAEQFGRAAIRGVGGTRHCDWWFTNDAVLIDTAGRYTTH

ESNRALDEAEWKGFVDLLKKYRARQPLNGAMLTISVADLLGASEAERTQHAMVLRKRLLELRAQLGIRFP

VYLLVTKADLLAGFAEYFGGFGRAECAQVWGFTFPLAESEAPGFELRAAFDREYRLLHQRLNDGLPELLA

SQTDARQREMTYLLPQQIADLQDMLGQFVAEVFSVSSFEPMPMLRGVYLTSGTQEGTAFDRVMSGIKRFL

KIEGVPPAAQTGSSGRSFFLKSLLQDHIFREAALAGSNLRWHQRQRALQIVGYAAIALLCVAVLFAWLRS

YSRNRDYLDEVAARVPAVDAQIGRAKFTGAADIVQLLPVLDELSGLPNAGGVDLRHPPLAYRWGLFQGEK

IEEASDAVYRRALDDVLLPIAASRMEQALRDARPDEVEYAYAALKAYLMLYDSAHYDPAFVQAVVDLEME

RALPADFSSAQRSALRAHLGALFGNRVAVSPFPMNERLVADVRERLRQVPFSQRLYRQLARTLHASTASY

DFSVARAVGPDASLVFRRQSGKSLADGVPGLYTRSGYRNVFAPRLPGAIDSYGREEVWVLNLGASEIPNP

ADAAAWARDIRQLYLNDYIKTWDDYLADIRLQRTSTLAQSIQVARTLSSADSPLTRLMVALARDTPLGDA

PGGARNLASRAQDKVDEARNSLAQIFAGQPGGEAGAAAAPPASPEQIVDSHFAGLRAFAPGGGDQAASFD

AVLKAIDALYTYLTATDDALRSGAAPPPSDAPARLRAQAGRLPTPVREVLDDLSNVANGSIASVEQRNVA

QRAGANVGDFCRQAIAGRYPFARGAARDVAPSDFAQLFAAGGLMDDFFQKNLQTLVDTTAHPWRFNNRNA

EADPSAAAMLGSFEKAAVIRDVYFGGGARTAQIKVEIVPLEMDPSISEMLLDVDGQIVRYAHGPQVPTAV

QWPGTRGSNQVRLQVTEQSGATGGFTTEGPWALHRLFDRAGVSGGRGPEQMVARFAVDGKPIVLQVTASS

VRNPFRLPQMESFTCPPKQ

>WP_004194034.1 MULTISPECIES: molecular chaperone DnaK [Burkholderia]

MGKIIGIDLGTTNSCVAIMEGNQVKVIENSEGARTTPSIIAYMDDNEVLVGAPAKRQSVTNPKNTLFAVK

RLIGRRFEEKEVQKDIGLMPYAIIKADNGDAWVEAHGEKLAPPQVSAEVLRKMKKTAEDYLGEPVTEAVI

TVPAYFNDSQRQATKDAGRIAGLEVKRIINEPTAAALAFGLDKAEKGDRKIAVYDLGGGTFDVSIIEIAD

VDGEMQFEVLSTNGDTFLGGEDFDQRIIDYIIGEFKKEQGVDLSKDVLALQRLKEAAEKAKIELSSSQQT

EINLPYITADASGPKHLNLKVTRAKLEALVEDLVERTIEPCRTAIKDAGVKVSDIDDVILVGGQTRMPKV

QEKVKEFFGKEPRRDVNPDEAVAVGAAIQGQVLSGDRKDVLLLDVTPLSLGIETLGGVMTKMINKNTTIP

TKHAQVYSTADDNQGAVTIKVFQGEREMAAGNKLLGEFNLEGIPPAPRGVPQIEVTFDIDANGILHVGAK

DKATGKENKITIKANSGLSEAEIEKMVKDAEANAAEDHKLRELAESRNQGDALVHSTKKALTEYGDKLEA

GEKEKIEAALKELEDVLKNASSDKAAIDAKVEAVATASQKLGEKMYADMQAQQAGAAGAAGAAAEGASAQ

GGAQPADDVVDADFKEVKKD

>WP_004193456.1 MULTISPECIES: ribonuclease HI [Burkholderia]

MTLQTIDIYTDGACKGNPGPGGWGALLRYGAQEKELFGGEPGTTNNRMELTAVIAALEALKRPCKVVVHT

DSQYVQKGISEWIHGWKKKGWVTAAKTPVKNADLWQRLDALVAQHDVEWRWVKGHAGHPENERADALANR

GVESLAQA

>WP_011203825.1 MULTISPECIES: chorismate mutase [pseudomallei group]

MKQSLRASLAAAVLGCIVLSAPRIAAADGDDTALTNLVALASQRLALAEPVAHWKWLNGKPISDPPREAA

LLADVEQRATANGVDPAYARAFFDDQIAASKQVQNALFATWRATHGPEGPAPDLATSTRPQLDRLTQSLI

AALARVAPLRDAPDCPSRLARSVSNWKTLTRYDSGREDALGTALSHVCTAGSTSAVG

>WP_041195265.1 GTPase Era [Burkholderia pseudomallei]

MNASAPTGFRCGMIAIVGRPNVGKSTLMNALVGQKISITSRKAQTTRHRITGIHTLDDAQYIFVDTPGFQ

TRHSTALNRSLNRAVTSTLTSVDAILFVIEAGRFGPDDQKVLDLIPPGTPTLLVANKLDRVSDKDTLYPF

FQKMGGLREFAEIVPLSAKHPEDIQRLMNTIKPYLPEGDAIYGEDDLTDRSSRFLAAEILREKVFRWTGD

ELPYTSTVVIDKFEEEGRLTRVFATILVERDSHKAMVIGKKGAKLKQISTEARQDMEKLFDGPVYLETFV

KVRSGWADNEAGLRAYGYE

>WP_024428574.1 MULTISPECIES: ribokinase [Burkholderia]

MTAAVERAAGGASPARAGRVMVVGSLNMDLVVRSARLPRPGETLAGRSFAQAAGGKGGNQAVAAARLGAQ

VAMLGRVGADAHGAALRAGLVAEGIDCVALSVSATATTGVALIVVDDASQNAIVIVAGSNGEVTPESIVG

HEAAIAAADVLICQLETPAAAVRAALAAGRRLGKAVVLNPAPAAGPLPADWLPLVDYLIPNEIEAAALTG

LPVRDPASAETAARALAAAGARNVIVTLGGQGVLALTADGDARHYPAPRVAPVDTTAAGDTFIGGFAARL

AARDAPHDAIRFAQRAAALSVTRAGAQPSIPTLAELDAFAPGPA

>WP_004528130.1 ribokinase [Burkholderia pseudomallei]

METIAVIGSNMVDLVTYVTRMPADGETLEAPNFELGCGGKGANQAVAASKLGARVAMISKLGDDLFAENT

LRNFERFGVDTEHVRRVSGVSSGVAPIFVSPDSRNRILIVKGANRHLRPADIDAAAAKIEASRLVVLQLE

IDIDTVYYAIDFAAARGIPVLLNPAPGVPDLDFARLAKLEFLVPNETELALVSGMPTDTPDAVERAAGSL

VERGVKHVIVTLGEKGSLLVSRAGAVRVPPVAVDARDTTGAGDAYIGCFARHYVATADIPAAMRLASAYA

AHSVTGLGTQKSYADAATFERFLQTIGFGA

>WP_004527915.1 MULTISPECIES: M20 family metallopeptidase [Burkholderia]

MTTAIAAAIDHDRLADFIERKWNDEILHALTDYIAVPAKSPAFDPDWAKHGYIERVIVDAAQWAERQPVK

GLRVEVVRLAGRTPVIFFETPATRAGSTDTILLYGHLDKQPEFDGWRADLGPWTPKFEGGKLYGRGGADD

GYAIYASLAALGALDAQGIGRPRCVGLIETCEESGSYDLLPYVDALRARLGDVSLVVCLDSGAGNYDQLW

LTTSLRGLVSGDLQVEVLEEGVHSGVYGGIAPSSFRVMRQLFERLEDAATGNLLPPSFHCEVPASRLRET

QAAASILGDAVWKGLPWACGQDGKPVLPTTADPREALLNSTWRPSLSVTGAQGLPALENAGNVLRPRTAF

KLSLRLPPLVDAAQAVQQLKALLELDPPYNAKVTFKPDAGAATGWNAPDVAPWLGAALDDASRRHFGANC

AYMGLGGTIPLMNVLQEGFPCAQFMVCGVLGPKSNAHGPNEFLHVPYAKKLTAAVADVIAAAH

>WP_004522669.1 MULTISPECIES: efflux RND transporter permease BpeB [Burkholderia]

MAKFFIDRPIFAWVIAIILMLAGVAAIFTLPIAQYPTIAPPSIQITANYPGASAKTVEDTVTQVIEQQMS

GLDNFLYMSSTSDDSGNATITITFAPGTNPDIAQVQVQNKLSLATPILPQVVQQLGLSVTKSSSSFLLVL

AFNSEDGSMNKYDLANYVASHVKDPISRINGVGTVTLFGSQYAMRIWLDPTKLTNYGLTPVDVTSAISAQ

NVQIAGGQLGGTPAVPGTVLQATITEATLLQTPEQFGNILLKVNQDGSQVRLKDVAQIGLGGETYNFDTK

YNGQPTAALGIQLATNANALATAKAVRAKIDEMSAYFPHGLVVKYPYDTTPFVRLSIEEVVKTLLEGIVL

VFLVMYLFLQNLRATIIPTIAVPVVLLGTFAIMSMVGFSINVLSMFGLVLAIGLLVDDAIVVVENVERVM

AEEGLPPKEATRKAMGQITGALVGVALVLSAVFVPVAFSGGSVGAIYRQFSLTIVSAMVLSVLVALILTP

ALCATILKPIPQGHHEEKKGFFGWFNRTFNSSRDKYHVGVHHVIKRSGRWLIIYLAVIVAVGLLFVRLPK

SFLPDEDQGLMFVIVQTPSGSTQETTARTLANISDYLLTQEKDIVESAFTVNGFSFAGRGQNSGLVFVKL

KDYSQRQSSDQKVQALIGRMFGRYAGYKDALVIPFNPPSIPELGTAAGFDFELTDNAGLGHDALMAARNQ

LLGMAAKDPTLRGVRPNGLNDTPQYKVDIDREKANALGVTADAIDQTFSIAWASKYVNNFLDTDGRIKKV

YVQSDAPFRMTPEDMNIWYVRNGSGGMVPFSAFATGHWTYGSPKLERYNGISAMEIQGQAAPGKSTGQAM

TAMETLAKKLPTGIGYSWTGLSFQEIQSGSQAPILYAISILVVFLCLAALYESWSIPFSVIMVVPLGVIG

ALLAATLRGLENDVFFQVGLLTTVGLSAKNAILIVEFARELQQTEKMGPIEAALEAARLRLRPILMTSLA

FILGVMPLAISNGAGSASQHAIGTGVIGGMITATFLAIFMIPMFFVKVRAVFSGEKEDADEALRLAHEHM

HRDDKPEHGDDAGKKD

>WP_004197879.1 MULTISPECIES: adenosine-specific kinase [pseudomallei group]

MLQLLSVAIDKPDTANFILGQTHFIKSVEDIHEALVGAVPGIRFGLAFCEASGKRLVRHSGTGAALTELA

CRNALAIGAGHCFLVFLGDGFYPLNVLNAIKAVPEVCRIFCATANPTEVVVAQSDQGRSILGVVDGFSPL

GIETDEDVRWRKDLLRNIGYKA

>WP_004194315.1 MULTISPECIES: D-sedoheptulose 7-phosphate isomerase [pseudomallei group]

MENRELTYITNSIAEAQRVMAAMLADERLLATVQKVADACIASIAQGGKVLLAGNGGSAADAQHIAGEFV

SRFAFDRPGLPAVALTTDTSILTAIGNDYGYEKLFSRQVQALGNKGDVLIGYSTSGKSPNILAAFREAKA

KGMTCVGFTGNRGGEMRELCDLLLEVPSADTPKIQEGHLVLGHIVCGLVEHSIFGKQ

>WP_004190112.1 MULTISPECIES: thiol:disulfide interchange protein DsbA [Burkholderia]

MKKLLSSLFLSLSLVAGFAQASPSAPVAGKDFEVMKSPQPVSAPAGKVEVIEFFWYGCPHCYEFEPTIEA

WVKKQGDKIAFKRVPVAFRDDFVPHSKLFYALAALGVSEKVTPAVFNAIHKEKNYLLTPQAQADFLATQG

VDKKKFLDAYNSFSVQGQVKQSAELLKNYNIDGVPTIVVQGKYKTGPAYTNSLEGTAQVLDFLVKQVQDK

KL

>WP_004521905.1 MULTISPECIES: translation initiation factor IF-1 [Burkholderiales]

MAKDDVIQMQGEVIENLPNATFRVKLENGHVVLGHISGKMRMHYIRILPGDKVTVELTPYDLSRARIVFR

AK

>WP_004198824.1 MULTISPECIES: 50S ribosomal protein L34 [Burkholderiaceae]

MKRTYQPSVTRRKRTHGFRVRMKTAGGRKVINARRAKGRKRLAI

>WP_004189550.1 MULTISPECIES: rod shape-determining protein [Burkholderiaceae]

MFGFLRSYFSNDLAIDLGTANTLIYMRGKGIVLDEPSVVSIRQEGGPNGKKTIQAVGKEAKQMLGKVPGN

IEAIRPMKDGVIADFTVTEQMIKQFIKTAHESRMFSPSPRIIICVPCGSTQVERRAIKEAAHGAGASQVY

LIEEPMAAAIGAGLPVSEATGSMVVDIGGGTTEVGVISLGGIVYKGSVRVGGDKFDEAIVNYIRRNYGML

IGEQTAEAIKKEIGSAFPGSEVKEMEVKGRNLSEGIPRSFTISSNEILEALTDPLNQIVSSVKIALEQTP

PELGADIAERGMMLTGGGALLRDLDRLLAEETGLPVLVAEDPLTCVVRGSGMALERMDKLGSIFSYE

>WP_038725275.1 YXWGXW repeat-containing protein [Burkholderia pseudomallei]

MDTLSSRVQSFMEAMMSFSLHRVVLPLVAAGAGVCALAAPLAASADEIVVEQAVPGVVVAPAMREVVVVA

PSAPPPVRYEVVPEPRVGYVWDRGHWRWDHDRYVWIAGHWEVERIGMHWAPGHWSQRGPGWVWVRGHWA

>WP_024428841.1 tetratricopeptide repeat protein [Burkholderia pseudomallei]

MESAFDRAFAAHRAGRLDDAEHGYRAALAANPADADALHLFGVLRHQQGRHEEAADLVGRAVGLRPNDAA

LQLNLGNALKALGRLDDAIERFRNALTLAPAFPLAHYNLGNAYAAQERHDDAVDAFKRALALTPGDASIH

NNLGNALNALGRHDDALEAFRRALELRPGHAGAHNNLGMALAALGDTDAAIAHFRAAIAAEPHFVAAHFN

LGNALDAIGQHAQAQHAFEAALALQPRFALALFGLANTLAARGRHRDALPHYERAVGLDPSFVLAWLNLG

TAHHALGAHEMALRAFDQALRLDPSFTLAQMHRAVTLLTLRDFARGLPAYEARHALPGAAPLGPLPRWQG

EPIANRTLLVRAEQGFGDTLQFVRLVPLARARCARLILQVQPALLPLIAPMAARWRVSVVSADAARTPAA

DLVCPLLSLPFALGLEYDAIPSRTPYLDVPDAARRRFRGSLGGQAKRKFGIAWSGSAPVQDNRALPLDAL

APLFALAGIDWIVLQPTLSDSEHAALDAHPDAARIHRLDGLTDFAATAALVDRLDGVVAIDTAVAHLAGA

LGKPLWLMLPVAADWRWSTGDDSAWYPRARLVRQSEPGRWDDVVATVAGAIAHG

>WP_024428704.1 MULTISPECIES: VRR-NUC domain-containing protein [pseudomallei group]

MPPDPPSPPAFYYLSNFERALAWLVERYDDVLDAEEHAFVAAFGALPRASRALLVRMLMRKGPMFRASKL

VYDEIGCPFAAAAPLVALGWIDPQPMLALDALFALATKAELRDAFSDAPASGALRKADWLDALRARHDGE

RPWAQWLPSIDDRVLRVTVDALCNRLRLMFFGNLHQDWSEFVLADLGLLQYEAVAFAPSSRAFQRRGDVD

AYLQLHACREQLDAWPDDAPLAPLVDAAAAVDCGNAWLAMRRAKLTYAIGRACERRADWGGALDAYASSA

WPGSRQRRVRVLERCERFDAALALADEAAREPENEAQAQQIARMLPRLRRRAGLPTARAPRAQEIPRGCV

ELARPGVPYPVEYVARDHLSRADAPVFYVENALVNSLFGLLCWEPVFAAVPGAFFHPFQRGPADLHAPDF

RARRAAQFDACLAQLDGAQYRDTIRRHYAQKRGVQSPFVFWAALDETLLEHALACLPAEHLRLWFERLLD

DVRGNRSGLPDLVRFWPAERRYELIEVKGPGDRLQDNQIRWLDYCVRHRMPVRVLDVRWTGDARASSQGE

EALA

>WP_004544598.1 helix-turn-helix domain-containing protein [Burkholderia pseudomallei]

MTRLAAPSLSLRRYGAVQASDVHDFHQIVLGVDGAMTMTVDGVDERIDRRCAWLIPAGARHDYAGLADNS

QLVLDLPPGSLAVPERLFERARTMAIDPGLTTLVGELASRIARGPLADAASARRLHWQAAARLCGALVGE

LGGAPSQPAVGLDFARIDGWLRAHLAQPLRIADLAAHCGFGMRRFHQLFCEAFGETPHRYLQRLRLDAAV

MLLGDPKHSLTDIASEVGFADQSAFTHAFTKRFGLAPGRWRSDRH

>WP_004535030.1 OmpA family protein [Burkholderia pseudomallei]

MTKPRLPLLRARRFALALAALALGGAARADNVGPATVTPIDAGGVPVKSVPLAAPVTAAPVAATAGAGAT

VSSPPPANATPGQVVAGGKVADEATKAAVLQRLRDTYGAANVVDQIEIGNVATPPNWSANVQKLIGPQLK

QISKGQLKIDGTQIDVKGEVHNEAQRQQLASDMANALNPTYTIKNGLRVSASEQGLLDQTLANRTIEFET

GSATLTPQGRAILDQMAGALAKMSNRTVEIIGHTDNSGNRTSNIALSQARADAVKGYLVTKGIASQQLTT

TGVGPDQPIAPNDSADGRARNRRIEFRAGQ

>WP_004525043.1 MULTISPECIES: aldehyde dehydrogenase family protein [Burkholderia]

MLKETYPYYLANEAVYANTGLEVTDKYSGKVATRVALADARAIDAAIAAAVAAQRPLRALPAFRRQAILE

HCVARFRERFDELAEALCIEAGKPINDSKGEVTRLIDTFRVAAEESVRIEGGLVNLEISPRAQGYSGYYR

RVPIGPCSFISPFNFPLNLAAHKVAPALAAGCPFVLKPASRTPIGALIIGEVLAQTDLPKGAFSILPAHR

DGADLFTTDERFKLLSFTGSPAVGWELKKRAGKKKVVLELGGNAAAIVDADQRDRLDYVVDRLAFGAYYQ

SGQSCIGVQRIIAHADLYDALREKLIAKTRSLKMGDPKDPATFVGPMISEAEARRLAGWMDAAVAAGARI

IAGGNVDGAMFEATLLEGVGRDQDLYRKEAFGPVALLERFADFDDALARVNDSDFGLQAGVFTDSLSHAQ

RAWDELEVGGVVINDVPSFRVDNMPYGGVKDSGLGREGIRYAIEDMTELRLMVVRQR

>WP_004200038.1 MULTISPECIES: alpha/beta fold hydrolase [Burkholderia]

MPFVTIDGQPLHYQIRGAGAPVLFGHSYLWDSSMWEPQLDALSKSYRVIAPDLWGHGRSGPLPDGTRSLD

DLARQMSELLDHLDIDTCSIVGLSVGGMWAVPLAHRAPQRIDRLVLMDTYVGVEPDATRNQYFQMLEAID

AQGAIPAPLLDAIVPIFFRPGIDPASELPTGFRRALQAFTTERLRDSVIPLGKITFGREDARAQLSALPA

DRTLVMCGANDVARPPEEADEIAALIGCEKAFVPNAGHISNLENPAFVTQALSDWLGRGAARA

>WP_004194969.1 MULTISPECIES: alpha/beta fold hydrolase [Burkholderia]

MHTPDLFVQSSNVRLAVYTWGDKPSADKPRDIVVLAHGFPDRALFWEQVAAALQRDFYVVAYDMRGCANS

THIKGARHYRFALLLADLYAVIDAVSAGRPVHLVGHDWGGVYGWDAIADPEGARRIASLTTLSPSLDQIG

FYLRRRLLRPTPRHLAQLVGQLMRNSLMTFFTAPLLPELLFASGLAMAMFRRIIAHYEPRITFRKNDGME

GDAIRYLGIYRANLLQRVLRPRKRVSTTPVHALMAIHDPFLPPALFEGCREFTTRYSESTVDAAHWAPLS

RPQEIAETVGAFVRQASRRPDVALQSAS

>WP_004192269.1 MULTISPECIES: nitrous oxide reductase accessory protein NosL [pseudomallei group]

MKRRFLSAAVRTSIAALAAAALVAACGHDAQTPPPAREITDATVSVLDGMSLKDYPGPKAQIVYADGEPD

FFCDTLGLFSVYLRPEHDRKVRALYVQDMGATDWQHPVGHWIDAKRAIYVIGSKKPGAMGRTFASFAREA

DAARFAKAEGGKLYRFGEITPEMAATDGGVVKDQTM

>WP_122837186.1 Kdo hydroxylase family protein [Burkholderia pseudomallei]

MSESQIIEIASADWSGQQLSVPREQLLAGLEDGKVLFFAHLRFAIEGGEEALLDPALADPKRKNISLAPN

GGALAGVAGDAVTQSAVRALIARYQQQAGALVDGLFPEYRGKLRVAPTSLRLMQVETRETSWRKDDSRLH

VDAFPSRPNYGERILRVFTNVNPAGVPRVWRVGEPFEDVAKRFLPHIKPQVPGVAWLLELLHVTKSRRSA

YDHLMLKLHDSMKADLDYQKNSPQQTMPFPSGSVWVCFSDQASHAVMSGQFMLEQTFFLPVGAMARPQRA

PLGILERLQGRALV

>WP_023358579.1 glycoside hydrolase family 68 protein [Burkholderia pseudomallei]

MTYSKQAVSRRHKRLALSAAALAAAACMSAHAQSDGAGPAPTPHTQQAYDPESHFTMRWTRADMRQLVKQ

SHTAGADKNSLPPALTMPDIAQNFPLVDSNVWVWDTWPLADMRANQLSYKGWEVIFSLSADPHAGYTFDD

RHVHARIGFFYRRAGIPASQRPANGGWTWGGHLFPDGASAKVFGTAPMTNNAEWSGSARLTHGENVSLYY

TALSFNRSAPGGADITPPIAIITRADGHIHADDKHVWFSGFDDHKALLQPEGKMYQTGQQNTYYSFRDPF

VFTDPAHPGNTYMVFEGNTGGPRGARTCTEADLGYAPNDPYREDLNAVMNLGAVYQKANVGLAIATNPQL

TEWKFLPPLLSANCVDDQTERPQIYLKDGKYYLFTISHRTTMAAGIDGPDGVYGFVGNGIRSDFLPLNGG

SGLVLGNPTDFSAPAGAPYAQDPNQNPRAFQSYSHYVMPGGRVESFIDAIGARRGGTLAPTVKIDIHGDS

TTVDRAYGAGGLGGYGDIPANLPAVGAGHHD

>WP_004552130.1 MULTISPECIES: ferritin-like protein [Burkholderia]

MNPNTVPPAARDVAWIKHALQTAIELEYSTLPLYLSAMFSLEVQNYTAYNAIRSIAMEEMVHMAIAANLL

AALGGSPQFKPIQIAYPTTGLPGGAEPDLHVGLARYSKPQLKNFLRIETPGFLLRQLGRDEGYPTIAAFY

ESIRSAIARNANAVRAAVQAGGPANQVGDDIGFTTVTYKPGVDPVESFDAGIGEILQQGEGSGRGDVFAG

NPFEDEESHYARFAELYYGARYQAPSPPVDFNPKNEPLFFGGPAIAAPVVVNTLAVPRDGYARILALDPE

RDTVESDLNAFDSGFSKILSTLDAVWNGPSSTSWKTLGAAVHGMVDLRVLSCFNIMRHAVPAAAVSQLAE

LYPDEIGFLRTYTDLSKPVFYGPRFVNVNGKQAS

>WP_004550623.1 YcgJ family protein [Burkholderia pseudomallei]

MKLSVLFMAALPLIAIAASAHAQPRHPAVYSPAAGVLCDRYVCADDQGISRALTERYLGKRVAAKAFSQG

DFDPTEFTFANGVFCDVKERLCRDDRYYGADGKRSGAVSRRYTELLFGRRSGG

>WP_004530470.1 GMC family oxidoreductase N-terminal domain-containing protein [Burkholderia pseudomallei]

MTTERTLEGEFDYVIVGAGTAGCVLANRLTEDPDVTVLLLEAGGRDDYHWIHIPVGYLYCIGNPRTDWLY

KTEPEAGLNGRALSYPRGRVLGGSSSINGMIYMRGQRGDYDDWARATGDAGWSWDSVLPVFRRSEDHHAG

ATDMHGAGGMWRVEKQRLRWEILEAFSQAAQQTGIPATDDFNRGDNTGVGYFEVNQKRGIRWNASKAFLR

PALARPNLTVITGAQAERLVFDGKRCAGVEYRGGGAPFVARARVEVLVASGAVNSPQLLELSGIGDGSRL

QALGIGVVADLRGVGENLQDHLQLRMAFRVRGVRTLNTLSAHWWGKLWIGAQYALMQRGPMSMAPSQLGA

FAKSDPNDPALAQPDLEYHVQPLSLERFGEPLHRFNAFTASVCHLRPTSRGSVHAASPDPARAPSIAPNY

LSTDYDRHVAANALRLTRRIASAPALARYAPEEILPGARYVSEAELIAAAGAVGTTIFHPVGTCRMGRAD

DPDAVVDSRLRVRGVTGLRVVDASVMPTITSGNTNSPTLMIAERASDMIRADRRGASERGASARAEAVLP

T

>WP_004526298.1 helix-turn-helix domain-containing protein [Burkholderia pseudomallei]

MTIRLKLLRKQKGWTLDVLAEATGLTKSYLSKVERGLSVPSIAVALKLSKALQVDVEQLFSEGRDRELIT

VTRASERTSMGRASSERVRTYESIAAGVAPKKLLPFIVHPPRDYVSSAFREHEGEEMLFVHRGSVEIEFP

NETIKLKTGDSVYFNALIPHRMRSVGATSAEVLVVVSNDEGVQEDAQ

>WP_004524995.1 MULTISPECIES: HlyD family efflux transporter periplasmic adaptor subunit [Burkholderia]

MIQVTRKQIVAAAAVVVVALGAYYGWTLLRHQGPGDGFASGNGRIEATEIDVATKLPGRIDAILVDEGDF

VKAGQPLANMQIQVLRAQYDEATAQRQRALNTAAGVQSQVAQRKSDKAAAQAMVVLRESELDAAERRLAR

SQTLSREGASSLQELDDDRARARSAQAAVSAAAAQVAAAQAAIEATEAQLVAAHSAVTAADATVARVQAD

IDDSQLASPRDGRVQYRVAQPGEVLPAGGKVLNLVDLSDVYMTFFLPETVVGRVALGADARIILDAAPNY

VIPATVSFVSSTAQFTPKTVETANERQKLMFRVKARIDRELLQKHLKLVKTGLPGVAWVRVDPSKPWPAQ

LMVKVPQ

>WP_004524888.1 MULTISPECIES: TetR family transcriptional regulator [Burkholderia]

MSAIPQAARKRGRPVKGESATLRDELILKSAKLFRTQGYERTTVRDIAAAAGVQAGSWFYYFKTKQDILV

AVMEQGMSNALARIEALDVEHLPARDAFRALVHTHLHTLVSPDHDFIPVLLYEWKSLDEAMQAKVLKLKD

RYEAVWDGVIERLQAAGEWPAPTPIDRLLMFGALNWVAQWYKPDGALGLDALAEHAVRFLLRTAETPAPA

PAGTAKRRRGVKAS

>WP_004523134.1 MULTISPECIES: substrate-binding domain-containing protein [Burkholderia]

MKVNLKALSEALGLSRTTVSRALNGYDDVSEATRERVMQAAREFGYVADPTARRLATGRADAVGIVYPFG

AGDLGDPRFAEVVAGVTERLGESGLDFFIVSARPNAELDTYRRLVDGRLVDGLIVARTLVDDPRLRFLQE

REFPFVAYGRTASAKPYTWFDFDNEAGMHAAAQRLIAFGHRRIALVCAPQTLNFAAQRRAGFERALREAG

IEPDAALIVECAFTRDGGYGAAQTLLALERAPSAIVVDNNIAGAGVFRAIVERGRRFVRDVSLIVYDGVS

PDVSFPHHASAVLQPTGHASGRAIAELMLGAIAHPGDAAHRLVSPVIEPGDTDGPFSG

>WP_004523127.1 MULTISPECIES: TetR family transcriptional regulator [Burkholderia]

MARTRAPDHESQREQILDLAAAKFAQTSYPSTSMTDLANASGTSKARLYHYYEGKEAILFDLLDRYTKRL

MLIIAEVEGASQRRGLTEREAFAELVRAFLAEYETSHSRHVALLNDVKYLEDTQRQIVLDRQRDIVAAFA

RQLARAYPERISKDNQTPVTMMVFGMINWTFTWLKPGGRLGYRDFAEQVIGMIEHGLGG

>WP_004521491.1 MULTISPECIES: putative DNA-binding domain-containing protein [Burkholderia]

MRSDAAASDYACAFAPGLTNPSIGAPDDAIAHGHKRVARRYDVYRNNVTVSLIEALAAIYPAVQRIVGVE

FFRAMARFHVRETLPVSPLLFEYGRDFPDFIAGYEYARTMPWLADTARIERAWLDAYHAADLSTLTADAF

TAIPPDALAAVRLVPHPATRIVRSPYPAVSIFAMNRSDGPVTSLHSAEAEDALVTRPAHDVIVSRLPLGG

AVFLISLIDGASLGDAVAITLEDNPAFDLPASLRGMIEAGVFTAIHSGA

>WP_004200927.1 MULTISPECIES: cupin domain-containing protein [Burkholderia]

MDSDIMHIGGRIRRLRRELKKTLLEVATEANLSVGFLSQVERNLTGISISSLVNVAKALRAPLSALIDQP

RQDQPDSHEGSRESYAIRPTQQRYERLSTTFAGSLLNAVKVSMMEGYSSEWVAHAGDEFVFVLSGHVRYT

VGKQDYPLGPGDSLHFDAHERHRVVNVGHGPVELIAVGTLPLFGDGQPAFPSLPGERKPPRTRAARKRIE

PPPTPSAQQDARHDEAAAPNAVTTHDGAPRAAKKNTR

>WP_004198976.1 MULTISPECIES: GMC family oxidoreductase N-terminal domain-containing protein [Burkholderia]

MQYDYIIVGGGSGGASLAGRLADACPDATIALIEAGGHTERNLLVNMPVGIAALVPFKLGTNYGYETVPQ

PGLGGRRGYQPRGRGLGGSSAINAMIYTRGHPLDYDEWEQLGCTGWGWRDVLPYFRRAEGNARGANEWHG

ADGPLTVSDLRFRNPFSERFIAAAHEAGYPLNDDFNGEHQEGVGFYQVTHRDGSRCSVARAYVYGRTRPN

LHVIVDATVLRVVFDGKRATGVEFARAGRTEQLAARAEVILSAGAFNTPQLLMCSGVGPAAQLRRHGVAL

VHDAPDVGENLIDHIDFIINKRVNSSELVGICMRGIAKMTPALFSYLSGRRGMMTSNVAEAGGFIKSEPG

LDRPDLQLHFCTALVDDHNRNMHWGFGYSLHVCALRPKSRGNVALASGDARVAPLIDPRFFSDERDLDLL

VTGAKAMRRILCAAPLASQGGRELYTDPGDTDAQLRAAIVAHADTIYHPVGTCRMGTDARAVVDPQLRVK

GVDGLRVVDASVMPTLIGGNTNAPTVMIAERAADFIVAARNGQAAPTRERVAATHGG

>WP_004196954.1 MULTISPECIES: bacteriohopanetetrol glucosamine biosynthesis glycosyltransferase HpnI [pseudomallei group]

MTESLSIVGWVLLALVCASCGYAVLAACAPAPRVPRAAARDGFEPVSVLKPLCGSEPHLYENLATFCEQR

HPRYQLLFGVASAADPAIAVVRRLQADYPDCDIELVIDARVYGSNLKVSNLVNLAERARHGRIVIADSDI

AVEPDYLTRVTAPLADPSVGVVTCLYHARSVGGFWTRIGAQFVDAWFAPSVRITHLGGSSRFGFGATLAL

TRATLDAIGGFKALKDELADDYWLAELPRRLGLRTVLSEVNVATDVAEPSFAPLWLRETRWLRTIRSLNP

AGFAFLFITFTAPWLVIGAALAAWLGPASAAGATAAWAAAIGTLARLALHARGAAGWRAFWRDLPLVPVR

DALLALEWLAAAFGTQVVWRGARMTVVGGDARATVVEAGDGR

>WP_004185974.1 MULTISPECIES: TetR family transcriptional regulator [Burkholderia]

MNQPKIKRDPEGTRRRILLAAAEEFATGGLFGARVDQIARRAETNERMLYYYFGSKEQLFTAVLEYAFSA

LMEAERAIDLEGVAPVEAITRLAHFVWDYYRDHPDLLRLLNNENLHEARYLQKSTRIREMISPIVKTLDG

VLERGQKAGLFRTDIDSLRFYVTLSGLGYYMVSNRFTLAAIFGRDFSAQHERAEMVKMNTELLLAFLLRR

>WP_004551611.1 MULTISPECIES: PenI family class A extended-spectrum beta-lactamase [pseudomallei group]

MNHSPLRRSLLVAAISTPLIGACAPLRGQAKNVAAAERQLRELESTFDGRLGFVALDTATGARIAHRGDE

RFPFCSTSKMMLCAAVLARSAGEPALLQRRIAYAKGDLIRYSPITEQHVGAGMSVAELCAATLQYSDNTA

ANLLIALLGGPQTVTAYARSIGDATFRLDRREPELNTALPGDERDTTTPAAMAASVHRLLVGDALGAAQR

AQLNAWMLGNKTGDARIRAGVPADWRVADKTGTGDYGTANDIGVAYPPNRAPIVFIVYTTMRNPNAQARD

DVIASATRIAARAFA

>WP_004196953.1 MULTISPECIES: hopanoid biosynthesis associated radical SAM protein HpnJ [Burkholderia]

MKTLFLQAPSYDGFDGGAGSRYQAKREIRSFWYPTWLAQPAALVPGSRVVDAPADGLSVEDTLKIAKDYD

LVIIHTSTPSFPTDAMFAEDLKKMKPSMLVGMVGAKVAVDPHNSLTATQAIDFVCREEFDYTCKDIAEGK

PFAEILGMSYRAKDGSIEHNGPRPMIENMDELPFVAPVYKRDLKIDNYFIGYLNYPYVSIYTGRGCRSKC

TFCLWPQTVGGHRYRVRSVESVLAEVKWIRDNMPEVKEIMFDDDTFTDFKPRVEEIARGLGKLGVTWSCN

AKANVPYSTLKIMKENGLRLLLVGYESGDDQILLNIKKGLRTDIARRFNEDCKKLGIKIHGTFILGLPGE

TKETIKKTIEYAKEINPHTIQVSLAAPYPGTRLYNQAIENGWMEENKTINLVSKEGVQLAAIGYPHLPKE

EIYHQLEHFYREFYFRPSKIWEILREMLTSWDMMKRRLREGVEFFRFLRAHEA

>WP_004199900.1 MULTISPECIES: twin-arginine translocase subunit TatC [Burkholderia]

MSDPQHNPDDGPEETFISHLVELRDRIIRAGAAVIVVFLGLVYWAPDIFRLLARPLMENLPKGGRMIVTD

VTGSFFVPMKVTMLVALVIALPVVLYQIWAFVAPGLYQHEKKLVAPLVGSSYVLFLCGMAFAYFLVFPTI

FRVMAHYNAPLGAEMSTDIDNYLSFVLGMFVAFGVTFEVPIVVVLLVRMGVLTVQKLKEIRPYVIVGAFV

VAAVVTPPDVFSQLMLALPLVLLYEAGIIAARLFVKPPAKEEDESKAAAN

>WP_164977635.1 CoA transferase [Burkholderia pseudomallei]

MGALGALGALGASGASGASGAAGAAGAAGAAGAAGAAGAAGAAGASDRRRSDRSRDNRARIALRCGGRRS

TEHAPARTRRERRAHPKSATNRGDRSKMGALSHIRVLDLSRVLAGPWCAQTLADLGADVIKVERPECGDD

TRHWGPPYLKTPDGADTREAAYYLAANRNKRSVTVDIATPEGQRIIRELAAQCDVVLENYKVGQLAKYGL

DYASLAAVKPGLVYCSVTGFGQTGPYAHRAGYDFIIQGMGGFMSITGERDGLPGGGPQKAGVAIADLATG

LYSTIAILAALAHRDRTGEGQHIDMALLDVQVALLANMNTNFLASGKPPVRWGNAHPNIVPYQTFETSDG

WIIVAVGNDGQFRKFVEAGGRPELADDERFATNPARVRHRDTLVPIVAAMTKTRTKREWLDALEALGVPC

GPINDLAEVFDDEQVRARGMQVDLPHPSGASAKLVRNPIRMSATPPDARSAPPLLGEHTDAVLRDMLGYG

DAAIAALRDKRVV

>WP_152768983.1 GPW/gp25 family protein [Burkholderia pseudomallei]

MWPRATTLGNTHVSDLQLYNKLSKRIRRHSLQEVVADHLVDLMNHAIRGARMRIADDSPAAHSVLNFGCP

PMQMAGATKINPVHAAAHICEVIRRFEPRVDPASTVVKPRTESRKRLAQTIYFDVSMKAREDGAELRASL

ALDYLSGYFSLADDR

>WP_152768791.1 gamma-glutamylcyclotransferase [Burkholderia pseudomallei]

MILAAPPAVSRPAPARRAYPPSLGEARYLSDAELAASLGATLERWDRTSDLWLFGYGSLIWNPGMPAVEA

LRAKVHGYHRGLYLWSRVNRGTPEQPGLVLALDRGGSCTGLAFRLAGRTAMPHLEALWRREMAMGSYRPA

WLPCALAGGERVNALAFVMRRDVPTYTGKLTDDVVKAVFGCASGRYGTTLDYVSRTVEALRDSGMPDRAL

EALLARCR

>WP_144397651.1 polysaccharide biosynthesis/export family protein [Burkholderia pseudomallei]

MKPFVRRLRPEPAGTRKSIEVWMGSLGEAGGIAPPKRTCGARVRRLMKRVTLCAAFAALSACGVAPGMRM

KQPANVPVSSAAADAPAEAGRKPRGEQLRVPITDIDLSLIRTLRDAQQAPRRAADLVSPASGYTIGRGDV

LQITVWDHPELAAALGTQQQTAARAADAPAGFVVDQDGTLQYPYVGRIAVVGLKPEQVQARLARQLAQTF

RDPQVTVRIASFRAKQVYIEGEVHTPGSQALNDIPMTLYDAVSRAGGFSASADQRRVTLVRDGVERRIDL

SGAAQGVNPSRIVLRDGDLLRIPPRDESGVFVMGEVNRPVTALPMRNGRLTLSEALSQAGSLNATTADAA

QLYVIRGSLDAKPHVYRLDASSPVAMVLANQFELEPKDIVYVDGNGLVRFSRVLSLLLPAVNAGLTAAVV

TK

>WP_122890358.1 methyltransferase regulatory domain-containing protein [Burkholderia pseudomallei]

MRRPPEPERTLRRAWVGNRVVFFILMQASDPSSGYLSDVTFPDRFHRELSPTWLNYASVLGGARPKELGR

PFRYLDLGCGFAHSTVINAAAFPHAEFHACDFNPAHIEAAARRASRLGIGNVAFHEASFDALLDRDLPPF

DFIVMHGIYSWVDAGMRRVIRQLLSRRLADAGLVYLSYNCQPGWAAEAPLRKLMLELAQAADGGIEARTG

SAIAGMRKLGTPSLRYFRDNPAAAEALAALANDPLDYLAHEFLNGTWKIHYSVDVVDEMAEAGLAYAGSA

TLADNHPMLLIDRQAADAIAALPNARLRHLAEDFAVNRRFRRDVFVRGARASTAPAEALRHLDEIAIGCT

TEIDRIDTRVTIPRGAISFQPDFIADLRALLRHGAMRIGEIVARLGAARRNSREIRQNLLFLVASGTLTP

FAQPGGPTDTGARRAASPAAAAALAGSVDDAAPAFVPSELLGNGLAVSPDEAAQALRWIAGEAMPRPERL

ARVGVLRGA

>WP_122809055.1 efflux RND transporter permease subunit [Burkholderia pseudomallei]

MSRNLFAVFIRYPVATCLMTAGILFAGVAAYFHLPVAPLPQVEFPTIQVSAVLPGADPVSVASTLAQPLE

TQFSKIPYVTQMTSQSTLSSTSIVLQFSLERSIDAAANDVQSAIDAAAAQLPADLPSPPTFQKVNPADSP

IMLLSAISSTLPLTTIDDYVETRLTKSLSQIDGVGSVSIGGQQKPSIRIQLDPVKLASRGLSSEDVRRAL

SGLSGVNPKGVFNGTTRSYTIYTNGQLTEPAQWNDAIVAYRDGTPLRIRDIGQAVLGPEDNTLAAWIDGR

RAISVGIYKKPGANTVSTVDKIRARLPELEASLPPSLKIAVLADRTQTIRASLLDIELTLLLNVVLVVVV

IYAFLGSVRTTIIPAVTVPVSLFGACALMWVCGYSLDNISLMAMTIAVGFVVDDAIVMVENIARHVEAGE

RPLQAALKGLSETSFTIASISLSLVAVLLPLLLMSGIIGRMFREFAVTLSMTIIVSAFVSLTLTPMMASY

LLRAHRHDAGRPPRPGLFERAFARTAAAYERALDVALRHRFVTLCAFFASVAASVFLYVGIPKGFFPQQD

TGVITGISEAAQTISVEDMARHSMALAAIIRADPAVEHCQMAVGGSAYAGTTVNNGRWYITLKPRDQRDA

TADEVIRRLRPQFAKVPGVRMYLQAAQDVIIGARLARTQYQLTLQSADVGALTTWAPRLLARLSGLPQLR

DVASDQQVNGSALSVAIDRDQAARYGLTPEAIDGTLYDAFGSRQVAQYFTQLSTYKVIMETLPSLQRDPG

TLDRIYMKAPSGALVPLSSVARWTTDTVQPLSVNHQSHFPSVTISFNLAPGVSLGEATAAIEAARASLRM

PPAVVGSFQGTAQAFQSTLATMPMLILSALIVAYLVLGALYGSFIHPWTILSTLPSAGVGAIATLWLFKY

DFNLIALIGVILLIGIVKKNGIMMVDFAIAATRERNMTSLDAIRSACLLRLRPIMMTTMTALFGALPLML

TPGMGSELRQPLGYAMVGGLLVSQVLTLFTTPVIYLYLDTLSNRLKARLRPLGADMPSNAHVEGATQD

>WP_122798662.1 carboxypeptidase regulatory-like domain-containing protein [Burkholderia pseudomallei]

MIHIRLKRASFLLLLGAQAVSLGAAAGTLSGTVSSAGTPLAGAMVTVFDAAQARRDTVYTDHNGRYRITV

DFAGELRVRARTPYFKDAAQDLALAPDASKTLDFSLARQTVADELSASLPASAHLATLPWSSQDSRTAFI

SQCNYCHQVGNALTRTPRDEAAWGATVRRMEGYAALLTDRQARDITHTLYQGMNAHAVAAVEKYLYDDRL

APAKIREWAAGDGLTFIHDADVGFDDHLYGADEGHDKIWELDRKTGRLTEWKEPDVDLPVGGIFSGVQLP

IGVFSGKHGPHSLAQAPDGRFWITNALSSTLASFDPATKRFKLYELGHSHLYPHTLRIDRAGIVWFTIVA

SNEVARFDPKTGRFTIIHLPDGGIWRAMSHYLFPLVVKMAAWFPGQNLHLALTHHKWAFQGRDAFPFPYG

IDVNPVDGSIWYAKLYANKIGRIDPKTLAVTEFDTPLGGPRRLRFDPQGNLWIPAFDDGGLMKFDTRTHR

FETFKLPLLAHNEYEVPYALNVQPKTGDIWITSNMSDRIFRFVPSTQTFITYPLPTRVTWLRDMTFTQDG

AVCSSSSNLPAYGIEGGRASFICLYPDGERGGTAANAAGNANAAANLARVRAAAARRTPERTAPARAALA

RAGSNRDGAIQTAAIQADGTRTDRSAH

>WP_080312945.1 alpha/beta hydrolase [Burkholderia pseudomallei]

MQLRERHMRSCSKSTWPPRLVTAPGLHGSEGAHWQTWLERQFARALRVEQDDWDAPHVERWAQKVRDLLA

RERGPFVIAAHSFGCLATAHALAQAAQTRAADVVGVLFVAPANPRKFAFAGDFDARRLNVPSIVVGSESD

PWMTLADAREFAQRLGSAFVNLGDAGHINTAAGYGPWPRAKYFVDTLVHCAAPLRLRDDDAPASGAPARQ

PLVTAA

>WP_050867939.1 trypsin-like peptidase domain-containing protein [Burkholderia pseudomallei]

MSRQTLGRALIHVAALGAVLAGFACLQPTPLAAGTLQSPAKAKRIAQIGAAGPVDFPTLVERYGPAVVSV

SVPAQDPQMSASGLEALDPDDPFFAYFKSAATQPALSPENGPRAMAGAGSGFIVGADGIILTTAYVVGQA

SEATVRLIDRREFKARVLAVDDSSDVAVLQIDATKLPTVRLGDSSRVRTGEPVLTIGTPDGSANTVTTGI

VSATARMLPDGGRFPFFQTDVTGNLDNSGGPVFNRAGEVIGIDVQIYGSGERNPGVTFAIPIDMAMKVRA

QVLQAQRQARQQAQPPMQQAQQAQQAPPAAAQNALGVDAQDVGPGLAAAFGLPRPAGALVNAVEPGSPAA

AVGLKPGDVIVQIGDRPLGRSAELAGDLAALPPGSSAPITLIRNRMPMTVMLGSGAAASAPTGATASPGN

AAAGRSETGGADRLGLTMHPLTDDERRSTGLPVGMVVDAVRGPAANAGIRPGDVVLELDDTLIETPDMVP

ALEAKAGKVVAVLIQRGSERRFVSVKAR

>WP_038707921.1 Tar ligand binding domain-containing protein [Burkholderia pseudomallei]

MVMNAFFSRFSIRTRIFSTLGLVAALLTITGVIGFFSMQNSNAALDDAYTRQLAAKTALASASLNLAITR

TTLDRVILHPEVPDAANTIAKAEQYLAASERGWRDYEALPRGDDEKTLAAQANAARRALADEALKPMIDA

LKAGRHDEADRLMMTVAPPLSVAWTKATNALDDARAAYGKAAYDDAEQMYGWLRLVLGGLIVFGLAACLG

CAIGLHFAITQPLVRILGHLRRLSDGDLTGELRWTSHDEMAELVSGLTTMQRSLSETVRKVTDGSESIAT

ATRQIAAGNTDLSQRTEEQAAALQQTAASMEQLTATVKQNADNAREAQNCADNATDIATKGATVVGEVVG

TMAEIDQSSQKVADIIGTIEGIAFQTNILALNAAVEAARAGEQGRGFAVVAGEVRTLAQRSASAAKEIKA

LIGESVERVATGSRLVGSAGDTMQEIQRAIARVTGIMTEISAASNEQRDGIEQVNRAVSQMDQVSQQNAA

LVEQAAAAAASLEEQADGLRRTVGAFRVVA

>WP_024429037.1 pentapeptide repeat-containing protein [Burkholderia pseudomallei]

MRHIKPQAALVATTNTQIGAQPMLGISVGIGFRLDQPSILVHEAAVWEALKAAAPSLPLYEAALPKQRAE

WLLAGHSVHAVGAGARARDVDWTAWVELDGVRKVVSCATSLGDEQAQSGYARIAVDHRHAAAGGARENPF

GVASGTPPLQQLRTFGVGPAPLAAMGAINPDWPERAQWMPTRPGTVDAMAQDGTHMGWPAEVDLRFFQQA

APDQWARGECWTPGARFELSGFGPRGEGFAGELPRLAPVALVTRNGRPGIERLSFKQQTAWFLPDRGIGV

LWWNGAVALDFLLDDSPTMLVTAFKDEAERIDVDALMKFADQRADLNCIDPLQQADHELMPAITRGWTWE

MILDTEDHPRFAPAPRGYEEVRARVEQNRRELVEARDASERLSAFEEANRNAKLPGAPRGGENWRTRLRQ

AKTPELANVTIRDADLSSLRFDGWKFDDVRFERCTLDRSEWTNCRLNQVHAVDCSFADVKMSDGWWKGGK

IQRCNLERSAWLNVEIERISLDECRLDDLKVAGGSWSMLSVQGRGGVRGDVQDIQWNSVSWSEVSAPGWT

WTRVRADDLAIVECAMAGLAVSQCTLAKPSILLTDLSASVWQRSMLTFAVLSHGTSINGARLTDCVFKSS

SLQELRADRVQVDHCSFMQLNAQHLHAQQSHWSRTVLDGANVMHAQLTGTSFDRCSLKEAMFYGADMRQT

RMRDCNLVRVRTSWIHPPEAGAWRGNLSAGQLDVPRRV

>WP_024428834.1 MCP four helix bundle domain-containing protein [Burkholderia pseudomallei]

MKLFFDMKIGTRLIVLILAALAALCAIAGFGIYESRRVYTAASYSTVNTVPSFVVLDDAQRAFDSMLLLV

NQQVFSTTADQAKALEPRIAQARREVDAQFAKYETLLSNDKDKALLAADRARVSQLDAVRENVLALSRDG

RKQEAGELMGTRMTELAQQTNAALAAHRAFNVDLGQAGSNEAKDIIDRAVTLEASAAAVVLVLVLWLGVL

ISRSITKPMGNAVKFARTVADGDLTTHIGAASKDETGQLLKALGDMNDSLKRVVERVRMGSDAVATASGQ

IAAGNLDLSSRTEEQAASLQETAASMEELTSTVRQNAENAQQASGLASNASDVALRGSSVVDRVVDTMND

ISERSSKIAEIIGIIEGIAFQTNILALNAAVEAARAGEQGRGFAVVAGEVRGLAQRSSSAAKEIKELISA

SVQKIRDGSALAGEAGRTMTEVTQAVARVTDIMGEIAAASAEQSRGIEQVNLAITQMDEVTQQNAALVEE

AAAASHSLEEQGHELKQAVAFFRFDGSAAGGTADTAAPRHRAAPPPHAAATAPAAHATPAPHAPHASHAS

HTIRTAHATPAPRAFDAVKPAASAAPAAAAVSAPALSGARATAADANADWETF

>WP_024428705.1 L-2-amino-thiazoline-4-carboxylic acid hydrolase [Burkholderia pseudomallei]

MGCARAAGARRAYLAQVPRAACVSYVLMRAMHAIRGAAERADSTDAARLPAVLHTLKRQGVTGAIAFGGE

GNLRDPAFTIYRAGGRTWAVVGVLGGVGGVGGVGASGNAAACVAAGVSGLSGGKAARLARFRESRERIER

RIEMATSPGGRPMTTIPIHPAAGGAAHDEPLGILARRRIEAEIIKPIYEIMKREFGLERAQAVIAEAVRG

AALDAGRAFAAKEPGGTSIASFVALQVLWEKDDALDVDVHRADDAHYDYDVRRCAYAQMYREMGLAEIGH

LLSCARDSVFIEGYDARIALTRTRTLMQGGTHCDFRYRLAQPPRDGAADATHAAPGGLAGGMREARVREN

EPAGQPEPAPGDRAGPSPESPPRSRAESRTETGTEPGTASQASGDAPGIRRASSPTFDRRAVAASPAGEH

TRPEAGDAAR

>WP_024428586.1 Lrp/AsnC ligand binding domain-containing protein [Burkholderia pseudomallei]

MRATKKSSPKAAAPPAGVDIDRVDRAILRVLQRDASISNVALAAKVNLSPPACLRRVERLKEMGLIRGIV

ALLEPKALNAGMLVMIGVVLDRSTPDSFGAFEQAAQKVSGCMECHVVTGEFDYIMLIRTRDSDTFNRLHA

EQLLYLPGVRQIRTFVVLKEILSTTSFPI

>WP_024428550.1 chemotaxis protein CheW [Burkholderia pseudomallei]

MSEVQTNHPAAPNAASRRDAEQGDAAGQELLVFTLGDEEYGIDILKVQEIRGYDSVTRIANAPDFIKGVI

NLRGIIVPIVDMRIKFHLGRVEYDHQTVVIILNVAHRVVGMVVDGVSDVLTLSTEQIMPAPEFGGVLTTE

YLTGLGTVDGRMLILMDIEKLMTSKEMALIETLGA

>WP_012730429.1 carbohydate-binding domain-containing protein [Burkholderia pseudomallei]

MNRISHSLCAALLAAATLLPTASRAQLPARPTAGAAAPATAAPVRPASTPAELAARLANGLAVRVAVDNN

HAASAGVPCADLGADWASCATGRLILQNRGHSPLTDGGWKLYLHSIRRLLRIDRPGFTLRHLTGDLYELT

PQPGTVRLAQGERIELPFVAEYWLRRYSDVIPRPYVVVDGAAPAVLRYDDTDDELRYVETLPADAQNNSP

GNAPPAAAQPVANRALPSVKRQRALPGALDLRGVELTLPELPSAQVAALRERAGTLGLDGARVPVWGVVA

PRRLPADIAVPGGYRLAIGPRGAFIEGADRAGLYYGVQTLFSLVPAGGATVPAMLIEDAPRFTHRGMHVD

LARNFKPPATLRQLIDQMSAYKLNRLHLHLSDDEGWRIEIPGLPELTDVGARRCHDPSETRCLLPQLGSG

PDDRSGGGYLTRDDYVALLRYAAERFVEVIPEIDMPAHSRAAVVSMEARYRRLHAAGREREANAYRLLDA

QDTSNLLTVQFYDRRSDLNPCMPGALNFASKVIREIASMHADAQAPLRIWHFGGDEAKNILLGAGFQPLD

GADPGKGRVDLAAQDKPWARSPACTALLRRGEIKSIDELPTRFAKQVSAIVNANGIGTMAAWQDGIKHAS

GPREFSTRHVMVSLWDTIFWGASDSARDLSAKGYRTVLALPDYLYFDFPYTRNPRERGYYWGSQATDEYK

VFSLAPENLPQNAEVFGDRDGNPFEVTSAGAAPSIEGIQGQAWGEVMRNGQLLEYMVYPRLLALAERAWH

KADWELPYAAGVRYKLGDTHHVDTAALERDWAGFATVLKQRELPKLERAGIGYRKPTFTLTGE

>WP_011205663.1 SPOR domain-containing protein [Burkholderia pseudomallei]

MRTERVERRPRRTERPDTDALLLDPTLPEKQRARRRLVGAIALVVAAVIVLPMVLDSHPKPVTDDVQIDI

PNRPSHQAAAANDEDASDVQAGVAHDEPPASEPAAAPTPAVAAKAQAKPDAKEAKKPPTAAAKAPSAAQK

PAAQPAQDTAAASVRAPAATATADGDASSPASPPGARFALQLGAFRDDATARNWATKLKAAGVPAYVEHR

KQADGSTATLLRAGPFADRAAASAAIAKVREAGLTQ

>WP_011205208.1 nuclear transport factor 2 family protein [Burkholderia pseudomallei]

MRASRKLAISRAARRARRRGRSMQAHQPYFDEIAAGGDAIGNWLSGDVSGPAALDALMARFAPHFTMIGT

DGAAYDRAATRALFARLAGCKPGLKITFSQMHALVADAAHGVISYCECQTDATGALPTRRSTAVFERDPQ

TGAVRWTHLQETFCAA

>WP_011205170.1 fatty acid desaturase [Burkholderia pseudomallei]

MRAAARALRRMAANRGATARRASLSLCVTDSMNDSSHPIRVDEPMPHRKVIRSWITPFGERVIARSIVLL

AVDYLLLFAAFAGALLAASGIVKIVCGMAAGFITGRLFIIGHDACHQSLTPNHRLNRWLGRIAFLPSLTP

YSLWEVGHNVVHHGYTNLKGFDFVWAPLTPGEYAALSPARRLLDRIYRSGWAPGLYYLVEIWWLRMYFPA

KAYLGASRPIFRRDCLLVTGFAALWIGAVVWIAAATRQSMPLLLVTGVAVPFLFWCSMIGFVVYVHHTDP

RISWHANRAEWSRAAPFVSTTLHLTFPFGIGGLLHHIMEHTAHHVDMSIPLYGLKDAQAKLEAMLPGRIV

VQRFSWRWYFDTARRCKLYDTDRKRWTDYRGRATSDAHLRADAPMAEQRPIDAPHGPGGA

>WP_009932959.1 CopD family protein [Burkholderia pseudomallei]

MNDGFLGMVRLAGVALQSVCFAIAVGVLLGDQWLARAASPWQAQVGRRLARTLRLAALGMLLSSMLAFWV

HCALMSETALWDAWPAVRSMLAGTGYGRAWLAGAALMLAVVGLSFARSGGDARVPFALWLALAGVALARS

NGGHPVDAGLFSAPVWIDWLHLLAISAWVGLVIVATYVVVPRLADARGGERRNGAAFVQSLSDAATFALV

VLLSTGAYNGWRGVGAPANLIGSAYGQVLLLKLGLVAFAAALGGHNRFFEMPALLAAFKAPPDARAFVRS

LRRFGVVLQVESVVLLGVIAAAAVLASSPLPGTL

>WP_004549699.1 thiamine phosphate synthase [Burkholderia pseudomallei]

MSARDGDRAPDHDMHDDLALPPYYLITPEPASGSDADLAAFLDRLSDALATGLTLVQLRVKTLDAPAYAA

LAAGALARCRAQRARMIVNGPIAVEAALALGAAGVHLGSAALRAATARPLGSEGLLSAACHSLDELRHAQ

RIGADLATLSPVLPTLTHPGAPTLGWTRFAECAAHTRVPVYALGGMTRTHLETARAHHAHGIASIRGLW

>WP_004547182.1 zinc-ribbon domain-containing protein [Burkholderia pseudomallei]

MLLATRCPHCETVFRLQREQLALHDGLVRCGHCQQVFDAARALVPTEPEAAAAQAAPRAPEQPAARRLFD

ATSPDRRPLEAGHRDFTPGAWDMRGPWLDGAIDPKLQMTSASVSTAAGGAVAAHAAAHETRNAAADTPAQ

EASKASDALSVSEASQASRASDTSTHDTLPVPETPETPAGGTPAPGERPAHAWPEANAGAPAIRPSDADL

PHAGAREFAPPPRPTLDLDLDRSATAKHKPDTARASASPPGDAVEPMLASHAAAAAALAGVPHDREPRFG

AAPPARADAEPFAAAPEADNREHFAMTRETRTDAARGGLARALVALVALALAALLAAQLAWWQRETITIY

WPSSEPLFKQACAALGCAVTPPRAIDGLRLDASDLRQLDGPRLLELKVPLTNRYRVALAYPSIELTLLDE

ANNITARRVLAPRDYVRPGTRVEAGMPAGATQTMIVRIDTDGIAASNFRVQIFYP

>WP_004538997.1 MULTISPECIES: collagenase [Burkholderia]

MKNSHNVVNRFIVAASIIIGVVLYSSAWANPQPMHTKQARMPRIPQNLPLSPDQAKYDLPLSKYDRATLM

EPLRRKQSAKPDRRTRPGADCRDMSIMTQYHGTALADYIANLPDYECHYGLFSIDRAMAAQIFNSENVWA

VASRLTQEINRYDATNITLVNLLIYLRAAYFQYDAAQLADPIPGLVVWLRPYILQSLSGDALYLENSRAP

STANELMILITNMKDEAYYLPTLKDRIAFYTASATNPQAAAPLLQRSAAGGFTGLLTVFFYAHQRSGAQP

MLDSDATLPETLNRFVTANRAYLSNTSAAYQLADAARETYRFLRYPSQKPRVKKMIQDMLASTTMTGPDN

DLWLAAAEAADYGDPGNCADYGTCDYQKRLIEAVLTHRYSCNANVRILAQDMTVPQFQSACQSVAQEEDY

FHRMMKTGHVPVANDHNDTIEIVVFGDYDNYRKYASVIYGISTDNGGMYVEGDPSAPGNQARFIAHEASW

LRPEFKVWNLEHEFTHYLDGRYDMAGDFAASTAKPTVWWIEGLAEYISRKNDDQESIDAVRTNAYRLSDV

LQTTYSSGDYVTRAYRWGYMATRFMFERHRADVDAIVSRFRVGDYDGYADYVAYMGNRYDSEFVDWARGA

TTTGEPPLPPTKAGH

>WP_004533858.1 MULTISPECIES: nuclear transport factor 2 family protein [Burkholderia]

MIGGLKIVDSSSNALYTRQIHAYVKALERGDVEAICVLFTPDAQIVSPFLGRMQPAPFFAKVAAASGESA

IALTDVCVSASGARRAMGYLVYDWGLKDGSSVRFECVDVFEFDANGLIERMVIVYDTHPIRDVVGDKYA

>WP_004531925.1 MULTISPECIES: chromate transporter [Burkholderia]

MTIASVEAACCGERESLWALFKTVTGVSAVSWGGLAMMAQLERHYVEHERRIDPLSFADLVALAWMMPGP

VGCNVAVQVGHALRGRAGAWIAGVASVLPFSAAMTVFAIFYQTPLVRSLASPVLLHHFAMVLAALIGLTW

FRQVRALVHAPLERVIAALATALLALAHNPAAFVAILAAAFAVGWLASGRKQGEALRLALPAREWRLLAS

LALLIALFALPLPNEYESSLLWPRLAGAGMTLFGGGFSALPVLKSLFVTRSTGITEQDFMLAFTLSPVSP

GPLLNVVPFLGYLEDGWRGALLSTVALFVPSGCLVIFARRHVERLKRHPRFASGMRVLRAATTAFLAIAA

VRLVAKTPAEPMYWATGVIAWLCLARFKVPVYALYGAVAAACGGWLILAAHG

>WP_004531444.1 MULTISPECIES: acyl-CoA-binding protein [Burkholderia]

MSDITAQFEQAQIDVKQLTERPGNLTLLRLYALFKQATDGDVHGDKPGFTDIVGKYKYDAWDALKGTSQD

DAKKQYVELVESLKNGTAS

>WP_004531247.1 TauD/TfdA family dioxygenase [Burkholderia pseudomallei]

MTQLSMPAAAACPTLGDLRVEPGLPTVVSPRDGADIALHEAAPLLREIADDVLERAGGVLFTGFRVASIE

TFQRFAADFGDPLIGYEFASTPRSQVEGAVYTSTEYPPHRSIPLHNEQSYTREWPLRIWFHCALAARTGG

ATPIADSRAVYRALDPALVARFAERELLYVRNFGQGLDLPWQQAFGTGDPREVERICAARGIDCEWRDGD

DGEPLLRTRERCQAVARHPRTGELVWFNQANLFHLSALDEDMQEALVDAVGIENVPRNVYYGDGAPLEPD

ALAEIRAVLDGQRIVFPWQTGDVLMLDNMLSAHARDPFEGPRKVVVAMARSYREKRGA

>WP_004530879.1 sensor histidine kinase N-terminal domain-containing protein [Burkholderia pseudomallei]

MATPVRSAHASRRASAAAPDTDAARDARYENPFAPPDEADAAEGTRPRSLFGEILDWMLAPLLLLWPMSI

AVTYLVAKTIANGPFDRALETDAYVLARQITPINGVAELRLPQATLDFLRADNVDSLYFQVLGTRGELVA

GEADLPLPRDDDRPAPGVVVFRDDLLRGNDVRVAYTSVALQGATGSQPVLVQVGETLDKRNALANDIIKG

VILPQFVILPLAILLVWFGLSRGLAPLTALQAHIRGRRPDDLSPVEARRAPPEIEPLVTSFNDLLARLEQ

NITLQKRFIADAAHQMKTPLAGLRTQAEFALRHEVNADVARSLEQIATSSEQAARLVTQLLALARAENRA

TGLTFEPVEIASLARQAVRDWVQAALAKQMDLGYEGPDTDAPLRIDGQPVMLREMLGNLIDNAIRYTPAG

GRITVRVHAERAAGAVHLEVEDTGPGIPPNERERVVERFYRILGREGDGSGLGLAIVREIVAQHGGTLTI

DDNVYQTSPRLAGTLVRVSIGLRQEAPE

>WP_004529898.1 (2Fe-2S)-binding protein [Burkholderia pseudomallei]

MIVCVCKSVSDRKIRASLAEGVNTFEELQFELGVATCCGKCEETVREIMAEQGVCASRCGVERPAAVPVA

VTFYERKAA

>WP_004529170.1 ABC transporter permease [Burkholderia pseudomallei]

MNGILRLAFKLLVNDSAKFTALLVGITFAVFLMVEMTSLFAGILDKSSSTVINVGAKMWVMDPGVQTIAS

SIGMPAYVLDAVRSVDGVKYAVPIYSGGALVKLADGTYQAVTVIGLDDASLLGRPTMKAGRIEDIYAENG

FIAIDDAEFPKLKNPALGTTFELNDHRGVIVGIAKVASSGLFGTPTLYTTYARATRYIPSTRYTTSYILV

EPKSQRDIARIKREVAALGYRAYTKEEFMRRISDFYKYETGVGTNILLMTAISFIVGLSISGQTFYAFII

ENLEKFGALKAIGAKNRELVAMILFQATFTALTGYGLGVGLCTALISVARLRLPSYAALITYGNLALAFG

MVVVIAGLSSYLGVRRVLRIEPFDIFRG

>WP_004528338.1 MULTISPECIES: efflux RND transporter permease subunit [Burkholderia]

MNAGAPHDSGAARARFTDVFVRRPVLSLVFSLLILLVGLRALMSLPVRQYPALESATISVATDYPGASQE

LMQGFVTTPIAQSIATADGIEYITSTTTQGKSVVKARLRLNANADRAMTEVMAKVQQVKYKLPADAYDSV

ITKLTDAPTAVMYLGFASDALSIAQITDYVVRVAQPLVTTVPGVASAEILGGQNLAMRVWLDATRLAAHG

LSAGDVAAAIRANNVQAAPGQVKGSLTIADISANTDLTDVGAFNEMVVKSAPNGGGLVRLKDVATVEIGG

ENYNSSALMNGRRAVYIAVNATPAGNPLEIVRGVGAVLPAMERNKPASVQIANMFDGARFVNASIAEVRS

TLTEAIAIVVVVIFLFLGSFRAVIIPVLTIPLSLVGSAALMLAAGFSLNLLTLLAMVLAIGLVVDDAIVV

VENIHRHIEQGEPPVRAALIGAREIASPVLVMMATLISVYAPIGLMGGLTGSLFKEFAFTLAGAVLVSGI

VALTLSPMLGSLLLTSRMSEGRVAKAIERTLERVTHAYRRGLHASLAARPAILLIGAGVLAGIVLLFSGV

KRELAPQEDQGSIMVAVKAPQYANLDYMERYAPDIERVFRTLPEADTSFILNSYGGSNLGFAGVNLVDWD

KRTRSAAALQALIQARGDGIRGERVFAFQLPALPASSGGLPIQMVLRTPSGFADLYAQMERIKAAAQKSG

LFAVLDSDLTFDSRAVGVTIDRNQANTLGVTMKDIADTLAVLVGENYVNRFNFKGRSYDVIPQVSRSERL

SSEMLSRYYVKTGSGQMIPLSTVIQVTNGSQANALSQFNQMNAATFSAVPAPGVTMGDAVAFLEAQKLPA

GFSIDWLGESRQYVQEGNRLAVTFGFALIVIFLVLAAQFESLRDPLVILVSVPLSICGALAPLYLGFATL

NIYTQIGLVTLIGLISKHGILMVSFANDLQRHEGLDRRAAIERAAAVRLRPILMTTAAMVAGLVPLVFAA

GAGAASRFAIGITVSTGMLVGTLFTLFVLPTVYTFVAKDHGAAMRSERAQLLAATR

>WP_004527379.1 MULTISPECIES: type IV pili methyl-accepting chemotaxis transducer N-terminal domain-containing protein [Burkholderia]

MAPALPDSPAPWRHRLSTRIVALSAIAWALVLAMVGGTLWLSWQLEGAGAAINDAGSLRMRATRTYVELS

GVGTEPRRQLAVELDAIDGTLARLRRGDPSRPLFLPNTTVIQHQLDIVTDRWNAVLRPLAAGALTNAPRA

DAAAAYLVALPGFVDEADLLVREIESDNARKTTWLRLSQIGLGLLACSGTVAVIYLLFLWIILPVMQLRD

GLKRMAAREFAVRLPVQTRDEFGDLAHGFNRMASELQEVYAGLEERVQQKTAQLAAQNRELSALYEITAF

LNRPQAVDEMCAGFLSRAIAQFDADAGSIRVTDPTGEKLHLVIAEGLSTELTELERCMPVDDCFCGTVTR

ADTAVLHDLRGSAARAPTPCSREGFAAVAVFKVMTQDAVLGSFSLHYRDPSRLPDAERRVLETLGRHLGI

ALDHVRLSASARQLAVAEERNLVAQGLHDSIAQSLNFVNLQTQMLGDAIAHDNLADAREIVPMLKHGVEQ

GYADVRELLLNFRTRLTQGELKAAIEETIARFEKQTRIACALNYRETGGAPLPPDEQLQVLFILQEALSN

VRKHAHARHVAVDVENGAAFRLSVADDGCGYDPAALAARADTHVGLTIMRERAARLRATLTLTGAPQRGA

RVELVLPADARQAA

>WP_004526813.1 FecR domain-containing protein [Burkholderia pseudomallei]

MNARGFVTKYVLTACCAAATALAAQHAAAQSAKNRSVATVDYTTRSGDTLYDVSARYLQGTDDWPLVAQL

NDVPVPKHLQPGVVLKLPAARLRKERLSARVIAAHGTVESAGRGSAQFAPVAVDATLTEGDRLRTGSNAF

VTLELSDGTHLSLPPDSQIDLATLRRTVLTGTLERVIDLRRGSVDSEVTHLKKKDDRFQIRSPSVVAGVR

GTRFRVNYDKDGRASTTVEVLDGTVGVAPSAKRSADTLVHANFGNVTSASGVVGSPIALLDAPQLANPAK

IQDDPQVAFDLVPLGGAQSYHVQIARDAGLYDLFKEVQVPAPRATFADVPDGTYFVRIAAIDSHGLEGQP

RIYAFERRRFGVDASAAPADGGYAFRWSTTQDGAAAGATRFRFVLSRSKDLSNPIVDQVDAQGGRIAVSN

LTPGDYYWSVIAERYEGGRFHEKASAVNAFTIAR

>WP_004525995.1 alpha/beta hydrolase [Burkholderia pseudomallei]

MNAHTQKSLIAGPVGHIEIAIDLPDAVRDGSAAPRGIALVAHPHPLFGGTMDNKVAQTLARIFVQLNYAV

IRSNFRGAGATEGEHDNGAGEVDDLLAVLAHMRALPGHADLPLVLAGFSFGTFVLSHVGKRLRDAGQAIE

RMVFVGTAASRWQVAAVPEDTIVIHGENDDTVPIASVYDWARPQELPVIVIPGAEHFLHRKLHILKRIVV

GAWR

>WP_004524539.1 MULTISPECIES: prepilin-type N-terminal cleavage/methylation domain-containing protein [Burkholderia]

MKPAVRSERRAPGEKRPRRARRAFRRGARHGARGFTLIEMMIAITILAVIAILSWRGLDQIIRGREKVAA

AMEDERVFAQMFDQMRIDARRAATDDEAGQPAVRVAGDTLQIVREFDAPGAAPRLQVVRYRISNGRVVRY

ASPPIGDVNALRDALRGGDTEGWSEVALMRGVGMINARLYVPKVGWTTSMPDADNALEQNNNALKVPMLG

NAPPPRAVTGLEVSIGATSLRVPITRIFLIGE

>WP_004523929.1 gamma-glutamylcyclotransferase [Burkholderia pseudomallei]

MSQVCYFAYGSNMSAARLSERLSRFGEALIERRPGIVEGYRLTFNKVSSRQDWVGFANIEAAADCRVEGT

LNAMSARALDALDSIELVPLHYRRAGVLVRDGATGRLTFAVTYVANPAMVRPNLRPTRDYLDHLLAAADV

LPRAYLDHVRAVECWT

>WP_004193734.1 MULTISPECIES: carboxymuconolactone decarboxylase family protein [Burkholderia]

MEFIESIKAQIPDYAKDIRLNLDGTISRSSLEGTDAVGAALAAAFAAKSPVLVKAIREAGVLSPEETQAA

LTAAALMGMNNAWYPYVEMADDADLKTQRAELRMGAYATHGGVDKRKFEMYALAASIVGKCHFCVKSHYA

LLKNEQGMTVTQLRDVGRIAAVINAAAQVISAEGK

>WP_004192197.1 MULTISPECIES: metallophosphoesterase [Burkholderia]

MPNPIQPLKRRDFLRLAACGGGVAFASALPGWSFAANAGADFFFVQLSDAHWGFTGPAINPDARGTLPKA

IEAVNALPVAPDFVMFTGDLTHTTDDPAERRARMRQFQSIVAQLRAKPLHLMPGEHDASLDAGAAYREIF

GDTHYAFDHKGVHFVVVDNVSDPAGRVGDAQIEWLARDLARQPKDARIVVFTHRPLFDLAPQWDWATRDG

AKVVDVLMPYPNVTVFYGHIHQEHHAMTGHIAHHAARSLMFPLPAPGSQDKRLPVPWDAAAPYRGLGWRE

VRVGDAARAPALTEMPVAAPQPQPRA

>WP_004191562.1 MULTISPECIES: chemotaxis protein CheW [Burkholderia]

MNAILEAPHAARMSGQDAARDAQEFVTFRLGTEEYGIDILRVQEIRSYEEPTRIANAPAFIKGVINLRGV

IVPIIDLRLKFALDSAEYNTSTVVIVLNVAARTVGVVVDAVSDVLELAAADRRPAPEFGAAIDTGFITDL

GSIAGENGNRMLILLDIERLIAAADVGLVG

>WP_004186322.1 MULTISPECIES: prepilin-type N-terminal cleavage/methylation domain-containing protein [Burkholderia]

MPCTGRNSPSRGFTLIEVVVAIAIVAVLAAFAVPSYRSYVERVNRLTAVAALYRAAQYVDAFGDAPPTAL

PEGVNRAPESGKLVYVLRIMFDDARGGYALEARPAADGAMRDDRCGVYVLHADGTRENRVAGGVALDGGA

AEGDACWRTG

>WP_080298288.1 endonuclease/exonuclease/phosphatase family protein [Burkholderia pseudomallei]

MTSTMMPPDTPTPAGAPPNAPTAGDALRDVAPQTGARALGAAAARAQPRTRELRIATYNIHGGLGAWTAS

AAERIAVVLDELRADVIALQEVPLGGVRGADVLAHLRACTGMHAAEGPTIDTPARRYGNAVLSRFPIRAA

RMLDLSFGNREPRGALDADIECGFGVLRVVATHLGLSATERSAQVARLLAAFDTRALPVILLGDINEWFV

RGRALRALVTHFRRAPAPRTFPTLCPLFSLDRIWVHPGEWLVDVAAHRSARARRASDHYPLVARIRAAPE

TDADIAQAPASASRTPPERPCEPTDSTEPASTTGAPARSPATTPRCASSPSRRASSSP

>WP_079997073.1 sugar transferase [Burkholderia pseudomallei]

MNVEVRIKLLTNRNRGSLMPAIFPRVVDIAVIVFGAFLPILIEASGGPHAEIFDGTLVAFAAALSLSVFP

ACGIYEASRRRSPVHLISRTALAWLVVQGGTVVLLYVLHRAQILSSAWFAYWTVTTGIGLLIFRAVTLAI

FGLLARASRQVKAATLDQVGHVAQRMRTSGIAKRIVKRAFDVAAASCLIVVLSPALAVIAFLVKRDGGPA

VFGHVRIGRDGRPFKCLKFRSMVMNADAVLKALLERDPHARAEWEREFKLKNDVRITPIGRFLRRSSLDE

LPQLMNVVRGEMSLVGPRPVVEAELARYGEDVRYYLAAKPGMTGLWQVSGRNDTSYATRVSLDVSYVKEW

SLRRDLVILLKTVNVVLRGSGAY

>WP_076936737.1 O-antigen ligase C-terminal domain-containing protein [Burkholderia pseudomallei]

MPSSFLRSLSLIALAVALILPYAITNHTYPIPTFYSEFAAFALYWGLGASVVLLVKAERAEQPFAAPMAL

VAPLGFGAVLLAQIALLPLRQPSMNWLAMGYLLGALVAMQAGYALARVNMVDMVARMIAGATIVGGVVAV

ACQFVQLFHLETTFSPFVVSYGVTVERRPYGNMAQANHLATYIAFALAGALYLVQTRRMPALAWAALSAL

LSVGLALTVSRGPWLQVGVMVVAGFWMAFAQARRDPAASRARAWAIPVVLGVLFVAVNVAVRWANVHYHL

GLAESAADRMRDSGQIAPRLALWKYGLTMFREHPLLGVGWGEFPIHQFELARRLGGVEIANNSHDIFIDL

LAKSGLLGLGVLFVALVAWFVRALRVPHTESRVFGFALVGIVLMHALVEYPQQYTFFLLPVMFVIGLLET

KPLRALPGRAAFVLFAALSVAGLASLYPILRDYQRAEVLYYGTNPAEQYRAHQSFLFGAWGDYGAATLLA

ISRENLPAKLAAHESAIALLPGETVLRRYAVLQALDGRETDALDTIERLRVFAEELHDWPVQLAALYKLL

DDQPSLKSFKAALVAKYGTPAANLSADDEEDDGDD

>WP_076903245.1 PIG-L family deacetylase [Burkholderia pseudomallei]

MTSTHRWLVISPHLDDGVFGCGQLLAQSPGSIVVTVFAGVPPPRTPAPPWDRRAGFATGADAMRARRAED

ARALRVLDATPVWLDFLDDQYGAPAAPDDIAKQLAATLDAHPGFDVAAPAGLFHRDHLATNRAALAVLAR

GPRARRWLFYEDALYRRVDDLMAKRLADWRRQGWIARVAAPLAHARAGRAAKAEAVRAYASQVALFDAHA

LVDLHVSETYWRLQCETSSA

>WP_076859550.1 lytic polysaccharide monooxygenase [Burkholderia pseudomallei]

MASSVGLADVRHVCSQGGEMSTTDSRSATKSSSISPRHGRVITPESRAVYLYEAGRLDFGQVNELEGGKF

FPATQSGLRDPDAPDDVASGMPPRDGEIASGGRTADARAQLNEPDSVAHWQKHAVRSGQSLQISWSYSMP

HKTRRWTYWITKPGWDTQARLARAHFEPDPLKVYLNTYQPYWGPDADKELIPQGETIHEFNLPTRTGYHV

LLAVWDVADTANAFYQVIDLNFA

>WP_073699290.1 MULTISPECIES: iron-containing redox enzyme family protein [pseudomallei group]

MTLSKERKLHIPFERDGDLMDIGSYPHWLQDVVGTVRAARDRVRFHEVFSLMRDSRLAPRQLAAFFVNGW

PVVEQFPKYMSMNLLKANGTNSSGEEKARRYLIRNIRVELNHVEHWVNWAEASGVPRRQLTDGDSPPAAL

ALSHWCWKSSSADTLAASIAATNYAIEGVTGEWSADLCRSDVYEMGFPEAVRGRAMRWLRLHSSYDDKHP

WEALDIVATILGQSPSTEQVRDVAAGIERSFRYFEMSLSCCLDA

>WP_038708282.1 CHASE3 domain-containing protein [Burkholderia pseudomallei]

MASGIGRFGKRASRLIDFGLWAAAALLLLTAGVGALGTARLSDNEADIHRASEVISGLERLLALARDMET

SQRGYILTGKTDYLQPYKAALPEIRKQFDALTTLVQDDAVQQQRLATLRMLLEKKQHELAATIELRKTEG

FDAAQAIVSSDAGKIFMDRARVMVNEMEATVQQQLRTLRDRARATRDIAIGVGLASGLLTLAVCVSFGYV

MRRLLLVEAAAAHDLFEQRELLHVTLASIGDGVIATDVGGRITFFNRVAEEMTALCADAVVGQPIERVLM

FRHATTQRVIDNPARVAVERRRTASLPAHTRFVGRSGGELPVDGNAAPTFDANGKLVGAVLIVRDVSERE

RAEERFRLAVEAAPTAMIMVGRDGRIVLVNSQAEKLFGYARGDMIGHGIDMLVPERFRAGHWAHRDAFFG

NPDARPMGEGRDLFGLRRDGSEFPVEIGLNPITTSEGAFVLCSIADITARKRAELELRRRSEELARSNQD

LEQFAYVASHDLQEPLRAVAGPLQLLQRRYQGQLDARADEFIGHAVDGATRMQSLIDDLLSYSRVGRLED

ARRPVDCEHVLDEALRNLSAAIRESGAQVTHDPLPTVHGIEAQLVLLFQNLIGNAIKFRGKDRVARIHLE

AHTRDDAWLLRVRDNGIGIDPQYFERIFLIFQRLHTRREYPGTGLGLALCKRIVEHHGGRIFVESEPGQG

TTFSFTLSRPGADETRWPRDPRE

>WP_029671474.1 haloacid dehalogenase-like hydrolase [Burkholderia pseudomallei]

MKTGRRHFVRSVASASAALAAAAWSPARAAIDAPTSPATALSLTPGRWSPNNVARLRAVLAGHGASSPRY

RPEHRPYAVFDWDNTSIMNDCEEALLMHQIDGLHYRLTPEQFSAILRQGVPDGPFDAKLGYTSVDGKPVR

MEDIAADVDADYRWLHANYRGLAGDKPLDEIHRSEQFRDFRAKLYFMYDAICDTYPVEIGYKWIMYWYAG

MTRDELQAMAFDSNVANLGDALRKVTYESSRALPGKAGVIAATHFHGIRIHEEIRAVMDTLRSNGIDVYV

STASLDDVVRVFAGHPAFGYGVPAENVIGMRLTMADGKYMNEYLPNWHFNYGPGKTVGIRRELESKKGYG

PLLVFGDSDGDAWMLRDFADTAVGVIVNRMKKGEIGIDSRKAAEQIGAKDARLVLQGRDENTGLMVADER

SIKYGKRDPKLLA

>WP_029671387.1 VanZ family protein [Burkholderia pseudomallei]

MTPAARRQRRHSTLARQAFAVYAALVVYASLYPFTGWRSLGIGPFDFLLAPLPRYLTAFDVVSNVLGYLP

FGALAVLALYPLRGVPAALAATLLGALLSGAMEALQTYLPTRVSSNLDLAANALGALVGAAAAAPAATAL

IERGVVRRVRFAWFEREASTPLFLSALWAGAILFPSPFLFGIGDWPSELWERADVTMRGALLAWAPDAWN

VPAWPERLDGLLSDSAWETLLAALGLFAALAVVSLAMRERAPRVRLVVGFAACALALKAAATFMQSYTGL

VLDWATPGALRGIAAGLVAALVALRLPAAWRAALAAAALAAALVLVNVLPVNPFFDFALSGWQQGRYVHF

NSIARWLAWIWPYAALVWLAGRAERAWLARRGAGASRRASGKRRRSL

>WP_024429131.1 SMP-30/gluconolactonase/LRE family protein [Burkholderia pseudomallei]

MTKRIHAVLLAALLIAALAPPCQARSSTDWLANTFATDAMHVGNAARSMWVAPEGVIYTASMWDENEGGV

ALYQNGRSIGSIGAHNEFQGGAITGNAASIFAALQFNRNGGSGMVGRYDRTTRRRDLLIPVSATTTERWA

DVVTGLATSGSTLYASDFPGNRVRIYTVAGVWRRDIDISEPGALAADSAGNVWVAQKRAGAIAEFDSGGR

LLNTIRMAAASRPSALYFDAATQRLMVGDEGPDMNIKIYDVSRTPALVGTFGVRGGYLDSASGIKGQVGD

KRFTRVAGLGKDAAGNLYVLNNPWGGTWDLGRDGATDIHAYSPSGTLQWKLQSVNFEGIAAPDPGTDGAF

FYSGTNVYAGTAGGTFVANTVDPIDYPTDPRIDGRDPARGEHFGQLAAIGSNRILAAGSQNPDVFHFFHF

NAAHGYIAIPDGSIPGAAFGTTARIRAGFCLDSRGGVWAGLDKTNAIWHYPLTGFDANGKPAWGPATVTP

IPNSIKPLTRIVYLPESDTMILARGRPVGTDWTAIGTRIEVYHGWLAGNTAAPNPVIGLSSANPKSITAA

GNYLFVGYVHTQPNIDAFNLSTGALAATLINSSPEQIYVGNDVDSMYGLRSYLRSNGEYVITKDNYNAAG

IVVYRWTP

>WP_024429128.1 type VI secretion system ImpA family N-terminal domain-containing protein [Burkholderia pseudomallei]

MTPSRKPAGRAAAARTPKPDGWMAPVDAAASCGADLEYDPEFVVLAAKVAPRAEAQYGDFVGSPEPVNWS

DVERDCRRLMMRSKDMRLAVLFARSRTRLAGATGLAEGIGLLAAWLAAFPDAIHPQADVDADRDAALEIR

RNALQALTDADGLLADVREIALTRSSATRLQVRDVERAFAQPRPGDALAPESVVRQLDDLHAQQPETLAG

FGDALAGLAAIDAWSGEHLGDYAPDLSALDALLRRIAGANARGDRAEAEPIAPAEADAPPASEAAAAHAH

ASPPRRREPAAQALAAAIAGEPAAQPVDRYAARELIRQARQWFEQHEPSSPIPILLRRAEHFVGKRYADV

VQAIPAELLALWSADET

>WP_024428898.1 SDR family oxidoreductase [Burkholderia pseudomallei]

MLIWKTALSLTWKAGLVSPASAPAAAAHVDRVVLTGATGFIGGAVLVSLVNAGLLDRVVCIVRACDRAHA

LARLRAAALRSGLAPYWAERLSEANVIAGELDGALADADAAHIALASHVIHCAGVASLADARIVNETNVG

ATLRFARRFAGSRRLQRFVHVGAAFACGLRARGTIREDDTPARGREIDFAPYTRGKRDAEAQLRALGLPL

VVVRPSCVVGHTLLGTQPSASTFWMFRIVHAARRFTARPMARIDVIAVDDCARALMLLALKPSLAHDTYH

VSAGDEAPTVTQIVRAMDEAVGLDDEPRYALCSPAEFPSIARDVLGRRDAPRERVIRRALQSYAAFAELD

HVFDNARVRREIDFEPLPFVDYVNECMRTSRGIDVLAQMPRTAAR

>WP_024428886.1 lytic polysaccharide monooxygenase [Burkholderia pseudomallei]

MKSLFDAPSSRPRARAALTLGAAATLTASFAALLAPMNADAHGAVGFPIARQYQCRLEGGYWDPPNGSAI

PHDDCRAAYRAGNNSAYPFTQWNEVSANPVGQGNDLVQLKAAVPDGLLCAGGDTSKAGLDKAPASVWRKT

QLTPNNGHIELQWENTTAHNPARMRVFISKPSYDPSRPLRWDDLQQIYDAPAPAPAPVPANGAGHLPGSI

QSFYKLDVTLPAGRTGDAVLYSYWQRIDAGNEGFFNCSDVTIAADERASGFPWVAARAFVEPGIAPRAGQ

QVRFRVMINDARGAEVVDVRQPITPYNAERSVWAKQIADQVNGRYGNIAKIGVRSGNTIYFDATNLDANK

VWLQPNYSSALSVVGAK

>WP_024428874.1 VCBS repeat-containing protein [Burkholderia pseudomallei]

MAKTSGTADEILNLPSGGGSVSGDGGDFSVDLNTGTATLKFDLAVPAGPNGITPPHTLQYSAGAGDGAFG

IGWSLGLMTIRRRITPATGAAEPAPPGACSLVGVGELVDMGAPRFRPIVDATGLLIEFTGASWTATDKTD

TQYTLGTSANARIGGGALPAAWLVDRCADSAGNAIAYTWLDVGGARVPQAIAWGTYRLDFVYEARPDVLV

DGSYGAPVTLDKRCARIELHATTEAPSLVRSWTLLYDDDGGRGRSLLATIREQGHAADGSILAAPERTFA

YSSPGAPALVPVTGWTTPLSDPNTDLVDLNGDGLPDIVHLGHGMPTMHPNLGGGQFGAPRPLARTAAPLR

LSAPSVAFADMSGNGSVDVLVLDAPFSGYYPLAAPGGSAPAGFGRPIVFERAPAVSPSDVRLRFVDLNGD

GITDILLDTGRGWLAYLRDGPASWSDAPRVLPPARTPPVSLADPHVHLADMTGDGLMDIVRVTGGGLTYW

PARADGGWDAAIAMTPSPAFGRDFDPHRLALYDVDGDGCADLVYVGLQSVTLWRNVGATRLAAPVAIPGT

PVALPGSYRIVDLLGSGTAGVHFQLPSIAGASRQSYLDLGGGVKPYLLTDLAHGAAQSTHIGYRTSTEYA

RDDAHAGAPWRTYHPFPIQCVARTDQTDHGTGATTSTRYAYHDARYDPATRTFLGFGRVDSEQLGDASCP

TLRVETTFHLGLDPADPARPLTGDEALKLGALRRKPLVTATYGLDGSPLEHRPYSITRHAYDALLVASTA

DNGKRIAVPYCTTSTEERWERQSAAVSARTVDYLAITSEGDVTSQRTRAQRAGIAAPDQDVTTTTTLATG

GKNLRLPARVTQTAPGGDIVSESICFYDGDAFAGLPEGQATRGLVTRIEDRVFDDAFVASVWGDTPPDLT

QYGYHRLPGEAGSWWKTRRAHARGANASGPTLATKGPLGALQTLQYDASGQRVVKVIDALGNAVAATTDA

RVFQTASLTDANGHRTADAFDALGRVVATIGPLDTPALPTIAFTYTVGAISTVTSATRGTHGGADAVPAL

TWIDGTGNVLGKGTPAAAPGEWTVTHAVRRNARGLVAASFLPYAATGANWQPPPADTGATTSTYDALGRL

VQLTRPDGLVVTSRREGDTLITSETWPGGAALDVERQVYDAAGQLVSVSRNAGDHWVEQRYAYVPSGKVR

DVTLPGGAHVAFTLDLLGRVFAQQSPDTGRTVFLLDASDNQRARTNAAGQIVRTEVDAMNRATNVYHDAE

PAPRVRYEYADASGAPPADGIVANRYTRLWRITDEIGTVDFEYDEAGRKTATTRTVAATGQRFVTRRAYD

ALGRLARATLPASAPGGAARTIAYGYAADGRLVSASGVVKDAAYDRFGRLTSIDYENGASTLIDYAANAG

GIARVRVLDAARNVLRDTTLTRGGGLVQTLASAHAGDDSVDFGYDPLRRLTSAHYRQGATAADAHDWTFD

DAFNATAATDAGVLTYEPGTHRLASVGGAAVAFDAAGRMTSGRFGAATFDAADHLSAVTLPDATHIAHTY

DYQGRRVRSTKNGAQTYFSPIEDIEFQGDTAIVWITFGKQRIAADIGGALTFVHPNALGVMDLITDSGGA

YGTRVRQTPFGYARAADGAAPAGGVAAVALALGAADATGLVCHGLRWYDPRVGQFISPDPVVTSVYTVGA

WNPYVYCLGNPILLADPNGCSFLSVLEIIGVAILAAACVVGAIFTGGATLVALGVLSANIGGWLLAGVAL

GSLGGAIAGELAAQKAGGNLWAGAFLGAFLGGATSLIGGALGGAAAAGIDTLIGGTKTFLSFVAAGAIQG

TLAGAGTGLAIGYAGGKGNAESMLIAMAKGAAWGAVLGTLLGAGIGAIAGTGISGAAKPDNFLNIGAFGQ

KFADFTSTSTAINSADNAAGVTESLAQLSMPNGFNAGNLLGLLPNLVTTNEQAAGWFSIPIGWLGPGVLN

DAGFAGLVDTSMALDQAGFSYAHQISLLLGAAPYFIDYAATMAQIVDANGVNNFEMAFNNAFGSGSPSNT

G

>WP_024428840.1 FAD-dependent monooxygenase [Burkholderia pseudomallei]

MAVVPLSTPPVLIVGAGPTGLAAALCLARARVPVRIVDKAARPARYSRAIGIQARTLELLEQQRVVDRFV

ELGHRARLAILYSAGQRIAELDFDPLQTRYPYLLFLDQTVTERLLTEHLATFDVHVERGVTLTHCAQPEG

ALDVRLRHGDGRDERLQPSYVVAADGARSTVRHLLDVDFVGHAFEQTFMLADLELGADWPDDEIHLFTTG

EGLAGLFPMGGGRYRLVADRPPRNGALDDEREPSLELCREVVRARVTPDLKVGDLAWSSYFHLHSRMVAR

LRVGRVFFAGDAAHVHSPAGAQGMNTGIQEAFNLGWKLARVLAGNAPERLLDTYHAERHPIERDVLRQTS

FATQVVEADRGPLKLLRDHIVPILASFGPLRDAARRTVSELAIQYRKSPLTLERVLDGGPRAGERAPDVL

VHVLDGPLGKAPGVARLFDLHDPAHFTLLVLEPREAIDDTLPSDPGEDGRALAGALERIMPGAVRCWCAT

DAEGEGAPLLADAYGRARPVFYLLRPDGYVAARGRPATDANALLRHCETWFSGMRLET

>WP_024428825.1 M81 family metallopeptidase [Burkholderia pseudomallei]

MPFAIQESDMNILIAGFQHETNTFAPTRASYQSFVRGEGFPPLARDVNVPIGGFIRAAQASGHALLPVVW

AGACPSAHVTSGAFERIGGEIVAAVQAGGFDAIYLDLHGAMVTEQFDDGEGELLARVRRIVGERMPIVVS

LDLHANVTARMAAHASALVAYRTYPHVDMAQTGERAARVLERLAAEARPLHCAIRRLPFLIPINGMCTHA

EPASGAYRLLAQLERDGVVSMSFAPGFPAADFPECGPTVWAHAFEADAAQRAADALFAKLVGDEARWSVP

LLAPDAAAAEAIRMSRTATRPIVIADTQDNPGAGGDADTMGMVRALLRCGARDAAVGVIWDPDAAAAAHR

AGVGARIGLRLGGRSRVRGDAPLDAEFEVEHLSDGRFRFDGPMFNGAHGELGPVACLRIDGVRIAVSTNK

MQTFERNQFRVAGIEPERTKIVVNKSSVHFRADFEAIADAILVAKSPGPMAADPADLAWARLDPDIRVRP

NGPTLRSLRAMAR

>WP_024428800.1 metallophosphoesterase [Burkholderia pseudomallei]

MIPIEHFEVPHLALWKSCVAEVLAHALGVEHRPPAGIDTDHPLMRATDRYCRAMLENRPLGAPAAGSDDE

QAVQTYLSYLHHRRAHAHIAGDAQIERDIERQTQQYKFGNPLWQQMYIQYFKYYWQYPYHKGGEPRYRSW

QAADAGNGDLRYGVIEWKLSARARVAIVGDIGTGTDVAAAVLVAALKFAPDAILHLGDVYFSGTRFETDH

RLIGLVREVLRGNGRRVPFFTVPGNHEYFTGAVSFLHALDSGELVDTPAQRQQASYFCLRTADDGWQFLG

LDTGYHGHYLNVAAAAQQATLERLHIGRVETAGEGASPHWPTDRNPYFRHASLADLPVRDTTSPVDQVTV

RTDEAVWHLDKLASFPGRSILLSHHQLYSALDVCGIAQHRDASGTPDPADFNREWINTGLWRQFGPTFGD

RVAAWIWGHEHNLGIFADAYRPPDWPTEGDEAVRVFKTLPKGRCAGHSAIPVQAGEAPYAQKYPVALKQA

DLQLDLTDGWYNRGFQILELAGAGKPARLSYFQVAGADPTPLPLFVEPIS

>WP_024428798.1 pyridoxamine 5'-phosphate oxidase family protein [Burkholderia pseudomallei]

MKQPAPAECDAPPPVAPFHDGELAAQRRAGVAAAAAAFGGRGIRTFMPDQHRAFFAQLPLLVVGGVDGGG

QPWATLRAGAPGFVSAPQPRTLDIAGGTLRGDPLEGSWRIGSLLGGLGIELHTRRRNRVNGVVASIAGDA

MSVTVEQSFGNCAKYIQGRTPTPVPPQAGGAPPQRRAARLDAADRALLARADTFFIATANTSDAAGGARG

VDVSHRGGPPGFVRVDDDSTLTTPDYSGNNFFNTIGNLLHDPRAGLLFVDFERGDLLYIAADAQIVWDGP

ALAAFDGARRLVRLRIREVRRSAAVLPFRWSAPRFAPQFAAWGDAGARSLASRAESAAVGGSATALVSVT

QAASAGRGESAEWPESSVSLKVLKASEASEAPEVSETPETPGVAGACVR

>WP_024428700.1 transglycosylase domain-containing protein [Burkholderia pseudomallei]

MNGLASTLARRAAQAAVPRWVWPFARPAGWSLGSAFGSAFGSAFGSAFGSAFGSAFGSAFAAFGLALAPL

LAAPPVRPRARRNPRPCGSPRRAALPRARTTASRTLAVAMLAAPLAAHALPAYDDVRRDWRSTDWLLLAR

DGTPLQRTRVELTERRGDWIALADVSPAFREAIVVSEDKRFYAHSGVDWRGIAGAAWGNLWNERTRGAST

VTMQLAGLLSDTPRRSGQRSLPQKAAQAMNALRLERGWRKDQILEAYLNLVPFRGETVGLDAMSHALFGK

APSGLDVRESAVAAALVRAPNASAAKVAERACRILRDLRAAQPCESLDGYVRFVTSAPPGAARDGREALA

PHFARRVAAEVKPGAGARVRSTLDAPLQRFARDTLTRALAELNAPAHRRHVQDGAVVVIDNATGEIRAWV

GSSGALSAARDVDAVLAPRQAGSTLKPFLYAQALDERRLTAASLLDDAPIDLAAGGGLYIPQNYDKHFKG

WVSVRTALGGSLNVPAVRTLVLVTPHRFARTLTALGLPLAEEGDYYGFSLALGSADVTLLSLANAYRALA

NGGVARATLDVPGGSGSRNGNGNGNDAVTSAPFAPAVSKSKVGASAVFPRAVSTKAEPAKPVPTAALSPT

SASTSTAGKPAAHAAAGTRVFSEAAAYIVTDILADNNARVRTFGFDSPLATRFFSAVKTGTSKDMRDNWA

LGYTSRYTVGVWVGNADGSPMWDVSGVTGAAPVWAAIVGYLHRDLASRAPRPPAGVEQRRVAFERDVEPA

RPEWFIAGTALDTVRLAAPATAAAGGADRPRAPLSIGAPTDGTIFAIDPDIPPKHQRIWFERAAGRAARF

SWRLDGKVIGRGERLAWMPWPGSHRLELVDARGNVADAVGFEVRGAFAKTAAPPKP

>WP_024428622.1 acyl-CoA dehydrogenase family protein [Burkholderia pseudomallei]

MNMMKETVEANAPAAGAANIPDSRGINFFTSDPDCAALLRLHLGEARFRGFEPELRALGLRASDELDRLA

SRADRNPPVLQPRTRRGEPLESIDKHPDYVELERVAYAQLGLAAMSHRDGAPPPLVKYALTFLFVQAEFG

LCCPVSMTDSLTRTLRRFGSRELVERFLPRLASRDFDVLYQGAMFMTEQAAGSDVGRIATRAVRGTSADG

SATWRLFGDKWFCSNADADLAMVLARPEGAPDGVKGLALFLLPKTLADGSRNRYRIVRLKDKLGSRSMAS

GEIALDGAEAYLIGEIGRGFHQMADMINMSRLSNGVRAAGLMRRATTEALHVARHREAFGRKLIDMPLMQ

RQLVKMLVPTEQARSLFMQIALLLAKADAGDAQAGGCVRILTPLVKFRACRDARRVAGDAMEVRGGTGYI

EEWSDARVLRDAHLGSIWEGTSNIVALDVTRAVKREGALAPLRAFLLGALDTARLPGASATRLRRALDRA

SDAIAHVARTGDDAFVRQAASALYHATTAILLASEGMRLAPDHRRLALAHLVLRYKLSPVDPLATPRADA

DAEAEAEAGVLAALLHDRPVTLDDALRLLDDRSGDAA

>WP_024428555.1 glutathione S-transferase N-terminal domain-containing protein [Burkholderia pseudomallei]

MSDLSEFPIMKKWPAQHPERIQLYSLPTPNGVKVSILLEETELPYEAHVVRFDTNDQMSPEFLSLNPNNK

IPAIIDPHGPGGKPLALFESGAILLYLAEKTGRFIPADPARRYETIQWLMFQMGGIGPMFGQLGFFHKFA

GREYEDKRPRERYVGEAKRLLGVLDGRLAHRRWIMDDAYTIADIATFPWVRNLIGFYEARELVEFDRYPN

VARALDAFVARPAVARGLNIPARP

>WP_024428449.1 FUSC family protein [Burkholderia pseudomallei]

MRYSVEIRKFFYSQYFFGGLRIALGVSLPAVLSLAVLHDRELGFTIATGALGACVVDMPGPLKYKHNEML

ACSVIGFFSALATGLATPNIFALWLTIVPLTFVLSLIVVYGNRWPQISFATLFMMVMTLEEKFTPLQALV

NASWILAGGLWYTYWATIVSRWQARRIEQQALAESLFACASYLLARADFYDLDADLDECYRNLVARQITA

VETQETARDIVLRNLPKLRRGKLDPGRTMMFNLFINSVDLHEMFVGAHTDYPLVRSTFGGSDLMIFYRDL

IRKAAADLEEIGFAVLENRLPHSRISVKAELRAIEYEIELMRKKQFAATHPEAYAAVLATFRRIWSATRL

IDKMRRNLSGSADAQRTELKIDKALTRFLQKRRMSPLLIFSNLNMGSPSLRHALRVTIAVAVGFWLGRLL

PLTNAYWIVMTSIIILKPGYSLTKQRNAQRIVGTLIGCAASLALIYTVHEPHVLIAIMFGSMVMSYSLLL

FNYAASVVFTSSYVLLLFHLLAPGNMRIIGERAIDTVVGCMIAIAASRLFPYWEYRAIGKLVADALATTR

KYFEATWRAGRGKAAPAVPAADGAAAVPAVAAVVDAVAPGLDGDYQYRLARKNVHIAFANLGQAFQRMML

EPKAHQKFVPELNDLLVQTHVLGAQITAAAPLLRHACEQGNPPALERGMAAVREHLEQAEAGVPPPANQA

ETSKQLTRELDSMVVDAERSDAGADLAHDLKVLAHQCKQMLASSLLIRKDASVIRLPE

>WP_011203932.1 MULTISPECIES: carbamate kinase [pseudomallei group]

MRIVIALGGNALLQRNQPMTEVQQRENVKIAVAQIAQIAPGNELVIAHGNGPQVGLLALQGAAYPAVAPY

PLDVLGAQTEGMIGYLIEQEMGNLLPPDAPFATLLTQVEVDPADPAFEHPTKPIGPVYSRDEAERLALEK

GWHIAPDGDKFRRVVPSPRPRRIFEIRPVKWLLEKGTIVICAGGGGIPTRYDANGKLSGVEAVIDKDLCA

SLLARELSADLLVIATDVDGAYLDWGKPTQALIEAAHPDELERLGFAAGSMGPKVQAAIEFARQTGHDAV

IGSLADIVAIAEGRAGTRVSVKVDGIRYRRPR

>WP_009945729.1 succinylglutamate desuccinylase/aspartoacylase family protein [Burkholderia pseudomallei]

MTESAPVFPHYSIEVDFPDLEAHRTGNAGVDYVHRFDSGVPGPRVMINALTHGNEVCGAIVVDALLKRGL

RPRRGVLTVSFANVTAYERFDPARPDAARFVDQDFNRVWAPAVLDDLSQHSVELDRARAIRPFVDEADWL

LDLHSMHERSAPLIVAGPLAKGTALALRIGAPATVIRDEGHPEGKRMRDYGGFGDPASAKNALLVECGQH

WEARAVAVARDSTARFLMASGIVDERDLPADWFLPLATTMRIVQVTQPVVATSRDFRFADAYTGLEHFAE

AGAVIGWSDGKPVTTPYPDCVLVMPSLRQLHPGVTVVRLGRVEREVAQRAARGTRGKC

>WP_009944255.1 glutathione S-transferase family protein [Burkholderia pseudomallei]

MRRNSRKVAIVPPRARRAVEPALGRRRGGCAARTARNPQEDVAIAQGRSVRRAPAPLRARAVVGERVGQR

RAQMRVDGTRHTETAALLQRIGDLAGAGTLIPAHGTPARYEVIEWLTFVSSELHKTYSPWLFHADTADST

RRQCLEKLDRRLQEVDAHLATRDYLTGAFTVADAYLFAVANWSNFLRIPLAPYPHLVAFMARVGARAKVG

EALAAEGLGR

>WP_009939741.1 VOC family protein [Burkholderia pseudomallei]

MHAVSRRPSLSSAVFYRDPRAALEWLERAFGFARGIVVSDAQGRIAHAELSFGDGVVMIGAAGWSEFAFS

PESLDGKNTQCVHVQLESDLDAHCERARAAGAVILQEPADQFYGDRTYRARDPERHVWTFARTVRRVSRE

EAERHGGFSIEGGR

>WP_009936117.1 acyl-CoA dehydrogenase family protein [Burkholderia pseudomallei]

MDFRHTEDRRMLADSLNRFIAEQYAFPVRDRIALSPAGYSDDMWRRFAELGAIGALFPQECGGFGGAGFD

IALVFECLGRGLVVEPFVGALMAGRALAQAGGRAHLERLAALIEGRAIGAFAHAEPDTHYEPHTVRTRAA

RDGEGWVLDGAKAVVDCGEHATFFVVSARIAGGDDDAAGLSLFVVPRTAPGVTVRGYRKIDGGRAADMRL

ERAALADDACVGKPGEAADAIERAIGFGLLALAAEALGAMDVAKAHTLEYLRTRRQFGVPIGSFQALQHR

MADLLLEIEQARSAVINAAAQLDAPRVARERALSAAKYSVGRIGALVAEESIQLHGGIGMTWELPLAHYA

KRLVMIDHQLGDEDHHLARYAVLSRQ

>WP_009936116.1 acyl-CoA dehydrogenase family protein [Burkholderia pseudomallei]

MDLNFTAEEEAFRAQVQRFLADELPPRISRKVKGGLRLTRDDMREWHAILNARGWLASHWPREWGGPGWS

VAQKFLFDNECAIAGAPRIVPFGVNMLGPVLIKYGSPAQKRHWLPRILDGTDWWCQGYSEPGAGSDLAAV

STSAVRGVDARGDHYIVNGQKTWTTLGHYANMIFCLVRTATDVRKQEGISFLLVDMNSPGVEVRPIVTLD

GEHEVNEVFFTDVRVPAENRVGEENQGWTCAKYLLTYERTNIAGVGFSVAALDKLRAVAAKVTKNGRPLA

DDPLFAARLARVAIELDNMKTTNLRVLAAVAGGGAPGAESSMLKIRGTQIRQEISSLMRRAMGPYAQPFV

DAALDADDGEPPGGLPEAASAATQYFNNRKLSIFGGSNEIQKNIISKMMLGL

>WP_009922758.1 anion permease [Burkholderia pseudomallei]

MPLDRAVSNSVVGRAPARIIAFVRHEPVLSILVAALIALQAVHSLSAIALVRLVDWQTVATLAGLLMLTK

ALELSGFLMWLAHRIVHRVRSERALAALLVVFAAALSVWLTNDVALFVVIPLVLSLRELTPLPFKRLVIY

IALAVNAGAIATPLGNPQNLFLWQLSGVSFGRFVIALGPLALVLMALLLAMVVFAFDGRPLDLSRDSTER

PVDRAQAFATVAMFAGFVLLADAHHALAGVLAVAAVFLIVRRDAVLKIDWLLLLIFVLMFIVLRSAASLP

AVHQAIAGIGLDTPLRAYAAGALLSQIISNVPAAILLSEFTHDWRALAFGVSVGGFGIAFGSLANLIAIR

LSRARGMWLPFHLVSIPFGIASAVLGALLLHYF

>WP_009918092.1 phytanoyl-CoA dioxygenase family protein [Burkholderia pseudomallei]

MRYIKFAATIARRFSLRGMVASMSRTSVLQSFIDNNDADHPMSSLHPELIHTQVQTLRERGFVVAPGLVA

PERCAQLKTIAERQLREAAQPLEFEADLRYPGAPESRHAPGGHTVRRLLDAYARDAAFAERATAPEIGAW

MRAYFDETPVLSRAHHNCVMTKHPAYGSLTGWHRDVRYWSFERPDLVSVWLALGPETDDNGALWLVPGSH

GAEFGPERFDEAKFFRGDVPANRRLIEQAVCPALAAGDVVFFHCNTLHSAGQNRSDQVKFSLVFTYHGDS

NRPVPGSRSASKPEVRF

>WP_004555233.1 transglycosylase domain-containing protein [Burkholderia pseudomallei]

MVRFTLSHPHSPHRVNRRIVSSFLERCAAWLAAAKPHAVAVLHRLRHPTRRGVLLAVAALPALCVLYVLA

LIPFTPSIGDIRKARVDAPAQILSADGKLLAEFKPSNREWVPLADISPKMVDALISTEDHRFYEHHGLDW

RRTAGAALRTFSGDRQGGSTITQQLARNLYPDEIGRAPTLTRKLKEAITALKIEAVYSKAQILETYLNTV

PFLYNAYGVEMAARTYFDKSADQLDALDAATLVGMLKGNSYYNPVLNPERALARRNTVLAQMVKYGRLSP

AAYASLQKKPLRIDFERQKEPPGPAPHFAQQLRKWLIAWADRNDYNIYSDGLVVRTTIDSRLQTYATQAL

ARQTNQLQGVANGMWNAGSGCAPGNPLFRAFVRETPEFRAALDGGATGDAALKRLLADRGFARALCKAKA

DVQAGFLAIDPRNGQIKAWVGSRDFTAEPFDHVQQARRQPGSTFKPFVYGAAFAAGATPDDTFVDQPVEI

PLAGGEIWRPDDDAPPTNKPMTLRDAIAYSRNRITAQLMMKVGPQKVARLARAMGVRDSALDAVPSLALG

TSPVTLKEMVSAYATIANVGEYVEPRMVTRIEGRNGEVLAEFASATPERALDAAAARTLIDVMRGVVERG

TGAAIRSRYGIRADVAGKTGTTQGDTDGWFILMQPELVAGAWVGFDDGRVTLGGDWGQGARSALPIVGDF

YQRAIRARLVDTRERFATEAPPSAFDTFRNKLGDWYRYLFEKPEPPRKAAPPKAPRAPLEEVMPASEVEA

ASAAVARAASEAAAVASAASTASAAPPASGVPFAPGGASAPALPPLLPPMPQSAPLPPAAQPGSSLPNDN

APMSPTPTPDAPAAGGSN

>WP_004553202.1 MAPEG family protein [Burkholderia pseudomallei]

MNVSQTCLLITALMPFVWTMCAKSSSRYDNHDPRGYLARLDGWRARAFAAHQNSWEAFALFTAALVVAWH

NGANMQRVDQLAIVFVASRVLYGVLYLLNWATLRSLVWTVGLVCVVWLFFAAP

>WP_004553143.1 acyl-CoA dehydrogenase family protein [Burkholderia pseudomallei]

MNDPRVLVEMTEQAGRPPRTLAELIAALRASAPERDRAGGHAAREKRWIADAGLLTLAVPREFGGQEAGW

PVIYHTIRALARVDSALAHLLGFQCLQIVSVDVWGSAAQRERYLRGTVEHDWWWGNAVNPLDTRLVARAT

GDGGYRLDGVKGFCSGTRGSQRMTVSAHDSVTGKPVFAVVPTQREGIAVRDDWDPIGQRQTDSGSVAFDG

VRVAPDEVLHRSEAPPTPRATLRALVSQLVLTNLFVGIAEGALAEARDYVQRAGRPWLHSGVERAVDDPY

TLQRFGDMRVQTVSAEALADRAARALQGAWAKHEALTADARAEVALAISEAKIVAQRAALDVSEALFDAC

GARATAAPLALDRFWRNARTHTLHDPLDYRLRDVGRYALTGALPDASLYT

>WP_004551638.1 MULTISPECIES: cation:proton antiporter [pseudomallei group]

MNSIFVFLSQALLIVAVPPLLMNYLRVGAVVPLVVVQIVFGVVIGPSGLGRLSPETYTALFPPDSLTFLS

HVSAIALFFFAIVTGLHLDATGLRGHGRKLGFISTASMLAPMSLGVLAGIAISARYPQALDPHASVAEFV

AGIGICCGVTALPVLAAILRETNLTSRRLGQFSLALAAVNDGMLWILLAAFLSVTGANAANGGRLFITSA

LIVLYFVSILFVVRPLLLVLANRLRTSVDTQILIACAVAVASAAATDRLGLHYLLGAFIAGAIIPGAWRD

ALLERMQPVTVNVLTPFFFISTGLRVLIDVDAPGFLQITLIITLCTAIGKIAGTTLAARMTGESWGFCLG

LGFLAQAKGLMELVVATILLDSHVISRTVFSALILMALISTAFAMPLLRLKPVRGAALSA

>WP_004549996.1 aminopeptidase P N-terminal domain-containing protein [Burkholderia pseudomallei]

MNQPTEPALALDVYRQRRDRVLASLRAQGGGVAIVPTAPEVPRNRDSDYPYRHDSYFYYLTGFAEPDALL

VLDASAAGDAPRSILFCRAKNPEREIWEGFHFGPEAARDAFGFDAAFPYDALDAEMPRIVADAPALHYRF

GVSAAFDARLNGWLDAVRARARAGVAAPGAAFDLGPLLDDMRLVKDAHEQATMRRAADISALAHRRAMAA

CRPGIREYELEAELLYTFRRHGAQSPAYGSIVATGANACVLHYPAGNAVVADGELVLIDAACELDGYASD

ITRTFPANGRFSGPQRALYDIVLAAQEAAIAATRAGTQFDAPHDAAVRVLAQGMLDTGLVPKTRFASVDD

VIAERAYTRFYMHRTGHWLGMDVHDCGDYRERGAPRDDDGALPSRVLHPGMALTIEPGLYVRPGEDVPQA

FWNIGIRIEDDAFVTPTGCELITRGVPVAADEIEALMRDARPAPRPQP

>WP_004549943.1 MULTISPECIES: NAD(P)H-dependent oxidoreductase [pseudomallei group]

MTTILQINSAARSQGAQSTLLADELTAKLQQGNPGATVKVRNLLADALPHLDDAVLGAFFTPADQRSAEQ

NAIVAKSDELVDELRSADVIVIGAPMYNFGVSSQLKAYFDWIARAGVTFRYTSEGPEGLIKGKKAYVVSA

RGGKHVGMPTDSQTPFLKTFLGFIGLTDVTFVYAEGLALGPDAATEALASAREAIAAV

>WP_004548306.1 alkaline phosphatase [Burkholderia pseudomallei]

MSTIKRIAAAAFAAAALSGGFGHAAHAAGQAKNVIFFLGDGMGPATVTASRLYKVGEAGQLTMEKLPRTA

RIKTFSNDAQTTDSAPSMAAYMTGVKMNNEVLSMSPDTRAIAPGSDANGNKTVNRCGVGNGTPAATLLEL

AKARGKAVGAITTTELTHATPAATYSHICHRDAQYDIAAQAVPGGAGYNAALGDGVDVLMGGGRNHWTPY

DPLANRRGRADGRNLLAELQAKGYAVVATKDQLAQAGAGKLIGLFSTTSHLEYELDRVAGKGEGATQPSL

AEMTAKAIDVLRKNPNGYFLMVEGGRIDHALHGTNAKRALEDTVAFDEAIRTALAKVDLSDTLIVVTADH

DHTMTINGYSKRGNPVLDISRNYRDGQPNKDADGNPYTTLVFGNGANRPNARVPVDSSTATNDAYLQEVG

VRMGSAGSETHGGGDVMLFADGAGAKAFKGTLDNTKVFGLVKAAFGF

>WP_004548302.1 alkaline phosphatase [Burkholderia pseudomallei]

MKRVRLAALLGASVFAAAGCGSDEPKTPGASDNGAQTGAARNVIFFLGDGMGMTTLTAARIYALGEDGAL

TLDTLPETAFVKTYSNDAQVTDSAPSMSAYMTGVKTNNEVISMTPDTKAIEPSASLTGNCGANNGKPVPT

LLEIAKAKGLATGVVTTTRVTHATPAATYAHVCHRDAENDIAAQLVPGGAGYNAALGGGVDVVLGGGAQF

FVPKEAGGKRADGRHLVNELKAKGYAIAQNRDELLAADATKRGKLAGLFASSHMSYDLDRGATKEPSLAD

MATRALDVLQKNPNGYFLMVEGGRIDHALHDTNAKRALQDTVAFDNAIKATLDKVRQTDPELKNTLVVVT

ADHDHTLVLNGYAARTGKTESGKPGVLGVLRNYQTGAVAKDADGAPYTIIGFGNGENRVQGSRAGTSLTD

AVTGADDYRQEAVVRMAKGGETHGGTDVFLGAIGRGADGFHGVIENNKVFELVRGAAQL

>WP_004547404.1 class I SAM-dependent methyltransferase [Burkholderia pseudomallei]

MTTVTFSGPIFDLVDGVLRDAPEYRGFRAGAQSRAEFAAFLERVRTQLVGRGVPANDVHFAKRFPVSIDA

AVEHAFAELAPMGLAWPAGFPAVCARAAARIGRGFDHQGLGTYIYPEEGRLLLAIALAFRPRNAVFLGSY

YGYWAAWALPAIVAGGARAVLVDPDPRVAEVARRSLARLYPGARVEIVCDTGEHYLAGGGGPFDLVVLDA

ELPRDHVDPTRRGKGVYAHLLRAALPRLAERSLLVCHNILFRDHSGCAFFDDVIARNREELAPFLALVAR

EYDCFVECPTTEGVGVGMRTAPRGRA

>WP_004547274.1 SurA N-terminal domain-containing protein [Burkholderia pseudomallei]

MLDFFRNHQRLMMFFLLLIVLPGLGIVGIQGFRGFFDESANVAAVNGHKITRAEFDGTLRQQVDQARQVL

GAQFDAKAFDTPERRQQLLDGLIQQRALADETQRLHLSASDGAVRQTLLSDPVIASLKKADGSFDAERYT

QMLAMQGMTPDQYQERVRYNLALQQIPASIVSSAFTPKSVARRLTELAEQQREVQPMVLKSADYAAKVQP

TDAQISAYYDAHKQAFATRETATIQYLVYSQAAAAAAAQPSDVDIKKYYDDNIAHYRTDAQVRVSHIFIA

AAKDASAAEKAAAKAKAEQLLAEVKAHPDRFAQIAEKNSQDAPSAAKGGDLGFITRGSTAGGAAFDDAAF

ALKKDEISGVVQGDFGFHILKATDVKPAVVKRLAEVKDSIATDLRQQFAAKAFADNAEGFTSTVYEKAKS

LQPAADKYKLTIQTATVSPQPNPALPPDSPLNNAKFLAAVFAADSVKNGNNTQAIDIGNNTLIAARVTNH

QPSTVPALDAIKDQVRAKVVADEAARLAQQAGEAKLAELRKSKSTAGFAAPDKISRTQAHGLPPAAVSAI

YKVDPKTLPAYVGVDLGNDGYAIFRVNSVVAAAPADDQRLAAAQQQLAQVYAQSETQAYLASLRARSKVK

LYGSTAGGAQDSSN

>WP_004546718.1 MASE1 domain-containing protein [Burkholderia pseudomallei]

MNTKRSRPELVAALLWAALYLASGYLSHALNGPVRLTGYIWLPAGVTVGAFMLRPVREWPMLAAAFVAAQ

LALTGIEHGNPFNAALFAIDELGAAALAVGFVRRIRFSLEGLYFLRSVILAGVIASVLGALGGAAWYTVV

NGASFVDVGLVWAASDFIGVLLVTPVLASWSRFRAHRSGDHERFDLMLGIAAFALVAIGAFAIFDGDSAS

KFGIGAGFAMTYIPLFLTVAVTLLLGGRAGSSSVLVLALIVIMQTAQGEGPFASLDANHGRSLLEAQLYL

AVASLLVLTVSTLKTTRERVHEHAQVLRNNMELALASAGQIAYVLDPSSGRIDWSGDVERVFGVGVDAAQ

IASVPLVLERVHADDRDALRDYWRAEIAGEDRASLSLRVVQRDGGTRTITDHGAPLLDSNVDVAVVAGVW

QIERVWPADE

>WP_004546699.1 thioesterase family protein [Burkholderia pseudomallei]

MTTAGMQATELTVHRDTVRAEWVDYNGHLRDAFYLLIFSFATDALLDAIGLDDAARRARGRSVYTLEAHV

NYLREIKEGTRVRVDARVLAHDAKRLHLYLEMFAAGIDGAVSASEQMLLHVDTGGPKAAPFDADVAERIA

NLHALQRAHPAPAYAGRVIGLPARR

>WP_004546165.1 SMP-30/gluconolactonase/LRE family protein [Burkholderia pseudomallei]

MVETRRIHVVLLAAAITAIAAIAPAGHAQNATDWLANTYGTLAAHVGNTARSMWVAPEGVIYTASMWDEY

EGGVAIYQNGKSVGSIGSHAEFQGGAITGNAASVFVALQYDKSHGSGAVGRYNRVTKARELSIQVSASTD

QPRVDVVTGLATVGSLLYASDFYGNRVRLFTTDGVWQRDIGVASPGALAVDRAGNVWVARKRAGEIVEFS

AAGALLNTLRMPGGSQPSALYFDAPSGQLMVGDEGPDMNIKRYAVTGAPALVGTFGIQGGYLDTTTGIKG

QVGARRFTRVAGIGKDAAGNLYVLNNPWGGSWDLGRNGATDIHAYDSAGNLQWTLQSLNFEGIAAADPAT

DGALFYGGTHIYAGSAGGTFVANTVDPFSYPSDPRIDMNDTQRDEHFGQLVAVGANRILVASGQNPPIFY

FFHFNKANGYVAIPDASLPGAAFNTAQRVTSGFCIDSEGGVWAGLEKTGAIYHYPLAGFDASGKPTWGAG

VPTRIPASIQPLTRIVYLAESDTMMLAQGIVGSADWTSIGTRIEVYHGWRAGNTAAPDPVITLANAGAKS

IDAAGNHLFVGYWFGGGGPARPNVDAFNLATGKLDATLVNTSRATVDASSAVDSMYGVRAYQRSTGEYVV

TKNNVKGNSITVYRWKP

>WP_004544631.1 zf-TFIIB domain-containing protein [Burkholderia pseudomallei]

MKCPVCVTPDLLMTERQSIEIDYCPTCRGVWLDRGELDKLIARADDDASERRRDPARDAGRDAPPARDEH

DEHGRRRHDARERDASYRSQQGGYRKKKSLFDMFDFD

>WP_004543593.1 MOSC N-terminal beta barrel domain-containing protein [Burkholderia pseudomallei]

MPVISELFVYPIKSCAGIATVRAQLLVTGLEYDRNWMVTDPTGAMLTQRTHPRLALVRTAIGERELVVTA

AGMPELRTPLAASALAGAERLAATVWRDTVSALDTGTHAARWFSEFLGAPARLARFAPDARRVVGAKWTG

PFTSYAQFADGFPLLVVGQSSLDDLNVRLRRKGASAVPMNRFRPNVVLAGLDAYEEDYVDYLDVQTGGGG

VRLSLVKLCTRCPVPTIDQRTGAPDPAWPNEPTDTMSLYRGSKQFGGALTFGKNAIVLNGDGAFLEVGQS

VDAEIAFGE

>WP_004543427.1 MULTISPECIES: cation:proton antiporter [Burkholderia]

MPHDVSLIALLAAGFGLAMIFGYLASLLKMPPLVGYLLAGIVMGPGTPGFVGDLALAQQLAEIGVMLLMF

GVGLHFSLGDLLSVRKIALPGAIVQIAVATALGAGLALWWGWSVGGALVFGLSLSVASTVVLLRALEGRG

LIESVNGRIAVGWLVVEDLVMVLVLVLLPPLAALLGGGAPTHGDAAAHAGEAGGSLWAALGVTLLKVAAF

VALMLVVGKRVFPRILWLVARTGSRELFTLCMIAAAVGVAFGAAKLFDVSFALGAFFAGMMMRESEFSRR

AADETLPLRDAFSVLFFISVGMLFDPRVLIDEPLHVLEVAAIVVLGKTLAAVALVLAFRYPLNTALTVGV

SLAQIGEFSFILASLGRGLGLLSAEGQSLILAVALLSIALNTLLFAAVDPVLAWIRKRSAFARRLESRDD

PLAALPMSTPQAHLTGQVVIVGYGRVGTRIAHALDARGIAYVVVEQNRETVEKLRADGVAAVSGDAVEPI

VLVQAHIARAGMLVVTLPDVFDVRQIVDISRTLNPAIEIALCTNSDEEAALLANEGMGEVFVSETELAHG

MTEHVLARMGAGEAHARRSAAH

>WP_004541243.1 MULTISPECIES: flagellar transcriptional regulator FlhD [Burkholderia]

MEQQKDVFEEIAAFNQRYLRLVRRWLCEDAERARTVLGISGELAIRLAAMTPTQLEQLADSGELVCRLRA

DAMPGRA

>WP_004541076.1 transglycosylase domain-containing protein [Burkholderia pseudomallei]

MNRPLIRIALRPTGTLSFWKGFKWSLIVIVAIAVAIVARLVQIEIETSRLQARFLSELTRDVGYSVDEGA

SNRIRFPDNGPYDLRLGYALLPSFQQRLLSRGFVVASQARVSDRMLSLADERLFLPYGEKDQAGLSIVDS

TGSPLFGVVYPHHAYVDFDTIPPLVVQSLLFIEDRYLLDPSQPNRNPAIDWGRFSRALADQGLRFVNRHQ

ATPGGSTLATQLEKFRHSPDGRTATPPEKLRQIASASVRAYLNGPQTMAARHAIVVHYLNSVPLAARARV

GEITGIGDGLAAWYGRDFDEVNRLLAAPTTPENVAAQGVAFRQVLSLMIAQRAPSFFLNRGYPALQRLTD

SYLRLLSTGGVITPALRDAALAAHVERGAVPAAADTRSFVARKAVTSARAHLLGALGIDNVYQLDRLDLR

ATDTLNNGVQQAVAAGLARAATRDGAREAGLYGFEMLRSGDDPSKIQYSFTLYERRDGANLLRVQTDSVD

QPFDINQGARLNLGSTAKLRTVVTYLQIVSELHARYANLSAAELAKVKPDPTDALSRWALDYLAHTPERS

LRAMLAAAVERKYSASPGETFYTGGGAQSFTNFDKSDNGKILTVHVAFQNSVNLVFVRLMRDIVHYEMIR

SSGPSSSWLDDPAQRQRYLMRFVDSESRVYVKRFYTRYAGKPDDDALAAMLGTVRKSPPRVATVLRSVAP

EQPRAWFDAKMRAALHGTPAAAKLSDDALDKLYTKYAIERFNLNDRGYIAGVHPLALWTLAYLRRHPAAS

LDDVQKASRDARIASYSWLFKTRYHATQDRRIKRMVELRAYDAIGESWRALGYPFDHLTPSYAAAIGASG

DQPAALAKLVGLIANHGEQVPNERISSLEFAHGTPYETRFVRAAAQPRPLLSPEIADQVHMLLGDVVQKG

TGRRLAQGLAFPDGQTLPVYGKTGTGDQRFNVYARGARLIESRKVNRSATFAFSLGDRFFGVLTAYAHEP

YAARYDFTSAMAVQLLKSLAPALQPLMAAPAQPSANTGTAPGADETNTDGASAQTAERG

>WP_004538574.1 acyl-CoA dehydrogenase family protein [Burkholderia pseudomallei]

MNFTFSEEQQQFAHALRRYLDERYGFDARRAIIHSDAGVCDEQWRAFAELGLTALPVPEAHGGFGGGAVD

MLVATQELGRALVVEPYWATAVGIEALRVAGSADGEDAALLARAAQGDAKLAVAFHEPHARHDLFAIETL

ARPDGDGFALTGVKSVVRHGAQADVLIAPARLPNGAIGLFAIARGAAGADIVDYRTIDGQRAATVRFSDT

PARALVGGERDAAALERIADYGVVLLCGEAIGALDALNHATLDYTKTREQFGVPIARFQALQHRMVDMLI

HAEQARSLTYLAAVRYASDDANARRKAVSAAKARVGQAARFVGQQAVQLHGGMGVTDEVAAAHLFKRLSI

IETTLGDVDHHLARFASLPDFALLQDA

>WP_004535911.1 CopD family protein [Burkholderia pseudomallei]

MSHAIAVALFLHLLAVAVWVGGMVFANFCLRPALSDLSPQLRLPLVEAVFGRFFNWVAGAVIVILLTGGF

LLVKFGGAHATWPLHAMAGLGVVMMLIYGHIRFALFPRIRRAVQAQNWPDGARAVNAVRLLVMVNLVLGV

VTIGAAVLSRGF

>WP_004535594.1 MULTISPECIES: PilW family protein [Burkholderia]

MMRTTRWRAHTLVEVMIAMALGLLILLAAMSLYRVQRAAYSAAVDAARLRDAAQAALALISQQIQMAGFV

PLDAYDARAVPGLFGCAAGRPVGADGQTACDPLASRSDGLVVRYVGDGVSTWPTASGQPTDCLGQGVGAA

DTQPLIVNRFYARVSASTGEPELYCEGSGRPGIAQPLVEGVERLSLRYRLYGAARWADASALSVDDWANV

AVVSVCVQVRGRRTGRPARYVDCEGRVASAPDTRARLAWRRYVAVRNRAGT

>WP_004534140.1 membrane integrity-associated transporter subunit PqiC [Burkholderia pseudomallei]

MHAHFMSGSLSRRGRPALALAVALVMASASGCAGTPAALANIRYDLGPAQPAASSGTGPALKVLDVSAPD

ALNTDRFVYRLAYSDAQRIAAYRDSKWTAPPAQLLTQRLRGALSGRGAVLAADDGVRAPVLKVELSEFEQ

VFDGRSESHAAVTARVTLTQEGKVLGQRTFVSRAPASTPDAAGGAQALATASDALVSQLVAWLGVQAYAA

VQ

>WP_004533219.1 autoinducer binding domain-containing protein [Burkholderia pseudomallei]

MARNRTVLPLERAQVDVVQERSGVVGDRRISLRVCGEATQGYLLERYHAAPGEPSFVQRVNLFDDATIAS

FVEHDPYSAELESLYRAVIDVPKKALKFAGHETIQPEFASECVSESGLLTVMRNTILECGATNCFYHYFR

IDEKTGNLKNHELLIGGTPVWPHRYVHRHWYLNDPAMAHARNDTRPLRASTLAPLPHDHWLNQQAQFLGL

TSNVFFPAHRRDDDTIGLLHVSSSLPAAQGEEIIWRNRRTLRGLATEMLEWKVTAQRNTLAREMSLSHHE

LVALRLVGRGANARHVAEELKLQEHAVYQLFTSINKKMNSSHIRTSANKAKQFGFLAEGYISE

>WP_004532819.1 VTT domain-containing protein [Burkholderia pseudomallei]

MPIHAAHAPRRASRLSAAPMPPGSAFVAFEVRFVWHFPQAVPASLGPWAVFASVLVTQLGMPVPAVPMLI

VGGTMAAMGQASYASMLVAAVAATVLADSMWFFAGRARGRRLLNALVRFSLSLDTTLRIARKVFEKHGAP

LLVLAKFLPGLGLVSAPLLGTTAVAVWVFLFWDVAGASLWASVWLFGGAALHDEIVRLMQWVSASGGTLF

DAFAAIFVTFLLYRWAMRMRFRRWLAKIRISPQQLDAMLKSAAPPIVFDARPRAVREKEAYRIAGAYPLD

LDSPDPLHPDLMTRPIVVYCVCPNEATAKRIVSQLQRKRIRHALALKGGLDAWEKHGYPVEPLPADFDAA

RYAAPPPVERIAPVEPGGRDGGYPMRAGLTD

>WP_004532659.1 MULTISPECIES: type II toxin-antitoxin system ParD family antitoxin [Burkholderia]

MRTTRTLSISLPYEMADYIRAKVSTGEFATESEVIRDALRTLIARDRVVEAWLRDAVNSGEFALDPDAQW

AADKDEAKPARPRRRP

>WP_004531998.1 CopD family protein [Burkholderia pseudomallei]

MAMLWVKTFHIVLIASWFAGLFYLPRIYVNLAMETDPAARRRLLAMARKLLRFMTIIAVPALACGLWLWL

VIGIGQGQGWVHAKLGVVLLLVVYHAYCGHLLKVFERGENRRSDKWYRVFNELPVLGMLAAVALAVIKPF

>WP_004531397.1 zf-HC2 domain-containing protein [Burkholderia pseudomallei]

MDCNETRALLGADVDHELSAADAWRIARHVGGCGACRLERERLVALRRAMRQAEYHRAPGALRARIAAGL

PLAAAPFAQAPVQDLPASDVPVSDVPVSDVPVSDMPVSDVPVSDMPVSDMPVSDVPVSDVPVSDMPVSDV

PVSDAPEQDTPSQDMRAPNKPAVDRPPEAKPRFGADARGGARAGRWFARPGSRGPTLDRPGPGPRAAALP

GLGWGVALTVALAAAAGFALDARRAATEHAVDEIVASHVRAGLSSRDIDVISTDRHTVKPWFNGRLDFAP

PVVDLSASGFALAGGRLDYVGQRRVAVLVYRYRQHVIDVYVWPSGEGGARPYATVSQGYALDRWEAAGMT

WWAVTDAEPSALAAFRTALDARVAAPRTE

>WP_004531370.1 saccharopine dehydrogenase NADP-binding domain-containing protein [Burkholderia pseudomallei]

MKIAIVGAGLIGHTIAHLLRETGDYEVVAFDRDADALAKLANEGIATQRVDSADAAAIREAVKGFDALVN

ALPYYLAVNVAAAAKAAGVHYFDLTEDVRATSAIRELAEGSNRAFMPQCGLAPGFIGIAAHELVNGFTEV

RDVKMRVGALPEYPTNALKYNLTWSVDGLINEYCQPCEAVRDGRRQWVQPLEGLEHFSLDGIEYEAFNTS

GGLGTLCETLEGKVETLDYKSVRYPGHRELIQFLLEDLRLATDRDTLKSIMRRAVPSTKQDVVLVFVTVT

GVKHGQLVQDVFTRKIFAKEICGMPMSAIQITTAGAMCAVLDLFREKKLPQSGFVRQEQVPLHAFLANRF

GKLYEGGTLERMHALA

>WP_004531196.1 MULTISPECIES: MCP four helix bundle domain-containing protein [Burkholderia]

MKGVSLHGKHFLPDDATGNRGRTPKPGKPRRARRAWSVKTTLRAAFALLLVGTLAVGLFSLAQISRLNSS

IRSVYEQGHVASRAAEEVRASVLRASRAQKMLITATTAKERDELGADIDKGLAAIGAELATLQRHADGAA

DDAARLKAFGAAVGAWSAHLRDFVRLVREQPLDLSQMSWQVGSQDVSLLVETGKLEKLVDGLVDARGDAS

KATLDASGAIFRESFAMLAAMTAALVVLAFVIAAWVVRRLGAQLGGEPAYAKEIAVSISRGDLSNVIRLD

TRDHDSMLHALRDMQEGLAGTVREISASAEAIASAAGEIAMGNLDLSQRTEQQAVALERTATSMGQLTST

VHQNAENARQASTLAANASSVAEAGGTVVGRVVSTMNEIDESAKSIRDIIGVIESIAFQTNILALNAAVE

AARAGEEGRGFSVVASEVRSLAQRSASAAKEIKALIGASVERVANGAVLAQDAGRTMDEVVRAVKRVTDI

MGEISAASSEQSAGIDAIERAVTQMDAGTQQNAALVEEAAAAARSLDEQAQMLKEMVGRFHLPAHAAG

>WP_004531084.1 aspartyl protease family protein [Burkholderia pseudomallei]

MVRHAFASTPAPSRAASVSILHPRALASIALASAVALTAAGCGERAEPRRDGATPVLSASFRPKSGFVVP

VSIAGKTYHFLVDTGASHTAIDNRLAQSITRPSTDEQIPIAYRTMLEKGLTTADGVLPRERVRLWQPLPI

ALGSYQVPSFYPWLGLDLSLLSQVLGTQIDGIVGIEIFRQLSWVADNRSGTLTVWRHPPAAQRFAHCVPY

QDSFGQSPAVSVDFRDRWTMFRFDTGARYSIASAPTLAYLASHKAAMPLGGTVPSMSANGVGESRDHFVS

GLSFDGRPVGRLRVAEGGGDMLGMNFLARLDRYMFVPSTMEFCYDAGRFTQDDPQPLRTIAIRFVDGRVE

LFHNRPEDLRRYGLENGDVLVEIDGKRVEAPAIDDVRDRLSTAPAGSLDIVVERGGSRRAVRI

>WP_004530973.1 MULTISPECIES: patatin-like phospholipase family protein [Burkholderia]

MRLALVLMGGGARAAYQAGVLKGLAEIAHDVDPKRRTSPFSVICGSSAGAINATSLASHADDFEHGVRRL

LEFWEQLRAERVYRTDWLGIAAAGARWLAAMSIGWAARRSPRGLLDNAPLAYLLRRELDFHRIELMLEAR

KLHALSVTALSYSSGRHLTFYQASEPIQAWRRAERTARMVDLSAEHLLASSAIPFVFPAVPLVLDGQIEY

FGDGSIRQIAPLSPAIHFGSDRIVVVGAADPRPEVPAANGNGRGYPSLAQIGQQVLASVFLDSIGADIER

IDHVNRMIEHLPAYVEPESGWRHVDVLAIAPSERIELIASKHLKRLPLTVRGLLGAVGGNKPAGASFASY

LLFEAEFTRELVELGYRDAHGQRERLAQWIASAERRGGPAERGPGAGTDARTGAGAHAARRTTT

>WP_004530908.1 MULTISPECIES: peptidylprolyl isomerase [Burkholderia]

MKKTLRFAAAASGLVASLITVAPSASAQALRAQGASLADEVVAVVNNDVITGRELDQRVGLIARRLQQQK

APVPPTDQLRAQVLNQMVLERIQVQRAKDDGIVVDNATVQATLGRLAQANGMQLDQYKARIEAQGVPWDL

FVRDARTELMLSKLREKEVDSKITVSDAEVASYIASQRGPNAGSQQDLRLEHIFVKAPANAPQADIDVAQ

KKAEGLLQQALASGANFERLAKNQSEADDAKKGGDLGFKSPASLPSDVVDAVSKLRPGEVNPTLIRVPDG

FEIVRLVERRASQNPAASPKIVQTHVRHILLRVGEGKSESQARQQLIDIRRQIESGGDFEKFARTYSQDG

SASQGGDLGWISPGETVPEFERAMNTLQDGQVSNPVRTEYGYHLIQVLGRRDAEGSVQQQMDIARQAIGQ

RKAEQAYSDWLRELRDSSYVQIKLPVAQ

>WP_004530825.1 acyl-CoA dehydrogenase family protein [Burkholderia pseudomallei]

MDDLYTEDQRMILDAARAFSAEVLAPNAAQWDRESHLPDEIVAQMGELGFLGMIVPADWGGSYTDYVAYA

LALEEIAAGCASCATLVSVHNSVGCGPVLNYGTAEQKARWLRDLASGKTVGAFSLTEPHAGSEAHNLRTR

AELRDGKWILNGSKQFVTNGARAGLAIVFAMSDPDEGKRGLSAFVVPTDTPGFIVGKPEKKMGIRASDTC

PITLENCAIAQENLLGKRGEGLKIALSNLEGGRIGIAAQATGIARAAFDRARRYARERVQFGKPIAEHQA

IAEKLANMATRINAARLLTHHAARLRTAGLPCLSEASQAKLFASEMAEAVCSDAIQIHGGYGFLADYEVE

RHYRDARITQIYEGTSEVQRMVIARQL

>WP_004530039.1 MULTISPECIES: type VI secretion system tube protein Hcp [Burkholderia]

MANALVDYFLQIDGVEGESTDQQYPGLIQIQSWQWAEENSGRWGFGSGGGAGKVEMKDFEFRMVSNKASP

KLFLMCATGEHIQNAKLICRKSGKGQQEFLTISFASGLVSSFRTLGNMPISQLGHASGEVDGVLPTDQIR

INFAQIEFEYREQRNDGTMGAVIKAGYDLKQNAPI

>WP_004529963.1 DJ-1/PfpI family protein [Burkholderia pseudomallei]

MHIAILTFEGFNELDSLIALGVLNCVKKPGWRVSIASPTPRVCSMNGVAIDAQASLREANDADAVLVGSG

MRTREIVADAALLAQLRLDASRQLLGAQCSGTLVLAKLGLLDGVPACTDLTTKPWVQEAGVDVLNRPFYA

NGNVATAGGCLASHYLAAWVIARLEGRQAAERALHYVAPVGEKEAYVSRAMAHVTPYLAVSATAA

>WP_004529776.1 MULTISPECIES: alkaline phosphatase family protein [Burkholderia]

MKRNSKQFEIGAWLGACALAFAGAASAAAVQDRDHDSRPVDAKRVLLVSIDGLHEQDLARCIGANTCPNL

ALLAKSGVTYTNARTPGLSDSFPGLAALVTGGSPKSAGLFYDVSYDRTLYAPSDATCSGKQGWNVVFDET

TGIDAMNGGALTHLDGGGAFNPQAIPHARVNGQCVSVYPHDYVKTNTVFEVVKEHLRGSHTAWADKHAWG

YDWVNGPSGKGVDDLARTEINSIDPATGTPYTDIYTHTEKFDDYHVQAIVNQIDGKNSTGTAAAPVPTLF

GTNFQTLSVAQKATVASGGGYLDASFTPGPEVANAIAYVDGALGRIVAELRQRGLYDSTVVIVTAKHGQS

PTDHTKLVKHGDTLTALLEANGFVDPNGNFGQNNTASGNPNDGTGLVGTGFVQTDDVGLVWLRDPRQLSA

AVATLKANLGCNAPGICADGPQAYILYGPSVAERFGNPALGRTPDIVVQPNPGVIYTSSKKKDEEHGGNA

PDDSHLGLLVSYAGLRQGRTIDAPVLTTQVAPTILRSLGLEPRLLHAVALEGTRVLPGLGLER

>WP_004529763.1 AGE family epimerase/isomerase [Burkholderia pseudomallei]

MSAPVSVSDQAARLRRHFAQIVLPIWRGPGFNPALQLPFEAVAPDTHAPLPVTRYRAMACARQLFIFSQA

GDAQHAHALFAALCRHFRDPRHDGWFYSVDAQGAPLDRTKDLYTHAFVVFACAEYFAAFGNRDARELTQR

TAALIVDRFAPRPGSALLDSARGEDFAAAAGGPLQNPLMHLTEGWLAAGRAFGDTAFDDALLRTAQAVER

TFVDPHTGCVAELPIGCADNRFEPGHQFEWFYLVASAGARLAATGLPDALARAYAFAQRHGVDLDTGGVS

AATDERGACVDGTQRIWAQTEYLRALATHGGEPDALARQIARFAERFLHPRGWYECKTAQGEVSRADMPS

TTPYHLATAYASLPAGT

>WP_004529476.1 autoinducer binding domain-containing protein [Burkholderia pseudomallei]

MARQSTSVPFELSSAELARTRVGIVDGKRISLGVQGDALRGFVLERRCKSPGEPVSTQRVGLRDPAAVAA

FVEHDPYVVQLGIDYRALLDVHRAADDAGSHGAFAVHDARYARPASEAGGAFRPAEHAGAAPAASGVPTA

SAASVAQPEFAVECEHDGALLALMRRICASCGATQCFYHWFVVDEDTGEFTAHDLLIGGAPAWAQRYVHQ

HWYLNDPAVAHARDNTQPLRGSALAELRSDHWLNHYAQTQGLGSNVFFPAHRRDVSTFGLLHVAAPLPAP

HGEDALWRNRRVLRGLANEMLEWRVVRRRRELAQELSLAAQDVLALRLVARGGGARHVAEELRLDERAVY

QLFTAINRKMDSKHIKSSATKAKRLGLLAEGYISK

>WP_004529232.1 MULTISPECIES: FTR1 family protein [Burkholderia]

MLSTAVIVFREVLEAALVVSIVLAATKGVPGRAWWVSAGLLGGVVGAAFIAAFADVISAWASGMGQEVFN

AGVMFVATIMLAWHSIWMGKHGREMAQQLSQVGRAVAAGSRPLTGLAIVVGVAVLREGSEAVLFLYGIAA

GDPGQAPQMIAGGALGVLGGVGLGAGMYAGLLQIPLQRLFSVTNALIVLLAAGMASQGTGFLVSAGWLPS

WGDTVWDTSWLLKESSVVGKMLHTLVGYTARPAGIQIVAYVATLLVIVLLARRVARKQAIVARPTRAA

>WP_004529103.1 histidine phosphatase family protein [Burkholderia pseudomallei]

MTELLLIRHAQASFDAADYDCLSPLGDEQSARLGAWMARGARRPALIATGTLRRHAQTADGCARAAGVDA

PRLALAGLDELDADELIARHRPALASRDALLRAMAAEADPRRAFQTLFAAAVARWTGGAHDGEYGCAWPE

FRARTLAAWDALASQPAREIWAFTSGGPIGVIVAALFGVPAERSFELAWPLVNTSVTRIRIGRGGARVTT

YNGWPHLDGERDERLVTHR

>WP_004528939.1 glycoside hydrolase family 3 C-terminal domain-containing protein [Burkholderia pseudomallei]

MHAKRLSIAVLSATLCALAHAAGNDAPSPDIASRDAYALRRAHALVRQMTLDEKLQLIHSKYPMSDVPGG

GAGFIQGIARLGIPDLNMVDSATGSGSTSQPSTTFPATIGLAASWDKRLSYAFGAVIADQLRAQGFAMGL

GGGTNLAREPRGGRLFEYLGEDPVLAGEMLAARTRGTQDRKVIATIKHYVGNEQETNRMGGDDQIDERTL

RELYLLPFEIAMKAARPGNVMCSYNRLNGDYACENAHVLTDVLKNEWHFQGQVQSDWGAAHSTAKAINAG

LDEEEDVGPTVFLTPALVKQALANREIAPARLDDMVRRKLYAMIRTGVMDDPPRGGGTIDFAAANRFVQY

AAEQSIVLLKNQDRQLPLDAAGLKRIAVIGGHADAAVLAGGGSGNTRHPVTGAFPGCGGLTFPTTTGCNW

WPNPWLKLDVPIVQAIRDLAPGATVAFAGNSDRQSPFAAYTPQQIDAAADLARRSDVAIVFVTQAAGEDF

GELRSLALANPTNQDALVQAVAQANPRVIVVVESGNPVLMPWRDQVPAIVQAWFPGEGGGNAIANVLFGK

VNPSGKLPVTFPARDEDTPTWGADGTLAPNPVYSEKLKIGYRWYDAHRIAPMFPFGHGLSYTHFSYSGLE

VKQRPDAATTVSFALTNDGPVAGAEVPQVYLGDLDDPQEPPKRLVGWDKVGLRAGETRRVRIVIPAEMRR

VWDASRNGWALAKGGRIYVGASSRDIRLQQP

>WP_004528930.1 MULTISPECIES: RICIN domain-containing protein [Burkholderia]

MHFFRFAWRHGRYWLCLLIASVLLPQGAAAAVTASYTDIVGADSGLCVSTAGNSSASGAGVVQTSCAGLS

NTTWSFVPVGNRYHIVLQGSGLCLNVPGGSLNSGTQLIQYACQGNGQTNDQWTVVAVGSSYRIVSASSGM

CVNVSGASHASGAALIQYPCQGAGALNDQFNLYLPVVAATNVTAANSNLCVSVNGGSTAAGASIVQGTCS

NQGATGWSLLPAGSGYHVVSQGTGQCLNVYGGYTTSGAPIIQYPCQGDAQTNDQWTLVPVGSKYRLISVS

SGMCLNVSGGSLSPGAPLIQYPCQGANALNDQFSLSLPQTFPVTLPSAWSPVIPLPVNPIGIANAPNGKL

VMWSADQQLSFQNDVGSKATQTQTAVFDPATNTATQYLETSAGSDMFCTGTAMLPDGRLLVNGGDSSPKT

TLYDWTTNTWSAAATMNIARGYQGDTLLSNGSVLTLGGSWSGGQGGKTAEVWTNGGAWTLLPGVPETNIV

GPDPQGIYRGDNHLWLFAQGNGTVFHAGPSSQMNWISTAGGGSIQSAGMRGVDPFSINGTASLYDVGKIL

KAGGAKSYQQNGSVTTYASNSVYQIDITRGPNQPASVQRLNGMTYQRAFANSVILPNGSIVMIGGQSVPM

PFTDTTAIMVPEIWDPATQRFNLLKPMQTPRTYHSTAILMADGRVFAGGGGQCGTGCAMNHLNAEILTPP

YLLNADGTPAPRPVITNAPATAKLGATIAVSTQGPVASFVLMRLSSVTHTTNNDQRRIPLAIASSGGTSY

QLAIPADPGVVLPGYYMLFALNAQGVPSVSASIRIS

>WP_004528666.1 3-keto-5-aminohexanoate cleavage protein [Burkholderia pseudomallei]

MVKTFYITAAPVGAVPKFLDPLEPKFIPHALLELLPADAREATTQALEANGWEAVPAGGIVREYGYDAPI

DLTDYDGAQASASVQDALRNTGWTPCGTVWHRTQTSPSLAQPPLITRTTLERLSSVDLVRQIVLQLTTFG

WTATEDGSLTWTHERIHSYLSPDFVERMRADKAAVLESLFDNGWRVCGAGYWQPGKARSPYLPITADGIV

DASREALREGAAVVHLHTRATDDQATLAIPGLNTPIGIGSQRNHIVLDDYDRIVPTMLDLEPSAILNLST

SARGDRRASQSPLRRAHLKRYGHAQLAPDVASFSPGPVVFQAGGGYDNPNAFLADQLAHFAEVGVRPEIE

VFNHTIVENSVTLYQSPLVKAGVPVLFMLVAAVDQYHRDPVSGDTSDDSLIDVPTRKAIAKLLQAGTDDA

HEKAVELAATQLRPTVDKLRDNFPSCKISLLLPGPFQALLVDVAIALDLDGIRVGLEDALNVFDARVPGG

VRKACGTGDQVRWLRLELERRGIGIVDAEALRDELGMSRPDVALFRQAEAALAHYPADERLVSADTILDA

LRPIVDTYRKVEDRLATHLASAEALPADPAALAEHVLTAARSFGVTIRSFVEELDRYEDHEYLVARYIQV

PQALNFARELLVPRGYSIDAYDRALEDYARPGKTVTREHASYSVRVDQFKPLPLRCLEYLVGIPCRYNGD

YSNVVNLGLRQSPRYSATMALLYHALRELTLELRERSNASRKTCGPVWTVLETSANASEPPVRRDIAPDA

LTAAIDGVDWVVLPSTPTTNYPLGLKLANGMAQLFHGFVAQIAADPTLRPSRQTHRDTPLRLLAITHSGR

RDDGETVIEASMLHNRFALNADPSGIYFSEESQLIYERLILPRLVDKPAKLAYNERQLVRRDTAGFPLYQ

DGSRARRIKAEQIERLPFLKCFAHSSGIATAQQLDVQACRDGERLGLTADELRAFFDRALLVSFGSAADI

HLDWLGTSVVDVTAFNDVRSLAGTTSRHYLIQPGEHADVLQHCLVHTQPADYRYDHATPVWQEGRQGKVV

ARLTGVFLLDDHARLDDGHSIRRYLAASPLWLRQWIARFHDAPADAGAHAILRELQASMTDYRSSANQTT

RRALA

>WP_004528298.1 fumarylacetoacetate hydrolase family protein [Burkholderia pseudomallei]

MTSLTERIDAAARHLVAARRSRVAGPLLPAACRPDDIEAALAIQRRVAERLGEPIGGWKCALPPPGRIVV

APILASSIRAAGGPFPVVADARAVRIEPEIAFVLDRDLPPRARPYDEGEVRAAVREVRLVLEVLGCRYAD

PSRATVPELLADNQFNQGLCVGPIVSGGLRASLETIALECRGGVNRMLDGRHPDGHPLEPLRWLVNFLAA

RGESVRAGQIVTTGSYAGAIDAPLGQALTVRFGELGGLAVEWVA

>WP_004528124.1 acyl-CoA dehydrogenase family protein [Burkholderia pseudomallei]

MAISSPDSTLRRATAPAAPPQADADASTHAHDGKIADAALLFDDDPLIRRFAPLFERIAHGAAARDRDRT

LPYEPVEWLRAAGFTKLRVPRAAGGAGIGLAPFFALIARLGEADPNLPQILRVHGGFIEMLHESADDALR

SRWFARIAQGTIVGGATAERSAVTSNTVRLSRENGKLYLDGEKYYTTGTLYADWIDVSANDGDTDLRVLV

PAATPGVERLDDWDGFGQRLTGSGTTRFTRVEVEPGDIYRRFDASRPRGNSLLTAYFQTLHLANLAGIAR

AVLRDAVAFTRDRTRTFGVPGASSPRHDPLVQRVIGRLASLAYSTQSLVATIARTLDDVCAARAAGRATE

DAYVRVDIQAFQAQQIVLAQTLEAATLLFEVGGASATSETRRFDRHWRNARVLASHNPAIVREAVIGNYY

LNGVGHNERFGIARTGVR

>WP_004527775.1 polyprenyl synthetase family protein [Burkholderia pseudomallei]

MSSTATPSLSAAHLLAPIASDMEQVNRVIRQSLASDVLLINQIAEYIIGAGGKRLRPALLLLVAGALGEN

TNQKHVLAAVVEFIHTATLLHDDVVDESELRRGRKTANALFGNAASVLVGDYLYSRSFEMMVGVGKMRVM

EILSEATTIISEGEVLQLLNMHDADVDEARYMQVIRYKTAKLFEAAARLGAVLAGADAPTEAAAAEYGRR

IGTAFQIMDDWLDYAGTAESMGKNAGDDLREGKPTLPLIYLIERGTPEQSTLAREAIEHGGTDRFDTIFD

AITRSGALDHTLECARQEAQAAAAAISSFPSSNFKESLLELCSYSTSRQS

>WP_004527730.1 MULTISPECIES: glutathione S-transferase N-terminal domain-containing protein [Burkholderia]

MIDLYYWTTPNGHKITMFLEEAGLPYRIVPVNIGRGEQFEPAFLRIAPNNRIPALVDHAPADGGAPLSIF

ESGAILLYLADKLGRFIPADLRGRNEALQWLFWQMGGLGPMAGQNHHFVQYAPEPLPYAIERYVKETSRL

YGVLNKHLSDGRDYIAGEYSIADMASYPWIVPHERQRQRLEDFPFLAAWFARVAERPGTVRAYARAKEIN

TAPTVDQQSRGVLFGQDASTVR

>WP_004527331.1 FAD/NAD(P)-binding protein [Burkholderia pseudomallei]

MSTTTVAIIGAGFCGATLATHLLRRPPVRPMRVLLINRSGAMARGVAYGTRALGHLLNVPAGRMSAVAGD

DDDFYRYASGRDPRVARGSFVPRRIYGDYLEARLTEAIEQAHAGIEFRSVVGSAVRIAPVDGGARGAITM

DGGAVIEADRVVLSSGNEMRRDPFIAESQRKFYDSHAYVRDPWQPGALRGIAPDTPVLLVGSGLTMMDVV

LDLRARGHAAPIHVVSRHGLMPLAHREMDAPPSYDDRLAARMLARADVRHYVRAVRDAIRRGGDWRDVIG

SLRAATPALWRQLPSDERRRFLRHVRPYWDVHRHRCAPEPAARLQAEFERGGVAAVAGRVTGYSEHPNGV

GVTVRRRGAAVDERLEVGAVVNCTGPAPDFSARAGSLLGNLYADGLIVPDAIGMGFEIADDGAVLDRDGS

PSAWLRYVGPLLQARDWEATAVPELRQYVQRLADTLLAPRDERALT

>WP_004526892.1 MULTISPECIES: permease [Burkholderia]

MSSSRSYTPHPALGLATFVVLAVAGLFYVKWFPYYHKAFAAAEHHSIGQSILMGAAAHAPQPSLQAALDY

AWAYGKAIWQAMVLGLLLGSAVQALLPAHWVARALGGTGFGSVAAGGLLALPGMMCTCCAAPVVAGLRER

DASPGGALAFWLGNTVLNPAALVFMGFVLGWHWSALRLVLGVAMVFGVGYLVNRLAGAQPRVVDDALRAK

LVAEQAAVGNAFVRWMKIFARMTVRLVPEYLVLVLLLGAARAWLFPHIGPDIGNGVGWIVAFAIAGMLFV

IPTAGEVPIIQAMLSLGMGVGPAGALLMTLPPVSVPSLAMLARSFKPATLALVAALVVAFGVVGGLAAVA

LGF

>WP_004525545.1 MULTISPECIES: type VI secretion system ImpA family N-terminal domain-containing protein [Burkholderia]

MNNDALPGHSPDLLDFDEDFIKIDAAICEYDSVGYAPQRKGESAFQWASIETGCLALLKKAKDVRVGIWH

LRACIARRGLSGLADGVRSLADLMSAPVEELHPRALPDESPGETLLIHLGWLAGPQFLHQLGSSRFEDRD

ATLNDLIGGRAAAIVEDRDYRLRANTLVHDIQDSLSRIRESIAAAEQELNVSRALDLLSVAASRLTQAQA

GGADGASVESDAPVDAPAGASAPGAQQPMAGPGGVLRSRQEVGAALDRIVEYFRVHEPSHPAPIFLSRIQ

RMLGAGFEEVMAELYPEAASLVAQLSRPQSSK

>WP_004525235.1 FIST C-terminal domain-containing protein [Burkholderia pseudomallei]

MKGRSVVTMLSSTIPAVHSTCAHARDAVREVHAALANCDAELVLFFCSSRFDLDALADEMRERFRGTRVI

GCTTAGEIGPAGYRNDSLVAVALPRALFTVETALLEDLQTFTIASGHACALDALHDLERRAPRASGANSF

ALLLIDGLSVREEPVTRTLQGALGDIALVGGSAADDLRFERTAIFYDGRFRDDCAALIVASTALPFRTFK

TQHFRCGTERLVVTQADAERRTVSEINGLPAAEEYARLIGARVEDLSPGHFAAAPVVVLIDGTDYVRSIQ

KLNPDGSLTFYCAIEEGLVLRVARALDLVDNLQATFGDLRDSFGEPQLVLAWDCILRHLEMMQRGTRDTA

AEVLKANHAVGFSTYGEQYGGVHVNQTLTGIVFSRAPEPDRG

>WP_004525198.1 MULTISPECIES: GDP-mannose 4,6-dehydratase [Burkholderia]

MRPSEPGARRALVTGIAGFTGRHLAARLEAHGYDVWGTVAPGTDVLGDPLLRRWRCVKADLLDVDSLHAA

VADARPHAVVHLAARAHVAHGNPQDTYLVNVVGTRNLLASLAGLDARPHAVLLASSANVYGNASTEVLDE

SAPPQPANDYAVSKLAMEYMAKLWLDRLPIVIARPFNYTGVGQSEAYLLPKLVAHYARGEPRIALGNLDV

SRDFSDVRDVVDAYARLIDAAPVGETFNVCAERGYALKEVLAMLARIAGYVIDVSVDPRFVRASEVKKLV

GSRRKLRAAIGDARRTPLDETLRWMYGDMRAALGAREDQPAS

>WP_004524883.1 acyl-CoA dehydrogenase family protein [Burkholderia pseudomallei]

MQFTEEHEAISRTVKRFIEQEVNPHVDEWEEAGIFPAHEVFGKLGALGMLGLSKPAAYGGGGLDYSYELV

MAEALGVCAAGGVPLAIGVQTNMATPALARFGSDALRDEFLAPAIAGRQVSCIGVSEPGAGSDVASIRTS

ARRDGDDYVISGTKLWITNGTQADWMCCLANTSDGPPHRNKSLIVVPLKSKGVHIEKKIRKIGMHSSDTA

QIFFDDVRVPRRNLIGEEGQGFTYQMLQFQEERLYGAAAALVVLDRSIDETIDYTRARKIFGRPVLDHQV

VHYRLAELKTEVEALRALTYRATELYVQGGDVTTLASMAKLKAGRLAREVTDSCLQFWGGMGFAWESSIS

RTYRDTRLFSIGGGADEVMLGIICKKLGTLPRE

>WP_004524882.1 MULTISPECIES: enoyl-CoA hydratase/isomerase family protein [Burkholderia]

MTGTTFSTPAPLHVAQADGVRFATLNRPARRNALSDDLVAALDAECERAAGDAAVRALVLRGAGGMFCAG

GDFGGFSAMMREPAPAGEPDPIAAANRRFGALLEKLAALPVPTLAVVEGAAAGGGCGLAAACDRVLIASD

AQLSLPETSLGLPPAQIAPFIVARAGAARGCWLMLTAGRLSAADALDAGLADEIAGADGIDALLRRALAR

VLACEPAALRATKRIVADALRRERGAALDAASGEFAAALRSGAVAEGLAALSGKRAPRWASDAPLPPEAR

>WP_004524568.1 MULTISPECIES: acyl-CoA dehydrogenase family protein [Burkholderia]

MIPRTIFAEEHEQFRESVRRFIESEVMPHHERWEEQGYVDRDVWTKAAAAGYHCASMPEAYGGVGADIRY

SVVLFEEIARAGASGLGFGLHSEIVAPYILHYGSDALKARYLPKLASAEMIGAIAMTEPGAGSDLQGVRT

TAVRDGDHYVLNGSKIFITNGWHADVVIVVARTTPEGGSKGTSLFVVDTGMAGFSKGKRLKKVGMKAQDT

SELFFDGVRVPAGNLLGEENRGFVYLMQELPWERLQIAISAIASAEAALAWTLDYTRERRAFGRAVIDFQ

TSRHALAELKSEIQIGRVFVDKCIELQLAGKLDAATASMAKYWTTDLQFKVIDRCVQLHGGYGYMWEYPI

ARAWADSRVQQIYGGTNEIMKELIARTL

>WP_004524351.1 CesT family type III secretion system chaperone [Burkholderia pseudomallei]

MSSERYIQLIQDLCATVGLANVDNVLEARTIEVEGFDVRLDCFDSDAEAMYANFHFGTVTAGRTLTVFRL

MLEANLLVYAQDQAQLGIDTNTGGVILIIRLPFSLGIDGQGLADLLAHYAEHGRYWRQSIIESSDEMFEG

IASGEYFWLRA

>WP_004524162.1 MULTISPECIES: cache domain-containing protein [Burkholderia]

MRSLSLNQKLTSMIVILWIGLLLIGATGAWQNRSSMIADRREQLAYLTAQAYNVSEHFYKLSQQNAMPEA

DAKRAALEAIAAMRYGKDGYLSVNDSKPVVVMHPIKPELNGKDVSGFTDPGGKHLFVEIVKAGNAEGGLG

FVDYMWPKPGADKPIDKTSAVRHFAPWDWYLVTGMYMDDLQAAVLASTGRWLAMTAVLGAAATILMVLVL

RSVRSSLGGDLEAAVATAQRIAQGDLTAHVDIRGGDRKSLLHALHTMQTALIDMVSRVRLGTENINVGAS

EIAAGNTDLSQRTEQQAAALVETASSMDEMTTNVKQNADSASQAAELADQAAQVATRGSSVVDDVVRTMD

EITDASRKIGDIIGVIDGIAFQTNILALNAAVEAARAGEQGRGFAVVAGEVRTLAQRSATAAKEIKSLIE

TSNTTVEHGATLVTRAGTTMTEIVQSVRRVNEILEEISHASREQSAGIDQVNRAVGEMDQVTQQNAALVE

QAAAAAHSLKDQADALRSAVAQFALPG

>WP_004523875.1 PucR family transcriptional regulator ligand-binding domain-containing protein [Burkholderia pseudomallei]

MSLTISEILQLPGLEELQLRAGERSVQRPVRWYYVAENEGIADWVMGGELVFVTGINHPRDEANLLQLIR

EGAKSRIAGMVILTGEAFIRRIPDSVVALAEQLEIVLIEQPYLLKMVIVTQLIGTALARHENTLRSQRDI

VNQLLTGDYPSIDIAAHRARNLQLALDRPRRVVALRLAGVPALFEGRDPAAAEALLQDARQTVQRGLDDW

LRDEEGALPVVEQGELFVLLLPCDDPRFRKQKLALGALRDALNRQTGPLALFVGISSTVGAARHYCRGLA

EARQALGVAEGMRAGQGLCDYSELGVLKLLAAIPDPTLIDGFVKETLGNLLDSNRKHPTMLIETLEALLQ

ENGNAIKAAEQLSIHRNTLNHRLRRIETQSGQSLADPYFRLNASVALLAWRMSDTQRQEF

>WP_004523279.1 nitroreductase family protein [Burkholderia pseudomallei]

METSPLKTTTPEAIPPFDPSASVEDKLRLAIHYAILAPSSHNTQPWRFMVGDGSVMLCADRLRALSVVDP

YDRELLISCGAALLNLRVALSRFGFAYVIDMFPSPSDPDVIALVRLDPHGYHDESLVPLFDAILERVTTR

TPYANEAVPCDVQRALVDAGAAEGAEIACVDAPHALDEIAELIADADRLQFADPRFRRELANWVHPRRHD

DGMPAFAAGVPALLDFATPVVASAIRTFDLGGGLAAMHHKLVDGSPLIVGISTASDDRDAWVAAGQALER

VLLVAAAAGLTASYLNQPIEIDALRERLRPLLHVDAHPQLLLRIGRGPVVAHAPRRPLMDVVS

>WP_004523273.1 MULTISPECIES: TauD/TfdA family dioxygenase [Burkholderia]

MRIEPLTCAIGAELVGVCLADAAHDDGLFAEIRAALLAHRVLFLRDQDITRAEHVAFARRFGELEDHPVA

GSDPDHPGLVRIYKTPEQPNEHYENAWHTDATWRQAPPLGCVLRCVACPEVGGDTMWANMVLAYENLPEH

VKAQIADLRARHSIEASFGAAMPIDGRLALKAQFPDAEHPVVRTHPETGEKVLFVNAFTTHFTNYHTPAR

VRVGQDANPGAALLLGYLLSQAYIPEYQVRWRWRKNSVAIWDNRSTQHYAVMDYLPCDRRMERTAIVGDV

PY

>WP_004522404.1 MULTISPECIES: acyl-CoA dehydrogenase family protein [Burkholderia]

MHFDYSAKVEALRARLGAFFDEHIYPNERAFYEEIARNRRAGDAWRPVELIETLKAKARAAGLWNLFLPD

SARGAGLTNLEYAPLCEIMGRVPWAPEVFNCNAPDTGNMETLERYGTDAHKAAWLEPLLDGVIRSAFLMT

EPEVASSDATNIRTRIERDGEHYVINGHKWWSSGAGDPRCKLYIVMGKTDPDAPRHAQQSMMLVPSDAHG

VTVHRPLNVFGYDDAPHGHMEITLENVRVPASNLLLGEGRGFEIAQGRLGPGRIHHCMRLVGLAERALEL

MCRRASERIAFGKPVAAQTVTQERIAEARCMIEQARLLTLKTAYMMDTVGNKGARGEIAMIKVVAPNMAC

QVIDWAIQAHGGGGVSDDFPLAYAYASARTLRFADGPDEVHRNAIAKLELARHAPRSA

>WP_004522325.1 3'-5' exoribonuclease [Burkholderia pseudomallei]

MSASPPPHPAIDTPLAFVDLETTGGSAAEHRITEIGVVVVNANGVSTWTTLVDPQQPIPPFIQQLTGITD

AMVRGAPTFADIAGALFERLDGKLFVAHNASFDRGFLRAEFERAGIAFNPDVLCTVRLSRALFPRESRHG

LDALIERHALAPSARHRALADADLIWQFWQKLHAVIPAEQLSEQIVRTTRRFRLAGALTEAHLESAPAGC

GVYALFGDGDAPLYVGRSVRVRQRLRALLTGERRSSKETRLAQLVRRVEWRETGGELGALLAEADWIASL

APSFNRRSDRGATGDAHWPFGGPVAFEERGESRVFHVIDQWRYVGAASSIERAATLAADARAAGEGAGSA

APAVRRILQTHLARGLQLIPIPLAGAAPAAA

>WP_004521805.1 MULTISPECIES: nuclear transport factor 2 family protein [Burkholderia]

MEIDAISRALQIYFDVMYECDLEKFDLVFHPTSSLFTMKDGELSVRPFARYRSEIAARTPPKSVSQPRMD

AILQIAVLSPEIAFAQVRVRLFEKVFIDNLNLLKFDGRWMIVAKIFHHARTIAAA

>WP_004266825.1 MULTISPECIES: prepilin peptidase [pseudomallei group]

MILPLKLVASWTLASLALADLRTRRLATFAVALVGALYAALALVGAPGDGGFASHAALGAAAFALGAAMF

RAGWIAGGDVKLAAVVFLWAGPAHAWPVAFAIGVGGLAVGAVCIAAGRAPRVLAWFAPARGVPYGVALAA

GGLLAVWAPAACRLPGCLG

>WP_004204527.1 MULTISPECIES: homocysteine S-methyltransferase family protein [Burkholderia]

MSEPTPIAPFASSAAPAAPYTRGAALPQLLRQRILILDGAMGTMIQRYKLDEAAYRGERFKDFPRDVKGN

NELLSITQPRIIREIHDQYFAAGADIVETNTFGATAVAQADYGMEALVVEMNVASAALARESAAKYATPE

KPRFVAGAIGPTPKTASISPDVNDPGARNVTFDELRDAYYQQAKALLDGGVDLFLVETIFDTLNAKAALF

ALDQLFDDTGERLPIMISGTVTDASGRILSGQTVEAFWNSLRHAKPLTFGLNCALGAALMRPYIAELAKL

CDTYVSCYPNAGLPNPMSDTGFDETPDVTSGLLKEFAQAGLVNLAGGCCGTTPEHIAAIAKALAEVKPRR

WPSQYSEAA

>WP_004202957.1 MULTISPECIES: TauD/TfdA family dioxygenase [Burkholderia]

MSEIRDSITFQNAAALKAQARLGPGVICGLRSERAPLPLVVSPHGDSALAADRDAALAWFDARRAAFDAL

LLEHGGLLLRGFAVPDTHAFRALTDRYPPHAFGYIAGASPRKAIDGNVYESTHLPAPYKLSLHQEKAYMS

HYPRLIAFYCRQAAAVGGETPLSDMRAVTRRLPARTLERFRGKGVMYRRNFSAKPMPAHFNQFYRRWQDA

FMTDERAEVESLCRATQLEYEWLPDGSLTVTHVGPATVVHPRTGEEVWFNHASTQHINARVVHPTILRAL

QSFYKTRAALPYDIRYGDGTPMPAEDLDPVYDAIDAEETAFRWREQDVLLLDNILVAHGRNPYSGQRDIQ

VAMMD

>WP_004202590.1 MULTISPECIES: acyl-CoA dehydrogenase family protein [Burkholderia]

MSHAPADPTRSTGADYASLAARFRPIFARIAEGAIERDRTRALPHEPIRWLKEAGFGALRVPVHAGGAGA

SVPQLVQLLIELAAADSNLPQALRGHFAFVEDWLNAPPDAARRAWFDRFASGQLVGNAWTEVGDVALGEV

RTKVAKRDGGWVVNGEKYYSTGAIFADWIDVYAQRTDDGGPVIAAVAARQDGVILGDDWDGFGQATTGSG

TTRFVDAHVDEANVIDFARRFKYQTAFYQLFHLATLAGIGRAVERDASALVRGRRRVYSHGNAPRVSDDA

QILQVVGEISAWAYAAEAIALRAAQPSQRAYEARVGGDAAAEHDANVAAEIESAQGQLVVSELVLRAATH

LFDALGASATRATNALDRHWRNARTVASHNPLVYKARIVGDRAVNGTEPPYVWQIGAGPGGPREPEQAA

>WP_004200722.1 MULTISPECIES: chemotaxis protein CheC [Burkholderia]

MSETVLTAEQRDALQEIANLAMGRAAARLALLLGAFIELSVPRVRVVRAADVGRVLRDMTGIHDNVTAVR

QGFRSDIKGEALMLCRSAGVGQLVSLVDGAYADDEFGTVTQAELVFDMANVLMGACVSSILDELGRTPVF

SPPGLLGEDIALDDVFQPDVLAWSETLLLEVNFGVADDGARTHFVMLLAEDSIRHMSDALDLLLSSL

>WP_004200504.1 MULTISPECIES: glutathione S-transferase N-terminal domain-containing protein [Burkholderia]

MMKLIGSLSSPFVRKARIVLAEKKIDYKLELENVWAPDTNIHASNPIGKVPCLVMEDGAAVFDSRVICEY

VDTLSPVGKLIPPSGRERVEVRCWEALGDGVLDAAVAIRIEHTQREPQHRSDAWIARQQRKVDDGLVAMS

QGLAGKTWCVGNHYTLADIALGCTLGYLDFRMPEINWRERHPNLDKHFAKLAQRQPFVDTVPQ

>WP_004199551.1 MULTISPECIES: sulfotransferase [Burkholderia]

MSASAPASRSSERRPVRVLRVLARRHVDSTRMVRPRDFDTALVAQVPGMSDIGDGERYVPLCVDWRDARL

FLSRWDDDCAMTDVPFLYQRQRRTARQLLDVPFEQLEAPGRAARMTPIFIFSVGRCGSTLLSRLLAAVGE

QAVSEPDVLTSVAHFDDAAERAAALPARERIVQSCVAAFEPACGPAPIIKLRARCNRAVDVFLNAMPHAR

YVFMCRNRDDWVRSSSRAFGDSGEALAELLKASVEAFDRMHAARVDPLLVWYEDLLADPLAALRRILRAR

DDLDAHRAAVERALRADAQEGSGLSRASLAARTGDAGALAAFDARWREIRPEALLREHGLARLR

>WP_004198768.1 MULTISPECIES: CoA transferase [pseudomallei group]

MFDLAASSHTEPSGHARAAAYLRDIWQAANGDPEWLRTLAFGGAGVLPSAFPVTDFASAAIGAASVALAE

FVHRATGRLPGVRVDRRYASIWFGTSLRPRGWNMPPLWDAIAGDYRTADGWIRLHTNAPHHRAAALAVLG

APPERQAVARAVGQWRGDALESAVVEHGGCAAVMRTEDEWARHPQGRAVRAEPLMIHDDVSIDGPRPAWP

MSAERPLRGVRVLDLTRILAGPVATRFLAGFGAQVLRIDPIGWDEPGTVPEVVLGKRCARANLACADGRA

LLRRLIGEADVMVHGYRPGALDRLGFDADERRRINPGLVDVSLDAYGWSGPWRGRRGFDSLVQTSAGIAA

AGMREAGADRPVPLPVQALDHGTGYLLAAAAIRGLVRRLATGAGTSTRASLARTAVLLTSGGLQEAGRQP

IAPETAGDLDAWIEDTSWGPAQRVRAPVTIECAPVQWPHPARALGTSPAEWA

>WP_004198629.1 MULTISPECIES: dienelactone hydrolase family protein [pseudomallei group]

MASQWIDIPAGNDTFGGYLALPKRGKGPAIVIIQEIFGVNGHIRSVADQYAADGYVALAPDVFWRTQPRI

ELGYEGADRDKGIELLQKTDVAQAVADIGAAAAALRARPEVAGKLAAIGYCFGGRLAYLAAARQHVDAAV

AYYGGGIQNHVDVAAQIAQPILFHYAGHDQSIPLDAVDKVKAAFAGRANAEFHLYPDAQHGFNCSERASY

DQRAAALAHGRTLTFLAERL

>WP_004198263.1 MULTISPECIES: enoyl-CoA hydratase/isomerase family protein [Burkholderia]

MSAHERVVEVREVADCVVQVVMQDRENKNTFSDALIRGLGEAFDAIRQNGRCKAAILTGYGNYFASGGTQ

ESLMYLSEGKGTFAEIAASASRNGANLYSLALNCPVPVIAAMQGHGIGGGFVMGLFADFVVLGKECLYTT

NFMKYGFTPGMGATLVLPHKLGLPLAQNMLMTARSFYGDELQKLGVPFPVVPRAEVAACALEIAKNLAEK

PRLSLVTLKDHLVAELRSRLPAVIEQELAMHEKTFGQPEVKERIRSLFGQ

>WP_004198111.1 MULTISPECIES: RcnB family protein [Burkholderia]

MKAHRTLRISMLAAGALVSSLAAAQPHGPGGPGDERHGPPPGKHVGRDHGKHGDDGARPGEHRGDENAAW

RRGERLPDEYRDRQYVIDDWRGYHLSPPPRGYHWVGIGGDYLLVRISTGVILQIGP

>WP_004197812.1 MULTISPECIES: CreA family protein [Burkholderia]

MKNRYFAALATCAALLPIASTARGEEVASVNTNFRLTGSDRVVVEAYDDPLVSGVTCYVSRARTGGIKGT

LGIAEDPTEASIACRQVAPIRFTEPLRQQTDVFSERLSFIFKTLHVVRVVDKKRNTLVYLTYSDRIATGS

PKNSVTAVPVPAGTPIPLR

>WP_004197761.1 MULTISPECIES: VOC family protein [Burkholderia]

MKFHIHEIDHVVLRAADLAAMTRFYCDVLGCHVEKEQRELGLVQLRAGRSLIDLLAVGAPIDRAGSGAPG

KGRNMDHLCLRVEPFDAAALRTHLAAHGARPGDEARRYGADGYGPSLYLFDPEGNMVELKGPPEAVAP

>WP_004197265.1 MULTISPECIES: flagellar brake protein [Burkholderia]

MNTEQSTSPAASAAAHSGHDYGRRNPLEIGVQLRNLVNRGDFLTVQYQGGQLVTRILDVDVGARTFVFDW

GALADQNAGILAAPRCVFDASPDGVRVEFSTATPRETRYENLPAFEADFPDVLYCMQRREYFRVDAPILD

PYVCRGRLPDGETFLFEVHNLSLGGLGLRTSDDRVASLEPGMTLPDVELNLNGHGMLSLDLQLVSHRASE

TPSGARRYQLGFRFVSLPGSAENTLQRIITQLEMKRRQLARA

>WP_004196743.1 MULTISPECIES: transposase [Burkholderia]

MARLARLYVPDQPQHVILRGLDQQPAFVDDQDYELFIDCLKAAARDHHLAVHAYVLLPRQVQLLVTPSDE

ASLPKAMQAVGRRYVAHFNRRYSRRGTLWEGRYRATVIEGERYFLLASRVVEMSPVRAQLVTSADAYRWS

SYRHHVGLTVDSLITDHPLYWALGNTPFERQRAYKELCEQPLDERQADQLQQATLKGWVLGGETYREWAA

RTANRRVSPLPRGRPRKVRENTPPIQQ

>WP_004196422.1 MULTISPECIES: enoyl-CoA hydratase/isomerase family protein [Burkholderia]

MSAELLASRPPESESTLVLTLSNPGARNALHPDMYAAGLEALNTAERDPSIRAVVLTGADRFFCAGGNLN

RLLDNRAKAPSVQAASIDLLGEWITAINTVTKPVIAAVEGAAAGAGFSLALACDLLVAADDAKFVMSYAR

VALTPDGGGSWFLARALPRALAAEILFEGKPAAATRLHQLGVVNRLTKPGTTRDEAIAWADQLGTMSPNA

LARIKSLIADAQTQPLAAHLAAERDHFVASLHHADALEGITAFLDKRAPIYKR

>WP_004195784.1 MULTISPECIES: cation:proton antiporter [Burkholderia]

MKSAFSFLPNWPLAPDAIFWAGFALFAAGLCGELCYRAWRLPRITGYAVIGLVAGSFGFGVIDASTDDTS

RLLVNVALGLLLFELGSRLDLRWIRRNPWLIASSLAEATLTFVLVLAVLLLLKVPGMIALVLAAIAISTS

PAMVIQLKTELRAEGQVSQRLITLSALNSVYAVVLTKLVTSWLHQEAYGNVFATILQPIYLLAGSFIVAY

LFARACNYLFRHVAATMRDEHSFVALFGLVVLAIAVAQVLKLSTMLTLLLAGIIVKNLEARPQLWPEHFG

TAGWLLTVILFVLTLTSFEGQDIAAGGLIAGALIATRFLAKLVGVLAFAKPSGLGVKQGIALGVSLVPMS

ALAYLLVDDTYQLYPNFDPRLRAVVMCSIVVLQLIGPLVVYRSLSAVGERRDAS

>WP_004195489.1 MULTISPECIES: GreA/GreB family elongation factor [pseudomallei group]

MRNRIYQLTELDVARLEKHAERNPRYQEMLDTLLERADIVEPNKIQANVVTMNSQIKLLDETAGQDRAWT

IVYPDAANFEQGRLNVFSPVGMALLGSRCGERVKVTLPGGADATLKIVEIVYQPEASGDYAH

>WP_004195230.1 MULTISPECIES: LON peptidase substrate-binding domain-containing protein [Burkholderia]

MSSLSTTLIDLPLFPLHTVLFPGGLLPLKVFEARYLDMARACLRDDAPFGVCLLKSGPEVAQEGEVSVPE

TIGCMARIVECDTGEFGMLLLRTIGTQRFELLSHRVEANGLLVGIAEPMQEDIPLEGDSALAQFGACAEA

LERIVEVLRRSDAELPFAEPFRFDDPTWVSNRLAEVLPLDLRARQKLMEFPDVGARIDAVHRELNRHGWL

>WP_004195164.1 MULTISPECIES: carotenoid oxygenase family protein [Burkholderia]

MTTIDLNAGALAPVADEIDAVDLRVTGALARELNGVLVRNGPNPLRGRFDGGDVLSWWPQDAMLHAISFD

DGRATRYRNRWARTRRWARVHDPAREPSLVDTNPNVNVLAHAGEILALAEGGAPLAITAGLDSIGAARRH

PGLAHGMAAHPKVDPHTGELIAFRADWNRPWLRYLVADAAGAQTVDTEIALPAPSMMHDIAITATHSIVF

DLNVAYDFSMLSRGHRMPLRWHDARGARIGVVPRRGGDARWFDIAPCFIQHVVNAYDLDRPAIVLDVVRY

PWFLRVARDGRGFDDNPPGVLWRYVIDLVTGTVAEQPLDDAGIELPRIDARRTGRRHRFLYAAEQPTPVE

LRGIVRYDLDGGSTQRYRVPPGDQNSEPVFVPRPGAAGEDDGWLLVCVYRHATDTSDVVILDGRSIGDGP

IATVHLPRRVPAGFHGAWLPAGA

>WP_004194596.1 MULTISPECIES: autoinducer binding domain-containing protein [Burkholderia]

MTIADSAPRIEWFSHAELAASRGGFAPRDTQPSVSSFAAPPDGPRRATFDVADCDARLRAAGFTTLCYGA

FELIGQQMLAAYLLRDFAPASFLRAYVEGRLFEVDPRFAEWRQGVSPIAWRLDELDAHARRTGERRMHAL

VNSLRAHAMHSGVIFCWPVPHLDLRVAVSLASEAHDGSLDDRVIGCALALSLDVHRAAQPHLDARVGGAR

TFVLERDEAAVLERLVHGLSDQEIANALSTSLHRVGAQIRTLEKRFNARNRAQLAYLAARRLRG

>WP_004194375.1 MULTISPECIES: VTT domain-containing protein [pseudomallei group]

METLLHFVSLVVHIDAFLGDFIRQYGAWVYLVLFLIVFCETGLVIFPFLPGDSLLFIAGAFAATGEMTLA

GLLVLLLVAAVGGNTVNYLIGRAIGPKVFNTHIPGLERFLDRAALQKTHDFYERHGGKTLVLARFIPVVR

TFAPFVAGASAMSVARFQLFNVIGALIWVLLLVFLGYFFGNIPFIRHYLNVIVLVGIGAAVIPVALGALW

KLLRRKSDARKTQANR

>WP_004194030.1 MULTISPECIES: glutathione S-transferase N-terminal domain-containing protein [Burkholderia]

MLRFFYHPSPNPAKVALFLEEAGVPYEFVPVDTRKGEQHSDAYKAINPNAKTPAIADGDAIVFDSNAILL

YLAEKTGQFLPDDTPAARGELLSWLMFVATGIGPYCGQAVHFKHFAPEPKAYAVNRYDFEAWRHWRIVDE

RLAGRRYMVGERYTIADMAVWGWARAIPRVLGDDAWAQLPHVKRLVDEIDARPAAQRADALKARHAFKVE

FDDEARRALFPQNARLAATAGTGA

>WP_004193694.1 MULTISPECIES: antibiotic biosynthesis monooxygenase [Burkholderia]

MSEVAVVALIVAKPGAEAKLLEQLEGIVGPTRSEEGALQYDLHRDLQEPRRFIFIERWASEAALAAHARS

AHIDAYKRAAADWIESSEIRVASKVA

>WP_004193409.1 MULTISPECIES: SCP2 sterol-binding domain-containing protein [Burkholderia]

MNAPVFQLPPTLGKLLSKLPAYPGALLFASGINLVLRRHLPAETLALLEDRPLRIQVKDAGVAFDFVCRR

GAFSALSGGRDVDLTIGATAYDFYLLSQRREDPDTLFFSRRLTMEGDTELGLLVKNSLDAIDLSVFSLER

WLPTRLFRQRPLERDAMPD

>WP_004192642.1 MULTISPECIES: pilus assembly protein [pseudomallei group]

MNERRAAFARRRERGAVVLWFVLFLPVLLLFGAFAIDLPRVAAARNELQNAADAAALAGAASLEAGAGAP

AWAAAASAAAAALSLNASDGAALSSGDVQTGYWNVTGVPAGLEPTTLAPGEYDVPAVQATVTRAPNQNGG

PLSLLMGGLLGLVGTPAAATAVAVAGAPATVGAGGLFPMVIDQCVLDQYWDARAGAPRVDPTTGAPYEFQ

VGNGRTYGGTCYAGQWTTFLVNANDVPTVRGLMAHGNPTPLSIGDSIWIEPGVKTALYYDVPVGVTVVVP

VATQISSKTYVPVVAFAAFYVDASDGANLKAITGHFVGGYKIPASASGIGPAYGAYVAPRLAY

>WP_004192266.1 MULTISPECIES: 3-hydroxyacyl-ACP dehydratase FabZ [Burkholderia]

MSTEKINFDIHKILTLLPHRYPILLVDRVLELEPHKAIKALKNVTVNEPFFTGHFPKRPVMPGVLIIEAL

AQAAALLTFAEAQPKDPENTLYYFVGIDNARFKRVVEPGDQLILNVTFERYIRGIWKFKAVAEVDGKVAA

EAELMCTVKTADAAP

>WP_004192100.1 MULTISPECIES: tryptophan 7-halogenase [Burkholderia]

MHLPNRTQVLVIGGGPAGATGAAFLAREGVEVTLVDKEVFPRYHIGESLLPSCLEILTLMGARDTFDRHG

FQRKPGAYFNWKGETWKLDFGELGGTYRYSYQVRREEFDHLLLQHARAVGAQVHEGVSVREILFDDGRPC

AALCVAQGAEEATTVEFDYLVDASGRNGLMSTRYLDNRKFHEIFRNVAAWGYWEGLSWPDDCAPGSILVS

SIPDGWWWAIPLADRPTSVGVVMHRDAFVAARRTGTLEQVYAQALALSPVMANLTEHARLVTPLKTEQDY

SYTCDSFAGNGYFLSGDAACFLDPLLSTGVHLAMYSGMLAAASLASILRAEVTEREAAAYYRDSYRQAYL

RFLVFVQTFYEAHGKLGYYSKADELSHYMIEAGDIRRAFLNLVSGLEDIADAEQATSHLMGEMSRRIDQN

LALRKDKRALSSAIGSTQVEDNARFFDAIEGLPCLSANMALDGLYVSTRPRLGLQRVAAM

>WP_004191996.1 MULTISPECIES: autoinducer binding domain-containing protein [pseudomallei group]

MRAAMGNWAEDLLAGLDSARSEEEAFRSVETAAAALDFEYCAYGLRVPWPLSRPRIETRSNFPEQWKRRY

VEAGFLDVDPILAHGRRSQQPVVLAETLFASAHQMWVEAQSFGLRFGWAQSSFDAYGGMGMLALVRSREP

VTAAELDAKEYRMRWLVRTAHAALGRMMLPKLMADPERGLTEREVEVLKWAADGKTSGEISKILAISVDT

VNFHVKNAILKLRTANKTAAVVRAAMLGLLS

>WP_004190545.1 MULTISPECIES: acyl-CoA dehydrogenase family protein [Burkholderia]

MDFSPSARCRELSERIAAFMRDEIAPVEARYAEQLTGDADWRRWRQPDVMETLKAKARAAGLWNLFLPEA

EHGGAGLSNAEYAPLAELMGHSFIAPEAFNCNAPDTGNMEVLARYGSPEQRRRWLEPLLAGEIRSAFCMT

EPEVASSDATNMRATAKLEGDEIVLNGRKWWSTGIGHPLARVVIFMGLTDPQAEPHRRHTMVLCPLDAPG

VRIERMLPVFNAYDEPSGHGEVSFTNVRLPASNVILGPGRGFEIAQGRLGPGRIHHCMRALGAAEKALTL

LCARATARTAFGKPLVKLGGNGDVVANLRMAIEQARLLTLKAAWTIDTQGVKAALSLISQIKVVVPAVAQ

QAADAAIQIHGGAGLSNDFPLAALYAYARVLRIADGPDEVHRAVVAKLEVKRQLAAAEAA

>WP_004189882.1 MULTISPECIES: NAD(P)H-dependent oxidoreductase subunit E [Burkholderia]

MSPHSVAPDALVRAHAQPGRSLVAILHAIQDDAGYVPPACVEPLAKALNLSRAEVHGVLTYYHHFRTAPP

AHVTIRLCRAEACRSMGGEALVAHAQARAGCRIDGGHGDRVALESVYCLGLCAQSPSLTINDEPHAKMSP

ARFDALFDAAVRTKEPA

>WP_004189874.1 MULTISPECIES: Spy/CpxP family protein refolding chaperone [Burkholderia]

MKIERYSKGLTVISLTAALWSLGMPVAPAAPASDARAQTPCGRGYGMGPGMMGGGYGRGMMGGYGMGPGM

MRGDGMGPGMMMGFGGWPSGLDLTSEQRAKINRIQDDTRKAHWALMGDMMDQQAKLRDLYDAPKRDGAAI

DETSRAIGSLRQKMIDASVDARKKMEAVLTSKQLDKLRAYEKQADDMSW

>WP_004189725.1 MULTISPECIES: helix-hairpin-helix domain-containing protein [Burkholderia]

MLKKLLATAALCAAAAHGWAAVDVNAANDDALRGIRGIGPAKAKAIVDERSAHGPFKDAADLARRVKGMG

GKTVERLQAEGLAIGAARAPAAAGAPQKAAAAGKK

>WP_004189074.1 MULTISPECIES: CopD family protein [Burkholderia]

MKLDSLWFGQAALAALGDVAFAVALGSAFIGAWLANDGARSVIAPSHPAWRQSLRSLAVAAAVLVLADAG

WLVYQAASMSGAGLRGAFGAMPTVLAQTHVGHAWAVACGGALLLLAVALARPSGPLAGALLALATLVVAA

GKASLGHAADSGAFSAAVGVQTVHVAATAVWGGLVIAGGMAVLPALGSSIARGAMIRIAQRLSGASIAAL

AFVIVTGALNTVRGTGGDFAALDGSTWGRVLLLKLALVALALVLAALNRFSALPRLRRTASTEDAHTFRN

VLHLEALAMIGVFIAAAVLSSTAPIAAG

>WP_004188348.1 MULTISPECIES: VOC family protein [Burkholderia]

MSVIGLDHYNLRAPRPLLDTLRDFYIDVVGLRLGARPPFRSHGYWLYAGAQAVLHLSQAGPDETRRANVV

NTFDHVAFSCDDLPGTLARLQRFGIRYSSADVPLTRQHQLFFDDPAGNGVELNFAVRDDG

>WP_004187469.1 MULTISPECIES: TraR/DksA C4-type zinc finger protein [Burkholderia]

MAAFDEHQLQVLGALLRACRQALQADVRAGERQRADEPYANLAGPAPDEGDEANADLFVDVDHALLGMKL

AELRDVDAALQRIARRDYGCCADCGQPIAYERLLARPTALRCAPCQRAHERRFATQPRPSL

>WP_004186084.1 MULTISPECIES: Smr/MutS family protein [pseudomallei group]

MAKNQPHPSDPAKRQAPARATEPAPASPPAAPAALRGQGLAGLGSLRAALTGAAERRAREAAQAQQSARQ

AAADADLFRREIGAIKPLAAPPRAASGRLAPAPVPKHTRQDEEAVLSETLSDEFDPETLLDTDETLYYHR

PGVSRDVVRKLRSGAWIVQAQLDLHGMRRDEAREALAEFIRESGKKGLRCLRVIHGKGLGSIGKEPVLKG

KVRAWLVQKDEVIAFCQARGHDGGAGAVLVLLQPS

>WP_004186021.1 MULTISPECIES: energy-coupling factor ABC transporter permease [Burkholderia]

MGFLYTPLPFWIAVGGWIAAVGLLVLALWKRPFGRLQDATLQHVWLALVTTITVLWASNAWLDDGLVMHL

LGATLLVTLFDWTLALVGMAAVIGVAAIVFDATWQGVGLTYLVYGALPVAVSTLLQRAALAWLPHNLPAL

IVGQGFVSPAIAVGIVAAAAAGVQIAIAEGTLVVVPSGYALNALLLALGEAWFTGMATALIAVYRPAWVT

TSDVRRYRLGGPRA

>WP_004185589.1 MULTISPECIES: O-antigen ligase family protein [pseudomallei group]

MTITTSRVERVLWIACPTLMFGVMFGHMNGVVNTTLALIGVGTLAGILSASRPPFRQWPLVLPIVVWAAW

SLASVGWSLYPRISLRAWFDEVLYPLVTFWGFWLFGSRVKRPEWPVLVVWAACVLLALLSAFYWGHLQPP

TPNTFPIRFYNRVGHTSTLVVFAMPLFATLLLRARWRAIGAVGLVACGFVGLASLNRFFWPAAGATLVVA

FYPLYRRRLGVSIVAIAILGATALGLLEYSARERDLSASTATASASSAARDITVAGQRVYVPGELNALGD

TLSSDTRPKLWAFYTEQGKQHAWLGVGFGKPLPGHAYAADAPPLLLQIEPQALTHAHNLFLNTWLQTGYI

GVALEALLLVSLAAAFWRLRRDVPAVSAAGLALVVGMIAKNTVDDFMWQTTMLAFWSFAGFLLGCGERDA

CMARVSQNADAR

>WP_004185551.1 MULTISPECIES: acyl-CoA dehydrogenase family protein [pseudomallei group]

MDLDYSPADDAFRAEIRAWLEANLPRALRDKVLNHKRLNRDDFAGWHRLLGTRGWSAPAWPAEYGGPGWN

ATQRHIWEEECARIGAPPVLPFGVSMVAPVLMKYGSEAQKRRYLPRILDGTDWWCQGYSEPGSGSDLASL

RTRAERRGDHYLVNGQKTWTTLGQHADMMFCLVRTDPDAKKQEGISFLLIDMKSPGITVRPIVTLDEDRE

VNEVFFEDVKVPVENLVGDENRGWTYAKYLLGHERTGIARVGASKRELDFLKRIASRERKNGKPLIADPV

FAAKIAALEIELMALEVTVLRVVSSEASGRGPGPEASMLKIKGTEIQQALTELMVDAIGPLAAPFDAAFL

EGERERSIAGDDDAAPLAAYYFNYRKTSIYGGSNEIQKNIIAQMILGL

>WP_004185460.1 MULTISPECIES: phage holin family protein [Burkholderia]

MTTETSSHQSGHGPLRRLLGSVLALLQTRLELVGIELAEEKERLMGVLFVGLAAMMLATMALISLTVLIA

IAFWDTYRWQSLAVVTALYALGALACWLKARSGLRDAPSVFEATLNELEKDRELFRGKP

>WP_004185251.1 MULTISPECIES: endonuclease/exonuclease/phosphatase family protein [Burkholderia]

MHVPQTAPLAEPLPVADEITAVSWNLHKGRSPLGFTAWEAMRSWVASTHADVYFLQEAMARRMPRPVLAA

GFGAPMAEPVDDIWHCQATEIARALDWQIALGPNVFKPSWRHGNAILSPHPLDLGGRWDISAHRFERRGL

LVARATLAGGAPVTLLCAHLALTRAARLRQMHWIAHWIERNARTGPLVLAGDFNDWRNDSIPLFGEIGLS

EVATLLGESGRTFPAFSPALALDKMFVRGMTPLEWRAPGDETAWLSDHLPYVARLRLDPA

>WP_004185176.1 MULTISPECIES: glutathione S-transferase N-terminal domain-containing protein [Burkholderia]

MMVLYSGTTCPFSQRCRLVLFEKGMDFEIRDVDLFNKPEDIAVMNPYGQVPILVERDLILYESNIINEYI

DERFPHPQLMPADPVQRARARLFLLNFEKELFVHVSTLENEKGKAAEKSHEKARLAIRDRLTQLAPIFLK

NKYMLGEEFSMLDVAIAPLLWRLDHYGIELSKNAAPLMKYAERIFSRPAYIEALTPSEKVMRR

>WP_004184923.1 MULTISPECIES: autoinducer-binding transcriptional regulator BpsR [Burkholderia]

MELRWQDAYLQFSAAENEQQLFQQIAAYTKRLGFEYCCYGIRVPLPISKPVVAIFDTYPNGWMERYQEMN

YLEVDPTVREGALSSNMIVWPEASASDATTLWSDARDHGLAVGVAQSSWASRGVFGLLTIARHTDRLTSA

EINHLTLQANWLANMSHSLMSRFLVPKLAPESGVALTHREREVLCWTGEGKTACEIGQILSISERTVNFH

VNNILDKLGATNKVQAVVKAIAMGLIDAP

>WP_122826984.1 catalase/peroxidase HPI [Burkholderia pseudomallei]

MSNEAKCPFHQAAGNGTSNRDWWPNQLDLSILHRHSSLSDPMGKDFNYAQAFEKLDLAAVKRDLHALMTT

SQDWWPADFGHYGGLFIRMAWHSAGTYRTADGRGGAGEGQQRFAPLNSWPDNANLDKARRLLWPIKQKYG

RAISWADLLILTGNVALESMGFKTFGFAGGRADTWEPEDVYWGSEKIWLELSGGPNSRYSGDRQLENPLA

AVQMGLIYVNPEGPDGNPDPVAAARDIRDTFARMAMNDEETVALIAGGHTFGKTHGAGPASNVGAEPEAA

GIEAQGLGWKSAYRTGKGSDAITSGLEVTWTTTPTQWSHNFFENLFGYEWELTKSPAGAHQWVAKGADAV

IPDAFDPSKKHRPTMLTTDLSLRFDPAYEKISRRFHENPEQFADAFARAWFKLTHRDMGPRARYLGPEVP

AEVLLWQDPIPAVDHPLIDAADAAELKAKVLASGLTVSQLVSTAWAAASTFRGSDKRGGANGARIRLAPQ

KDWEANQPEQLAAVLETLEAIRTAFNGAQRGGKQVSLADLIVLAGCAGVEQAAKNAGHAVTVPFAPGRAD

ASQEQTDVESMAVLEPVADGFRNYLKGKYRVPAEVLLVDKAQLLTLSAPEMTVLLGGLRVLGANVGQSRH

GVFTAREQALTNDFFVNLLDMGTEWKPTAADADVFEGRDRATGELKWTGTRVDLVFGSHSQLRALAEVYG

SADAQEKFVRDFVAVWNKVMNLDRFDLA

>WP_006026960.1 MULTISPECIES: RNA chaperone Hfq [Burkholderia]

MEYAMSNKGQLLQDPFLNALRKEHVPVSIYLVNGIKLQGNIESFDQYVVLLRNTVTQMVYKHAISTVVPA

RPVNFHPDAEAAS

>WP_004193640.1 MULTISPECIES: RNA polymerase sigma factor RpoS [Burkholderia]

MPKSKRHDPQAESEKISRAKQASVERTGASADEDEDAADNERDYESRDADPDESGEGRGDAQPDLDDFRA

LLQAELTADTIQHYLNRISVKPLLTVEEEQRYSRLAKAGEFEARQVMIERNLRLVVSIAKGYLNRGVPLL

DLIEEGNLGLMHAIEKFDPTRGFRFSTYATWWIRQSIERAIMNQARTVRLPVHVIRELNQVLRAKRHLEK

NSMSTGEAAERREASIDDIAYLTGKTAEEVTDILALNEHTASLDAPLDLDPASSLLDLLPDDQSQSPDAE

VQHRELETLTRAWLSRLSDKHRHVIERRFGLNHIEPATLEELADEMGLTRERVRQIQQEALVRLKRFFAS

NGVRKDAVL

>WP_024428514.1 tetratricopeptide repeat protein [Burkholderia pseudomallei]

MKKLLAAVGLSLILLSAAANAAVPSLQQIQQSIAQGNWQRADAQLSQVIDAYPDNARARYLYGQVLDREG

RPAEALAQIERAKSLDPQLRFTDPSRFAQTEARVRADARRATAAQDSRSATSGGMLAAPQAPAQARAPFS

AAPVAPAAPVHRGPSVGMWIGFAVLIGVIVIVLRKTLRRARSTDDQRADDERRAQLKRATDILNEVRPLK

LDARLSTAPGAAALNGEIEGLEAQARELVETLSNGKNPAPPYRLDELEKQFASLKARVEGRPDPNAAAPG

GPGQTGSVFAQEADRLTGAQGQPPYSPYPPQPQQPPPVVIQQGGGGFGGGMGGLLTGVLLGQAMSHSRDR

VIERDVIVDDEARRRAGADPGIDFGQGDSWDSGGSDGGGSIDLGSSGDDWSNNG

>WP_004546282.1 GNAT family N-acetyltransferase [Burkholderia pseudomallei]

MSHAIGVRRIGPDEAAACVDALSDVLIDCVEGGASVNFMAPLARDKARAFWREAAEGVARGERTLFVAED

ADGRIVGTVQMITRQPENQPHRADVAKMLVHRDARRRGVAQRLLAALDDAARAAGKTVLVLDTVTGGDAE

RLYARAGWQRVGVVPDYALMPDGAPCATTFFYKQI

>WP_004538671.1 MULTISPECIES: helix-turn-helix transcriptional regulator [Burkholderia]

MDINQRIARRVRALRDLRGYSLDALAERSKVSRSNISLIERAQSNPTAVVLERLANALGVSLASLFEDDR

AARAASPLSRATEQPVWKDPASGYVRRSLSPAVASPLQLVEVKFPPGGRVAYDGGERNADVHQQIWLLEG

EMDIASGDDTWRIAAGDCLAMRLDRPAVFFNPGRKAARYVVALAAANAVRPGWIE

>WP_004538054.1 pirin family protein [Burkholderia pseudomallei]

MIEVRAANQRGRAEHGWLSSRHTFSFAEYYDPAQVGFSDLLVINDDRVAPGRGFGMHPHRDMEILSYVLE

GALEHRDTMGTGSVIVPGDVQLMSAGKGIAHSEFNHSADRPVHFLQIWIGPSVRGVEPRYQQASVSDDDK

RGRLRLIVSPQGQDGSLKIRQDARVYAGLFDGDASERFALPPQRFAYLHVARGSVTVNGVELHEGDGARI

RDEQALQIANGKQAEVLLFDLRPIEVTAEWA

>WP_004537316.1 MULTISPECIES: DUF3326 domain-containing protein [Burkholderia]

MHIHEKAFSVSGPLASASLDHLRELTRQQLGPDETPVRFVVTRSDEHGFDCEIGVLSDGEMPESMRADGL

FRCERRTRGGGGEFNAVLIVPTGVGAEIGGHDGDAGPVAMLLSSICDRLITHPNVVNAADINELPANGLY

VEGSVICRLLLGQIGLQPVRANRVLAVVGSNECPTFVNAAINAVNAARAAYGLDCPRVLHLDPGIRLTST

YASSGRAAGTVENLDALVALLAEHRDTFDAVAIASHIHVPDACRTGYYAGTLGLVNPWGGVEAMLTHTLS

TLFSVPSAHSPMYEDPRFAVRDYGIVDPRIAPEVVSLTFLQCILKGLRRAPRIVELARAGNAAGTLSVSD

VSCIVIPQGCVGLPVLAALAQRIPVIAVRENRNVMRNDLRSLPWAPGQLRIVDNYWEAAGVLASMRAGLN

PDSVRRPLASVDVVRASAAHAHGASSIATAANIA

>WP_004532671.1 MULTISPECIES: helix-turn-helix transcriptional regulator [Burkholderia]

MASSSGARRTTAPASPATGATPPRVGEQIQRLRNERKLTLDDLSRAAGVSKSMLSEIERDKANPTIAVAW

RLTNALGISLDELFAQPKAPETIRVDGPHDIPTLAGHDAQYQLRVWGPIELAGKFEWYELTLPANGALVS

NPHEPGTREHLTVLNGAVEIEAAGTARRLKAGDTARYAADGPHAIRNAGKSEAKALLIVIHR

>WP_004531622.1 5-carboxymethyl-2-hydroxymuconate Delta-isomerase [Burkholderia pseudomallei]

MPHIIVEYTANIRDDARIPVLLKSINETLIAQGGVFPTGGIRSRAIELQDYCVADGTAADAFVHVTLKIG

AGRDDATKKAACDALFDAIKAHFEALYARRYLALSMELAEFSETGSYKHNNIHARYKRGA

>WP_004529642.1 type VI secretion system tube protein Hcp [Burkholderia pseudomallei]

MSHDIFLKINGIDGEAEDATHKGEIEVLSWSWNVSQQSNMHLGSGGGAGKATIDDLQFEHYIDRASPNLV

QYCLLGKHIDEARLVVRKAGGSPLEYIKLTMSDVLVTQVSPAGVAQDESRPRELVRLSFSRLKQEYVVQN

PQGGSGGAITATFDIKKNAA

>WP_004528095.1 response regulator [Burkholderia pseudomallei]

MALPVLVVDDSTLARKLLIKSLPSDWDVEITQASNGAEALLHYRAGKGAVIFLDLTMPVMDGFQVLEHIR

EEGLDAFVIVVSADIQEGAVDRVKASGAIGFVAKPVSTERIVPILKGYGLYE

>WP_004524001.1 MULTISPECIES: cupin domain-containing protein [Burkholderia]

MADADIERKSWDQPAGESFAQWMDGRVARFETRRYDWDALKFQADFDPKYRRAQMRYVGTGGTGVAKDAN

TVPAGGFTFSTMVIPAGNVGPSHIHIDVEEIFFVLRGRMKVICERDGETWEAVLGERDLISVPPGVYRTE

INVGEEDALMCVMLGSPKPITPTYPPDSPLAKIKR

>WP_004523721.1 MULTISPECIES: YjbQ family protein [Burkholderia]

MQQSIQHITVEARGRGLVEFTPQVRAFVEVQSVSTGLLTVFCRHTSASLLIQENADPSVQRDIERYFAAL

APEDDARYEHDTEGADDMPAHLRTALTQVQLSIPVEHGRMVLGTWQGIYLFEHRRAPHRRDVVLHLIGE

>WP_004523327.1 MULTISPECIES: response regulator [Burkholderia]

MTYERTESGEDVVLWRSLDGERAGHRRVLVVDDYRDAAEALRVLLDARGFECRVVDDPFEVCDTARDWQP

FAVVLDIAMPGLDGLEIARRLRRERATAHMLLVACSAFSARHDLERAHDAGFDAHCAKPLTPHRLLRFLE

AASAGTHDD

>WP_004521613.1 MULTISPECIES: LacI family DNA-binding transcriptional regulator [Burkholderia]

MATIKDVAAIAGVSFTTVSHVVNNSRPVSADVRAKVERAIRELNYVPSAVARSLKARSTATIGLVVPNST

NPYFAELARGIEDQCALSGYCVFLCNSDDDPEKQRNYLRVLQEKRIDGLIIASAGDDAVLAQTLADTREP

LVVVDRNIEGLPADLVQIDHERGAYLATRHLLELGHAKIGCITGAVSTAVSAMRVHGFIRAMAERGIDIV

PGAIAESDFSCMGGYHAAVRLFDSVRPSAIFAGNDLMGLGALRAAAERGIRVPADCSIIGFDDIELSRYT

YPALSTVGQSVRALGEMAAQTLIERIGGGASGAPQRRRVVSPRLVLRESTTVYVEPADAGYRG

>WP_004521166.1 MULTISPECIES: GNAT family N-acetyltransferase [Burkholderia]

MSDSAAAPLVYLLRPAASGDFEFAEALTHGNMNAYYQRHGLRWRADLFFASWRDSENFILELDSVSIGLL

RITEEGDSLHIRDVQIAPGHRQRGAGTYLLETSHRFAKARGLRETQLRVFVDNPAARLYLRMGYRLAGPR

LAQFGSIRHMARPVS

>WP_004198195.1 MULTISPECIES: response regulator [Burkholderia]

MIRTILAIDDSATMRTLLSATLGEAGYDVTVASDGEVGLDVALATRFDLVLTDHHMPRKNGLELIVALRR

QLGYEATPILVLTTENGDAFKDAARAAGATGWIEKPIDPDALIELVAALSASSSLS

>WP_004192434.1 MULTISPECIES: sulfate ABC transporter ATP-binding protein [pseudomallei group]

MGITVRNLHKRFGEFAALDDVSLDFPAGELVALLGPSGCGKTTLLRVIAGLEHADSGQVVLQGLDVASVG

ARERQVGFVFQHYALFRHMTVFENVAFGLRVKPRRERPSEAAIRAKVHELLSLVQLDWLAQRYPSELSGG

QRQRIALARALAVEPKVLLLDEPFGALDAKVRKELRGWLRRLHDDLHISTIFVTHDQEEALEVADRIVVL

NHGRVEQVGSPQAVYDHPRSAFVYEFLGAANRLDGTVSGNGFVAHGAAQAIAVDADFAGPARAYVRPHDL

ELAAPHARAQGIAADVRRVVPLGGSVRVELAARSGEVLEAELDRNAWRALALDVGDALTAVPRAVRVFPA

R

>WP_004192042.1 MULTISPECIES: helix-turn-helix transcriptional regulator [Burkholderia]

MSPSVPLFAQPPAATIDVPSEFAPTRSHPMRVRARAIAAGMRVPPHAHAWAQLAYASRGVLRLATAGSTW

MVPPSRAIWVPPRIAHEVVIVEDAYLRTLYVDESAVPGGLDACRVVEVSGLLRELIVALEARSLNRTRER

LLAELVLDELTRAEPLPLAVPMPTEKRLRALCEAVLAHPAQGESLEHWAAGVGASTRTIARLFKQELGVS

FSQWRQQALLARAIPLLNQGRPLSHIANELGYQSQSAFSAMFRRAFGASPRAFIQRGDMHAASELASTDD

GDAEPLL

>WP_004190980.1 MULTISPECIES: response regulator [Burkholderia]

MPLPIVIADDSLLARKLLTKALPADWDVDVNYASNGREALALYRDGKASVMFLDLTMPDMSGYEVLETLR

GEDLNTFVIVVSADIQPMAQARVRELGAAAFVAKPVTSEALLPILKEYGLYV

>WP_004189647.1 MULTISPECIES: response regulator [Burkholderia]

MATILVVDDEMGIRELLSEILSDEGHVVDVAENAQAARDYRLRQAPDLVLLDIWMPDTDGVTLLKEWASQ

GQLTMPVIMMSGHATIDTAVEATKIGALDFLEKPITLQKLLKSVEHGLARGAAPLPASAAAKPAAGAAVA

SAAALPTLGDDPAVALAGQTTAAIPFDIPLREARDAFERAYFEYHLARENGSMTRVAEKTGLERTHLYRK

LKQLGVELGKKPAEGAA

>WP_004188466.1 MULTISPECIES: response regulator [Burkholderia]

MIADERGNFVEILLVEDSPTDVMMTKEALEYYKVLNPLNVVEDGVAAMAYLRREGPYAGARRPGLIILDL

NLPRKSGREVLQELKLDPELSTIPVVILTTSKSEEDVARSYGLHANCYITKPVDFAKFTDVVHSINEFWL

SVVTLPPAKS

>VUD44784.1 unnamed protein product [Burkholderia pseudomallei]

MARIARLRMPGRARRPRLAAAVAAIVLGGFRPMRSRAAQPGKEGVMKWMLIVALCASTAGCGLAAAPCRV

ASAGLKIVPLVGHVAAAPTDACAGVIDPD

>VUD68404.1 AMP-binding domain protein [Burkholderia pseudomallei]

MREAVPNRRKPARVGAVRTRRSRKATTHRTETAAAPRAATRKETTLTSIAPENLPLERLQHWERARADDV

WLVQPGADGGVRRFTWRDAVDEARRVAAYLRAFDLPRGSNIAILSKNCAHWVLADFAIWLSGHVSVPLYP

TLGAESIRQVLTHCEAAALFVGKLDAWETMRAGVPPHVRSIGLPYLHDASSVGATWDDIVRDTAPLAEHV

TRAADALATIVYTSGTTGEPKGVMLTFGALGWCVEPVFDLVAIGPDDRMISYLPLSHVAERGYVEMLSVR

AGFTVYFSDSPDTFIADLQRARPTFFISVPRLWAKFRHAVASRLPPGPVPDAMKPMILRQLGLDQVRLAA

SAAAAIEPALLRWYRDLGLELLEGYGMSEVCGVSHSCRQHDMRLGYVGTPVRDVESRLADTGEIEIRSPG

NTIGYYKRPDLSAALFTPDGFVRSGDKGELDEAGRLKITGRVKEIFKTSKGKYVAPSPIESRLATHPFVD

ACCVVGSGLAQPCALVSLSDEGRRFDGDGRRAALETSLGEHLAHVNASLDDHEKLRFVVVVDSVWNETSG

FVTPTFKVRRNRVEARYAPFLDAWDARGATIVWESDTA

>VUD67135.1 Probable D-lactate dehydrogenase, mitochondrial [Burkholderia pseudomallei]

MTAGANAARTAGDTRAGARGGAGMGRPRVCAGCAPRRAGAIDERRRRMREVTDDGQQWDGRAAAFAGVPP

GGGAPAAFATPQTIGRCVEALRAIVGERLAVGRAVREQHGKDISFHPGAPPDAVAFVRTTAEVAEITRAC

HRLRVPLIPFGTGTSCEGHIAALRGGVCIDLSGMNRILRVSAEDLDCTVEAGVTRKQLNAHLRDTGLFFP

IDPGADASIGGMCSTRASGTNAVRYGTMRESVLALEVVLPNGDVVSVGSRARKSAAGYDLARLFVGAEGT

LGTITGVTLRLHPRPDRLSAAVCSFPDLKAAVDSVIGVIRSGVPIARVELLDALQIVACNRYAKLSLDER

PTLFFEFAGFGAAVDEQIDWVGEICAGYGGGEFRWASRPEERSALWAARHDAWWAALALRPGCEGMPTDS

CVPVSRLAEAVLAARADVDALGLVATICGHVGDGNFHVCIAIDPADADELARATELNRRLALRAIALGGT

CTGEHGVGYGKIGYLEREHPTALQLMAAVKRALDPRGIMNPGKVLHHDRYPPFD

>VUD53320.1 hypothetical protein UKMH10_2794 [Burkholderia pseudomallei]

MKASPPIRAGMDFIVERAQSAPALQRAGRRRVDRAPGAPVDARRPLGRASLVARRPPPVARDSSFVVSRS

KRFRPCAARAYDRSHFERNPERKSRAMSDSRRPPSNPSPAMRDGAPPVDAERIDAAVRALSRADALLVTA

GAGIGIDSGLPDFRGAEGLWRAYPGLGHVGYAFHEIASPRAFRERPRLAWGFYGHRLAMYRATVPHEGFG

ILRRWIGAMRHGGFVFTSNVDGQFQKAGFDPERIVEVHGSIHAMQCMSSCSAYTWDAESFAPVVDEPACS

LVGALPRCPRCGGLARPNILMFDDADWLGARYDAQHEALRAWLARAGRVAVVEVGAGTAVPTVRAFSERT

GDDVIRINVREAHARRDDVIGLPGGALETLRALDAAWRRD

>VUD49633.1 hypothetical protein UKMH10_1987 [Burkholderia pseudomallei]

MTMLHIELKERIETEFETARVESVTLRRDALDVRLANGVELTLRIANAREYAMDWRWGEAAMRIDTAPRH

APPEAAHPNHLHAPDGRVLADPVTEFGGEPWRNVGALIERLAAAPLLGHEPPSAHGLPSDARAIAS

>WP_024428951.1 MULTISPECIES: COX15/CtaA family protein [Burkholderia]

MYLLQLGLIGLCIALLPLSYVWVKADDDKFRKLVWITTFLTLDLVMFGGFTRLTDSGLGCPDWPGCYGTS

SPFIAHAAITAAHQAMPTGPVSMTKAWIEMIHRYFAMAIGVLIIAQTVIAWAARLRRKPLHVSPWWPTSL

LLLILVQGAFGAWTVTMKLQPVIVTIHLLLGLTLLGTLGWLAARQTPLPAHEPGASRYRAAALAALVLLV

VQIALGGWVSTNYAVLACTDFPTCNGAWIPPMDFRNGFHLWRALGMTNDGDAITQDALVAIHWTHRTFAF

VVVAYLAAFALKMRRFASLRRPANGVLAVVVLQFVTGLTNIVLQWPLPVAVAHNGGAAILLLLVVMLNFR

ILSSRPGRVAQPARDAAPA

>WP_024428674.1 hybrid sensor histidine kinase/response regulator [Burkholderia pseudomallei]

MSTDDDFGRASLLELFREETHTQTQALSERLLALERGAQDAATLEACMRAAHSLKGAARIVGVPQGVDIA

GRMEDCFVAAQHGRQPLTPCHVDALLTGVDLLVRVGDPQTAASVAPHEIDAFAAALAAADAGVDPGADAR

GANAYESDGNAHERGGAARASVAAAPGEAADASRESGAREAAGPNRAGAAASAADVRASADAGDARGASD

TRDVRHTAGARGANRSTEVFGAGEATGIGASTHANDANGMNATNLATSAAHPPRGAFAAAPDAPPSSAPS

DAPRAPSQMRRVRADTLNRLLSLSGESLVESRWLKPFAESMLRVKRAQRDAARSLDSAVEALADDADPRV

RGALNEARQMFTDLQRTFAARLDELDRFERRSSHIAEQLYDEALQCRMRPFGDATRAYPRVVRDLARSLG

KRVRFSIIGEATQVDRDILDMLDAPLGHLLRNALDHGVEPPEVRLARGKPAEATITLEARHSAGSLLVSV

SDDGPGADLAAVRAAIVRHHLTDEDTAARLSDQELLEFLLLPGFSMRERVTDVSGRGVGLDAVQEMVKSV

RGAVRIFNEPGLGMRFVLQLPLTLSVIRSLIVDVGGEPYAFPLVQVRRTLELERADIDVLEGQQHFPLDD

RRVGLVTAHQLLDAGELDESRPATAVVVVGGEPETYGVAVDRFLGERMLVVQPLDGRLNKIQNIAAGALL

ENGDPVLIVDVEDLIRSIDKLIRGGQLAKVRRGDRDALARRAKRVLVVDDSLTVRELERKLLEKRGYDVT

IAIDGMDGWNAIRGDAFDLVVTDIDMPRMDGIELVTLIKGDPLLKSVPVMIVSYKDRDEDRRRGLEAGAD

YYLAKGGFHDEALLDAVHDLIGDA

>WP_024428614.1 nitrous oxide reductase family maturation protein NosD [Burkholderia pseudomallei]

MPIPHALATLAAAALLMHAACAATLAVHPGERIGAALAAARPGDTVLVQHGRYEENLRIDKPLTLRGVGR

PTIDGRLAGDVIRVAAPDVTISGFSIVDSGASLTAQNAGVYVAPGSDRTRIERCELVYNLFGLWIERSAD

VRVTGNVIVGKRDLLSPRRGNGIQLYNTTGARIAGNTISYTRDGIYVDVSHHARFEHNTIHDVRYGTHYM

NSYYNVWDGNDVYHNRGGLAIMEARDQIVRGNRVWGNTDHGIMLRTIQDSLIENNVVAGNQRGLFIYDAE

YNTIRGNLVVDNKIGVHLWGGSIHNDVTGNDFADNREQIRYVAASDVAWPGNYWSNYLGWDRRGRGIGDV

PYKANDLVDRLTWRVPSVKVLMNSPAVQALRVVARQFPLLAVPSVVDDAPRMRAAHAGWPQWVGKR

>WP_024428519.1 TonB-dependent receptor [Burkholderia pseudomallei]

MDSICNRRPRRAHAGVCADTANAPAGLPEAAQPAARLGAGRPRDAAAPRCARQATSHTSHASHTLHAPCM

PQFAPRRVVARVAAAALFSACAAPGVSGAAEPAAATRGYDIPAGPLDAALTRFGREAGILLSFPGELTTG

LRSPGLHGRADPAAALDRLLTGTGLVALRQPSGGYTLARLPGPAAAGADAALAADTTLPTVAVRASGPHA

DSYRPPREAAGLRSDAPLAEVPQAVAIVAQQVLRDQRPRNLDDALANVSGITQGNTLGSTQDTVMKRGFG

DNRDGSVMRNGMPIVQGRSLNATTDSVEVLKGPASLLYGIMDPGGVINVVTKQPQLARRHAISALGSTYG

GGRNGGELTFDSTGAIGESRVAYRLIVDQTNEQYWRNYGEHRETLVAPSLAWYGRDTQVVLSYEYRRFLM

PFDRGTALDPRTNAPLAIPARRRLDEPFNDMRGESNLAQLAIDHQLAPDWKVHVGYSYNRETYDANQIRI

TAVDPLKGTLTRSNDATHGSRSTDSYGIAYVDGRVTLAGMRHDVQFGVDGEYRQVYRADMLRQPIKTPFS

YLNPTYGLVPPSTSVSASDSDQSDTLHTASLFFQDSIHLSERWMLVGGARWVRYSQLAGRGRPFQVNTNL

SGTKWLPRAGIVYKWNDALSLYGSYTQSLKPTSKIAPMAGGYVIDGSTAPEEGASWELGAKLDMPGGLAG

TLAFFDIDKKHVLVSQYDDATNQTAWRTSGRARSRGIELDVSGRIGARWNVIASYAYIDAKTTEDPLYAG

NRLWNVARHTASLAAVYDVGTVLGGDDLRIGAAGRYVGARPGDSANSFTLPAYATADAFATYDTRLGKQK

LQFQLNVKNLFNRTYYPSSVNRFFVSVGDARQVSLLTTLQF

>WP_009950383.1 MULTISPECIES: polyhydroxyalkanoic acid synthase [Burkholderia]

MDTRHAPESGAPDAPLPAHPPASYAPESPYRIFDLAKEASVAKLTSGLSPASLQLALADWLIHLAAAPGK

RAELATLALRHAALLGQYLLEAATGRTPAAPAQPSPGDRRFRAGAWQLEPYRFWHQSFLLAEQWWRAATR

DVPGVSPHHEDVVAFSARQMLDTFAPANYVATNPEIAQRTALTGGANLAQGVWNYLDDVRRLITKQPPAG

AEQFELGRNLATTPGRVVFRNHLIELLQYSPTTPDVYAQPVLIVPAWIMKYYILDLSAHNSLIRYLVGEG

HTVFCISWRNVDASDRDLSLDDYRKLGVMDALDTIGAIVPGEKIHATGYCLGGTLLSIAAAAMANTGDDR

LASITLLAAQTDFAEPGELQLFIDDSEIHFLESMMWERGYLGAHQMAGSFQLLMSNDLIWSRVIHDYLLG

ERTPMIDLMAWNADSTRMPYRMHSEYLRHLFLDNDLATNRYVIDGQTVSVHNIRAPFFVVGTEHDHIAPW

RSVYKIHYLSGSDVTFVLTAGGHNAGIVSEPGHAKRHYRMKMTAAAAPSISPDEWLAGATDFEGSWWPAW

HAWLARHSSPQRVAPPPLGKPGARTLGDAPGTYVFQK

>WP_004547716.1 cysteine dioxygenase family protein [Burkholderia pseudomallei]

MNHAASSLPHAPDPHPKPRERAPSAPCRDRATPASTPIARFCTRLDAAFDSLRDETAPSRHPAFARALRA

ALAEAAAAPGLLTPAQRESGAAGYRRHLLAADPAGRYAVVSLVWEPGQWSPVHGHHAWCGYAVLEGKLTE

IVYRWSDAACCAIDARRRARPMGAVSYVCAGRDAIHRLGHAGAKTDAPAISLHVYGVAGEQVATHVNDVV

AVDARAYA

>WP_004540506.1 cupin domain-containing protein [Burkholderia pseudomallei]

MFQAGIGVNFEAALRDVSEYWSPRVVGRVNDQYVKVAKLKGEFTWHKHADEDEMFYVVYGRLRIQFEGRD

DVVLNPGDFCIVPKNTMHNPVAEAECGIALIETVTTRHTGDTPSPLAKSIDAQLAG

>WP_004535221.1 VC_2705 family sodium/solute symporter [Burkholderia pseudomallei]

MLTNRLVRAYALYTIGFAAFVLLLWRIERATGPGVWIGYVFLFVPIAVYAVIGLLSRTSDLVEYYVAGRR

VPSAFNGMATAADWLSAASFIGLAGSLYATGYDALAYLMGWTGGFCLVAFLLAPYVRKLARYTIPDFLGT

RFSSTLVRALAAIAAILCSFVYLVAQIQGIGLIATRFIGVDFSIGIFCGLAGILVCSFLGGMRAVTWTQV

AQYIILIIAFLLPVSLIAMKNGLGPVPQFNYGRLMSRVETLEGEMRDAPQERQVRETYRRQAGAIQARLD

RLPASYDEARAKLVDQVAELRRHNGPLREINQRERALAEFPRDPAAARVVWEQARDELLARAAAPVPMHE

PFPAASGDDRRPRGRNFLALLLCLSLGTASLPHILTRYNTTTSVAAARRSVGWTLFFIALFYLTVPVLAV

LIKYEILTNLVGRPFADLPAWITQWHRFEPGLIGVTDLLRDGIVHWSEIQMQPDIVVLAAPEIAGLPYVV

SGLIAAGALAAALSTADGLLLTIANALSHDVYYHMVAPDASSQRRVTISKVLLLGVALFASYVASLNTGK

ILFLVGAAFSLAASSFFPVLVLGVFWKRTTTRGAVAGMMTGLGVCVYYIVSTYPFFTQITGFAGPGWLGI

EPISSGVFGVPAGFATAIVVSLLDRRPDAYTNALVDYIRHP

>WP_004533348.1 AraC family transcriptional regulator [Burkholderia pseudomallei]

MKPRYERVAIPDGCSVRVYRRRLAQIPFEWHHHPEYELTLTLNSLGKRFVGDHVADYAGDDLVLVPPNLP

HTWVSDERLDPGEPLVALVLWFDGDWAQRVADCCPEFAGLRTLLRRAAPGLAFAPAAAADMRARLPALVD

RAPRVRLAAALDVLACLADAPATPLATAAAYRAASGAALAPEAERLDRVLDLLDRRFHEPLRVAELAALA

HLSERSLQRRFARHVGESIGSYLQRLRLAHAARLLASTDWPVSLVATRSGYANLANFNRQFLAARRVTPR

AYRRFLAEHGRAPDDMPAHEASIDVRPPSLDHGPPRAGKNKIAR

>WP_004531083.1 sensor histidine kinase [Burkholderia pseudomallei]

MGELADESRIFLSTLQPERRERGLAMTAVALSVVLFATLAPFVRIQMPQVWAFIPIYQSAIVICDLITAG

LLLGQFGILRTPSLVVLAGGYLFTACMASVHMLTFPGLFLPSGLLDAGPQTTAWLYMFWHSGFPLCVIFY

TLASRRERAEPLYAGRLALPVIVCIGTALVATGAFAALATSGRELLPPIMANNHYTPLMRGVVTTVWLMT

LAALILLTSRRPHSVLDLWLIVVLCAWLLDIALSAVLNHGRFDLGFYAGRVYGLVASSFVLLALLLENGK

LYARTVHALQGERIEHRRVQEKTVQLNEANELLEQRVAERTRELQAANDELRREIVERERAEEALARSRE

ELREIAAISSTAREQERGRIARELHDELAQTLAMLRLDLERVSTTGNELAGSFMEMRGLLDGAVAATRRI

ASDLRPLMLDDLGLVPAIQWLVQAFQQRHGIDCTLIVDPPELDLVEPQASTTFRILQESLTNVGRHARAS

HVDIRLIADDREVMLSVRDNGVGFDTENPRKPNSFGLVGLRERAYLVQGTLSVQTAPGQGTAIEVHIPLV

RAREVTVLQSGELRE

>WP_004530710.1 AAA family ATPase [Burkholderia pseudomallei]

MINILVASEDASRLAHLARLVGDAGRYRVTRTVGRAAQIVQRTDGLDAFDILMIDGAALDTAELAAIEKL

SRLHPGLTCLLVTTDASSQTLLDAMRAGVRDVLRWPLEPRALDDALKRAAAQCAQRDTPDTRIVSFMSCK

GGAGTSFIAGNIAYEIAEGSKRRTLLIDLNQQFADAAFLVSDQTPPSSVAQLCGQLERLDGAFLDASVVR

VTDTFHVLAGAGDPIKAADIREDALEWILGVAAPRYDFVIFDLGVSLNAVSMVALDRSDHIEVVLQPSMP

HVRAARRLQELLVSLGCPPERIQLVLNRQTRASERARAALEEVLSTRAAHVIADDPATVNEAVDQGVPLS

RLSRNCGVARSLQAFARQLAEGEQRPRRDSARDSPLLARFFSRGAAPKLKSM

>WP_004530630.1 MULTISPECIES: NAD(P)H-dependent oxidoreductase [Burkholderia]

MTYRIAVIVGSLRRESFNRALANAVASLSPADLSFEPVDIGSLPLYSQDYDADFPDVAKHFKQTIEAAQG

LLFVTPEYNRSMPGVLKNALDWGSRPWGSNSWSGKPAAVLGTSPGATGTALAQQHLRNVLAYLDVPTLGQ

PEMFIKHDASKIDAQGNIVNEDTRKFLQGFVDRYADWVHRHAESA

>WP_004529801.1 MULTISPECIES: substrate-binding domain-containing protein [Burkholderia]

MQTRKTWFGRFAAAAALGATLAIAAAGSAAHAASGPYRIGAAVYGMKGQFMQNWVREIKAHPAVKSGAVQ

LTVFDGNYDALTQNNQIETMLTQQYSGILFVPIDTKAGIGVAARAGASDTPLVASNTMLATPKVPYIGND

DVEGGRLQAEALARRIGGKGNVVIIQGPIGQSAQIDREKGEMEVLAKYPGIKVIEKKTANWSRAEAMNLM

EDWLNAHPKQINGVIAQNDDMALGALQAIKNRGLTPKDIPITSIDGMPDAIQAAKRGEITTFLQDAQAQS

QGALDLVLRQLVGAGYKPQSVIWQRYAKDLKWDGGTAKRYILPWVPVTPANADQLYKQVTGG

>WP_004529123.1 hybrid sensor histidine kinase/response regulator [Burkholderia pseudomallei]

MMNKTNRDNASDSLESGISVLLVDDQAFVGEVIRRSLVPESDIVLHTCTDAQRALAMAKEVKPTVILQDL

VMPDIDGLELVRAWRADAQTARVPIIVLSAKEEPAVKRAAFVAGANDYLVKLPDTIELIARIRYHSNSYL

MLRQRDEVLDFLSHDMRTPQTSILALLNVYRNEHGAMPPILERIETHARRALALADGFIHLTRAQSEKRP

SETVSLNEVVLDAVDQMWEKAGSVGSRVRADVPPTECLCIGDRMMLTRAVANLVDNALKYGPGGSEVRCT

LVEDGDGWLIGVEDEGAGIAPDQRAAATESFVRLVPAGGAQRGGFGLGLAFVRATAAKHRGQILMRYTPR

GFLAGLRIPKDVSAA

>WP_004529020.1 methyltransferase domain-containing protein [Burkholderia pseudomallei]

MLEQTAEHWGQSYFSETFNRREWQAHPLSIQRQYELQGNLMREEWLFSRYLNRKRVQRAASLGAGRAETE

IALLELGAVEHFDLFDVSSVGIEYAKSIAEEKGFGHKVTCHVGPIGCAELNENTYDLITFVASLHHMEPL

AETLERANRALTQHGIIWCANEYIGPDRFDYPVAHAAIAKSFFQQIPPGLRNHWHRELQFPTPQEVAEVD

PTEAPCSSKIEPTMRDMFPALEIMPLYGAFAFMVFWGLNHDALYETPEGTELTRFILGMDKALTDAGILP

TYFAHIIARKNNAPRQQIHRPGRHSSALLYRYAQRAASILRHLKIR

>WP_004526471.1 carboxypeptidase family protein [Burkholderia pseudomallei]

MTLSITSNFDAGAIDVVSCERADAIRLRVRGDNRSEFAQWFYFRLTGARGERCVMTFENANDCAYPAGWR

DYRAVASYDRVNWFRVPTSYDGQMLTIDHTPEFDSIHYAYFEPYSEERHSEFLGAVQQMPQASVVELGRT

VEGRPMSLVVLGTPDEAGAAKKKVWIIARQHPGESMAEWFIEGLVKRLVGWGDWSGDPVARKLYDHATFY

VVPNMNPDGSVHGNLRTNAAGANLNREWMEPDAERSPEVLVVRDAIHAIGCDLFFDIHGDEDLPYVFAAG

SEMLPGFTEQQRVEQSAFIDSFKRASPDFQDEHGYPPGKYREDALKLASKYIGHRFGCLSLTLEMPFKDN

ANLPDEHVGWNGARSASLGAAMLGAILEHVRAFA

>WP_004526300.1 MULTISPECIES: CbiX/SirB N-terminal domain-containing protein [Burkholderia]

MNKHGIVLFGHGARDARWAGPFERLAAKLRAARGAEASVVLAFLELMEPSLAAATAALAAQGCDAITVIP

VFFGEGGHVRRDLPGLIDACRAAHPGVDIRCATAVGEDDAVLDAIVAYCMRASADPTS

>WP_004526276.1 aldo/keto reductase [Burkholderia pseudomallei]

MTSRLETVALPGGERVPKLGQGTWEMGERPAKRAAEIAALREGVDLGMTLIDTAEMYGDGVTETLVGDAL

ADVRERLFIVSKVLPHHASRGGVVAACEATLKRLRTDRVDLYLLHWRGSIPLAETIAGFEALRDAGKIRY

WGVSNFDVDDMEALVAEAGGAVCATDQILYNLARRGPEFDLLPWLARREMPAMAYSPIDHMRLPKRTALD

EIARERGVSPTRVALAWVLGQPNVLAIPKAGSVEHVRDNRAALDLVLGEEELARLDAQFKSPRGKRPLEM

L

>WP_004525793.1 molybdenum cofactor biosynthesis F family protein [Burkholderia pseudomallei]

MNDKPQDWKNYEDFAAGIDTNRLPATDALVGRALTFELPGGAFAANFVDGQTLSWRRGETGGTDWYEAIE

VAPDTFFVDVTFKSRPAEALTLVFNTATRRALGILSRIRSRDEAGAAPRVAQDFLVGTIAGGQADAGVSG

ILGIAGAEPAETRDLIGTRTLNVYSPNHTYEHTYLSSARYCWQCLVGEQRGHGDVDLATTYKFADDLYVF

TFREFLIPVASVFVFNFAAGRSTGKFLGETGDGAIANRPAGSFIRKLSQAVYPDDAQPI

>WP_004525403.1 aldo/keto reductase [Burkholderia pseudomallei]

MNYRRLGRSGLQVSELSIGSWVTYGNQVDRRAARESLAAARDAGVNFFDNAEVYAGGKSEEIMGHALKSL

GWPRISYVVSTKFFWGLAEAPNQYHTLNRKYLLNAIDASLQRLQLDYVDLVFCHRPDPNTPIEETVWAMS

DIIARGKALYWGTSEWSADEIRAAYEIADRHHLRKPTMEQPQYNLFHRKRVEQEYRRLYEDFGMGLTTWS

PLASGLLTGKYRHGVPAGSRAEIHGYDWLRAQLTDPAKNRVVEALGEIANDLGCTVGQLALAWVLKNPRV

STVITGASRVEQIIENMRSLDVTTQITPDMKQQIEQIVGDAYQ

>WP_004523532.1 MULTISPECIES: L,D-transpeptidase [Burkholderia]

MSNAPAPRTLTVPPLPPLTEAESADVTRRALALRAAFAQDVARRLNVPDAEQRAYGERLQQTLDANGLGE

LAREYVVLVDRAPNVQAVFLYFRTTRSNAWQMIGASPVATGLPGQYDHFVTPLGVFEHTPENMDFRAEGT

TNDNGIRGYGQRDMRIYDFGWTDAERGWGKGGVSQMRFQMHATDPEYLEPLLGIRHSKGCVRIPASLNVF

LDQHGILDAEYEARAAGGDPPWVLRAHRQVTPWAGRYLVVIDSQRKTRPAWSPAPGKKAQAKLPKGGDTA

D

>WP_004522783.1 MULTISPECIES: helix-turn-helix transcriptional regulator [Burkholderia]

MHSPLALVRDVSSQDAAAAADADASHAPFDALERLVGVNLARLRAERQLSLDALARLSGVSRAMLAQIES

ARSVPSIKVLCKVAAALKVSVAAFLRRHAVNGFEHLSAERAVRVVSSNGRFSARALYPEGEPAVAEFHEL

RIAPLHTEHGAPRAPGTSVNLVVSEGTLEVSIHERRQLLATGDAIVFDADQPYTLRNPGDTEARAFRVTV

NAEVPPRWHVPA

>WP_004521738.1 spore coat U domain-containing protein [Burkholderia pseudomallei]

MTPFRLFFVRIAATLALGLFSALHAHATCSVVSAAAASFGTVTSFAVARQPQSTSTTSSGLSCSGALLGL

FVIGDQINASITSANGGKLVGPTGDAVPYTVFADQNYSIKLDLGVTYNWASGQLLNLLGIFGGPAQTLPM

YFRTVQGSNVAAGTYTDTLTIAWNWDYCSGIGVLGICLGRDRGSGTAVVPVTITVTNDCMIAAPDVNFGA

APTVASFAPVTGSVSLTCTKGMVYTVGLSSGANPHASGRRQMANGANRLQYDIFGPGAAAVWGQSANRAG

SGAAADGRSAQQFPYTARLYPDQPTPAVGTYTDSVIVDVRY

>WP_004266710.1 MULTISPECIES: iron transporter [Burkholderia]

MLRSSFVRSGVALAAACAALSATAAEYPIGKHQIQGGMEIGAVYLQPITMDPEGMMRKASDSDIHLEADI

HAVKNNPTGFAEGDWMPYLQVTYKLTKQGDAKWAAQGDLMGMVASDGPHYGDNVKLNGPGKYHLTLVVKP

PMQTGHMAFGRHVDKETGVGAWFKPITLEYDFPFAGIGKKGGY

>WP_004202953.1 MULTISPECIES: ABC transporter substrate-binding protein [Burkholderia]

MKRFKIVAAHSIAAGVAAFAMLGAGAAHAQTVKVLSIVDHPALDAIRDGVRAQLKAEGYGDDKLKWEYQS

AQGNTGTAAQIARKFIGDRPDVIVAIATPAAQAVVASTKTVPVVYSGVTDPVAAQLVKGWGPTGTNVTGV

SDQLPLDRQVALIKRVVPKAKTVGMVYNPGEANSVVVVKALKEILAKQGMTLKEAAAPRTVDIAPAAKSL

IGKVDVIYTNTDNNVVSAYESLVKVANEAKIPLVAGDTDSVKRGGIAALGINYGDLGRQTGKVVARILKG

EKPGAIASETSSNLELFVNTDAAAKQGVTLAPDLVKEAKTVIK

>WP_004202229.1 MULTISPECIES: pentapeptide repeat-containing protein [pseudomallei group]

MSKIRSAVPPPPLPEVVEGQRYATPQRGVTLADTMFVDCHFERVEWTGCRLANLRFVNCTFDANRFDRCE

LEKLSYDSSRIRAGAWTQSALQRVSFNECELDGGTWTGSLVKDVVCTQSKGGAWTFDAVRGAHVSLVAGD

YAGVTLRGGRWSDTSWIGSRLADLRLESVELENLIAGQSGFERVVLVECRGVNVRWIDSRIERMTVHGCE

LKQAAWSHSTWATGEIHASRLPIASFDHASVNGLTVTNSELPQAIFDSASVADSALQGVRAPRIALRDAW

LTRVNLSGAQMQQLDARGVHLERVDLRGADCRGGNLIGQLSHTWAAADTRDAIFEEATSADDRLWWQRVQ

PGARGV

>WP_004200939.1 MULTISPECIES: universal stress protein [Burkholderia]

MYSIILVALDGSQTASHALDAALELAADAHARLVPVYVVDMPVFAFDTPGYDPSILVDAFREEGRRVLDD

AQARMTRRGVAGAPRLVEVEPPGEDVAERLERAAREIGASLIVMGTHGRRGVRRLMLGSVAERLLRHARC

PVLMIPARGAPAADANATHPTETA

>WP_004198453.1 MULTISPECIES: ATP-dependent helicase [pseudomallei group]

MARIIPDDWKNLAATGAAERERETLATLERTLPDAYTVYHGVHWTRADHNFSVFGEAHFVIVSPAGRVLV

IEQKAGFLRETPKGLVKVYLQTERNVSIQLARTLENLHRRLTAALGAGAYGVEELLYCPDYTIRNPAIAG

VAPARIVDATRRHALAARIVDALPADEPPFPSAAKVHHFLADELSLTPDTSALVGQADTLVTRLSGGLAA

WARRLEFEPFRLRVIATAGAGKTQLAVQAMRGAISAGKRVLYVCFNRPLADHVARIAPQGVKIANYHQLC

DWIARDGGHVPDFGARDAFAQLEARFAQTPIAERWRFDTLIVDEGQDFQPSWAHALERLVAPGGAWWWLE

DPLQNLYMRERVPLPGWVTLKETTNYRSPRDILDYVRDVVGSVEPLAAELVSGSPFGGSEISISAYDDAH

AQSPAQACIDATKRAITHALALGFRKQDIAVLSFRGREGSALATLDQLGPHRVKSFTGKYDLFGNPEYRE

GDVLLDSLHRFKGQAAPCVILTEVDFDAFDERAARKLFVGATRATMKLIVVASKRAAQRIAPSA

>WP_004195547.1 MULTISPECIES: 2,4'-dihydroxyacetophenone dioxygenase family protein [Burkholderia]

MNSPLPPAPAAFLPPISCLQDDDRPWLPMDASLPGLAIKYLHINVADDEMTVLLKMPVGLALPRHRHDGA

VFVYTLQGEWRYREYDWIARPGSAVLEPAGSHHTPEALASETGAVVTFNVMRGDLVLLDDAGRETARENC

RVALLRERRFARGAPDAAAPFVTR

>WP_004193771.1 MULTISPECIES: inorganic phosphate transporter [Burkholderia]

MHSIQLALWVVAALVLVALVFDFMNGFHDAANSIATVVSTGVLKPQQAVVFAAAFNVIAYFIFHLKVAQT

VGKGTIDASIVDHYVVFGALFGAIGWNIITWYYGIPSSSSHALIGGLVGAAVSKSGWGSLNVDGLMKTVA

FIFISPLLGFILGSLFMLGVSWLYFRTAPSKVDRRFRRLQLLSASLYSLGHGGNDAQKTIGIIWMLLIAS

GYASAASDAPPAWVIGACYLSMGLGTLFGGWRIVRTMGQKITKLKPVGGFCAETGGALTLFIASWMGIPV

STTHTITGAIVGVGATRKLSAVRWGVAGNIVWAWVLTIPASALIAAAGWWIGHRVF

>WP_004190237.1 MULTISPECIES: diguanylate cyclase [Burkholderia]

MNLLSSLSDLVIERVSFGIFVLDREMRVLMWNRFMQDHSGVAAADVIGRNLFDSFPELPRAWLSRKIESV

FQLGSFAFSSWEQRPYLFRFDHDRPITGGVDFMQQDCTFVPIARAREVEAVCVTVSDVTHVSVMQREREE

AVAKLQEYANRDGLTGIANRRFFEARLRDEFSRWQRYGGNLSVLLFDLDHFKTINDRFGHVVGDNVLRVM

AQRVADVVRSQDTFGRFGGEEFALLLPCTSLDEAMLVAEKIRQTIGSTPIEAEGVRVPVTASVGGAAAHA

GALTHETLVNEADAALYRAKRQGRNCSVAFG

>WP_004190182.1 MULTISPECIES: GNAT family N-acetyltransferase [pseudomallei group]

MNAYTLQSIRYNDAPDALRRRFALLHQRVDPDDAFAPDGPPPVIHDPRLDAMSFYASTRSNGEVVSYAAV

VRKTIAHAGKRFSIGGLSWVATDPAHRRAGLGARTVSAATQWMAASGLDIGVFTCDPPLARFYARAGDWP

IAAHVTLIGSRAAGALRSDTLNKVVLMRLFTAKARAAEAELSRATIDLDLPPGQFL

>WP_004189263.1 MULTISPECIES: LacI family DNA-binding transcriptional regulator [Burkholderia]

MGTTIRDVAQAANVSIGTVSRALKNQPGLSEATRARIVEIAHRMNYDPTQLRPRIKRLTFLLHRQHNNFA

TTPFFSHVLHGVEDACRERGIVPSLLTTGPTDDVIRQMRPHAPDAIAVAGFMEPETLEALAATGRPLVLI

DIRAPGLRSINVDNAGGAALAMHHLFASGRRRVAFIGGSLAHYSIAQRALGYRRAFFDAGMLFDPSLELS

IDTSLGPDAGAANAMRQLLDAGGALPDAVFAYNDAAALAAMRVCVERGVRVPEDIAFVGFDDIPAAAHAA

PPLTTLAIDKEALGRQGVELLLADSPAQAELSLPVQLVARASSAGAARAPRTLTSVLGS

>WP_004184857.1 MULTISPECIES: TetR family transcriptional regulator [Burkholderia]

MPPSDHAKMKQGSKAAAPDAEARRDEARRKYDPEQTKRNILDVATQEFSAMGLSGARVDAIAERTNTTKR

MLYYYFDSKEGLYEAVLEKVYGDIRTLERELNVGELEPREGLSRLVEFTFDYHDRHRDFVRLVTIENIHG

AKYIEQLKSFKNRNVSIIKTLEELLARGVESGVFRDDVDPFDLHLLISSFCFHRVANRYTFGTAFGRDPS

SPRLRARHRAMITDTVLRYVAR

>WP_004184602.1 MULTISPECIES: acyl-CoA dehydrogenase [pseudomallei group]

MSLNHAPFALASAFDRYLGDPSMPDNAFSYARCAQLDAHERFPDAVCAMLSQWGLHRYYVPERFGGMLRD

FEQPLQLIRMLARRDLTVAIGHGKTFLGAVCVWVGATHEQASRFARRIGDGAIVSWGLTEREHGSDLLRS

DTYAEADGDEYRIAGEKWLINNATRGDMISLLARTRREGGPRGFDMLLVDKHALSQGRFRPLPKERTHGI

RGADISGIAFDDARIPASARIGARGAGLETVLKGLQLTRTLCASLSLGAGDHALRLALGFACERRLYGRT

LIDLPNARRTLIDAYTDQLLAEAAALVGARSIQALPDEMSVTSAVVKYLVPTRAEAMLDTLAELLGARAF

LADTYAHGMFQKVQRDNRIVSLFDGNTLVNLNAIVNAFQTLAKRYRGRDGADAGQALAAFDLGMPLPAFD

RGALSLVSRHGCRVVGALPALADGIAQRAACEPALSRLVAPVRRLAALTDDVHRRMLACPPVRVHVPVEQ

FELAKRYALCHAAASALGLWWHTRHRVGEASAAPPWRDGLWLRAVLRRVRVALGDAADFDDEAGDALWRA

LLRQHRDGLLFSLLPCELAEHRGEAA

>WP_004528943.1 MULTISPECIES: formate dehydrogenase accessory protein FdhE [Burkholderia]

MTDPTQRILDPSEIASLDHSAIPRVRMPERGAVFAARAARLRKLADLNPIAGYLRLMAAVADAQHELLQT

FVPNAPEKAAAERAQRHSMPLMPALGGERDPRWRDLLQSLLDRVERAGLVNPPLAKLIDGMRLREAAALD

AQADALVAQRFAEIEPASAPFLMAALQVVWTALASRIAPADVPYLEQPGLCPVCGAQPVASIVRVGGQYQ

GYRFLQCGLCSTEWHMVRTKCSHCDSTKGIAYHGIEGGSEAIKAESCDACHTYRKIGYQEKDYDVEPLAD

DLASLTLDLLMGEAGYRRASPNPLLWPDLPAEAGAAAGDERT

>WP_152768689.1 DnaA regulatory inactivator Hda [Burkholderia pseudomallei]

MIRTVTVTRQLTLDLGTPPPSTFDNFYSGTNAELVTRLRELDLALAAGPVADRTFYVWGEAGSGRSHLLQ

ALVHDTTYGHARYVSPQGGLDALAFDPRVSLYAVDDCDALNDAQQIALFNLFNEVRAHPMTALVVAGPAA

PLALDVREDLRTRLGWGLVFHLAPLTDEGKAAVLKHAAKERGIALADDVPSYLLTHFRRDMPSLMSLLDA

LDRFSLEQKRAVTLPLLRAMLAAPEREEPAPGRFK

>WP_004525505.1 MULTISPECIES: co-chaperone GroES [Burkholderia]

MSLRPLHDRVIVKRLDQETKTASGIVIPDSAAEKPDQGEIVAVGPGRRDADGKRVEPDVKVGERVLFGKY

AGQSVKVDGNELLVLREEDIVAVVHS

>WP_004521321.1 MULTISPECIES: transcription termination factor Rho [Burkholderia]

MHLSELKSLHVSELIEMANGLEIENANRLRKQELMFAILKKRAKTGETIFGDGTLEVLPDGFGFLRSPEM

SYLASTDDIYISPSQIRRFNLHTGDTIEGEVRTPKDGERYFALVKVDKVNGQPPEASKHKIMFENLTPLH

PNKPLSLEREMRGEENVTGRIIDMIAPIGKGQRGLLVASPKSGKTVMLQHIAHAIKQNHPDVILFVLLID

ERPEEVTEMQRSVAGEVIASTFDEPATRHVQVAEMVIEKAKRLVEMKHDVVILLDSITRLARAYNTVIPA

SGKVLTGGVDANALQRPKRFFGAARNIEEGGSLTIIGTALIETGSRMDDVIYEEFKGTGNMEVHLERRLA

EKRVYPSINLNKSGTRREEMLIKPEILQKIWVLRKFIHDMDEVEAMEFLLDKIRQTKNNAEFFDLMRRGG

>WP_004186469.1 MULTISPECIES: tRNA glutamyl-Q(34) synthetase GluQRS [Burkholderia]

MTRYRGRFAPSPTGPLHFGSLVGALASWLDARAWGGAWLVRIEDIDGPRTVPGAAEDMLATLRGFGFIAD

EPPVWQSARVAHYEAALARLTAAGLVYPCGCSRKEIADSLRAAHERHTTLAYPGTCRTGLHGKPARAWRL

RVPDGAAAVVAFDDRWQRAQTQNLATEVGDFVLKRADGQWAYQLAVVVDDGDANITHVVRGADLLDSTAR

QIYLQRCLGLPTPRYLHVPVVLDANGEKLSKQTGAAALDPAAPLPALAAAARHLGLALDGAACASLDAFQ

AAAIAAWDARFGPNARG

>WP_162487073.1 flagellar basal body P-ring protein FlgI [Burkholderia pseudomallei]

MVKTMRTLFARVVRPFVAARRRAAACCALAACMLALAFAPAAARAERLKDLAQIQGVRDNPLIGYGLVVG

LDGTGDQTMQTPFTTQTLANMLANLGISINNGSANGGGSSAMTNMQLKNVAAVMVTATLPPFARPGEAID

VTVSSLGNAKSLRGGTLLLTPLKGADGQVYALAQGNMAVGGAGASANGSRVQVNQLAAGRIAGGAIVERS

VPNAVAQMNGVLQLQLNDMDYGTAQRIVSAVNSSFGAGTATALDGRTIQLTAPADSAQQVAFMARLQNLE

VSPERAAAKVILNARTGSIVMNQMVTLQNCAVAHGNLSVVVNTQPVVSQPGPFSNGQTVVAQQSQIQLKQ

DNGSLRMVTAGANLADVVKALNSLGATPADLMSILQAMKAAGALRADLEII

>WP_155018364.1 uroporphyrinogen-III C-methyltransferase, partial [Burkholderia pseudomallei]

MNTMGKVTLLGAGPGDLDLLTMKAAKALAAADVLLLDDLVDPGIVALAPRARVIRVGKRGGCRSTPQAFI

ERLMCRYALRGAHVVRVKGGDVLLFGRAGEELAALRAARVPVEIVNGISSGFAAAASLGVSLTHRDHCQG

VTFVTAHLQDHGAVPYTHLRPHEPVLD

>WP_122883406.1 sn-glycerol-3-phosphate ABC transporter ATP-binding protein UgpC [Burkholderia pseudomallei]

MARIVCTGLTKRYDGGAPVLHPLDLEIGDGEFIVLLGPSGCGKSTMLRMIAGLETITGGTLAIGGAVVND

LAARERNVAMVFQNYALYPHMSVYENIAFGLRRLKVPAAEIDRRVREVARVLGLDALLDRKPRAMSGGQQ

QRAAIARAMIKTPDVFLFDEPLSNLDAKLRAQLRGDIKRLHQRLKTTTLYVTHDQLEAMTLADRVVLMRG

GRIEQLGTPAELYGCPHTVFAAGFVGTPAMNFADGVIERTAGHVALAAGGARWTLAARRFAGLPDGLRVK

LAIRPNYLRIAPGAHAPAATLALEGRVELVELLGAEALVTFGCNGAPFAALVPASAAPALGAVVTFTFDE

RDLHLFDAATGRNVMLPEAGAPADAERPGAAMRPSPGAPHRFTMSRPSIRTRSRNGIFIRSTKST

>WP_024429081.1 protein-disulfide reductase DsbD [Burkholderia pseudomallei]

MFNRIPRHAQSRFCFLIAVVAMLGVLFGTSLAARAADDFLDPAVAFKFSASEAPGQVDVHFKIADGYYMY

RERFAFAVKSGSATLGEPQLPAGHVKFDPTFQKNVETYRGDLTIHLPIKQASGPFELAVTSQGCADEGIC

YPPAEHVARIEGAALGAAGTAPAAAGAGADTSAADGGSWYERVTSADYARSLLEGHGFLTIVALYFVAGM

VLSLLPCSYPMIPILSAIIVGEGARATRARAFALSLTYVIGMALVYTALGVAAALVGQSLGAWLQNPWVL

GAFALLLTVFALLLIGGVDITLPQRWQNGAAQTSGPRKGGRFAAVATMGALSALVVGACMTAPLFAVLAF

IAHTGNALFGGAALFSMGIGLGVPLLVIGIGAGTLLPRAGAWMDGVKVFFGVLLLAAALWIVWPVLNAAS

QLGLGALWLLIAAAALGLFTPHSGSSSVWRRLGRGLGAALAIWAATLLVGLAAGSTDPLRPLAVLAARAA

PSNGAAGAGAGAHEGPAFAPVRSIAELDEIVKTSTRPVMLDFYADWCVSCKEMEHLTFTDARVGARLSQM

HLVRADVTANSPDDQALLKRFGLFGPPGIIVFDANGQERGRVVGYQSADRFLRSLDRMSLPAAWSAS

>WP_024428955.1 cation/acetate symporter ActP [Burkholderia pseudomallei]

MLRQSTARCARLMPAALFACAALAPAAARAMGAAAAPMPEKVVPNPVAIGMFFVFVFATLALTRWAARRT

RSARDFYTAGGGITGLQNGLAIAGDYMSAASFLGLSGMVFMFGFDGLIYSIGFLVGWPFVMFLIAEPLRN

LGKFTFVDVVAYRFAQRPIRLLTSASALTIVVQMVGAGKLIQLLFGLSYGVAELIVGVLMVVYVFFGGMT

ATTWVQVIKAVLLLAGATLLAALALGEFGFSLDEMFRRAVAVHPGALGIMGPGKLIRDPANALSLGIALM

FGTAGFPHILMRFFTVPNAKEARKSVLYATGFIGYFYLLTFVIGFSAIVLLAQHPEFFRHDAAGAFNLTR

DLVGGSNMVAVKLAQAVGGNWFYGFIAAVTFATILAVVAGLTLAGATTISHDLYAQMWARGKPHERREMR

ISRAATLALSAVAIGLSILFEHVNVAFMVGLVAAVAASANFPVLATSIFWRGMTTRGAVAGGGLGLASAV

ALTVLSKSVWVDVLHHAHAPVFLDNPALVSVPLAFAGIVIGSLTDRSERARRERDAFARQEFYAQTGALA

TRAATH

>WP_024428946.1 potassium-transporting ATPase subunit KdpB [Burkholderia pseudomallei]

MTQHSATRSMFDPALVRPAIVDSFKKLTPRTQLRNPVMFCVYVGSILATILWIAALTGQAEAPAGFILAV

ALWLWFTVLFANFAEAIAEGRSKAQAASLRSAKKDVMAKKLNEPHPKSPVRIMTASDLRRGDVVLVEAGD

TIPADGEVIEGVASVDESAITGESAPVIRESGGDFSSVTGGTRVLSDWIVVKVTANPGEAFLDRMIAMVE

GAKRKKTPNEIALTILLVALTIVMLLATATLLPFSMFSVDAMHSGYVVTITALVALLVCLIPTTIGGLLS

AIGVAGMSRMMQANVIATSGRAVEAAGDVDVLLLDKTGTITLGNRQASAFLPAPEVTEATLADAAQLSSL

ADETPEGRSIVVLAKERFNIRQRDMGALHAVFLSFSAHTRMSGVDLSPEREIRKGAADAVKKYVESHGGR

FPAEVTNAVTDVARRGSTPLVVAEKRGALARVLGVIELKDIVKGGIKERFAELRKMGIKTVMVTGDNRLT

AAAIAAEAGVDDFLAEATPETKLSTIREHQAAGRLVAMTGDGTNDAPALAQADVAVAMNTGTQAAKEAGN

MVDLDSNPTKLIEIVEIGKQMLMTRGSLTTFSIANDIAKYFAIIPAAFATTYPQLNALNVMHLATPASAI

MSAVIFNALIIVLLIPLALKGVKYRPLGAATLLRRNLLVYGLGGILVPFVGIKLIDVVLAALGWV

>WP_024428785.1 histidine kinase [Burkholderia pseudomallei]

MRRVRGLAGLVLFNCIVGLSSWASRRDDAVLPYLVLANGIGLSALLLSLCADKLTRGKLAVLPKVLIVAP

VSVLAGFEIAASTIGHAPHLIGRASVREWLGYGSSFVVTGIACAFVSLFMQAARMRASLETQRREAAEAR

QAETAARLALLQAQIEPHFLFNTLANVQSLIERDPARASTMLDSLNRYLRASLGRTRKAASTLGEELELI

EALLQIASIRLGERLRYAIDVPAPLRELAFSPLLLQPLVENALLHGIEPSLDGGEIRVCGRRNGKLLELS

VIDTGVGLGNGETRLHGGVGLANVVARLTSLYGARGRVSVGATAGATRGVTATLQIPID

>WP_024428609.1 Nramp family divalent metal transporter [Burkholderia pseudomallei]

MNSRPLAARSPSSRRPSDSGGPGGGRRWAAFVGAGVLVAVGYMDPGNWATALAGGAGYGYALLGVVIAAS

LMAMLLQWLAARIGVVSGRDLAQLCRERFDPRATVLLWIAAEIAIVACDVAEVVGAAVALQMLLGVSLST

GVLVSAVGTLAMLALERHGRRTLEAAIASLIFFVGLCFVIEVALARPDWHAALAGAAPSPRLLREPGMLW

LAAGILGATVMPHNLYLHSMLVKHHASARDEPSIRRALFGVNLDTFVSLGFAFVINAALLIVAAAVFHAS

GHDDVTDLADAHRLIAPLVGNRWAGPLFAAALLACGLSATVTGTLAGQAIMEGFLKLTLPRWQRALLTRA

LAIGPALAAVGLFGQHGSNQLLVASQVVLSLQLPLAMVPLVVFASSRALMGRWRVRGLPLAAAWMCAAAI

VALNGALVWQVVAAG

>WP_024428584.1 dipeptide ABC transporter ATP-binding protein [Burkholderia pseudomallei]

MSASRAAPSLPDARVLAVDGLTVTFRREDAAFVAVRDLSFHVDRGETLAIVGESGSGKSVTSLALMRLVE

HGGGAIAGGAIALRRRGGAVLDLARATPSTLRTVRGADVAMIFQEPMTSLNPVFTVGDQISEAIALHQHK

SAGEARAETLRLLDLVRIPEARRVFARHPHQLSGGMRQRVMIAMALSCRPALLIADEPTTALDVTIQAQI

LQLIRGLQDEMDMGVIFITHDMGVVAEVADRVLVMYRGEKVEEGACDAIFAAPSHPYTKALLAAVPRLGS

MRGTDAPAKFPLLRFDPAAGDALVVAGGDATAASGDAAREAVLFVDSDAAAASAASTVSTVSAVSADSAA

SAAPTACARPAIDAGAPPLLRVRELVTRFPVKSGVFGRVSQYVHAVERVSFELRAGETLALVGESGCGKS

TTGRSLLRLVERVSGSIEFEGREIGALKGRELQALRRNIQFIFQDPFASLNPRLTVGFSIMEPLLVHGVA

SGRQAQARVDWLLERVGLPADAARRYPHEFSGGQRQRIAIARALALNPKVVVADESVSALDVSVQAQIVN

LMLDLQRELGVAYLFISHDMAVVERISHRVAVMYFGQIVEIGPRRAVFETPRHPYTKKLMSAVPIADPAR

RHAPRTLPADELPSPIRALGDEPEVAPLVAVGPAHFVAEHRVGGAY

>WP_011205239.1 MULTISPECIES: glutamate/aspartate ABC transporter substrate-binding protein [pseudomallei group]

MTTMKFPKAMLMVAALSTFAGGAIAQETGTLKKIKDTGVIALGHRESSIPFSYYDQNQQVVGYSRDFQMK

VVDAVKKKLNLPNLQVKNIPVTSQNRIPLVQNGTVDIECGSTTNNLDRQKQAAFSDTIFVIGTRLMTKKD

SGIKDFADLKGKTVVTTAGTTSERLLREMNNKNQMGMSIISAKDHGESFQTLETGRAVAFMMDDALLAGE

RAKAKQPGEWVIVGKPQSQEAYGCMMRKDDPAFKKVVDDAIVQVEKSGEAAKIYSKWFENPIPPKGLNLN

FPLSDEMKKLYANPNDKALD

>WP_004554234.1 histidine ABC transporter permease HisQ [Burkholderia pseudomallei]

MRLSASKRRGRACAVPITITETSVFLYGFGPLIWAGTVQTIELSVLSLAAAVLLGLVGAVAKLSHNRVLR

AIATGYTTLIRSVPDLVLMLLLFYSIQIWLNQFTDLMSWDQIDIDPFVAGVLTLGFIYGAYFTETFRGAF

LAVPRGQLEAGSAYGMNGMRVFARIMFPQMMRFALPGIGNNWQVLVKATALVSIIGLADVVKAAQDAGKS

TFNMFFFILVAALIYLAITTASNLVLGQLEKRYSIGVRHAEL

>WP_004548171.1 nucleoid occlusion factor SlmA [Burkholderia pseudomallei]

MQPTHPHDPAVTAAAENERSAATANRTRPKPGERRVHILQTLASMLEAPKSEKITTAALAARLDVSEAAL

YRHFSSKAQMFEGLIEFIEETFFGLVNQIAANEPNGVLQARSIALMLLNFSAKNPGMTRVLTGEALVGEH

ERLAERVNQMLERVEASIKQCLRVALLEAQAHAAGGAPPPVPLPDDYDPALRASLVISYVLGRWHRYAKS

GFTKAPGEHADAQLRLILQ

>WP_004535527.1 MULTISPECIES: sn-glycerol-3-phosphate ABC transporter ATP-binding protein UgpC [Burkholderia]

MASLSIRDVYKTYPNGVPVLKGVDIDIEDGQFLILVGGSGCGKSTLLNMIAGLETVTKGEIRIGDKVVND

LSPKDRDIAMVFQSYALYPSMTVRENISFGLNIRKVPKNEQKQIVDRVAAMLQIEHLLERKPGQLSGGQR

QRVAMGRALARDPALFLFDEPLSNLDAKLRIEMRAEIKLLHQRLGTTIVYVTHDQIEAMTLGDRIAVMKD

GVVQQFGAPQDIYDSPSNLFVAGFIGAPPMNFINGKLVEQGSGVGIELDTGAMRGVLNLPFDAKRMNGHV

GRDVILGLRPERITDARSAHNGEGARLQPVDVTVDVTEPTGPDTHVFAQVNGKRIVSRVHPAANPQPQQK

LSLLFDVSKAVLFDPSTEARIA

>WP_004535091.1 MULTISPECIES: SDR family oxidoreductase [Burkholderia]

MHAWSAQHVPPQGGKVAVVTGANSGLGWQIAQTLAAKGAQVVMGCRDTAKGELAAHAIRTRYPRARIEVE

ALDLADLASVCRFADAVADRHGRVDILCNNAGVMFLPLRHTRDGFEMQMGTNHLGHFALTGLLLPALRAS

HRARVVTMSSGFNRLGKIRLDNMLAERGYNKYRAYCDSKLANLMFTLELQRRFDQACLPILSVAAHPGYA

ATHLQFAGPEMANSSLGTFAMRLSNRLVAQSADVGALPAIHAATAVDVDGGAYIGPAHLCETRGYPAEAR

IPRQARDVRMGKRLWEKSEQLTGVRYLDTPPPPGSRRRASRDDATFGAL

>WP_004534248.1 MULTISPECIES: NADPH-dependent 7-cyano-7-deazaguanine reductase QueF [Burkholderia]

MNPEHSPLGKATVYANQYDASLLFPIPRAGAREQIGIGAPLPFFGTDIWNAYELSWLNARGKPQIAIATF

YVPAESPNIVESKSFKLYLGSFAQTAFESADAVRDALKRDVSAACGASVTVRLATPAEFRKLQMDELDGL

SLDRLDLDAHVYETDPSFLTASHGEAPVEETLVTDLLKSNCPVTGQPDWGSVQIHYVGAPIDHAGLLRYI

ISFRNHTGFHEQCVERIFVDILRACQPVKLAVYARYTRRGGLDINPFRTNYNQPMPDNARTARQ

>WP_004534144.1 MULTISPECIES: L-glyceraldehyde 3-phosphate reductase [Burkholderia]

MAYEAASERYADMQYRVSGKSGLKLPALSLGLWHNFGDTTPISTQREILRTAFDLGITHFDLANNYGPPY

GSAETNFGRLLREDFKPYRDELLISTKAGWDMWPGPYGSGGGSRKYVLASLDQSLRRMGLDYVDIFYSHR

FDAHTPLEETASALASAVQQGKALYVGVSSYSAASTREIAKLLAEYKVPLLIHQPAYNMLNRWIERELLD

ALDETGSGCIAFTPLAQGLLTSKYLNGVPADARINKPGGGSLKEAHLSAENLEHVRKLNEIAQRRGQSLA

QMALAWVLRDSRVTSALIGASRAEQVRENVAALAHLAFSDDEIAEIDRYATEGGINLWEKPSTDQAI

>WP_004531992.1 lipoprotein signal peptidase [Burkholderia pseudomallei]

MAKTLSKSSGGALAPWLGISLIVILFDQLTKIAVLKTFAYGAMHALTPFFNLTLIYNRGAAFGFLATAGG

WQRWAFTALGIGATLVICYLLKRHGHQRLFSLSLALILGGALGNVIDRLIYGHVIDFLDFHVGAWHWPAF

NLADSAITVGAVLLIYDELRRVRGTR

>WP_004531833.1 MULTISPECIES: fumarate/nitrate reduction transcriptional regulator Fnr [Burkholderia]

MLQAQTQNAYTPAQQRVTVPLRAAKRDDAAKRTAARCSSCAMRSVCMPVDLNTHEFAKLDALICSTRQVK

RGDTLFRTNDGFQSIYAVRTGSFKTVVMHRDGQEHVTGFQIVGETLGLDGVCAGHHNSDAVALEDSTVCI

IPFAQLEAVCREVKPMQHHVHRLMSGEIVRESSQMMLLGTMTAEQRVAAFLLNISERFQKRGYSAAEFNL

RMTREEIGCYLGMKLETVSRMLSKFQRDKLIAPRGKQIRIIDPVGLARV

>WP_004531309.1 sigma-70 family RNA polymerase sigma factor [Burkholderia pseudomallei]

MESRLRALFVSGLAGHAPDYRSFLSELTRHLRGFLRKRIPQFDADIEDLVQEILLAVHNARHTYRADEPL

TAWVHAIARYKLMDFFRTRARREALHDPLDDHTDIFSEPDDDASDAHRDIGRLLDHLPDKQRLSILHVKL

QGLSVAEAAQLIGASESVVKVSIHRGLKALAARVRGVV

>WP_004530071.1 MULTISPECIES: high-affinity branched-chain amino acid ABC transporter ATP-binding protein LivG [Burkholderia]

MSATTEMLKVSALQMRFGGLLAVDGVDFDVRRDEVFAIIGPNGAGKTTVFNCIGGFYRPTGGRVVLDGHS

IGGLTSHLVARKGLVRTFQNIRLFQSLTVVENLLVAQHRQVKSGLLHGLFATPAYRRAEREALERAAMWL

ERMNLTAVANRPAGTLSYGHQRRVEIARCMITNPRLLMLDEPAAGLNPQEKVELQHLIDRLRREFGISVL

LIEHDMSLVMGVSDRILVMEHGRPITIGRPDEVRNDPRVIKAYLGEE

>WP_004529578.1 MULTISPECIES: fumarate/nitrate reduction transcriptional regulator Fnr [Burkholderia]

MLTPVARPAAAAAPVHASSTWAPRQAAHCSTCAMRHLCMPQGLAPEALARLESVICTARPVRRGETLFRE

GDTFDNLYAVRSGSLKTIATRHDGREQVTGLHLAGEALGLDGICDDAHPRTAVALEDSSVCVIPYSALKT

LCSEAGTMQLRMHKLMSEQIVRETSQTMVLGSLNAEERVAAFLLDVSSRYMKRGYSPNEFNLRMTREDIG

SYLGMTLETVSRTLSKFHKRGLIEMQGRMVRIVDFDGLHRL

>WP_004524345.1 MULTISPECIES: type III secretion system export apparatus subunit SctR [Burkholderia]

MVQFSDITGLLLVVIAISLLPFIAMVVTSYAKIVVVLGLLRNALGVQQVPPNMVLNGIAILVSLYVMAPI

GMQAAKALDEQQLASQSSQAIIQALGSAREPFRSFLEKHTPEREKRFFIRSASVIWPKEEASLLNERDLI

VLAPAFALSELTDAFKIGFLLYIVFIIVDLVIANVLLALGLNQITPTNVAIPFKLLLFVAMDGWSTLIHG

LVMTYK

>WP_004523579.1 glutamate/aspartate ABC transporter substrate-binding protein [Burkholderia pseudomallei]

MMKPLRPLLSIAVGCALAAAAHADDAGPTLKKIRDTGVVTLGVRESSVPFSYYDQQQRTIGYSQEIALKI

VDEIKKTLNRPNLTVREIPITSQNRIPLVQNGTVDLECGSTTHTKERANQVSFSNSIFQYGMRLIVKKSS

GVKDFPDLAGKTVATTAGTTEERLLRQWNAEKGMAMQIISAKDHADAFLNVKSGRAVAFFMDEPLLYGAK

AKEANPGDYVITGNSPVSEAYGCMLRKDDPGFKQLADRVIARMQRSGEAEALYVKWFNRPIPPKGVNLDY

PLSADMKQLFANPNDKALD

>WP_004522525.1 MULTISPECIES: histidine ABC transporter permease HisQ [Burkholderia]

MFLQGYGPLILSGTWQTVKLAVLSLALSFLLGLVGAAAKLSRNRVSNGIGTLYTTLIRGVPDLVLMLLLF

YSLQIWLNQLTDLMNWDQIDIDPFAAGVLVLGFIYGAYFTETFRGAFLSVPRGQLEAGSAYGMTNWQVFA

RVMFPQMMRFALPGIGNNWQVLVKSTALVSIIGLADVVKASQDAGKGTLRFFFFTLLAGAIYLAITTISN

FVLMWLEKRYSTGVRKADL

>WP_004200262.1 MULTISPECIES: flagellar hook-associated protein FlgL [Burkholderia]

MRISSTQLYSLNVDMMNDQQAQIAQLYQQVSSGISLTTPADNPLAAAQAVQLSATSATLAQYTQNQTIVQ

TALQTEDTTLTSVNDVLNAAYQALMHAGDGGLSDSDRAALAAQIQGSRDHLLTLANTADGAGNYLFAGFQ

PTTQPFSNKPGGGVTYAGDYGARAVQIADTRTVSQGDNGANVFMSVPFLGSLPVPAAGASNTGTGTIGAV

SITNPSDPTNTHQFTITFGGTAAAPTYTVTDNSVTPPTTTAAQAYSSGQGINLGGQTVAVSGKPAVGDTF

TVTPAPQAGTDVFATLDTVIAALKSPVGNSQTASTALTNTMATASTKLMNTMTNVLTVQASVGGRLQEVK

AMQAVTTTNTLQTTNSLSNLTDTNLPAAISQFLQLQNSLSAAQKAFVQMQNLSLFQYLNP

>WP_004199919.1 MULTISPECIES: ABC transporter permease [Burkholderia]

MSGFRTLFYKEILRFWKVSFQTVLAPVVTALLYLTIFGHALTGRVNVYPGVEYVSFLVPGLVMMSVLQNA

FANSSSSLIQSKITGNLVFMLLPPLSSADIFGAYVLASVVRGLAVGAGVFVVTVWFIPMSFAAPLYIVAF

ALFGSAILGTLGLIAGIWAEKFDQLAAFQNFLIMPLTFLSGVFYSTHSLPPVWREVSRLNPFFYMIDGFR

YGFFGIADVNPLASLSVVAGFFVLLALIAMRLLATGYKLRH

>WP_004199035.1 MULTISPECIES: sn-glycerol-3-phosphate ABC transporter ATP-binding protein UgpC [Burkholderia]

MASVLLRNIGKRYDDNEVLRNVNLDIADGEFVVFVGPSGCGKSTLMRMIAGLEEISGGELLIDGAKVNDV

PSAKRGIAMVFQSYALYPHMTLYDNMAFGLKLAGAKKQEIDDAVKQAARILHIDHLLDRKPRQLSGGQRQ

RVAIGRAITRKPKVFLFDEPLSNLDAALRVKMRLEFARLHDELKTTMIYVTHDQVEAMTLADKIVVLSAG

SVQQVGAPNALYHAPANQFVAGFIGSPKMNFLAGVVESASADGALVRFESGETQRAAVDASTLRAGERVT

VGIRPEHLHVGVTGGDGIVARTMAVESLGDAAYLYAESAVAPDGLIARIPPLDTYRAGEMLRVGAQPEHC

HLFDADGRAFKRKPRHALAA

>WP_004198660.1 MULTISPECIES: histidine ABC transporter permease HisQ [pseudomallei group]

MLFQGFGPLLWAGTIETVKLAVLSLAASLVLGLAGAAAKLSSNRALASVGTFYTTLIRAVPDLVLMLLLF

YGIQILLNDVTDMLGAEQIDIDPFVAGIVTLGFIYGAYFTETFRGAFLAVPRGQLEAGFAYGMGAWRVFA

RIMFAQMMRFALPGIGNNWQVLVKATALVSIIGLADVVKAAQDAGKSTLNFFFFTLAAGAIYLAITTLSN

LVLMYLEKRYSAGVRRVAL

>WP_004198657.1 MULTISPECIES: UDP-N-acetylglucosamine 1-carboxyvinyltransferase [Burkholderia]

MSNLIVHGGAPLRGEITPSANKNAVLPILCATLLTDRPLRLVGVPDITDVRKILDIFRTLGSDVSIDYAS

GVLDLHHRATAFDPAVHRLPEEMRSSIMLVPPLLARFGVARLENDVKGCTLGVREIDPHVEVFERFGARI

ERTSDSLIVRADGPLTPNHHWLDYASVTTTENFVLCAASANGTSTLVNAASEPHVQEFCRFLAMLGVPIE

GIGTSHLSVQGGRALAGGEYRFNEDFHEIATFLALGAITGGDIAVRNGSPEQFPLIDRTFAKFGVQVTHE

NGWSHALRDGPLKVKQPFTRNILTKVEAAPWPYLPVDLLPIFIALGVQAQGSVMFWNKVYDGAMGWTGEL

SKFGAHVFLSDPHRLITFGGLPLSPARVESPYIIRVAIALLMVAASIDGRSEILNAQPIRRAHPHFVENL

RSVGANVEWTSGE

>WP_004198210.1 MULTISPECIES: dTDP-4-amino-4,6-dideoxy-D-glucose aminotransferase VioA [Burkholderia]

MSALPLRAVEPWIDERIFVTQPHLAPLAEFLPYLEAIWESKVLTNGGPFHQQLEKALCDYLGVRHLALFT

NGTLALVTALQALRITGEVITTPYSFVATAHSLLWNGIKPVFVDIDPSTLNLDPAKIEAAITPQTTAIMP

VHCYGRPCDVAAIQKIADNYNLKVIYDAAHAFGVKTPDGSVLEHGDMSILSFHATKVFNTFEGGAIVCPD

AKTKQHIDHLKNFGFVDEVTVVAAGINGKMSEINAAFGLLQLKHIDDALARRAKIDAAYRDALARVPGIR

CLPPVDAPVANHSYFPILVGDDYPMSRDALYQRLRDHQIYARRYFYPLISDFPMYRGLPSAQRGNLPVAA

DAAARVLCLPIYPALSDASLERIVSLMAGAHA

>WP_004198205.1 MULTISPECIES: 30S ribosomal protein S21 [Burkholderiaceae]

MTTILLKENEPFEVAIRRFRRAIEKNGLIAELRERQAYEKPTAVRKRKKAAAVKRLHKRLRSQMLPKKLH

>WP_004198009.1 MULTISPECIES: molybdopterin-synthase adenylyltransferase MoeB [Burkholderia]

MNDEQLLRYSRHILVDEIGIEAQQRFLDAHAIVVGAGGLGSPAAMYLAAAGVGTITLVDADTVDLTNLQR

QILHATDSVGRKKVESGRDALARLNPDVKVNAVAERVDDAWLNAHVPHASVVLDCTDNFATRHAINRACV

AHRVPLVSGAALRFDGQISTFDFREPGSPCYACVFPEDQPFEEVACSTMGVFAPTVGIIGAMQAAEALRV

IGGIGATLVGRLMMLDSLRMEWNTMKIARQPDCPVCGAGGAH

>WP_004197255.1 MULTISPECIES: flagellar hook protein FlgE [Burkholderia]

MGYQQGLSGLAGASSDLDVIGNNIANANTVGFKGSTAQFSDMYANSVASAVNNPIGIGTMLASVQQQFSQ

GTITSSTSSLNVAINGNGFFQMSNNGVTTYSRDGTFQRDKNGYIVNSQGLNLMGYAANANGVINTAATVP

LQAPTTNIAPTATTKITGQFNLNSQDAVPATTPFNYTDPTSYNYTTSVQVFDTLGGSQNVNLYFVKSATS

GQWEAYAGPAGKTPTDLGSVKFSTAGTITGTSTPAGVPTTNVGQFSFSIPTTTGAANPQNLTLDLTGTTQ

YGGKNGINNLAQDGFASGVLTTFSIGADGKVTGNYSNGQTSTLGQIVLANFNNPNGLVNVGNNQYVESAA

SGVPQISAPGSTNHGTLQGSALENSNVDLTSQLVKLITAQRNYQANAQTIKTQQTVDQTLINL

>WP_004196764.1 MULTISPECIES: iron donor protein CyaY [Burkholderia]

MSDTDYLTRAEAVLAAVERSVDAANDGDADIDLERNGSVLTLTFENGSKIIVNLQPPMKEVWIAAKAGGF

HYRFVDGAWRDTRSGDEFFAALTGYATQQAGMPIAFSA

>WP_004195084.1 MULTISPECIES: L-arabinose ABC transporter permease AraH [Burkholderia]

MQARENLPPAAAHAAAVPTEDRQRWRQHAADYSLVAIFAAMFVAMSLTVDHFFSIDNMLGLALSISQIGM

VACTMMFCLASRDFDLSIGSTVAFAGVLCAMVLNATDNTFVAIAAAVAAGAVIGFVNGAVIAYLRINALI

TTLATMEIVRGLGFIVSKGQAVGVSSETFIALGGLTFFGVSLPIWVTLACFVVFGVLLNQTVYGRNTLAI

GGNPEASRLAGINVERTRVYIFLIQGAVTALAGVILASRITSGQPNAAQGFELNVISACVLGGVSLAGGR

ASISGVVIGVLIMGTVENVMNLLNIDAFYQYLVRGAILLAAVLLDQLKNRGARD

>WP_004195011.1 MULTISPECIES: cytosine deaminase [pseudomallei group]

MKLINATLRKRSGLFSIVLDGGAIASVTPQPARVDAPHAPRADVIDADGKLVIPPLVEPHIHLDAVLTAG

EPEWNMSGTLFEGIERWAQRKATITHEDTKARAHAAIGMLRDHGIQHVRTHVDVTDPSLAALKAMLEVKD

EARGLIDLQIVAFPQEGIESFGGGRALMERAIEVGADVVGGIPHFENTREQGVSSIRFLMDLAERSGCLV

DVHCDETDDPNSRFLEVLAEEARVRGIGARVTASHTTAMGSYDNAYCSKLFRLLKRSQINFISCPTESIH

LQGRFDTFPKRRGLTRVAELDRAGMNVCFGQDSIRDPWYPLGNGNILRALDAGLHICHMMGYQDLARSLD

FVTEHSARAMHLGERYGIEPGRPANLVVLDASDDYEALRRQAKALLSIRGGEVIMRRVPERIAYPAAR

>WP_004193901.1 MULTISPECIES: CysB family HTH-type transcriptional regulator [Burkholderia]

MNFQQLRFVREAVRQNMNLTEVANVLYTSQSGVSKQIKDLEDELGVDIFIRRGKRLTGLTEPGKAVHQLI

ERMLLDAENLRRVARQFADQDSGHLVVATTHTQARYALPKVIRQFTEVYPKVHLALRQGSPQQIAQMILN

GEADLGISTEALDRFPDIVTFPCYSWHHVVVVPKGHPLVGRENVTLDDIAEYPIITYDQDFTGRSHIDQA

FANAGAVPDVVLTAIDADVIKTYVELGMGIGVVAAMAYDAQRDTGLVALDTQHLFEASTTRVGLRRGAFL

RAYAYRLIEMFAPHLSEGEIAGQLREAA

>WP_004193435.1 MULTISPECIES: CysB family HTH-type transcriptional regulator [Burkholderia]

MNLHQFRFVREAVRQNFNLTEAAKALYTSQPGVSKAIIELEDELGVEIFTRHGKRVRSLTEPGRIILASV

ERILQEVESLKRVGKDYAAQDQGNLTIAATHTQARYSLPAAIAEFKKRFPKVHLSILQGSPTQVAEMVIH

DQADLAIATEAIADYKELVSLPCFQWHHAAVVPADHPLLERKPLSLDDLAQFPLITYDDAFAGRKKINQA

FALHRLTPDIVLEAIDADVIKTYVELGLGVGIMADIAFNPERDRNLRAIAVGHLFGSNVTRVALKQGAYL

RGYIYTLVELLSPTLNRKLVETALKGEAETYEL

>WP_004193360.1 MULTISPECIES: 30S ribosomal protein S18 [Burkholderia]

MARPTGKKFDKRRQQQNPLFKRKKFCRFTAAGVEQIDYKDTETLKDFIGENGKITPARLTGTKAHYQRQL

DTAIKRARFLALLPYTDQHKA

>WP_004192125.1 MULTISPECIES: phosphate regulon transcriptional regulator PhoB [Burkholderia]

MPSNILVIEDEPAISELISVNLQHAGHCPIRAYNAEQAQSLISDVLPDLVLLDWMLPGKSGIAFARDLRN

NERTKHIPIIMLTARGDEQDKVLGLEIGADDYVTKPFSPKELMARIKAVLRRRAPQLTEDVVSINGLRLD

PATHRVAAQSDGSEIKLDLGPTEFRLLHFFMTHPERVHSRTQLLDQVWGDHVFVEERTVDVHIKRLRAAL

KPAGCDAMIETVRGSGYRLAKHA

>WP_004191744.1 MULTISPECIES: ribonuclease E activity regulator RraA [Burkholderia]

MMFATTDLCDAHEDRLAAGTLRVLEPVFRPFGGVRRFAGPAATLKLFEDNSLVRTALEQDGAGRVLVVDG

GGSLRCALVGGNLGKLAEKNGWAGIVVNGCVRDSDELAECRVGVLALAAHPRKSDKRGAGVSDAPVDVRG

TRIVPGDWIYADADGVLVSDDALLE

>WP_004190805.1 MULTISPECIES: Pr6Pr family membrane protein [Burkholderia]

MQKAAFVAAYRLVGCGLILSATCHSIARRWNAPTFRLDNFLSYFTQLSSLYTAIVLLAGLWLATKPPSRR

YESARGAVVLYMAITGIVYELLLAHLDAVHHATPYYTNWILHRVIPIAVFLDWLYVAPRVRIDWSQLARW

LAFPVAYLGYTLVRGALIDWYPYPFVDPRAHGYLMVAAYSGAIAAGSIGFAALIVLLGNRTGAPAPQAEH

A

>WP_004190342.1 MULTISPECIES: 30S ribosomal protein S21 [Burkholderia]

MTIIRVKENEPFEVAMRRFKRTIEKNGLLTELRAREFYEKPTAERKRKKAAAVKRHYKRIRSQMLPKKLY

>WP_004190136.1 MULTISPECIES: acetyl-CoA C-acetyltransferase [Burkholderia]

MTAVDQDPIVIASAARTPIAGFQGEFASLAAPQLGAAAIAAALERAGLQPEQIDEAVMGCVLPAGQGQAP

ARQAALGAKLPLSVGCTTINKMCGSGMRAAMFAHDMLVAGSVDVIVAGGMESMTNAPYLLPKARAGMRMG

HGQVLDHMFLDGLEDAYDKGRLMGTFAEECAGEYAFSRDAQDAFAIESLARAKRANEDGSFAWEIAPVTV

AGKKGDAVIARDEQPFKANPEKIPTLKPAFSKTGTVTAANSSSISDGAAALVMMRASTANRLGLAPLARV

VGHSTFAQAPSKFTTAPVGAIRRLFEKNGWRAAEVDLYEINEAFAVVTMAAMKEHGLPHEKVNVNGGACA

LGHPIGASGARILVTLIGALRARGAKRGVASLCIGGGEATAMGIELI

>WP_004190034.1 MULTISPECIES: bifunctional UDP-N-acetylglucosamine diphosphorylase/glucosamine-1-phosphate N-acetyltransferase GlmU [Burkholderia]

MNIVILAAGTGKRMRSALPKVLHPLAGRPLLSHVIDTARALAPSRLVVVIGHGAEQVRAAVAAPDVQFAV

QEQQLGTGHAVRQALPLLDPSQPTLVLYGDVPLTRTATLKRLADAATDARYGVLTVTLDDPTGYGRIVRD

QAGCVTRIVEQKDASPDELRIDEINTGIVVAPTAQLSMWLGALGNDNAQGEYYLTDVVEQAIEAGFEIVT

TQPDDEWETLGVNSKAQLAELERIHQRNLADALLAAGVTLADPARIDVRGTLACGRDVSIDVNCVFEGDV

TLADGVTIGANCVIRHAAIAAGARVDAFSHLDGATVGANAVVGPYARLRPGAVLAADAHVGNFVEVKNAT

LGQGSKANHLTYLGDADIGARVNVGAGTITCNYDGANKFRTVIEDDVFVGSDTQFVAPVRVGRGVTVAAG

TTVWKDVAADMLVLNDKTQTAKSGYVRPVKKKS

>WP_004185818.1 MULTISPECIES: multidrug transporter subunit MdtD [Burkholderia]

MPPAPPAAAPSEKSLTVMLWLVATGFFMQTLDATIVNTALPSMAASLGESPLRMQSVVIAYSLTMAVMIP

VSGWLADTLGTRRVFFSAILIFTLGSLLCANAHTLPLLVAFRVIQGVGGAMLLPVGRLAVLRTFPAERYL

PALSFVAIPGLIGPLIGPTLGGWLVKIASWHWIFLINVPVGIAGCIATFYSMPDSRNPAAGRFDLKGYLL

LTIGMIAISLSLDGLADLGMQHAMVLVLLILSLACFVAYGLYAVRAPQPIFSLELFGIHTFSVGLLGNLF

ARIGSGAMPYLIPLLLQVSLGYGAFEAGLMMLPVAAAGMFSKRIITVLITRHGYRKVLLANTIMVGLMMA

SFALVSDAMPTWLKIAQLALFGGFNSMQFTAMNTLTLKDLGTGGASSGNSLFSLVQMLSMSLGVTVAGAL

LATFTGMMRSVTPTHTLPAFHATFVCVGLITAASAWIFAQLSPDVRGAVRRKTDPSERA

>WP_004199523.1 MULTISPECIES: two-component system response regulator OmpR [Burkholderia]

METKNPSKILVVDDDPRLRDLLRRYLGEQGFNVYVAENATAMNKLWVRERFDLLVLDLMLPGEDGLSICR

RLRGSNDRTPIIMLTAKGEDVDRIVGLEMGADDYLPKPFNPRELVARIHAVLRRQAPAELPGAPSETTEV

FEFGEFSLNLATRTLTKSGQEIPLTTGEFSVLKVFARHPRQPLSREKLMELARGREYEVFDRSLDVQISR

LRKLIEPDPGSPRFIQTVWGLGYVFIPDGAA

>WP_004544173.1 MULTISPECIES: hypothetical protein [pseudomallei group]

MRKTISMYWPLAIVVPLAAAAYLYLSEAAPRRAPAVARDTSAELARAVSYGFVDDASQPLPAASHVAPLG

DPKAL

>WP_004198518.1 MULTISPECIES: hypothetical protein [pseudomallei group]

MNTLNTLQPSAKVPTNERGGSAFADRQAAVAAVELLLPAFSAALQSDFVGDSGCLHIVVMDPALGPSLAS

FDDAILYEFSLPDPQYWDADYRAYARAKARLSWETGRDGHLLQANEPHRLRAGDTTLWGSVALDGIVVGV

SGAQPWFDEAFAGCIAHCLRAIAKHRAHATPDTLAL

>WP_165496393.1 aspartate aminotransferase family protein [Burkholderia pseudomallei]

MDLEEGIRRLYPYAEEFGVMRGFPEQGTPRDELLAQLRSMAEREDRNWESGHSSGAMYSGDRDHHAWLNE

AYSVFSHVNALRRDMCPSMNRMESEIVGMTVAMLHGDAVAAHHPGQRACGMITLGGTESILGATLAYREK

ARAERGIERSRMIWPASAHPVFRKAAHLFGFDVTVAPIDPVTMQVDADFVRDAVDANTVMLVGSACNYPY

GTIDPIGALSAIAVEKDVWLHVDGCLGGWMLPWGEALGYPDIPAFDFRLPGVTSISADTHKFGYGPKGGS

VLAWRDASFRRHQYFLMTDWVGGVYGSPGLTGSRSGGLIAATWAALRGLGREGYLARAKAIFETAFDMQA

AVRAIPELRVLGKPTFCFAFTSDAFDIYHVNDFMRQRGWRLNGLRRPDALQMCVTGPQTQPGVAEQFRCD

LGAAADYARAHAQERPKTSGVYASDAAGVDLSDDARTRAFFTQVIDLFTDCPL

>pdb|6NMO|A Chain A, 2-C-methyl-D-erythritol 2,4-cyclodiphosphate synthase

SMDFRIGQGYDVHQLVPGRPLIIGGVTIPYERGLLGHSDADVLLHAITDALFGAAALGDIGRHFSDTDPR

FKGADSRALLRECASRVAQAGFAIRNVDSTIIAQAPKLAPHIDAMRANIAADLDLPLDRVNVKAKTNEKL

GYLGRGEGIEAQAAALVVREAAA

>WP_004553866.1 DUF1841 family protein [Burkholderia pseudomallei]

MFNPSRDDVRRFFIDTWRKQRSGEILTPLEAMAADWIVEHPEYHAELEDAGRSAAHDYTPDEGRTNPFLH

LSMHLAISEQLSIDQPPGIRAAHEKLAARCDSAHDAQHAIMECLGETIWEAQRTHTPPDSDAYLQRILRR

ASRG

>WP_004198199.1 flagellar transcriptional regulator FlhD [Burkholderia pseudomallei]

MSATSEMLAEINEVNLSYLLLAQRLLREDKAMGMFRMGISEELADVLVNLTLAQTVKLAASNQMLCRFRF

DDHALLSSLADKGRSSAVSHAHSAILMAGQPVESLR

>WP_004191307.1 ferredoxin--NADP reductase [Burkholderia pseudomallei]

MSANAHETVLSVHHWNDTLFSFKTTRAPGLRFKTGQFVMIGLEIDGRPLMRAYSVVSAHYDDHLEFYSIK

VPDGPLTSRLQHLRAGDKLLVAGKPTGSLIIDNLRPGKHLYLLSTGTGLAPFISVIRDPDYYGAFDKIVL

MHGVRWKSELGYFDHITTELPENAYFGDLVRDKLIYYPSVTRETFERQGRLTELIESGKLFDDVGLPPLD

PAVDRAMVCGGPSMLADLVEMLERRGFVEGTSHAPGDYVIERAFVEK

>WP_004532936.1 hypothetical protein [Burkholderia pseudomallei]

MITFGEPLIALFDGSAVRNIQVGAPCYPTSRRYAAIDHGGAPAGA

>WP_044362781.1 cupin domain-containing protein [Burkholderia pseudomallei]

MPKRPLPEPSGRVRERTRAPLSEAPTPLLGNLSPAQFMRRYWQKKPLLIRQAITGIAPPLSRDALFELAA

DYDVESRLVTHFRNRWQLEHGPFEPEHLPSVKRREWTLLVQGLDLHDDRARALLERFRFVPDARLDDLMI

SYATDGGGVGPHFDSYDVFLLQVHGKRRWRIGAQQDLSLQEGLPLKILANFEPTDEWVLEPGDMLYLPPH

IAHDGIALGECMTCSIGFRAPSAGELRAQFLYHLAERGGLRTGARDNARYRDPAQPAVDSPAMLPAAMVK

RVAATLAGIQWDEHDVGDFLGCYLSEPKSNVVFEPPTPRLGEAAFVTQASRRGVRLDRKAALLYNARSYF

INGDAHPLATAAKWLPELADTRRMEAKRFVTLSRDPAMTGLLHEWYCAGWIRVGDMG

>WP_038715256.1 succinyldiaminopimelate transaminase [Burkholderia pseudomallei]

MSPTPVNPRLDTLQPYPFEKLRALFAGVTPSTPLPPISFGIGEPKHATPAPIRDAAAAALDGLASYPATA

GTDALRDTVARWLERRYGLPAIDATTQVLAASGSREALFSLAQAVIDSSPRENGATQDGERPIVLCPNPF

YQIYEGAALLAGAAPYFVNSDPARNFAPDYSSVPADVWARTQLVYVCSPGNPTGAVLTLDDWRELFALSD

EHGFVIASDECYSEIYFDETKPPLGGLEAAHRLGRDFTRLVMLSSLSKRSNVPGMRSGFVAGDAALLKRF

LLYRTYHGAALSPVWQKASVAAWGDEAHVRENRALYAQKFATVTPMLAEVLDVRLPDAAFYLWANVARTG

LSDTEFARRLYADYNVTVLPGSYLARDAHGANPGRDFVRIALVAGTAECVEGAQRIVDFCRGLAR

>WP_004554096.1 helix-turn-helix domain-containing protein [Burkholderia pseudomallei]

MSEPQPSKSPETNAGEKPATAGGGMASVAAVGARLAELRRTKGWSVDDVSARLKVAAPKLQALEAGDTSQ

LPGTTFALGLVRSYAKMLGVDPEPFAQALRREKGVPEVDLSMPASAGTDLPRGRVSIPLGGASRGRPWLW

AVAAVMVALVAAAMWRTGGDSSNWLARFKSSGAGEAASAAHAQREGAASDLPASAAVSGTVMQAGALAAS

ATQSVGGASAGDTAAASVPAAGAAPVVASAAAALSASAPVAAAQPAQGAQQPAASAADGASAAVTAAPGQ

SVVALKVAQDCWFSVRDKNGKELFSALVRAGETRQVAGDAPLKVTIGNKAGLESVAFDGKPVDPAKYSTA

RGNVARFALP

>WP_004547401.1 S8 family peptidase [Burkholderia pseudomallei]

MNKKRSNPEQMRLGQVRAVAGILSMSVLVPLAGCGGGGDGGGSGTPSAAAQPTPAPAPAPAPAPAPSSGS

SQSTNSSTSTAACPVTQAASTAAGETLVTRTVSHEAPVDHLIVKLQRTAAASASGARIMAAANDAARLDS

VIQRVMSQWSAKSGAVRSYAQNIAPTNAVQVERTMSDGAALLALGQKMSADNAGALAQTFAADPDVAYAE

PDRRVFARTVATDPDYAQQWNYFDPAAGINLPDAWNVTNGLPSVVTAVLDTGYRPHPDIIANLLPGYDFI

SDINTGNNGHGRGPDATDPGDWVTQQELTDPSSPFYQCASAPSNSSWHGTQVAGIIGAAANNGIGIAGVS

WYGKILPVRVLGKCGGTTSDIADAMRWAAGIPVAGAPTNLTPAKVINLSLGGTGPCGDTFQQAINDVIAR

GTTVVVSAGNDGQATTLDRPANCKGVISVGATDSTGQRAWYSNFGSDITLSAPGSNILSTSNAGTTVPTT

DAYGTHSGTSLAAPQVAGVASLMLAVNPNLTPAQIAQKLASTARPSPATASCLARAPGAGIVDAGTVVAS

ATK

>WP_004531198.1 hypothetical protein [Burkholderia pseudomallei]

MNTPLLMGFGALALFGAIGIAASRELLRAIDAQQRRAPVPVRMRTRRPHRDGRRTVL

>WP_004525851.1 DNA-protecting protein DprA [Burkholderia pseudomallei]

MIGQSPWARARGIRSISDDENFITALRSPQAALFEEFIMSPRALTRAELSGWLRLAGASGVPAAACAALL

GAFASLEALFAASHAELAALVGDAAAQSVLAPPAADFEQRVDAALAWLDEPGNALVTRHDPAYPGPLAEL

YDPPPLLYIKGRVALLHARAVAIVGSRGATPQGLADATRFARELSDAGLAIVSGLARGIDGAAHRGGLDG

ASGTVAVIGTGADLVYPACHHALAHEIAERGALVSEWPLGTPARSAHFPQRNRLIAALSGGVLIVEAALR

SGSLITARLANEMGRDVFAIPGSIHAPLSQGCHALIRDGAKLVETAADVLDEFGLDPARPVNSKRGAPAA

ASADADLDDDTRRLLDAIGYGPVPLELLAQRTSLPSGTLHRLLLQLELAGRVAALPGGRYTRIDGAQAEP

AQARGTSAVLHSGA

>WP_044363455.1 MULTISPECIES: type III secretion inner membrane ring lipoprotein SctJ [Burkholderia]

MIFGRVPLHRLVVRLLGVLALSVLLAGCKKELYTGLSEQDVNEMMVALLENGVDASKDSADGGKSWKLNV

DGDQLVHAMEVLRTRGLPRSKFDDLGNLFKKDGLVSTPTEERIRFIYGMSQELSSTLSKIDGVLVARVQI

VLPNNDPLAQTAKPSSAAVFIKYRPSADITALIPQIKTLVMHSVEGLTYDQVSVTAVAADAVDLARSRPA

AVIMPPWLVGLLAFGAMSIAAAGLFVVSRRPWTRAGAHEAGSAAPWRKRVAELAAHLRRRQRAN

>WP_004539494.1 type III secretion system protein SctP [Burkholderia pseudomallei]

MVSVIVERQSHFTRIVGMLAREIGAFCSDGAIVCAGNWEAQLTLDPELLPRTTLHVALSRFIIRIRFDVP

DGETRDLLLTHRSLLERELDEVMRCWGEARDIQLSVW

>WP_004530538.1 MULTISPECIES: type III secretion system outer membrane ring subunit SctC [Burkholderia]

MKAKALLCAAWCVAIFGTIGIGAANAMPVRWRSTVVHVVLEGKDLKDVLRDFAASQGVATSIAGNVQGTV

TGRFDLSPQRFLDTLAATFGFVWFYDGNILSITNANDMTRQVLPLDHASIGELRSALRQIGMDDKRFPIF

YDEVSGTVLVSGPAQYVQIVTDIAQRLDTLSGRRNGSTIRIFKLKHAWAADRNVQIDGNTVTMPGIATVL

ANLYRVRGRAGTGQSSAAPGVQRVQPMGDVSGSAYGGNRLMPPLPPSMMGGGRGDAFAKGASIDGESGGA

ASAAPGGAPRTEEASAERDELPIIQPDPMTNSVLIRDLPDRLAQYAPLIQQLDIRPRLIEIQAHIMEIDN

DLLREIGVDWRAHNSHGDIQTGTGNTAQNDYSGGQINPFFGTTTLAGNAVVNAAPAGVSVTAVVGDAARY

LMARVSALQSTSKVKIDASPLVATLDNSEAVMDNTTRFFVKVSGYASAELYSVSTGVSLRVLPMIVQDGS

ETRIKMNVHVTDGQLTGDQVDNLPVITSSEINTQAFVGQGQSLLIAGYSTDKRANGVAGVPWLSKIPLLG

ALFRYHSDSQNHMERVFLLSPRIIDPGT

>WP_004528711.1 MULTISPECIES: type III secretion system export apparatus subunit SctV [Burkholderia]

MFKSLKLPAGGEIGIVALVIAIISLMILPLPPVAIDLLLGINITISVTLLMVTMYVPDIAALSAFPSLLL

FTTLFRLSLNIASTKSILLHAEAGNIIESFGQLVVGGNLVVGLVVFAIITTVQFIVIAKGSERVAEVGAR

FTLDALPGKQMSIDADLRSGLLSTEEARKKRATLAVESQLHGGMDGAMKFVKGDAIAGLIITMINIVAGI

AVGVAYHGMSAGDAANRFSVLSVGDAMVSQIPSLLLSVAAGVMITRVTDERAPKRRSLGDEISHQLGSSA

RALYFAAFLLLGFAAVPGFPAALFVLLAAALAFAGYRLSRKGSPSSGAARGQEQEALRAMQRSGAKADVP

PILPRAPQFACAVGVRIAPDLASGLALPRLDEALDLERARLQDELGLPFPGVTMWIHPTLSAATFEVLIH

DVPHLSVTLPARKAMLPQQRHLSPAHAALTDEQKARHRDLIERSEAGPPIDASGQATHWIDEPAVTAKDS

VWRAEQIIAYASVAAIRTHAPLFVGIQEVQWILDQLGADSPGLVAEVLKILPSTRIADVLRRLLEEQISI

RNVRTIMESLVAWGAKEKDMLMLTEYVRGDLSRFLAHRAAQGERVLSAVLFDLQAEQHIRQAIKQTPTGN

FLALPPDDASRLVDRIQSLVGANARTDIALVTSMDIRRYVRRMIERRIDWLAVYSYQELGEHVELRPVGR

VSLQG

>WP_004528707.1 MULTISPECIES: type III secretion system ATPase SctN [Burkholderia]

MSADEPLRTAEFSHLADAIEREILAVSGVVRTGRVLEVIGTLIKVSGLDVALGELCELRTREGRLLQRAE

VVGFTRDVALLSPFSQLEQISRTTQVIGLGRPLSIPVGDALLGRVIDGLGEPLDGGPPLASETLQPLIAA

PPEPMSRRMIDAAMPTGVRVVDAMMALGEGQRMGIFAPAGVGKSTLLGMFARGAACDVNVIALIGERGRE

VREFIELILGQAGMARSVVVCATSDRSSMERAKAAYAATAIAEYFRDRGRRVLLMMDSLTRFARAGREIG

LAAGEPPARRGFPPSVFAELPRLLERAGMGRTGSITALYTVLAEDESGSDPVAEEVRGILDGHMILSREV

AAKNQYPAIDVLGSLSRVMPLVVDDGHMRAAARVRELLAKHREVEVLLQIGEYKAGANPLADEAIAKADA

IRSFFSQRTDDYAAAEDTIARLYALSGAN

>WP_011325916.1 Flp pilus assembly complex ATPase component TadA [Burkholderia pseudomallei]

MSATPSNPSFPAVRSAASALPRPRAPELADDAPAVGIVADMLRAAHERDASDIHVEPGESGWRVRLRIDG

VLHEFARPPAHLRDACVTRIKVLARMDIAERRVPQDGRLRLPLAPGRAGDYRVSSLPTLHGEKLVLRRLE

TLPADLSLAALGFDAAQARGVEAAIGAPHGLILVTGPTGSGKTLSLYCFLQMLNDVSRNVCTVEDPAEIE

LDGINQVGVREKAGLTFAVALRAFLRQDPDVIMVGEIRDAQTADVALKAAQTGHLVLSTLHTNDAPAAVA

RLLDIGVAPYNLAAALRLVTAQRLVRRLCPACRAPSDAPPATLRAAGFDAASIDAGWRPYAANGCAACHG

IGYRGRIGIHQVMPLSAELRNLTVARASSAELARQARAEGMASLRDAALARVRDGTTSLSEALATTEAA

>WP_011205023.1 sigma 54-interacting transcriptional regulator [Burkholderia pseudomallei]

MAELAANAAAGRAAGAPCMNQRDAIPIVPISPPDVGVLVSYLEQDPQPMIVLDPSYRILAANAAYQRQFG

IAGEAHVGRRCYQVSHHYDMPCDQAGEHCPMKQAGESRGLNRVLHIHHTPRGPEHVDVELRPIFDARGAV

IAYVERLTTVRSASAKPSGEGLVGGSDAFNAALSALQRVAPSMLPVLLLGESGTGKELFARALHEASARA

MGPFVVVDCSGIAETLFESELFGYEKGAFTGASARKPGLVETAQGGTLFLDEIGDVPLSMQVKLLRLIES

GTFRRVGGVEALCADFRLVAATHKPLKAMIGDGRFRPDLYYRISAYPISLPAVRERPGDMPLLVDSILRR

IAALGPVAGQHFVVAPDALARLEAYAWPGNIRELRNVLDRACLLTDDGVIRVEHLPDEVAGGARIEPGAP

AKLSDDELARIARAFDGTRRALAERVGMSERTLYRRLRALGIATREA

>WP_004524346.1 MULTISPECIES: type III secretion system export apparatus subunit SctS [Burkholderia]

MEIDSLIRFCTQAMLLCLSVSLPPVIVAALVGLGVSFVQAITSLQDQTLPQVLKLIAVTITIMVAAPTGC

AAILHFANQMMQLAVPQ

>WP_004524341.1 MULTISPECIES: type III secretion system export apparatus subunit SctU [Burkholderia]

MSDEKTEEPTDKKLRDARRDGEVSRSTDLSDAVSMSAAILLLVAAADHFGDAMRALVNGALAFVSADHSL

VEMTARLYQFGGIALSAVMPLLFVAALAGIGGSVLQVGLQISLKPVMPNLGALNPAEGLKKLFSPRSAIE

SIKMIVKAVIVFCVAWKTIVWLFPLIAGALYQSPPELSRIFREILAKWLMVVAGLCLLMGAADVKLQRFM

FMQKMKMTKDEVKRESKNDEGDPLLKGERKRLARELAAAPPQHQVAHANFVVVNPTHYAVAVRYAPDEHP

LPRVIAKGLDEAAIALRRAAQDANIPIIGNPPVARALFRIGVEEPVPEELFEIVAAILRWIDAIGPRRNE

RA

>WP_004523454.1 LUD domain-containing protein [Burkholderia pseudomallei]

MSARDDILGRIRAALGSERARLAAQFPAPAQAKGAGASAAPNANAAFAPDASLSTASSAARGSAISLGDV

DLVARFVAKAQQVSATCTHIATAAGAPAAVDAYLRDAGLADAPLVVAAALDALPWRAHRALPGVDLRRDG

LVSVTPSFAAIAETGSVVCLSSSATPTSLNFVAATHVVLVNRSAIVATMEDAWARVRATIATLPRAVNVI

TGPSRTADVEQTVQVGVHGPKRVLVLICDDA

>WP_162492307.1 hypothetical protein [Burkholderia pseudomallei]

MSSRVVACRRVSSRVVACRRVSSRVVACRRVSSRVVACRRVSSRSSRSSRSSRSSRVVAGRRGFAFGARA

ARRRRTRSSGAPTVEAIVFQKDSMKKILAVIAFLAVVGWLAATTTVLHAPSAQPCTDAWFDAIDKQFDIT

DNAGHGPDPGSGEWLGVVERKAKLPESGQLTEQQRCEAIQRELSQRTYLVNRRLGLKLAL

>WP_162486944.1 betaine/proline/choline family ABC transporter ATP-binding protein [Burkholderia pseudomallei]

MEMSAGLFFVRARHERQRVAAAGRRRDAAVVSALPSRVRSRCGVARSGRRIEAAARTGLRANGAGIRHLA

FGSRVFGGARPTVVYPAKPSRAGSPRCGPAFQSRRETAADAEAKRNRSVVSAPSGPHRYPSGMHVEPLGT

RSTAETAPRLRSRRRSDRTGRASPAIAAIAGECVCGNPTCANRCLGTASGEKRQVSRDQDSTVERADHAR

GRAHAVRATASRAASDAASRKRGMNASARSAGAHRGTPRPPRPRAARCGASRRPPVGFVSAPKRPFAALV

RTCRICASVCRNSSAARGFFWQPCRHAMTAYFPAQRGDVAMDAPKVVVEGLCKVFGSNPRQALDMLAAGA

TKDEVFARTGQVVGVHNVSFDVREGEIFVLMGLSGSGKSTLIRLVNRLVEPSAGKVMIDGRDVAAVRRAE

LTALRRTDMSMVFQSFALMPQRTVLSNAAFGLEVAGMGRKDRERRAMDVLEQVGLAQFAHKLPAELSGGM

QQRVGLARALAVNPSLMIMDEAFSALDPLKRKEMQNVLLQLQKEQRRTIMFVSHDLEEALRIGSRIAIME

GGRLVQVGTPQEIIANPADDYVRAFFEGIDTSRYLTAGDLMLTGAVPTLSKLDAKHVAASLNGSAEYAFV

LDEARKIRGFVTRDALNGATPNVRQVESIPRDASLDHVVERCVAHPHALPVVDDDGCYCGSVDRAVLLKA

ITRSRGSHV

>WP_162481305.1 catalase HPII [Burkholderia pseudomallei]

MTLPTDLPQRLVEIEHLLSDCFASPSTTVLHVDDSSGRLTIQVSWVAARAASSLLEARCALNLVFEPNVL

RRYGRLDAAARARVRERLAALARQTVDAARPVEGGEADACNATLPVTDALLAAAPRRERLARTRRARAHS

RCGRRARRPRHERDSSPMADSDRNAPHETPRAAQPDDARADRKSQDLEPYRSKPQGEALRTNQGVRIADD

QNTLRAGARGPSLLEDFIMREKITHFDHERIPERVVHARGSAAHGVFRVYESMAEYTKAAFLQDPAKETP

VYVRFSTVQGPRGSADTVRDVRGFAVKFYTDEGNYDLVGNNMPVFFIQDAIKFPDFVHAVKPEAPNEMPT

GASAHDTFWDFVSLVPESAHMVAWLMSDRAIPRSFRTMEGFGVHTFRFVNAQGVARFVKLHWRPVLGSYS

LLWDEAQKIAGHDPDFHRRDLWEAIERGDYPEYELGVQIIEARDQHAFGFDLLDPTKLIPEETVPVKLIG

KMTLNRNPDNFFAETEQVAFHPGHLVPGIDFTDDPLLQGRLFSYTDTQLSRLGGPNFHELPINRPVCPFA

NLQRDAMHRQTIDVGQASYEPNSLNGGWPKETDPAGRDGGFDSYPEAVEGAKVRVRSGSFADHFSQAALF

YQSMSDVERRHIRDAYRFELGKVTRPEIRARVVDEILARFDAGLAAEVAEGLGLPPPPASAPAIVAQPLS

PALSLLTRGKRSVRSRKVALVATPGADTRLIERVRRALTDARAVPVLVAPTLARIGELTPEATLAGMPSV

MFDAVFVCGGDGDGRDLVHSSDARHFLQEAFKHLKAIAAVGSGRQLLGAAHLPEQGDGVCVGHAADLDQV

VAKFFDALSEHRVWSREPLAQGVPA

>WP_162481303.1 zinc-binding dehydrogenase [Burkholderia pseudomallei]

MRGLTSRVRRRRHFRAPPAKLEKSRRCDCIVADRGSRIADRGSRIADRGSRIADRGSRLAPRASRLAPRA

SRLAPRASRLAPRASRLAPRASRECDRGALAAFADAGACRPFPFLPKVMPMSTPVNRQLRLKARPDGRVG

HEHFTLAEAPLPALGPGEMLVRVLYLSMDPTNRVWMSDIPQYLPPVAIGDVMRALGIGRVVASNAPGFAE

GDLVQGLVGWQDYAHVRADEIAQYTKLPAALGLPLPRLLGACGMSGLTAYYGLTEIAPVQPGETLVVSAA

AGSVGSVAGQIGKIHGARVVGIAGGADKCRYLTDELGFDAAVDYKSDDWKRALKDATPDGVHVSFENVGG

EIMRAVLSRMAIGGRVALCGVIANYNNGRPADDVSVLIAKRLTMRGFLILDYRKSREAIATLAGWLRDGR

LKAEETVADGLTNAPDVLNRLFDGSHRGKLVLRVDPQA

>WP_004527905.1 MULTISPECIES: protein-methionine-sulfoxide reductase heme-binding subunit MsrQ [pseudomallei group]

MENDMQSSTFAGSASPGAGARGRAPRRDAGRAGGAPGWLAPVKIAAFVAALYPLARIVFFGVTDRLGANP

IEFVTRSTGLWTLVFLCITLAVTPLRRLTGINALLRLRRMLGLYAFFYAVLHFTTYIWFDKWFDVVEIAK

DVVKRPFITVGFAAFVLLIPLAATSPRAIARRLGRHWQTLHKAIYAIAAFGILHFWWMRAGKHDLALPKL

YGAIVLVLLGWRVLVWARQRWGAAAAGGRAS

>WP_004202378.1 MULTISPECIES: hypothetical protein [pseudomallei group]

MQRDRHTRWLLTSGDIRKDAPFFGTLGVLIGVAQLVGFRCFHKADWGTKLLFEHIVFDTLIVALIVVWLA

RIPTEWLRIERKREFPMLDKLIAHVARRAASFAVTAASVVAGVAAVAALSGSPVHAVKFAFFCAYLLSIG

EAVLNPLIAPGQSRLNGVAKALLIGMPLGFQLTG

>WP_004195733.1 MULTISPECIES: sigma-54-dependent Fis family transcriptional regulator [pseudomallei group]

MDKKEKVETNASISGGWVRLPADYGDVLRRAAESLFKTFEHSSVGTLIVDKDARVVWINQRYAARFGFAD

PQQAIGRDCEAVIPHSLMREVVATGRPILLDIMETGREPLVVTRLPLTDDAGETVGAIGFALFDELKTLT

PLFSRYMQVQQELIATQRSLAQARRAKYTFASFVGTSAVSLETKRQGRRAAQVDSPVLLLGETGTGKELL

AHAIHAASARALKPLVTVNVAAIPDALLETEFFGAAPGAYTGADRKGRVGKFELADGGTLFLDEIGDMPV

PLQGKLLRVLQDKEFEPVGSNRIVRANVRIIAATSAELPALVAEGRFRADLYYRLNVLTIHAPPLRERAS

DIEALVYTMLEELAAQHGLAEHCELTDDALRLLCAYPWPGNVRELRNTLERALMLSDRALIDARALAPFI

GPARGAGGGVGAGGGAGAGGVGPAAVAIAAQTAMADTRAAASSYADAFAAWERQFLIDALAASNGKVTEA

AARIGIGRATFYKKLATLGIDT

>WP_004194484.1 MULTISPECIES: replication initiation protein [pseudomallei group]

MATKRAKKTDVDVVSASSAELRKAVEAIAIQPKNGKITLLTRKLFNVLLAVAQQADDSGDTYRALLSDIV

ANSAFDSNDTALVKEHLRRMVSVQVEWSTGTSSQKPGRKWGISTLIADAEILEDPATRRVWVEFSFAPKI

KKKLLDPVQYARLSLQFQSQLRSSAGLALYEICVRYLTNPSHLTMREPWEWWRPILSGTPDTEAGDEAKR

EYKYFKRDYLRPAIAEVNAVTNIFVELVEHREGRRVAEIQFRVSERKQPMLALDEHPNVFDSTLVDRMVK

IGIPLKEAQTLYADNEENRIRAALQLTEQRVRSTTLPPVRSAPALFKDALKKGYAPPVDTVEAPPLGGSA

KLAAAAANVGQADDPKARLRSEYDAYRRKEAKQLYEEQGDAEREVARASFESDVLPALGSHLRDDWRRRG

LDSKLVETAFFDWLAQRTWGEPTDGDLLAFTLSQSRAA

>WP_004194359.1 MULTISPECIES: 4-hydroxybenzoate octaprenyltransferase [pseudomallei group]

MLARFPLYLRLIRMDKPIGSLLLLWPTLNALWIASDGHPAPSLVVIFALGTLLMRSAGCAINDYADRDFD

RHVKRTAERPLTSGKIRAWEAIAIAVGLALVSFLLILPLNGLTKELSVVAVFVAATYPFMKRFFAIPQAY

LGIAFGFGIPMAFAAVQDTVPMIAWAMLAANVFWSVAYDTAYAMVDRDDDLKIGMRTSAITFGRHDVLAI

MLCYAAMLGIYVWLGAALHFGWPYWAGWAAAAGCSIYHYTLIKDRERMACFAAFRHNNWLGGVLFAGIAA

HYALAVR

>WP_004194228.1 MULTISPECIES: mechanosensitive ion channel family protein [pseudomallei group]

MDLASVQTFIVTRGLDFGLQVIASIALWIFGRWAIRIVTNLMGKLIRRSGKVDSTLSHYLESVVSVLLTV

LLILAILQVFGVQTTSFAALLAGLGLAVGTAWGGLLAHFAAGVFMQVLRPFKVGDVISAGGVTGTVKELG

LFGTTIVTADNVVTLVGNNKIFSDNIANYSATPTRRVDLTAKIANGVDAADAIKRLKAALELIPNVVKHP

APDVGVLSFTPEGPLLFVRPSTQPANYWQVYCDTNRAILDTFREAGYPTPETPVAHRTTADVAKS

>WP_004190973.1 MULTISPECIES: glutamine ABC transporter ATP-binding protein GlnQ [pseudomallei group]

MSMVEFRNVSKSFGHVPVLKDIDLRIDAGEVVVVVGPSGSGKSTMLRCINALEKITGGDLLVDGQSVKGK

AAVIHGIRLEAGMVFQQFNLFPQMTALENVMFGPIQVRGASRAQARDQAMALLAKVGLEARANHYPSELS

GGQQQRVAIARALAIRPKLMLFDEPTSALDPELRHEVLKVMRDLANEGMTMIVVTHEIGFAKQVGTRLLF

MDQGGIAEDGDPKTLIDHPPTPRLKDFLKHVS

>WP_004189576.1 MULTISPECIES: heavy-metal-associated domain-containing protein [pseudomallei group]

MKLEVKDMSCGGCANAITQAIQTADSSARVSVDVASKVVDVGSALGIERVVAIIEAAGFHPAVLAA

>WP_004189276.1 MULTISPECIES: pilin [pseudomallei group]

MGIARGLRRVWRVSRRRLRARGFTLIELMIVLAIVGVVAAYAIPAYQDYLARSRVGEGLALAASARLAVA

ENAASGNGFSGGYVSPPATRNVDSIRVDDDSGQIVVAFTTRVAAAGANTLVLVPSAPDQADTPTARVALS

KGAVQAGAITWECFADGKASSSLPAPGAGPLPTDAPTLAGKLAPPECRA

>WP_004189150.1 MULTISPECIES: D-alanyl-D-alanine carboxypeptidase/D-alanyl-D-alanine-endopeptidase [pseudomallei group]

MNHHACRIAASPSPPPPAAVRARRTLARGAMLVAACAALAFAAPADARRKPKPPRYPAAVSAARNVLPAS

VLVALQRARVPASSLSVVVERIGDRTPAVAWNASRPMQPASTMKLVTTYAGLSLLGADYRWRTSAYADGD

VDENGTLHGTLYIKGTGDPKLVPEELIDLVNKIRRAGIVNVDGALVLDKTFFAPQTRDLPPLDDDASAPY

NVGPDPLLYAFKALSFTVTPTDSGAIAVDVVPALANLNVDNRLVEGQGSCDAARPTLVTDANGELTASFA

GDYPPSCGPTTTNLAVLNHSTFFARGFLALWRQTGGSFSGTIAEGKVPGRARPVASHHGPVLSSVVHDIN

KFSNNVMARNLFLTIGAVEHRPPATPAQSADTIRAFLAHSGLPTDGLALENGSGLSRDERVSALGLADML

QAANASPVAQAFVDSLPIAGVDGTMRNRLTNAPVGGNAHIKTGTLRDVRAIAGYVASADGSSYVVVSFIN

DDRASAARAAHDALLEWVYEGPR

>WP_004188758.1 MULTISPECIES: hypothetical protein [pseudomallei group]

MAKKQLGEGVRELLYQALETELGGAKVYEAALSVVTDEALREEWQKYRDETLHHQEVLRSVFDALGLDPD

EKTRGRAAVAGLGKALVEVIAYARGGGNPLVAQIVAAECVVLAETKDHLNWELIGLAAGELSGDAARVLK

DAHDEVEPDEDHHLYHTRGWTRELWMDSMDFKAALPPPEEVRKVDTAIGAARAEHARGRMAR

>WP_004188705.1 MULTISPECIES: membrane protein [pseudomallei group]

MSAARPENKENDMTHPDRKAIASTVAVFASQIGLIAAVRGLFVADGSAPYFVLFAIVAGVLGFVDLYYRP

IFAPAFARQRVAFVHRRRGAARDVS

>WP_051236681.1 SDR family NAD(P)-dependent oxidoreductase [Burkholderia pseudomallei]

MNGLKERVGSGYVAIPILVALRGARAPGAAGDRDAAWTERIAAAARLDADGAAALREWLNDAAPAAGADD

ARLACGDAIPTDILEPYRFEPDARAFFDDAFRECLNRWSRRLLEQDGATWLSDVVLVPFLVAAARARESA

PSCEARSIRALADAFGADLRRLLDASRVLDDGDIGDIGAMRASLECVTSFRDGLLHADRRLSNGAPAACR

PQVCAAAYEDLEAAVLRRFDASANARAGRRPRYLAFVGGEDRDLPERLVERIVRQGEPRNGGGTGVEPPR

IVIPRADAAPADDSAQPDPAAAFGVDAIDDVLPVCVLSGVDDAAAIRRLRALLVRGAHAAVVLACHGLPA

ASAALPDAWAAARPARMLRLLGFGAARDAQAFLVDMAADGLFARTPPIGYPRASRARRATLGEFEARDYR

VRPAAPHDLPALQALELACWPAALRMPEATLAARVGRHAAGQFVLELDAREAAAGPRRLAGVIYSQRIAS

VRALDGVDADTVDRLHEDGGPVIQLLAVNVDPACQSRRLGDQLLEFMLQRCAALADVESLVAVTLCRDFH

KHASMPIDDYLRLRNAFGFLADPILRFHELHGARIERPMPGYRPRDARNAGFGVLVSYDLARRARNEAGA

PAASDAPATPDGAPARADGGHAGVAASPRPERDAATAADPDALDAFIEAEIRRIVGGGAELAYARDLPLM

QLGLDSVGLLELAEALALRCGVALPATFLFQHNTPARIVAYFDASRHAPPRAGCDTGCETAGAAAAAARP

SSRGCPATEPGAAREPAQAAPFAPDGIAIVGIACRLPGGLDTPEAFWDALKAGACVVGELPGDRWTWPAD

IDPGARHRGIDRGGFLDDIRSFDAGLFRLSPKEVATMDPQQRILLELAWEAIERAGHCADAVAGSRTGVY

VGASGSDYRLLLERAGTGVDAHVATGASMAVIANRISYTYDLRGPSIQVDTACSSSLVALHQAVQALRAG

ECDQALVGGVNVICHPGNTIAYYKAGMLSPQGRCKTLDDAADGYVRSEGAVMLMLRRLEQAVADGDPIHA

VIRGSACNHGGLTGGLTVPHPDRQADLLRAAWAAARVSADDIGYLEMHGTGTRLGDPIEVRGLADAFGAR

DDAAARGTCGIGSVKSNLGHLEAAAGLAGVLKTVLALKHREVPATLHFSRLNAQISLARTPFAVVDTHRA

WPARGGARRLAGVSSFGSGGANAHVVLEEYPSEAPPRAAAGDALFVLSAHSREQLAEYARRVLAYCERRL

QSGDDAPAAAAVAHALQRRQAMAWRLAFVAASLEEAVRRLRAFAAGAAQPGTFVGGGAPKTSVADFVNQN

PDVQQVVSAWLRERQLAKLARYWADGVRIANWSALYDARPACVPLPCYPFARERHWIAARPAEASEATAA

AAPPAAPDDAYRAAPRLEAESRDAAGASFRVRLDGDASFLIDHRLRGRKILPGVVHFELAHAAWAALARA

DAPAIEFRDLAWTHPVDVATPERVLGVRLRRVAARPGAHAYEAYSPPDAAGGGERVHARGTVLDVAGPPE

PALDLDALRARFDGEPGAHERDAHDCHRAFERMGFGYGPAHRGLRGLRWRGGARGATEVLAHIVLPDCIA

DARERHRLPPGLLDAAVQAAMAAAIGRDALGASPACVPFSLDRLVCAGSCPARAWVWARRRDGARAALAP

VDLDVCDDRGRVWASFRALTFRPLREAAGRDVAAPRLFRPRWAARPLSGERASAGAADVAHWLVLCGFDE

GAALRRDLATLRARLPDTSIVAIDSDAATLESRFADSAGQLFELFRELALSGATPRAAVQVLVPADGPER

AFAALSALVRGARLEHPSWSVQLLSLERATQAADAAARALENRGDDADFVRYRRARRESLVFEALPAGRD

EPPRPWKEAGVYLITGGAGGLGLAFAKDIAAHVRRATIVLAGRAAAPDAALSASLGAIARPAGVDIRYRS

VDVGDAPAVAALVGDLLREHGRLSGVIHAAGVTRDALLVRKPRAEFDAVLRPKVAGAAALYAATQDIDLD

FLVSFSSIVGVTGNLGQTDYGAANAFLDALAGLRAQMGGERRARVLSIAWPLWRDGGMGREPEVAAHFER

AFALAPMDTAAGIDAFHRALASDAAYVVVANGDADWTPERAIARALSARARPGAPEPAERAHGPAREAAA

AACEAVAVSASASSLSPSPSPSSASASVSAECAPADDGAARRHAVVAYLTRQIGAVLGHAPDSLDIDAPF

TSYGMDSILALDTTRAIETDLGSLSKTLFFEHENVRQLSDYLLDEHADRLAACAWFAQAGEPARAAGPAL

AAGGEPTRETAAPSAAADTTADTTADTTVGIAIETSTETPTAATTQAGAAPEGRYRRVAKAALPADGQLA

AAVAAIGGAAATKGVALFEIWPELFVDSAGHGYCHLLVDGGVLFAAQHAGDARHRAALFAALLAYCDRHG

YAFGYLDLSEGRKPDLEAQCGLLAAPVGVVQIVEAIASFSLAGGRMRRLRYMVERFRKAGACRVVEYRAP

DPDVAREIRRVICAWSDAKKVVNNVDIVLGEMASGSLHERYRVFLTYLDDVLQNVIMIARDGDGYLMDQE

YYVADMPLGGTEYAVTEILAALAAEGRERFSLGLTWGLFDTGEGSSDPAADAFLASTQTQLRRIFERGAA

NRQYKSKYGTRDHAVYLYRRPGKPEPAIVGCLSQFYRKGLTHHEVRRLAGLADAPVPVAAPATVPQVTPA

PAPVATPTADERAYDVTRIDAATIRVDLVSDSWAHVDYPFMRARAATLDRHAPPARGGDPGRAVAELLGF

AQCLLTTSGRAAEHLFFRARRSARTRVPQNLLFESTLHNLVKSGFEPVELPDARALDPDSRDLFRGGVDL

AALDRELQAHADATAMVMLELCNNASGGYPVALAQIRAIAAACRRHGVPLVMDVTRIVKNAELIRRGEAG

YAQRGLWEIVREIADHADAVVGSLCKDFGLGAGGLLAARDARVVANAAGIARLEGGLPGPAELRRIAAAF

DDRAYLEREIGRQLDFARDLHIELERCAVPVVQPGAGHCVLVRVDQLAPPGGSAPSRGAYLRLLAERYGV

RGGLHLVGNLRDSHLNACVRLALPLGFDDPRGPGALAAALAAARDGRDHALDDLMRAPRARAAHGGRCAD

GIAIIGLSGRYPDAPTLDAFWRNLVSGRRSISEIPAERWDWRDHYERDPDTAVAHGKSYGKWGGFLDGFS

AFDPLFFQIAPREAEFIDPQERLFLEACWHALEDAGCPPSALTRAQRAKAGVFGGMTKQGFNLYGAGGAQ

PYQSTSLAALVNRVSHCFDFNGPAVAFDSHCASALVAIHEACQYLRREPEGIAIAGAVNLNLHPSNYQQL

SKMQVLASGAESASFASGGLGYVPGEGVGAVVLKDYRRALEDGDPIYGVIRGSAVNQNGRMNRFGMPSQK

QQEAVVRAALAQAGVDPRSITYVEASAHGSAVGDAIEMAALTRVFGARERADGRYRIGSVKPNIGHGEAV

SGMSQLTKVLLSLRHGQLPPTLVCGAPNPDIDFDALPFELNTSLTDWARARVDSERVPRRAGITSTGASG

LNAHLVLEEHAAPAVPAQAGPGEADARAHVFVLSARDRARLDDYARDWIAFLNDDPQRDLAAIAYTLQVG

REPMACRLAVVAADCRDLAGKLARWREAAHADCDDVFHGEARAAAGKPHREAARDAREPRDVARAWVGGA

VVDWAARHAGARPARVAGLPGYPFERRSYWPGAAAALAAAPATARATAASDASEAARALEAREAHEATRA

TRMTRAARESEARQAPEAPSMSEATEATEVAEVAEVAEVAEARDARPGTVADDAAARLEAAFLPRFIELV

ADVFRLPAGELDADRPLDEYGINSFLIKVLNVRFADIVGRVSSTLPFEYRTAGEMARHFLTAHRDACAAW

VAFDGAASPGAADASSAPPVPAASAPAASAASAASATPATQATQATQATQATGPTGEPSRASAGVPSGAS

SSIKRPGATWDEPIAIVGVSGRYPQARDLDAFWDNLMRGRDSITEIPPERWPLDGFYDEDRERAIGASRS

YAKWGGFIDGFAEFDPQFFNLSPREASNMDPQERIFLQACWEALEDAAYTRARIAREHGGRLGVFAGITR

AEFCLYGAGNLKQGKAPFTSFCSLVNRVSYFLDANGPSIPIDTMCSSSLVAVHEACDKLRLGECEVALAG

GVNLSLHPYMYVSLSAQRMLSSDGRCKSFGLGGNGYVPGEGVGVIVLKPLSRALADGDRIHATIRATSIN

HGGKTNGYTVPNPIAQQNVIRSALDRAGVHARAVSYVEAHGTGTELGDPIEIAGLSGAFRRDTSDRGFCA

IGSVKSNIGHLEAASGLAGLTKVLLQMKHGLLVPSLHASELNPNIDFPASPFVVNRETRAWERPVIDGRE

HPRIAGVSSFGAGGTNAHVILEEPPRQASPARAPTPAGAPALIVLSAKKPEQLRRYASELLARLRDADYR

ARVDADGLRSLAYTLQVGREAMDERLAVIADSVQALEGKLRQFVDGKTDIQDLHVSRVGRSAHHVI

>WP_038775967.1 SDR family NAD(P)-dependent oxidoreductase [Burkholderia pseudomallei]

MSFDQTLIADLLDQWLGGQSPDWDRLYPAGRPPIEHAPTYPFARNVYWVHAADRTPEPGLAAAPPRSRDA

APPLEAGTMPAAAIAVVGATAAATATTAVTARAATTVTNAIDATDATNATNATNATAATTAAAATTAVTA

SGATGASAAIVLKSAADEARRHFTPSRERAPVVLRALAPAAADVADVTDVAASRAAQTQVFDGAAVEAAR

LAPDAIGAALIDSLARRLLVSPQSIGARDAFDALGVDSLIGQEWLRELNRTYGTSIDGATLAECGHIGAL

ASRIAGARADAAAPAWVPRAEPPQRAAQCAPDAGTVSASVLASISTAASTLASASASASAAAAAATTHAG

DRSAPRITRDALVHALAASLAKALYMDVADIDIEQPFMEMGLDSIVGVEWVHQVNRAYGIGINAIQVYDY

PNIVTFAGLVESLTNAGGAAGGDVASGVGGSDSNADTDRDRDRDRDGDGDVDVDVDVDVNSDGVLTRPTG

AAEPARAAAAATSPATAEIAAATAAAASSAIAAPPTTAETMAAASAACAPRGELRREVIDSLVAALYMDA

SEIDVLQPFVEMGLDSIIGVEWIHALNRRYGTSIEAIQVYDYPNVEKLTELLSKSIDALSPPHARCESDS

DVLPGEPMTAATAANDTNDTNDTNDTNNANNANNANNANNANNANNANKCGVVAAEAAFAAPPAAPAAAC

AAAAIAAPIAPIAPAPLAAPAAKPATLLDELVASLAQALFRPADTIDAERGFEAAGLDPIVADEWLARVH

RRYGVRVRADEALACLRIADFAALVAARQEAGGAQAAGAAPVAAHDARCAARASADARAGDREPIAIVGM

SGRYPGARDLDAYWENLAAGRSAIGEIPASRWDVARHFDAHPATPGKVYSKWIGLLDDVDCFDPAFFRIS

PAEAQEMDPQHRLFLQEGYRAFENAGYSADTLDGRNCGVYLGIMNQEYRQLGAGGAVTMLEKSNSFAIGA

ARLAYHLNLKGPAIPVDTACSSALVAIHLACQALRAGEIDMALAGGVTLYLSPDAYIEMCSSGMLSPDGR

CKVFDDSADGFVPGEGVGAVVLKRLGDAQRDGDPIIATIIGSGINQDGKTNGITAPNMASQFELVSGVHG

RYGIDPATIRYVEAHGTGTKLGDPIELTALGDAFRVRTAQTGFCALGSVKSNIGHTSAAAGVAGLHKVLL

CMRHRTLVPTLHFAVPNRHFDFAASPFYVNTERAPWAPLAASPRRAAVSSFGFSGTNAHLVVEEYVHPAA

AAPEAGGPFLFPLSARTREQLAAYAAQLRDHVRRAAHEDAGLADLAYTLQVARKPMAERVGLIARTKHEL

AALLDAFVDGRDGGDGLIAGRRDRAGGTPPAPSPEALRALVDAGESRTILQRWALGATIDWACLYRDLDA

AARPRRIAAPSYPFARERYWLPDPGTRRTPAARAARAAGPHPLLREIDSAQSAACFGATLAGDEPFLRDH

RIDGRPVMPSSAYLEMVREAAARALGEPADAMLVIEGIAWRNPLTVSGGARRLQLRAQAEPGARALRFDV

SSQAAGDAAAEPLAHCDGVARYVPRAPAGAPDLAALRARLASGPRAADEAACAALHARFARLGIEYGATH

RVLLRLRVDGDEALAELAPAADGIAQHELHPGTLDAALQPMLALLGERVGDGVPVVPYRIERAEIHAPTH

GARWAWLRMRPDAHEWIFDVDLCDARGALCVALRGIAVTAWRRPDEVVRLEPVWRAAPVEADLHDERAGL

DAQRVVFVCGAHGAPRAWPADGIAPVRYAALAAGAPPGEPDALAGWFEAHALALFDEVRRLLAPGMRQAT

LVQIVVPAAGPGAILHALGALLQTAHLENPLLHGQVIAIDDIDAPDLPRRLARDARRAADTRIRYVNGER

QVACFDEAAAPDGARALPWRQGGVYLVTGAAGGLARALADAIARGMGGDAPRATLVLTGRSPARDDMRAL

VASLCALGVAADYRVLDVADRDAVARMVEAIVGEFGALHGVVHCAGVLRDNYLLRKSADEFAQVLAPKVR

GTVNLDFATRDVRSLDFFVTFSSGAGVVGNPGQADYAVANAFMDAFAAHRASLGAARPGVSVSIAWPIWQ

AGGMRIDRQTEAELERRLAMRPMPTALGLDALHACLLGASPCPTVIHGARARILALARQGFAAPPAAPPG

FGAADRGADAPGADADADAVKARVRAAIDGALSAVLKLPDARLREPEYFESYGIDSINAIRLTVELERTF

GPLPKTLFFEYRHADELERYLVSVHGAAVGARIRSAARGAPAGPACARAGESGEPPGAPTRPPASEGGAA

GAPRASAAPPLAERDIAVIGMAGRYPQADDLQQYWDNLRDGRDCIEEIPPHRWDWRKHYDPARGHGAHHS

KWGGFINDVDAFDPLFFNISPKEAVSMDPKERLFLEQVWTAMEDAGLRPEDLRRDAQRGTGVYVGLMYEN

YQLLAAEAAAAGSDVGMAGGSYASIANRVSFFLDLRGPSLAVDTMCSSSMVAVHLACRDLLAGEIGVAIA

GGVNLSLHPNKYRMLSAARFMSGDGRCASFGSGGEGYVPGEGVGVLLLKRRADAERDGDRILGLIKASAI

NHGGRSNGYTVPNAAAQGGVIANAIRAAGIDARTINYVEAHGTGTALGDPIELAGLARGFADSGANGPCR

IGSVKSNIGHCEGAAGIAALTKVLLQLAHRQIVPSLHSRELNPDLPLDGSRWIVNQSLCDWERVVVDGVP

LPRTAGVSAFGAGGTNAHLILSEYPADACAAPAGVIEPAGRDAHDMHDTQDMQDMQDMQDMQDALVVPLS

ARNAQRLHAYAQRLRAFVAAHARGERGAPPRLVDLAFTYQRGRIAMPERLAIVARSLAELERALTAYVAG

QRTGDGIYAGRADRAAAGDARGAASERTAADFADRLAARWVAGEAVDWHALFDGRAPRRIAAPTYPFERG

RYWIGASRAAAGAAEAAMRAGGGASRATTAPRALSESGGEVRQPDQTIRRVRLAPTSSFTATARAPSPTQ

ATQATQATQATQATQATQATQATQATQATQATQATQATQATQAAQAAQAAQAAQAAQATRATRAAAAFAP

ATKASLEAALRDSLASALFVGVDEIDPSRPFSELGLDSIVGVEWIRDVNRRYGVSIRTTDVYDYPSVGEF

AGLLERLLRESSVAGAPPAPATEPTMEPTMEPVTAPRPEAAGAAPCEPPPDEGRGGAPGNVERIRRELMR

SLADALFVDIAEIDVDRPFAQIGMDSIVGVEWIKGINQRYRVALKATDVYDHPTIASIAALVDARGASAA

STASTASTASTASTASTASTASTASTASTASTASTARPEADVRLAADARIASAASGAPESEPARAAHPGA

AASRARADVAPDAGPAPRRLDAAAPDARAARAEPVPERIAIVGMSGRYPGAPDLDAFWDNLAAGRDAIAE

IPPSRWPVGAFYDPEPGKPGKVYCTRIGLLDDVDRFDPDFFRISPAEAEEMDPQHRLFLQEGYRAIEQMG

CAPASLSRRKCGVYLGVMNHEYGELAMRHRGAASGIGSSYAIGAARLAYYLNLKGPAIPVDTACSSALVA

THLACQALRNGEIDLALVGGVTVYLTPESYVAMCAAGMLSPEGRCKTFDDAADGFVPGEGVGALVLKRLA

DAERDRDPILGVIVGSGLNQDGRTNGITAPSGSSQTELLRDVYRRHRIDPAGIGYVEAHGTGTKLGDPIE

LTALSAVFGDYTDRRGFCALGSVKTNIGHTSAAAGVASIHKVLLCLAHRELVPTLNYANPNRHFDFADSP

FYVNTDRRAWDAAGDAPRRAAVSSFGFSGTNAHVVIEEYRPAAAAAPDASPPRVIVPLSARHPERLRAYA

RNLADWLAQAAARGAPERLAAHLAYTMQVGRDAMAERVAFVADGRDELERQLRRYADTGETSDGVYAGRA

EPHAQASNALMLDEAFGAAIDGWMRTGKHEPLAKLWAGGFDLDWARLYDGVPAAAMPRRIAAPTYPFASG

RYWIDVEPDGRAAGPDADAASPEADSGSDSEHAHEHEHEHEPAATLAYLPVWEELPPAQPRAAPDAQAGG

VLVVHRGGAWGLVDAIERECVDGRHAGATCMTLDLSGHAPSPEGRAWRNAAPGAARLAAWLGEFGPVRAV

FFAAGCSEARHDASGAHGWASAPDAHGEDERALLQLAQALMRSQAADASIEFVVLSLDHHRTDGTPSNPA

GGGVAGIAYAIAQGDHRFRVTNVDVSLDELRAARHAPAPHPVLAAVLRLAPSDRGALVRLRAGRGYRQAF

VRLDWAAEAGASGLKQGGVYVMLGGAGRVGRALTRRLIERYRANVAWIGRSPADSASVAHALRALGPAGP

APYYAQADATDAAQMRRAIEAVRQRHGRIDGAVFCGMVFDANHAIASVPAHRFDEILDVKARGSRIFYEA

LAHEPLDFLCYCSSAQSFSFSGAARLGAYAAATTAGDAIVRSIAPVAAFPVGTIHWGFWETSVEDSALGS

RHLGALSDDEGFACFERFVGQCMRGNPLREVVCMRASPEVEHLMQVLPGETATLAAPGQPAQPAPLRAAP

DGAAASEAAPPDGAADVSADIDAWLARLTFATLRPMLDGPRPARACHARWWDETLRIFAARGWLRIVDGA

PRVIAEPDAGEHVWRDWARYRFDTPAARGRRAQIDLADVCVRALPDVLAGRLPAADVLFPGGSMERVEGV

YRDNPISDYFNAVQADALIRHVRAWIDAGRREPIRILEVGAGTGGTTALALERLRPYAAAIGEYCFTDVS

QAFLQHAQAAFGARAGYLRTALFDVERPLDAQRMPAGRYDIVIATNVLHATRQVRGALRNVKACLRAGGV

LLLNEISEKSLFAHLTFGLLEGWWLHEDSSLREPGSPVLAPATWRRLLEDEGFGAIAFAARDAHALGQQV

VCATSDGVIRQRAGEPSGHSSRQGHRNRQDHQGRQESSTHAGEAGAPAPASAGRAGAAPAASPAGTAREP

VVAAIHRALQQSLKLPEARIGDHTPFLDYGIDSILGVRFVDSLKQALDVPLNTAVLFDYPTVERLADFIV

ATYGARLAARGVSAAPASVATASATLAASTASTASTAPTAPTAPTAPTASAAPAAPAAPNAPAAPAMPAE

AVSRDAAAPRAEPAGARPADIAVIGMAGQFPDAPDVDAFRALLEHARDGAPGVSGGMLENRDRFDHAFFH

ITPDEADAMHPYQRLVLQESWKALEDAGYNPAALAGARVGVFVGAEPADYRSTTFSGSSDALIASRVSYH

LNLRGPAYVVNTGCSSGAVAIHLACESLRRNESDVVLACGIFAAMGPRMLGALGQAGMLSAGGRCRSFDA

GADGTAFAEGIGVVALKRLADAIADGDPIHGIVKASGVNQDGTSNGIMAPNGVAQEELIVDVYERFGIDP

ADIRYVEAHGTGTLFGDAVEANALVRAFRRFTERSAYCALGTVKATIGHTAAAAGVIGLIRILLSMRARR

LPGMPGLGRANPMIDLDASAFSLGLVSREWPAGRDGRPRLAALNTFGHSGTNVHIVVQEPPQARARPARA

ADGPRVAVPLSAMDREALRRYAARLCERLEAEGAALCVGDVAHTLRVAREPMAQRIVLFASTTGELAALL

RAFVDGRDSPCLLDGAVTAAARAAGLDAAQLAQAARWLAGERVDWPPAGGTPMRVHLPAYPFAGRRCGAA

GWARAEAGASRDCAAAGPCEPPAGVAAAAMTVAAAGPRVDATPSAAADRARGAARPAEWLAARVAARLGV

PAARVDRRRSLLDLGLTSQDLVSLAGQLRDATGEALLPSVLFDYPTIERLAAHLADTCPAAFGAAEPAEP

AEPAETGRAAAGDAASGPAPGVIALLERLEGGGLSLEETIYLIENTK

>WP_024429026.1 CHAD domain-containing protein [Burkholderia pseudomallei]

MARVLEIVLDFSLQDWQATRGARVRAARDLGAELARAWRICPPVKMRRGHERVTIEPCRFVEAQPNDGSR

WQTWIETTAQARRVLAARCRPFVPGVTVREHFDDYRGDVRVATPLADEQAPIAAGSTPVPRDSAAAARDV

LDGGDAPPSRGRASRAAAARTAARAAASTPVAPDADVSGAADASGSSRSSASGSASESAAESASDSVSAE

STAPSAGAVPVGDAVAGTTSLESGSTARPAPDFVVERRRGRWLDADGIEVELTLDDIVFAPAAASSDTIR

AVASRVCELRLAVADPDDPDDPDDPDARAAALRALFNAARELSGAWPASLSPISVLDRACAVDAPDANGA

PAKAQSVDLSNTRTQRAAFFALGCSVTGQWLGNEAGVRDTAEPEYVHQMRVALRRLRTLARLFPRYADAE

WKDAFSGDIRWLAGMLGAVRDWDVCVTSTLPALAAADGDEAAWAGTLDAARAQGDAARAELRQALGTARY

TRLVFAWLEWLSLFPLGEDDPARGKAPSLKRHAAKRVSRLFGHLYGARRLTALDAAARHRVRIDAKRLRY

ALEFFSSLASRRTREDTVRLLARVQNALGDANDATVALRCLERLSAPPYQLGFARGYGAAAQRYAAEAAE

QLLRGMRAPKIGGRKA

>WP_024428952.1 ComF family protein [Burkholderia pseudomallei]

MANPAVLRCGIRAMAARAGIVLARFSAAALPNRCALCGNLSHRTICDCCDDAYWNEARLRCPRCALPLPG

ARGAMRFHCGACAKAPPPFDATLALADYRAPLDSLALDLKFRAQLALGREFGERLARLATDALDGAPPLD

VIAPVPLARRRLVERGYNQAWAIARPLARKLKVRADAALAARVADTAPQSRLAFDARRANVAAAFAVARP

VAGLHVGVVDDVMTSGATLDALARTLKEAGARRVTNFVALRTAKD

>WP_024428928.1 Rieske 2Fe-2S domain-containing protein [Burkholderia pseudomallei]

MDHPDADQEHERQLIACGREQYEWVVRAREAAASSLADWDARAEALLDGVRGMPAHFDHAAARYAARTGE

PLTLDHARAFCAATMLRAVEHPAGTDDWHLLCRLDEIPDIGDYVTGYAFQESMTAVRASEHEVRVFLNVC

PHRQTQLFQAAGSFDPHRHVRCPYHGWTFDVDGRCVNAPGARRCEFGADFREQDFSLSRVPARIEDGGIL

VRKAVSRVGRRMAALYERVYFAFPDVKRPFVPDRFRVLLRIGFLAADIRHARDSSILSALRAELRQHLLT

YPDLPVRYASLAKRHGEPTVDAVDAVDAVDAVDLGPRLAGGPLRETVATWVYGDAELHALERDTLVLPAW

HFVGHVAELRGGERTLTLDVSGERAYVCRARDGTIHAGRLVDIDRRTDDPARLRADGALAPIDVDVWQGL

IFVRLAAGRGGVADIWEHARMLEPYRIGQMAPLAGKGWYDFAVEADAKILWENFLEMYHFPAAHQAMIRL

FELSSRSDLLTLREPASTRLSPIEARYRDALKRGATHGADEEARQRAMADGDAALARPLQLTVFGSIPET

AQTATMLGITVFPDHIQAMSFIPVGPRECRVKIRSYGHAAEGALDIARQCNVEQLQIELVEEDIQLNHRS

QIAASTRSYERRGVLHDMEVSVADFQQLLRRVLPVTRCVRRPPVGALARANQALRLRDRDERGAPRRACA

DAARRARAPAAASAFAAPPPVSRQRNAETLEALDTPAFVYDERTLRDALSSMHTLLGDSGCRLLYPLKPF

SHAGALECIGPHVDGFAASSPFEAAFSRMLLGRAGSVHLATPALRDRDLGTIVDNCDYVALNSLSQWRRL

HDALAGRLSCGLRINPQYSVVADARYDPCRPHSKLGVPLDAIERVVASDPRALHGLQGIHFHTNCDSTDF

SRLAETIEHLEKRLARTLDGLAWINLGGGYIADGARYVDRFRACAKRLREHFGVALFIEPGAAIVRAAGT

IIASVVDLFDSGGMPIAVLDTTVNHYPEVFEYQFQPDVDGHHPAHAHAYLLAGASCLAGDVFGTYRFDAP

LRVGSRVAFVNAGAYTLVKAHRFNGINLPSVYRRGEDGALAAVQRFSYEDFLRQNGGLDHAYP

>WP_024428862.1 hypothetical protein [Burkholderia pseudomallei]

MRRVSVPTLLFAASACFFSLNAFSVRATFAAYALLALLALLRAPTLFARQQRLPAIVAYWLLSVSSAFLV

SAFGDFYANFFVKFVLIQTYVALAFWLFASGVLAMRSLERTCETLIYVHATFFIVQLGCYLAFGHFIDFD

SYIRESDSEALYATKALSDSLISIRALGLYSEPSFYAMTVVPAGAILLLAKRRMTAATIVAFATALLSFS

IAAILICALLGGVHFFAGRTSIRIKLVIAAVALAIAPAMYGVYDKRVNQSADYDALGSRTLVLRELRERD

ALADVFGSGLFWDERNNVGKTHLRGYQVRDSSFYVYLLFATGVAGATAFVGALFMLFRRRGRRSLLPYLL

PLLLFKFHALYGMLWLTLLMFVVVAGHAERLPERREPPGTGTGRLSATGASGP

>WP_024428809.1 DUF1929 domain-containing protein [Burkholderia pseudomallei]

MTEWIRVRSPAFKRKTKFKRFQWEKARDRSPFLYMEINMKKREENSLSVSHSRRAFLVWVPASLLVTACG

GDNGSSAPASIKKAVAALTGPGGATWTGMGKGGILIQSPYSGAGNFELLAGDEYGMLHCLRRTNDNGAQW

MVAVGDGMGSKNSVSGYCHTYSTNYTNIEAAVVDDGAVKTYFRSNSTPWSAHKTIPIGDAQGFPAYLQSN

RNGAQQLELVVGMRRGGMVHYWRDDKDGFTWKQSSIFGSGQVKGVSVMQGNYIGPDNNANLELVAWVDDR

LESWCCEGRAWRKICNIADGGVGGAPALLQSNYGTKGNFEVLVPLATGGIAHYWRNNDAEGIPWSFTGTI

GTGAYVAAGLLQSQNGVKGDFEAIGLRASGKADVFRRGDTLVWENSATVSPFSEGAAADVGVSSHIVDVN

VTGINSVLLKNGSVLMFGYYKGGSTNKTIPACIWNPVNDQITAIPSFRNNFCAGQTAMPDGKVLIVGGHI

GDTLKDVVVFDPDNHTATLVATMTKGRWYPSTATLPDGQVFIISGTETAGWNTSVNDTWQTYANNALTAP

EDVISPFSPYYPKSQTQIDLYPFLFVLPDGKLLVHARNTTRFFDIGTRSWSATLYKTVSDNSRTYPFMGG

TAVLPLRPSENYRVKVVVAGGADKSAQIAIGDDSQYDNSVPGMTSCEMLDLGDAAPAWKAIAPLNEGRVM

CDLVTLPDGKLFIVGGNKTGKADYGRGPTYRPELYDPQTNTWTLLASTRIARGYHATALLLPDGRIAITG

KDGDYQGSGLQYAETRVEIFSPPYLFKGPRPAIQSAPASINHGGSFTLGLSSGTSPEDIGSIVIVACGSA

THQINFSHRIVELVFAVSGGTLTVNAPPNANIAPPGYYMMFVLSKLGVPSVSSIVHVAAGSAQAASATPE

ALTRQPRVATVSIEQAGDAPRCAPLTVAEKQSINGMG

>WP_024428631.1 non-ribosomal peptide synthetase [Burkholderia pseudomallei]

MNQSYDVVETALRLRAGLAEHLGAAGPDVDEPLVDHVGGALPPALLVDVVRACGGMHATRVDVLGCATLR

ELAAWIERTGGAAREPRAAPDAAPNGARPEAPLAPEDADDAPASPAQEDLWLAAQVQEEGAAYHLSIALR

FDAPVDDASMRRALVEVIARHPSLRTTFRRDAQGLRQCVRAAAHAEVDWSEAVLPAPDEAGERESGLRDA

LTRFAQPRFDLHAGPLLRARLHRGAPDCDVVQIVVHHIVFDAWSRMVVSREIVDAYHALRAGRAIAWSAA

AASPGAFARGQRDALTPQRVARLREYWRAQLAGVPPVLDLPTDFTRPAVRDGIGATALRMLPAALAERLA

AVGARHGASLFMTLMAAWQILLWRCSRQHEFAIGTPMAARDAGAFESTVGYLVNTVVLRASVRADEPFGG

LLARVAQTVLDAHEHKALPLREVIELAGADRNPSHTPLFQAMFEFHNERPGAEAAQSAPVRAVPHDVGAA

KYDLSLEIAYRHDGLACTIEYATALFARSSIDGLLAQYEALLEQIAAMPDAPVGSLSCLPDGERERVTRT

WNATAFERTGSPLIHARFDARVRACPAAIALRTDAATMTFAALGERVDALASRLLERTGGEPERIAICLE

RSFDMVSAILATLKAGCAYVPIDPQLPADRVAFMLSDSAAALLLTIEPIRRERLASFDIDTLCLDAPAPP

RAAPPRAAPAVDPHAAAYVLYTSGSTGKPKGVAVTHANVTNLLDVMEASYPVGAHDRYLLKTNYAFDVSV

PELFGWFVGDGSLAILAPQAEGSPDLIVEALLRHGVTHLNFTPSLLRQFVTEAAADARFARGHRLRHVFV

VGEELTSALANDAWHALRPATIYNMYGPTETTVFATGYAHTAPIPNGRVPIGRALGNMRVYVLDERMRPM

PIGMPGDLYIAGDGVARGYLNRDELTAERFLPDPFTPGGRIYMTGDLARWTRDGMLEFLGRTDQQIKIRG

YRVELDEIASALNAHPLVGEAAVILKREPDGDARLVAYVVPAEGAAAAPSRDERARLRDALVGALEQRLP

DYMVPADYAFAHALPKGITGKLDRKALEALPVERPARDAALGAVAPRNDTQRALCAIWQAVLRVPALGVT

DNFFAVGGDSILSIQAAARAREQGIVFSARDIFRHQTVELLAAHVRWQQAPDEARRASAGDMPLLPIHHW

FFEVDSTHVDHYHQSRLLDVPAGVDAVFVRAWLAALVARHDALRLRLRDTPAGWRAHFAEAPHADASARL

MVCDVAWAGAPDAALDAFFADARRGVRLADGPLFVAALVAAGATGRGRLLLIGHHAVVDGVSWRVIVADL

RRTFAQWPALRPDAPGPASDAYQAWARALVAAADSPALHAERAHWLRVLRAPAPALRLDRAAGGDATRRA

TRIGAVSLNAADTRALLADTHRAYRTQTIELLLAGLLLAFRRWQGHDALRIALEGHGREADALAHYGAER

GLPDVGETVGWFTSYYPQWLTLAGAASAPAPAADSVAAAIMAVKTQYRATPHHGIGYGILRYVAGDPELA

AEAAAHAPEIVFNYLGQFDVANDEAAGIAVLDLSSRDDIAAARPREHALGIDGGVKDGCLAFEIDYSGAA

FDDESIAAFGAHFMHALREIAAHCKACAAWRPTPEDFPLAAVTQAELDVWHARHPSLETLYPATAIQRGM

VFHSLLPKQASAYTNQVHARVGGGTFDAARLRHAWQTVLERHAALRTAFVGFEREQPLQIVLAHAACPWR

DIDHRHLAPEARDAAFAALLAADKAAPFDFGRAPLMRFHLVRDDDAHYRFIWTYHHAVLDGWSVQLVWSD

LLRVYEALAAGRPAALPAAVQFDAVLAWRQRHASDADKRYWREQIGARTQRTVLDIEQAGLAAASPAAPA

VVERSLDEAASERLATAARACRVTLASVLQAAWALLLARHSGETAPVFGVTSSGRAIDVPGVETIVGPLI

ATVPARVDVDAALPLGDWLRAVHERHVEREAHAQLELVDILRESGVRGGQPLFDSLLVVENYPLAEPSAA

RILGLRDYGYAEDTHYGLTVSATPGPRLRVEINFDRSRYHIEDIEAMADNLKAVLTRVPDDLARPAGDVL

GGADAARAQAGAEGEPRLSRVSRQGELRLGRHLVPHLVEDHAAATPERRALVYNERAYSYGELNRAANRI

ANRLMQAFPDLGTDALVGVRVSRSDRLVLTVLAIWKIGAAYIPIDPVLPGQRMREMLELAGAKALVVDAA

VAAAEPAVAGVPRIAFDDLVQDDPRLEDNPDVHLSGNDLSYVLFTSGSTGKPKGAMIEHIGMLNNIANKA

LDLEMDEDSRVAQNASMSFDVSVWQMFIALTKGGTTFVYDERAVNDIAGLIRRMAADGVTILEVVPTYLI

AVVEYLEEHPECVRPASLRFLIVNGETVDATLIRRWFALFPATKLINAYGPTEASDDITHHIMSPGDEIV

NPVPVGRALANFDLYIVDDELRPVPIGTRGEIVATGVGIGRGYIGMAGATAQAFVKSPFPDRYKGRLYRT

GDLGEMREDGVLMFHGRKDRQVKIRGMRIELDEVEASLRAIAAVRQAVVLAIRPENREAFLCACVVPLDG

AREEIVDALKAKLPPYMVPSVFRFERELPQLPSGKVDRNRLREQCLNETPRASEHALAPRSPLERRLAEV

FGEVLGHDAFGVLDDFFALGGDSFKAIRIAAKYGPPLEVTDIYDHPTIEALAAHLERAPRAERSIVQMAG

DPATAKAALVCIANAAGGPVNFVEMSRAMAEQAGELAVFAVKLPRNAVDSDAAMLAEVTRLANAVCDDLL

AASGLPIIVFAQCNGSALAIAVARELTRRSADLRALCMGGALMRTASGKRDARTDDEILGFLGGIGSTLP

GRPDERAFFLHEFRYDSWLADVYYNHLVDEASRGALTPLDIPVWCLVGTDDPLVSQYETRHRDWLRIGRS

VKLAEFAGIGHYLLRDCPHALARTLGQAWEAVSRRSVEA

>WP_024428624.1 molybdopterin-dependent oxidoreductase [Burkholderia pseudomallei]

MSHFLDRLKFMSRVKSTFSDGHGAVVDEDRRWENGYRSRWQHDKIVRSTHGVNCTGSCSWKVYVKNGLIT

WETQQTDYPRTRADLPNHEPRGCPRGASYSWYVYSAQRVKYPMIRGRLMQMWREARKTMDPIAAWESISQ

NPEKARRYKSVRGLGGFVRADWNTATEIIAAANAYTIKRYGPDRVVGFSPIPAMSMVSYAAGARYLSLIG

GACLSFYDWYCDLPPASPQVWGEQTDVPESADWYNSSYLLVWGSNVPQTRTPDAHFYTEVRYKGTKTVAI

SSDYGEMVKFGDIWLAPKQGTDAALAMAMGHVVLKEFHASNQSAYFRDYVKQYTDMPMLVMLRERDGALV

PDHFLRASHLAASLSEANHPEWKTLAIDAATGDIVAPNGSIGFRWGEAAHNGGEKVGRWNLEMKDGGSGR

AIDPRLSLVDAHDEIVDVGFPYFGGEHEAVLARRVPAKRVALADGTSALVATVYDLQMANYGVDQGLGGP

NVAASYDDDIPYTPAWQEKHTGVARHLVIQVAREFADNAGIRDNDWVEVFNVNGTLTARAVVSQRVPAGM

CLMYHAQEKIVNVPGAQTSGKRGGIHNSVTRTVTKPTHMIGGYAQQAYGFNYYGTVGSNRDEYVIVRKMN

RVDWLEEPLNEGAEQ

>WP_024428610.1 polyamine ABC transporter ATP-binding protein [Burkholderia pseudomallei]

MNSQSGAAMAGAPSYRPQTGADGRAENFVQIIDVVKKFGETVAVRNVELSVRKGELFALLGSSGCGKSTL

LRMLAGLETITSGKILIDGEDLAQMPPYKRPVNMMFQSYALFPHMTVEANVAFGLKQEGVPKAELKERVR

DALELVQMARYAGRKPHQLSGGQQQRVALARSLVKRPKLLLLDEPMSALDKQIRQRTQIELVNILDKVGV

TCIMVTHDQEEAMTMAGRLAVMSEGQIVQIGTPHEVYEYPNCRFSAEFIGSTNLFEGATVEDEPDHVFIE

SPDLPCRLYVNHGITGPLGMPVTVSVRPERIALTRKPADGAYNWGRGVVTNVAYMGGYSLYHVKLDSGKT

VIANVSSLAIADLDSPGWGDEIYVRWSAAAGVVLTS

>WP_024428500.1 cytochrome c [Burkholderia pseudomallei]

MALIAARGPRLRFSAAAATAATASALVMAALLGGCERDAGDAGGAGGARAAAAPPPASAASAAQAAGARA

AARIASDASRADPAAIARGRYLARAGDCAACHDAADHTPYAGGQPVNSPFGPIYASNITPDPDAGIGRYS

LRQFADALRLGKAADGRRLYPAMPYPSFAKLDDSDVAALYAYFMHGVQPSDKRAPATRLPFPFNQRWGLA

IWSALFGNRERFVPNPQRPAAWNRGAYLVQGLGHCGACHTPRGPAYDERGYDERSAAYLTGGVNDHWFAP

NLTGAPLDGLGRWSERDIAAFLRTGHARHGAAFASMAPVVEASTALLSDDDLHAIAVYLKSLPAQRTPAI

ALVPGRARQPPARAADDGTQQRPGAGVYFAFCARCHGADGAGTPDRGPALAGNSLVLAPDPTSAIRIVVE

GSAEPRVAGRDARRMPGMRGRLTSTEIAQVVSFVRGAWGNQAAPVSDREVESLRSAVHR

>WP_024428451.1 SDR family oxidoreductase [Burkholderia pseudomallei]

MSMKVLLIGASGRTGRALADLLLKQQDFEVTALVRRPDFALPGAKVVVADLTGDFSSAFNGITHAIYAAG

SAESKGATEEEQIDRDAVARAADYAKARNVQKLVVISSLSAYWPQSSPEFLRHYSQMKREGDERVIASGI

DYVILRPGPLTDDPGVGKIALTEERLDDAPPVARQDVAWAAIEAIKLGISKKTIGFVGGSVPIEQALRA

>WP_024429113.1 amylase [Burkholderia pseudomallei]

MSARHFGFKQAVLVGSLAALAGAAALPANTSAAEAFGSPGAAPVRGPAAKSFLGTAVNGASRVYFTGYRG

ILSEVYYPVLDTPESVDLQFLVGDAGKTFVDEEKQQAYSAAQTDTRTMSWQVTTGNSSHNWQIRKIVFAD

PNHNAIVQRVTFTALDGRKVGDFNLYMLSKPYLDNAGANNTAQTVSGSGGTALVANHNSRYSALVASRPW

KVVNGVGMTSNGFVGQSDGWTDLFGGTADKTMDWTFSSATNGNVAQMGWLDLGDPSATSVSFDVVLGFGG

TQDQALGDANTVLGSDLAGEQQQYDTGWHNYAAGLSSQNGAADDGYYLAAMTLKTMQDKSNGAMIAGIGT

PWGETQGDANAGGYHLVWPRDLFKFANALTTAGDTSSATSVVNYLFNTLQQTTDCGTAEYNAPGCAQGYS

RVGRFPQNAWVNGWPYWQGTQMDEQAMPIILAWRLGPSVFNPLWPKIKLTADYIVNTGPWTYQERWEENA

GYSPSTIAAEIAGLVTAADIATQNGDTASAARYLAAADYWQENVAAWTYTSSGAFGNGSYYIRINPAGRA

GTGTDRASFAPTAGPDTPQTLSVKNGGGSHDARRVVDGGFFELVRMGVKRATDPTIANTISVYDSVLGQT

LNVANAPALSPNAWFRYNFDGYGEHNDGGDFDGTGAGRLWPIFTAERGMVEIARQGSGGAGSAYFSTLKQ

LTTPEGFVPEQVWSNSTTLPDGWAVTTPAGYTPGQPTKSMAPLNWAMGEYISLLASIQAGRIVDVPSVVC

ARYNNCAAPPQSGQVPVAVNVNASTQLGQQMYVTGNVAALGNWNTDLGIPVDPASYPVWRNTVNLPAAQA

IQYKYYRKNADGSVTWENRSGNRQLQTPASGTLALNDQVSW

>WP_024429033.1 acyltransferase [Burkholderia pseudomallei]

MNASSMASGARPSATDHKEHLIDALRGFAALVVAYFHCRQVVWVGMQHFHRTYGHALEPGAIVGYLTFPF

AWGSAGVPIFFVISGYCIHRNAALKLAADPSYRLNAPNFWTRRFARIYPVLLAALVATLVFDSISLQIEP

VSHKIRDIGLASFVVNLLSLQGVAGYTYGSNGALWTLSLEVQFYAIYPLLFAARRRFGMVPVVVAIALVN

VASAWLLERHDLQFFTSYWLSWTVGAWIADARAQRGACAAPAPSRRWYAAAALGIALGCVAFHFGQYGAF

QLWAAGFACYLHAALARPAPAFAPMRALSWFGDFSYSLYLIHLPFFVCLASVLYRSEPQLSIWPSFAFIA

AVIPVAYLFYRMFELPAMRWSTSLKPKRAVAASARQQMPV

>WP_161645725.1 DUF4148 domain-containing protein [Burkholderia pseudomallei]

MMSKAKSIKHSEVLMKSLVYAVVAASALTASFGAFAQSSQPLTRAQVRAELVQLEKAGYHPGVSSPYYPE

DIQAAQARVQGADTSGYGAQAAPVAHVGAPVAAAPRSPRESIYFGQ

>WP_161642416.1 zinc-binding dehydrogenase [Burkholderia pseudomallei]

MHRAIPRAAGWSAPAAQRLPCAWARAVAGASEIREARRSREPAASDEQGASMKAAVVHRAGERPTYAEFE

PPRALPGHRLIDVSASALSRLAQARASGAHYSSTGGFPFVAGVDGVGRLDDGRRVYFFGPPAPFGALAER

TLVPAAQCIPLPDSIDDATAAAIAIPGMSSWAALTERARLAAGETVLVNGATGASGRLAVRIAKHLGAAS

VIATGRNAHALDALSSAGADATISLAQDDEQVARAFEAHFRAGVDVVLDYLWGASARAALLAAAKAPQQA

RPVRFVQIGTIGGAELPLPGAVLRASAITLMGSGLGSIALPRLLNAARAVLGAACEARLRIDTRTVPLAD

VDAHWGDTGSTLRPVFTMRAPG

>WP_119637616.1 DUF2591 family protein [Burkholderia pseudomallei]

MRTDRLDGTRLDYWCARALASDDATLRFVAVAPSVVVTVTSGALRKLDQRFAPSASWADAGDVLARADAL

SVARRDDGRVECTARFAGVRAVGCADGPDLRVALARAFVRARFGDAVDTPPEKPHQVRGGKIEPYDAGEP

IPQHGDASATGNAGDIRSVPRM

>WP_041222426.1 DUF3348 domain-containing protein [Burkholderia pseudomallei]

MLGRAVAVAAAAAAPCSLADAPPATPERAPIPSSRIAPKPPVARSTSRTPAHASPTCKLLPFFDIDTRRM

MQVPQRSALGGPTLIRLLARLAHADVPPSGQSLSDRLSQWLAWTDAIALSSALSMSPPAAAAGARPAGDD

PHGQGARVRASLAKAIARTGAFAAGRDGAARVAARGAPHDTPADFTLFRHDYLSMQQAMEIDIGELRGRL

RQTMAAQTPALARLAALDATMERALVARERSLFASVPKLLGAYFERLREAEQQRLAEAEAKAHANAEADA

HAEVARKTAAPAPHAWLDAFRQDMQSVLLAELDIRFQPVDGLLAALRAS

>WP_038793679.1 MipA/OmpV family protein [Burkholderia pseudomallei]

MPFAGFVSITAAQAENVYMLSLGAGFAPRYAGSSQYRAVVAPSFSAEFDNGIFVGPLGGVGYRLKLPGQA

FVAAAVSYDDGRADEDRFDRPGSNYLKGMGNVPGSVIVAVQAGVVLYGGSVLSVSVDTPVTHTSRGVSGH

VDLAVPVFSAGRHEIVLTGSVHAASGRYTQTFYGVTDAQAAATRFAPYSTKGGIDSASMSVAWTYTFSKH

WSVDTTLGLTRLVGRYGNSPIVQRKTQYFGLSALTYRF

>WP_024429102.1 DUF2244 domain-containing protein [Burkholderia pseudomallei]

MTARPRTRRHARAHADAGANANADADADADADADANADANADANADADADADADADANVNMKADARCDTR

KHAQRHCTHAFVAHDPRLMTRDVAENRRVWLLKRNCALSPRQSLISMGLLAALTLAIAMPFAIAGAWAVF

ACAIGEIAAMCGCFLRYARHAVDYDCVALTEQRLEVIQCDGAQLRRYDWNPLWVAVDLDAADARDPTIRI

RHGSETVLVGRHVTLARRRHVAEELNAALAASAGDFGDTDFAPSVRQRQPEPLAAHR

>WP_024428860.1 helix-turn-helix domain-containing protein [Burkholderia pseudomallei]

MQAGRMAEIFNLANRFRHAGEDSGGYEMLLLSATGGAVYSSSGIPVWTQPLHERAIERVHAIFAIGEPDP

ADRDDEKVSEWLRSARAHVRASGGARRLMGLAALERGDAAADDLSIDAMLSGAASAEKVRPTAAERAAFS

AALAIIRHDCGDSAAHEVAERLCPALSARPAESAFAAREARASKLIRASVQQLRDNSANRISIADTAHAA

AMSERNFLRRFKQEIGVTPSEFVQKVRLEHACHMLVHTDLPVDKIARRTGLGSGDRLAKLFRQHLSMSPT

EYRAIERSRGADADLACGDFVSRLSGSVS

>WP_012729940.1 DUF2249 domain-containing protein [Burkholderia pseudomallei]

MLQAGRTPSALSGEWRALRTGRAPRLSPFKDQVMNEQYASIIDVRRIPHHKRHALIFGTFDALPGGEALQ

LINDHDPKPLYYQLEERAADSFEWTYLEAGPSQWRVQIAKRGDALGHHDAGDSCCSGGGCGG

>WP_009973107.1 chorismate lyase [Burkholderia pseudomallei]

MARMRFDAADAHWRETPRPGASSAQKDWLTRGGSLTAHLARLGRVTVRVTRETVAAPWADEHRALSCASR

APVWVREVVLAVDGAPFVAAHSIAPLAASKGVWQAMRRLRTRPLAELLYSDPEVTRSALVSRRVLAGHPL

FSLASLALVRAHATEHAFAARRSVFERRGTPLMVTECMLPALWRHLDAHGERRARGLEQT

>WP_009970868.1 oxygen-independent coproporphyrinogen III oxidase [Burkholderia pseudomallei]

MDMTTQSNDGADTTAPAPQHDVSAFADVQISEALIRRFDRQGPRYTSYPTADRFSDAFDERAYREHLSRR

ASAERNPPLSVYLHLPFCESLCYFCACNKIITQDHTRTSAYVDYLIREMELVAPDLGRDRRTTQLHLGGG

SPTFFAIDELARLMRALREHFDFAPHAELGVEIDPRTVNERTLQSLAALGFNRTSFGVQDFDPSVQEAVH

RIQPLPMVERALEASRAAGFESVNIDLIYGLPRQTPASFSRTLDEVIRLSPERIAVYNYAHLPSRFKAQR

LIVEAQLPPAEDRLRIFIESTRRLLDAGYVYIGLDHFAKPNDELGNALRERSLHRNFQGYTTQAECDLVG

FGVSAIGKVGASYSQSTRSLKTYYRQLDAGRLPIERGFALTADDLLRREVIMTVMCSTPVDFAEIGHRHG

IDFARYFAPELAQLEPYRDAGLLTIDAQRIAVTPKGRMFVRAIGMVFDAYLGRSAAASYSKLI

>WP_009941461.1 phage portal protein [Burkholderia pseudomallei]

MSSWRRRASTTAPSACSNVTSSAATTLRNRPRQIRAPARAEVFTFDDPMPVMNRAEILDYVECWSNGEWF

EPPVSFAGLAKSFRASTHHSSALFFKANVLASTFRPHRWLSRHAFERWALDFLTFGNGYLERRRNMVGGT

LRLEPALAKYVRRKADFSGFVYVNGWQERHEFEPDSVFQLVRPDINQEVYGLPEYLSSLHSAWLNESSTL

FRRKYYENGSHAGFILYMTDAAQKQDDVDNMRDALKNAKGPGNFRNVFMYAPGGKKDGIQLIPVSEVAAK

DEFFNIKNVTRDDLLAAHRVPPQLLGIVPSNSGGFGTPDTAARVFGRNEIRPLQARFAELNDWLGDEVVR

FDDYEIPPAPVAA

>WP_004553190.1 MULTISPECIES: DUF1275 family protein [Burkholderia]

MKPNLPSLLTFNGGYVDTAGFLALQGLFTAHVTGNFVTLGATLVAGSTGAIAKLLALPVFCGVVLAAGVA

RRLMLRAHAPALKILLGVQWLLLVAGAALAIRLGPFANGDAWPAIATGMALVMAMAIQNAVHRLHLPDAL

PTTLMTGTVTQLMLHLSERIAGDAAPVRGSPARLANMAWTVVTFALGCAAAALAYLLGGAWAFALPPVLI

AFALFLGDERKLEHAL

>WP_004548157.1 M48 family metallopeptidase [Burkholderia pseudomallei]

MQAKLRVARAALTFVGGVALGVAGPVLGFELCMAEPMLVADNGAPPVPSAPAVATPAAPAAAPAAGNPQP

YALGDQQVRYGNAIVFRSLIPSPLLEQLTDNEYRQTVQDAAQRKRLLPPNNARVKRLRTIVMRLAPYAVK

WSERVKGWNWEIEVLRSRSIRAFCLPGGKVLVDSGLLERLRLTDDELGVLFAHEIAHALREHARSSLGEQ

QAASLGTGATPLPPLFGLSEPLPAPLGVVERFASVRYDPTDETEADVIGGDIAARAGFDPRAAITLWDKL

AVATRADKDNGFIHAHPYDTRRRNDLRKRLADLMPLYRKALVKNADARANAAGAGAAAGAKQRGAATANR

>WP_004548112.1 diaminobutyrate--2-oxoglutarate transaminase family protein [Burkholderia pseudomallei]

MQDVDPAYAFVRARESGARTYADTFDTVFAHGAGTVLTDTAGRRYLDFLSCAGTLATGHNHPAVVERVRA

FVDSGQAMQMLDMTTPVKHRFVERLFGILPPALARHARVQFCGPSGADAIEAAIKLFKTATGRRSVIAFH

GGYHGMTAGALALTGNLRAKHAVASLMPDVHVMPYPYAYRCPFGLGAPHTAAASLHYIESMLSDPESGIA

KPACVIVEAVQGEGGVIPAPPEWLAGLRALTARLDIALVIDEVQTGIGRTGAMFAFEHSGIRPDAVVLSK

AIGGGFPLALVAYDERYDVWQAGAHAGTFRGNQIAMAAGVACLDVIESEGLIAGAAAKEAHVRARLERLA

ARHPEIGDVRGRGLMWGIELVDPDAAPDAAGARPAAPALARALKRYCFAHGLIVETGGRHGAVVRLLPPL

TVSAAELDLAFDTLDAGLAALGEHARAEFA

>WP_004547134.1 glycosyltransferase family 4 protein [Burkholderia pseudomallei]

MNLSSPLSNPSPRVRRDRQDFDVTTPSVSQAAGVLRDAPPRAAFAGDHAGVAALPRPVAINGKFTSQRLT

GVQRVAHEFTSALARLLPGERNPTLVVPRDHASDTLPPTVARRVVPRLRGALWEQLALPFATRGQTLVSL

CNIGPLFKRNQVVMIHDVAVLDFPQGYSLKFRLWYRFAFWMLKRRARHILTVSRFSKERIVARLGVAPTD

VSTIVSGVDHFGRIEGDPSVLDRLGLAYDGYVLIVGSLAPGKNLARTLEAIARLERMRPELRFVIAGGSN

VRIFGASALGERASAGNVTWAGYVSDGELKALYENAGCFVFPSLYEGFGLPPLEAMYCGCPVIVSREASL

PEACGDAALYCDAHDAIDIAATIAQLMGDAELRRELREKGRARASRYRWDAAAKQLIGVLRALD

>WP_004546982.1 flavodoxin family protein [Burkholderia pseudomallei]

MAGVSAEFKAFEEATSGAVMTKGFLWRSKIAAGFTNSGAHAGDKLSMLMQLALFAARYGMHWVNLGLPPA

NDSMAGSPAELNRLGFGLGAGAQSNTDQGPDAAPPEQSEQSEQSEQSEQSE

>WP_004546013.1 cytochrome-c peroxidase [Burkholderia pseudomallei]

MMRRLPRYARQHRSFFVAPRAFAAAAALAAGVAACDANGPGASAAAAVAPAALAVPAASAASAASAARPA

PLAQPAAPAVVDSQPQTRAQVYEAVKQMTALGRQLFFDPSLSGSGKLACASCHSPQHAFGPPNALPAQFG

GDDLRQQGFRAVPTLKYLQKVPAFSEHYHESDDEGDESVDAGPTGGLTWDGRVDSGAEQARAPLTSPFEM

NGTPEKVARAVRAAPYAPAFRAAFGARVLDDDRATFEAVLQALGTFEQAPDVFYPYTSKYDAYLAGRARL

TRAELHGLQVFNDEKKGNCASCHVSRRGLDGSPPQFSDFGLIALGVPRNRALAVNRNPNFYDLGACGPER

RDLKGRDEFCGLFRTPTLRNVALKKTFFHNGVYHSLDDVLRFYAERDTHPEKFYPVKRGVVQKFDDLPKR

YWKNLNDEPPFERKRGDPPAMTDAEIRDVIAFLGTLTDGYDPRAKPAGGAR

>WP_004545670.1 glutamate--cysteine ligase [Burkholderia pseudomallei]

MPNNMTSRQTDLLQQRLAVLSTSPTREQLPDGLRGIEKESLRVTRDGMIAFTPHPRALGSALTHPSLTTD

YSEALVELITPAERDAATTLERLDELHRYVYASLGDEMLWTDSMPGLLPADDEIPIADYGTSNIGRLKTV

YRRGLAYRYGRTMQCIAGIHYNYSLSEEVWRRLHAEEGSTAGIVDYQSERYLALIRNFRRTSWLLMYLFG

ASPALDAGFLRGRAHKLDTFDAATLYRPHATSLRMSDLGYSNTTAQAALQADYNTLSGYLDALSKAVSEP

YPPYEAIGTHRDGEWIQINTNVLQIENEFYSTIRPKRVTYSGERPLHALASRGVQYIEVRCLDIDPFEPT

GIALDTARFLDAYLLVCALDESAPLTCDAYRESNANFGRVTMEGRKPGLELTRDGAAVPMRAWADEIFAQ

IEAAGRVLDDIRGDDAHARAIAAQRAKLDDPELTSSARVLRAMRENGQSFLEFARAQSAAHAAYFHGRPL

DEAATRDARALAERSLAEQAELEGKNAGSFDAFVAAYRAYTLNRFSV

>WP_004538570.1 RDD family protein [Burkholderia pseudomallei]

MIAPALPSEPPPPSVRRRLAALAYEGLLLFGVVFFAGLMFGVALQQRNGLDHRNLLAGWIALVVGVYFVW

FWTHGGQTLPMKTWRLRVETARGAPLSAGRALARYALGWLWFLPPLALHPLAGFPLPRTLAATAVWFALW

ALAARLHPSRQFPHDRLAGTRIVDAPRRG

>WP_004536796.1 diaminobutyrate--2-oxoglutarate transaminase family protein [Burkholderia pseudomallei]

MHRFETIRQLESSARTYASSFEAVFESGAGALLRDQSGREIIDCLSCAGALPLGHNHPEVNDAIRRFFQS

GHVQQALDLSTPAKFEFVKTLFETLPDDWARRAKIQFCSPSGSDAIEAAMKLTRFATGRQTIVAFSGAYH

GMTRGALAAMGNLGVKSGLGLGAGDVHFAPYPYRFRCPFGTDGTETDALSIQYLRNLLADPESGVSKPAA

VVVEVVQGEGGCIPVSDAWLRALRQLTRAHEIALIIDEVQTGFGRTGSMFAFERAGIRPDVLVLSKAIGG

GFPLAVVVYDEVLDVWPRGKHAGTFRGNQIAMVAGKATIEILRRDRLDAHAAQMGALLVDGLREIARAHP

EFGDVRGRGLMVGVEIVDPSSDGAPQDGELARAIKLEAFRNGLLVETGGRHGAVLRLLPPLIVNRADISA

ILERLDASIAQAKRGRGPRGGANHRSDERDGEAARPVAQGRSALPQAEPVKREAERRRGLLKPAFLLLWL

GETALDFGSALMSFALAAWIFQKTGSAERFSFAVLSAAIPALLLTPVAGALADRFDRRWVIAGCDVAMAI

MIGALAWLLFRGALAPGHLYFFNATSAAIGCIRMPAYRAAVTAIVPRERLTQASGFTGTSQALLRIAAPL

IAGYVLADYGLKGIVGLDMLMIAAGSAAIFAALLRAAHAIRGVVGAVDASLIEGVSASFAAAVRYFKSVP

MFQGLAAYNMLQESLLLLSSVMLTPLVLSTHSSSTLGLILTYGALGGLAGSLLLLVLRIEARLMSLALFA

DAVLAMLIALVGFSRSPAAWCAFVFLAYFASSASSACTSALWARKTPREMRGSIFALNTSMNLAAMSIVV

LVGGVLCQRLFEPALADGGAWAPTVGAWLGTGKGRGIGLLLVLCGAAGCVASLLALTCTRLRHLDALVPD

QTHDDARDLLRNGVAVT

>WP_004535560.1 sensor domain-containing phosphodiesterase [Burkholderia pseudomallei]

MEAIRNKTPRKGSWRAALHQLRRFAWSGGALMPARAGTPRADDTVRSWLEQTVGAVDFLAHVDRDLRFLY

VSDASLRFIGYHRDYLHTLTLRELIPEQDTATLDGLLARASQTGDVEKATLCLVKSLTYPLDVEVRAVRS

RHHGIDGFAIAAFDVSSWRAIEARLTYELHHDPMTGLDNLSALLPALMRAQQIADEGGGCTALLLLDLDD

YQRINRALGYDAGDALLRDTAQRLQRLATRGERLARVASDKFAVMLSAPTRVAALDAAEALARQLQTAVQ

RPYAYDGQAVHLSASVGIAIYPDARATSKHAQHHSPLLRRADRALAQAKAAGGNALAFHQPTDDPADAER

LKLEADLYNGVRNGEFSLHFQPITKSLSGAVVGVEALIRWHHPVHGLVAPATFIPLAESIGLINYLGNWV

LKAACMQLVSWDRQGIALQYVAVNVSPQQFRDPRFTQSVRDAIKLTGIDPRRIVLEITESLLMHDPNHAK

TLLEEVTELGIRFAVDDFGTGYSSLAYLQSFPLAKLKIDRSFVENLLTSRNDRAIVSAVVGLAQTLELEL

VAEGVETEAQRTLLTEMGCNHIQGWLVCQALPSDELAQRFEAQELHLHATA

>WP_004535351.1 sugar ABC transporter ATP-binding protein [Burkholderia pseudomallei]

MVSGPRDEADDMDEASGAARAPDEASEEAMDTILALTGITKRFPGVVALRGIDLRVARGEIHALLGENGA

GKSTLMKILCGIHPPDEGVIALDGEPRRFANHHDAIAAGVGIVFQEFSLIPELNAVDNLFLGREWRGRLG

LRERARMRRAAADIFARLDVAIDLSAPVRELSVAQQQFVEIGKALSLDARLLILDEPTATLTPAEAAHLF

GVMRELKRRGVAMIFISHHLDEIFEVCDRITVLRDGQYVGTTEVARTDVGALVEMMVGRRIEQSFPPKPR

LARDAAPVLEVDALQVRENGPVNRFALREGEILGFAGLVGSGRTSSALALIGAKPARVRRMRVRGRPVRV

ADPAAALAAGIGLLPESRKTQGLIPAFSIRHNIAINNLGKHRRLRWFVDAAAETRTTLELMQRLGVKAPT

PHTRVDTLSGGNQQKVVIARWLNHHTRILIFDEPTRGIDIGAKAEIYQLMRELSARGYSIVLISSELPEI

VGLCDRVAVFRQGRIEAMLEGEAIEPNTVMTYATSDVRGANHEHA

>WP_004523328.1 PRC-barrel domain-containing protein [Burkholderia pseudomallei]

MNTPQQGTTRIIGKGRASAAGPGPDVMAASTLDGDKVYTTDGDDVGKIKEIMLDVRTGRVAYAVLSSGGL

LGIGDKLLAIPWSALTLDTDRKCFLVAVSSERIKNAPGFDKSHWPTMADPNWAASLHQYYGSAPYWSDIE

EELGIDAPPHEASPDRPERGGRH

>WP_004198117.1 MULTISPECIES: DUF3563 family protein [Burkholderia]

MFAYIIEKLSNWFEAAERERREAYLASSSDIVQLEQRIRSLETNGYSL

>WP_004190675.1 MULTISPECIES: presqualene diphosphate synthase HpnD [Burkholderia]

MAVSNPVVDDNETDAAAVTSGSSFYLAMRILPAEQRDAMFQVYAFCRAVDDIADEGGARAERAAQLDRWR

ADIDDCYAGKPRASLVPLAREIGKFGLHRDDFHAMIDGMAMDAAEDICAPDEATLDLYCDRVASSAGRLS

VRIFGMPDEPGRVLSHHLGRALQLTNILRDIDDDAAINRCYLPRELLAREGIAIADPQTIARDPLLPRVC

ATLVERALHHFAQADAVMDASPRAQVRAPRIMSGAYRLILEAAVARGFAPPRAPLRKPRARMLLLAARYA

LF

>WP_024429030.1 membrane protein [Burkholderia pseudomallei]

MTPRLLSSTTIFDGLPRARRAVALAVLALAGASAAYAAPDAASAASAEQAAPLTITVRPGQSLNDIAVAV

TQSHDPGVLARAGRALFDANPQAFMKRDPSRLKIGAQLMVPALDATGGAAASGATAASAAASAASTSAVT

GAAPHPSASASAPAAASRPAAASGASHAATASGAAAFAASGVAAGSASGANGASAPGVSAPGAASAHAPS

AVAPAAPVASSGAGSAHVWSGSIQQAPAGANGASAAGAEALLAPGAANRAPSGSGAQAAGASQARPSSLQ

QLLALKNRVLMELQKHGIGKPAANVAPAPAAGSAQKPQASAAAGAAASATAAATTASETIASDAAAGSAA

SATPASAAPAASVPAAAPRVSAPAHAAAPQAPFNTGAAVAIGAALAALIVGFALRRRKKGASADAIEPGA

FTPAPALDPDAAAAIADAPAGPNAAEDARHDEAEPDKAAAARRASSAAPLAEAKRPPAQHGSEPPSADED

AAPAREPQAPAARPLTLKTPRPDEQGGAPQSAGDEPSGAKPAPYAWRQAFPSDAIAALDSLDMPLPPREA

GAAADRPEQSQHAEPAGSRDAQSATNGLTAAPPPMGAENAREGQTHGEAGSATDFEGAGSFDNDARVAPQ

RPEGATPRPVTAPDAMGRDEAAARAAESSPTESTRDAPRTPGAPAAESRLSTAGAGAAHSPMPDAAAAAE

AAAPHAATPSDAAPHDDGQTQAAPRPLDGGPQPPAVARHEAAPAHAEIPNAPLAPAAPAWAAPQPPGMSF

AEPAQAEPGDVPAAPDRAPAANPPPLGVAQFGALNLDFDLELPSGPSASLPAFTPEALAKIARNKLELAS

EYVELGDLAGARTLLHEVIEADHADTRDEARVMLAKLADLS

>WP_004548018.1 ABC transporter ATP-binding protein [Burkholderia pseudomallei]

MAPVRRFGSAAASEIGSQNDPGVRSKAIDALTRAALAADMLDDPRTATSALDVSIQKQALNLLTNLQKKY

KLSYLFITQDLAVMRAMAHRVRVMKEGRVVEAGDTPDVPDAPSHPCTRSLLASSMPAARRSPRENEDD

>WP_160924666.1 pseudouridine synthase [Burkholderia pseudomallei]

MRTKLTVKNPRPATPGRAPVRSGSLTARKVARPDPKAAGAKPAAAKPAAKSASAAKPAAPRGAANAAPKR

APGPSHPAAASEGKRVAKPRVAHDAGRTGGERAPAKRATAPGAPGAPGAPSAPRTRRTDAKPARRTDERP

AGRAGNRPAGRDERAPRDSDARAFDAGTRGKDRAPREGARPGARGATGAKFGGAAHRSDDADRRTPRATR

ADSRARDAAPSSFAGKTTTAGKHAPQRADDRYGAAGKRTSPRPERTERPARFGERPATRASASGERRPTA

RAATGSRLKLAQPIKRGGGELGESARGGEHGEHGERGKRIERGDETGLVRLSKRMSELGLCSRREADEWI

EKGWVLVDGERIDTLGTKVRADQRIEIDSNARAAQAAQVTILLHKPVGYVSGQAEDGYAPAATLVTRENH

WSGDRSPLRFSPQHLRALAPAGRLDIDSTGLLVLTQNGRVAKQLIGEQSDIDKEYLVRVRFGERTADIER

HFPAESLAKLRHGLELDGVPLKPAMVSWQNGEQLRFVLREGKKRQIRRMCELVGLEVIGLKRVRMGRVML

GALPQGEWRYLGPDESF

>WP_038753017.1 trans-2-enoyl-CoA reductase family protein [Burkholderia pseudomallei]

MIIKPRVRGFICVTTHPAGCAASVREQIAYVARRGPIERGPKKVLVIGASTGYGLAARIAAAFGAGAATL

GVFFERAPADAKPGTAGWYNSAAFHDEAAARGLQATSINGDAFSDEIKHKTIDAIRRDLGQVDLVVYSVA

APRRTHPKTGVTHQSTLKPIGHAVRLRGIDTDNEAIKETLLQPATPDEIADTVAVMGGEDWRMWIDALDA

AGVLADGAKTTAFTYLGEQVTHDIYWNGSIGEAKKDLDRTVLALRGKLAARGGDARVSVLKAVVTQASSA

IPMMPLYLSLLFKVMKARGTHEGCIEQVDGLLRDSLYGAQPHVDAEGRLRADRLELDPAVQARVLELWDQ

VTDDNLYTLTDFAGYKAEFLRLFGFGIDGVDYDAPVEPNVRIPNLIE

>WP_024428857.1 oligosaccharide flippase family protein [Burkholderia pseudomallei]

MRAREREDATMIRVRDSLISLCGVVAGQAALFGCISLIGRFQGPEALGHFNYLLALATFAGTLLACRYEL

ACVSDNPADSFNAFVNVMVLAGAAVSIGCVAIAVAGRTDLYIVAAYGFAAFAQLAAGGYLNSLRRYGWIA

ASRIAVNGSFLVGVLAATFTPTFRARSGSIDAFRAYAAITAAVALLMLIAVALHGRRRGYSFAVTRAFFV

EHSRFAKYILPSTLCGSVLTYALAIVIPHWFDAQSAGYFAAAYRLGFFPVSLIAQSLGGVFRRDAMAAMA

GDDAHMLVPKVFGAYARALALLAAGYALGGALLFGPLVELFFGARWNGAIAFFLCMIPLFAFQMIYVPLS

QIFLAARAQRIDFLFQLGCGAVLAGALVTAKLAGLPVLASVRLFSFSGAVLMIAGIALTFSVMSGSVSRR

RAGA

>WP_009941093.1 AAA family ATPase [Burkholderia pseudomallei]

MDLKAQPIPAAPHAKRNDGLASLGWSVVRHRRLFATIVGVFFALGVLYALLATPQYRAEALLRIQTKPGA

SISALSDVSGTISNGPSANDESEVLTSRAIVGAAIEQIGANLEIRTLDHFPLIGRLLASGHANDDKLASP

LLGLSGFAWGGEKLKLGEFSLPDAALGEKFRLVAGDDGQWSLYDKKDRLLARGALGKSVPFAVALDGERA

QPGRIRVDTLRARSGIAFSIVKEPTQLVYDDVMKKMKTVVSNRDSTLEEPSMMKLVYQADTPQRVQEMVN

AIVRVYLDQDIKYRAEKAQRNLDSLHARLPGLKKDLEKAEDALNRYRTTTGTIDVDQQGIALINRLNSLS

EHQTVLQLALDNVKARFLPGSTTYQTVQTQLDQVKKEIQQTTTAANKLPTAQREFVRLSRQVSVATQLYT

SVLTNAQQLEIAVASTTPGVSVVDWANKPYKKAWPQRGIVVLGALLGGLFAGLAAAYLLARHRDELSGPQ

SIADVSDIPCVAVVAPSAESLALGDDRPGNARARVKPLAAQRPNDPGIEALRALRTSLRAALAHEGRGGG

KVLVFAGPTARVGGSFVASNLAYLFADSNASVLFVDADMRGGSHNPLGVGRNGGAIGLANVLEGGQPLEK

AIVKLGKGKLSVMTPGTLTGSNPGELFERAEFPQLLAALRTRYDFVIVDAPPILPYSDTLSIAAQDCDAL

LLVSRGRTTRASELETALQRLDSVGAKIAGHVFNAYVAPPRPPRTSLRDDWREIVARWMKPRRPARPAQA

KQTFRLAKGMAAKASRN

>WP_009940936.1 MULTISPECIES: oligosaccharide flippase family protein [Burkholderia]

MDKGMLKNVAINFFGLVLPTFVSLVTVPAYIKALGVERYGVVSLVWTLIGYFGILDLGMSMAAQNHISKA

LASGDAKESARVFWSAFWLNLATGIAGGLLIYFGAFMYTAYFTKVSAELQHEVYLALPWLALAIPLANVS

WVFAGAINGAERFGVFNTNQTIGTFLFQLMPLFAAWLVAPTLQVVLAAAVGARFIAAVMLGAASMKVLGI

RRIAPPQLGTAKGLFSFGGWMLIASTTGMIADTLDRVMLGAGLGAKYVTYYTVPQNLVTRLNMLPNALVR

TLFPRLSALGRDHADTLVKQSLEFLNGVFTPIAIAAIFALGPFLALWVGRDLAELSAPVGRVLVISVWLV

GQASVTRILIQSQVNPARAAAAGLVQMPFFVGALWFGIHHFGLIGAAVVVAARALVDYGVLLYLSAIRMR

AIVLDMVAHLAFLLASLYVAHALPGLAAAIAACAAALALNVGWSITMTPGMRALARSALMRLNPRKSV

>WP_004553586.1 S8 family serine peptidase [Burkholderia pseudomallei]

MNRTADFALSLSPLCKRLACVWPLALAAGIAHGATDWVDTHTKAFLNHAQIETLARGANAASLEVASGEA

THVVVSLKLRNAEQLKAVARNVNDPHSAQYRQYITSAQFLANYAPTEAQVKQVVAYLRKNGFVDIHVAPN

RMLVSARGTAGTVKQAFNTSLVHFEYAGRAGFANASTAQVPRALGDIVGSVLGLQNVARARPLTKIGAIA

KPLALASGTATGHYPSEFPALYNATGVPTAANATVGIITIGGVSQALSDLQQFTSANSYPDVSTQTIQTN

GSGGNYSDDQEGQGEWDLDSQSIVGAAGGQLGQLIFYMADLDASGNTGLTQAFNQAVSDNAAKVINVSLG

WCETDANADGTLSAEEQIFTQAVAQGQTFAVSSGDEGVYECNNRGYPDGSNYTVSWPASSPHVLAIGGTT

LYTTSSGAFSNETVWNEGLDGNGKLWATGGGVSTILPNPSWQSGSHRKLPDISFDAAQSTGAYIYNYGQL

QQIGGTSLSAPIFTGFWARLLSANGTGLGFPAARFYHSIPTHASLVRYDVTSGNNGYSGYGYKASTGWDY

PTGWGSINISNLNQLIQSGGFN

>WP_004552117.1 S8/S53 family peptidase [Burkholderia pseudomallei]

MQRTQHVNRLFSKRFALSPLPLAIAVALSPLLAHAAPDWVPTRTDAFLIARAPAAAASQTLAKTAPSYAL

NMIGTPELADTNVTPLELSQPLRVTIVLKSRNEAQLDSLVREVNQPGSANYRKYLTPEQFKARFAPTDAQ

VRAVVAHLKANGFGDITVSANNKLIFAQGNASNAEHGFHTTLKRFSYRGKAVYANDSAALVPASLSPVVE

SVLGLQSAAVPHRLIHRGTPADARIQTDAITKNATASGTQTGHQPTDFAQIYNASGLPAATNTTVGIITW

GDMTQTIADLNTFTRNAGLPNVNTAVVAGSSGTLADDGDPGEWDLDSQTIIGTSGGVKQLIFYAAVNGDS

NDSGLTNATLTAAYNKAVTDNVAKVINVSLGEDEAAANSDGSLAANDAVFKQAVAQGQIFSVSSGDAGVY

QWSTSPYGAPGYVGTYSGGKVTTKINLAKYSVSSPASSPYVVAVGGTTLSTSGTTTWAGETVWNEGLAYA

DVSSSGQPLDNAVRLWATGGGVSGYEAAPSWQTAALGSSVTKRVVPDVAFDAAQSTGAYLVINGQPNQLV

GGTSLASPIFVGGWARVESANGNGLGLPTSAFYQGLPGNPSLVHDVTSGNNGYNSYGYSAKSGWDDDTGF

GSLDFAKVSASSVK

>WP_004534003.1 MULTISPECIES: aspartate carbamoyltransferase catalytic subunit [Burkholderia]

MTTDTSGRTGNPAAAAPAERFRYGFLKGNPQLTKNGELKHLLTIEGLPRAILNQILDTAEQFVSVTDREV

KKVPLLRGKSVFNLFFENSTRTRTTFEIAAKRLSADVINLNINASSTSKGESLLDTINNLSAMHADLFVV

RHASSGAPYLIAEHCAPHVHVINAGDGRHAHPTQGLLDMYTIRHYKRDFTKLRVAIVGDILHSRVARSDI

HALTTLGVPEVRAIGPRTLLPGGLEQMGVRVFHNLDEGLRDVDVIIMLRLQNERMSGALLPSAQEYFKSW

GLTPERLALAAPDAIVMHPGPMNRGVEIDSQVADGPQSVILNQVTFGIAVRMAVMGIVAGTSD

>WP_004531547.1 MULTISPECIES: ABC transporter substrate-binding protein [Burkholderia]

MLEIKHKLVQTAAACALAFAACAAHAADKPLKSIGITVGSLGNPYFVTVVKGAQAQAKEINPSAKVTAVS

ADYDLNKQFTQIDNFISAHVDMILLNAADPKAIEPAVRKAQAAGIAVIAVDVAASGADATVQTNNVKAGE

LACDYLAKKLNGKGNVIIENGPQVSAVVDRVNGCKAVLAKNPGLRLLSSDQDGKGSREGGMNAMQGYLTR

FPKVDGLFAINDPQAIGSDLAAKQLRRTGIVITSVDGAPDIETALTSDTQVQASSSQDPFAMAKKAVSVG

YGIMNGHKPANAMILLEPTLITRENVKRYKGWSAH

>WP_004530042.1 type VI secretion lipoprotein TssJ [Burkholderia pseudomallei]

MRHKMGPFKSIAAALALGVLAGCSAFSSSKPEEPRQLHVTLVGGARLNVAPTGEPRPVQTCVYVVTAADW

LPTQGGDDSSCASRGQDSTVVADSRHVIAPNQLLQFSLNLPRSGELWLVADADYARRPANYGPLRVRVEG

RGLIHMAVWLDRDGIYNALLPGPVPVGGAIAPAAVRPDEPPPERKTKITYGTRRSRQ

>WP_004527787.1 2-polyprenyl-3-methyl-6-methoxy-1,4-benzoquinone monooxygenase [Burkholderia pseudomallei]

MVFDELITEFDRGLRSIAGVSRMSRPVPKPAAAAPAELSAAERKHAAGLMRVNHVGEVCAQALYQAQKLT

TSSAGLKEMFEHAAREEEDHLAWTAHRLKDLDSRPSLLNPLWYAGALAIGVVAGRLGDKMSLGFMAETER

QVESHLDGHLSELPAADVESRAIVEQMRADEVKHGKSATDAGGIELPMPARMLMRAASKVMTSTAYYL

>WP_004526239.1 serine hydrolase [Burkholderia pseudomallei]

MVLSSWSALGERVDAAIDAALAQRRLVGAVVLVARRGELAYRRAAGLADREAGVPMREDALFRFASVSKP

IVSAAAMRAVAAGKLDLDASIARWLPAFTPALAGGRPARITARQLLSHTAGLGYRFLETHAHGPYARAGV

SDGMDCAGISLAENLRRIASVPLLYEPGTSWAYSLATDVLGALIEAVCDQPLEDAVAEFVTTPLGMVDTR

FYAHDAARLAAAYVDASDAAAPGGPRRMAALEIASPFPDTAGIRFEPARALDAHAFASGGAGMVGTASDV

LALIEALRTGGDGWLPAARIDEMARIQPGAEDLPTAPGYGFGLGFSVLRDPAAARSPESVGTWRWGGAYG

HAWFVDRAAGLSVVALTNTAYEGMSGRFVADLRDAVYGAGAAAQERAA

>WP_004525971.1 dethiobiotin synthase [Burkholderia pseudomallei]

MSAPLSLFVTGTDTEIGKTFVSAALLHGFARAGLRAAAMKPVAAGAYERDGAWRNEDADQLDAAANVALP

AAIRTPFLLKAPAAPHIVAAREGVALDIGTIVDAHRRACEMADVIVVEGVGGFRVPLADTRDTADLAVAL

GLPVVLVVGVRLGCISHALLTAEAIAARGLPLAGWVANRIDPAMPFADDNVDTLRAWLEREHRAPLLGAL

AHMSPPSPDAASHALDVNLLLNALRAAAPR

>WP_004525894.1 MULTISPECIES: lytic transglycosylase domain-containing protein [Burkholderia]

MSTSLFRVYRAVAAPLVAIALAAVAAACAAQTADEASSNDDRVFVQLREAARRNDPVRAAQLAALIPNYP

APSYLEYFQIKPQLFDSAGHARLDAPDAPVLSFLSRYDGQAIADRMRNDYLLVLGARHDWRAFDEQYKRF

VLDDDTQVKCYALESRASRGENVADMARELLVEPKYYGDACVDLITALATNKQFSSDDVWAQVRLAYEQN

YTTLGGKIADALGPRPVGFDQVTSAPPLFLARGVGSDATSRQLALVAITRMARNDPEAAAGQLASLASTL

SAAEQAIGWGEIGYQATVKRLPQAASWYRKSMDAPLSNPAYEWRVRAALLAGDWPMVRRSIEQMPERLRD

DTAWIYWRGRALKESGDTLKANQEFERIAGQFNFYGQLAGEELGQKTTIPPRTKVTDAEIDAMSKVPGFA

LAQRFYALNLRLEGNREWNWPLRGMTDRQLLAAAEYGKRVDLLDRTVNTADRTTAEHDFSLRYPSPYRDI

VERYARTNGLDVEWAYGLIRQESRFITNARSTVGAGGLMQLMPATAQLVAKKLGLGTVSRAQMHDIDTNV

QLGTWYLSDIYQKFDDSAVLATAGYNAGPGRPAQWRQVLTRPVEGAIFAETIPFNETREYVKNVLSNETY

YAALFEKKPQSLKARLGFIAP

>WP_004522130.1 alpha-D-ribose 1-methylphosphonate 5-phosphate C-P-lyase PhnJ [Burkholderia pseudomallei]

MNAPDHCAAAAPDTHAAEGYNFAYLDEQTKRMIRRALLKAVAVPGYQVPFASREMPLPFGWGTGGIQVTA

ALIGRDDTLKVIDQGSDETTNAVNIRRFFARTAGVATTTKTAQATIIQTRHRVPETPLTERQILVYQVPM

PEPLFRLEPRATECAKLHAHADYGLMRVKLYEDIVHHGSIATTYDYPVIVNGRYLSSPSPIPKFDNPKLH

MNPALQLFGAGRERRIYAIAPYTPVASLDFDDHPFEVQRWDAACALCGSRESFLDEMIVDDHGTRRFVCS

DSDYCSDRRGGAQARADGGDEGNGGGDGEMPAPLAAEQPLGETT

>WP_004522020.1 MULTISPECIES: cell division protein FtsQ/DivIB [Burkholderia]

MWNNVRQLNLAASALYALLLLVLAAAGCYWLIQRPAFALREIRIDGDTEHINAPTVRAGVVGRLKGNFFT

VDLDLARVAFEQMPWVRHASVRRVWPNALAVTLEEYKPLGTWGNDQLVSVDGELFTANQGELDAELPSFD

GPEGSAKEVVARYRDFAKWFAPIHATPEEVTLSPRYAWTVKLSNGMQVELGRERNSDTLPDRIQRLVAAW

PSVTQRWGGDIEYADLRYPNGFAIRAAGMRFLTDTDKGKK

>WP_004200429.1 MULTISPECIES: lytic transglycosylase domain-containing protein [Burkholderia]

MNAWLSWRPSERHAQMLRAVLRRGTRVSHHLFSVVGCCAVAVALALWLLPNVRGTLAAKVMPFVSAAVQA

GPARLLSGHPLPTFGPANADESESVNADAAPSTTAAPADAGAMLDAARNSPSPVSLAKLIPTQRVAADAR

DDRVLASNREQALVATYLARRYRVAQEPVGQLVKAAFQTGRDVGLDPLLILAVMAIESGFNPYAESGVGA

QGLMQVMSKVHSDKFEYFGGTDAALQPVVNIQVGALVLKDCIARGGSLAGGLRLYVGATTPDDGGYGAKV

IGERDRLRDVARGRKVSIYASQQPAQGAVTVATVSTSSGATQKRVRTTLDGAHPLTIKAAAAPKAPQQDD

VSADTAASSKHETASPELGT

>WP_004195781.1 MULTISPECIES: HU family DNA-binding protein [Burkholderia]

MNKQELIDAVAAQTGASKAQTGETLDTLLEVIKKAVSKGDSVQLIGFGSFGSGKRAARTGRNPKTGETIK

IPAAKTVKFTAGKAFKDAVNKR

>WP_004195053.1 MULTISPECIES: GntP family permease [Burkholderia]

MGAVQGSMLLIYTVIAIAVLILMIARYKVYPFLVLIIVSLGLGLAVGMPMDKIVKSFEGGTGGTLGHIAI

VVGLGTMLGKMMAESGGAERIATTLIDWFGEKNIHWAMMFVAIIVGLPVFFEVGFVLLIPIAFNVAKRTG

KSLLVVGLPMVAGLSVVHGLIPPHPAALLAVQQYGADIGKTIAYGLIVGVPTAIVAGPLFALTISKFVKL

PEHNPLASQFVETRAAGETRALPSFGITLFTILLPVVLMLVGSWADLVFAPKSLPNSLLRFAGNSDVALL

IAVLVSFFTFGKLQGFNREQIQKFCGDCLAPIAGITLIVGAGGGFGGILRDSGISQQIVATATHAHLSPL

LLGWFVAALIRLATGSATVAMTTACGIVAPIAAASGVTVRPELLVLATGSGSLIFSHVNDGGFWLIKEYF

GMTVGQTFKTWTLLETIISVLGLTFTLALSAVL

>WP_004193415.1 MULTISPECIES: Rieske 2Fe-2S domain-containing protein [Burkholderia]

MTGDGDARFVCAADALVDGGEGVRLDATLRGEPAVVFFVRYAGRAYGYLNRCAHVPMELDWIEGQFFESS

GLYLMCATHGAIYEPETGKCVGGPCRGARLRAVRVDERDTPDGRAVFWLPDGDLRPAPSDH

>WP_004193385.1 MULTISPECIES: fimbrial protein [Burkholderia]

MKKALLSAVACAALSTSAFAAGTGTLNFTGEIVAGACGIDAGSVDQTVRLGFVPANTFKAAGDKSTPQNF

DIKLVDCDTSVAKNAYFTFTGTSNATQPKLIATVGSATNVGIRLQSASGEYLDNGAEQKGPVVLSNGTSV

ARFAAMYESTAASVTPGTADGVANFTVRYQ

>WP_004190377.1 MULTISPECIES: TssQ family T6SS-associated lipoprotein [Burkholderia]

MKSRSSLYIFLGFFLAGMGGCGTGATSPASTPTVAQATLDSARAAYDAGDYRRTIALLGGHAREIDGADV

NTQVAAHKLLAFSYCLTRRTTQCRAEFSRILDLNPRFDLSPAEKGHPIWGPAFEYARRKHALS

>WP_004186915.1 MULTISPECIES: HAD-IA family hydrolase [Burkholderia]

MTVKAVVFDFGGVLIDWSPEYLYRKLIPDEAERRWFLTHVCGMDWVVRQDGGQTIEEGTAERIALFPEHE

ALIRAFYARWHEMIGGELADGAALVDALDARGVPLFGLTNWSAQTFPYAWENFPVLRRFRDIVVSGRVKL

AKPDPAIYREMHARIEPHLPGIAPHELVFIDDNAKNAAAAAALGWHGIHHTSAAATRARLRELGALG

>WP_055312928.1 ribonucleotide reductase-like protein [Burkholderia pseudomallei]

MRTINKRTRTIAHADSFVRMIGDMDDQPICDAVFADRYALPGEHSRAQACARVARALALAEPMATRSGAA

RRFYRNLLNGAFGAGRIMARAGAAPDQTMASCFVHPIRAPAALTRFHPNLDEALDEARLALAMGADIGYD

FSAIPPAGARPDADHPDSPGVCAALDRFDRIGTQAGERDGRRRAQLAVLRCDHPDLLAFAAAKHRHAGRH

ARWTTLELAVAATDAFMQAVEQDLPWTLRHTAPPRDAPGGALPGADGAWTYASVPARHVWREIVSAARDG

AGPGLVFVDAIDAADPLRGRERIDATSPCGAQPLPPYGSAMLGAIDLSRFVRNPFGAGGEPRFDFAAFDT

AVRIEVRLLDNALDVTRWPLAAHARESYQKRRIGVGVTGLADMLAMMRLRYDSPAAREMARYIASDLRHH

AYAASAELAAERGAYPLCDRHAHLDALRVGPPLPHAVCHAIERDGLRHSHLTSFAPVIGVSLAFGDHCSP

GIEPARAWIEHRPVRAGAAGAPGMRAENHAHRLFRSLRGERAALPDYFATDADVAPGERLAMRVALQPYV

DAGIDNTLTLANHYSLEEVNALLFAAWRAKLKSVAIRRADLARDAQPSDDDGV

>WP_038733453.1 GMC family oxidoreductase [Burkholderia pseudomallei]

MLGATRSTLWGSRERPRIAAARAPGAAASRMREEARECAGPRGAGRAPHRATAAGGKTGRRTVRAPSRAR

AGSNNQKVNRMKQQSYDYDYVVVGSGFGGSVSALRLSEKGYRVLVIEQGRRWTPENLPESTWNLSRWQWR

PALGLRGFFSMRFFRHVVVLHGNAVGGGSITYANTLLVPPDKVWREGTWAGLEDWERVMPAHYATAKRML

GVVTNRRMDAADFRLKDMAKLIGVEKSFYPTEVGVFFGDDADAPGTRYADPYFGGAGPERTSCIGCGGCM

VGCRHGAKNTLDRNYLYLAERLGAQVREQTKVVDVRPLDARADGAAGYAVEAVSLAAGARGAKSRLTCRG

VVFAASSLGTQDLLMRLKEKGSLPRLSDALGKRVRTNAESLIGVRFPKSRVDLSKGVAIGSGIYIDEHTH

IEATRYPSGSDTMGLLTTVLTRGAPGGLRVLVWLGALAKLVLTRPLSAWRMIDPRGFARETMIFLCMQTL

EGHLTMRLKRRWFWPFSKQLATSGAKIPAYIPAANDFAQKAARALGGVPMTSLTEILLNVPMTAHCMGGA

AMARDARDGVCDGRSRVFGYRNMYVCDGSVLGANLGVNPSLTITALAEHAMSHVPAAREQRWDSTAETPV

AA

>WP_025988074.1 L-aspartate oxidase [Burkholderia pseudomallei]

MRWRARKRFCDAVCSALSATLRMAGKIRQNAQETEMQMNFDVAIVGSGLAGLSVALNLAQTRRVALIAKR

SMMEGASDYAQGGIAAVLDSADSVENHVNDTLIAGGGLCDEAATRYIVEHGREAIEWLISQGVPFTKDDA

AELGFHLTREGGHSHRRIIHAADATGHAVLATLSERARAHPNITFFEDHHAIDLITSDRLGLPGLPGRHC

VGLYALDVQTGRTMTIEAPHTVLATGGAGKVYLYTTNPDTATGDGIAMAWRAGARVANMEFIQFHPTCLF

HPYAKSFLISEAVRGEGGLLKLPDGTRFMPAHDPRAELAPRDIVARAIDFEIKKRGIDCVYLDISHQPEA

FLREHFPTIYARCAEFGIDISKQPIPVVPAAHYTCGGVVTDLAGRTDITGLYAVGETSYTGLHGANRLAS

NSLLECLVIGRATAEAIEAAGYDSATHGPLPAWDESRVADADEEVVVAHNWDELRRLMWNYVGIVRTDKR

LERAQHRLKLLRDEIHEYYANFRVSRDLLELRNLVDVASLIVDSARARHESRGLHYSRDWPHALPKALPT

VLTPPRRASK

>WP_024428974.1 molybdenum cofactor guanylyltransferase MobA [Burkholderia pseudomallei]

MPSHAAPACPITGLLLAGGRGTRMGGVDKGLQPLRGEPLALHVLRRLAPQVDALVISANRHFDAYAALGA

PFLAPIVADAHADFAGPLAGLAAGMRAARTPLVLCVPCDSPFLPDDLAARLAAALDAQHADIAFATTLDA

HGGIAPQPVFALVRTALADDLAAYLAAGERKMRAWYARHKSVEVPFGDERAFYNANSLRDLAGLERA

>WP_024428947.1 hypothetical protein [Burkholderia pseudomallei]

MSGGFPRSAFRLPRPVAAAAPVVLATLALSGCALFRAPQASAPVVEAVAVPVEPASEPIAAPEPASAPEP

AETAPPRKPHREAAPPRKPARVAPPAPAPAPAPAPLVTTRAIERSQVHALLDSEVRRGGKVIGRAVDMTA

DANGAPREMLVNLQGFMGVGDRKVSFPWKLFRFTPGGRHEPVILDMPATAQLQPADRPKAVPLTGSTQAG

AEPGQMRIIDADVERPNGAKVGRVVDVLIGRDAQPQAVVLDVGGLVDPDRRTIAANWSALRFAPKDKSLR

ALLDLNDAQLKASPPYAGDKPILAVSPAAGGAPAAAPATARAGAKR

>WP_024428664.1 glycoside hydrolase family 3 protein [Burkholderia pseudomallei]

MKRRECRGNARGKDSVKMRKKSLVHLPVALAVAAALGACSGDDDATLESRADAIVERMTTRQKVGQKLMM

AFRYWCPDGQPACTAGMTEFPDAARDALRENGIGGVILFSNNLTGIEQTRRLIDGIRAAPAADSPLGLMI

GIDEEGGNVFRLPRVEATAFAGNMALGAAYEATRDDRLAYDMGRVLAAEIAAVGFNVNFAPDVDVNSNPL

NPVINVRAFGDDPATIGLLGRRMVQGMKSERVIGTFKHFPGHGDTDTDSHYGLPVVIKSRADAYAIDLAP

YRQAIEAGEAPDMIMTAHIQYPSLDDTRVATRTGEQMIAPATMSRRIQHDILRGEFGYQGVTITDALDMK

GIAGFFDEDDAVVKVFQADVDIALMPVEFRTAADAGRLAALVDRVAAAVDSGRIDRAEFDRSVRRIVLTK

LRHGIVASDRGRPIDELASIGGPAHRAIERDIAQKSITVLRNENGALPLQAAGRRIFILTPWGEQAEAMR

RRFVELGHPLVTGAKLSAITWAEQQQAIDAADVVIVGTLSTGVTPVEHNGDPNARVSPPAPSAVRMRQAA

PANGEEEGSVIFDHVERADAAKDIGARPSVLAAIAAPSEAQQMRDAMDYAKARRKTVIHVTMRAPYDVIS

YDDVADATLATYAYYGYEGGLRGPSLPAAVDAMLGVARPVGRLPVAIHALNADGSTGPLRYARGFGLQY

>WP_024428547.1 hypothetical protein [Burkholderia pseudomallei]

MLALKLTLVPLFLLLVSIAGRRWGPSIAGWLAGLPVVAGPILFLVAVERGPAFGAHAALLSLSAIAASEA

FSFAYAWTCRRHRWPLALAAGLAAWAAAASALARLPATPPAATAVAFAATCFGQSCLPRGATLAPRAPLS

HADLAGRLAAGAALALAVTSLAGALGPAWSGLLAVFPLLGSVLAVSSHRAHGPDFVVPLLRGMVFGRFSF

AAFCLCVALTLPRQPALQAFAEAAALSVAVQGATKRLAERGPRPTALAQTATPD

>WP_024428454.1 flagellar basal body-associated protein FliL [Burkholderia pseudomallei]

MATTANPTVDKPASSGKLKRLVLFLLIGTVAAAAAAGGTYFMLSKEGAHSAAPSAPAPLAVPAFFPLEPL

TVNLLSDDGIQHYLRVGLSLKLTDPKAQEYLTQHMPELRSRILLALSNKHPEQLATLEGKHALADELKTL

IEQPTQPGNQSARVDDVLFTEFVVQ

>WP_023360548.1 type IV pilus secretin PilQ [Burkholderia pseudomallei]

MRRGLVRFLLGCALGAAGEAVASLPPLPVGAPSGWSASASVGAAGRAPLPEAAAPQWRFDSARDPVAGAP

SPDVDGGAPAAEFAGEAMPERMPAAPTAEPARSTSADAGTSSAVASAGLQAQEAALEGPPVPLAPAQRMS

DESDEHRSSPPAAGAVSTASVAGTGTETGDPSGDNRPISINLQQASLAAVFDAFARFTGLNIVVSERVRG

TVSLRLNNVRWRSAFDALLDAHGLAMARRGSVIWVAPVAELAERERQRFDAHARAAQLEPLASRGFVLRY

ARAADVQRLLAGSAGQRILSKRGSVLADPRTNLLFVTDLSGRLAQIADLIGKLDTPSRQVLIEARIVEGD

RGFSRNLGARLALRAPDAGERATGIVAGRNGTLAELAARPISGFDAATAGLTLFAARASRLLDVELSALE

AEGRGQIVSSPRVVTADRTKAVVEQGAELPYQAKVGNGVSGVQFRRATLKLEVEPQITPDGRVILDLDVA

KDSVGEETASGPAIHTKHVQTRVEVENGGTVSIGGIFESDDRDDVTRVPLLGKIPVLGALFRHRAQRAQR

SELVVYITPTVVIGP

>WP_009950267.1 alpha/beta hydrolase [Burkholderia pseudomallei]

MQGRAANRHAAFESFECSVHAPAQAARGARAVIRRAIEEQAMLAGMMLIGSAVTFGVWAGRNPLVVLNAA

ARRPTFVGRFGLTYGAGPRRALDVYLPAAREPRPAGGGAPLVVFFYGGSWQRGRRGDYRFVGEALASRGC

VVAIPDYRLYPDAVFPGFVEDAAAAVRWARDHAAALGADPRRIHVAGHSAGAQIATLLATDSRFLRAHGL

DKRDLAGVVGLAGPYDFLPLEDATLKRIFPEPVRDASQPIRFVDGREPPMLLASGLRDATVKPGNTVRFA

SRVAAAGGAVQVRLYPGIGHALLVGALGLPMRRFLPVLDDVAAFVRAAPRAPA

>WP_009928462.1 HpnL family protein [Burkholderia pseudomallei]

MQNEPQRTRSDAHTPRLIKHAGRIAALAGLAISLWLVARDDPHAVLALLRAAGIGLVVAAASHLLPMLAN

AKDWQTLIAPRCRPSLPAMLRLVWIRESVNGLLPVARIGGEFASFRLLRAHGLTAPAAVASLVSDMQLTL

ISQALFALAGVGYLLVHATSDTARVAGMFAWGLAVLAPLLLLFALVQHAKPFERAMHALNRVAGGQLASL

VERSAQIDDALKAVWRQRGTVVRYLFFWQPLQHLATSLEIWLALHFLGASVSLVDALVIEALIQALSSAA

FFVPGGLGVQEGGFVLIGGALGLDPATSLGIAGARRIRDLLIFVPGLFAWQHAEASVAASTP

>WP_004554845.1 peptidoglycan DD-metalloendopeptidase family protein [Burkholderia pseudomallei]

MGPMVWRGASDSITRWLAAPLIAAGLTGAASAAPVDMPDLQHAVRQAFSARLGKQAPAAPAVRDDLIESV

RSDPEAGWVLGTVTQVVPNDTPAYPVTKLFIARRASEGWAVGIEGTDAFYTLAAASPAKLLAGDERAHLN

AGRAPTAPPRKAVPAQTGLALPWQQGTAWYWTGGAHGWSGDSRPFNSLDFSGGNGQVLAARDGTLYKSCE

RNGSAIVKVVHDNGYTSTYYHMVQLTQAGSGTRVRQGQYLGRVGNGLPCGGQTTGPHVHFALSQGGSDVP

VNGKTIGGWQFFEGSNAYSGYAVRNQRRVSVQASLTNYGADDSGGPTEPSPPVKATVQSPGPVNLRSAPS

LSASIVGTVANGAAVQLACYAYGDTVQGNWGATRLWYRLDSNRWVSDGFVYTGSNDPVVSACAN

>WP_004554025.1 MFS transporter [Burkholderia pseudomallei]

MHAADAASPATDTVPPRVWRAVVAASIGNALEWFDLVVYGFFAITISRLFFPAGNDTVSLLLTLGTFGVS

FFMRPLGAIVLGAYADRHGRKAALTLSILLMMAGTFVIAVLPTYATIGIAAPVVLVGARLMQGFSAGGEF

GSATAFLAEHVPGRRGFFASWQVASQGLTTLLAAIFGTALNAGLTSAQMAAWGWRVPFCFGLLLGPVAYY

IRSKVDETPEFLASTPAANPLRDTFAAHKGRLAAAIGAVVLGTVATYLVLFMPTYGVKELKLAPSAAFAA

ILVVGVIQMGFAPVVGHWSDRHGRVRTMCVPALAILVLIYPAFAWLAAHPTFGALIAVQIVFAFLMTGYF

AALPGLLSEIFPVATRTTGMSLAYNVAVTVFGGFGPFIIAWLIRATGTKAAPSFYLIFAALASLAALVAL

RRRFGLR

>WP_004553722.1 flagellar biosynthetic protein FliO [Burkholderia pseudomallei]

MKPRFAPLAARAVPVCVPAFLSPRRAAERAPRGRRRAPAALAVLASGTAALPVSAADMNAVNHAASLASG

VVVGSAAPSLGVGAVLQTLVGLAVVIGLVFGCAWLARRFGLQPQRRGGALKIVASVAVGGKESATVVEIG

DTWLVLGVAPGNVRLLHTLPAGSAGVIGAPAGGGLTRSPGAPGGTAMSGPLPEGASFGARFRDAMLGEAA

KRFKRDGGKDR

>WP_004553259.1 peptidase [Burkholderia pseudomallei]

MAKRIPIGTTVAALATVTLVACGGDDPSSGAVSAGNSPAIVPSVPAATPATPTATAAESEQDVFARTAPA

DKVLDDSNVYDTTKDGSIALSKVNEDSSVKRHTITINGKVLPYVARAGHLVAYRQNGAGKKAEAAIFYTA

YTRDALPKEHRPVTFLWNGGPGSASIWLHMGSWGPKRLKSDAPNMADPTKQPDSFPFEDNAISLLDQSDI

VFVDPPGTGLSTAIAPLKNGDLWGTDDDAQVVADFITSYTNKYNRQSSPKYLYGESYGGIRTPIVANLLE

QAGTSGYVPDPSGKPARVLDGFILNSPLVDYNSNCDMMGGRVTCEGYIPSYAMTADYFKKSVKRGTRTQE

QYLGELRTLARTTFQTTYGTYFTNGKPNSQWNAYAASTAGQALLNRIADYTGIPASTWNGSFNYTPRPFR

NALVPGYELGRYDARMKVPNGNSFAADSYIDVAFLNQLKTYFPDFVNYKTQSIYEPLNNATIRNWKWKRA

GSKYDYPQSITDIQAVLTANPDAKLLILHGYEDIATPGFQTELDLEGVNLSDRIPVKWFEGGHMIYNTEA

SRVPLKQAIDSYYKSPTRIADGSLL

>WP_004553203.1 SRPBCC family protein [Burkholderia pseudomallei]

MITSTMRRDTASRLVKASPSAVYAAFVASEAVAQWLPPEGAMMEIQVFEPRVGGRFQMTLIFASAPGKST

ANTDVVVGRFVERVPQQRIVQAFEFDSPDPVFAGAMTMRWELEAAAGGTAVTVVAENVPPGISQTDHETG

MNSSLTQLAAYVESHD

>WP_004552047.1 RES family NAD+ phosphorylase [Burkholderia pseudomallei]

MGVTSPERQTNWPTASLDWAPAYRVIPTRFPAINLFDRVASAEDFDALYALEALTNDRVRTEVGMLDLVP

PQERRFGPGYGPIMAAFTHLNPNGSRFSDGSYGVFYCARARNTAIAETRYHTSLFLAATKEPPMRQQMRL

YTVAAQGEVADVRAWRERDPALLDLVDYSAGQALGRAVRDAGGAGIAYPSVRDPGGECLAAFRTTLLHDC

RHAAYLEYNWNGAAIDAVFELNQVG

>WP_004550971.1 TraB/GumN family protein [Burkholderia pseudomallei]

MPEAVAAREAARRSRNGVRRAATHAARAPRRWRARSLASAVLAGACATGGLAWPPAGALAAGSVAAAPLP

QAPIPAPGMSLPGFHAPPPSTSNGTVASGAVRTQPARMPFYVATKGKVTIYVLGTLHVGDPADYPANQPF

RRPILAALAASPTLALELSPDDLLESQDDVSKYGVCNYACLPRLLPPPLWQKLANRLRGNPAALAGIRNM

RPWLASLVVETYDSLSAGLQTEYGTEAQLQNVFLRKKGGKVVGLETLAEQMRAFTGLTLAQQREMLAQDM

VQTPAQNAADVRALHRLWRIGDADAIAAWANAKTERLARARSIADSIDNKIVYERNRRFVARMTAIAAPN

RPLFVAIGSLHLGGPKGVLELLRQQGYRVDAG

>WP_004550328.1 SCO family protein [Burkholderia pseudomallei]

MNETSMMRRKALGALCSLALAGAPVGSRAATPFYSLPLSSKDWHKGFRLTDLHGHTKTPQDYRGNVLLLF

FGFLSCPSICSTTMLELTQAKERVGAQKDKVKILFVTLDPNRDTSPTIARWLASFGDDNIGLRDSEAHVR

KAATALNLKYERVEGDVPGAYTIDHGVQTYVFDPQGRLRLIARAGIEPEYVAKDIVQLLSGR

>WP_004548868.1 acyl-CoA desaturase [Burkholderia pseudomallei]

MSSGQPPSVNEGASQPEGAIGSARPASLSSGSHGFARPPTILPRSAVRLDRRIAAVVTLLPALGTLAAIA

LWIGGHGPGAVEWIVFALFYFATALGLEVGFHRHVTHKAFKAKPWVRTALIALGSMGAHGPVNWWASTHR

RHHSTSDGDGDPHSPHLSGEGFGGRLRGLYHSHMGWLFVGESTRPAGWEKYVPDLYQDPLVFRQHMAYYR

WVAIGLVLPPLVCGLASRSWTGVLLGFLWGDMARIFAVSHCIWALNSLCHVIGRRDFHTTAHDRSRNSLL

LAIPTFGQGWHNNHHAFPASAFTGLHWWQIDPGGLLVRVLERLHLVYDVHRPNAELIEKKRIAP

>WP_004548243.1 tRNA adenosine(34) deaminase TadA [Burkholderia pseudomallei]

MRLAGAPASGGELDDSTQSPPHAALLSAAAERDRRYMRLALAAAEEARAAGEVPVGAVIVRGDEVIARGF

NHPIGGHDPSAHAEMAALRAAARALRNYRMPGCELYVTLEPCLMCSGAIMHARIARVVFGAPDPKTGACG

SVVDAFADARLNHHTTVEGGVLADECGAVLKSFFAQRRQAVREARRAQQAPGAPPRDE

>WP_004547320.1 branched-chain amino acid ABC transporter substrate-binding protein [Burkholderia pseudomallei]

MSIHAESDWASEPIDSFEAARQSKIVTFSRARVDMNVRMGCLSLLAAVAFDAHAQSSVVRIGVAMPLTGP

VAHLGKDVQNGAQLAVDELNRAPPTIDGKPVKFALVVEDDQGDPRQAVQVAQRLVDARVAGVVGDLNSGP

TIVAAKVYAAAGIAQIAPAATHPAYTQLGYKTAFRLMATDNQQGASLAGLAAKLAKGRPIALIDDRGAYG

QGLIDQTEKTLRASGVTRIIRDYTTDTAVNFASILTRVKGAHAAVIVYGGADAQAGPMVRQMKALGIDAA

FVGSDGVCTGQWTALSSGANEGQFCTQAGDPRARMAGYAAFERRFEARYGKVIVFAPYGYDAVMLLADAM

RRANSTEPAALLGALATTRYDGVIGRIRFSPQGDNLNGAVTVYRVQRGALVPVSD

>WP_004547060.1 sulfate ABC transporter permease subunit CysW [Burkholderia pseudomallei]

MSRDTARSRAGAARADAGPEFGARFGLESGLESGDDARPRARAPARARRLDPVSEPRAVRWLLTGAALAF

LALFLVVPLAAVFFEALRKGVDFYLESLADPDAWSAIKLTLVVAAIAVPLNLVFGVCASWAIAKFEFKGK

AVLTTLIDLPFSVSPVISGLVYVLLFGAQGWLGPWLQAHDVQIIFAVPGIVLATIFVTFPFVARELIPLM

QAQGADEEEAARVLGASGWQIFRRVTLPNVKWGLLYGVILCNARAMGEFGAVSVVSGHIRGQTDTMPLHV

EILYNEYNFAAAFAVASVLALLALVTLALKLFAERRLSAELAHGRDDASAPAAHPGAAVTSSIS

>WP_004546872.1 DUF3472 domain-containing protein [Burkholderia pseudomallei]

MRRSELKFNCIAATILAAVAADATAAGACLNGSTIASTTRAPLVARQGSVFSSTLYDPAITSNNRTHNPV

MLTVKVTNNGRPVAGCDVAWQPRGAGGASGWLFPASASTDANGIASAWWVAGSGAAQTAVASIRRFDGTT

QGVAIGGSAQPHATRANSIHLNYEPASDWTAFRVDVTPEALAPTTYWEAIGWPGAYTGIQSIDGKQDGLV

LFSVWDVNGKSPQIIAKGPGVDCTQFGGEGTGYKCAKRHAPVAGRTYRFMASIAPVAGQNQTDYSVWFTD

TSTNARELIATLRYQKAVQSANYANSFVEDWATQGASCLGATQRAGQYGNVWALDRASAQWRTVKRASTS

AVYTPDHNEVCSNYQFSVVNGRFRMSTGGHAVGQPLNLPNGPKSFPLTLP

>WP_004544472.1 haloacid dehalogenase type II [Burkholderia pseudomallei]

MIVERNVMPGTPPAFPKAILFDAYGTLFDVHAVVAAAEQLFPGHGDALSQLWRRKQIEYSQLRTLADPAG

GRYRPFWELTLDALRFAARTLGLALSAAAEKRLMDEYACLSTYPDTVPTLRALRARSTPPKLAILSNGNP

QMLDIAVKSAGMSGLFDRVLSVDAVRAYKPSPAAYALGTAAFDAAPADIAFVSSNGWDVAGAGWFGYRTF

WLNRTGAPLEELGAAPAGTGAGMAELLAFLDAPRAAAKAGGRARPAPRVSRLGAPPT

>WP_004540623.1 amylo-alpha-1,6-glucosidase [Burkholderia pseudomallei]

MPRQADPASPRAPQAAAPASSSARCAAPGAAPPQSAQPPQSPQPAPSTPAFIAPEPDAQKAVRGSQFVLK

AGDAFVVSDALGDIAGRDDGLFVDDMRVLSQWRLTFGGRAPSLLSGATSADNASFTAHLTNRPLPPLGGR

ETPEGVIHIERMRVLADNVLHEALTLTNYGTCDAEVPLSVSFGADFKDMFEVRGSRRERRGAVAPPCVEA

GAVRLRYDGLDAVERSVRIGFEPKPDTLAVDRADYTLTIAAQACVSLYLTVEARVGAAHAGGDAFARRPC

VATGRGALRKALADVHRAMRERRRTIARVHTSNPLFNAWLDRSLADLGLLTTSLETGPYPYAGIPWFSTP

FGRDAVITSLQMLWLQPSLARGVLRFLAEHQARETSAFRDAEPGKIMHEFRKSEMAATGEVPFALYYGGV

DTTPLFVVLAGAYLEHTGDESLIDELWPALERAAQWVSTVCERNPRGLLDYQRTSARGLANQGWKDSQDS

VFHADGRFPSGPIALVEVQAYACAAFDAMATFSRRRGHAADTVRYAQRAKRLREQVEALFWMPEAGFYGI

AVDGHGELCRVLASNAAHLLAFGLPEQSRGESVARVLGSTLFRTGWGVRTLAAGQPRFNPMAYHNGSIWP

HDNALAARGLARYGDKRAVLDLLRALFEAAVSFDMRLPELFCGFPRRRGEPPTAYPVACLPQAWAAGAPF

MMLQACLGISVDAARGEVRVERPELPEGVDWLRVDDLRVGGDSVSLTFRRVEGQVVAAAEPGGARVVAVL

>WP_004539393.1 peptidoglycan DD-metalloendopeptidase family protein [Burkholderia pseudomallei]

MAAVRDALTGRGRFIGLGAARGGRFATRGLALVPHRAAGDMTCLPLARLVPTGFGPDAVESVRRGPTLSG

RSRTKVPAAVSMPPPRSELMEVRLRSILFVQRAGIGRTVRAALVAAGAALVGGCTVTPWTDSWQPTHVPS

QPAPRASSGVPAGYYRVNSGDTLASIASAFGQRTLDIASWNHMAPTDMVMPGQVLRVAPPPSTATFAPPP

AAEPQPEAAASALAWPAHGTVTTPFGAGRNHGIVITSTGGDRTVRAAAPGRVVYAGTGVAAYGPLVILKH

ENGLITAYGHNEKLLVNEGDAVSAGQPVAEMATDASGRSTFEFEVRRNGKAVDPLGLLPRNGS

>WP_004539390.1 DUF938 domain-containing protein [Burkholderia pseudomallei]

MRSATRTARLTSDSIFPIHFVMPIDSASRQWAPAAERNREPILAVLKRVLPARGAVLEIASGTGQHAVHF

AAALPDLVWQPTDVDAAARESIAAWAADAALPNLRAPLALDVCVEPWPLAAADAIVCVNMIHIAPWAAVC

ALFVGAARALPDGGVLYLYGPYRRGGAHTTESNAQFDAQLRRRNPAWGVRDLEAVVELGGASGLALDEVV

EMPANNLSVVFRKRA

>WP_004538844.1 biotin carboxyl carrier domain-containing protein [Burkholderia pseudomallei]

MAQNEIVSPLPGTFYRRPSPDAAPFVDVGSTLAPGAVVGIVEVMKQFTEIETAAAGRVVEILVDDGEPVD

AGQVLMRTEE

>WP_004538739.1 helix-turn-helix transcriptional regulator [Burkholderia pseudomallei]

MARRRDMSDDACPVARAVDAVGDRWSLLIVRDAFDGVRRFSEFQRNLGIARNMLADRLKTLVEAGVLAAA

PASDGSAHHEYVLTDKGRALFPVVVALRQWGESQLFGAGEPHSRLVERRSGLPVAPMSPRTQSDRALRPE

DAVVDKRGADDAAT

>WP_004538449.1 EAL domain-containing protein [Burkholderia pseudomallei]

MPKTQGFSPESLISQFTRCDAGWCVRYHDVTLASAFQPVISITHKRVVGYEALARATDANGAPISPDTLF

ARAQARGETILLDRLTRCLHVANFAAQDTGACWLFLNVLPQMFDAGIAPGAFIEALCAHFALPPTRVVLE

VIEQPSRNEVALARTIDMIQHGDFLIAVDDFGTGFSNFDRIWQIKPDIVKLDRSILERSLAASDAHRIIH

HLVTMLHHAGTMVLAEGVENEDALQILMDADVDFVQGFCFGRPAPSLEHARRAAPARIEAAWRRFAERTK

ARCGDVVHPGFDTIERIMLTGAADYAQTRNLRDAAQRLLTSPIVRRVFVAEANGEQVEPSVTIETPDAPS

ATARRLAPLLPELHCNWSRRAYFQRAIAAPGRVALMGPHFSLTDGRDCYTAAVAIHLGTTLKVFCVDFDF

SSYGDGR

>WP_004537427.1 MFS transporter [Burkholderia pseudomallei]

MSKSTMPAGGVAIRLGLKENWRQFALLVLINAFVGGMVGIERTVVPLIGSETFHIQSTTLITSFIVSFGL

VKAVANLISGQLADTWGRKRVLVAGWLLGLPVPFMIIAAPNWEWVIAANVLLGLSQGFAWSMTVIMKVDL

VGPKARGLAVGLNEFAGYFAVGLTAFLTGYLASRHGLRPAPIYLGVAYAIAGLTLSILVVRDTRDHVCLE

AGKPKEATSLSFHDVFLLASLKDRNLFAASQAGLINNLNDGMSWGIFPLFFTGLGLGVERIGILKAAYPI

VWGVFQVVTGPLSDRWGRKGLIVAGMWVQAAGLVLTASMGEFRWWLVASVLLGLGTAMVYPSLIAAVSDA

SDPRWRARALSVYRFWRDLGYAIGALSAGLIADRFGFADAILSIAAVTFLSGAVVAIVMHARH

>WP_004534690.1 alpha/beta fold hydrolase [Burkholderia pseudomallei]

MHVSTDTSAEAHSPAPNSLDMSDSDALRRIAEAVDGGAANIERIVPLARARERMPTRPRLERCGGGRVTA

AHITLDSRARLDALLHALQRAIDQNADLRTCILGACLRRPMQVTLREVRLRVHAATLDPDLDPAAQLAAL

STGPGMRIDMQRPPWVLACIARIPGSGQWLLRLVAAPIAAGFDALDALLRETVIHGDREPGPAPFHWTVE

TAVESCGGEPASLPTAGAVWPSNDVSRACDPDAASCVEARIAAIASDLPGVVHGGPRDDLRALGRTPLQA

LRLARRIRDALGVTVPVESILASPTIVELAGYVEQLRSRDVRDGAAPVSIGEKPADADARAQAQADTDTA

HTDCLIVIQAGGAEQAPVFCIPGAGGSVASFVALASMLRADIPVYGLQPRGLDGLGPPDRSVEAAARRYA

RAILDAAPPGPPRIVGHSFGGWIALETARLLDGMGARCAPLVLLDSNPPPASQAWRAPSEADMLRTLVGL

LEQAAGGAPSGIGDEEIARCAAAGEDARDALVHACMVRTALLPPRAPVEAVRHLRRVFEAHSSTRYAPGG

RYAGDATVIVANGDRDAGEMVPAFGWAALIERVEVAVTPGNHMSMLAAPYVRHVALTMKTVWRMI

>WP_004534251.1 molybdopterin-dependent oxidoreductase [Burkholderia pseudomallei]

MRISTNWIKHLVLALLLGVTLSSYAAPFKFTVDGNIKRTNQPGRRAYVFSEAALMALPQHSITTSTSWTP

KATFTGPRLSDILKAVGATGTQIEFHCVDEYTFTIPTSDADKYGVILARTMNGKVLDNNNYGPLWIMYPR

DQFPDELKTPISEAKFAWQIIGLTVK

>WP_004534156.1 ATP-binding cassette domain-containing protein [Burkholderia pseudomallei]

MSPVPFPAGEAGEALEGAQTAEAVEAAEAMEGGAAVARGATPGRAASALAARRIDVKAGDFTFHATDIAF

RTGALTAIVGPNGSGKSTLLEALFGFRRAQLEGATILGVPAARFMRDTRALRRFGAQLQRVEYAEHARVD

EILAVHRALYRQQDAAVARALAIDELRAKPYSGLSKGQRQRLDLFIAFAHRPALVALDEPFTGLDRTMTR

SVLGLLRGPLAGITIAMICHAGEELAIADDVLWVRDGTLRYQGGKDALKRRLVGEFRALIHVDDDAQAER

VRAVLARDAHVQRIVAPAPRQIGAFGRAGLDAALRTLMEQAGIRHFEFAPTDEGDLLRACTEGATDA

>WP_004532930.1 NAAT family transporter [Burkholderia pseudomallei]

MIVDRLISEILFGFTGLMGIINPFGIAFLFLERTESLTEAQRAALARKVAINAFIVLLAAFFVGTPILHF

FGISLEALRIGGGLAVAVAGWQMLNEPDVPTGGDTAAQPVEPRNAMARAFFPLTIPLTTGPGSIATAIAL

NANRTHKLSEFVLSSIVSVAVSALAALVIWQTYSRAAFLARYLGTEGTKVAMRVSAFLLLCVGVQIMLTG

FSEFLRPIADQVK

>WP_004532120.1 adenylyl-sulfate kinase [Burkholderia pseudomallei]

MNMKGGFPPEAVAPVIWMTGLPGAGKTTTANALAERLLEDGAKAIVLDGDALRSGLCADLGFSDADRMEN

IRRFAHVAKLFQREGYVVIVATISPLQAHRDLARSIVGEGFFETYVATPFDVCRSRDPKGMYARAEQGRL

MQFTGVSGVYEPPVAPDISIDTTEHSVAFSLAEVMAQLARVSALPAPLDAAASAPVAASAPAAAGG

>WP_004531693.1 hypothetical protein [Burkholderia pseudomallei]

MQKGINMKRSRSRFRASFAVAVALGVPSGFAHAQSLPGIPDPAAVGLTPGTGQNGAPTVSACFYNYRTKV

IAGFPTQIASGWIVTNQRNDPACIPPGGLPGPNWQEMTYFRDMPVGASMSACWSFSWPSNWQPVGYTSDP

SKCGYYPGGQIPGAQPNVVFLKRVQ

>WP_004531413.1 hypothetical protein [Burkholderia pseudomallei]

MSHRTRPFKAGSHEAVYPGLAQDPYAMRRKLPEPTVCPGCGAVFSAGRWQWIARPDGARERQCGACRRSA

EKMPAGYLHIDGPFAAEHWTELLQLLRSREEQARTAHPMQRIMSIDTDGGATVITTTDVHLARNLGSALK

SAYRGSLDLKYSPDAQLVRAHWRR

>WP_004531184.1 NUDIX hydrolase [Burkholderia pseudomallei]

MSAMKPPGRTRARTVSCGVVLLDSDGRVLLAHATDTTHWDIPKGQGEPGETAQQAALRELAEETGIVLDP

ARLVDLGLFAYRRDKDLHLFAARAAAGETDLSRCTCTSMFPSRRDGTMIPEMDAFRWTAPADVDAYASRS

LARLFGTTLSLAALHRTLAG

>WP_004530912.1 Tim44 domain-containing protein [Burkholderia pseudomallei]

MSVSRSSFNRSREPKSLARRIGTFLMVGLLAAGTFASLDAEAKRMGGGRSVGRQSQSLQQRQATPPAQQP

MQQAAPTQAPRQQPTPAAQPNRSRWLGPIAGLAAGLGIAALLSHLGLGGAFAGMMANVIVIALLAMAGIW

LVRKFMNRRGAQQPAYAGGQPTLNRGHDGYSQDAGVQRQAAPGGAFPGSGSAYAGEAQRVFGGAAATTAA

AAAPAVPAGFDTEAFLRNAKVYFVRLQAAWDEGNLADIREFTTPEMFAEVKIDLDGRGAGANQTDVVQLD

AELLAVEDRGGEHLASVRFHGLIRETQGAAAEPFDEVWNLSKSGSQGWLLAGIQQASTH

>WP_004530759.1 alginate export family protein [Burkholderia pseudomallei]

MIARRAVALGGWALMSVLAAARAHAADAMDAPAAAAAAAAPGSRFARVSATSESAPSSAFEFASPAASAS

TSASALAVVPMSALTLASAPAPAPAPSAAPSPSPTSSPVQPGVRPGASCAKTRPAILFNRWQEDWSALAD

PCVPRRPLDALKYVPLFGRVDSYLSLGAGLRERLELNDAPLFGLGRARGDTYVLQRVQMHADLRIAGHVQ

AFVQLEDARPFGKDNVGPVDRNRVDLRQAFVTYVDAIGSGAFKARVGRQEMAFDLQRFVSVRDGPNVRQA

FDGIWADWEQGPWRLIGYATQPVQYRDDGAFDDVSNRNLTFSGVRIERQRVGPGDLSAYYSRYNRTQAQF

PDGAGGEHRDVFDVRYAGKRRNVDWDIEGMYQTGRVGAQRIEAWAVGSLAGYTFAGVGWMPRIGLQVDAA

SGDRRPRDGRIETFNPLFPNGYYFALAGYTGYTNLIHVKPSLTLKPSSALTLLAAVGLQWRATTADAVYA

QGATPVPGTAGRGGNWTGFYTQLRADWAVTANLAAALEVVHFQIGDALRAAGGRNADYVGAELKFGW

>WP_004530728.1 energy transducer TonB [Burkholderia pseudomallei]

MQVSNSLPGASAPAFSRMNPRIVTAAVTVLAGHALLLTGALLMRNDVPHRPLESKTITAQLLSAPVAQPA

GIQSAPAPTPPQPVPKAKPKPAPTPVPRPVAKPSPTPLPVTHEPAPNAITAPEPAPAAPVAPAETSAKAA

PPAGAPTNRPTMEIVAPKEGAHLSCQIAKAAYPSMSKRRGETGVVKVRFVVGLTGKIESAQVVQSSGFAR

LDDAALDAIRSSPCQPYLQNGQPMRAAYTQPYDFTLTD

>WP_004530469.1 branched-chain amino acid ABC transporter permease [Burkholderia pseudomallei]

MQRKALYGLLLLGLLVAPFVGAYPVFVMKVLCFALFAAAFNLLIGFTGLLSFGHAMFLATAGYVTGYAMQ

SLGTAPELGVLAGTAAATLLGLVVGLFAIRRQGIYFAMITLAFAQMVYFIYLQAPFTHGEDGLQGVPRGR

LFGLVDLSSDLALYYVVLVVIACACFFIVRIVHSPFGQVLVAIKENEPRAISLGYDTDRFKLLAFILSAT

LAGLAGALKVVVLGFETLSDAYWTMSGLVVLMTLVGGMGTLFGPLVGAALIVALEDRLGDIGGWLASVTH

IDWFRSLGESATIVTGLIFIVCVLAFRRGIVGEVIARVRPLRAS

>WP_004530335.1 hypothetical protein [Burkholderia pseudomallei]

MLPAFSMDVGRARSACAALHGAAGWLACLLPVAVAHAPAFWAWNALAAAACAAAGAGQARASVHSRRSPL

AGVAVLSGAQLADFASTYPDAVLALCSSASFVDDWVLHVRLFPFTAAAMVGLSVAAARGERGRRTRWPKR

IVDAAVALGAMMFAMNLAAMLLKTWAGLAGWPWGAHALVCTMLVGMMLDHAAMRTMSDAIAAQRASHAIE

>WP_004530026.1 multidrug effflux MFS transporter [Burkholderia pseudomallei]

MNTTETNTLSDRGWLSLLMLVVCLPRMTIDAYLPSLPAMADALHGTDAQMQLTLTMYMAGYALSMLLSGP

LSDRYGRRPVLLAGMLVYLGASVACATATSVQGIVVARIGQALGGCCGTVIGRVIVRERFGTAMQAAMLS

RISAGMALSPVVAPLAGHVVAQWLGWRGVFASLAFGGAAAALMVHRFLPETRVRAAASERGAGLVKTYLS

LLRDRRFVRYSLAIGFVYCTYFPFIAESSALFQRTLRASGAVYAAIFGITVLGYLIGSSVFRRASGRFGI

DAIIASAAFVNLVGAAALWIGTSVAPPSVWSIALPMFVVMVSVGVSIPACQFAVLQPFAKLAGTASGLFF

FIQMAMTAGCGGMLSRLSDGSARPMAVVTAASSAAFMAVVMLVRTRGVNGRRG

>WP_004529975.1 copper homeostasis periplasmic binding protein CopC [Burkholderia pseudomallei]

METMKRIAHPKRAGLAALVAACAAIASAAAFAHGKLESAVPAPGGTVDTAPDALRLTFNESLESAFSTVK

IADAGGAPVAAPKAKVDAANPRVLTLAVPKLAPGAYTVQWTVMTADAHKTKGSYAFKVK

>WP_004529767.1 DUF4087 domain-containing protein [Burkholderia pseudomallei]

MIAFLARRAARRTPTPTTASHSPISRSRGAGRHLFAALAVALIGLPHAHAEIRCGWLQNPTPGNWWLDDR

AGSWTLGTMGGPETEGMDVIPDMAGKQYVETNGSHGYACACLTVVTDKQERRIVKVLKAKQLPLSRCRRD

KRLKEPT

>WP_004529638.1 DotU family type IV/VI secretion system protein [Burkholderia pseudomallei]

MSIEMPAQPLRMLLQDTMLHVALLTQGARISRVHDWRARCIALVRQFEQALQDGGYRGGIANELGLAQCV

FLDEITMRNLSTEQKEEWLRELLQFRFHSIRDGLSKVRARIDRLCRGHSANAALLEMYSLFHEFDLLGDK

QGHALADARVSEARPTPGKKAVETAEVWKDGGAANEAAHGRSWRIVSGLLAFAALMTMWLVIDTRLSHEV

EQMEVTSGDEAAIHPASGD

>WP_004529636.1 fimbrial biogenesis outer membrane usher protein [Burkholderia pseudomallei]

MMIAAGAVACGVGSLCMPVVAAEFDGTRTGEQSRPDAMGEAPSGAEPGTRGRQALLVASHASPSAPTTVS

FNSRLLMGGGIDLSRFERGNPVVAGIYPVDVTVNGERRGRMDVEFRDVRGGDSAAPCFTRAMLDRLGVEG

DIVAKRLDAARDATGAASGQTPTLSASACIGLRDALPDATYTLDSADLTLDLTIPQVDMRKTARGYVDPS

RWDNGVNAGLLQYNLSGYASENKFFGNHASSLFLGLQAGANIGAWRVRQRSNLMWANRSAGMSWRSLETY

VQRDITALRSQITLGDSYTTGEIFESFGVRGMQLASDDRMLPVSLQSYAPTVRGVADTNARVAVRQRGNV

IYEASVPPGPFEFDDLPPTGYGGDLDVTITESDGRTKHFTVPFASVRQLLRPGMQRFNFTVGQYRDALSN

GKPWVAQLTYQRGMTNLLTGYAGLLSSTGYASGLIGVALNTPIGAFAFDVTSARTHLPGQGARNGFSSHL

SYSKMVPSTGTHFSMAAYRYSTANYYSLADAVTARYGYNAEERAWRNDYRARTRLQLNVNQRIGDRSSAY

VSSSLLSYWDGRGRDIQFQAGFSSVFKRVSYTVYAQRSRSSDDRTVTQVGVNLSIPLGSGAYTKRNAFSS

LTTSLSRASNGDSSIQANLSGSTAHVVPIDYGVNVSRSVTGDSNFASLGVYGTYRSPFGTYSGNASVDNR

ARQASFGANGAVVLHRGGVTLSPPLGSAAALVEAKGARGGKLINGQGATIDRFGYAVIPSLMPYRANTVA

IDPSELPDDVELANTSEEVVPRNNSIVFVKMETKRGRPVFAATETEDGKPLPMGSELFDVEGKSLGGVGQ

GGMAFLRGLQGAGNVVAKWGAGASEQCTMPYLVPADQAQAKQSRAIVRIRLRCEPRLSADASQTPDGDGG

TRND

>WP_004529402.1 DUF2029 domain-containing protein [Burkholderia pseudomallei]

MSRSGGWLTSDRVLAYGGATLILSIALLGAWGWTTSGFTANTTVRPGIDFTVFWSGSHVMLHGAPASVYD

YPAFSRAEAAQLGAYVNRSFLPWVYPPTLLVLVTPLALLRYLPSLLLFCALGLLAYAKSVGALTGLRDRL

AKPRLASFVILSFPGVLVAAVIGQNSLLTAACAAFATRLLAKRPALAGLCISLLAVKPQMALLFPLLLVA

TRAWRACFAAAAGTLVFAAASVAMCGTASVHAFLSGTTMLRELVLEQGTQYWLASPAPFSAMRLVGAPLR

VAYAVQIGVGALAAAAALDVWLRTRDTRLRAAALAVATLLATPYLWHYELAWLGIALFGVLACALDTGWL

PGEQPVFVAGWLLPFFEFFNRATHFPQIGPVVLLAVLLVVMRRARTMPGVAR

>WP_004529129.1 GNAT family N-acetyltransferase [Burkholderia pseudomallei]

MFVAPAARRPGVGAALLDAALAAAARTAGQVALAAVDGRGAARAFCERHGFVAYGVEPRALDAANGCADA

LPMVKFPPLAPPHA

>WP_004528126.1 metal-dependent hydrolase [Burkholderia pseudomallei]

MPAIVRRDVRFALPPDRIGDWHVNGVANTHYFNALSLMFPAGERFFIDAVRHYRDRIQDPELLRQVQGFI

GQEAMHSREHVEFNDVAEAAGYPAHRLDRGFWRFTGLMQKILPPPLRLAQTIAFEHYTAIMTDMLLGNFE

RFHDSADAYANMWLWHSMEETEHKAVAFDVWNAVMKPGPARYLMRTGSMLLTSVVFWGTNFYFHLAFMNA

HRRMHGKVTGKWAFLRFFLKGMVRIAPKTLAYFKPGFHPWQHDNRRHFAQLDQLLANIDASNARYAAQAA

PRRIPLHPMTIQPS

>WP_004527801.1 AraC family transcriptional regulator [Burkholderia pseudomallei]

MPAYRRLHAPHPRLRDIVNCYFVELGRAGPMLYPATPSASITVFASGASVHTNGIAHAETLMCRPQRSAV

WATLLPGTTYVSVVFRPGQIRRVMPRGASAAYDDGSPLDAVFPRAAVHALVDGVRGAPTLGHAVAALEDW

LMAQAHVAEEFDAGGILLPPACVDAPREALTAQFSRSPRQLERLFIDTFGASQREMRSLLRYARTLSRLI

AADFALPALSDMAIDCGYYDHAQMTRAFRRYAGMAPVQLAAAVRDARQASMTMYRYELADKRILLDA

>WP_004527799.1 hypothetical protein [Burkholderia pseudomallei]

MRGGLPASEVSAPLAACDSNRSSMYFCAWRDRLVVERERQRGVDRQASAHPAISTARYRPFHSRSLRARL

HALALRYGMSAGGTAALPEYRSS

>WP_004527612.1 helix-turn-helix transcriptional regulator [Burkholderia pseudomallei]

MTTMRKNAPEGEQITDRKPSLLDAPRKGNVYASECPTRLVLDRIADKWTVLILALLSHEPLRFNTLLRHI

EGLSQKVLSQTLKRLERDGLVARTVYATVPVSVEYALTPLGHTLAHSITPIIVWSETHIDAVLDARTAYD

ARQGGRAPE

>WP_004527607.1 hypothetical protein [Burkholderia pseudomallei]

MPDKHNHRADAEGLSLGSLRTAAWLRYLLDHAERFAAAAASSREARVAAAARDTLAKAGAHARREPAADA

GLLLNELLAALEGDGASVDEARGNAYGELARTPPKRLLAAVDRYLPGSKAVFDGAHGVADVLGYDDAELG

WAALDSIELGIGRALASDGKAADDAMRGIAAFEGPAAGRVIDHAIGRMWGADEKRADALSALAAFSATLA

MNAPITLDAYGGEHNGVAAAILRERGDLASAPGLLAALAVCRLDRLTGAGSFWAYYLLAGVMIAAPQAVA

SIAARFHRDALSAWLDMLLAVPARSPIGDAGEAALARLAASMSFTSRHNPIVHTRHRSMG

>WP_004527460.1 excinuclease ABC subunit UvrC [Burkholderia pseudomallei]

MTSPDAPESRFEPKPILAQLPHLPGVYRYYDAQDAVLYVGKARDLKKRVSSYFTKTQLSPRIAMMITRIA

RIETTVTRSEAEALLLENNLIKALAPRYNILFRDDKSYPYLKLTGHRFPRMAYYRGAVDKKNQYFGPFPS

AWAVRESIQILQRVFQLRTCEDSVFNNRTRPCLLHQIGRCSAPCVGAIGEEDYARDVDNASRFLLGRQGE

VMGELERKMHAFAAELKFEQAAAVRNQMSSLAKVLHQQAIDVGGDSDVDILAVVAQGGRVCVNLAMVRGG

RHLGDKAYFPAHVETALALAGDIEALAGEGAGDGVQAAAQPAQAPLATDADATDAAATEAKTVTAAAAAR

AGARTAQAAGARAAASAEGDVERRAEGETHARADAREAAALPDGAAAAQEADADVDAAPLETEVLEAFIA

QHYLGNRVPPVLVVSHAPANRELIDLLVEQAGHKVAVVRQPQGQKRAWLTMAEQNARLALARLLSEQGSQ

QARTRSLADVLGYESDDLAQLRIECFDISHTMGEATQASCVVYHHHRMQSSEYRRYNIAGITPGDDYAAM

RQVLTRRYEKMVEEAAAEASADEAAGIDGNAVHAAASAGRLPNVVLIDGGRGQVEIARQVFSELGLDISM

LVGVAKGEGRKVGLETLIFADGRAPLELGKESAALMLVAQIRDEAHRFAITGMRAKRAKTRQTSRLEELE

GVGAKRRQRLLARFGGLRGVVAASVDELASVEGISRALAEQIYRQLH

>WP_004526999.1 pyridoxal-phosphate dependent enzyme [Burkholderia pseudomallei]

MNDIMTQTFDRPAIPVYDSVSGSLDHPDLIRLAPGLVAAAFRLMKLVPAKYIIENAIASGQLNPGMPVLE

TSSGTFAMGIGIVCAEKRIPFHIVSDAAIDERLQARLRQLGGRVQIVGANATGSNVQVLRLEALQERLRE

NPGGFWPRQYDNPDNQHAYRAFAAQLIRTFGTNLTIVGTVGSGASTCGTIRALREVDPSIPLVGVDTFGS

VLFGLPVGPRALRGLGNSIYPNNLDHTCFDQVHWVAPDAAFGSTRRLHRQHGLYCGPTSGAAFMVAEWLR

AQRDDGRTIVFIAPDEGHRYADTVYDDAWLRGQGYAGADAAPAAAPVRAVSPNAASGPWAYVEWGRRTFE

QASGRPRPAGSALEQIRDVRPAPVA

>WP_004526065.1 membrane protein [Burkholderia pseudomallei]

MEKPSEPTREPQAREPHLRSVRLTSDFSLPKLSAIEIGSYLLALFGLWLVLHLKLLGGLLAGLLVYQLVH

MIAPAIERHMSSQRARWVAVVLLSTAIVGGLAGLTIAVIEHFEHTVPNAQNLLGQVMQIVDQARTRTPEW

ISNLLPVDAEQMRAKAAGLMNRHMDQLQQGGKSVARGFGHVLFGMIIGAMIAIGIDRHKLRQPLSTALVA

RISRFSDAFRRIVFAQIKISALNAFFTAIYLLVALPIFHQRLPLSKTLVLVTFIAGLLPVVGNLISNTLI

VAVSLSVGMGTAIASLVFLVVIHKLEYFLNAKIIGGQIESRAWELLLAMLVMEAAFGVPGVIAAPIFYAY

VKRELIYLRLI

>WP_004524376.1 hypothetical protein [Burkholderia pseudomallei]

MKSTQILASCALVGAVAALAGCAGSPANGPQAESPSGRIIYVASPRSPHDVESCLTSRVSQARVAQRNPT

TVLIGPYSADSDWTVTLAPTASSGTTIGVYRPRSGDGDPEESELRFHIARCAV

>WP_004523238.1 acyltransferase domain-containing protein [Burkholderia pseudomallei]

MAQVAFLFPGQGAFYAGATRALSGAYPVVQRAMQAIDAVSVRRLRRPLLATVWDGHIGAEDLLQHAPDLL

QLAIYATSVSYFEALRARGVRPDVLVGHSFGEIAALTCAGAFSIETGAEIVCDRIDALRAAAPADGRMAA

VGASPDAVRAHLAACFRGAPDARAGRVRIAVENHVSQTVVSGACEYVDEFRAYCARNNISAQVLNSPYAF

HHEDLENARIEFGRRLKAYKNKALEYPVYSPILNRYYTAADDLGACLARHLVLPVRFGDGVARLKAAGIG

AFVECGALNALSRISVRALGPGAVKTFPGASSAGGELAGLENVLAYFNGGSVMNDDIRASNLQLDFDEFW

RNSGPGIVDKIKGELKRFFDARGLQATPAPQIVADHGVAAGFAGGLTAGAADYAPKVAAGVSSAAAPVAA

PIVAAAPAAPLAVAPAAPVAQAAAVPAASARVPRDRLFVELVAIYAEAMEYPVEVFTETVELEAELGIDS

VKQTEIIQRISARYGLPPLPANFRAGDFKAMGQIVDFVYEHQGEAALVTS

>WP_004523043.1 flagellar protein FlgN [Burkholderia pseudomallei]

MHHMAHGRSARRHVERNEMRDELLATVNDEHATVEAFASLLAYEEKALTTAAPLDALPGIVERKTELLEK

LAQLERRRDTLLASLGLPAGKKGMDLAAENDARLANGWQLLQHSAERARHANAINGMLIRIRMDYNERTL

SVLRAAPQRNGFYGPDGRVAAVAR

>WP_004522915.1 ammonium transporter [Burkholderia pseudomallei]

MRKILMSLLMAGSLLAGGIGAAMADDASSAPAAASAATASDTSAGAAASAPAASAAPAAPAAPAASAPAA

ASAAAPASAAAAPAAPTAPFSVDSSKINSGDTAWMLTSTALVLFMTIPGLALFYGGMVRKKNVLATVMQS

FAITALITVLWTVVGYSLAFTPGNGFIGGFSRVFLSGMNYIHGDKATTLTVSHLASTIPESVYFVYQMTF

AIITPALICGAFADRMKFSAMLVFMTLWSLIVYVPIAHMVWEPTGWLSADGVLDFAGGTVVHINAGIAGL

VSCLMLGKRVGYGREAMAPHNLVLTLIGGSMLWVGWFGFNAGSAVAADGRAGFAMLTTQVATACAALGWM

FAEWIAKGKPSVLGIVSGAVAGLVAITPAAGFVGVAGALVIGIAAGVVCFWSATWLKHKLGYDDSLDAFG

VHGVGGILGALLTGVFAVKDIGGADGSLLLQAKGVAITLVYSGIVSFVLLKVIDIVIGLRVTEEEEREGL

DVILHGEHVE

>WP_004522592.1 copper homeostasis periplasmic binding protein CopC [Burkholderia pseudomallei]

MTMTITSLARRGLAAALALALSSAAFAHAKPEKSDPPANAAVAAPQAVSIDFTETLEPAFSSIVVVDAAG

APAQAAKAVVDASDRKRMSVALKPLQAGVYTVKWVALATDGHRTQGRYTFTVK

>WP_004522327.1 DUF2946 domain-containing protein [Burkholderia pseudomallei]

MKRSTRWMSLVWLALVLNVLSPVVGYARAAANAADSPFALELCSAAGAQRVVVDSGGEPRRGDASLAHVA

HCVYCPGFAANLALGSSTPALPGFVRAFVYASRVERPAVFFRRGVRIAQPRAPPETVPV

>WP_004522006.1 hydroxyphenylacetyl-CoA thioesterase PaaI [Burkholderia pseudomallei]

MAARSPERSPTATAERSAEALARATAEAMYAADACSRSLGIEVLEVRPGYARARMPVRGDFLNGHQICHG

GLVFTLADSTFAFACNSYNINTVAAGCSIEFLRPVAGGDVLTAEATEQTLNGRHGIYDIRVTNRAGETVA

MFRGKSTQIKGTVIPVDR

>WP_004521312.1 helix-hairpin-helix domain-containing protein [Burkholderia pseudomallei]

MLKKLLMLVVALSLSLTAALAAAVEVNTADQAALESVKGLGPVKSKAIIDERAKNGPFKDADDLANRVKG

LGAKSVAHLEAAGLTIGGSTTPPTGAKAKTGTAKPAATATPAPAPAPAPTTSSATPAPSAATPAPATATP

STAASAPAAKKSRSSKKQDKAAAASAAAQASAPAAASTTKAKGSKKSKKNKAATPASAASGA

>WP_024428580.1 hypothetical protein [Burkholderia pseudomallei]

MRRAPPIPILFAVACVAARAAGLPMPVLSRLPPGYEVMAARQGPDVDAGRISYLIVLHRPADSASEPSPR

PLVIVEQQADGTFRLAARNDEVVLRANEGGQCDPFDPQDADENGLAVKGRFFTVQNFVACGQHWSDYVTF

RHDARTGRWLFANEIRTESFPLEGKPDRVRAIRADPRKPVALDAWRRGD

>WP_004548294.1 hypothetical protein [Burkholderia pseudomallei]

MAAMRTFAMKLDVVRRACAALGGAAVLAAAPALAAPPAPDGGRAPDLDAVLLHESMTVSADGVTRTVTYR

ERMVRRDGHVWIERVPPSGAKRAHAGGAHAHEGLDAARTPPSRGGAHPAAAAADAHAGHRHFDFDSAARH

VTNDGGRIGVEYVDAAQRTVVAVPPAEYETTGFDGSWDNAFYITPPSQLKRLAAQSKPGPAPGTRWYEQT

VGAPRAQGTNRVLWSDALQAPLVVEYRSADGHASRKLTLTPAPRANALPWRQLQSYTRKAYVDYLD

>WP_004546378.1 capsular polysaccharide biosynthesis protein [Burkholderia pseudomallei]

MQALDAALEQDCTAGTAAAVAELMKRVLASHAIRGRDGVSEFRAPPRLPGETRVLLIDERKYSQGIGAVA

TRNNRGAFERMIQAARAAHPDAEFWLARTRDRGSGVWLSASAADILPPDIHRLGEHESLCAALEHVDHVY

TVGASEGMQALLAGRRVHVFGAPYYAGWGLTDDAVQLPGRHARPTLAALFDVVFLRFARYLNPATHAPGR

IDDLLDAIEWQNTVRRRFADLRQVAGIRFQWWKRPFATPYLTAGGGTLRWTRDASRLREGEHAALWGARG

TNDLPPGTRVIRIEDGFLHSTGLGSDHVAPCSQVIDRSGLYFDPSRPSDLTTILNETDFDDAELVRANRL

RREIARLGLTKYNLGRRKPAWSPPPGKRVVLVPGQVADDASIRLGTRGITTAEDLLREVRARRPDAFIVY

KPHPDVLSGNRRGAIEVNAWADLIEQDADLISLIEVADEIHTLSSLSGFEALIRGKAVHTYGLPFYAGWG

LTQDALAQPWRKRTLSLDMLTAGVLLRYPVYWDWSLRLFASPELVVRQLAIPAARPLTSIRGDRLRPVRK

ASRWIASCLRHLLWQCGK

>WP_004546377.1 pilus assembly protein TadG [Burkholderia pseudomallei]

MSRVTSSSGGARPCGRRRQRGVVSILVALMLAVLIGFVGLALDLGKLYVTRSELQNSADACALAAARDLT

GAINLSVPEAAGITAGHLNYALFEQFPVQMQTNSNVTFSDSLSNPFQPKNAIASPSSIKYVKCTTSRTGI

VNWFIQTLNLVPGVTVANASVSATAVATVGAAQTTCAIPVFICKAGTQTSPPVAGATYNIGDWLSAKTGS

PPSFGAGNFGWSALDGSNSASSIANELTGNYCALPATGSQVGTPGDKAATTNAYNTRFGIYANPYKNPSY

GTPDFTGFAYDATTWPSQSNAYSDFVSKRLTFASYQGDLITGINTGGSYNPSYYAAGADRRLALAPEVDC

SVLLSGHSAPVLSWDCVLMLDPMGSGGSATPVHLEYRGSSTASGSPCATQGTPGNGSSVGPQVPVLLQ

>WP_004534636.1 ABC transporter permease [Burkholderia pseudomallei]

MTPRTAGGAAAPAPAPRPRAMPAWAARVDKVGVLIAALVAYAAFVLPFVTLRANRIAAGAELAPAAVFPA

LHAYALDALWAAGALFALVHSRAAWRAAVGVGLVFALGVAIGAAPAHLVTPDTPLARVSPAAGAWLLLFA

FAVLIADALARIALAPAMRLVALAAASAALAAFIHGGFWDGLSVMQEYAVRADTFRNEAIRHLALVAGSV

AAAVALGVPLGIGCTRSAALRGALLPLLNVVQTIPSIALYGLLMAPLAILAARVPLAARLGVSGIGVAPA

LIALFLYALLPIVSSVVVGFAQVPAAVVEAALAMGMTGRERLVAIELPLALPVVLSGVRIVLVQNIGLTA

VAALIGGGGFGTFIFQGIGQSATDLVLLGALPTIALALVTAVLFEAATDLAKGARR

>WP_004534057.1 peptidase S10 [Burkholderia pseudomallei]

MKKSMHGGYGGLARPCGRMPATAAVAAALLLALGGCGDDLQSTTTPAQLNQPYTDTTAYSPKAGDGLPAS

QVSERAAVMSHQWTANGASVDYLTTTGHLTATDPNGNAEATMSYVAYTAPSRDGSPRPVTFFYNGGPGSS

SVWLRLGSFAPTRVATPDPLMTNWPNFPLVDNPESLIATTDMVFIDPPGTGLSEAIQPNTNQTFWGADAD

VKVMRDFIRRYLSVNGRGGSPIYLYGESYGTPRTDMLALALESAGVPLTGIVLQSSILNYMAAAGDQAVG

TFPSYAQVAAYFNQVSPSPTNLGAYAQRIENFVTAQYAPIVHYATASSPISPDAGTLAAWSSQTGMATAS

IGAYFQYFYDTEPSPGQTTLVPGYTIGRYDGRVSLPNGDARLASDDDPSDILISKPFTSALASQMPNYLG

YTAPNATYQTLNPDIIGVWNFSHAGQPYPDTIPDLLAALQLNPKLKVLASNGYHDLATPYFETEKELARL

QTVSGLAPNLQVTFYQGGHMIYLDDVARPQMQADLVAFYQNRPVANALTLAALPSPWPDESPANTPTAKI

ARAAAAR

>WP_004530192.1 hypothetical protein [Burkholderia pseudomallei]

MKHDAAHHGERAISMNHSTITWAAVLALCTGFAVTNAHARDSAECRRLSDATGGADDNFRPPLTATVTGA

GRAYFHSAPASACIARRVFVVPGDTVTVYKPYRHWYQVTYVNGRTGEDVEGWIEAGRLRLGGHLGGGQ

>WP_004527761.1 2-dehydro-3-deoxy-6-phosphogalactonate aldolase [Burkholderia pseudomallei]

MSMDAEALRFPAPYAPHRALMRAFGACPMIAILRGVTPADAAAHGAALYEAGFRIVEVPLNSPDPFDSIA

ALRTALPADAIVGAGTVLRAGHVDEVVRAGGELIVMPHADGDVVRRAKTLGIGCAPGVATPTEAFAALAN

GADVLKMFPAEQLGTPAVKAWRAVIDARVPLVPVGGITPENMGPYLAAGASGFGLGSALYAPGQTSAVTA

VRAHAFINGWRIARHGAAR

>WP_004548250.1 phosphatidate cytidylyltransferase [Burkholderia pseudomallei]

MLKTRVITAVVLLAVLLPVTLFAPLAAFGALIALALVFAAWEWGRLLKLGGAGPLGYAAVAALALGASTR

LGIGAAAARPLFAAAGVFWLLAGPYALWRKPALAERAWKPFLLVAGLVVFSACWHALVAARAQGVPFVLS

LLVVVWLADIGAYFAGKAFGKHKLAPSVSPGKTWEGAAGGWLAVMIVAGAAVAAHAFEPTLYSTFVARYG

AAGAFAALTVLVAFSVVGDLFESLLKRQAGVKDSSGLLPGHGGVLDRVDALLPVLPLAMLLLG

>WP_004538773.1 MULTISPECIES: cytochrome c biogenesis protein CcsA [Burkholderia]

MDIVLYALTVLLYGGLAVAGWRARRVGAARPLVASVPAVAPARESAPGGMGGLGRTILGAALLAHGVLLH

MTIFPHDAMVFGFAFALSAMFWLGAGIYWIESFFFPLDGMRLLVLPLAGVASLLPLAFGGVRVLPYAAAP

LFKVHFLIANIAYGLFAIAALHAVLMLMVERRLHALRHDGLRDASGWVAGWLDTLPPLLTLEKLLFRLIG

AGFVLLTLTLATGILFSEQIDARALKLDHKTVFAILSWLMFGGLLVAHKASGWRGRGAARWVLASFVALL

LAYVGSRFVLEVLLHRSVV

>WP_134778877.1 ribonuclease G [Burkholderia pseudomallei]

MNEEILINITPQETRVALVQQGAVQELHVERTLSRGRVGNVYLGKVVRVLPGMQSAFIDIGLERAAFLHV

ADIWQPRAGDAPAAATHQPIEKIVFEGQTLMVQVIKDPIGTKGARLSTQISIAGRTLVYLPQEPHIGISQ

KIESEAEREAVRARLTAVIPSDEKGGYIVRTIAEDATSDELAADVAYLRKTWATIVAQGQRVPPTSLLYQ

DLDLAQRVLRDFANDDTTRIQVDSRETHQRLVEFAAEFTPAVSPKLHHYTGERPLFDLYNIEAEIQRALS

RRVDLKSGGYLMIDQTEAMTTIDVNTGGYVGARNFDDTIFKTNLEAAHTIARQLRLRNLGGIIIIDFIDM

ENAEHRDAVLAELKKALSRDRTRVTVNTFSQLGLVEMTRKRTRESLAHVLCEPCPTCQGKGQVKTSRTVC

YDILREILRESRQFNPREFRVIASQQVIDLFLDEESQHLAMLIDFIGKPVSLQVESNLSQEQYDIVLMWE

QYDIVLM

>WP_052133812.1 thiol peroxidase [Burkholderia pseudomallei]

MSKVTLGGNPIDIAGTFPTVGSPAPDFKLVGKDLADVSLATFAGKRKVLNIVPSLDTPTCAASTRKFNEA

ASKLDNTVVVVVSADLPFAATRFCTTEGLANVITASTFRGGRAFADAYGVNVTSGPLDGLAARAVIVIDE

NDKVTYTELVGEIKNEPNYDAALAALKKGSSQKTVEPWRTIA

>WP_024429041.1 endopeptidase La [Burkholderia pseudomallei]

MSGTQLLPSERTALPLLPLRDVVVFPHMVIPLFVGRPKSIKALEVAMEGGKHIMLVAQKTAAKDEPTEKD

MYDVGCIANILQMLKLPDGTVKVLVEGLQRAQALSIEEQETQFSCEVMPLEPDHADSAETEALRRAIVSQ

FDQYVKLNKKIPPEILTSLSGIDEAGRLADTIAAHLPLKLDQKQHILEMFPVIERLEHLLAQLEAEIDIL

QVEKRIRGRVKRQMEKSQREYYLNEQVKAIQKELGEGEEGADLEELEKRINAARMPKEAKKKADAELKKL

KLMSPMSAEATVVRNYIDTLIALPWRKKSKVNNDLSNAEKVLDEDHYGLEKVKERILEYLAVQQRVDKVK

APILCLVGPPGVGKTSLGQSIARATNRKFVRMALGGVRDEAEIRGHRRTYIGSMPGKILQSLSKVGVRNP

LFLLDEVDKMGMDFRGDPSSALLEVLDPEQNHTFADHYVEVDFDLSDVMFVATSNSLNIPPPLLDRMEVI

RLSGYTEDEKVSIAQRYLLPKQKKNNGLKEGEIDVAEQAIRDIIRYYTREAGVRSLEREVSKICRKVVKM

LLLKKASGAIKVDGENLDTFLGVRKYDFGLAAKENQVGQVTGLAWTEVGGDLLTIEAAVMPGKGNVIRTG

SLGDVMKESVEAARSVVRSRSRRLGIKDEAFEKQDIHIHVPEGATPKDGPSAGIAMTTALVSVLTGIPVR

ADVAMTGEITLRGEVLPIGGLKEKLLAAHRGGIKLVLIPEENVKDLADIPDNVKNAIEIVPVRWIDKVLE

LALERLPNALPEEEAKAAPVAEPAKDAGSTEVVKH

>WP_024428839.1 formate dehydrogenase subunit alpha [Burkholderia pseudomallei]

MTSSAFDSSRQGCGSGQCACRRAAQTRRADPFDDTDYGTPTRHADTDVTLDIDGRAVTVPAGTSVMRAAI

EAGINVPKLCATDSLEPFGSCRLCLVEIEGRRGYPASCTTPVEAGMKVRTQSDRLQALRRNVMELYISDH

PLDCLTCAANGDCELQDMAGAVGLREVRYGFDGKNHLSDAKDESNPYFSYDPSKCIVCNRCVRACEETQG

TFALTIASRGFESRVAASAGEAFMDSECVSCGACVAACPTATLIEKSVARLGQPEHEIVTTCAYCGVGCA

LKAEMKGEQVARMTPHKNGLANEGHACVKGRFAWGYATHKDRITKPMIREKITDAWREVSWDEAIGYAAA

RFRRIQDEHGRDSIGGITSSRCTNEETYLVQKLVRAAFGNNNVDTCARVCHSPTGYGLKTTLGESAGTQT

FASVGSADVIVVIGANPTDGHPVFGSRLKRRVREGAQLIVVDPRRIDLVDGPHVKAVHHLQLRPGTNVAL

VNALAHVIVTEGLVDEAFVAERCEPHAFDVWRAFAARPENSPEATADITGVPADAVRAAARLYATGGRAA

IFYGLGVTEHAQGSTMVMGIANLAMATGNLGIEGAGVNPLRGQNNVQGSCDMGSFPHELPGYRHIGDAAV

RARFDEAWSTTLQPEPGLRIPNMFDAALDGSFKGLYCQGEDIVQSDPNTQHVAAALASLDCLVVQDIFLN

ETAKYAHVFLPGATFLEKDGTFTNAERRISRVRRAMRPLSGYADWEVTLMLSRALGYDMHYAHPSEIMDE

IARLTPTFAGVSYALLDELGSVQWPCNDAAPQGTPTMHVDQFVRGKGKFVITQYIASPEKVTPRYPLILT

TGRILSQYNVGAQTRRTENVRWHGEDRLEIHPHDANDRGIRTGDWVGVASRAGQTVLRALVTERMQPGVV

YTTFHFPESGANVITTDSSDWATNCPEYKVTAVQVAPVAQPSEWQRAYTRFRAEQLALLEQRTAERAPAI

ATGK

>WP_004557110.1 MULTISPECIES: D-alanyl-D-alanine endopeptidase [Burkholderia]

MKAQLFAPLRMMQSVALGTAVSVAVSLMVATAAVAPADAFAATAKTAQSAKGKKSAAKKSLRAASSSAEP

RAKGARKRVTYVSNGRHRSGVRRVAFQPRPPSVGQAFGLHETPDSLALRSSVAYVVDQNTSEPLFDKNSH

AVVPIASITKLMTAMVVLDSKSPLTDQIDVTDEDRDYEKGTGSRLSVGSVLSREDMLHIALMASENRAAA

ALSRYYPGGRPAFIAAMNAKAKSLGMTDTHFENSTGLSSQNVSSARDLVKMVNAAYQYPLIRKFSTDRSY

EVNTGKRTLAYNSTNALVRNPSWDIGLQKTGFINEAGECLVMQTTIHGRPIVMVLLDSFGKYSRFADASR

VRNWLDNGGGERLTAANTSTGGT

>WP_004551024.1 MULTISPECIES: 1,2-phenylacetyl-CoA epoxidase subunit B [Burkholderia]

MNNEWPIWEVFVRSKQGLDHKHCGSLHAADASMALRMARDVYTRRREGVSIWVVPSAAITASDPNEKAEM

FEPAGDKIYRHPTFYTLPDEVNHM

>WP_004543903.1 glycerol-3-phosphate dehydrogenase [Burkholderia pseudomallei]

MTQQNRYDLLVVGGGINGAGIARDAAGRGLSVLLCEQDDLASHTSSSSTKLIHGGLRYLEYKEFGLVRKA

LQERETLLRAAPHIMWPLRFVMPHMPNLRPAWLIRVGLFLYDHLAKRELLPGSRGIDMRRHPAGAPLVDS

IKRGFVYSDGWVDDARLVVLNALDAQERGARILTRTKLVSAERRDGQWHARLQRADGSTLDVLARAIANA

AGPWVGEVLHGALGRGAQHSVRLVKGSHIITPRLFDHDHAYIFQNPDKRIIFAIPYERDFTLIGTTDVEY

RDDPSRVAIDRDETRYLCESINRYFKRKISPADVCWTYSGVRPLLEDENADNPSAVTRDYRLELDDGEGA

PLLSVFGGKITTFRKLAEEATDMLGGALGAARGAWTAGVPLPGGDIANARFAPFAEAFAKRHPWLPAALA

RRYARAYGTRAERVIGRARSLAELDAQLAPGLYEAELRYLRDVEWAGCADDVLWRRSKLGLHVAPGTLEP

VTAALDAWFGAAREAASAAH

>WP_004530939.1 aminoacyl-tRNA hydrolase [Burkholderia pseudomallei]

MIKLIVGLGNPGAEYTATRHNAGFWLVDQLAREAGATLRDERRFHGFYAKARLFGEEVHLLEPQTYMNRS

GQAVVALAHFFKILPTEILVAHDELDLPPGAAKLKLGGGSGGHNGLKDISAHLSSQQYWRLRIGIGHPRD

LIPESARAGAKPDVANFVLKPPRKDEQDLINAAIERALAVMPTAIKGETERAMMQLHRNGA

>WP_004524905.1 MULTISPECIES: UDP-N-acetylglucosamine 2-epimerase (non-hydrolyzing) [Burkholderia]

MKKILLAFGTRPEAIKMAPLVRMLQTRADVDARVCVTAQHRQMLDQVLALFDIAPAYDLNVMRQSQTLAD

VTTGILQTIGAVFDDFDPDIVLVHGDTTTTLAVSLAAFYRYLPIGHVEAGLRSGDIWSPWPEELNRRVTD

AVSSWHFAPTERAQHNLFSEGVPTQGVVLTGNTVIDALQDVKRMLDADAPLAREIAAQFPFLGHDERVVL

ITGHRRESFGEPFAHFCDALRTLARRYPGVRFVYPLHLNPNVQGPAHALLDGLPNVHLIAPQEYLSFVFL

MSRAHFIITDSGGIQEEGPALGKPVLVTRDTTERPEAIQAGTARLVGTDTERIVGEASRLLDDDDAYDEM

SRATNPYGDGHASERIAHALLNMPHAADTARFPLAAIEAPCNALALGLRALRSA

>WP_004522377.1 MULTISPECIES: molybdopterin adenylyltransferase [Burkholderia]

MTTTKRNHPDELVIGLVSISDRASSGVYEDKGIPALAEWLAAALVSPWRAETRLVQDDAPTISATLVELV

DTLGCDLVLTTGGTGPARRDVTPEATLAVATKEMPGFGEQMRQISLNFVPTAILSRQVAVIRETADHAAL

IVNLPGQPKSIKETLEGLRGADGKVEVPGIFAAVPYCIDLIGGPYAQTNPAVCAAFRPKSAVRAPK

>WP_004522034.1 signal recognition particle protein [Burkholderia pseudomallei]

MLDNLTQRMARVVKTLRGEARLTEANTQEMLREVRLALLEADVALPVVRDFIAKVKEKALGEDVIGSLSP

GQALVGVVQKELTAVIGGDYEGKAAELNLAVAPPAIILMAGLQGAGKTTTVGKLAKLLREKYKKKVLTVS

CDVYRPAAIAQLKTVSEQVGADFFPSTPDQKPVDIANAAVDWAKRHYHDVLLVDTAGRLGIDEAMMQEIA

ALHTALKPVETLFVVDAMLGQDAVNTAKAFNDALPLTGVVLTKLDGDSRGGAALSVRHVTGKPIKFVGVA

EKLDGLEIFHPDRMANRILGMGDILALVEEAQRGVDIQAAEKLANKVKKGGDFDLNDFRAQISQMKNMGG

LSSLMDKLPAQFQQAAAGADMSQAEKQIRRMEGIINSMTPAERAKPEIIKATRKRRIAAGAGVPVQEVNR

MLNQYDQMRTMMKKLKGGNLQKMMRGIKGMMPGMR

>WP_004266656.1 MULTISPECIES: 1-deoxy-D-xylulose-5-phosphate synthase [pseudomallei group]

MYDLLKTIDDPADLRRLDRRQLQPLADELRAFVLDSVSKTGGHLSSNLGTVELTIALHYVFNTPDDRIVW

DVGHQTYPHKILTGRRDGMKTLRQFDGISGFPRRSESEYDTFGTAHSSTSISAALGMAIGSKLNGDDRFS

IAVIGDGAMTAGMAFEAMNNAGVSEDAKLLVILNDNDMSISPPVGALNRHLARLMSGRFYAAARAGVERV

LSVAPPVLELARKLEEHAKGMVVPATLFEEFGFNYIGPIDGHDLDSLIPTLQNIKELRGPQFLHVVTKKG

QGYKLAEADPVLYHGPGKFNPAEGIKPSTTPAKKTYTQVFGEWLCDAAELDARVVGITPAMREGSGMVEF

EKRFPERYYDVGIAEQHAVTFAGGLATEGLKPVVAIYSTFLQRAYDQLIHDVALQNLPVVFAIDRAGLVG

ADGATHAGAYDLAFLRCIPNMTVMAASDENECRQMLHTALQQPNPTAVRYPRGAGTGVATVKAFTEIPLG

KGEVRRRTSQPDGKRIAILAFGTMVAPSLAAADALDATVANMRFVKPIDAELVQALARTHDYLVTVEEGC

VMGGAGSACVEAMMESGAVRPVLQLGLPDRFVDHGDPAKLLSLCGLDGDGIAKSIRERFLSHAADVASPA

KRVA

>WP_004199927.1 MULTISPECIES: outer membrane lipid asymmetry maintenance protein MlaD [Burkholderia]

MTMKKTALDFWVGLFVVLGFLALLFLALKVGNMSSLSFQPTYAVKMKFDNIGGLKPRAAVKSAGVVVGRV

KTIGFDTNTYQALVTIELDKQYPFPKDSSAKILTSGLLGEQYIGLDPGGDTEMLKAGDTITMTQSAIVLE

NLIGQFLYSKAADAGGAKPGAAASGAPAPAPVAASAASAASGAAAQ

>WP_004194543.1 MULTISPECIES: L-threonine 3-dehydrogenase [Burkholderia]

MKALAKLERGPGLTLTRVKKPEVGHNDVLIKIRRTAICGTDIHIWKWDDWAQKTIPVPMHVGHEYVGEIV

EMGQEVRGFSIGDRVSGEGHITCGFCRNCRAGRRHLCRNTVGVGVNREGAFAEYLAIPAFNAFKIPPEIS

DDLAAIFDPFGNATHTALSFNLVGEDVLITGAGPIGVMAVAIAKHVGARNVVITDINDYRLELARKMGAT

RAVNVSRESLRDVMADLHMTEGFDVGLEMSGVPSAFTSLLESMNHGGKVALLGIPPAQTAIDWNQVIFKG

LEIKGIYGREMFETWYKMVAMLQSGLDLSPIITHRFAVDDYEKGFAAMLSGESGKVILDWADA

>WP_004193561.1 MULTISPECIES: nucleoside-diphosphate kinase [Burkholderia]

MALERTLSIIKPDAVAKNVIGQIYSRFENAGLKIVAARMAHLSRADAEKFYAVHAERPFFKDLVDFMISG

PVMIQVLEGEDAILKNRDLMGATDPKKAEKGTIRADFADSIDANAVHGSDAPETARAEVAFFFPEMNVYS

R

>WP_004193488.1 MULTISPECIES: aminoacyl-tRNA hydrolase [Burkholderia]

MTLRYAFSPDEVELTAVRAQGAGGQNVNKVSSAIHLRFDIRASSLPDDVKARLFARADQRITREGVIVIK

AQEYRTQEKNRAAALARLDTLIRSAGVTPRKRVATRPTRASKERRLAEKGRRSEVKSGRGRVVE

>WP_004192585.1 MULTISPECIES: phosphopyruvate hydratase [Burkholderia]

MSAIVDIIGREILDSRGNPTVECDVLLESGTMGRAAVPSGASTGSREAIELRDGEAGRYGGKGVLKAVEH

INTEISEAIMGLDASEQAFLDKTLLELDGTDNKSRLGANAMLAVSMAVAKAAAEEAGLPLYRYFGGSGAM

QLPVPMMNIVNGGAHANNSLDIQEFMIVPVSQPTFREALRCGAEVFHALKKILGDRGMSTAVGDEGGFAP

NFGSNDECLSTILQAIEKAGYRAGEDVLLALDCAASEFYHDGKYQLAGEGLQLSSAEFTDYLATLADKFP

IVSIEDGMHEGDWDGWKLLTERLGKKVQLVGDDLFVTNTRILKEGIEKGIANSILIKINQIGTLTETFAA

IEMAKRARYTAVISHRSGETEDSTIADIAVGLNAGQIKTGSLSRSDRISKYNQLLRIEEDLGDIASYPGK

SAFYNLR

>WP_004192168.1 MULTISPECIES: amino-acid N-acetyltransferase [Burkholderia]

MNSQTDLPAAQSGAAPQPSVDEAVHHAQFVDWMRSVAPYIHKFRNSTFVVGFGGEVVQHGLLSALVSDIA

LLQAMGIQIVLVHGSRPQVEEQLHLHGVESAFSHGLRITDARALESAKEAAGEVRLDIEAAISQGLPNTP

MAHAHISVVSGNFVTARPVGILDGVDFAHTGVVRKIDAESIRHSLASRKLVLLSPLGFSPTGEAFNLSME

DVASAAAIALRADKIIFLTESPGIVDEAGELVREMSLDAAAELLDSGDLQGDDGFFLKHAIRACRGGVAR

AHLIPQSLDGSVLLELFLHDGVGTMISYENLESLREATPDDVGGILTLIEPLETDGTLVRRGRHQIERDI

DHFSVIEHDGVLFGCAALYPYPTEKIGEMACLTVAPEAQGSGDGERLLKRVEQRARARGLTRIFVLTTRT

EHWFLKRGFVKATVDDLPEDRRKLYNWQRKSLVLMKQL

>WP_004192143.1 MULTISPECIES: ribosome recycling factor [Burkholderia]

MSVADIKKSVEQKMQRSIEAFKNDLAKIRTGRAHTGLLDHVQVDYYGSMVPISQVANLTLVDARTIGVQP

WEKTMVAKVEKAIREADLGLNPATSGDLIRVPMPPLTEERRRELTKVVKSEGETAKVAVRNLRRDANEQL

KKLVKDKEISEDDERRASDDVQKLTDKHVAEIDKLVQAKDAEIMTV

>WP_004191092.1 MULTISPECIES: Hsp33 family molecular chaperone HslO [Burkholderia]

MSDQLQKFMFNAAPVRGEIVSLRSTWQEVLTRRDYPTPVRNVLGEMMAACALLSANLKFDGTLIMQIFGD

GPVKMLVVQCSSDLAMRATAKFSGDAARTVGDGTSFAELINASGHGRCVITLDPADKRPGQQPYQGIVPL

NGEDGPLASIADVLEHYMRHSEQLDTRLWLAADHDRAVGVLLQKLPGDGGIVPRVEQTDTDTWERVCTLG

GTLSSKELLEVEPETVFRRLFWQENVQHFEPTSTRFQCTCSREKVGGMLRMLGRVEIDGVIEERGHVEIH

CEFCNQRYEFDPVDVAQLFSTPELGTGVAPAAAQRH

>WP_004189767.1 MULTISPECIES: oligoribonuclease [Burkholderia]

MTDISAVAGQPALVRNELNLVWLDMEMTGLDPDTDRIIEIAVVVTNSTLDIAVEGPVLAIHQSDETLAKM

DDWNKNTHGRSGLIDRVRASSVTEADAAAQIAAFLAEHVPPGKSPMCGNSICQDRRFMARWMPELERFFH

YRNLDVSTLKELCRRWQPAIYKGFQKRAMHTALADIHESIDELKYYRERFLIPAAPAGETA

>WP_004186923.1 MULTISPECIES: ribulose-phosphate 3-epimerase [pseudomallei group]

MTQFRIAPSILSADFARLGEEVRNVVAAGADWIHFDVMDNHYVPNLTIGPLVCEAIRPHVQVPIDVHLMV

RPVDRIVPDFAKAGANLISFHPEASDHIDRTLGLIRDHGCKAGLVFNPATPLNYLDHVMDRVDLVLIMSV

NPGFGGQSFIPEALNKLREARARIDAYTARTGREIHLEIDGGVKAENIAEIAAAGADTFVAGSAIFGKPD

YRQVIGEMRDALATVGRACAR

>WP_004186718.1 MULTISPECIES: dUTP diphosphatase [Burkholderia]

MKLDLKILDARMRDYLPKYATTGSAGLDLRACLDAPVTLKPGDTALVPTGLAIHLADPGYAALILPRSGL

GHKHGIVLGNLVGLIDSDYQGELMISTWNRGQTEFALNPFERLAQLVIVPVVQARFNLVDDFAQSERGAG

GFGSTGRG

>WP_004186400.1 MULTISPECIES: ribonuclease PH [pseudomallei group]

MTNSSLRPSGRRADQLRDVRITRHYTKHAEGAVLVEFGDTKVICTASVAERVPEFLRERGQGWLTAEYGM

LPRATHTRSDREAARGKQTGRTQEIQRLIGRALRAVFDLNALGPRTLHLDCDVIQADGGTRTASITGAFV

AAHDAVTKLVAAGRIARSPITDYVAAISVGVFGGTPVLDLDYDEDSACDTDMNVVMTGAGGFVEVQGTAE

GAPFSRTEMNALLDLAQAGIGELVRLQRAALEA

>WP_004185877.1 MULTISPECIES: guanylate kinase [Burkholderia]

MTDSNRDGAAAHSLHAGVYPGNLFMVVAPSGAGKSTLVNALLSKDPEIRLSISYTTRKPRPGEQDGQHYH

FTTVEDFRERHARHEFLESAEVHGNYYGTSRVWIEEQMKIGHDVLLEIDWQGAQQVKKQFRNAVGIFILP

PSLAALEERLKKRGQDEPNVITRRLLAAGSEIAHAAEAQYVVINETFEHALAELECIVAATRLRFTSQYA

RHAELFVELGIHLPHAE

>WP_004185840.1 MULTISPECIES: adenylate kinase [Burkholderia]

MRLILLGAPGAGKGTQANFIKEKFGIPQISTGDMLRAAVKAGTPLGVEAKTYMDEGKLVPDSLIIGLVKE

RLKEADCANGYLFDGFPRTIAQADAMKEAGVAIDYVLEIDVPFSEIIERMSGRRTHPASGRTYHVKFNPP

KVEGKDDVTGEPLVQRDDDKEETVKKRLDVYEAQTKPLITYYGDWARRGAENGLKAPAYRKISGLGAVEE

IRARVFDALK

>WP_152769068.1 DUF21 domain-containing protein [Burkholderia pseudomallei]

MRRVDQIPLWAQIGAVFLLLLCSSFFSISETAMMALNRHRLKHLASHGALGAKTTQRLLARTDQLLSVIL

IGNNLFNTIIPVLTTSIALRTFGHDNFVLSIATGIVAFLIIVFAEITPKIVGATFPERIALPASVVIAPL

MRVMMPVVWFVNTLSGAILAALRINTKAGRDQRLSTEELRSLVLESGSFMPTKHRSILLNLFDLENITVD

DVMIPRRQIESLNILAPLDDVLHQLETCYHNRLVVYEGDIDRVLGVLHVRKTLAAFHNQDFDRGTLRELL

AQPYYVPSGTPVFQQLQYFQESRHRTALVVNEYGELEGLVTPEDIIEELIGEFTTTMPRSENSKSGWDAN

GECIVAASMPLRELNRWLHLGLPTNGPKTLNGLILEELEEIPEDDVCLKIGDVMIEVMRTEDQGVRTVKL

FKPRAPRGGRAAGA

>WP_152769066.1 helix-turn-helix domain-containing protein [Burkholderia pseudomallei]

MENDVTSAAAAAPAACAEPVPSVAHFGFLTLPNFSMIAFTSAVEVLRMANYVARADHYRWSIFSLDGAPV

RASNGIAVRPTQPLDVDDPPDVVIVCGGIRIREAVDERVRDALGALAARDVPLGGICTGAYALMACGLLD

GYRCAVHWENLSALHAEFPRVRFADELFAVDRDRLTCTGGTAPLDLMLNLVGARLGQPLAAQVSEQFILE

RIRGATDPQPIPVDARVGFSRAELIEVVRLMEANIEEPLSLEELARLVRLSQRHLQRMFKIYLNVSPTHY

YLTLRLKRARDLLRTTDASIARVTAVCGFHSPCHFSKAYRAQFGHAPSHERRVSAR

>WP_152769026.1 L-rhamnose mutarotase [Burkholderia pseudomallei]

MTRLHARRTTERTVRYCLALDLKDDPDAIARYEAHHERIWPEVAAYLRAHGVVAMEIYRLGTRMTMVMDT

DDACFDAARFDADARADAKIVEWEALMGAFQQPTPWTPAGVKWTPMTRIFDLSKQ

>WP_152769015.1 FAD-dependent oxidoreductase [Burkholderia pseudomallei]

MGHRIVTDSPTDVIVIGAGIVGAACAHEFALRGLRVVVVDDGGGGATAAGMGHLVAMDDNAAELALTHYS

IGLWRALRDAMPEGCAYRNCGTLWLAADAHEMDLARAKQAALGARGVAGELIGRAALAALEPMLRTDLGG

ALKIPGDGILYAPAAAHWLLHRLPGVALRRARAVALDGARVALEGGDVLRANAVVVANGVAARALVPELP

LRPKKGHLLITDRYPARVSHQLVELGYAASAHASDGTSVAFNVQPRPTGQLLIGSSRQFDTEDPRVEAPV

LARMLRRALGYLPALAGLNGIRAWTGFRAASPDGLPLLGEHPSRRGVWLALGHEGLGVTTAPGSARVLAA

LMFGERAAIDAEPYRPARFLSTSKSAAAGELR

>WP_152768972.1 GNAT family N-acetyltransferase [Burkholderia pseudomallei]

MGRIAFRRHRDRAVGRAGGGVSAIAGNPIAIRAAAAADVPAMLALMRELAEFEQLTHLFVATEADLADAL

FGARPSAEALVAVNGGTLVGYALYFHNYSTFVGRRGLYLEDLYVQPSQRGTGLGTRMLRRLAAIAVERRC

GRFEWTVLDWNQPAIDFYRKMGADVMPDWRVVRVAGDALARLADGAA

>WP_152768964.1 glycosyltransferase [Burkholderia pseudomallei]

MAEAATRERRALSGSRPVRIAIVTHVVRHNDGQGRVNYEIARAALAENCQVTLVASHVAPELLADARVRW

IAVKAGRFWPSNLVKQQVFAIKSAWWLRRHREAYDVLHVNGFISWVRADVNTAHFVHSGWFASRYYPFGL

SKGLWSAYQYVYTRVNTRLERWAYRRARAITAVSQKVADEIRRIGIDGGRIGVIYNGVDAQAFANAAPDR

RAFGLPAEPFMLLFVGDLRTPRKNLGTVLKALAHLPPNVHLAVAGYLPGSPYPDEARALKIDSRVHFLGL

VKNMPTLMSSVDAYVFPSRYEAMSLSLLEAMAAGLPVVTARTAGGAEIITPECGIVLDDPDDPAALAAAI

ERLARSRDVCRAMGEAARRLMEGFGWARMGAQYIALYRRLRQSSQPSPLAGTEHVVTQERS

>WP_152768918.1 RNB domain-containing ribonuclease [Burkholderia pseudomallei]

MNERREYRVNVFFEESGSFKAGSVLSRQGDAFQVELPGGRRAKVRAKDVLIEFEKPAAGELMQQADEAAQ

QIDLDFLWECAPAEEFAYAALADEYFGASYGPVERAALVLRLHGAPVYFRRKGRGQYQRAPEEQLKMALA

GLERKRQQALVQAGYEEELKAGRLPDAFAGKALGLLTKPDKNSIEYKALDAAALARGVSPARLMLDCGGI

PSARALHEARFLAEYFPHGTGFPAVAVGKLPEDLPRADVNAFSIDDITTTEIDDAFSVEHLSDGRVRIGV

HIAAPALGIVRGDAVDAIARARLSTVYMPGDKITMLPDDVVDVFTLKEGDYRPALSLYIIVKRDTQEIVA

NETRAEYVYVKSNLRHNTLDELVTEDALAAGTGDYPHKDDIAALWPLAQALFERRQVARAGYGLKREVQR

NTDYNFYVEGEHVSITPRRRGSPLDLIVSELAILANSTWGAFLHDHSVPGIYRTQRAFGMPSGPKRTRMQ

TSAAPHEGLGVPQYAWSTSPLRRYVDLVNQWQLLACVQHGVTAKLAAPFKQKDADLYAVVQGFDDTYAAY

ADHQRRMEYFWCLRWIKQEGRKQVSATVVKGELVRLDEVPLLLHVPALGVHARGTRVLLDVMSVDELTIE

ASVRLVSVLDAPMVSSGEPADEDEDADAADETLLDAADESAQGEAEALAEAGGAASGNGESSANGEEQSK

>WP_152768859.1 LysR family transcriptional regulator [Burkholderia pseudomallei]

MAGLKSAIRLIDFPRIVNNSPNLDDLRVFSVVVRLASFSAAAEQLAVSPAYVSKRVALLEKQLGTRLLHR

STRRVAVTEAGERVYAWTEKILDDVDHLVEDVSTTRSVPRGTLRISSSFGFGRHVLAPALLGFNERYPQL

NVRLDLFDRLVDVAGEGFDLDIRIGDEIADHLIAKRLATNYRVLCASPGYLARYGTPRQLADLGAHQCLA

IKERDHPFGVWRLTVRGETSTVKVGGALSTNHGEVAVQWALAGRGIVLRSIWEAGPLLASGELRRVLPEA

SQPANVWAVYPARLAASAKVRVCVDFLVDAFAHLNERANGG

>WP_152768796.1 ATP-binding cassette domain-containing protein [Burkholderia pseudomallei]

MRIRIVRSVSSSSESLLELRDVDFGYGERLVLSNLNMRFARGQVVAVMGGSGCGKTTVLRLIGGLVRARR

GQVLFDGADVGAQTRDGLYALRRKMGMLFQFGALFTDMSVYDNVAFALREHTDLPEALIRDLVLMKLNAV

GLRGARELMPSEVSGGMARRIALARAIALDPQLIMYDEPFAGLDPISLGITANLIRTLNHALGATSILVT

HDVPESFAIADYVYFLANGGVLAEGTPDALRASTDPSVRQFIDGAPDGPFKFHYMSQPLAADFGLGGGRR

>WP_152768765.1 aminotransferase class I/II-fold pyridoxal phosphate-dependent enzyme [Burkholderia pseudomallei]

MSLKCLSAGWGRRRNEAYARGETGREHTLPERKRAQFLVMVRCVNKLYSCISTNGPAGRAVKRYEQLADD

IEAMIRRGVYRPGERIPSVRQASVQHRLSVTTVVRAYLVLESRGAIESRPQSGYFVRARQDAPALALHAS

APVAVSSAVDVSRLVLSTLRSIARDDAVPLGSPYPDATPFPAQRIARHAHAIARRRARWGVIDDLPPGNP

ELIRQIARRYLEGGVAVDPGEIVVTIGATEAINLCLQAVAKPGDTIAVESPTFYAMLHAIERLGMRAIEV

ATHPGEGIDIDALARILATERIAACMVMPNFQNPLGFLMPDERKRALVELLARHDVPAIENDVYHELYYG

AARPSTLKAFDARGLVLHCASFSKSLSPAYRVGWAMPGRYRDQVEKLKFLNTLATPAIDQLAIAEYLRHD

GYDHHLRRIRKLYAQQASMMAALVRRFFPDGTRLSQPKGGYVLWIELPDGVDAMALYRLALAQKITLGPG

HMFSTTLAYRHCIRLNYSYAWSPQIEAAVKTLGRLAAVCAKR

>WP_152768709.1 MerR family transcriptional regulator [Burkholderia pseudomallei]

MAFGRPELHCANSSPLEVKRVGNLDIAEVAQRSGVPASALRYYEEKGLIASTGRRGLRRTFDARVLERLA

LIALGRAAGFSLDEVASMFDAQGRPNIDRALLVAKADELDGAIRRLTSMRDGLRHAAACTAPSHMECPKF

RRILRAASDARRARKKAPPPGG

>WP_144399182.1 DUF3579 domain-containing protein [Burkholderia pseudomallei]

MVCRTQSTKASADSTARLRTCRCPPHARSGALVASRRETASIVFEVSRRGCASALLGRHLGTLMAETLPT

EYFIQGITKDGKKFRPSDWSERLAGVMSCYGPGASGPNARLQYSRYVRPTLLGDLKCVIVDARLRDVEPM

AFDFVMNFAKDNNLVVTEACELPDYGKTQE

>WP_124518673.1 NAD-binding protein [Burkholderia pseudomallei]

MTMSRNVGVIGLGAMGLGVARSLLRAGFRVHACDVREDVLAAFAAEGGVRCATPAELGALCGVVVTLVVN

AQQTDAVLFGEQGAAAAMPRGGVVISSATVAPDFAAQLGARLAAAGLLMLDAPVSGGAARAASGEMTMMT

SGPAAAYEACGDVLAAIAGKVYRLGDAHGAGSKVKIINQLLAGVHIAAAAEAMALGLREGVDPDALYDVI

THSAGNSWMFENRVPHILNGDYTPLSAVDIFVKDLGLVLDTARRSQFPLPLSATAHQMFMSASSAGHGGE

DDSAVIKTFPGIALPARR

>WP_122806868.1 response regulator [Burkholderia pseudomallei]

MQGLLQELDGSPLRKFYSLESNLKRERRVFTIVIVLLVCAALSIAAMTVTGLFQTAFRQEEQSARIHEKE

VVDVFLQRRMMLTTASLVLQLRMNGAPSALNVPAPNACTPMAHNVRDDAILRESCDYTVQLLSNSGQTPS

VEMVTADGSVGYGYLFPTGDLSALRSSTPSELVSAVLERYGKRGLDPLEAARKKRILWFAVGRGGRGEEL

HMIGASVVFKDERLYALVLTSVDLHSLVSPIERAGRVQQPVVVDSEGVPLVNADDAETVRKVDGRLAGQQ

DGLYHWIPGFGWALRRPAPFSGFGHMTYLLPLDLQLRSMRYELSLVGGATLVLIVLLFVAFRYWNYRFLT

RIYEEASRALESEMLNHLLVHATPVGLCIVRRATLEIVVANPIARTMLGLRLSDRHLPQELLSAFESSLA

EQDTQSDDARIFQFPFTLSRAGHAAVHIEITYAPAMLNAREVFFCAITDMTAHHQAEILLREAKLTSDAA

AKAKVAFFASMSHEIRTPLASLVGNIELIARGPLAPEQQARVKAMETSARGLMQIVNDVLDFSKIDVGEL

SLMEEWSNIAELLDRLALSHAPLATQQGLKFYMVFDRSLPARLYFDPIRVSQIVNNLLSNALKFTPSGKI

VLRAGWRAGALEISVTDSGIGIPDDLKHRLFLPFTQGDSNRLRQARGTGLGLSICARLCELMKGRIDLES

TVGVGTRIAVTLPLGVSEADSSDAYWTLPYRRVAVLGRAQENLEWLANLFDPGVTAVTAFSRPAEPIDAH

AHDFLMVTDEFSPAEVLPWWRRPDSIVWVGQAGPLVPRRRDDGGVEISMYSLAGLKSATHMLAAGRTALA

EAGHEPPGAEAGMTVLIAEDNLLNRSLLLDQLTTLGVRVIEAKNGEEALALLLKEPVDVVMTDIDMPMMD

GFQLLAEMRRLGMTMPVYAVSASARPEDVAEGRARGFTDYLAKPVSLERLETVVRACCSAPAGARADEDA

QDELPGLPDVPPAYASAFVAQAGSEIAEFDAILRERALPKLRRWLHGVSGGIAVLGPSALHEQCQELRAY

ARESGEWNREIELQALAIRNALERMVAALTSA

>WP_080248721.1 EAL domain-containing protein [Burkholderia pseudomallei]

MAFVPSGPSCDVNARCARFVRSAVAVALAVALLGAALLSVERFGAARSRASAAIPASIGARLSGCAFIAV

RIGAALSRRVPSPAHAAAPGVARPDFDAIERVFGPIRMRERRAHGATCTADAPDASHAGWIEPARVCSAA

AHAAPGRGAEGAPNTMPGPSEAGAAAVLQSQPYLDMIDSTFREPAAIVLALSLLGMIALALLPVFRIRRL

ALRLGEAQGALEMSEARARAALVAVGDGVIFTGRAGRVECLNPAAERLIGMLADDCRGRPLVSTLRLSRT

ATSASSGAFAASAGLDAPDSPGSAASPDAPLGDLEDGGSCDATLYRADGGAIAVRATASSIAPPPGHARR

ACGSGRVLVLKSLATEHELVRRLAWQTTHDPLTGLASRAEFERHVGFALAADVREPVALLFVDLDRFRIV

NDTCGYAAGDAMLAALAARLVSCAASADVVARLGGDEFCVLLDARDEASAVSAAERLRASVDGFVFVWDG

QPFSVTASVGVALLGGPGRAPRVEDAVRLAGIACDVAKARGRNRVQLADPHDHELAHHISDVSWCARVRQ

ALEYDDFRLYVQPIVDTATQGATGLPRARRGELLLRMGALGEREGVAPPGLFIRAAERYGLVTDIDRWVV

RTVLDALARTRSRRFSEYAINLSGISIGDERFLDYVLEQFARTRVAPALICFEITETAAIANLAGALRFM

HELKALGCRFALDDFGSGMASLSYLKQLPVEYLKIDGSFVTGIANDAASLDIVASINDIGHAMNCKTIAE

YVDSAATLQKLAALGVDYAQGYYIGRPVPWCEAACA

>WP_080248676.1 LysM peptidoglycan-binding domain-containing protein [Burkholderia pseudomallei]

MAGLNDVIERLSQGTQNVLTVHLGARITKVRDYLNALDEIRVFTANSQNFGHQSSSVNILRNLIRMGAPG

PYTFALSASNSADYADLEEKIRLLIPQFRQVGVTFELGAGGRTADVTVVRLDKALAPAQFAISGGFDDLE

NKTPPYHLLNVTNYVQLQPYAWNRGTNMVRIMPPGGTASEYNLDELNPTTLLARRAFYLADPELTQSDRE

AIAQTPYANKARVIERLLERREAGEITLFPVYGVTTKGSAYTSLYNAVTGALIAQNTHPAVKKTVMVQIT

TLTASEWEAFLFLMRDPAGQMVNKIRTTPDFRGWNEENKVKDRVQDLGSPNSVPTVEQLDEELSYLEDDQ

LLVVYIGKIPAPLFDALYASATLPPVLEGQNTAELMLNLGKPYFKITSNNSREADARFSYATLPLSSTGA

GTDATNSLDESFNGIYFTRPDNWYRDRPTYPPTQLPAMINAYVQPAGNARATYFAAQRTFFHDELNDKLL

RGLDLFVNLIGPAALEEHRLALAHAELDDANAPVHEAGAAARAPARIAHRKPPTRVAGGDANGASELLEA

FYEDLTSHTVDGVLDFLLAVTDGILNEFFRQVVIDVVFTITDTVTEINADKTEVTLTGKSKAFGAGNLTL

AFSFTDNGGTIAGKMSGAFTDTVWAFPGAQWLSVANPSLALAIDSNAAVPVTGTVGATFTAGIAAKASLT

LPSEPGRLLLQAEFLTPRPSITNIFQMLGGINIQALLPSQIQFFSDIEVQNLALRYSYANGVMEYIGVTL

GTPENRSWQLVPGVTVTGLSFSALTDYPGDLQRRSTRYVIGGRFDIAGGHAQLEARVPALRVTGGLIDGS

PPITLAAIVTEYLGADFAAAIPASVSSTAIEQLSFMVDQAQGAYSFSMDVSAQWPVPSAANALFTITGLN

FAIDAVSRDINPPKADAGGNNGAGGTQTEIEGSFGGSLIVLPNSESPIGLSTTATYKTAAKAWTFDAQQT

SGVVSLGALLVYYLGNTWQAPQGQEYAIDGLGLTITSSPTDSTWAFTGKTADNWVVPFLDVSLAAKLRMG

DAGAKAEVPGKFGRLDLEVIWQNIDLTVWFDYNPKIKQYGITWGLLEGVVDGPDPTTQDWTATLGFKQNT

TLGSMIETMVSWATGSKFGLESPWSFLNAIPLSNLALKYTFNQTTPSRNKVSFAVTIGPINLGFARIDSI

DVGYQSTGEDRGVMVTLNGSFFWQSDPSTPLEWDASKPGTAPAPPGNGNKYLDLRLLAMGQHITLPCFAT

ADTVQKAIACMATLPDPKPGQIPAVRFDAQSAWLIGTDFGVLKIDSGQTGNNANALRVTNDGSALAESSG

YVLTLQAVFNDPHLYGLRIALDGAAAKVFKGLDFQIMYRQVSDTVGVYQAEITLPDLMRHLTVGAYSLTL

PVFGIAVYTNGDFQVDIGFPWNENFSRSFTIEAIIPPGIPVLGSAGFYFGKLSSASTNRVPASSYGTFNP

VLVFGFGMQVGFGKSIEYGILSAGFSVTVVGILEGILAKWNPYQLTHSGREPSTQLQGDYYFWLRGTVGI

VGRVYGSVDFAIVKANVDITVKLLLQLTYESYVSITITVIASVDVSVSVKINLGLFKISISFSFSMRLKE

TFTIDNRGAAPWLGDGRNVRGVLRLPVERRLSGFARAQARDSLLVSAPNWGNLRPGGVTDLSGYLVPGLT

AARDEWTPQGEPANQLSCWVALLLIESVPPAGQDAGASKLKAAGSAPDSSFEALAKMVLRWAIAAVQGPM

TPDEVDRCPVPATLLDWLADDVLVSTGDDPTPIPLDAVQAFLDTHFRFNLRVPPTDQDASADTAYFPAPP

QLRVMIPPYGNDYPGVQYTLGSYNALGENTLAELRAWFDQLAVQVEREQAANGAAARAFVEEAPLSMAGW

MFSDYFLLLARQMVKAAQDALRDFKYALDANETPDDVVSWVNTTGQLNGLYTLNDVFGANALHALVAEKT

LTIGVTSSISLAKTGQTFTSLAKAFDDALPASAIASANAADAALLQPGATITYPGFDPYTSVAGDTLVSI

AAHYQAKLNDLLADSDVLDAAGMLRIGASALMPYTAYTALATDTFASVAALPVYAGGFGAAALATANAGR

SVLLEGVKIEYPDKDAYTVQPRDTLGDVANAFGVTVSDLLATSAVLTQPGLLAPVASLTVPAFRYTTQQG

DDLAQVAARFGVTVSVLADQPANGTVAGLFDTGDTLDLPHLPQFPLAELLAEAQRSGMLQHLSGIASSYT

MHGLRFPTSGPTGTGGQWSIVPNEMGMWVHDVNGTLKLPPQAGLYALTGQQFPLPALGADPFAATFDSVA

GAGSSWLRFVDGNGGPTDRLTLSVTPGTPDATRIAQVTAAAKTRLVVPMDMLGAGKMYDTALATYPFTSA

LQWLSTNTVALPYGQPPAGVQSLRVWQLPGALAALPDPATHAVNPRFALRVARYDDATGATETTGVDSYG

WASTIGFTVRRIPPVAGSPASVDTYEVVGASGAAIVVLEQLLSQVQADDSAYFGLSVGFAPDSATGGGEG

VQTGGAASVVFGIAQVNLSTETRPPAGAAFAALRETAGETPQLTLLNSPSEFVRLLWEASITRSGGFFLY

YYDRAAGGGLPDRIFNDRNEASLTLIVLYAKPAAVDDQDRVTNYMNAVVTTDALDTGNAVLFAEAAPVPA

TVTSGAGETLASLAAQWYSDEADIAEANANVALRAGALVRVSEGVYQAPPGGIALAQVASRFGTTVQALN

DANPLWGGLPDPLPFPAAIRVPDLTLTAGTSAHTASLADIAGWYGEPVDALASHNARVAQLFAAGVPLVI

PGGPRVRSAAVQPGVQALAALRPAPPQVDGTSPDYGTELLLNNFSLLNQQVYGNVDFRPSDPPGLPAGPT

TKAPEENGNDKVRTVVPADQVEAWNFSQALPYARFAKHVPQAPRAAVALPPASASPYFGVGGILQISFAW

QDYYGNVLSTPLSDPLAGDAAPYNDAPLLTGYTDPLVSLSQWPSIASNWQVLPGSGGANPRLNIELSFDP

SRYQGLLQASAATQTTITVVFTDALDAASVGELSRWQLVPGTVDSASLAADGKTVTLTVPALDDDLRYTV

IAADIKAQASDMRYSGQASFDWPDNPVTRSSTVQQNASQDLHVYTQLYYQLTDPAGVDLSAQSSLLADAH

GAPGSVAYAPAAVDKLMDWLFGTAGAASSVYAFVLDRSKFQSVAVPPAAGLPLDVDVPPQQVNTAQIFPL

WTSFTMTRAHGPVLPGLETVAGIRSASTRVAPLQDALGATGGTLGLVTFATGFEQALSTPGSVRLKVATG

VDRTAPPATGAASTVWAVRVGLAAGKAISYAIADAGNPAVFAPQPASNRLISRTQVPIYDYTTGKGISST

PSRTTDFTDVDLDTWCAQVFAAVDDVLTPQFTAPMQIVGELKSADYLQSILDGKKGLATVAKLWMIPVFA

GETSDPSAAREAFYQQLLVRLSAAYTTRAAVEFHANVTADVIEPAADQPPRLFGPVTRNGPVFEAANVDG

QALTTVFLLFSDPMDPVTAGNIENYALNSGTGVLTATVDRGTVTLTLATDVQPGQTTVTVSNLKDATGRA

VRPPLTRTVTTGSASLPASTLAFSSPKLTLQAGDTRALTYLVNAPDSVRGAGGEIVSYVELDMTYQGSQI

EHQIGALPGIEDYQASTWLSFVVPDTDGPLAADLGNFAVPLVLRAFPASPAMTEQSGTPTHDLDTASLPL

LKQWDYAFTYSLPFHYPQDRIYGEVEFNLRTAPTLFASFPDAFAQLAEFITVFPKVNADLQTILAGIDAT

VDPMTDQQKIDDASIALQSFIQLVDELVDAAGGNTQGNGERRGGTGLTFQAPARLLTGDPSLTFAFYEEE

GSAEVGDTEGALVVTLVGAVPAGMGQPVVEIDPALYDAQPWQPPGDTQKAGDVFHYVYKRKAGPGPEGSY

LSAANGQNIPGRTVRLPALDILQRQDAWSTVWVERNRELVPGKPSADAFVYTTPEVRFASPLYPTNDANA

IIDVAAIPSGTPVKRSLQEHFDALFAYLLAGDTLPQIVAQVEVTYGYALNAALDKIVLPVLMQAPLTVDV

AGTGAGTIAKMTADWTAAIETWFSTYEPTGGGTLWMDLTLMSNLTGQPMPLLRMRRLMLSIAQVVPPLPC

R

>WP_080248664.1 c-type cytochrome [Burkholderia pseudomallei]

MSVKPAQRPERGFAKTRRRTAAYRPAATRAAGVARAAGAATPPLPAAPHAAAAHAQAHAAQPAALPLAYV

FDSAGPAPRPVLILGWALLALCTSVCLVIAVLPALALFLGRPASVGLTERGGLGFVYVGSAISTALLLAA

LVYMLWVLAAVAKPPRPPAVTIAVTAYDWWWKADYGGGPPDGFTTANELHVPVGEPVLIELRSADVIHAF

WAPQLAGKTQAIPGQINRQWMQADRPGVYRGQCTQFCGAQHAQMGFEIVAEPPDAYRRWYASQRRGAEAP

RTADALRGQRIFADRCAGCHAVRGTGAAGTQAPDLTHVGARRLLAAGALANTPDELRRWIADAQQVKPQS

LMPSIRLDPAQQRDLSAYLATLR

>WP_076865154.1 family 10 glycosylhydrolase [Burkholderia pseudomallei]

MSMLSTRLRHLAAAALVLSASACASSPQAVPEVACRPDETMPKRQFRGTWIASVINLDWPSRPSLPAAAQ

QAELSAWLDDAVRMNRNAVILQVRPTADAFWPSPFEPWSKYLTGAQGGDPGYDPLAFAVAEAHRRNLELH

AWFNPYRVAMDDRLDALVATHPARAHPDWVVRYGGKLYYNPGVPAARAFVVDAIMDAAARYDIDAVHLDD

YFYPYPVAGATFDDASAYAQYGTGFATLADWRRDNVDRLVESLAQRIKAAKPWVKFGISPFAVWRNAATD

PQGSRTSASVQTYDDLYADTRRWVRERWIDYVVPQAYWARGFAPADYDEVVAWWANEVRGRDAHLYIGQA

AYKVGTSNQSPGWSDPHELSRHLAFNLTAPEVKGDVYFSAKDVRADRLGATTRLNRTWYSRPALVPTMPA

LGGNAPPSAKALRAQRTPDGVRLQWQAGSAAAASYAVYRHALGRQDMCADSDARHLLATVRGTQYVDVTA

RADRDYRYVVTALDRLWHESEPAYVAMPAAKPR

>WP_076852884.1 methyltransferase domain-containing protein [Burkholderia pseudomallei]

MRIDRIGAFQFHPIQPIKADIVLRRGLCNLKTRKGVAMQNVDRPTGSSLAGAAILLEIGAAYGIVDFIRH

SNGFDVAALAERSGIGEPMIADYVDAMCEAGLVTLAKAGERDIYRPADDFPDVVNDVGYLSWALLACAPL

IVNAREFAADNALAQARHPRDGGLVARTSKWMGELSFYPQPERAILKLRPKKIVDLGSGSGGFLIRMLHQ

LDGARGVGVDLSATATEQAKQAAEANGFAGRLEFLHRPIQSLVDDPAPLKGADVVHAGFVMHDLLPAEER

TLDALLETCREHASSGTVVIVDAVPLAPDRWERSFAAAFNHLHRNFMSRRLLSEDAWREKFEQAGFGNVA

VETLGHPGGRMILGSA

>WP_076850621.1 DUF3596 domain-containing protein [Burkholderia pseudomallei]

MKLNNPAGFFFTLFKKPTAANSPPDPASVMAAPIPRENNIAIDDGRAVVRDSLSSAASVAATITGKGARC

RERIRPEPTSANIRAAADFLGAVRTAITNGTFDQPAERAAPGGLQAAREARRAPTATASDREHYAEFGDK

MSADDAKHVETESIEAAVFRKAVCAGFDHKMVALALMKRGGRSAANRRTPTCQSGMAADSRNIGEARCAR

RLCSQSLLFPCPRGALETETLQGQSIVTNCAGGAYQFR

>WP_076823475.1 DUF1571 domain-containing protein [Burkholderia pseudomallei]

MSHTLPLHPSSSSRTRFARRRLATASAALSLSLSLMLGHGAAHAQVNAEASAAVESSAQEAALPAELAKV

AKLPVEQQARWLRTAARQGTLEKLDDATLTALFKSLDPQTVPDYVAAGPIGHPSYEFTMLRQERISGKWS

DTPDHMLVKVTRSPLRVYAKWLPDGAHSGQEVIYDSAKRADEMYGHLGGLLGKVPMWTAVDGTLARAQSN

HQVRDLGTEFVANLYLTEAKKYREAGALKPTHVEAKTVKGVRVVALTYETPGGRPQFYAKKETLGLDLRQ

PYFRTVESYDNDGRVFEKIVFEKITPKSLDETAFDPKNPDYKF

>WP_076823272.1 alpha/beta fold hydrolase [Burkholderia pseudomallei]

MNERARAMTGRTGILLIHGLGGTQYDLGSLHKAMRRAGGDTHMITLPGHGTRPEDLVDVRAEAWLDAVTA

QYRALEREYDTVHVAGMCMGALVARLLCRRVRHARGRLALLAAPMFIDGWSTPWYRSLRHLLYRVPGVPA

RLRVDEDEPFGIKNPTIRAIVKKKFERQDNFHYPWVPLACIRQVDRLRSWARAAAADTHCPTLVLHARED

ELTSLRSADFLLKHLPDARGIVLENSYHMICADNDRDEVARQVLTFFGFDPSHAMSPAMARRMGRADPRA

>WP_076802643.1 tetratricopeptide repeat protein [Burkholderia pseudomallei]

MIGMYEQSKAAKRRYRDGDFASRYFVGDGIDVGAGPDGLGRYRRQFAAMRSVREWDLADGDAQLLAGVSD

NTFDFLHSSHCLEHMRDAVEALHSWVRVVKPGGYLVICVPDEDMYEQGVYPSRFNPDHKWTFTIQKPQGA

SWSPRSINLLEMLIPLSGVLEIERIQRVQEFFDDTLHGRDQTLMPNVECAIELVLRKRGAAGAIMGDVRP

AAAEGAAAMLPCARAGTGRFDAPAGREALMLLDSGHRDAAERKALEALVREPADCTAMDVLERLYLDSAR

YAECADILEKLVAYAPNNAGAWNNLGCCYGELRRFPQARAAFQRAVDANPEYVTAYGGLAAMHYALGDPA

SAEATYRAVLARSPEHVDSCVNLANCMLAQEKVEDARPYIERAGEKLLPDLNKPKDVALCAPREGLTVPY

RSRIENRPIFESMHARLGMSLLMLGRATEGLRLLEWRLGSAFVPGFDARRAQRMWRGEPLRGKSLLLLWE

QGYGDVLQGLRHVSRLAELAERIYLPADAAVRELFEASFSAWGQKVVVCDWRDEPPAFDYYVSILSLHHR

MRELGVACPEAVGPYLSCNPAEVERFRSYRLDENALAVGVIYAGKSEMLLDAQRSLPESVVRDLTAPIAG

VRFYSLQIGCRAADCAMLGKMNGVIDWSMLLSRFADTAAAMRALDLVVCVDTAAVHLAGALGVRAMLLNR

FGSEYRWQLDRDDGVWYESVSQVRQHAMNDWTNAVACVRSRLRQEALDLGRDAVVPGAQFSASEAGQPDV

HRQLRAIEQALGNSQLDEARGLITGYLAQHADSPKAQCHAGVLELLSANPDAARAYLDRAIRLRPDYAVA

YSNRAATRKEGDAEREGDIIRSIACDPSFHAGWLNLARIRRPHDAALALQCARRAVQLAPDDADAVLLRA

ELALDTGDFDEALSQFERLREQRPDAPESYANLIPALAALERRDDAIAALQRALELNSKHPGALNNGVQF

YLRTQQYDKAMELAQRYVGAHGELASAHTMCGLVYHNLKAYDRAEASLRRALEIEPHNAEALFALGTVLE

RVDRLAESEEVLRRALTIRRDYRVLVNLAVTLNRQQRYEAARSLNDEALAIGGGKMKSELKPRMALMRKT

FAGQPFRAELATVDMTFGPDWNNALIALVQGDYVRGFAAYELRQEHQTVSGMTADEYRALLWRSEPLAGK

RILLLPEQGYGDVIQFLRYVPELKRKGATVLVGASAPLRRLLEVAPGVDEVVQLDDARRPAFDYVCPLMS

LAHRLDVSSAEDFLPSFPYLRVPEAASQTWRARFAADSRRKIGLVWAGRKIYPADRLRSVPLPEMRDVLS

DIPDVQWISLQVGDCASEAASWPAVLDVSAELTDFAETAALIESLDMVITVDTAVAHLAGALGKPVWMLN

RISTDWRWGQEGQRCGWYPSMRIFRQQVHLSWAEPIGELADALEQWLDVERLNA

>WP_063929575.1 transposase [Burkholderia pseudomallei]

MMLLAAASRAHARIPNDGTKPARRRLRLRLPRLTGVSSASRIACAFACTFSPISRRGPTMQFEELSDDEW

ARIEGLLAAQPVRNTRCGRPRAQARAVANAVLWVLTTGECWAALPRRYPSSPTCRRRFDEWSADGTLAAL

IERLRGAGRQVELRERIGAIDAKPHARARRRFQHNAFWSSPESWCAPAVAVVGGVIEAGRDDCVPEHGEG

NAN

>WP_052113886.1 TonB-dependent receptor [Burkholderia pseudomallei]

MDDHRRIAPPFARRLHPLSLLLAASLAHGETGAPPAERRSDAPPATALAPIFVTANPLGASALSSPTASL

SGDALTLRRTDSLGDTLNGLPGVSTTTYGPLVGRPIIRGMDGDRIRLLQNGVAAYDASSLSYDHAVPQDP

LSVERIEIVRGPAALLYGGNAVGGVVNTIDNRIPREAITGVSGALDASYGGANNARAGAALVEGGNGRFA

FHLDAFGRETDALRIPGHAHSARQRALDGEDASEPYGKLPNSDGRRYGGAAGGSYTWADGYVGASYSGYE

SNYGSVAETDARLQMRQERVALASEVRNLRGPFSQLKFDFGYTNYQHKEIEDGMTGTTFRNHGYEARVEA

RHRKLGPFEGALGVQVGQNTFSALGGEALAPTTRTTSVALFGLEQWQATDALKLSAGARIEHVRLDPSAN

GDDKFGFARSRDFNAGSVSAGALYQLAPAWSLAGNVSYTERAPTFYELYANGPHGATGQYLIGRPDAQKE

KAVSTDLALRYASGPNRGSIGVFYSRLRNYLAEYDTGRLVDDDGVPVAPGADDALREAVYRGVRAEFYGV

ELEGRWRAFERRGHRVDLELSADYTHARNADTGEPLPRIAPLRATLAADYGYGPFGARAQLTHAWAQHRV

PEHDLATDGYTSLGVVLTYKLRVGATNWLAYLRGDNLTNQDIRYASSVVRNIAPQGGRSVSIGMRTTF

>WP_050040888.1 HlyD family efflux transporter periplasmic adaptor subunit [Burkholderia pseudomallei]

MPIRMNALREQKTPRKLTRGTTFGTVINGVVDVPISMRFFCYLSIMMFAMFIIALVRLTYANTESVMGML

TPRSGLIGVGAPPGWAVREVFVAKDQHVKAGQKLLSVTRDTSFVSQANNVQGMRESIKRQRVEVGQQIDA

AKLEYQSTIQQINQQIAAFDESRGLIDKQIQDQKRIVSEYQERRDRVKQLLNEQVVTLEQYNQVNTQYLQ

ASQAYQDLMLRRADLAKNAMKLRGDLETVQSKYDGSNAELKIKQEELNSKEYNIDESVNQVLYAPADGQI

VRLDVVQGSVIDPPGTRVVEILPAKADGLIAELYIPSSKAGFVKPGQEVKLAYGSYPVEKFGTYRGKLLS

VSPVAFTAKELNLPADNGAPQTYFKSWVELVDRKPAFEGKALSLKAGMTLRADIVLEKRTLLEWLFEPLY

RIRQRIFGTPA

>WP_044365864.1 DUF802 domain-containing protein [Burkholderia pseudomallei]

MSRFHVHLVVFFAGLAAVCWIGAGYAVSNPVALAVTLVIAAGYLAGALELRRYRQATSTLAQAVAALSEP

PAALGAWLERLHPSLRHAVRVRVEGERVALPGPALTPYLVGLLVLLGMLGTLIGMVMTLRGTGAALESST

DLQAIRASLAAPVKGLGFAFGTSIAGVATSAMLGLLSALCRRERLDAAQALDAKIATTLRVHSHAHQRDE

TFRLLQRQADLMPTLVERLQAMMHSLEQQSAASAERQIAGQQAFLGKAEETYARLASSVGQSLTDSVAES

ARVAGSALQPVMETTMAGLARETAALHDALTQAVQRQLDGLSAGFETTAAHVADVWRHALADHQRSSDAL

AQRLHGSIERIVESRDEQRLATWTDSLGSIAAKLDTEWEQTSAQAANRQQAICDALAHTARDLSAQATAF

EQHTAALLRAMSESHSALQATLESRDEQRLATWTDSLGSIAAKLGTEWAQTSAQAANRQQTICDALAHTA

RDLSAQATAFEQHTAALLRAMSESHSALQATLESRDEQRLATWTDSLGSIAAKLGTEWAQTSAQAANRQQ

AICDALAHTARDLSAQATAFEQHTAALLRAMSESHSALQATLESRDEQRLATWTDSLGSIAAKLGTEWAQ

TSAQAANRQQAICDALAHTARDLSAHTQAHASATIAEISQLVQAASEAPRIAAEVVAELRQKLSDSMVRD

TAMLEERNRMLATLETLLDAVNHASSEQRAAVDALVATSSALLQRVGTQFTDEVGTQTDRLGGVAAQITG

SAVEIASLGDALGAAVQSFGESNDKLVAHLQRIEAALDKSLARSDEQLAYYVAQAREVIDLSMMSQKQII

EELQRVGGERASAGAAAA

>WP_041198119.1 c-type cytochrome [Burkholderia pseudomallei]

MESRVSSRRLFSPLLAVVLMGAAGLLSTAHAQTKPTEPAAAKAPLKAPDTMAERVRGCTACHGTHGQGTD

NDYFPRLAGKPAEYLYNQLVNFRDGRRKYPPMNYLLTYLSDDYLREIAQHFSDERPPYPAPTKPTVPAAV

VERGKQLALHGDPARKLPACVACHGNGLTGMQPAIPGLVGLHSDYLSAQIGAWRSGTRHAKAPDCMHDVA

SKLSDEDVTAVTAWLAAQPAPANPVPAPARSMKTPLACGSEPQ

>WP_038792201.1 MFS transporter [Burkholderia pseudomallei]

MPSALYAFIAPLIVACALFMESVDANIIVTALPAMARDFGQNPVTLNIAITSYVVGLGVFIPICGWLADR

FGARTVFRTAIGIFVAGSLLCAASNNLELFTFARFVQGVGGAMMVPVGRIIIFRAVPRTELVRAMNYLSV

PALFGPAAGPLLGGFITTYLHWRLIFFINVPIGILGIYLANRHIANTHELDPGPLDGFGFVLSAAGAALL

LMGLTLLDGALITRGAALAMGATGAALLGGYVLYARRVERPVLDLRFLRIPTYHASVVGGSLFRIGLGAV

PFLLPLALQEGLGMSAFHSGAITCASAVGGALTRMLAPRTLKRFGFRTVLMYNAAFSGLAIAAYGVFHPG

MSTLAIWLIVLVGGIFPALQFTSLNSMIYAEIAARDAGRATSLGSVVQQMSLGLGVTVAALVLHVSHWAQ

GHPAMVWSDFWPAFVVVGLCSFASIPITRRLSPNAGDEVARGKRG

>WP_038787697.1 MMPL family transporter [Burkholderia pseudomallei]

MLTSVLVRLVAWSVRRPIWVVVLSLVIAALSSVYVAHHFKINTDISKLVENDPKWAALGRAIDDAFPQRN

QTILAVVEAPAPEFAGAAADALAEGLRRETDAGRIGQVSEPAGGPLFEHDGLLFLPEQDVATTTAQLASA

RPLINVLAKDPSIAGLATTLSTTLGVPLQSGQVKLSGMAKLLSRSAATVDDVLAGKPAAFSWRALVDADA

AREPARAFVTVQPVVNYGALKAGEQASRTIRATAQALKLDERFGAAVRLTGEQPLADEEFASVQDGALVN

GIATLAIVLVILWIALRSKRMIASVFVTLFVGLVVTAALGLMMVGSLNMISVAFMVLFVGLGVDFAIQYG

VKYREERHRDPNLDHALVGAAHAMGMPLTLATAAVAASFFSFLPTAYRGVSELGLIAGVGMFVALFTTLT

LLPALLKLLAPPGERKPPGFPRLAPVDDYLDHHRKPILIGTLAVVIGALPLLAHLRFDFNPLHLKDPRSE

SMATLLALKDSPEASVNDVSLLAPSLAAANAAAQRLGALPEVGRTTTLSTFIPDAQPQKLATIAAAAREL

LPALTQPSAAPVPDAQRVAALKRASNLLEYASEDYPGPGAAAAKHLSESLAKLAAADAATRERAEHAFSV

PLKIALNQLAMLLQPSEITRENLPPQIVRDWIAPDGRALVQISPKVVKGADPGDDAMLRRFAKAVKAAEP

GAIGGPISILHSADTIIRAFLQAAALSVVSITVLLWITLRRFGDVLRTLVPLLVSGVVTLELCVLLGMPL

NFANIIALPLMLGVGVAFKVYFVMAWRAGQTGLLQSSLTHAVLFSAATTATAFGSLWLSHHPGTASMGRL

LALALSCTLIGAVVFQPVLMGKPRTKRVTNQSQGIDE

>WP_038757498.1 DEAD/DEAH box helicase [Burkholderia pseudomallei]

MSDSVAKPVDATFDQFGLAAEILKAIAEQGYTTPTPIQAKAIPVVLSGRDVMGAAQTGTGKTASFSLPII

QRLLPQANTSASPARHPVRALILTPTRELADQVAANVHAYAKHTPLRSAVVFGGVDMNPQMAELRRGVEI

LIATPGRLLDHVQQKTANLGQVQILVLDEADRMLDMGFLPDLQRILNLLPKERQTLLFSATFSPEIKKLA

STYLRNPQTIEVARSNAAASTVTQIVYDVAEGDKQAAVVKLIRDRSLKQVIVFCNSKIGASRLARQIERD

GIIAAAIHGDRSQSERMQALDAFKRGEIEALVATDVAARGLDIAELPAVINFDLPFNAEDYVHRIGRTGR

AGASGDALSLCSPNERKQLADIEKLIKRTLSLETLALDLPRHRHDDRGGRRERDRDERRGAPAGRRSAGG

ERTHHPRREAPIDDFFLKPYVPSPSANQPDEAKPVQPEKKAPKQPLAALLGGFGMPRKTSSSS

>WP_038732972.1 helix-turn-helix domain-containing protein [Burkholderia pseudomallei]

MEWLHGHLREPSTRDAIARQSAMSVRTLARRFQEWTGTLPLQWLRPARVRRAQPLPQTTALSVERIATEA

GFGLASAFRERFVRIVGTTPKRYRQAFRLQAG

>WP_029671417.1 multicopper oxidase domain-containing protein [Burkholderia pseudomallei]

MNPAVDPSPLSRAAQALALASIGGALALGACTSRPSGERYAPSRMDFNPGPAIVTQTRHTGPFASGSALS

FDAALQPLDAARDQAIRLDTTHTVIRIAPGIAFAAWTFGNQVPGPTVHVKVGDRVRLSMTNRSDEPAPGG

LQLTAPMMHSMDFHAAMVSPTDKYRSIAPGQTMHFEFTPNYPGVFMYRCGTPMVLEHIASGMYGVVVVAP

RDGYPTRADREYVIVQSEFYTKPDPQHRSVGTDALHVLDGERLRRKAPTYTVFNGRYNGMVTQPLIAKPG

ERVRLYVLNAGPSDTSSFHVVGAIFDRVWLDGNPDNQLRGMQTVLLGSSGSAIAAFVVPEAGAYVMVDHQ

FANASQGAVGVIDAGAHEESTIEHHNIPASATPTDAEAIQGKLDFESKCLACHTLGHGAKLGPALLGVTQ

RRSDAWLRRWLASPEAMVASDADARALRAHYPITMPDQNLSDSEIRRYVRYFHWADEASKQRDHAMP

>WP_029671358.1 YbfB/YjiJ family MFS transporter [Burkholderia pseudomallei]

MSSLPSFLAAASGPRGPSHDQRAACRAALAGAVALAVALGVGRFAFTPLLPLMLAGGELDIRHGGWLASA

NYAGYFVGAMTCARIAVDPARMVRAGLAATVLLTFAMGLASPFWVWALVRFVGGAVSAWTFVFASQWGLR

RVVEHGAPAWGGVIYTGPGIGIVATGLIGFALAGRHAALGWIGFAAASAVLTAFVWRAFGAAGAGTDGGQ

SRGGAKRAGDGVGVADAAGRAGERLAAGATGAAEGAEGAEAVADAVSAANVADTADTANSANSANSANSA

NSANSANSANSANSANSANSATATAERNSPVAGVAGQHGAMAAPAARAPHVESAAARRDAFWLVLLYGLP

GFGYIITATFLPVIARAALPAHSRWPDLFWPMFGAALIAGALLGARLPSGWDNRLLLGACCAVQALGVAL

GIVWPTAPGFALGSALVGLPFTAITLFAMREARRLRGERAAGLMGYATASYGVGQIAGPLAAAPLAAHAG

SFSPALWLAATMLAVGAAGFAAVALRAWRAKTRGGGVG

>WP_024429130.1 DUF2169 domain-containing protein [Burkholderia pseudomallei]

MKIVKPLAISPLTRVYRMHGREHLGVAALLIATLGDEPKLLAESALWRLAGDELRGYPLDMALPKACPEF

LVSGYAYGKYASDPHACACEVGVRIAGLEKRLRVCGDRQWAGARITAPRPFERLPIDWDLAYGGAGCADN

PRGRGAHAREGAPRDLPNVEYAHSPMRFAHEQPAPAGFCPVDAAWPARAGLYGALDRQWQEEDCPGFPRT

LDPRYFNIAPADQQLPELRAFPDGARYELTHMHPDHATLAGNLPALRARSFVVRRGSDAPEEMPMRLTTA

WFVPHRERVILIYHGVTPVRAFDASDVQTVLFGAEASGHARPADWYRQVIEWRTRDDRAALYALRDRDLL

PEHALAPEAAATPEPTQQSAKQRQLRERLSVFPDAPRAQTPAPDRLAEFVEQQQALADEKRAALEAMRRE

LATSEVFSVGRRRGPPGRIAPADEDPARHAGASAESPDIRALERDADERLRGLYQQCAQHQDAPARLHGA

AARARRECVASAAAAGQSLQVADLTGADLSGMDLRGARLAGAMLENADLSDADLTGADLSRTVLVRADLT

RAKLVDARLTAANLSLAHCERTDFSGSDLSDGIFEQVHLRDCRFNGSVLASTRFDACRFDAVDFGRATLR

ELIFIEQSFSGVSFSDATIRKMLLMRCAFADVRFSAASIDGFGIVETQASGQLRFDRASVNKACFVGRCD

IGRADFSFATLTEVNFRETQLVEANFGGARIGNCDFTDACLRAADLRGAKAEGSPFVRADLTRADLRDTD

LIAAYLRGAKLDGADLRRANLFRANLSQILTDADTRWQGAYLNRAVRFPLAEART

>WP_024429110.1 MULTISPECIES: methyltransferase domain-containing protein [Burkholderia]

MTEVDAGQTPHAAILQMVTGKWHAQALYVAAELGIADLLAQRERTADELAAETQTHPEALYRVLRALGSL

GVFVEEDGRRFRNSPLGDTLRRDAPGSMRGFVRFAGMDAGWRAWGQLMYSVRTGRSAFEHVVGGPGFAYI

DSHPEIAAIVNDAMTSMSELEAPAVARAYDFSQARTIVDVGGGHGFLLATLLRANPHANGVLFELPHACE

GARQLFAKHGLTERVKVIAGDASHSIDAHGDVFVMKHVICDWDDEQATRIMTNCAEAMRPGGKLLLVEAV

LTPPGEPHFAKLHDLEMLIMSSGGHGRTADGYGRLYAAAGLMMTAVRPTQGMNSVIEGVKP

>WP_024429100.1 ABC transporter permease subunit [Burkholderia pseudomallei]

MTPVTSSIELPTAADAPPRSRTPLGLAARRFVRNRAALASLAALAVIALMCFVGPLLLPNDPAASDWSSI

SLAPTLANAHWFGTDELGRDLLVRTLIGGRVSLEVGLLGTFVSGLFGVAWGAIAGFAGGRVDAAMMRVVD

MMYAIPYLLIAILMMTLFGRSFILVVLTISAFSWLDMARVVRGQTLSLRTREFVDAARAIGVTPAAIVRR

HIVPNLLGVVVVYATVSVPAVVLTESVLSFLGLGVQEPMTSWGVLIQDGAQKLEAMPWLLLAPAVMLCVT

LYCVNFVGDGLRDALDPKDR

>WP_024429098.1 GNAT family N-acetyltransferase [Burkholderia pseudomallei]

MTVRNLDALFHPTSVAVVGASPRPGSVGAMVWACVLDGRFGGAIWPVNPKYGELNGHKVYPYVDQLPSAP

SVALICTPPATWPGIVRKLGGLGVRAAIIVGETRSGADRAALERALAAAKPYLLRVVGPGSLGVVSPALG

AHFGAPACIVKAGGVAWVSQSNALTNAVLGWAHARGLGFSHAVALGGEADVDAADVLDYLASDAETRAIL

LELDTVKSARKFMSAARAAARNKPVLALRAGRGDSGDLLYTAAFQRAGMVRVDALDDLLDEIEALGVGRA

AATSGGVTLVTSDRGVAKLAVDALAAAGETLAQWPRAAVDEVGGALPAGIVAGNPLLLGDDARPEYFGAA

LKALAQHPPTGTVFVVHATSHSAPAVDVARVLIESRKFARRGMLACFFGGVDAATRDALHVHGIPVHTTP

QRLARAHARLVDYQLGRELLMQTPEGTPPQPAASISAARHTARAALAQGRDGFEGDAALEWLAGFGIERA

TDADVDMGDTIVDITVGMYDDPNFGPVFRYSVPPADGVSAPFVVYGLPPFNTVLARAVVARSPYAHRAPP

EPLLQALTALSQAVCDVREIVEMSLVLRVRPTRVVALGPHIRLATGRSRLAIVPYPRYLEQQLDWRGERI

TVRPIRPEDEAAHRELLSAMTPDDLRMRFFGAIRNFDHSQIARMTQIDYDREMALIATLDDADGRAHTLG

AVRAVTDPDNEATEFAIAVRPDQKGKGLGRMLMTRIIDYARSRGTAWMIGEALRENTAMISLAKDSGFAV

SSTEEPGVVAFRLKLQP

>WP_024429076.1 response regulator [Burkholderia pseudomallei]

MHVEVFAQHPSCPGWHGEMARRIRAFDWSATELGPLERWPASLVAAVRTVLASPVPLVMLWGASGYMLYN

DAYAAFAGGRHPYLLGQPVELGWPEVADFNRNVMRTCLAGGALSYRDKALVLLRNGRPEEVWMDLYYSPL

AGDDGAPAGVLAVVVETTERVLAARHRDRAKAALHETNAELRRLTETLEQRVADAITERASIEEQLRQAQ

KMEAIGSLTGGIAHDFNNVLQVVSSNLQMLSAELGEDARAQRRIASAANAVARGAQLASHLLAFARRQPL

APAVLEPRGLIDGMREMLHRALGESVHVDADLRDDVWNVLADRHQFENALLNLAINARDAMRGEGTLKIT

ACNIVLEAGRHARRNGLPPGEYVAFDIADNGVGMTPDILERVFEPFFTTKPDGQGTGLGLSMVFGFVKQS

GGHTSIESAPGRGTTVRLLLPRCREAPAGEPAAQRRLPAEGGRETILVVEDDADVRLAVVDMLAQLGYKV

ITASDGEAGLLALESGAPIDLLFTDVIMPGRIKGGELARRAAQRVPPVPVLFTSGYTRDEIFHAGRLDPG

VMLLGKPYRRDELAHKVRSVLDAHASARRAHGARHAQADPRKKTAAARVQR

>WP_024429075.1 ATP-binding cassette domain-containing protein [Burkholderia pseudomallei]

MSAPSVVRFSDVSLRYGKTVALERITLEIPAGLTIGLIGPDGVGKSSLLALAAGARALQTGAVDALGGDM

RSRRHRERVCRRIAYMPQGLGKNLYPTLSVEENLQFFARLFGHDADERRRRIDALTRSTGLFAFLSRPAG

KLSGGMKQKLGLCCALIHDPDLLILDEPTTGVDPLARAQFWDLIARIRSERPAMSVIVATAYMDEAQRFD

WLIAMDAGRVLATGAPAELLARTGCDSLEAAFIALLPEDERRGHKPVTLEPLRADAQTGTAIEARGLTMR

FGDFTAVDHVSFRIRRGEIFGFLGSNGCGKSTTMKMLTGLLPATEGTAQLFGKTVDPKDINTRRRVGYMS

QAFSLYSELTVRQNLVLHARLFGVPAAEIDARVDEMARRFGLADIYDMLPDSLPLGMRQRLSLAVAMVHK

PELLILDEPTSGVDPVARDSFWQLMIDLARRDRVTIFISTHFMNEAQRCDRISLMHAGRVLASDAPAALV

RARGAATLEEAFIGYLVDASAQEAQDAQEAQGGAGERAAGESLAGRGAATDGDADGAHARPAAYANGAAQ

HRAPDADAAGTDVAAETGARDDARTAHAAGHSAGNDVRATVGSEVGSEVGSDVGSEVGSEVASEAGLAAS

AESGSERGVAHGAERAREPAADAGAAIDVEPGASAESATAAVAPARHAESAATSPRAGNAPAGTPFAAAP

AEPPHRAFSAQRALSYMWREMLELRRDPVRATLALIGSLVLMCVIGIGISLDVEDLTYAVLDRDQTELSH

DYALNLSGSRYFVERPPIADYAALDRRMRDGELSLAIEIPPNFARDVERGAPAQIGMWIDGAMPQRAETI

RGYAIGMHTMWLADKARHRLGVTLAPRAEVVTRYRYNPDVKSLPAMIPAVMPLLLLMLPAMLTALAVVRE

RELGSILNLYVTPVTRTEFLIGKQVPYVVLAMLNFLLMTMLARIAFDVPVKGSFMTLLLAVLIFNVVATG

IGLLASTFTRSQVAAIVMTIIGTMIPTVQFAGLLTPLSSLEGTGRLIGLVYPATYMLSISRGVFNKALSL

HDLYSQFWPLAACVPVILGATILLLKKQER

>WP_024429068.1 helix-turn-helix domain-containing protein [Burkholderia pseudomallei]

MAAQTPLRHRTTTTVDVVIYPGFKVIEAVGVINVFDYANARLAAAGLAPVYDLQIAAPAKGAVKSDTLIV

LEATKALDTLAVPDTAIVVGARDIERALRDTSMLVGWCRDVSARIGRMVGLCSGCFFLAEAGMLDGRRAT

THWSVAPLLRARYPAVKVEPDAIFVREGNVWTSAGVTAGLDLALAMVEEDLGREIALAVARDLVIYLKRP

GGQSQFSVYLASQMTAHASIRDIQDWILNALDARLSIAQLARRAAMSERNFIRVFVRETGYRPAEFIEIA

RLEKARRLLEQEALPLKTVAVRSGFRSDDQLRRVFMRRLGVTPGAYRERFSGTGVREARGSGDVD

>WP_024429057.1 LysR family transcriptional regulator [Burkholderia pseudomallei]
[truncated: 1,573,260 more chars]
