# Supplementary material for: Prediction of Burkholderia pseudomallei DsbA substrates identifies potential virulence factors and vaccine targets
Source: PLoS One. 2020 Nov 20;15(11):e0241306. doi: 10.1371/journal.pone.0241306 (PMC7678975; doi:10.1371/journal.pone.0241306)
Supplement: S3 Data — (DOCX) [file pone.0241306.s003.docx]

S3 Data

*B. pseudomallei* proteins from the core genome with a signal peptide (removed before counting cysteines) and an even number of cysteines (263 proteins), fasta format.

>ABA47916.1 putative membrane protein [Burkholderia pseudomallei 1710b]

MTSSRRSCSARTGRRGFTRSSCVTRDAAAAASRAVSIRISRGIHMKRHAAWAGMALGGALGAAPALASAATLDGAALSAFWAVPFAGILLSIAVFPLIAPVFWHHHFGKIAAAWAVAFLVPFAATFGFGTAFGTLMHALFEEYIPFIVLLSALYTVAGGICVRGNLHGTPRLNTGILALGTVLASVMGTTGAAMLLIRPLLRANDNRKHVVHVVVFFIFLVANAGGSLSPLGDPPLFLGFLNGVDFFWTTIHLALPMLFICAILLTLFFVLDTYFYRKGGEEGKPFLDPTPDSHGMSIEGKVNFVLLGAVIALVLMSGLWKPGIAFDLFGTHVALQNAVRDVALVAVALVSLAVTPRSAREGNAFNWAPIEEVAKLFAGIFVTIAPVIVILRAGGDGVFAPIVHLVTGADGKPVDAMYFWATGVLSSFLDNAPTYLVFFNLAGGDAQTLMTTGATTLAAISAGAVFMGANSYIGNAPNFMVKAIAESRGVRMPSFFAYLGWALAILVPVFLLTTWVFFSAQAAQ

>ABA48964.1 branched-chain amino acid ABC transporter, periplasmic amino acid-binding protein [Burkholderia pseudomallei 1710b]

MATAAARRAAGRVFCNRGAGGIGRKIAVEAAEGRVRCARARGAQQQEERAPRRFHVERETGAAACRFDRCAMSPCPLDRVSRSVWRHTMKMNRWIEVAFAAGCLCTAGLASAQVKIGVTVSATGAAASLGIPEKNTVALLPKEIGGKRVEYIVLDDASDTSRAVQNTRKLIDEDHVDAIVGSSITPNALAMIDVVAQGKTPAISLAASAHIIAPMDAKRAWVFKTPQNDRLMADALAGYMAKHGVKTVGFIGFADAYGDGWYGVFSAAAAANGLRIVANERYNRTDASVTGQVLKTLGARPDAVLIAGAGTPAALPAKTLKARGYTGKVYQTHGVANNDFLRVCGKDCDGELLPAGPVLVADQLPDSNPAKQPALAYKAAYEKAYGAAAVSTFGGHVWDAGLMLQRAIPDALKKAQPGTPAFREALRGALENVKDLPVSHGVINTTPADHNGFDTRARVIVQIVGDKWKLQAD

>ABA50111.1 ABC transporter, periplasmic substrate-binding protein [Burkholderia pseudomallei 1710b]

MACRFESGPRHQARPSARTPLRRAPAIRIRAASRRPRFRRAPPARLPSVREAALSRDSTARSFDAIHPPRSRPPRLLSASCPPRVAERRPAAALQPRPLAFAAVISSSSTPPIIPSTKTFTRLRFPLHDAHHQRRRRARRLFDRHRVARDQRAAHRPSGDARKDPRGDRRAALSPESARPAIAQRPHAIDRRRAADARESRVRRMPAGRRRTRDAGRLQADRDVDRIRCGARAPCDRDTARAARGRADAHRRRRRRAPAARRARPRRPALRADAQRHAASPVGGGRQSPRRVRRRADADRARPSARADARGLARRIRSRAAARARLCAGTRRARARTAARARARLQCTRAAARDARASERARDAPHRALLQQRLARDGRDSRAARRAPRGARRHVGARLRRPRGRRAARAAARERRDAESRDRPRRVAAPCRAHRRQAPCATRADAAARGARRRDRRAAARCARCARGAHRLNRAFAFRRFPFASPSRRPAVTVRRSLLPRSPLAAVRRACAAMLIPVASLALSLGASAAHAQDTAICYNCPPEWADWAAQIAAIKQRTGIRVPFDNKNSGQAIAQLIAEQKSPVADVVYLGVSSAFQAKDKGVVAPYKPAHWSDIPANLKDPQGYWFAIHSGTLGFFVNKDALDGKPVPRTWADLLKPEYKGMVGYLDPSSAFVGYAGAVAVNQALGGSFDDFRPALDWFRKLKANQPIVPKQTAYARVLSGEIPILLDYDFDAYRAKYKDHANVEFVIPKEGTISVPYVMSLVKGAPHEANGRKVLDFVLSDEGQKLWANAYLRPVRAQALGADVAAQFLPASEYARAKSVDFGKLAAGQQAFGKQYLQAMQ

>ABA50268.1 family S13 unassigned peptidase [Burkholderia pseudomallei 1710b]

MANRPHALRFLHDHVAEAADVADQVRAELLAQRVDIDLDGVAADLLAPAVHLLLELALRQHRARTREQRFEQRELARRQRDGRPVERHLMRRRIERQRAVPDHRFAAPRLAADQRAHPRGELVEIERLDEIVVGARVEALHAIGDRIARGEDQHRQREPARAQRAQHVDAVALGQPEIEQQQIVRLAAERGERRVAVLHPVDRAAVGLQRLAHAFGDHPIVFNQQYPHPEKRPSHAFEMADIVAPPRAGTDLRAISALLARRFLYNVRLAPRARPLLTSPSVLPHSSMNHHACRIAASPSPPPPAAVRARRTLARGAMLVAACAALAFAAPADARRKPKPPRYPAAVSAARNVLPASVLVALQRARVPASSLSVVVERIGDRTPAVAWNASRPMQPASTMKLVTTYAGLSLLGADYRWRTSAYADGDVDENGTLHGTLYIKGTGDPKLVPEELIDLVNKIRRAGIVNVDGALVLDKTFFAPQTRDLPPLDDDASAPYNVGPDPLLYAFKALSFTVTPTDSGAIAVDVVPALANLNVDNRLVEGQGSCDAARPTLVTDANGELTASFAGDYPPSCGPTTTNLAVLNHSTFFARGFLALWRQTGGSFSGTIAEGKVPGRARPVASHHGPVLSSVVHDINKFSNNVMARNLFLTIGAVEHRPPATPAQSADTIRAFLAHSGLPTDGLALENGSGLSRDERVSALGLADMLQAANASPVAQAFVDSLPIAGVDGTMRNRLTNAPVGGNAHIKTGTLRDVRAIAGYVASADGSSYVVVSFINDDRASAARAAHDALLEWVYEGPR

>ABA50277.1 aminopeptidase P [Burkholderia pseudomallei 1710b]

MSPIITGRCAFVDNRPSGLWMVCNNRVGRAGHATRTKQRRLPRGFPFRFAPCDRGARRRAARIGHANRACKAALGSSTRESGTRINAGMKRAVRLHISPRTAPSRIGRSLPPAVRAAACGCHREHAFARGPRARMQPRSRIAARAASHCRTAPRPHERRAIRRARPARRRYNRVMNQPTEPALALDVYRQRRDRVLASLRAQGGGVAIVPTAPEVPRNRDSDYPYRHDSYFYYLTGFAEPDALLVLDASAAGDAPRSILFCRAKNPEREIWEGFHFGPEAARDAFGFDAAFPYDALDAEMPRIVADAPALHYRFGVSAAFDARLNGWLDAVRARARAGVAAPGAAFDLGPLLDDMRLVKDAHEQATMRRAADISALAHRRAMAACRPGIREYELEAELLYTFRRHGAQSPAYGSIVATGANACVLHYPAGNAVVADGELVLIDAACELDGYASDITRTFPANGRFSGPQRALYDIVLAAQEAAIAATRAGTQFDAPHDAAVRVLAQGMLDTGLVPKTRFASVDDVIAERAYTRFYMHRTGHWLGMDVHDCGDYRERGAPRDDDGALPSRVLHPGMALTIEPGLYVRPGEDVPQAFWNIGIRIEDDAFVTPTGCELITRGVPVAADEIEALMRDARPAPRPQP

>ABA50993.1 dipeptide ABC transporter, periplasmic didpeptide-binding protein [Burkholderia pseudomallei 1710b]

MVFSPACSSYESSSNIRLRRPRPALNRRPGGNMEHNRLLRALRATAIAGVAAASFGIAGSAFAQIPNKTLVYCSEGSPAGFDSAQFTTGVDFTASTFPIYNRLVEFERGGTKVEPGLAEKWDISADGKVYTFHLRHGVKFHTTDFFKPTREFNADDVAFTFERMLDPNQAFRKAYPVSFPYFTDMGLDKLIVKIEKLDPYTVRFTLKEPNAPFIQNLAMEFASILSAEYADQLMKAGKAADINQKPIGTGPFIFRSYTKDATIRFDGNPDYWKKGAVKISKLIFSITPDPGVRVQKIKRNECQVMSYPRPADIATLKADSNVDMPSLPGFNLGYLAYNVQHKPVDKLEVRQALDMAINKKAILESVYQGAGQAASAPMPPTQWSYDKNLKAAAYDPAKAKALLAKAGYPNGFPITLWAMPVQRPYNPNAKLMAEMIQADWAKIGVQAKIVTYEWGEYIKRAHAGEHDTMLIGWNGDNGDPDNWLGTLLGCEAVKGNNFSEWCYKPFDELIQKGRVTTSQDGRTKIYMQAQQIFAQQLPFSPIANSTVYQPVRKNIVDMRIEPLGYARFDGVSVK

>ABA51121.1 Periplasmic binding protein family [Burkholderia pseudomallei 1710b]

MRATSRTARTRRRRRTAARNGCSARRARSSSSRRCASSPAAHPDARRRTHAHVRDPRHRTPELVARRRGRVAARAVAHGARGRARGPHRPERQRQDERAALRVPLRAAACRHGDARRAGRVACAAALERAAHRGAAAGGAGRFRADGRGTRAHGPHAAQAPVRRGHRGRCARRRSRAARRRSVGAARAGLRVAVGRREAARAARARARPAAGAAAARRTDQSSRRAPSARTACARARAARDDARDDPRSEPRRRVLRPAARARGRAHRRERRACRRADARAHRRRVRRRRDRRCAPGVGAAARHADLSGGCRTMLKRTAAERGAPALRAAFTLPFAAAAMAAAAIVATVATAAEANAYPVTVRSCGRDVTFERAPARAVSNDVNLTEMMIALGLQTRMAGYTGIAGWKTGNARLRAALAGVPELATHYPSLEALVDARADFYFAGWNYGMHPGGPVTPASLERFGIRTYELTESCSLVMRRPPASLDDVYRDLANLGRIFGVDARAAQVAGAMRARIDAVRRTLASAAGGGTPPRVFVYDSGTDKPMTAGALAMPTALIAAAGGRNVMDDVPRGWTQVGWESVVARDPQAIVIVDYSAVTAEQKKQFLLSQPALAQVEAIRARRFIVIPYDAATPGVENAAAVETIARGLHPRAFAKAAR

>ABA51731.1 Substrate binding domain of ABC-type glycine betaine transport system protein [Burkholderia pseudomallei 1710b]

MGLAAVPGCEPALHAQSPRTRRARGSRAFSSSARMTPGVTHFQMEQAMKRYESIARRLARRAAAASPAFAALAWCAAAAAATTTAAAAEPAACRDVRMAGPGWTDIEATNALAGVVLKALGYRQSVSNLSVPITYQGLKKGQLDVFLGNWMPAQAPLVKPFVDARAIDVLHANLSHAKFTLAVPDYVAAAGVHSFADLAKYAQRFGAKIYGIEPGAPANQNISRMLADKALGPANWQLVESSETGMLTQVERAVRERQWIVFLGWEPHLMNTKFHLVYLSGGDAYFGPDYGGATVNTVARADFASQCANLARLFRQMTFTVDLENGMIAAMLQGKRSAVDAAQHALRANPSLVEAWLDGVRTASGAPGLPAVRAALDAQ

>ABA51907.1 trehalase [Burkholderia pseudomallei 1710b]

MLARATTCCRHALPFARRVRSGRGARCRGGVVGAARVCARRARAARVPDASIRARCAAPISGEPLARTMTAVFHRRGSALRAGVHAMPKKRRAARSAGLRGIGTKRPRRRPTRPAAGHRSRSAVPARRAGAAAPASPGAACLVRAGACATRRMPRPTVICAKCGKNRLPERRFSCESAAAAYDRQTRRHASGPRDQETVLPFISKTGGGDMVTPRHRPLHVENPFYRRLLNAPAWVALVAAAGIGCTSATLARADSSPAHASTAAAVASAASAPGAASIPPPPSQLYGDLFVAVQTAQIFADQKTFVDSTPNADPATIVQLYQQQKGQPGFSLKAFVAQYFTPPSDESVTPPPNQTLREHIDWLWPKLTRTTTTAPPYSSLIALPKPYVVPGGRFREGYYWDTYFTMLGLQEAGREDLVDNMLDNFAYLIDTVGHVPNGNRSYYVSRSQPPFFAYMVTLAAKAEGNRVYQKYLPALRKEYAYWMQGERTTPRGQATRNVVAMPDGSVLNRYWDASDTPRDESYLEDVKTAQQASGRPAAEVWRDLRAAAESGWDFSSRWFGDNRTLATIRTTAIVPVDLNSLMFNLETTIVKGCAVTRDFACVAEFAGRAGKRAVAINRYLWNRNGYYGDYDWKLGKPRDNLSAAALYPLFAGVAWPERAKQTAKNVQKALLKPGGLATTTYDTAQQWDAPNGWAPLHWIALVGLRHYGEKSLADDIGTRFLADVKGVYAAQGKLVEKYIVEGVGTGGGGGGEYPLQDGFGWTNGVTLKLLDLYGG

>ABA52048.1 spermidine/putrescine ABC transporter, periplasmic spermidine/putrescine-binding protein [Burkholderia pseudomallei 1710b]

MLRQGAARHRRARCVRARRADLARVPARASAGLRMNAKPRVARRASYNHHASSSVTRGNIMHHTFNTPRRAVALLSLAAFCATAGAAELTVVNFGGANGDAQKAAFNQPFEKATGNKVTAVEYNGEQAKVKAMVEAKHVNWDVVEVESGDLNRGCDEGLYEKLDWAKIAKKSDLIAESPQTCGVGFFVWSTALAYDADKLKRAPAGWADFWDVKKFPGKRGMRKGARYNLEFALMADGVAPKDVYKVLATKAGQDRAFKKLDELKPNIQWWEAGAQPPQFLVAGDVVMSTAYNGRISAAQKEGKNLKVVWSGSIYDLDYWAIPKGTPNKALAEKYIAYTLSSKPQQDYAQHIAYGPANVAAIKALDAKTLANLPNSPANGKNAVLQNITFWTDHGDELEQRFAAWASK

>ABA52198.1 beta-glucosidase [Burkholderia pseudomallei 1710b]

MRRPANVRYQTARAAQPTATSRISSASASASASASAATATATATATAAAAAAIPRCRPASILPLDSRSIRSPKPDPATARRARARPAGGRSVAYARPNPRQSKTSSIDHPIVQKFTCSQSTYALSLNLKFCYIPTQPPLTPDSSPAARGRSVTHDRVSSVTRLTAPVAARSRGVSRRAERQNQANIHGDKRMHAKRLSIAVLSATLCALAHAAGNDAPSPDIASRDAYALRRAHALVRQMTLDEKLQLIHSKYPMSDVPGGGAGFIQGIARLGIPDLNMVDSATGSGSTSQPSTTFPATIGLAASWDKRLSYAFGAVIADQLRAQGFAMGLGGGTNLAREPRGGRLFEYLGEDPVLAGEMLAARTRGTQDRKVIATIKHYVGNEQETNRMGGDDQIDERTLRELYLLPFEIAMKAARPGNVMCSYNRLNGDYACENAHVLTDVLKNEWHFQGQVQSDWGAAHSTAKAINAGLDEEEDVGPTVFLTPALVKQALANREIAPARLDDMVRRKLYAMIRTGVMDDPPRGGGTIDFAAANRFVQYAAEQSIVLLKNQDRQLPLDAAGLKRIAVIGGHADAAVLAGGGSGNTRHPVTGAFPGCGGLTFPTTTGCNWWPNPWLKLDVPIVQAIRDLAPGATVAFAGNSDRQSPFAAYTPQQIDAAADLARRSDVAIVFVTQAAGEDFGELRSLALANPTNQDALVQAVAQANPRVIVVVESGNPVLMPWRDQVPAIVQAWFPGEGGGNAIANVLFGKVNPSGKLPVTFPARDEDTPTWGADGTLAPNPVYSEKLKIGYRWYDAHRIAPMFPFGHGLSYTHFSYSGLEVKQRPDAATTVSFALTNDGPVAGAEVPQVYLGDLDDPQEPPKRLVGWDKVGLRAGETRRVRIVIPAEMRRVWDASRNGWALAKGGRIYVGASSRDIRLQQP

>ABA53699.1 putative inner membrane glycosyltransferase [Burkholderia pseudomallei 1710b]

MAANTGNVRIHAHAFAAHASRCRVRDGFSRVDSPMNQNAAHEPAAVARLRRARLVARPFPSDGSPFALNQRRSVPTRYDVSTPKERPTGRNRKPAARRAFGRTARQAARRDGRYARHAAPVSFCKSVRGERSRRRCGHGDTSMKSGRAMSLCVQCVLLLIVFACSLSARGTALGPASASLATAPFARTAQARAFAQPSLPHAEAVAFAFAQAEPFDAERPAAAGRPAAADAPPAPGKGIVISLCAAYLWVFALLTLVYASRHYVFGLDRLFKPQRAPYRAITHADWPEITVFVAAHNEEAVVADCLTALLATTYPRERLTIVPVNDRSTDNTRALIDEVQARAPELIKPFHRESGKPGKAAALKDALREIRGDIMVVFDADYLPRPGLLKELVAPFFDPEVGAVMGRVVPQNADRNLLARLLDLERAGGYQVNQQARNNLGLVPQYGGTVGGVRKSALDAVGGWRDDTLAEDTDMTYRLLLSNWRTVYLNHAECYEEVPERWPVRARQLTRWAKGHNQTLFRYLIPLLRSPVTPRRCRLDGALLLGVFVMPALLALAWGIALALYLTNGIDSLVLGLLVSVFALFAFSTFGNFGVFFEIVVAARLDGRATRLRLVPVNVVGFCVTIAAVVAALWGLALDALLRRELRWDKTERFRRPLNSGR

>ABN85841.1 conserved hypothetical protein [Burkholderia pseudomallei 668]

MCAFVAAPLLWGLLAGVAAAQALAQPAASTVSVCAAVITGSSGTTVDVSYAGLPGNQPKLYGDFVALWQSSVVPWSIAPAARLGIPTDAELGTVVMTGVSISATPYTVGYAVGPEVDDVCASALLAADGSTGVVDTVTLQLASVGATSLTFRYHTLSGYLPATAGNWVGLWRGRASPYNAAASVARVKVAADVTDDSVVMEGVTLSPGEIYTAIYFMGEPLTTAAVLLNFVAPAR

>ABN86005.1 glycine betaine/L-proline ABC transporter, periplasmic glycine betaine/L-proline-binding protein [Burkholderia pseudomallei 668]

MLTTSLLMRRSTMKRNLIAAACGLAIAAAPFASARAGDAPTCKAVRFADVGWTDIAATTGLASTMLAGLGYAPTKTIASVPITFAGIKSKQIDVFLGYWSPTMDPMIAPFTKAGTIKVLAAPNLTGAKYTLAVPDYVYQGGLKSFADIQKYADKLNGRIYGIEPGNDGNALIKKMIDGNQFGLGKFKLVESSEAGMLVEVNRAIRDKQWIVFLGWEPHPMNVQMKIDYLSGGDDVFGPNYGEAKVLTATPPDYAARCPNVAKFVSNLQFTTSIENHVMLPIMNKEDPNKAAAEWLKANPQSLDKWLAGVTTFDGKPGLPAVKHYLGIQ

>ABN91854.1 type I pilus protein [Burkholderia pseudomallei 1106a]

MLFLLGVAAWPAAGRADTLLPRTQAFTVSAQIVAGCGVAGGGPASGLNFGTLDFGAHPAVATGQVSAAVGGGALQIECSPGSTLKMTIDGGANASAGNTQRNLASGGARVAYRLYSDPARTQAIAVGQAVSLPVSGTITLPIYGALTLPGGGAPAGTYTDTAQVTLSY

>ABN92885.1 ABC transporter, quaternary amine uptake transporter (QAT) family, periplasmic substrate-binding protein [Burkholderia pseudomallei 1106a]

MQAARLSRNRLRNPSNREATMKSTTTFVFGAALAAACALPSQSFAQDSAACRNVRFADIGWTDITSTTALASLLFDGLGYKPTTTIASVPISFAGLKNRQLDVSLGYWWPVQQHQLQPFLDSKSISVVEPPNLSGAKATLAVPSYVYQAGLKSFDDIAKHRAELDGKIYGIEPGSSANATIQKMIDTNQYGLGGFKLVESSEAGMLVTVERAIRDKKWVVFLGWEPHPMNIQIGMNYLSGGDAAFGPNYGEARVYTLTSPDYMARCPNAGKLVGNLRFTTQMENQLMQAVMNKVKPAEAAKAYIRKNPQVLDAWLAGVKTYDGKDGLAAVKAYLGL

>ABN94432.1 putative peptidoglycan-binding LysM/M23B peptidase [Burkholderia pseudomallei 1106a]

MTGVVSSMSKSEIDRSVAWLSGVAAAFVVAGCATTSPVTPTDTLAAAASSASAAQPAAAPPGDAAQHPEHAAAPAPIATRYVVRRGDTLSAIAQANGCTVRELQAWNRMGRRTRIGIGQVLRIAPPGAENAVASEAADGAGATRAAGNAAGAARAADPASGAGGAPVADAPQPASAPESAADRVADHRVVQETKRHAQSIALAWPAKGAVVETFQPGRNRGIRIVGRAGDPVRAAASGRVMYAGTGLNGYGTLILVQHNADFLTAYAHNRKVLVKTGDVVQQGEQIAEMGTGDSTRAGMLFEVRRDGKPVNPMPYLASRQQG

>ABN94719.1 putrescine ABC transporter, periplasmic putrescine-binding protein [Burkholderia pseudomallei 1106a]

MRRRSNWVFPNGIANRAGGARAPRAHLHHQGNNMSVSHLRHAVARAALVALAGASALALPAAHAAGAELNVYNWSDYIAKDTIPNFEKQSGIHVKYDNYDSDDTLQAKLLAGSSGYDIVVPTSNYMAKQIQAGVYQKLDKSKLPNLSNLDPTLMKMIADADPGNQYGVPWAFGTDGLGYNVQAVKKALGENAPVDSWALVLDPANVSKLKGCGVSFLDQAVDVFAATLQYMGRNPNSTNPADYQAAFEVLKKVRPYITQFNSSGYINDLANNDVCVALGWSGDVGIAHRRSAEAKRPYDIKFSNPKEGGLLWFDVMVIPKDAPHAEAALKWINYIEDPKVNAAITNEVFYPSANRAARQFVVPAVAQDPTVYPPEDVLKKMTLMRPMPADILRLENRLWAQLKTGH

>EBA48499.1 putative amino acid ABC transporter, periplasmic amino acid-binding protein [Burkholderia pseudomallei 305]

MSAPVRAHTPTGKGNSMKRRSLLKFGSMAGVMALAGQSPIARAADSGKGPIKVGILHSLSGTMAISETSLKDTALMTIADINKNGGVLGRPLQPVVVDPASNWPLFAEKARQLLTQEKVACVFGCWTSVSRKSVLPVFEELNGLLYYPVQYEGEEMSRNVFYTGAAPNQQAIPAVEYMMSAEGGGAKRFFLLGTDYVYPRTTNKILRAFLKSKGVKDSDIQEVYTPFGHSDYQTIVANIKTFAQGGKTTVISTINGDSNVPFYKELGNQGLKATDVPVVAFSVGEEELRGIDTKPLVGHLAAWNYFMSVKGPANAKFKEQFAAWVKSQNLPGGAKRVTNDPMEATFVGIHMWKQAVEKAKSTDVDRVRTAMIGQSVAAPSGFTLTMDGNHHLHKPVMIGEIRGDGQFNVVWKTKTAIRAQPWSPFIAGNQGKPDVVGSIPEFLRRRRAALA

>EBA51467.1 putrescine ABC transporter, periplasmic putrescine-binding protein [Burkholderia pseudomallei 305]

MHGRAAGAAFGRAGARPPTRNDERAPPEFRVGDPFGPRGLFSGWTRADNPIKLRPPAASRRPAPCNRKVRMCSVCATTAGNFCGGDGLVVAFGVFKGRRETTRRSGEADGDRIGSIRENGMKAKVAGRLTALALCAGATVAAAKDTQLNVYNWSDYIAKDTIPNFEKQTGVKVRYDNYDSDDTLQAKLLTGNSGYDIVVPTSNYAGKQIQAGIFTPLDKSKLPNLKYLDAQLMALVAGADPGNKYVVPWAYGTTGLGYNVDKAQKVLGKVPLDNWDILFKPENLSKLKTCGVSVLDAPDQMFAATLHYIGKDPMSTNPADYQAAMQVLKKIRPYITQFNSSGYINDMVGGDICFAFGWSGDVVIAKHRALEAKKPYKLEYYVPKGGAPVWFDVMAIPKDAKNKDAALQWINYIEDPKVHASITNAVYYPSANAQARKYVRPDVANDPAVYPPPDVVKTLFLLKPLPPEIQRLQTRLWTELKSGR

>EDO83218.1 NAD-dependent glycerol-3-phosphate dehydrogenase family protein [Burkholderia pseudomallei 406e]

MLSMKVAVLGAGAWGTALAAHLAVRHDTLLWARDAALVAELAVRRENARYLGGVALPPGLRYEADLATALSHAQADDALCVIAAPVAGLRALCRAMRDARRVPAHFVWVCKGFEADTRRLPHQMVAEELPDHASYGVLSGPSFAREVAQGLPVALTVASASAACRERTLAAFHHGAMRIYTGDDVVGVEVGGAVKNVLAIATGIADGLGLGLNARAALVTRGLAEMSRLGVALGGRAETFTGLTGLGDLILTATGDLSRNRSVGLQLAAGRSLDDILAALGHVAEGVRCARAVLSIARERGVDMPITEAVCAVLFDGVAPRDAVSGLLRRDAKAE

>EDO83840.1 chitin-binding protein [Burkholderia pseudomallei 406e]

MTESLKESANASHFDIQNNRLISPHRGTRTVSFLAWTHLEERYMKSLFDAPSSRPRARAALTLGAAATLTASFAALLAPMNADAHGAVGFPIARQYQCRLEGGYWDPPNGSAIPHDDCRAAYRAGNNSAYPFTQWNEVSANPVGQGNDLVQLKAAVPDGLLCAGGDTSKAGLDKAPASVWRKTQLTPNNGHIELQWENTTAHNPARMRVFISKPSYDPSRPLRWDDLQQIYDAPAPAPAPVPANGAGHLPGSIQSFYKLDVTLPAGRTGDAVLYSYWQRIDAGNEGFFNCSDVTIAADERASGFPWVAARAFVEPGIAPRAGQQVRFRVMINDARGAEVVDVRQPITPYNAERSVWAKQIADQVNGRYGNIAKIGVRSGNTIYFDATNLDANKVWLQPNYSSALSVVGAK

>EDO85817.1 putative lipoprotein [Burkholderia pseudomallei 406e]

MRMPKQSAAIVVIAAFSCGAMESSRYIISDVSRNQSSLPSRHVPDSTRTAGESGRSARAPDAPIQSTSRNVRNRNASMRRATRALLRSLDIRHFFVVGRARLPLSLRTTTIIACCESLVATHKKSSRLASFRHRGAADAIGPAACAVANALSCRDTCLRSHAQAAAVPANRAARDGLSSRQSVAAAPNPRIVPAGAALSGRMRRRIACMREHAGAMPRPPAAAHASVPFVPSARFARFAPFAPSALPIRCFT

>EDO89205.1 class D beta-lactamase [Burkholderia pseudomallei Pasteur 52237]

MPAAGAGRTRADRLLADPFCFSGLARGAATIVLVARVLVRKLSVCHNASPITRARAMPFSDRPAFQAHAFDPDRANAVCRAVAFVGRKRSRAASWLDDPSRFAMFKSQQFIAAIWLSPGCCIVFAARVAQFAAAAETGRMGQQDGLDERLLGVCGVRAVEEDRRRDSGEQNRIRRANEWRPAIESSLRSFGPNDEPFPTLCGTTMKFRHALSSAFVLLGCIAASAHAKTICTAIADAGTGKLLLQDGDCGRRASPASTFKIAISLMGYDAGFLRNEHDPVLPYRDSYIAWGGEAWKQPTDPTRWLKYSVVWYSQQVAHHLGAQRFAQYAKAFGYGNADVSGDPGQNNGLDRAWIGSSLQISPLEQLEFLGKMLDRKLPVSPTAVDMTERIVESTTLADGTVVHGKTGVSYPLLADGTRDWARGSGWFVGWIVRGKQTLVFARLTQDERKQPVSAGIRTREAFLRDLPRLLAAR

>EDO95399.1 putative ABC transport system, exported substrate-binding protein [Burkholderia pseudomallei Pasteur 52237]

MGGISSNHGSFGIARYRRRCAACVRRVYERLRHQLFLGWCRHDHLAYVHLQRIEDRNYAKGKCYLDSRHWTFVDSTTDGLGSRQEGCRTDCHAGEIRAVSREHSNDRNRMKKVVGAMLLLAAGHAVSAELHIGSWPDYLPDTLIKKFQAETGVKTTLDTYASDAALTQKLQSGGGGYDVVIAGDYYVPVLVKSGLLQKLDKNKLPNIANIKPEYRHPSFDPQRDYAMPYTVVLTGFAYDSARVSGGKLDESWKSFFDPPAQLRGQIGDLDVEEELYMAASWYLGQDECTENPADAKRVLDVLQKQKPFVKTYSNDGTIDRLASKQIAVQHIWSGAAARAQDRLPSITFVYPREGVRLFMDSLLIPAKAQNTASAYQFVNWMMRPENIAQVTNAVRYNNEIIGSERYIDAALLKNSAIKTPEQYKIRLRPYKICSPAAIQLRNKVWLKLKGNR

>EDS82086.1 putative phosphatidylethanolamine-binding protein [Burkholderia pseudomallei S13]

MGRRAVPRGSSRRLLSLALVCACALAPVGGSRALAEDAFMLTSDDLHPGGRVGAPHVFNQGDCKGGNRSPQLAWRNPPPGTKGFAITMFDPDAPGRGWWHWAVVGIPASVSSVPADASASGFLRKLGAAEARNDFGTDGYGGPCPPPGKPHRYVITVHALKATDLRVATGRPAQMFDHEIGTESIGSAQLVVTYGR

>EDU07436.1 conserved hypothetical protein [Burkholderia pseudomallei 1655]

MPQREAMSALARLLPRRAPGWLAWACAALVVALAQPAWAVRCLTNSGQTSLTEPIGNVASYPTDAPDGYVIWISAPRTTDGYCYKDLGGAGNLNIVDNIYFYANPDGKNPAAWGLEIGIRYRGVDHFGAGSRPGTGVPTGFAVPPCSQTDFNAKRCPKIPVSITYQVVVRKKGAWVQPPGDVYTVFQFDGEKGLNNTNPSFQYKLSGLQNLKPTPCMVDVTVTPEPGIVKFGQVQSSGNGFSPAVPRKPFSLALTKKCSIAVRVDGYFETSQAVRDGLLVPQSDSNFGIGIEDRNGHAIAFNRQFVLTQMPANVSYQNIVLDAVLKSFGAPKIGPFTGTATIRLFIY

>EEC34719.1 carboxymethylenebutenolidase [Burkholderia pseudomallei 576]

MGIVCGFRLGGAGGGARRDCIAPAAVIRIHVGDACAARARQREERSASMLKPEVDSLVPHVPFSRRKFVQAALGGTFAAAVLPVSAQTITTDAAGLDVDTVQIRSGDASVPAYRAQPDGKSNLPVIVVIHEVFGVHAHIADICRRFAKLGYLAIAPDLYARQGDPSKHASIQELIDQVVSKVPDRQVIEDLDATVRWAGKNGGDLSRLGVTGFCWGGRQTWLFAEHNPHVRAAVAWYGKVAGETNEMTPFNPDDHAAQLKAPTLGLYGGKDDSISQRSLARMRERLAAAGTQAARESEIVVYPDAGHAFFADYRPSYVKADADDGWKRAIAWFRHHGVM

>EEH25189.1 bacterial extracellular solute-binding protein, family 3 [Burkholderia pseudomallei Pakistan 9]

MHCIVEPARLRLAAAIRTAAAASIGRRPPRRRAHADRATTPIARCTARFVTRPPFRQETTMMKPLRPLLSIAVGCALAAAAHADDAGPTLKKIRDTGVVTLGVRESSVPFSYYDQQQRTIGYSQEIALKIVDEIKKTLNRPNLTVREIPITSQNRIPLVQNGTVDLECGSTTHTKERANQVSFSNSIFQYGMRLIVKKSSGVKDFPDLAGKTVATTAGTTEERLLRQWNAEKGMAMQIISAKDHADAFLNVKSGRAVAFFMDEPLLYGAKAKEANPGDYVITGNSPVSEAYGCMLRKDDPGFKQLADRVIARMQRSGEAEALYVKWFNRPIPPKGVNLDYPLSADMKQLFANPNDKALD

>EEH25224.1 putative glucan 1,4-alpha-glucosidase [Burkholderia pseudomallei Pakistan 9]

MLFCYKNHNRRIHNGDEKMSARHFGFRQAVLVGSLAALAGAAALPANTSAAEAFGSPGAAPVRGPAAKSFLGTAVNGASRVYFTGYRGILSEVYYPVLDTPESVDLQFLVGDAGKTFVDEEKQQAYSAAQTDTRTMSWQVTTGNSSHNWQIRKIVFADPNHNAIVQRVTFTALDGRKVGDFNLYMLSKPYLDNAGANNTAQTVSGSGGTALVANHNSRYSALVASRPWKVVNGVGMTSNGFVGQSDGWTDLFGGTADKTMDWTFSSATNGNVAQMGWLDLGDPSATSVSFDVVLGFGGTQDQALGDANTVLGSDLAGEQQQYDTGWHNYAAGLSSQNGAADDGYYLAAMTLKTMQDKSNGAMIAGIGTPWGETQGDANAGGYHLVWPRDLFKFANALTTAGDTSSATSVVNYLFNTLQQTTDCGTAEYNAPGCAQGYSRVGRFPQNAWVNGWPYWQGTQMDEQAMPIILAWRLGPSVFNPLWPKIKLTADYIVNTGPWTYQERWEENAGYSPSTIAAEIAGLVTAADIATQNGDTASAARYLAAADYWQENVAAWTYTSSGAFGNGSYYIRINPAGRAGTGTDRASFAPTAGPDTPQTLSVKNGGGSHDARRVVDGGFFELVRMGVKRATDPTIANTISVYDSVLGQTLNVANAPALSPNAWFRYNFDGYGEHNDGGDFDGTGAGRLWPIFTAERGMVEIARQGSGGAGSAYFSTLKQLTTPEGFVPEQVWSNSTTLPDGWAVTTPAGYTPGQPTKSMAPLNWAMGEYISLLASIQAGRIVDVPSVVCARYNNCAAPPQSGQVPVAVNVNASTQLGQQMYVTGNVAALGNWNTDLGIPVDPASYPVWRNTVNLPAAQAIQYKYYRKNADGSVTWENRSGNRQLQTPASGTLALNDQVSW

>EEH28759.1 putative secretory lipase [Burkholderia pseudomallei Pakistan 9]

MKNPIRNTDVQPQHRRARRTLRVLRRAAHRLAMWDDTVEIASASAFRDRSQSARLPRRTLEEASLMSRTGFMPLSTLATMAVAAAISTPAAFAESAPVSGNGGIPSFYRWTDKVPQTRGQMLRTEVLTPRQGLAAAGQNLRILYTSTDGIDGHTPIVVSGALFVPKGTPPQGGWPLMAWAHGTVGSADICAPSYAGHSERDVRYLSHWLAQGYAIVATDYQGLGASGPHPYGLTRPLAYSVLDSIRAVQNGGFELSPKRIVVFGQSQGGRAAFATAVYAKSYAPELDIGGVVATGTPYSTIRHRADDAEHSKLHRSVIHNILRLNATPKLLDPSFATSDYLTERATPAFEFSQHACLHAIKQKIASDGLTFENSFKRSPQPVLDWIRREAAYPTLKSAIPIFIGTGGKDRNVPVSDQAALVKDACEAGDRIEWHVYPALDHSETVNGSLSDSTPFVARAFAGEHIDGNCASLPSP

>ACQ98979.1 serine protease, subtilase family [Burkholderia pseudomallei MSHR346]

MTSRKWARSRASQAKHAIYAATFFAAAALSAHAAAAWVDTQTGAYPALAQQALAASQASAAATAAGKAIDTAPGEPVRVVVSLNLNDEARLDRFLRDLHTPGSAAYGRHLTPAEFAAQYAPTPQQVALVEAHLRRAGFRDIEVAPNRLLISATGTAAAVKTAFNTRLKRFTLEGRRVYANQDAAQVPAELGRIVGAVLGLDNATLARTYNRQAAVTGAVGGAKASLAARASDASAAASGAPVLTGHDPLEFSRIYRAGSTPTASQTTVGVIMAGDAAPVLRDLDTFAAKAGLARVAATVTRTGPPGSDYSDNSGLSEWDMDSQAIVGAAGGAVKGLVLYAAPSMLLSDITSAYNRAVVDNVAKVINVSLGVCEADARASGTQAADDRIFKSAVAQGQTFVVAAGDAGAYECSVSRVSGGQGVPARSNYSVSEPATSPYVVAVGGTTLSTDRTTLAYAGEVAWNEGLQPIGVYDAYGSYDGTRRLWATGGGYSRSEAAPAWQRSVLGASAKARALPDVAFDADGRSGAHVYVNGRTEQWGGTSLAAPIFTGIWARVQSDNGNRLGFPLASLYRYAPANGAFAHDVKSGNNGSGGYGYKAGAGWDPVTGFGSLDIANFAAFVKQTADFAR

>EES27008.1 bacterial extracellular solute-binding protein [Burkholderia pseudomallei 1106b]

MERSPMQRASGRPFSSASSKERSMKRIAWLAAVLASLACAAARAADGNVLNIYNWAEYFAPDTIAGFEKETGIKVRLDVYDSNEALQTKLTTGNSGYDLVFPSNDFLARQIQAGLYRKLDKSRLPNLTNLDPAIVARAAEVDPGNQYSVPYMQGTFGLGLNVAKVKQALGGPLPANTLELIFNPAYAAKLERCGIAFNDAGSEVFPLALRYIGRDPNTTDPRDYEAALDMMKKIRPTIRQFIATPVMNDLATGDVCVVTGYSGAVLVAARRAAEAKNGQQIVYSLPSAGAPFWFDSMAIPKGAAHADHALRFIDYILRPDVVAKISNKVMYPNPNRVATPLVDRRLTANPAIYPDAATMRTLWVKRPMPPQAIRMQTRYWTRFKTGY

>AFI68205.1 Gfo/Idh/MocA family oxidoreductase [Burkholderia pseudomallei 1026b]

MRRPSRSAPFRKQTGSFMSSTLKIGLMGFGFAGATFHAPVITHSGRTRLAAIATGQPERATAAYPEAAVVPDLDALAARDDVECVVIATPNDTHVDLAKRALEAGKHVVVDKPVALSAADALVLARLAAARGRVFAPFHNRRWDGDFLTVRQLVESGELGRIVCFESHFDRFRPHVRTRWREEAARGGGLLFDLGPHLIDQALALFGPPETVSATVKTRRDGGDAPDFVHLQLGYPDKDVVLHASALAAIEPARFTVLGTQGGYQKHGLDTQEDQLKAGLTPDDVEFGGGNPPGVLRGLDGELEVERPVPTLDGQYAEFYRALAASIHDGAPFPVAPQDAVDVMTIIETAMRSEREGRRLPFVREAV

>AFR15131.1 hypothetical protein BPC006_I1246 [Burkholderia pseudomallei BPC006]

MRAARRHCLTRKKNMNRKTRAAIGVAALCAAAGAAHAARLTVEEIDADARETVAYRCANAPKPVRVSYWRAGNGQSFALVPVNGVRLLFVDTVSASGARYQAGRYVWWTKGRDANLYDEIAGERAPPVLADCSEVRKPRGKG

>AFR15554.1 type I pilus protein [Burkholderia pseudomallei BPC006]

MSACAGVQAQTSPLTGTVNSQLVLTTGCAVDTGGGSVNSANFGTLDFGTQPSGFTGRLTSAAKGGGSTSTQVTCSPDVTSIQVTIDGGQNASKGATVGTGTRALANGASFVPYEVYADAGHSQQYVSGTAQSVAVPTPGAAFELPLYGVVNKTNASALAAGTYTDVLNVTLGW

>AFR16106.1 hypothetical protein BPC006_I2236 [Burkholderia pseudomallei BPC006]

MRHASITRRFAQIVAIKGEVRMSQRILSPLVASCAAAALLSAFAAHAAPPIKGSVLGGNDGQLQYTVKVDSKQFGTMQETRKIRSGETDDYNWKSVPPSGAVPMPDACPNADTLPRDANGAMVRQAQVRLAPAVDSKGVANVQLSFQASAPSGTKKVTVNGKALQCPNVVAVSQVKWLSIPTGGSKSITMRDGTKITVSIKR

>AFR16694.1 glycosyl transferase, group 2 family protein [Burkholderia pseudomallei BPC006]

MRVVESASKSERSWDRVRNRPRRSIASPSRLPPDRREARGMTESLSIVGWVLLALVCASCGYAVLAACAPAPRVPRAAARDGFEPVSVLKPLCGSEPHLYENLATFCEQRHPRYQLLFGVASAADPAIAVVRRLQADYPDCDIELVIDARVYGSNLKVSNLVNLAERARHGRIVIADSDIAVEPDYLTRVTAPLADPSVGVVTCLYHARSVGGFWTRIGAQFVDAWFAPSVRITHLGGSSRFGFGATLALTRATLDAIGGFKALKDELADDYWLAELPRRLGLRTVLSEVNVATDVAEPSFAPLWLRETRWLRTIRSLNPAGFAFLFITFTAPWLVIGAALAAWLGPASAAGATAAWAAAIGTLARLALHARGAAGWRAFWRDLPLVPVRDALLALEWLAAAFGTQVVWRGARMTVVGGDARATVVEAGDGR

>AFR17326.1 glutamate/aspartate ABC transporter, periplasmic glutamate/aspartate-binding protein [Burkholderia pseudomallei BPC006]

MEAASRRTRGRHRNGVARVRIRAPNAGLSAHVEMRMPPGKSVVNNHYFHKNYFAKLARVFAKEMTTMKFPKAMLMVAALSTFAGGAIAQETGTLKKIKDTGVIALGHRESSIPFSYYDQNQQVVGYSRDFQMKVVDAVKKKLNLPNLQVKNIPVTSQNRIPLVQNGTVDIECGSTTNNLDRQKQAAFSDTIFVIGTRLMTKKDSGIKDFADLKGKTVVTTAGTTSERLLREMNNKNQMGMSIISAKDHGESFQTLETGRAVAFMMDDALLAGERAKAKQPGEWVIVGKPQSQEAYGCMMRKDDPAFKKVVDDAIVQVEKSGEAAKIYSKWFENPIPPKGLNLNFPLSDEMKKLYANPNDKALD

>AFR18870.1 collagenase [Burkholderia pseudomallei BPC006]

MTIHDHQSNSRQQRANMKNSHNVVNRFIVAASIIIGVVLYSSAWANPQPMHTKQARMPRIPQNLPLSPDQAKYDLPLSKYDRATLMEPLRRKQSAKPDRRTRPGADCRDMSIMTQYHGTALADYIANLPDYECHYGLFSIDRAMAAQIFNSENVWAVASRLTQEINRYDATNITLVNLLIYLRAAYFQYDAAQLADPIPGLVVWLRPYILQSLSGDALYLENSRAPSTANELMILITNMKDEAYYLPTLKDRIAFYTASATNPQAAAPLLQRSAAGGFTGLLTVFFYAHQRSGAQPMLDSDATLPETLNRFVTANRAYLSNTSAAYQLADAARETYRFLRYPSQKPRVKKMIQDMLASTTMTGPDNDLWLAAAEAADYGDPGNCADYGTCDYQKRLIEAVLTHRYSCNANVRILAQDMTVPQFQSACQSVAQEEDYFHRMMKTGHVPVANDHNDTIEIVVFGDYDNYRKYASVIYGISTDNGGMYVEGDPSAPGNQARFIAHEASWLRPEFKVWNLEHEFTHYLDGRYDMAGDFAASTAKPTVWWIEGLAEYISRKNDDQESIDAVRTNAYRLSDVLQTTYSSGDYVTRAYRWGYMATRFMFERHRADVDAIVSRFRVGDYDGYADYVAYMGNRYDSEFVDWARGATTTGEPPLPPTKAGH

>AFR20596.1 serine metalloprotease [Burkholderia pseudomallei BPC006]

MSFLAGSAAKQAATAQHRRLIPFNFVWGSMSMSILIRTASFKATVLCAALAGLVSAAQAETAAAPQVPGPADAVNQLIVKLRAVKTPPGATAAKAERADVQAVIDRVLAARNARAAGRAFGAAAASAPGNPDDPAAGIRIKRDMSGGATVLSLQRHVSLAQAEALARDFAADGAIEYAEPDARMHPFVVPNDTRYSEQWGYFNPTAGANLPKAWDRTTGSARVVVAVIDTGYRPHADLAANLLPGYDFISDIPSANDGNGRDSDASDPGDWVSAQEDGDPSGPFYGCGASDSSWHGTHVAGTIGAVTNNGVGVAGISWVGKVLPVRVLGKCGGMLSDIADGMRWAAGLPVPGAPSNPNPAKVLNLSLGGYGRTCSSTYQNAINEITSRGANVVVAAGNNGGSVSTTQPANCRGVIAVGAIDSRGVRASFSNTGAAVKISAPGVGILSTLNAGKTSPGADSYASYSGTSMATPHVAGTVALMLAVNSTLSPSQILQRLQSSARPFSSGSSCSTSTCGAGLLDAGNAVDAAAQ

>WP_004185598.1 MULTISPECIES: 16S rRNA pseudouridine(516) synthase [Burkholderia]

MDLESILFTQGFGSRRQCRALVEAGHVAVGGATCADAHASFDTTQLVFEVDGVAWPYRARAYVALNKPAGYECSREPQHHSSVFALLPPQFAERGVQCVGRLDQDTTGLLLLSDDGQFVHAYTSPKRKVPKTYVATVRHPLDDAQLDALRAGVLLHGEPKPVAALAAVARGERLLEMTIAEGKYHQVKRMVAAASNRVEALHRSRIGGFALPEDLAEGAWRWLDERDLAALRDTAETLSG

>WP_004185616.1 MULTISPECIES: right-handed parallel beta-helix repeat-containing protein [pseudomallei group]

MMNRSQQTFAGFAAACAFAAGSAAQAWAAPADAIVYPAGNGSDQADALQAALDALQTGQRLVLAPGQYVVGRSLLVKNAQVVVSGYGATLVATNDADQTIEMRGRDSTLVGVTLVGTGATRLTTPESTKVDVTGAGVQVLDVEVRGGASAGIFVFGGTDVAVVGNTVRDTLADGIHTTYGSRNVLVRDNTVQNTGDDMVAVVSYKGDGKLSSNVLIQNNTLLGNYWGRGATVVGGADVTITGNTVRNVQKAAGILVGQEDPANTYDARNVIVSNNTISDIEWPDPDNTRPPAYMAAIDVNTWSGKATSVSVTDNRISRARYAGVRALGNVCQLRVSRNALASIDGTPIALQQAPSCAAGQIVCASNTLDGAALASPAGCSATDGLTITGANVARMPQVRAYLRQTAAPSAAKAGSAAASGE

>WP_004188402.1 MULTISPECIES: succinate dehydrogenase flavoprotein subunit [Burkholderia]

MAAIKTSLPRRKFDVVIVGAGGSGMRASLQLSRAGLSVCVLSKVFPTRSHTVAAQGGIGASLGNMSEDNWHYHFYDTIKGSDWLGDQDAIEFMCREAPNVVYELEHFGMPFDRNADGTIYQRPFGGHTANYGEKPVQRACAAADRTGHALLHTLYQQNVAAKTQFFVEWMALDLIRDADGDVLGVTALEMETGDVYILEGKTTLFATGGAGRIFAASTNAFINTGDGLGMAARSGIALQDMEFWQFHPTGVAGAGVLITEGVRGEGGILRNANGERFMERYAPTLKDLAPRDFVSRSMDQEIKEGRGVGPNKDHVLLDLSHIGAETIMKRLPSIREIALKFANVDCIKEPIPVVPTIHYQMGGIPTNIHGQVVGTSRDHKEPINGFYAVGECSCVSVHGANRLGTNSLLDLVVFGRAAGNHIVEHVKNQRDHKPLPADAADFSLSRLAKLEKSTSGEYTQDIANDIRATMQKHAGVFRTSALLKEGVEQMAGLKERAAAVHLKDKSKVFNTARVEALELANLIEVARATMVSAEARKESRGAHAHSDYEHRDDENWLRHTLWYSEGDRLDYKPVQMKPLTVESVPPKARTF

>WP_004188537.1 MULTISPECIES: efflux transporter outer membrane subunit [Burkholderia]

MTMRNDARHDERHQRRRGIARRAARHAAALALCAAVSGCLSLAPDYARKPPPVPAAYPAYPGEPGAGSRTLEQLGWRAYFVDPRLQALIETALANNRDLRIAAQRVEQARAAYGIRRADQYPTIAANAAYLRFREPGGLLLPSPIIGELYSASLSEVQWEIDFWGRVRSLKAAALDTFLASDASRRAVTVSLIGQVADAYLALCAYDERLALTNETIASRRDSLRIFRRRYEEGAISKLDLTQSEILLQQAQTLGSQLQQARDVQQHALDLLVGTNAAARTPASLDDASIAPDLAPGLPSELLANRPDVVAAEYQLRAAHANIGAARAAFFPRIALTSSIGTGSTELHNLFASGTAAWNFIPNLSVPIFDAGRNIGNLKLANARRDEALAQYEKTIQTAFRDVADALAARHWLADQVRIAQETLATQAERARLAKLRYDSGAARFLEVLDAQRDLLNAQQQLVQTRRALLASRVALYAALGGRLDEPARDGAAPRPSPEPSAKQGPLS

>WP_004189053.1 MULTISPECIES: DUF2501 domain-containing protein [Burkholderia]

MKKRIHRIAAAGILVAGVLSLSAAHAQLGDFLKQGADAGNGGAGGIAGALGNLGGGGGAASAGSLLTPGSTGNVAGLLQFCVKNDYLGDGGASSIKDALMSKLGAGVTSDSTYASGASGILDAGNGRTLDLSGGQSFKQQLTKQVCDKVLSQAKALL

>WP_004189371.1 MULTISPECIES: hypothetical protein [Burkholderia]

MKLLTQLAVASAVAGALAAAATAAFAQNSPGMPGGILNDQFRLNEHPQMQFAASAPTKKYQSGKKSDLRRKGDMGDPNGCNLKCPMD

>WP_004189720.1 MULTISPECIES: hypothetical protein [pseudomallei group]

MNRAIRMRAAVAAAAFSASLSAGAAGPAIDPGRYLYVEGGSAHGVLTVKGNAFEIETIGGNCHTCALSGTFDGRVGIARDGENVCRIAVSGGHGDLRLDTSGSDACRDFCGMRASFDGEYRRPGAACTDRARDVRTERSHRQYAAHDYDAARTTLKALLAECGGFMGWIELDRAKSDLALTEYHRGDRAQCVAVLSDTIAYRAQQDHSDAFGLPPCDADNYKSTGDAILHNLALCQAPAKR

>WP_004189892.1 MULTISPECIES: OmpA family protein [Burkholderia]

MNKLSKLAFIAATAVMAASASAQSVPASRQAVNDNWVNGTGEWVWMNGTNELCWRDAFWTPATANAKCDGALVAQAPAPAPVAPVAPAITSQKITYQADTLFDFDKAVLKPAGKQKLDELAAKIQGMNVEVVVATGYTDRIGSDKYNDRLSLRRAQAVKSYLVSKGVPANKVYTEGKGKRNPVTGNTCKQKNRKQLIACLAPDRRVEVEVVGTQEVQKTTVPAQ

>WP_004189909.1 MULTISPECIES: 3-hydroxyacyl-CoA dehydrogenase [pseudomallei group]

MEIRDNVFLITGGASGLGAGTARLLTEAGGKVVLADLNQQAGEALARELGGVFVKCDVAREEDAQAAVAAAAKLGTLRGLVNCAGIAPAAKTVGKDGPHPLELFAKTITVNLIGTFNMIRVAAAAMAANEPAQTGERGVIVSTASVAAFDGQIGQAAYAASKAGVAGMTLPIARDLSRNAIRVMTIAPGIFETPMLLGMPQDVQDALGAMVPFPPRLGKPAEYAMLVKQIFENPMLNGEVIRLDGAIRMQPK

>WP_004190112.1 MULTISPECIES: thiol:disulfide interchange protein DsbA/DsbL [Burkholderia]

MKKLLSSLFLSLSLVAGFAQASPSAPVAGKDFEVMKSPQPVSAPAGKVEVIEFFWYGCPHCYEFEPTIEAWVKKQGDKIAFKRVPVAFRDDFVPHSKLFYALAALGVSEKVTPAVFNAIHKEKNYLLTPQAQADFLATQGVDKKKFLDAYNSFSVQGQVKQSAELLKNYNIDGVPTIVVQGKYKTGPAYTNSLEGTAQVLDFLVKQVQDKKL

>WP_004192320.1 MULTISPECIES: EamA family transporter [Burkholderia]

MNPVSLFCIVTGVMLNACAQLLLKAGVRAVGHFEFTRANIVPIGFKIATQLPIIGGLGCYVLSVVVWIVGLSRVDVSVAYPMLSLGYVVNAFAAWYLFGEVLSVQRLVGIGIILIGVFVLARS

>WP_004192750.1 MULTISPECIES: phosphate starvation-inducible protein PsiF [Burkholderia]

MKIRSLMAALLVGGVLATPAFAANSQQDKMKACNAQAAGKTGDERKAFMKDCLAAKPAKKMSQQEKMKACNTQAADKKGDERKAFMKDCLSAKPAA

>WP_004193385.1 MULTISPECIES: fimbrial protein [Burkholderia]

MKKALLSAVACAALSTSAFAAGTGTLNFTGEIVAGACGIDAGSVDQTVRLGFVPANTFKAAGDKSTPQNFDIKLVDCDTSVAKNAYFTFTGTSNATQPKLIATVGSATNVGIRLQSASGEYLDNGAEQKGPVVLSNGTSVARFAAMYESTAASVTPGTADGVANFTVRYQ

>WP_004193508.1 MULTISPECIES: glycine zipper 2TM domain-containing protein [Burkholderia]

MDNQNTTTQQRQRLHPLVATAAGAVIVASLAATAAITGVFPKASSTNAQNAQTQAALIASQPAVDTAAAASAALAAQAAQQAAQQPQQTVAQAAPKPTSHQTTRHHHKTSAAPQPPQYAQQPYPQQQQSTYCASCGTVASIVPVRTAGTSSGLGAIGGAAAGGLVGNQFGRGNGRTAMTIIGALGGGLAGNAVEKQVRSETDYQVQVQMQDGSTRTFTYHNPPPFGQGQRVRVENGTLVGA

>WP_004193933.1 MULTISPECIES: PHB depolymerase family esterase [Burkholderia]

MTRNARRTWQWISGLLAICAAALAALPARADVAAGPGAWSSQQTWGADTVNGGNLTGYFYWPATQPTTPNGKRALVLVLHGCLQSASGDVIDNSRGAGFNWKSVADRYGAIILAPNATGNVYGNHCWDYANTSPNRASGHVGVLLDLVSRFVGDAQYAIDPNQVYVAGLSSGGGMTMVLGCIAPDIFAGIGINAGPPPGTTTLQIGYVPSGYTAQTAANQCKAWAGSKADQFSTQIAGAVWGTSDYTVAQAYGPLDTAAFRLIYGGTFAQGSKVSIAGGGTNTPYSDSNGKVRTHEIVVSGMAHAWPAGAGGDNANYVDATHVNYPAFVMDYWVKNNLRANGAPVQAGTPPTGLTVTNATQTSISLAWNPVANASSYNVYRNGNKVGSSTSTAYTDAGLIAGTAYSYTVTEIDPSLGESAPSPAVSATTQPSFACSATTATNYAHVQAGRAHDSLGIAYATGSNQNMGLDNVFYTNTLAQTSAGYYIIGDCP

>WP_004194152.1 MULTISPECIES: ribonuclease [Burkholderia]

MARKWLRNGALASVFAIVAMGNIGATPGSLVSAAYAREAATVDGAAAQVDTIPASRLPREAATTLSLIAAGGPYPYGKDGAVFGNYERILPKMRRGYYHEYTVPTPRARNRGARRIVCGGPLRRVDNCYYTGDHYNSFKRIVD

>WP_004194433.1 MULTISPECIES: polyisoprenoid-binding protein [pseudomallei group]

MNKQLMIAAGALAAALSFSAHAAPATYQFDPSHTYPSFEADHFGGLSVWRGKFDRSSGTVTLDRAAKTGTVDVTTKVASIATGSQKLDEHLQTADFFDSAKYPDATYKGTIKFEGDRPAEVVGNLTLHGVTKPVTLKIDSFKCMPHPMLKKEVCGVDAVGEFNRDDFGLDYGKQYGFKMKTKLLITAEAVKQ

>WP_004194773.1 MULTISPECIES: c-type cytochrome [Burkholderia]

MKKPQTALKTAAALALAAGFAIGTAHAANVAKGKELVESHNCAACHGAKLDNPINAEYPRLAGQHADYLVWAMRQYQMGLTNPLLGRNNAIMQAQVQSLSIADMKDIAAYLESLQGSLVFKK

>WP_004196853.1 MULTISPECIES: BON domain-containing protein [Burkholderia]

MSNSRVRQTLVRTTLVIGLSAGLAATLQGCVLAVAGAAAGGGALVATDRRTLGAQTEDREIQVKALTQMNNGLPDGSHVNVTVFNRRVLLTGEVPSDAAKQRAEEIVRAINNVNAIVNELAVGPASSLSDRANDSYLEGRVKTAMIAEKGISANNYKVVCERGNVYLMGLVTTDEGSRGADVASRVPGVEKVVKVFQYIKPQDAQALTAATPASGASAAAAPATAPEGATVGAVPDSSVTATPLSAPAPVSNSSNVHPGNPKAGTP

>WP_004197153.1 MULTISPECIES: sensor histidine kinase [Burkholderia]

MSHSLRGRLLWWLLLPLAVFVAIAGAMSYDTARKTADLVQDGALVASARVIAEDVDWEGGALVANVPPAALELFASPAQDHVYYKVRTGGGRLLAGNPDLDGPAAPAASGAQPVLFDTALGGLAIRAVAYTRELYNAGNTETVTVVVGKTQTSRQMMIAAIWHPQLWRLALMLALAMALVYLGLTFELRPLMKLKEDVADRGPMELEPIRTERLHFELRPIVDAINQCIAQLNLHAATQRRFIADAAHQLRTPIAVIDTQIQCARQRENGDAALAALLASMQRSSRRMADVTDKLLLLAHAEAASPARLAARVDIAAVVSGVLEEAIVLAERRRIDLGAELDDDLQVAGSESLLSALLMNLVDNAVRYAHEGGRVTVSARRDGDAVVLEVVDDGPGIPAEARPHVFKRFYRVARDEEGTGLGLAIVEEIAQSHGGAVSLATGPGNRGVRMTVRLPAYRN

>WP_004197194.1 MULTISPECIES: iron-sulfur protein [Burkholderia]

MKTSRRSFLITSVGAVSALALTREAFSADLPMLSETDPTAVALGYKADATKVDKAKFPKYAAGEACAGCMLYQGKKGSASGPCGAFPGKQVAAKGWCNAFTKMA

>WP_004198637.1 MULTISPECIES: flagellar biosynthesis protein FlhG [Burkholderia]

MDKRITDQAEGLRRLLAGRASRVVAVTGGPSGVGCTSTVANLAAALTALGKDVLVVDERANVRSIAATLCGSWLRDGEPVRHELGFAVCEASRLARVGYSDAQLDALGDGAADIVLIDAQLDANGALSALARDAHDVLVVTRVSASAITEAYACMKRLHYAHARAQFRVLTNHVQSAADARAAYENLAGVASRYLRVSLSNAGCVAADSLIERARGLAHTVVDAFPSAAAARDYRQIAADLLYWPMRPGPGIGRVRTGSSAFERGAAHAA

>WP_004204600.1 MULTISPECIES: GNAT family N-acetyltransferase [pseudomallei group]

MSVAPAARRPGVGAALLDAALAAAARTAGQVALAAVDGRGAARAFCERHGFVAYGVEPRALDAANGCADALPMVKFPPLAPPHA

>WP_004521493.1 MULTISPECIES: DUF2282 domain-containing protein [Burkholderia]

MSAKLSFHTALLVGAVASLVASGAHAAPLTKAEADAAVAAHKEKCFGVALKGQNDCAAGPGTTCQGTSTVDFQGNSWKFVQGGTCTSIQVPGGGHGSLTPLKS

>WP_004521540.1 MULTISPECIES: heme-binding protein [Burkholderia]

MSPELTLERALRIVEAGVAHAARLSAAGAAVAIAVVDAGAHLAAFARMDDAFIGAIDLATRKAGTAARFRMPSASLGALSGAGGPVRSIEHSNGGLVTFGGGLPLVDAHGRCVGAVGVSGGTVDDDAAIAQVCVDAFITLLNAAQGART

>WP_004522915.1 ammonium transporter [Burkholderia pseudomallei]

MRKILMSLLMAGSLLAGGIGAAMADDASSAPAAASAATASDTSAGAAASAPAASAAPAAPAAPAASAPAAASAAAPASAAAAPAAPTAPFSVDSSKINSGDTAWMLTSTALVLFMTIPGLALFYGGMVRKKNVLATVMQSFAITALITVLWTVVGYSLAFTPGNGFIGGFSRVFLSGMNYIHGDKATTLTVSHLASTIPESVYFVYQMTFAIITPALICGAFADRMKFSAMLVFMTLWSLIVYVPIAHMVWEPTGWLSADGVLDFAGGTVVHINAGIAGLVSCLMLGKRVGYGREAMAPHNLVLTLIGGSMLWVGWFGFNAGSAVAADGRAGFAMLTTQVATACAALGWMFAEWIAKGKPSVLGIVSGAVAGLVAITPAAGFVGVAGALVIGIAAGVVCFWSATWLKHKLGYDDSLDAFGVHGVGGILGALLTGVFAVKDIGGADGSLLLQAKGVAITLVYSGIVSFVLLKVIDIVIGLRVTEEEEREGLDVILHGEHVE

>WP_004523300.1 NAD(P)-dependent oxidoreductase [Burkholderia pseudomallei]

MEAIMKLGFVGLGVMGQPMALNLARAGTELVVWNRTRERCEPLRAAGAQVADSAADVYRRARIVILMMATDAAIDAVLDRGKPAFASNVAQHTIVQMGTVSAEYSRGLEADIRAAGGRYVEAPVSGSRQPAEAGRLVAMLAGEPAAVEEVRALLAPMCREIVATGQVPSGLLMKLAVNTFLIAMVTGLAEAAHAARGFGLDMKQFQAVLDAGPMASSVSRVKIDKLVNEDFEVQASIVDVFKNSRLATEAAHGAHLAAPLLEVCCELYRETEALGHGQADMAAVVHAIEARSVARYAGS

>WP_004523794.1 DUF3455 domain-containing protein [Burkholderia pseudomallei]

MTAALLTSATMAAHAAPPPPPELVPRDAARDRAFAAAGIQVYSCEYDGNHRLAWTFQHPEATLYDAAGVAVIKHGAGPAWEAQDGSRIVGEKLADAPSPNADSIAQLLLSTRASASGSLASVRYVQRLDTKGGTAPAAACTAEHQTGGSPYYARYVFWK

>WP_004523965.1 MULTISPECIES: hypothetical protein [Burkholderia]

MKTFSHALKRVVALVIAMPAAAGWASGQHRHGAADADELPRAFAAKEMGRSAAGARSYGSPVTSVFEEAKGALPAFDAKRSGAAGAAGFAHELGAAERRQRLAEEDRAVQRLRDSRHDGRGRWPGGAMPPRVPGRPDIAIEKPVPGVTCASGSDNSIGCGTGF

>WP_004524043.1 MULTISPECIES: hypothetical protein [Burkholderia]

MQRPSLIASSLLTASMFALSFAAHAQQTGIVRFTGMIVEPPCSFSVAGGAGTHAQLQPACPRPAKGSVTFVDPQSGAALRTVTFTELSRAIALPAGQTGDARPIVAVVSYL

>WP_004524330.1 MULTISPECIES: helix-turn-helix transcriptional regulator [Burkholderia]

MFSMLYFPMVSALSLLGADAPTHLHSHLKLILGGEFNAALERSSEWAETTVASERTSWDLQLHADLQLVLGFEVEAEENYRRAQRKIRGSNSKIRIATCRNAAWQALFRYRVTTALACFSRICDEPGIEAGGLVEARFGIACALYEMGRIDDAFDAIDSMEKIAEQQSDEMRAHWKDLIAVLRFDLVVQSELRRAAAFVDHVYWQSAQSMSRVDRAHGVSEAAVSVETPLLRGRVAYLLQLRCAAAGNRDAVAELARCLDAAGEQGFVDFRYTLRLEIALALLAGDAPNLAQFVLEPISDTLHGAESSRRYREYFYCAAKVHLAQDHTQESLALYRRYALIAMRCLREDALIGRQFLVGQELKQLPQSDDVTVRLPLKYRRAYHYILQNLNRSDLSVREIAAEIGVTERALQNAFKIYLGLSPRELIRSRRMERIRTELVDFTLTGERNVKEAARKWGVQNGSTLVIAYRKEYDETPSETLAR

>WP_004524503.1 transporter substrate-binding domain-containing protein [Burkholderia pseudomallei]

MKMNQTLLALACCAGLAHAQTAPQPGAPASRLDDVLARGTLRVCTTGDYKPYTYRREDGAFEGIDIDMAASLAKSLGVKTAFVKTTWPTLTDDFVAKCDIAVGGISTTLERQKRVFFTQPYVSDGKTPIVRCADAERYQTIAQIDQPQTRVIVNPGGTNERFAKQHLTRAKLTVFPDNVTIFKQILAGNADVMVTDASETLLQQKLNPGLCSVHPDKPFQFGEKAYMVPRGDVVFQQYVDQWLHLARETGELRAISDKWLK

>WP_004525656.1 SGNH/GDSL hydrolase family protein [Burkholderia pseudomallei]

MTSRRWFSALSCCLALAASQPGSAAQADAPARWVASWATALQPIPDLAAPPPLYRAPDVAGRTVRQIVYPTLAGKAVRIRVSNAYGKTPLAIGEMNIGRSAGGAAVAAGSSTAVTFGGRRETEVPPGQERDSDPVAYDVRAGEPYALSLYLGSRQTMTVWHRVSNQVNYVSAPGNHTGDASPDAFRTRFTQSAWIAELAVEVQQPGAAAIAAVGDSITDGLRSSLNRNRRWPDALAARLERAGAGDIGVANLGISGNRLLSDSRCYGIALERRFERDVLTRAGVKVAVLLIGINDINFAAMPARSGLDCDAPHTRVDAQALIAGYRRVIAAAHARGVAVFGATLTPASLPPAREAIRREVNEWIRTSGAFDGVVDFDAALRDPAKPSTLLRRYNSGDDIHPSDAGYAAMAEAVPLERLAAAAGRR

>WP_004525898.1 flagellar basal body P-ring formation protein FlgA [Burkholderia pseudomallei]

MTTDAGRATRTRFAVALALAGWMGAALAQQAGDGGMIVIPGRGESAQTALANANAASARGANGAGAAGPGGADRTASPGGWHAPAAAQAAAAWSGRAGAASGAESAAESGDVRAATEPRTGLIVIEPGPAESNGRIPAAAPSGWHPSAAMPRAESNGGANPVPPRTGSNRSAGSASASAGWTAPPGAASQGNPAANVAPAPRVSAASRAPATTDMQRVAPVIAQDEGAAPAGRPANARAAAQSWPARDPSVAAGGVIPVSLRAQPAPRTLPVRSAPIRAAATSAAGAQPAAASATAAGAVPAAQQDGESIRRAALAFLQQQAAGLPGKTTVTVAPAFPRGLAACTTLEPFLPSGARLWGRTTVGVRCAGERPWTIYLQAKLAVQATYYVAARQIAPGETLTAADLVARDGDLTLLPLAVITDAQQAVGATALMRVAAGLPLRRDLLKSAASVSIGQTVRVVASGQGFTISAEGSVLNNAAPGQQVRVRMAAGQIVTAIVKDAATVEIPL

>WP_004526045.1 family 20 glycosylhydrolase [Burkholderia pseudomallei]

MNRISHSLCAALLAAATLLPTASRAQLPARPTAGAAAPATAAPVRPASTPAELAARLANGLAVRVAVDNNHAASAGVPCADLGADWASCATGRLILQNRGHSPLTDGGWKLYLHSIRRLLRIDRPGFTLRHLTGDLYELTPQPGTVRLAQGERIELPFVAEYWLRRYSDVIPRPYVVVDGAAPAVLRYDDTDDELRYVETLPADAQNNSPGNAPPAAAQPVANRALPSVKRQRALPGALDLRGVELTLPELPSAQVAALRERAGTLGLDGARVPVWGVVAPRRLPADIAVPGGYRLAIGPRGAFIEGADRAGLYYGVQTLFSLVPAGGATVPAMLIEDAPRFTHRGMHVDLARNFKPPATLRRLIDQMSAYKLNRLHLHLSDDEGWRIEIPGLPELTDVGARRCHDPSETRCLLPQLGSGPDDRSGGGYLTRDDYVALLRYAAERFVEVIPEIDMPAHSRAAVVSMEARYRRLHAAGREREANAYRLLDAQDTSNLLTVQFYDRRSDLNPCMPGALNFASKVIREIASMHADAQAPLRIWHFGGDEAKNILLGAGFQPLDGADPGKGRVDLAAQDKPWARSPACTALLRRGEIKSIDELPTRFAKQVSAIVNANGIGTMAAWQDGIKHASGPREFSTRHVMVSLWDTIFWGASDSARDLSAKGYRTVLALPDYLYFDFPYTRNPRERGYYWGSQATDEYKVFSLAPENLPQNAEVFGDRDGNPFEVTSAGAAPSIEGIQGQAWGEVMRNGQLLEYMVYPRLLALAERAWHKADWELPYAAGVRYKLGDTHHVDTAALERDWAGFATVLKQRELPKLERAGIGYRKPTFTLTGE

>WP_004526282.1 DUF192 domain-containing protein [Burkholderia pseudomallei]

MRSSPRSSLARLARAAVFPVALALAASGMHAAFAQTTPLPPGAKQPSEFPRVKLRAGMYVIDAAIAANDADREQGLMYRSRLAPNEGMLFVFNENAVHCFWMKNTLIPLSIAFIRADGTITDIDEMKAETTDNHCPRNNGVYALEMSSGWFAAKGVKPGMKIDGLPKPQ

>WP_004526472.1 hypothetical protein [Burkholderia pseudomallei]

MDSFPASLRLLGALGKLTACAAGLSLALAAPAFADLLDQRAELVNKYVNEMHADPLVADCAAHGNFIASTSSAFDHVEFAPNAFDSGSASITPWNDSFDEGKQRVKVDNIVTVEGLGVRASGGDPEPLKFRCGYVGTQMLAFSWNDPVPPAKPRVERATSSKKKLRAHSGKGKVKAKATGKSGKRAVSKKSSTTKKAAKKRSTRKS

>WP_004526830.1 cellulase family glycosylhydrolase [Burkholderia pseudomallei]

MINAKTGFLFFIFVMSLIGCRPAFAQTNTPVTGIDLGGEIIEIIKQNPQDPTQAINNYMSGVAATNVRWVRIHLDWSAIQPASTWIFTSDPNPGRSSGLTWGATDIVVRAARNYGIRILGVITSTPRWATHAQCPSYYRQSTDDAYKLCAPNLTSYTNFARAAAKHYGNDSYGGKIDAWEIWNEPNCGPNFIPHDPVLYTRLVKSAYPAIKQANPSASVYAGGSSGCLTSPNNGTGALPSSGVAGLVNSTDARYPAPTQWEPRDWLAVMYANGARGYFDGLAHHPHCHSDDWQLPGDQCPSTSVNSVYPDYSNPFNIMWHTFTSPSYGWPAPTGKTFASYTGTSLRELMNANGDGGKPIVITEFGVATTASDGATDFTGQLGGSAQQYANADFQTIAPAYLTQANQEREYKALIAWIANQPYGQYGPVYAYCYSDMALSSPYSANIYEPYFGLVTITASTATSGTTGAPKIAGAYQAASGQYYPAWPNFGAAATAAANHTPGYAPVGPGW

>WP_004526889.1 dicarboxylate/amino acid:cation symporter [Burkholderia pseudomallei]

MKKRRNITSYIVIAMILGIAVGYGCHSAFPDPAIAKEIAGYVSLLSDVFLRLIKMIIAPLVFATLTVGIAHMGDTGAVGRVGVKALGWFFIASFTSLLLGLLAATLLQPGSHLSLPLPATDAAVNLKTSAFTLKDFVIHLVPKSIAEAMANNEILQIVVFSIFFGTALSALGDAGKRLTGVIEDLAQVMLKVTGAVMWFAPVAVFAALASTITTEGLGILLTFAKFMGSFYIALALLWGVLTLAGLVFLGKRTFTLIRLIREPFLLSFATASSEAAYPKLLDALDRFGVNRKISSFVLPIGYSFNLDGSMMYCTFAVLFIAQVYGVHLPLGTQITMLLLLMLTSKGMAGVPRASLVVIAATLNQFHLPEAGLLLIMGVDMFLDMGRSATNAVGNSIAAAVVAKWEGQLDAPRDDADSDSDSGDASRGSPRITQRV

>WP_004527347.1 MULTISPECIES: hypothetical protein [Burkholderia]

MKKTFAAIVSAGLLASSSAYADTLCTSGAITKIFIDNNGVMEVTVGTLAYLNGNKDVYSVLTSAFVANKQVYIYAPNCQPGTSMGGFAVR

>WP_004527508.1 MULTISPECIES: UDP-glucose/GDP-mannose dehydrogenase family protein [Burkholderia]

MKITIIGTGYVGLVTGACLAEIGHDVFCLDVDPRKIDILNNGGMPIHEPGLQEIIARTRAAGRIAFSTDIEASVAHGEIQFIAVGTPPDEDGSADLQYVLEAARNIGRHMTGYKVIVDKSTVPVGTAQRVRAVIDEALAARGLAGSAEHRFSVVSNPEFLKEGAAVDDFMRPDRIIIGVDDDAAGAIAREKMKKLYAPFNRNHERTIYMDVRSAEFSKYAANAMLATRISFMNEMSNLADRVGADIEAVRRGIGSDPRIGYHFLYAGVGYGGSCFPKDVQALIRTASENGQPLRILEAVEDVNHAQKNVLLDKIEKCYGADLAGRTFAVWGLAFKPNTDDMREAPSRRLIASLLARGATVRAYDPVALDEARRVFALDLHDGADALARLAFVDSADDALAGADALVIVTEWKEFKSPDFAHLKSVLKAPVIFDGRNLYEPDAMAELGIDYHAIGRPYVEPFSSERG

>WP_004527659.1 hypothetical protein [Burkholderia pseudomallei]

MRRASVPTLLFAASACFFSLNAFSVRATFAAYALLALLALLRAPTLFARQQRLPAIVAYWLLSVSSAFLVSAFGDFYANFFVKFVLIQTYVALAFWLFASGVLAMRSLERTCETLIYVHATFFIVQLGCYLAFGHFIDFDSYIRESDSEALYATKALSDSLISIRALGLYSEPSFYAMTVVPAGAILLLAKRRMTAATIVAFATALLSFSIAAILICALLGGVHFFAGRTSIRIKLVIAAVALAIAPAMYGVYDKRVNQSADYDALGSRTLVLRELRERDALADVFGSGLFWDERNNVGKTHLRGYQVRDSSFYVYLLFATGVAGATAFVGALFMLFRRRGRRSLLPYLLPLLLFKFHALYGMLWLTLLMFVVVAGHAERLPERREPPGTGTGRLSATGASGP

>WP_004527768.1 MULTISPECIES: TlpA family protein disulfide reductase [Burkholderia]

MNSKGIFAGIVVAAVAVAGGLAAGHWMRGNPASGPQAGTPAGAPAAGNPVDTLWAASYPDVDGKPQRLAAFKGQKLVVNFWASWCGPCVEEMPELVKLSREYEKKGVRFVGIGVDSDQNVKNFLKKVPVDYPIVVSGYAGADLARHFGNTAGALPFTVVIDETGKIRETKLGQIRPDELKRTLDTL

>WP_004528277.1 lipoprotein [Burkholderia pseudomallei]

MIRNVLIALVCAAPVAALAEPPKAAGGMIVDDDGMTLYTFDRDTMPGKSACTGGCTANWPAALADAYDKPGGDLGFIAAAGGKHQWTYKGRPLYRFSGDAKPGQHTGDGFGGMWHVARP

>WP_004528537.1 D-alanyl-D-alanine carboxypeptidase [Burkholderia pseudomallei]

MTTEPLSTTVARGSRRAACALLRTPSRLFASLFVSAACAAPNGARAAPAPAPAIQAASWLVVDADSGKTLGAHNVDARREPASLTKLMTAYVAFDALERDALRWDDTVTVAADDIASVGRDEARMYLTPGQRVRVRDLMRGLIVASANDAALVLAKRIGGSPAGFATMMNDTARRLGMRDSHFVSPSGITTPDHYSTAHDLSILAQRLNRDFPAFYTFSSQRHFAYGAFAKTNKNRLLGADPSVDGMKTGHTNAAGWCMVVTAKRPVGGARARHRVIAVLLGEPTEKQRLADARALLDQGFASLGADAPAANAVRRANARPGEHRAQARARML

>WP_004529035.1 MULTISPECIES: trypsin-like serine protease [Burkholderia]

MISCKLKALESSKIRISEKSSRTYQGIQMKNFVAKIYIVALGSLIVTSSHSQQIRTHGAVTQITLPEHGSQASSIDFKNAIPIPLPKPIMAPPTMLDSLMYNAKPSLGVPGYSPGGAGDGKQRPMRLVAPMDLNQIGIEPQEFGSSNQPYTTSQVNAYGDNTQYYYPFRPAGKLFFNIGSASYLCSASLIKRGVVVTAAHCVANFGQRQFYSNWVFVPDYSNGSAPYGVWSAASATVMTSYYDGTDSCAVSGIVCQDDVAVILLAPQGGSYAGNSAGWYGYGWNGYGYNPSGLALIDQLGYPVALDSGVFMERNESQGYVSSGYSNNTIIGSLMTGGSSGGPWLVNLGMPPTLSGTTFGNSGAHNTVVGVTSWGFTDLAVKQQGASPFTSSNIVTLVNTVCSGTPAAC

>WP_004529236.1 MULTISPECIES: DUF411 domain-containing protein [Burkholderia]

MAQLPEDKPVSRARRAIAAGLALLPVVAFARKTATKPVVQVWKTPTCGCCEDWLSHLKNNGFDVVVHNVEDTSDVRQQAGMPERFASCHTGIVQGYALEGHVPAREIKRLLREKPNAVGLAVPAMPIGAPGMDGPVYNGRHMPYDVLLIKRGGQSSVYQSYS

>WP_004529364.1 peptide ABC transporter substrate-binding protein [Burkholderia pseudomallei]

MRRALPFRYHYQSHTMKHTHAFAAVLAALALTIAPSAPAVTVASNVTLADQQDLTRQVPAEVESLDPAHIESWTGNTIGLDLFEGLARIDASGAVVPGVAQAWEHKAPDTWVFKLRRDAKWSNGQPVTAADFVYAWQRLADPKTGSKYTILVEFVKNASAIIAGKQPPGDLGIRAIDPYTIEVKTEVPVSYFPELTAMAPLTPVNKDAVAKFGDAWTRPKNIVSNGPYTLVDWQPNNRIVMAKSDKYWNARNVVIRKVTYLPIENDETALRMYQAGQIDYTYSIPAGGFGQISKQFGKELRPGLQLATYYYYLKNSDPALKDKRVREALAMVLDREILTSKITQAGEVPMYGLMPKGVKGVQRPFMPDWASWPMARRVDYAKNLLKQAGHGDANPLTFTLTYNTNDLHKKVALFAASEWRTKLGVTAKLENVEFKVLMKQRHDGKVQVARDGWFADYNDAMTFFDLIRCGSSQNTVGYCNPKVDSLVAEANQKLDDGARAALLTQAHDLAMNDYPMVPLFQYSADRLVKSYVGGYTLTNYIDMRASQDMYLIKH

>WP_004529401.1 MULTISPECIES: class I SAM-dependent methyltransferase [Burkholderia]

MSPDAYLQMAATEATHWWFRARREILQALLAGLGLPAGARILEIGSGTGGNLGMLEAFGAVSALEMDDTARAIANRRTGWRFDIRAGRCPDDVPFGDQRFDLICFFDCLEHIEDDAAALRRISDYLAPGGAIVVTVPAYQWLWSGHDTFLHHYRRYDRAALERCARAAGCTIVRSSYFNTLLFPLAVVARTIDRLLHRARSSGDALGPRAVNAALYRIFRTERGWLPKHSLPFGVSLFAVLTKCSEPR

>WP_004529879.1 oxidase [Burkholderia pseudomallei]

MIIYLDRRALEVADGTTVAAALALAGDDTARTSCTGAARAPFCGMGVCHECRVTIDGRRRLACQTLCREGMQVERTR

>WP_004529973.1 copper oxidase [Burkholderia pseudomallei]

MKMVSRRTFLGGSGAALLGAALVSKAGAASLPEAPTMAKAATQPPLAPPNGRPYTPVVTLNGWTLPWRMKNGWKEFHLIAEPVVRELAPGMNAHLWGYNGQTPGPTIEAVEGEKVRVFVTNRLPEHTTVHWHGMLLPCGMDGVGGLTQPHIPPGKTFVYEFQLEKAGTFMYHPHADEMVQMAMGMMGTFIVHPKDRGAMPADRDFVFLMSAYDIDPGSFTPRVNEMTDFNMWTWNARVFPGIDPLPVRAGDRVRIRFGNLTMTNHPIHLHGYAFEVAGTDGGWIAPAARWPEVTVDVAVGQMRAIEFTANRPGDWAFHCHKSHHTMNAMGHQVPNLIGVPQQDLAKRINKLVPDYMAMGSTGGAMGTMEMPLPENTLPMMTGTGPFGPLEMGGMFTVVKVRQGLGRNDYRDPGWFRHPKGTVAYEYAGALPDD

>WP_004530215.1 DUF4148 domain-containing protein [Burkholderia pseudomallei]

MKATFLTVALAAAFVSPAAFADAPAGKTRAQVYQELVEAKANGLDYVTDASYPEISPLYAPRFANKAKPQQPAHAVADTRSDAKAGSSGNLSEAAQDEHCVGPRTFCNPYAGS

>WP_004530759.1 alginate export family protein [Burkholderia pseudomallei]

MIARRAVALGGWALMSVLAAARAHAADAMDAPAAAAAAAAPGSRFARVSATSESAPSSAFEFASPAASASTSASALAVVPMSALTLASAPAPAPAPSAAPSPSPTSSPVQPGVRPGASCAKTRPAILFNRWQEDWSALADPCVPRRPLDALKYVPLFGRVDSYLSLGAGLRERLELNDAPLFGLGRARGDTYVLQRVQMHADLRIAGHVQAFVQLEDARPFGKDNVGPVDRNRVDLRQAFVTYVDAIGSGAFKARVGRQEMAFDLQRFVSVRDGPNVRQAFDGIWADWEQGPWRLIGYATQPVQYRDDGAFDDVSNRNLTFSGVRIERQRVGPGDLSAYYSRYNRTQAQFPDGAGGEHRDVFDVRYAGKRRNVDWDIEGMYQTGRVGAQRIEAWAVGSLAGYTFAGVGWMPRIGLQVDAASGDRRPRDGRIETFNPLFPNGYYFALAGYTGYTNLIHVKPSLTLKPSSALTLLAAVGLQWRATTADAVYAQGATPVPGTAGRGGNWTGFYTQLRADWAVTANLAAALEVVHFQIGDALRAAGGRNADYVGAELKFGW

>WP_004531414.1 2-dehydropantoate 2-reductase [Burkholderia pseudomallei]

MDRAPVRAAIVGIGAIGGLFAAGLARAGWDVSALARGATLDALRGRGLRIVGESGGETAVALRASDDAHALGEQDYVVVALKAHALPALASRIAPLVGPRTIIVAAMNGLPWWFFDGFGGPLDGAALDAVDPGGVTAAALPPARAIGCVVHLSSATREPGVVVRGRGNRLIVGAPRAALREPGERVADALAAGGFDVERSADIRTDIWAKLWGNMNMNPLSALTGSAADRLLDDPHTNALALRMMEEADAIGARLGLSAGMSGAERIAVTRQLGAFRTSMLQDLEAGRALEIGPILGVFPELGRRLGVPTPYCDAVLGLLRQRAFNAGL

>WP_004531547.1 MULTISPECIES: ABC transporter substrate-binding protein [Burkholderia]

MLEIKHKLVQTAAACALAFAACAAHAADKPLKSIGITVGSLGNPYFVTVVKGAQAQAKEINPSAKVTAVSADYDLNKQFTQIDNFISAHVDMILLNAADPKAIEPAVRKAQAAGIAVIAVDVAASGADATVQTNNVKAGELACDYLAKKLNGKGNVIIENGPQVSAVVDRVNGCKAVLAKNPGLRLLSSDQDGKGSREGGMNAMQGYLTRFPKVDGLFAINDPQAIGSDLAAKQLRRTGIVITSVDGAPDIETALTSDTQVQASSSQDPFAMAKKAVSVGYGIMNGHKPANAMILLEPTLITRENVKRYKGWSAH

>WP_004532251.1 MULTISPECIES: urea ABC transporter permease subunit UrtB [Burkholderia]

MPLSRAARALAALAACAAFSFAAPRAALAVTAADVAALAGDDFDAKRAAIDRLAAGHDAAAAALLNALANGDALATDAGRILIQHGDAARDALTNAPAQAGDAQPVMLNNLLRTKIANALSGLDLASPDIDTRRRAIDALLKRPDAALKPMIDAARAKETDPVLKRRLDALWAIAALRDADPAKRLEAVRLVAARSDLDMIEQLRPLVAKRPDGGDAEPDARVREAAQQGLGALYAIQRRGEIAGTLFAGLSLGSVLLLAALGLAITYGLIGVINMAHGEFLMIGAYATYVVQTLVQRYLPGAFDWYPLAAIPVSFAAAAALGIVLERTVLRHLYGRPLETLLATFGVSLILIQATRMIFGAQNVQVVNPSWMSGGVTVMQNLILPYNRLAILAFALVVVGIAWAVLTKTRLGLFVRAVTQNRRMAACVGVKTARVDSYAFAFGAGIAGLGGCALSQIGNVGPDLGQSYIVDSFMAVVLGGVGQIAGTVLGGFGLGLVSKAIEPFWGAVLAKIAVLVMIVLFIQKRPQGMFALKGRSAEA

>WP_004532434.1 MULTISPECIES: class I SAM-dependent methyltransferase [Burkholderia]

MARRKLERWSALDLAEGLQLAHAVAALQTLGVVDAMAEPTTAQTLSAACDLDPELLRGVLEYAASRTNLIRKTGDRFSASEHYTAQARFLLNLYAGAYADNASRLATLLRRPALAGTTVDLVAHARAFEASEAGGGALAAIISQLHLNHVLDLGCGAGTLLHALAADDREFVGWGLDRNPSMCKAARMRARQAGIAARVKVFQGDGRHPGASIPPRVLARVRNVVASQFVNEMFRGGTSRAQAWLRRMRRLLPDRLLVIADYYGRLGHGFQTPHRETLLHDYAQLISGQGVPPPNADAWLAMYRATGCRPLHVIEDRAATTRFVHLVAL

>WP_004533337.1 F0F1 ATP synthase subunit gamma [Burkholderia pseudomallei]

MSDKLAAIEARTDTARQLQTVIGAMQGVAAARAHEAQQRLPGIRASAATVGAAIGDALSAGARSGDTAAGARAAPRARLVVVLCSEQGFVGAYNAQLIEHATRPEPSVSREYMMVGSRGAMLADAGGVPLVWRTPMAARADDVVHLANRITDALYAHLAKRGAQPVSIVHAMPGGAARLEVTERRLLPFDYARFDTAPRAQPPLVHLPPATLLAELAQAYVFVELCEAAMLAFAAENEARTRAMIAARESVERALGDLLQAYRIARQDEITADIVELAASGL

>WP_004533800.1 D-threitol dehydrogenase [Burkholderia pseudomallei]

MTNLWDRAFDLTGRVALVTGGAAGIGHACARLLAQRGASVALVDRHPETAGIAATLEGGAARHSGMSLDLRDCSAAQAGVALAASRFGGVDMLVNSAGVALLDKALDVGEAAWDATMAINVKASFFVAQAAARQMIAGARGGRIVNLASQASVVGLERHAAYCASKAAIVGMTKALALEWAPHGITVNAVSPTIVETALGKQAWAGEVGERAKREIPAGRFAQPDEIAALVLYLLSDAAAMMTGENVVIDGGYTVR

>WP_004533895.1 D-amino acid dehydrogenase [Burkholderia pseudomallei]

MRVVILGSGVVGVASAYYLARAGHEVTVIDREAGPALDTSFANAGQISPGYAAPWAAPGVPLKAVKWMFEKHAPLAIRLDGTRFQLQWMWQMLRNCTTERYALNKGRMVRLAEYSRDCLQALRAETAIQYEGRTGGTLQVFRTQQQLDGAAKDIAVLREANVPFELLSSDELKKAEPALAAVSHKLTGGLRLPGDETGDCQLFTTRLAALAEQLGVKFRFNTRIDALAVAGGKIAGVQCGGEMVRADAYVVALGSYSTNLVASLVKIPVYPLKGYSITAPIVDAAKAPVSTVLDETYKIAITRFDDRIRVGGMAEIVGFDKRLRDARRGTLEMCVNDLFPGGGDTEKATFWTGLRPMTPDGTPIVGRTPVPNLFLNTGHGTLGWTMSCGSGQLLADLMSGKKPVIRADDLSVHRYLSETDGEHRPAYA

>WP_004534049.1 Bcr/CflA family multidrug efflux MFS transporter [Burkholderia pseudomallei]

MPHVARRRPDGRLILLLGALAACGPIATDMYLPSLPAIAGGFGVTAAAAQRTLTSFMAGFSIGMLLYGPLSDTYGRRPVLLGGIALFTLASVGCFVAGSIDMLIAVRFLQAFGAGAASVLARAIARDAHEPADAAKVLSMVAIVTAIGPLLAPLIGGQVLRFSGWRGVFVVLALFGAVCATAAYLRVPETWPREKRASSAVLNSFAAYGRILVDPVAWGHMLCGGMAFASMFAYITATPFVYIDYFHVSPQHYGLLFGLNVIGIMLGNFLNARLVGRVGSLKIIAGASLLSGAASFAVAFFALTGLGGLWSIVASLFFVVGVVGILSANCTTDLMLRYPHNAGAAAAVFGAMQLALGALASAAIGALANGAPFAMGVTIGATGLLCLGGRMLVLRWHGRPVKGALGRVDAA

>WP_004535146.1 TonB-dependent siderophore receptor [Burkholderia pseudomallei]

MLIAAHPGAAALAGTAAKSDAQKNDMQRNENVREDTRRLAAGAPPAGGELKAISVSAPKDAADDPSVATVGKMPLALREIAQSVSVTTRERIEQQNLFSLDDVMQQSAGVTVQPYVLLTTAYFVRGFKVDSFEFDGVPVVLGDMASAPQDMSAYERAEILRGANGLLHGSGNPAATVNLVRKRPQHRFAASASASVGSWGRYRAQADIGGPLNPAGTVRGRLVAAYEDRGFFYDRAKQDTRSIYGIAEIDVARDTLVTVGAQYQSVASVPDMSGVPMARDGTSLGLPRSTFLDVGWGHFDWDTTRVFGSVERELGGGWKAKVSGEYQAVRSDLKYAGAYGAIDRATGAGGTLMGSAYQFSSYSRSVDANVQGPVRAFGLTHELLFGATYASSSSGQLTAPLLAGAGTPVNVYRWNPDGVPEPGVGPYRQDMQNDISQKGVYGLGRIKLAQPVTLVLGGRLSWWNQDSLGAHYNAGRQFTPYGGLIWDVARDWSWYASYAEVFQPQTKPTWDGGILTPVKGRTYETGIKGELAGGKLNVSLAAFRIDLDNNPRIDATRPCAGRSCYYVNGGSVRSQGFEFEANGRITPWWSVWASYTFDTIRYAKDVANGGAFAAELTPRHLLRVWTNYDLPWQERRWSVGGGVQVQSDFSAASSGVTMRQGGYALASVRLGYRYDRHWRAALNINNLFDRTYYQSLSQPGWNNRYGEPRNVMLTVRGQF

>WP_004535373.1 sn-glycerol-3-phosphate ABC transporter substrate-binding protein UgpB [Burkholderia pseudomallei]

MKYKMLVRSLAFGGALWFGAQQAACAATEIQFWHAMEAALGERVNEIAAQFNASQSDYKIVPVFKGTYDQALAAGIAAYRSGNAPAILQVYEVGTATMMQAKKAVLPVSDVFRQAGVPLDEKAFVPTIASYYSDARTGRLVSMPFNSSTPVLYYNKDAFRKAGLDPNQPPKTWADVKADAEKLKKAGYACGYTTGWQGWIQLENYSAWHGLPFATRNNGFDGADATLEFNKPQQIAHIQFLQVMAKDGTFTYVGRKDEASAKFYSGDCAIMTTSSGALATIHKYAKFDFGTGMMPYDAGVKGAPQNAIIGGASLWVLAGKDPATYKGVAKFLAYLSSPAVAAKWHEDTGYLPVTTAAYDLAREQGFYAKHPGADTAIKQMMNKPPLPYTKGLRLGNMPQIRTIVDEELEQVWAQKKTPKAALDSAAARGDELLRRFEKSGG

>WP_004535631.1 ABC transporter substrate-binding protein [Burkholderia pseudomallei]

MRFTHIAAAALVAAASVAAAKPLTVCTESSPDGFDVVQYNSLVTTNASADVVFNTLVSYDEATKKVVPALADKWDVSADGLAYTFHLRPNVAFQTTDSFKPTRALDADDVVFTFGRMLDDANPWHKVAGASGFPHAQSMGLAKLVKAVTKIDESTVKFELNEPNATFVPILTMGFASIYSAEYADQLLKAGKQADLNAKPVGTGPFVLKSYTKDAVIRYEANPTYWGPKPKVERLIYAITPDPSVRAQKVKAGECQIALSPKPQDVAAAKADRALRVVETPAFMTAFVALNTQKKPLDNDKVREALNLAFDRATYLKVVFDNTATPATNPYPPNTWSYAKSIAPYPHDPAKAKQLLAQAGFPNGFSTTIWVRPTGSVLNPNPKAGAELLQADLAKIGVKADVRVIEWGELIKQAKLGQHDLLFMGFAGDNGDPDNILTPQFSCNSVKSGLNFARYCDPKLDKLIADAKETADQAKRAKLYEAAQKIIHDEALWIPLGYPTAAAITRPNVAGYRVSPFGRQHFDAVSVQ

>WP_004535805.1 c-type cytochrome [Burkholderia pseudomallei]

MTIAKRVKRTMSAAAAAMAVVSCAMAATPAAHADAGDGLKVARSNACMGCHAVDRKLVGPSFQQIAERYKNDKQAEPKLAKKVKDGGSGVWGAIPMPAHPRMSDADVRSVVQWVLAGAPSK

>WP_004536717.1 cytochrome c [Burkholderia pseudomallei]

MLKRTLSFMLAGCLALPGLSSAADAAASGAPASAASSVPAASAAPAAVRAADATLVERGRYLAVAGDCMACHTAKGGKPFAGGLPMRAPLLGTIYTTNITPDKETGIGDWSFADFERAVRHGVAKNGDNLYPAMPYVSYTKVTDDDVKALYAYFMHGVEPVRQPPRRNDIPWYLSMRWPLKIWNLLFLKEGVYQPKPERGVEWNRGAYLVQGLAHCGTCHTPRAVTLQEKSLDETGGSFLAGSVLSGWDGYNITSDPNAGIGGWSQPQLIQYLRTGSVPGLAQAAGPMAEAVEHSFSRMSDADIGAIATYVRTVPAVADGAAKARSAWGKPAEDGIRLRGVALASTGIDPARLYLGNCASCHQMQGKGTPDGYYPQLFHNSTVGAPNPTNLVQVILNGVARKAGGEDVGMPAFRHELSDAQIAALANYLTVQFGNPAAKVSEQDVAKLRAAQ

>WP_004537213.1 D-amino acid dehydrogenase [Burkholderia pseudomallei]

MRICVLGAGVVGLTSAYCLAREGHDVTVLEARPDAVLDASFANGGQLSYSYVAPLADPAVLGKLPAWLARRDSALRFVPRLDVDQWLWCAAFLAACRTARARRTAAEMLELGALSRIALHALVERETLDFDYARNGKLVVYRDRREFDRARRAMDHLIAAGARQQALDTAACVALEPALAPASGLLAGGIHTPSEEVGDCRRFGIELARVLRERLRVAIHYETPVDTLRTEGATIVAARTPRGEIAADAFVLSAGNGSAPMLRRLGMRLPIYPLTGYSLTLPARHDATPRVSVTDLHRKIVYAPLGARLRIAGMVEIAGLRSSGAARRLALLAQQAQEIFPHAGDYAHGETWCGHRPATPDGKPLIGATPFRNLWLNTGHGALGFTLACGSARVLADLIADRPPALDTRAYALHR

>WP_004538457.1 MULTISPECIES: cytochrome c [Burkholderia]

MEASVHKHLIVKFRSACANAALGLLAATLAASAAHAQPAADAATLKRGEYLARAGDCVACHTVPGEKLFAGGRAMPTPFGTLYSPNITPDNDAGIGKWTADEFYTMMHTGRSRDGSLLYPAMPFAAYTKVTRADSDAIFAYLRSVPPVKLANRPHDMRFPYNNRQLLIGWRTLYFKEGEYQPDHTKSAEWNRGAYLVQGLGHCSMCHTAINALGGSSEANAFEGGLIPMQAWYAPSLTSNKEAGLGDWSIADITGLLQAGASHRGAVYGPMAEVVYDSLQYLSDDDVRAMSVYLKSLPQRGSDAESAPKTTMAASEQARLSKLGAKIYDAQCASCHGKTGRGKTPAFPPLAGNQSIQMTSAVNPIRMVLNGGYAPGTAKNPEPYGMPPFAQSLSDDEVAAVTTFIRTAWGNRGAPVSAKEANALRSAPLY

>WP_004538458.1 MULTISPECIES: c-type cytochrome [Burkholderia]

MTVTSRPTAVLAVLCGLLSQTALSQEPQTERAPDTMEARVLACAACHGRQGEGTSNDYFPRLSGKPAGYLYNQLVAFRDGRRKYPPMNYLLAYLPDAYLRKIADHFASRRPPFPTIAAPAAAPAVLERGRLLVKSGDPSHKIPACASCHGASLTGMEPAVPGLLGLHAEYLSAQLGAWRYGTRTSIAPDCMQQVASRLTDTDVTAISAWLASQPAPANPSPAPAGSLDMPLACGSEPR

>WP_004538839.1 hypothetical protein [Burkholderia pseudomallei]

MNLHRSIVVPLVFSATFLAGCGSSVDGRFTSGMVAMGAVAGSTVAAYPIGADGTIGAQPLAVTTSDASGAFSLPALDRWPALIRATHGAYVEEATGAAATLEGDGLEAVYASAPSTIVVSPYSSAVVATARAAGGLTQANIAAAIATISAFVGDFDPQQTRPATMAAGAAAPAIEPGHQMALALGAESQSRTDAAADIATSIAAIVAQAAAGDTLDTCHAGAGDPRADGTLAAPVSSGCAITRGAARYAANARNTSGVTSLASLSAARTSRASPDQTAAATPDACADRAALLAQNLALFDGRRDDVQANLVGGVTRDNWRTVTTRSTWGPTAAFYGTLATPAACADTDTFARELVIAAENYWIDQGINYCHHHIPGWTPPDDSAAAAPRYRNSSAGSTSGASSNGMTCTAQRSGTGAQIVPGTAPSAGFSASEIQWNGVDCSDFTSWIYNFVGLTGVRLPTAIGSQACAANEGADALPTPGVLLDIDSGNIDAMLPALKPGDLLYITQVDPLASGSDAGGYQLAHVVTWTGKRWSDLQAGPDAARYALARLGQPGSRLGGDLAKYLPLADLATQNPWMIIDSHYAGPAYRPFVGWYRRSLSNVRRIVGADAARRDPALAPYVIAPIASDARGDTLTLASPHANASAQQGWRMIYRQSGGTPSCVRAGIAQ

>WP_004540441.1 MULTISPECIES: molybdopterin-dependent oxidoreductase [pseudomallei group]

MLQLSRRQFLKLSATTLAGSSLALMGFSPAEALAEVRQYKLARTVETRNTCPYCSVGCGILMYGLGDGAKNATSSIVHIEGDPDHPVNRGTLCPKGASLIDFIHSPSRLTQPEYRAAGSDKWQPISWSDALDRIAKLMKADRDANFVETTDDGMKVNRWLTTGMLAASAGSNEVGYLTHKTVRSMGMLAFDNQARV

>WP_004545891.1 branched-chain amino acid ABC transporter substrate-binding protein [Burkholderia pseudomallei]

MMLSRLTSMSLAAMLVAAGAAAHAETVKIAIAGPMSGSVAQYGDMVKAGALTAVEQVNAAGGAGGNKLEVVMMDDACEPKQAVAVANKIVSQKIKFVIGHVCSGSTIPASDIYENEGVVMITPSATAPQLTEGKKRKFIFRTIGRDDQQGPAAAQYIINKVKPKKVAVLHDKQSYGQGIASAVKKELDAAKVPVVIFEGINAGDTDYSAIVTKLKSQGIDFVYFGGYHPEMGLLMRQAREQGVKAVFMGPEGVGNKDVTAIAGPASEGMLVTLPADFTIDPANAGVMKAFAQKKRDPNGTFQMPAYSGVKIIADSIAGAKSTDPAKVAAYMHQHTFDTPIGKVAYDAQGDLKSFKFVVYKWHKDATKTAAN

>WP_004546221.1 multicopper oxidase family protein [Burkholderia pseudomallei]

MLRRHFLSSALAAAAASLFARGAFAAGQAMDGMQGMEGMEGMDDMPDMKPAPAHARHGKPAPGPASALAAADALPAGAPLAALRVLANESREPGVFRATLVAQPVARALLPGAAPTTLWQFGADTQGPAVGPLIDVREGDAVEIRFVNRLPQPSTIHWHGLPVPPDQDGNPSDLVAPGATRVYRFTLPKGSAGTYWYHPHPHMATAEQVFRGLAGPIVVRAADDPLAGWPERHLFVSDLKLARDGAIAPNDMMDWMNGRQGQFALVNGARRPRISLTGDERWRVWNGCSARYLRIAFDDGRPFAHVGTDGGLFDAPREVASLLLAPGERAELVVRAGDRASHAVLTALEYDRGKMAMAMSEAAHGSLPPDPALPLADVAFEPAAPRALPARLRAVPALGEPVARKEVVFGEQMDMAAMMRAGAHGRPAGMRFMVNGATFEPHRATLTSRRGEIESWTIRNETDMDHPFHLHGTQFQVVEREIDGETTPEPYRAWRDTVNVRKGERVRILTTQTERGERMFHCHILEHEDLGMMGTLKVV

>WP_004546662.1 hypothetical protein [Burkholderia pseudomallei]

MKSLQIAVVALSLSAVMATAHAQPAAADAGRQPANRTEAVQAAGRVPAAHQDRQDPNACVGPVSFCNIYFGS

>WP_004546872.1 DUF3472 domain-containing protein [Burkholderia pseudomallei]

MRRSELKFNCIAATILAAVAADATAAGACLNGSTIASTTRAPLVARQGSVFSSTLYDPAITSNNRTHNPVMLTVKVTNNGRPVAGCDVAWQPRGAGGASGWLFPASASTDANGIASAWWVAGSGAAQTAVASIRRFDGTTQGVAIGGSAQPHATRANSIHLNYEPASDWTAFRVDVTPEALAPTTYWEAIGWPGAYTGIQSIDGKQDGLVLFSVWDVNGKSPQIIAKGPGVDCTQFGGEGTGYKCAKRHAPVAGRTYRFMASIAPVAGQNQTDYSVWFTDTSTNARELIATLRYQKAVQSANYANSFVEDWATQGASCLGATQRAGQYGNVWALDRASAQWRTVKRASTSAVYTPDHNEVCSNYQFSVVNGRFRMSTGGHAVGQPLNLPNGPKSFPLTLP

>WP_004547886.1 MULTISPECIES: DUF1853 family protein [Burkholderia]

MSAAASPVPPVALRDAAVRDLAWLLSSASLLAPSAGAPLAQPWASVADAARTAAWLAALDAQPAPLHDALAHARPVRLGRYAECLLGFFVGHAPSLRLVAANLPLRSNGRTLGECDFLIETARGERLHWELAVKCYLCVATAGTASLADFVGPNLADRFDLKRAKLVDHQLRLTARDEFASLGHAGPWRAQMFVKGWLFYRADDGANAAGAVADPPEIGADHPRGFWITREAWPKFALAAARRDVAWIVLPRLAWLAPRAFDDADVACGRCVPLASDAVIDEVARQSGPLLVAALARDASGGWRETARGFIAPDDWPRRASAFAAR

>WP_004548048.1 TonB family protein [Burkholderia pseudomallei]

MKTYSTYLALPLAASLLAGCAAFAPRDAAKLECTMPVAAYPENAKPLERRATVLVRAMITASGNAENVTVTTSSRNAAADRAAVDTMSRIACSQTPACGGEPYPFTLTRPFVFEPRAKAPQ

>WP_004548157.1 M48 family metallopeptidase [Burkholderia pseudomallei]

MQAKLRVARAALTFVGGVALGVAGPVLGFELCMAEPMLVADNGAPPVPSAPAVATPAAPAAAPAAGNPQPYALGDQQVRYGNAIVFRSLIPSPLLEQLTDNEYRQTVQDAAQRKRLLPPNNARVKRLRTIVMRLAPYAVKWSERVKGWNWEIEVLRSRSIRAFCLPGGKVLVDSGLLERLRLTDDELGVLFAHEIAHALREHARSSLGEQQAASLGTGATPLPPLFGLSEPLPAPLGVVERFASVRYDPTDETEADVIGGDIAARAGFDPRAAITLWDKLAVATRADKDNGFIHAHPYDTRRRNDLRKRLADLMPLYRKALVKNADARANAAGAGAAAGAKQRGAATANR

>WP_004548306.1 sulfatase-like hydrolase/transferase [Burkholderia pseudomallei]

MSTIKRIAAAAFAAAALSGGFGHAAHAAGQAKNVIFFLGDGMGPATVTASRLYKVGEAGQLTMEKLPRTARIKTFSNDAQTTDSAPSMAAYMTGVKMNNEVLSMSPDTRAIAPGSDANGNKTVNRCGVGNGTPAATLLELAKARGKAVGAITTTELTHATPAATYSHICHRDAQYDIAAQAVPGGAGYNAALGDGVDVLMGGGRNHWTPYDPLANRRGRADGRNLLAELQAKGYAVVATKDQLAQAGAGKLIGLFSTTSHLEYELDRVAGKGEGATQPSLAEMTAKAIDVLRKNPNGYFLMVEGGRIDHALHGTNAKRALEDTVAFDEAIRTALAKVDLSDTLIVVTADHDHTMTINGYSKRGNPVLDISRNYRDGQPNKDADGNPYTTLVFGNGANRPNARVPVDSSTATNDAYLQEVGVRMGSAGSETHGGGDVMLFADGAGAKAFKGTLDNTKVFGLVKAAFGF

>WP_004550304.1 MULTISPECIES: hypothetical protein [pseudomallei group]

MRAMFCAAAFAVPLTAAAFTAGDLDKLCAKTDVKSRASCAAYIEGAADGVYNTIDAIGGTTGPRVGQYFCLPPDIKAQQMTDAVRKYIAENPKLADYNASTAVSLGLGKAFPCRSY

>WP_004550328.1 SCO family protein [Burkholderia pseudomallei]

MNETSMMRRKALGALCSLALAGAPVGSRAATPFYSLPLSSKDWHKGFRLTDLHGHTKTPQDYRGNVLLLFFGFLSCPSICSTTMLELTQAKERVGAQKDKVKILFVTLDPNRDTSPTIARWLASFGDDNIGLRDSEAHVRKAATALNLKYERVEGDVPGAYTIDHGVQTYVFDPQGRLRLIARAGIEPEYVAKDIVQLLSGR

>WP_004553586.1 S8 family serine peptidase [Burkholderia pseudomallei]

MNRTADFALSLSPLCKRLACVWPLALAAGIAHGATDWVDTHTKAFLNHAQIETLARGANAASLEVASGEATHVVVSLKLRNAEQLKAVARNVNDPHSAQYRQYITSAQFLANYAPTEAQVKQVVAYLRKNGFVDIHVAPNRMLVSARGTAGTVKQAFNTSLVHFEYAGRAGFANASTAQVPRALGDIVGSVLGLQNVARARPLTKIGAIAKPLALASGTATGHYPSEFPALYNATGVPTAANATVGIITIGGVSQALSDLQQFTSANSYPDVSTQTIQTNGSGGNYSDDQEGQGEWDLDSQSIVGAAGGQLGQLIFYMADLDASGNTGLTQAFNQAVSDNAAKVINVSLGWCETDANADGTLSAEEQIFTQAVAQGQTFAVSSGDEGVYECNNRGYPDGSNYTVSWPASSPHVLAIGGTTLYTTSSGAFSNETVWNEGLDGNGKLWATGGGVSTILPNPSWQSGSHRKLPDISFDAAQSTGAYIYNYGQLQQIGGTSLSAPIFTGFWARLLSANGTGLGFPAARFYHSIPTHASLVRYDVTSGNNGYSGYGYKASTGWDYPTGWGSINISNLNQLIQSGGFN

>WP_004553625.1 polysaccharide deacetylase family protein [Burkholderia pseudomallei]

MSFRSRFTWRTAVFGPGFRPVSRIVATTASSGAPAVAAPPTDVAVLVYHRFSNVCGADPMTVGVATFEAQLAHLRRLGYRFVPLRDVIGWLRGEPVALPSKAIALTIDEGHASIFDWARTVALRERVPITLFVYPSAIGEAPGALTWHQLRVLHKTGWFDVQSHAWWHPDLNAAHRPPSGTFREATRAQFAQARARIAREIGNQVDLLAWPFGAFDGELGAAAREAGYVAGFTLEPSKIRRDTPLLTLPRFLMVEECTPAVLRRLLSKSDAAREEAARR

>WP_004554341.1 MULTISPECIES: nitrous oxide reductase accessory protein NosL [Burkholderia]

MKRRFLSAAVRTSIAALASAALVAACGHDAQTPPPAREITDATVSVLDGMSLKDYPGPKAQIVYADGEPDFFCDTLGLFSVYLRPEHDRKVRALYVQDMGATDWQHPVGHWIDAKRAIYVIGSKKPGAMGRTFASFAREADAARFAKAEGGKLYRFGEITPEMAATDGGVVKDQTM

>WP_004554469.1 SCO family protein [Burkholderia pseudomallei]

MLAGAALVSLPACAAESREIAHRPWGAIEPAEAAPDIEVSLADGTRGRLVDLLRGKTSAIQFVFTGCSATCSLQGAIFQSLQAQLARHPIGGAQLLSISIDPANDTAVAMTAWLRRFGAQPGWQAAIPASGDLARLARLYRDERNPADSHIDQAFIASRSARFVWKTDHLPTPESVHDALRYYASH

>WP_009918239.1 hypothetical protein [Burkholderia pseudomallei]

MRAATSNPIASNIARRTANHGYFAALYSCAMKIVRLLLVLLWCATLPLTGLAASGLAGDCPMQQAMSMSEDGAMPSMQDCESMRSSVAGKSGKAGKAANVFCKVMVQCQFGSLYHPMPAADVARPASPGRPLVFHYAKSLTVREPGGLWRPPRTA

>WP_009950543.1 ShlB/FhaC/HecB family hemolysin secretion/activation protein [Burkholderia pseudomallei]

MTTATRFAVLPITALITLAAQAQQTPATIDQAAAARANAEQNQQVQQRRDAQRRDATVQAPGVRSDVPSPEAYPVLPAETPCFRIDRFALDVPDSLPAAAKVQGASALPMDRFAFARDWLAHYAGQCVGKQGVDLIVKGLSQAILTRGYITTRVLVPGQDLSTGTLKLALIPGVIRHVRFEDDKLRGTWKTAFPTRDGDVLNLRDIEQGLEQMKRVSSQDVSMRIAPGDRPGESDVVLDAKRGKPWTVVASIDNSGTRATGKLQGNVSLGIDNPLGLNDIFNVGFNQDLEFGDKRFGSHGWNAFYSIPWGYWTGTLSAYTSTYFQPLAAVNQTFVASGNMKTVDFRLNRVLARSRNDVFGAQVRLTRRFGDSFIEGTTIPSSHQNATFLEFVLNDRHYFGSSQFDGSLAYRQGLGWLGSTDSMFAVEGGQTYRFKMVVLDANLSTPFVIGTQPFKYVTTFHGQYTGNTVSYLDSVTIGSRYTVRGFDGERLLAASRGFYWRNELQAPIARTGLSAYAGLDYGRVWGPEPVALVGTQLAGAVIGVKGSVSTRFGAYAYDLFAGTPVYKPSGFETARVTLGFQLTAQF

>WP_009952932.1 SDR family oxidoreductase, partial [Burkholderia pseudomallei]

AEATADAVRAAGAQACVVRGDVAHEADVIHMFDAVQSAFGRLDALVNNAGIVAPSLPLADMDIARLKRVFDTNVLGAYLCAREAARRLSTDRGGGGGAIVNVSSIAARLGSPNEYVDYAGSKGAVDTLTLGLAKELGPHGVRVNAVRPGLIATEIHASGGQPGRAERLGAQTPLGRAGDADEVAETIVWLLSDAASYVTGALLDVGGGR

>WP_009956690.1 glycerophosphoryl diester phosphodiesterase [Burkholderia pseudomallei]

MGQELKTRNILWRIVLLGIVSLSMVTMTSNVCVAAPGFPAIVAHRGGTGDAPENTVYAISKALQNSADAVWITLQLSSDGVPVLYRPTDLKVLTSGSGPISALSAAQLARLDAAYYYNPKDGYPLRGKGYGIPSLEEVLKTFKDTFFYLDIKSPDADPNRMATALSQVLERTGALSRVRIYSTEAKYTAAVQAMPHFETRDETRTALANVTMNHTCQLSPKFGSWYGYELRRDVTLIEETTLGVSPPSPSRLVWNLEAKTCFIDTGKGSILLIGVNSADDYATAASLGAAAVLVDSPAQARHWPTQVSMP

>WP_009966818.1 hypothetical protein [Burkholderia pseudomallei]

MKNRYLAALATCAALLPIASTARGEEVASVNTNFRLTGSDRVVVEAYDDPLVSGVTCYVSRARTGGIKGTLGIAEDPTEASIACRQVAPIRFTEPLRQQTDVFSERLSFIFKTLHVVRVVDKKRNTLVYLTYSDRIATGSPKNSVTAVPVPAGTPIPLR

>WP_009966896.1 carbohydrate ABC transporter substrate-binding protein [Burkholderia pseudomallei]

MKIRAMMGALGAAGLLFGAAAAQAAENVTVLHWWTSGGESKAVGVLKDDLQKQGYVWKDFAVAGGAGAAAMTALKTKVISGDAPSAAQIKGPLIQEWADQGVLVNIDAAAGDWKQNLPPEIDKIIKYKGNTVAAPFSVHRVNWLYINKAALDKIGAKPPATWPEFFQVADKLKAAGIQPVAMGGQPWQDLTLWEDVVLSQGADFYRKALVELDQKTLTSDKMLEVFNTVRKIQGYFDSGRNGRDWNLATAMVINGRAGMQFMGDWAKGEFEAAGKKPGKDYICAAVPGTANAYTFNVDSFVFFQQKGQKAATPGQIALAKTIMTPAFQEQFSLLKGSIPVRLGVKMDKFDDCAKKSYADEQTAIKSGGYVPSLAHGMAQGDATAGAISDVVTKFMNSQQDAKSALAALARAAKVK

>WP_009972253.1 MFS transporter [Burkholderia pseudomallei]

MHPLRSILPSALFTAVGLLATDLYLPAVPSLPQQLGGSIESAQATLAAFSAALAVSQLVWGAAADRFGHRRTLAFAVLLQLVAGAACALAPSMGALIGARLAQGFGVGAAMVIVPALVRQSFGDGGAVRALAWLGIVESAVPGLAPLVGAALLVVADWRTSFWIIVALSAIAAPLVFRVIPTARAMRACAPANVGAHAGGYRRLLRSPVYLGYALGHALCFAALLAFVASAPQVVEIWLGAGPSTFSLMQACGVAAFMLSAARSGKWSDALGLDRIIALGALLQFAASAAFLLLAYADWRSTPLVVASWMLFCGSLGLRGPASMARALAAEPAVAGRAAGLLMFFGLGGAALATQAVAPFLRLGLAPVAWMCAGFTLASGAVVLWGIAIRGRHRAAATEIA

>WP_009981055.1 ABC transporter substrate-binding protein [Burkholderia pseudomallei]

MKLKTLAHACLAVAAAWSVGAVQAADSVKIGFITDMSGLYADIDGQGGLEAIKMAVADFGGKVNGKPIEVVYADHQNKADIAASKAREWMDRGGLDLLVGGTNSATALSMNQVAAEKKKVYINIGAGADTLTNEQCTPYTVHYAYDTMALAKGTGSAVVKQGGKTWFFLTADYAFGKALEKNTADVVKANGGKVLGEVRHPLSASDFSSFLLQAQSSKAQILGLANAGGDTVNAIKAAKEFGITKTMKLAALLMFINDVHALGLETTQGLVLTDSWYWNRDQASRQWAQRYFAKMKKMPSSLQAADYSSVTTYLKAVQAAGSTDSDKVMAQLKKMKIDDFYAKGSIRTDGSMIHDMYLMEVKKPSESKEPWDYYKVVATIPGEQAFTTKQETRCALWK

>WP_009981467.1 esterase family protein [Burkholderia pseudomallei]

MRASNHYAVMRRFVSPLVSPLAALLAALLAAALTAAAHAAPASSIVTRTFRSPALHRDWSYTVYLPAGYNPEGARYPVLYLLHGNAGNANDWITQGRLQLTADALIERRDIAPVVIVMPQGGTDWYVDRKEKMQSAFLDDLIPDVEAHYAVSNQRAGRAIGGVSMGGYGALRFAFLEPERFCGAMLLSPAIYANEPPASSAARYVGVFGDRQFDPKVWHELNYPALWRGYFAQPLRLRMFIAAGDDDLSIQAESSALYTSLRRAQNPAALRIVDGAHTWDVWRRLIGPALKYTLECVK

>WP_009981622.1 hypothetical protein [Burkholderia pseudomallei]

MIHIRLKRASFLLLLGAQAVSLGAAAGTLSGTVSSAGTPLAGAMVTVFDAAQARRDTVYTDHNGRYRITVDFAGELRVRARTPYFKDAAQDLALAPDASKTLDFSLARQTVADELSASLPASAHLATLPWSSQDSRTAFISQCNYCHQVGNALTRTPRDEAAWGATVRRMEGYAALLTDRQARDITHTLYQGMNAHAVAAVEKYLYDDRLAPAKIREWAAGDGLTFIHDADVGFDDHLYGADEGHDKIWELDRKTGRLTEWKEPDVDLPVGGIFSGVQLPIGVFSGKHGPHSLAQAPDGRFWITNALSSTLASFDPATKRFKLYELGHSHLYPHTLRIDRAGIVWFTIVASNEVARFDPKTGRFTIIHLPDGGIWRAMSHYLFPLVVKMAAWFPGQNLHLALTHHKWAFQGRDAFPFPYGIDVNPVDGSIWYAKLYANKIGRIDPKTLAVTEFDTPLGGPRRLRFDPQGNLWIPAFDDGGLMRFDTRTHRFETFKLPLLAHNEYEVPYALNVQPKTGDIWITSNMSDRIFRFVPSTQTFITYPLPTRVTWLRDMTFTQDGAVCSSSSNLPAYGIEGGRASFICLYPDGERGGTAANAAGNANAAANLARVRAAAARRTPERTAPARAALARAGSNRDGAIQTAAIQADGTRTDRSAH

>WP_011203825.1 MULTISPECIES: chorismate mutase [pseudomallei group]

MKQSLRASLAAAVLGCIVLSAPRIAAADGDDTALTNLVALASQRLALAEPVAHWKWLNGKPISDPPREAALLADVEQRATANGVDPAYARAFFDDQIAASKQVQNALFATWRATHGPEGPAPDLATSTRPQLDRLTQSLIAALARVAPLRDAPDCPSRLARSVSNWKTLTRYDSGREDALGTALSHVCTAGSTSAVG

>WP_011204325.1 MULTISPECIES: tRNA (adenosine(37)-N6)-threonylcarbamoyltransferase complex transferase subunit TsaD [pseudomallei group]

MLVLGIESSCDETGLALYDTERGLLAHALHSQIAMHREYGGVVPELASRDHIRRALPLLEEVLAASGARRDDIDAIAFTQGPGLAGALLVGASIANALAFAWDKPTIGIHHLEGHLLSPLLVAEPPPFPFVALLVSGGHTQLMRVSDVGVYETLGETLDDAAGEAFDKTAKLLGLGYPGGPEVSRLAEAGTPGAVVLPRPMLHSGDLDFSFSGLKTAVLTQMKKLEAAHAGGAVLERAKADLARGFVDAAVDVLVAKSLAALKATRLKRLVVAGGVGANRQLRAALSAAAQKRGFDVHYPDLALCTDNGAMIALAGALRLARWPSQASRDYAFTVKPRWDLASLAR

>WP_011204795.1 MULTISPECIES: prolyl oligopeptidase family serine peptidase [pseudomallei group]

MMTGWIAACVLSAAQADPLPSDAPSAPAAAPYAPRVSHAPRAAGALANAERFDYGDSGLPPVAANLNETIIRIPVDAAGAITLEATVYKPDGPGPFPLVVFNHGKNPGDLRAQPRSRPLSFAREFVRRGYAVVAPNREGFAGSGGTYIQEGCDVERNGVAQARDVAATIGYMSKLSYVDARHVVVAGTSHGGLVSLAYGTEAARGVRGIINFSGGLRQDLCEGWQKNLVDAFDTYGSRTHVPSLWLYGENDSVWSPALVAQLRDAYMSHGASTLFVDFGRYKDDAHRIIVDRDGVPIWWPPVASFLAQLSLPTSVRYAVANPHEPKASGYAAIESVDAVPFIDDAGRAAYRRFLAQHPSRAFAVSSEGAWSWAEGGDDPMALALEGCRKQGAGACQLYAVDERVVWRDAGTQTADESTSAAHALASR

>WP_011205039.1 OmpA family protein [Burkholderia pseudomallei]

MEKTGRGGGMNSTNVFGGIFVGMLSVALLGCASVEKPAAYGEPSPMFIFASNEGFVAPSKMAFPQGIPIDPDALKKLQLGMSKDQVRDALGDRQYNGEDLPIWHRWDYLFQLRAPDGAVHQCQYQVRFLGGLAYRGYWADSACRDIAARYADAAGLGVIAVAPCNPSDVAAPLPERVELPTDTLFAFDKGGFEDISADGRRQLGDLVASIKAKIFSINHLIVTGYTDRLGSDEHNARLSSERARTVADYMIAEGIPAAKITAVGRGAADPVVVCNNGEQPELIRCLQKNRRVEIRIKQK

>WP_011205373.1 ABC transporter permease subunit [Burkholderia pseudomallei]

MASKERDVQRSFLERSLLRRFGAAPAGGLLALFFVLPLAALVSAAFAEGGRAFAAVLHDPLVGDAIGRSLALAVGTGTLSTCVGVPLALSFAEQPPARRRWLLALLGVPLAFSGLVIAYGFILTFGRAGFVTTLLAGLGADAAKVGGVIYTTFGLVVAYAYYLIPRVALMLFPAFANLERRPLEAAMSLGAKPWRAWLDVAWRELWPSVMAAWCLVTAIALGTYGTALALAGTQINILPLLMYLKLSDGQTDFSQAAVLSIVLTALCTCVLAMGEYVGRRHR

>WP_011852052.1 lipoprotein [Burkholderia pseudomallei]

MKSNSSLSILIAAACIQAFAATASLAQGPAHPPSYVEGTRVPKGFARPPFHTNPARFSATTVSGLAPATVRHAYGFDSIANQGDGMVVAIVDAYDDPKIESDLGVFSKNFSLPPCTTSNGCFKKLYASGSKPSPNAGWALEMSLDVEWVHAIAPKAKIVLVEAASNSFNDLMTAVDVAVGAGASVVSMSFGGSEFSSETSFDSHFGAPSNVTFVASSGDSGNGTEYPAASPYVVAVGGTTLSADASGNYVGETAWSGSGGGVSAYELEPVGQTLWPIPYAGQRGVPDVAYDANPNSGFAVYDSVTYQGQSGWFVVGGTSAGAPQWAALFAIANSMRTAAGKAKLAGAYNQLYTVGKTAYGSDYHDVTSGTNGSCGMICTASGGYDYVTGLGSPQALNLVQALVAQP

>WP_017881844.1 MULTISPECIES: sugar ABC transporter substrate-binding protein [Burkholderia]

MQRKTLTAAAARVAALAALASSALAAQAATLTIATLNNPDMIELKKLSSAFEKANPDIRLNWVILEENVLRQRATTDITTGSGQFDVMAIGTYEAPQWGKRGWLAPMSNLPADYDLNDVIKTARDSLSYNGQLYALPFYVESSMTFYRKDLFAAKGLKMPEQPTYEQIAEFADKLTDRANGTYGICLRGKAGWGENMAYVSTVVNTFGGRWFDENWNAQLTSPEWKKAINFYVNLLKKNGPPGASSNGFNENLTLTASGKCAMWIDATVAAGMLYNKQQSQVAEKIGFAAAPVAATPKGSHWLWAWALAIPKTSKQQDAAKKFVTWATSKQYVEMVGKDEGWASVPPGTRQSTYQRAEYKAAAPFSEFVLKAIQTADPTDPSLKKVPYTGVQYVGIPEFQSFGTVVGQAIAGAVAGQTSVDQALAAGQAAAERAVRQAGYRK

>EQA86889.1 DeoR faimly transcriptional regulator [Burkholderia pseudomallei MSHR338]

MQAAALAAEFHVSEDAIRRDLRALAAEGRCRRVYGGALPVTPACAPMAARIDAARERKAALARTAASLIERGELLFLDSGSTALALVEYLPEDAELTIATNSIDIAAAVLRRADLSLIMIGGAVDQAVGGCVDASAVQSVARMNIDRGFLGACALSPQRGLAAFGLADATFKRAVVAASARCVVLATTDKLAARAPHRVAALDEIDCIVVEHDLPREDRAALSSAGASILAANPPAQP

>EQA87624.1 ABC transporter substrate-binding protein [Burkholderia pseudomallei MSHR338]

MGAATGAGAARADASQTLRFGLEAQYPPFESKAANGALQGFDIDVGNAVCNAAKLTCKWVETSFDGLIPALQGRKFDAINSAMNATDQRRQAIDFTTVIYRVPTQLIARADSGLEPTPASLKGKRVGVLQGSIQEAYANAHWAGAGVQVVAYQDQNQAYADLTAGRLDGTLVLAPAGQRGFLSRPEAKGFAFVGPPVRDDKILGSGIAFGLRKGDDALKARLNAAIDKLKADGAVKALGRKYFGDIDISAK

>WP_023360415.1 tetraacyldisaccharide 4'-kinase [Burkholderia pseudomallei]

MSARPGLLARAEARLTREWQRRGALAWALAPFACAFGAIASLRRAAYARGWKARVDCGVPVVVVGNVTVGGTGKTPTVIALVDALRAAGFTPGVVSRGYGAKIAAPTAVTAASPPQQAGDEPLLIARRTLAPVWVCPDRVAAVRALKAAHPEVDVVVSDDGLQHYRLARAVEIVVFDHRLGGNGFLLPAGPLREPLSRRRDATLVNDPYSRALPPWPDTFALSLAPGDAWHLDRPSRRKPLAQFAGERVLAAAGIGAPERFFATLRAAGVAPATRALPDHYAFATNPFVDDHFDAILITEKDAVKLGTSWRDARIWVVPVEAALDPRLIALVVEKLRGRTSA

>AHE31311.1 methyltransferase domain protein [Burkholderia pseudomallei NCTC 13178]

MQASDPSSGYLSDVTFPDRFHRELSPTWLNYASVLGGARPKELGRPFRYLDLGCGFAHSTVINAAAFPHAEFHACDFNPAHIEAAARRASRLGIGNVAFHEASFDALLDRDLPPFDFIVMHGIYSWVDAGMRRVIRQLLSRRLADAGLVYLSYNCQPGWAAEAPLRKLMLELAQAADGGIEARTGSAIAGMRKLGTPSLRYFRDNPAAAEALAALANDPLDYLAHEFLNGTWKIHYSVDVVDEMAEAGLAYAGSATLADNHPMLLIDRQAADAIAALPNARLRHLAEDFAVNRRFRRDVFVRGARASTAPAEALRHLDEIAIGCTTEIDRIDTRVTIPRGAISFQPDFIADLRALLRHGAMRIGEIVARLGAARRNSREIRQNLLFLVASGTLTPFAQPGGPTDTGARRAASPAAAAALAGSVDDAAPAFVPSELLGNGLAVSPDEAAQALRWIAGEAMPRPERLARVGVLRGA

>AHK68005.1 bacterial extracellular solute-binding family protein [Burkholderia pseudomallei MSHR520]

MNTIRLLGAAALVGACAPLAAAAATPVCKVPTLKVLAQKSLGLSVMEKSLPDYEKTSGTRIEINYFGENDRRAKSRLDASTGAGSYQIYYVDEANVAEFASAGWIAPLLKYYPKEYDYDDFLPGRRAVASYKGVAYFAPLIGGGDFLFYRRDLLDAAHLPVPKTLDELVAAVRKLNAPPKLYGWVARGQRGSGMNVWRWAPFMLAQGGAWTDPHGQPAFNSPAAVQAAERYRDLFKYAPPGAATYDWSNALEAFRSGKVAFMIESTPFADWMEDPSKSSVAGKVGYARPPAPLPSAAYGHGLAISSVGAKDDCTRQAAGRFIAWATSKEQEQARLRNGVFSDYNRTSTIGSDYFRQHVKPQILAGLNDTNPVTKATIWATPQWPDIGDNLGVALEEVFTGTQTDVRGALDDAAQYAKDAMAHGARRAQVTRVTQVARGTRRRDRQMKARRRDAAPRPRRPQPPRPRRCLAARASASQETPMFNHGKTSLPWLFLGPPLALMAVLGLVPTIAAINLALKNRVLRYADSDYVGLRNFVRLASDRRFLNAIEVSALWESVTVAGAVAVGIVLAVFLFERVHGRWRHAAALILIMPVLLPRVSAAFIWKFMYAPLTGILGWLLGALGMTNVAWLADPRLALAAVALVDVWQWGLFFCVVVLKLLETLPPEPLEAARLDYASTWQVYAYIALPMLKAPIMSLVFIKMVESLRSFDLIYVMTKGGPGIATETLDMYAYAQGIGLSGKVSYASSMAVLMMVATTLVFTFIWKRVDKWDD

>WP_024428428.1 type II and III secretion system protein family protein [Burkholderia pseudomallei]

MDTKPQLSRPTPARSRAVRGAALAAACCAGLFTVFAAAAQDAVENAAIAKGTAAAPMALGRAPMQMTISMAPAPAAGAAAAAAAVPLRGPSCVGEMRESTSVSVPLGKSLLVPLDEPVRNRTIGNPAVAQATMVSPRTLYLVGMSVGTTNMIVQGKSGACRIIDVAVGADAGGLQASLRQLMPRERDIRVSTAADTIVLAGNVSSAQAAQQAVAIAKAYSNRTNAGAAGSGGGARADVLNMLTVTSPQQVMLEVKVAEVSKTLINQMGSAVNIQGGFGSWTGALVTSLLTGVGSGIAASKSNKLPLNGSIDAQNTDQPVKILAEPRLVTISGQEATFLAGGKIFIPVPQSSGIGTNTITLQEEEFGVGLKFTPTVLSNGRISLKVAPEVSELSPTGVTLSAANVGNVSILPLITTRRASTTVQMGDGETFAIGGLIKDNAQGTLKALPGIGELPVLGALFRSTSFQQDRTELVFIITPHLVQPLETADVPLPTDSFTKPNEADVYATGNMEGRGGLRQHGAAPAAAPANAAPGAAAPTGAAPAAAVPAPAAPAPAAPAAPAAAAAPAKDAAASPAPAKQAPIAAQAPAPQPAKPAASAPALANARPPATVQAPPPALPSAAALAQRQGAHAAAPDTAHAGPAESAAATASPAPAAALAAPAAAGATAKRQGAQPPQQLANAGAIAPAADR

>WP_024428446.1 AAA family ATPase [Burkholderia pseudomallei]

MSVVDERFAWTGGLADRLPEPADFGLALAEGFARRIGMLSRRVGASAAAARWAARAAFAASRATAAGHVCVALRALAQRYDEPYDDVRAALAASGVTAFDGIVRGGECPLVVDRDGRLYLARYFEYETRLANALVERSRCGGAPAGGADALSPDALGERLVRYFGPQKERGVDWQRVAALIALTGRVTIVSGGPGTGKTTTVVGVIACLLDAHPELRIALAAPTGKAAQRMQEALHARAGSLPAELAARLPRTSCTLHRLLGGGPGGRFAHHRDNPLPYDLVVVDEASMIDVALAAHLLDALAPNARLVLLGDKDQLAAVEAGAVFAELSARPAFSPATCATIARALGVGEAEFVAALPDGFVVQAVGAERVADARAAAADMDVAARGTAGAAAAAARATAAAAARMTTAAAAARGGAPESAAQRAPAPHAGRRGGGRASNRTVDDAQGSLFAFDDDAFGVGTSGGEPGAGAQGAGRRDGGAPSLDGPHAGRRDGGASPGVAHSRRDAFDARARSGPADVSDLENAARAYAPAGAAGPADSPADASFVEPAAWIEADELAWLDNAVFSFSEGASANAEADGRRSAAATATDAGARTAPPVAREATAPTPRETARSDGSAVAPLTDCVVWLERNYRFGLDSPIGRLSLAIRRGAVQDALDALSTQDDAAARFSDDGGATLSAATVEQLARGFAGYAAALRDALATPEPDALPLFDVLNRFRVLCATRTGARGAEEVNARMAAEVRRAVRVPLALGAHWFAGRPVMVTRNDYALGLFNGDIGIALPGARGVLRVWFRGADGRARAVSPAALPPHDTAFALTVHKSQGSEFDDAALILPASFNRVLSRELVYTAITRARARVQVIGSRAVLALAIATRTARDSGLAARIADALRARQEGAR

>WP_024428496.1 hypothetical protein [Burkholderia pseudomallei]

MRTMIAAGALALLAGCAGGPAGFGSWGGPSFAQLQRGCGDVRDYGDDARSVYSAVFDAWVAKCHGKLTDARFCAFENELAQRHAALGTNADAAARGRWVSYLNDARARALSWRAAVDPSLRGG

>WP_024428498.1 FAD-binding protein [Burkholderia pseudomallei]

MKKILGDPASRRERRAFLGDVARLAGAGIIAGWTPIRPIAAHAQAAGAAPPNFPAGIALYKQAFRNWSGEIAVADLWTAAPATPADVVAIVNWAADNGYRARPLGHMHNWSPLTVAANGAHERTLLVDTTRHLCAVSVDPSTTPARVVAQAGVSLDTLLATLEQHGLGLTAAPAPGDITLGGALAIGAHGTALPAANETRPPGHTYGSLSNAVLALTAVVYDTASSRYALRTFDRADPDIGPFLAHVGRAFVVEATLQVGANQRLQCESFVDIPAAELFAAAGTRGRTVESFVQRSGRIEAIWFPFTDYPWLKVWTVRPNRPSGARVVEEPYNYPFSDSISRELSDLVSRIVLNGEIQLAPLFGKTQYTIAYLGLTNIFRPLTNLWGWSRSVLHYVRPTTLRVTANGYAVLTRRENVQRAINEFVGAYRQRVAAYRAAGRYPMNGPIEIRVTGVDTPDDIGRGAVPPSLSAIRPRPDHPEWNAAIWFDILTIPGTPDANRFYREIEQWMLSNYSGDYATVRPEWSKGWGYADTAAWSDDAMLRTTIPDLFRQGLSSADDWDAALRTLERYDPRRVFSSPLLDRLMG

>WP_024428546.1 alginate lyase family protein [Burkholderia pseudomallei]

MVRQTFPGRAQALRQRLSALAPALVAAAALAAAGPARAAMNFCAAPALQSSEATHAEPGVQALIKSVDAHLNDEPKALPRVHTEGTLPHEGIYDQSAEALNDMELIRNAALAWRVTNQSRYLALVDRFLSTWVNTYRPSFNPIDETRFESLILAYDMTASALPVKTRNAAAAFIAALGNGYVQQIDAQKRPLKGTWRNNWQSHRIKLIALAAFTLGDRRMMNAAQRLFVEHLADNIEPDGTTYDFLERDALHYAVYDLQPLATAALAARRFNRNWLRERAPNGATLAAALDWLAPYARGEKTHEEFVHSPVPFDAKRREAGLPGYSGMWEPKNATELFHLAARLDGRYAGIAQQLSPMPPAWLAACLPLPAR

>WP_024428578.1 metal-dependent hydrolase [Burkholderia pseudomallei]

MASHAAHHASGWAAGLVAAALVAQAGAAGPWHVYSLAAFAAGVAGGTAPDWLEIAWWRRTRRLWVTHRTITHWGVGWAALLAFAYRSLGHRHPWAPPLFGFACGGLVHLIADWPNPLGVPWLWKRHSLNWWNSGHCDLIVVAAAWAGALWLAQAAWGHAAPLAHWLGWLRTA

>WP_024428614.1 nitrous oxide reductase family maturation protein NosD [Burkholderia pseudomallei]

MPIPHALATLAAAALLMHAACAATLAVHPGERIGAALAAARPGDTVLVQHGRYEENLRIDKPLTLRGVGRPTIDGRLAGDVIRVAAPDVTISGFSIVDSGASLTAQNAGVYVAPGSDRTRIERCELVYNLFGLWIERSADVRVTGNVIVGKRDLLSPRRGNGIQLYNTTGARIAGNTISYTRDGIYVDVSHHARFEHNTIHDVRYGTHYMNSYYNVWDGNDVYHNRGGLAIMEARDQIVRGNRVWGNTDHGIMLRTIQDSLIENNVVAGNQRGLFIYDAEYNTIRGNLVVDNKIGVHLWGGSIHNDVTGNDFADNREQIRYVAASDVAWPGNYWSNYLGWDRRGRGIGDVPYKANDLVDRLTWRVPSVKVLMNSPAVQALRVVARQFPLLAVPSVVDDAPRMRAAHAGWPQWVGKR

>WP_024428630.1 autotransporter domain-containing protein [Burkholderia pseudomallei]

MTIGSKTKLALGIALAMPAAAWAYTFYALGDSLTDNGRVVRLTGILPNATSTIFRGGRSSNGPVWAEYLPGLIGARFAPDDDYAINGALSGHGGYLNIIPTRPTWRTLPGFVDQVEQLIAAHPRLRRDDLVGVWIGTNDQDLTKASLNGIEPFLGVPRPANIAEMSAYTLTNLNAQLQRLIGAGARQFVILNLNDAGGTRPGYIDYNGKLPEDLARFSRQGVNVHLFDVSALLNQMRRNPSAYGLNDTPDVQCRYVPSCSGGSIELQNTYLTADGTHMMTSVHEYIARYLANQLNAPAAISTAPGLGLDVARAGALSALNAADGGPLGPARVAWSDRLSLFADVGYTRNFHGAAGGMNAFDSDVEMFSIGADYKLSEASRAGALLSSGNANGSLAGGQGRIGLHAYRLGVYHAFERASLFVRAYAGAGWSRYRLDRAAVLPGAVRASTSGFDFGALVKAGYLFALGGVRLGPVADVGYTQLVARGYTEDGDPILAQNVGVQRLKGVSAGAGVRFAAPLAAIGLRAGELSAEAQLRHDAFGDRTLVTAQRYAPGLPIGTAVDGASATYERLSVALTVNPAKRWRGKLVVQTDLGTAQRRSYTLLALLGGTF

>WP_024428767.1 ABC transporter substrate-binding protein [Burkholderia pseudomallei]

MKKLALCAALALAAGGAFAKEWKTVRIGVDASYPPFESTAPSGEIVGFDVDLAKEVCKRINAKCTWTPQDLDGIIPALKAKKFDVIVSSLTVTDKRREQIDFSDKLYDAPARMIAKAGSPLAPSVESLKGKRVGVEQGSTQETFAKAHWEPKGVTIVPYQNQDQVYADLGSGRLDATLQDELQADYGFLRTPRGKGFAWAGTAVKDPRTLGDGTAMGLRKEDADLKDAINRALASMHKDGTYDRLSRKYFPYSVYSAK

>WP_024428782.1 autotransporter domain-containing protein [Burkholderia pseudomallei]

MLMTRHKKRKTMKRSGAKLLAPVVVAAAAAVAARPGWAQAAPYPDPGRRGDPASWRTPEFTNAWGLGAMHAEYAYAAGYTGANVAIGVLDSGYYAQHPELPDSRFVPVTAAGVSGVLNPNNNNHGTLVSGVVGGARDGVGMHGVAPDATVYEGNTNAIDGFRFGVSDPKFPASDAKYFAEAYDALAAKGVRIISNSWGSQPANENYSTLNKLTDAYKLHEAVRTATGRGTWLDAAAKVSRDGVINNFSSGNTGYDNASLRGAYAYFHPELEGHWMTTTGYDQLSGQVYNQCGIAKWWCVMAPTGVPSTSYSGGAAAPTGATYANFNGTSAAAPHASAALALIMERFPYMTSEQALSVLFTTAQNMEPDPSRPDYTNNGLFSTVHPAKPGASGVPNAFGGWGLVDLRRAMNGPGQLLGTFDAALPAGTADVWSNDISDVALAARKREDDAEHRAWLDTLRTKGWEHGLPAGASDGDRIDYALGVARETAYQAREYQGSLVKSGGGTLTLAGANTYRGPTTVDGGELRIDGSIAARAVVNPAGRLTVNGRAADIAVNGGVATIAGTSANLSIDRQGRAAVTGTTADVRVASGFASLGGTSGNVAVGALGVAAITGRTADVAVDGGRASLDGASGNVAVGNGGVVSGSGTVRTLTAAANGTVAPGHSVGTLTVSGDVRFAPGSIYAVEVSPGGAGDRIVAGGRAQIDGGALALALENTPPPLTPEQSRSVLGRRFEILNAAGGVAGRFDAPSGYLFVNPVLAYGPTTVSLTIDRNATPFASVARTANERGVADALETADPGSAVYNSVLFAASAQAPQAALAQLTGEIYPAAYAALVNESRQVRETALERLWTARGAPGRAGAWARLLGAWGSARGGDVNGYTSSTGGFLAGADAALLDGVRAGGFAGYSHTGVNLRNQPSSASFDSFHLGAYAGWQPGALGVRIGAAHAWHRGGVDRAVQYGAVAENETTALHAETTQVFGEAGYRFALDGAATLEPFFGVAYVHLKNQGTTETGGAAALRVRQGNHDVTFSTLGVRGETRLGLTSRLQLTLQGSAGWQHALTDGQPSGTLAFATGSDTFTVSSVPVAKDAAVLNVGAGLELGKNGWLRVGYSGSLASRQSEHAVQGSLHWKF

>WP_024428812.1 AzlC family ABC transporter permease [Burkholderia pseudomallei]

MNSLPTSTVISEASPARSEAWRGLRASLPVMLGFVPFALVLGAQAAQKGLGIVEVPLLTGLNFGGGSEFTAIRLWTSPPHILLIVAMSFLVNCRHILVGAAFAPYLRHLSRRQTFPALFFMCDESWAMALADARREGRGRVSLPYYLGVAAGLYLTWISCTALGAALGPTIGDVERYGFDMAFTAVFLVLLRGMWKGARASRPWLVSLVVAAATYLLVPGAWYVAAGALAGLVAAVVCGEPA

>WP_024428855.1 MFS transporter [Burkholderia pseudomallei]

MKTILTRDFLALILSVAVVGLGTGATLPLTALALTEAGHGTRIVGILTAAQAGGGLAVVPFVTAITKRLGARQVIVASVVVLAAATALMQFTSNLVVWGVLRVVCGAALMLLFTIGEAWVNQLADDATRGRVVAIYATNFTLFQMAGPVLVSQIAGMTHVRFALSGALFLLALPSLASIRKTPIADEPHHDAHDRWTRVMPKMPALVVGTAFFALFDTLALSLLPIFAMARGVASEAAVLFAAILLFGDTAMQFPIGWLADKLGRERVHLGAGCVVLALLPLLPAVVTTPWLCWPLLFVLGAAAGSVYTLSLVACGERFRGSALVTASSLVSASWSAASFGGPLVAGALMEQFGGDALIGVLIVSAIAFVGAALWERRALPMQAARRGR

>WP_024428898.1 NAD-dependent epimerase/dehydratase family protein [Burkholderia pseudomallei]

MLIWKTALSLTWKAGLVSPASAPAAAAHVDRVVLTGATGFIGGAVLVSLVNAGLLDRVVCIVRACDRAHALARLRAAALRSGLAPYWAERLSEANVIAGELDGALADADAAHIALASHVIHCAGVASLADARIVNETNVGATLRFARRFAGSRRLQRFVHVGAAFACGLRARGTIREDDTPARGREIDFAPYTRGKRDAEAQLRALGLPLVVVRPSCVVGHTLLGTQPSASTFWMFRIVHAARRFTARPMARIDVIAVDDCARALMLLALKPSLAHDTYHVSAGDEAPTVTQIVRAMDEAVGLDDEPRYALCSPAEFPSIARDVLGRRDAPRERVIRRALQSYAAFAELDHVFDNARVRREIDFEPLPFVDYVNECMRTSRGIDVLAQMPRTAAR

>WP_024428916.1 Gfo/Idh/MocA family oxidoreductase [Burkholderia pseudomallei]

MSTHPLPVVVAGSRFGQFYAAGLAASGAYRIAGILGQGSARTAALARRVGAPVFTDPGALPDDVRIACVAVGGAARGAQGAALARGLLERGIDVLIEHPLLPAEWDAVLRTAVRHGRRCLLNSFYPHLPAVARFVDVAQRLSRIGRPLHVDLACSVQASYAALDVLASALRGVGPWSIEPARQASSPMRECTMVLAQTPVALRVHNEMAAAADGRMHLLFRIALMTAHGTWTLASPHGPLAWEPALREPPADDDGLFPIFGAAAPPHGLPSMQLYEAEPPAWDAIHARHWPRAAVRAVDRLASGDGLAASNQRSVEVTRVWQHLTAQLGFPEPPPGDASADTLEPLLERAA

>WP_024428940.1 branched-chain amino acid ABC transporter substrate-binding protein [Burkholderia pseudomallei]

MSSYWMRFAAAASAALTLALPMPFAAAAATGEPIRIALVEGMSGPFANAGAAVERNLRFGIERVNAQGGVRLRDGAHPLELVVLDSKGSVEEALVQLRAATDKGIGFAAQGNGSAVAAALVAALDKHNARDPEHRALFLNYSADDPALTGRDCSFWHFRFDAHAGMRMDALADVLARDRAVKKVYLLNQDYSFGHDVSALARAALKARRPDIAIVGDEFHPIGRVKDFSPYAAKIRASGADAVVTGNWGNDLTLLVRATREQGLAAKFYTFYGNSLDAPAALGDAGVKRVLAVADWHPNAGGARSDAFYRAFRARFPAAQDDYPVRRMSEMIEMLAAAMTRAGSADPVAVAKALEGMRYDDGFHPAQMRAADHQLIQPLYVIEMERAGAPGVRFDNAGSGYGFATVLEAPPTQGAAPAECRMKRP

>WP_024429081.1 protein-disulfide reductase DsbD [Burkholderia pseudomallei]

MFNRIPRHAQSRFCFLIAVVAMLGVLFGTSLAARAADDFLDPAVAFKFSASEAPGQVDVHFKIADGYYMYRERFAFAVKSGSATLGEPQLPAGHVKFDPTFQKNVETYRGDLTIHLPIKQASGPFELAVTSQGCADEGICYPPAEHVARIEGAALGAAGTAPAAAGAGADTSAADGGSWYERVTSADYARSLLEGHGFLTIVALYFVAGMVLSLLPCSYPMIPILSAIIVGEGARATRARAFALSLTYVIGMALVYTALGVAAALVGQSLGAWLQNPWVLGAFALLLTVFALLLIGGVDITLPQRWQNGAAQTSGPRKGGRFAAVATMGALSALVVGACMTAPLFAVLAFIAHTGNALFGGAALFSMGIGLGVPLLVIGIGAGTLLPRAGAWMDGVKVFFGVLLLAAALWIVWPVLNAASQLGLGALWLLIAAAALGLFTPHSGSSSVWRRLGRGLGAALAIWAATLLVGLAAGSTDPLRPLAVLAARAAPSNGAAGAGAGAHEGPAFAPVRSIAELDEIVKTSTRPVMLDFYADWCVSCKEMEHLTFTDARVGARLSQMHLVRADVTANSPDDQALLKRFGLFGPPGIIVFDANGQERGRVVGYQSADRFLRSLDRMSLPAAWSAS

>WP_024429096.1 MBL fold metallo-hydrolase [Burkholderia pseudomallei]

MILRQFLHCDPVGISYLLGCGGKSTGVVVDAVAEPGTYLQAAADAGMKIDYVIDTHVHADHLSTGPALADAAGAAYVLSAKADVVLPFKGVEDGDEIRVGNVVIKVLETPGHTPEHISLLVTDRTRAEEPWLALTGHTLMIGDLGRTELAESAEAGARNLFRSVRTLKSLPDYLPLLPGAFAGSACGRSLSATPLSTIGFEKRHNAAFGIDDEAAFVRFMLSDMPPAPPEAARLRAANSGRNPDEI

>WP_024430699.1 membrane protein [Burkholderia pseudomallei]

MNVSQTCLLITALMPFLWTMCAKSSSRYDNHDPRGYLARLDGWRARAFAAHQNSWEAFALFTAALVVAWHNGANMQRVDQLAIVFVASRVLYGVLYLLNWATLRSLVWTVGLVCVVWLFFAAP

>WP_028359473.1 Fe(3+) ABC transporter substrate-binding protein [Burkholderia pseudomallei]

MFVKQARPLLRTLLRTLAFAFALAAVAPAAHAANEVNLYTTREPKLIQPLVDAFTKQSGIAVNTVFVKDGLLERVKAEGARSPADVLMTVDIGNLLDLVDGGLTQPVRSASLDGAIPANLRGAGGNWYALSLRDRVLYVDKNMQLDAITYESLADPKWKGKVCVRSGQHPYNTALVAAMIAHDGEAAAEQWLRGVKANLARKATGGDRDVARDILGGICDIGLANAYYAGHMKHAQPGTDARKWGDAIKVVRPTFANAKSGGTHVNVSGAAVAKHAPNRDNAVKLLEYLASPPAQALYAQANYEYPVRAGVALDPVIAGFGPLKVDPLPLVEIAKYRKRASQLVDKVGFDN

>WP_029671417.1 multicopper oxidase domain-containing protein [Burkholderia pseudomallei]

MNPAVDPSPLSRAAQALALASIGGALALGACTSRPSGERYAPSRMDFNPGPAIVTQTRHTGPFASGSALSFDAALQPLDAARDQAIRLDTTHTVIRIAPGIAFAAWTFGNQVPGPTVHVKVGDRVRLSMTNRSDEPAPGGLQLTAPMMHSMDFHAAMVSPTDKYRSIAPGQTMHFEFTPNYPGVFMYRCGTPMVLEHIASGMYGVVVVAPRDGYPTRADREYVIVQSEFYTKPDPQHRSVGTDALHVLDGERLRRKAPTYTVFNGRYNGMVTQPLIAKPGERVRLYVLNAGPSDTSSFHVVGAIFDRVWLDGNPDNQLRGMQTVLLGSSGSAIAAFVVPEAGAYVMVDHQFANASQGAVGVIDAGAHEESTIEHHNIPASATPTDAEAIQGKLDFESKCLACHTLGHGAKLGPALLGVTQRRSDAWLRRWLASPEAMVASDADARALRAHYPITMPDQNLSDSEIRRYVRYFHWADEASKQRDHAMP

>KGC51336.1 L-xylulose reductase [Burkholderia pseudomallei]

MTGASSGIGRAAAVALRGCGARVVAAARNARELERLAHETGCEPLELDVGCDASVRAALSGERMRDAFDGLINCAGVTSLAAAIDTTADEFDRVMAVNARGAMLVARHVARAMIRAGRGGSIVNVSSQAALVALPSHLAYCASKAALDAMTRVLCVELGPHGIRVNSVNPTVTLTPMAERAWSDPHASGPMLAAIPLGRFARVADVVAPILFLSSDAAAMVSGVALPVDGGYTAR

>KGC89431.1 spore Coat Protein U domain protein [Burkholderia pseudomallei]

MRIAATLALGLFSALHAHATCSVVSAAAASFGTVTSFAVARQPQSTSTTSSGLSCSGALLGLFVIGDQINASITSANGGKLVGPTGDAVPYTVFADQNYSIKLDLGVTYNWASGQLLNLLGIFGGPAQTLPMYFRTVQGSNVAAGTYTDTLTIAWNWDYCSGIGVLGICLGRDRGSGTAVVPVTITVTNDCMIAAPDVNFGAAPTVASFAPVTGSVSLTCTKGMVYTVGLSSGANPHASGRRQMANGANRLQYDIFGPGAAAVWGQSANRAGSGAAADGRSAQQFPYTARLYPDQPTPAVGTYTDSVIVDVRY

>KGD30356.1 hypothetical protein DO70_5810 [Burkholderia pseudomallei]

MIAFLARRAARRTPTPTTASHSPTSRSRGAGRHLFAALAVALIGLPHAHAEIRCGWLQNPTPGNWWLDDRAGSWTLGTMGGPETEGMDVIPDMAGKQYVETNGSHGYACACLTVVTDKQERRIVKVLKAKQLPLSRCRRDKRLKEPT

>KGD44734.1 hypothetical protein DP43_550 [Burkholderia pseudomallei]

MMKFLTREIVVLAALGWALLAGCAPVQAPPSSSSAPPASSCCGPITASGQTLRHTLDASDVEHLWLPRQHVDWSTGKPDPTAEGFKSHCSAFAAAMGARLDVYMLRPPEHSQILLANAQAAWLASDSGRAAGWRELHEAYEAQAAANRGELVVAAFQSADPKMPGHMAIIRPSLKSNVQLADEGPEIIQAGAVNRLDWNVRDGFARHPGAWPNGIKYFAHVVPAK

>KGU91512.1 bacterial regulatory helix-turn-helix, lysR family protein [Burkholderia pseudomallei MSHR4032]

MAAIRTFLVACRAGSFTAAADELCVTHSAVSRQIQTLESWLGTSLFEKDGQRMVPSVHARAFAQELGGALDALTDVVQRYGKGSARQALRVSVPTTFGMRWLIPRLAAFRDVHPDATIQVLTVTTQQQPSGGNCDVAIRRDDQVFNPESAIRFLSDHHTVVASPSLLSRTPLERPEDLVRHTILETETRPRHWDDWFAEAKLSASRFTRRQRFDHFHVTFQGIVDDLGVGIGPVVTLSRDLANGKIVAPFSDIRIAPHNYYAVTPIGIQKTALHREFEDWLVATAAAAETRAA

>KGV04506.1 beta-lactamase Toho-1 [Burkholderia pseudomallei MSHR4377]

MRRSLLVAAISTPLIGACAPLRGQAKNVAAAERQLRELESTFDGRLGFVALDTATGARIAHRGDERFPFCSTSKMMLCAAVLARSAGEPALLQRRIAYAKGDLIRYSPITEQHVGAGMSVAELCAATLQYSDNTAANLLIALLGGPQTVTAYARSIGDATFRLDRREPELNTALPGDERDTTTPAAMAASVHRLLVGDALGAAQRAQLNAWMLGNKTGDARIRAGVPADWRVADKTGTGDYGTANDIGVAYPPNRAPIVFIVYTTMRNPNAQARDDVIASATRIAARAFA

>KGV09504.1 lysine-arginine-ornithine-binding periplasmic family protein [Burkholderia pseudomallei MSHR4503]

MVTATALAAGSAFAADLKEIRFGVEASYAPFEYKTPDGKLAGFDIDIGNAVCAKLKVKCVWVENAFDGLIPALQARKFDAINSDMTITEQRRKAIDFTDPIYTIPNQLIAKKGSGLLPTTASLKGKRIGVLQGTIQEAYAKKRWAPAGVEVVPYQTQDLAYEDLKSGRLDATFQDSEAGAKGFLSKPQGAGFAFAGDHVSDAEILGTGVGFGMRKNDAQLKSAVNQALKELKADGTIDGLAKKYFSVPVTLK

>KGV62168.1 traB family protein [Burkholderia pseudomallei ABCPW 91]

MPEAVAAREAARRSRNGVRRAATHAARAPRRWRARSLASAVLAGACATGGLAWPPASALAAGSVAAAPLPQAPIPAPGMSLPGFHAPPPSTSNGTVASGAVRTQPARMPFYVATKGKVTIYVLGTLHVGDPADYPANQPFRRPILAALAASPTLALELSPDDLLESQDDVSKYGVCNYACLPRLLPPPLWQKLANRLRGNPAALAGIRNMRPWLASLVVETYDSLSAGLQTEYGTEAQLQNVFLRKKGGKVVGLETLAEQMRAFTGLTLAQQREMLAQDMVQTPAQNAADVRALHRLWRIGDADAIAAWANAKTERLARARSIADSIDNKIVYERNRRFVARMTAIAAPNRPLFVAIGSLHLGGPKGVLELLRQQGYRVDAG

>KGX12970.1 hypothetical protein X984_4408 [Burkholderia pseudomallei]

MLLLRHSILTALTATACASAHAQTADDAPVAAPACAIAIVTGVGGAATNLREYLAARERDRYRYLADHPLDCRVSEDGRASGCTGLAYLRRERVSVYDDGDDTVQNVVARVDLDHGTYPAIIAVQKRDVRCEQ

>KGX72041.1 hypothetical protein Y026_6279 [Burkholderia pseudomallei TSV28]

MNHKKGDVARFENMQKGINMKRSRSRFRASFTVAVALGVPSGFAHAQSLPGIPDPAAVGLTPGTGQNGAPTVSACFYNYRTKVIAGFPTQIASGWIVTNQRNDPACIPPGGLPGPNWQEMTYFRDMPVGASMSACWSFSWPSNWQPVGYTSDPSKCGYYPGGQIPGAQPNVVFLKRVQ

>WP_038707916.1 Tat pathway signal protein [Burkholderia pseudomallei]

MDRRKFLSTSGMVIGAGAVQIVVPARTVYAATAASQLSNISSVAASSKLPNFANTKAILFPGSDKTVEALLDNAKSKIKSGTFDGSLLATSIEKQNPITGLYVILETAAITVTESIPVVGWLISGFLSIGLKYLNESLKEKTDYEKLIKSIVNKSIDENNYRLMKAAFEGVTQVYGNFSDLAASLSTNKPKPDQKTQLISLFTNTISNCEQNAPTMLLKNLNDKAAYQGIPLFCSIANVELSAHADMLKNKVAFDLDDSFYKNNYAAMEKKAWRMHKKAREVVVELLDNVGDLSLDYPSVWAKKNSIIRQIYLSGLGAFLENYRRRYEAKFLKKIKLRNSIEIYSNSYADQGVIEKTINADITRRSSLLSRLSGYSHIDRVQRIGVGYLSSSENSKGFFKEYESCTGRVNDRRDEKDIDSKFPIDQFANSSRTGRELPVKIYSNAPKAGTYGLKIITNDGKNIDFGSMNGDLKKIYSFDVPGYYISGLVVAESNAKRGSCAADSGLSIDFICSVHRHCSSIDYIAGFTDANIGIELDNSVWSFENNSSSNVTGYADYLVGKNILSVPSGSVKFDLYNTTGKAISVTPVLMAAIPGRKDGESASLKVSIGNSVAQVKLSDKDSDKEDSYIQGIDGEKYLDAASGEKITVPVGRSSISIENVDKKAGFKFASLLLIPG

>WP_038708181.1 collagenase [Burkholderia pseudomallei]

MTEVFRKTRRWSAVAALSAFVGLAGAASANTQPMQPTQQKQARMPRLPQNLPVSPEQAEYNLPLSEQDRAALTRPSPLKQPAKRGKRSAPGADCRDMSVMTQYRGAALADYIANLPDYECHYGLFSVDKTLAAQIFSAENVHAVASRFVQDIYRYDASNLILVNLLIYLRSAYYQYDVSGIANPIPNLAVWLRPYIKQSLEGAALYRENARAPSTANELMKLITNMKDEAFYLPTLKARIAFYTASATNPQAAAPLLQPSAAGGFTGLLTVFFYAHQRSGAQPMLDSDATLPETLNRFVTANRASLSNTSAAYQLADAARETFRFLRYPAQKPRVKKMIQDMLASTSMTGADSDLWLAAAEAVDYGDPGNCADYGTCDYKKRLTDAVLTHRYACNAGVRILAQDMTLPQLQSVCTSVAQQDDYFHRMMKTGRKPVAGDRNDTIELVIFDDYANYRKYASVIYGISTDNGGMYLEGDPSAPGNQARFIAHEASWLRPEFKVWNLEHEFTHYLDGRYDMAGDFAASTAKPTVWWIEGLAEYLSRKNDNQESIDAARTGAYRFSDVLGTLYSSSDYVARAYRWGYMATRFMFERHRADVDTIVSRFRVGDYDGYANYVAYIGNRYDGEFVDWARAATTAGEPPLPTKR

>WP_038714058.1 spore coat protein U domain-containing protein [Burkholderia pseudomallei]

MHKREVQVVSGAAFMLFFAAVSAGQLTGTMQVNLQVSRGCEVAGVAASGDLGRLDFGAQGPLWSDYLTADGRATSSGAVRVVCSPDVNGFLVSIDGGRNGDQSTRYLVKRGANGRVAGRIPYNVYRDAARSVPYVPLMPQSFFVDGGRDDVTLPVYGVVNGMTRAVPSGTYEDLLGITLDW

>WP_038724299.1 sugar ABC transporter substrate-binding protein [Burkholderia pseudomallei]

MRLCTGKAVLRACVAAIAVAAGVGSAAPAAQAAGARFALVSHAPDSDSWWNTIKNAIKQADEDFDVTTDYRNPPNGDIADMARLIEQSAARDYDGVITTIADYDVLKNSLRKVTAKKIPLVTINSGTEEQSAQLGAIMHVGQPEYVAGHAAGEKAKAAGVKRFLCVNHIATNSVSFDRCRGFADAIGADYKSSTIDSGQDPTEIQSKVSAYLRNHPNTQAILTLGPVPAAASLKAVQQMGLANKLFFATFDFSDDIAKAIQSGAIKFAIDQQPYLQGYIPVAVLAIAKQNKTTDPAKIRQILEANPKFQARLSTYGLQPSYGPKNIRSGPGFITKENLEKVIKYAGQYR

>WP_038726191.1 hypothetical protein [Burkholderia pseudomallei]

MKRTIRKSRAALVPLLVAGSVCAAAPLCQTQKLGAHTSKMCVEQTPFKHDYYTLWVDDSPIFMLPDDYVEKVALTHTVPEDGAIEFPLSKQGTPTVTISGGCAPVSETQGKGADAVNLETGRVCSFNWGKEPVVKDLRFSFE

>WP_038727163.1 hypothetical protein [Burkholderia pseudomallei]

MKSSVLFMAALPLVAIAASAHAQPRHPAVYSPAAGVLCDRYVCADDQGISRALTERYLGKRVAAKAFSQGDFDPTEFTFANGVFCDVKERLCRDDRYYGADGKRSGAVSRRYTELLFGRRSGG

>WP_038729084.1 hypothetical protein [Burkholderia pseudomallei]

MRNRKFVSFVVALAFAAAAATPALAVTVVRVDGQPMNPNGEPFSATSAPLETTLSKGSISANCVATFNGTITPAGIVNITSTTFTGTNSLCGLIKGSASGTNPWTGQADSATQLTINNAQVNVTLLGQCGPSKVVTSWTDANSSLTFSNAALAPDCKVTGTVVTSPKFHVQ

>WP_038730428.1 peptidase [Burkholderia pseudomallei]

MQTSRKALPLALGLAIGLGAALPAWADSKAPSPQEESRRASLTRGVVAPAEQAGKTGQFRPGAVAVTLASPAFHAKKADAAAMAREYVTARAAQLGLDKAALANLVVASERADAAFTVVRFQQRAAGLPVYDSDIAVTVAPDGRVLYVASKAVSGVAAVSSKTQAVDEQQALDRARAYLGVGGFVNVQSQLVAFVDGAGTHTAWKVSGRPQDSLHGDWELIIDAGSGEVLRAQDKASYATDGSGLVFRPDPLSPTKSSYGSPGFKDNNDADSPQLSAARVRVTLKDLTQTSGGYKLSGPYASCIDFDAPLDKACPVQASTTFDFTRSNLYFEAVNAYYHIDTFLRYVNLTLGVKALPYQYAGGVQYDPHGQSGDDNSSYSPSSGRLSFGQGGVDDAEDADVVIHELGHGIHDWITNGGLSQVEGLSEGTGDYLAAAYSRDFNQWSPSDAQYHWVFNWDGHNEFWAGRVTNYNVGRTYAQIRNAAIHTAGQYWASCNMVARDAIGGAAMDKAFLKGLSMTNGSTNQKAAAQAVLTAAAALGYSSAQLNAIGDAYNKSCTYGVTVPQKL

>WP_038730764.1 cytochrome c4 [Burkholderia pseudomallei]

MNRLSKSLVVLEFAVAGLAGFMAEARAADAAKPDLDRGKAIATQVCAACHGVDGNSATGSFPKLAGQHPDYLVKQLHDFKTQPGAKGPVRVNSVMVGFASALNDQDARNVAAYYGSQSAKPGAARNAATVPVGQKIYRGGIAEKGVPACASCHGPTGQGIPVQYPRLSGQWADYTVAQLTAFQQGTRSNDAMHQIALRLTDSEVKAVADYIAGLH

>WP_038740236.1 hypothetical protein [Burkholderia pseudomallei]

MISFKRKMFASPASRPARAGKFVLEACFAGALAGAAHAQGAPQPVVDWEIQVVRDGQTIDAFEQKTTVGQARSDTHSLPIAPAAGCAATSAAASAADAPGSGLSRTITVAPLYVEDGAVALAIDAQETLADEGATPPAGAPCTFALPRRIVASHPGLNARAAQWTDWTLAERNPQLVYRVRARVVED

>WP_038741088.1 hypothetical protein [Burkholderia pseudomallei]

MTTRARVSILPRMLVGAACAAAALGAHAQNNLNFLNDTPISYFSKADTASLAKAVQKVRDEGKDGETVDWVNDGRGTKLAAKLTPSTTEQEGRTCREIKTEIEAKGQSMTLRPLYCKTAAGKWQLQKR

>WP_038741497.1 triacylglycerol lipase [Burkholderia pseudomallei]

MARTMRSRAVAAAVAFAMSAAPAAGIGTFLSLAGTQSAAAATSAVDNYAATQYPIILVHGLTGTDKYANVLEYWYGIREDLQAHGAQVYVANLSGFQSDDGPNGRGEQLLAYVQQVLAATGASKVNLIGHSQGGLTSRYVAAVAPQLVASVTTIGTPHRGSEFADFVQTVLAYDPTGLSSTVIAAFVNVFGILTSSTNNTNQDALAALKTLTTSQAATYNQNYPSAGLGAPGSCQTGAQTETVGGNTHLLYSWGGTAIQPTLSILGVTGATDTSTIPVVDPANVLDASTLAMLGSGTVMINRGSGANDGLVSRCSSLYGQVLGTSYKWNHLDEINQLLGVRGAYAEDPVAVIRTHANRLKTQGV

>WP_038749854.1 TonB-dependent siderophore receptor [Burkholderia pseudomallei]

MKSRSDELKLGKFTTLCSVLAASPAFAQDAAPPAASADHDKELAPIQVKGAAEHSYKADFSSSVKFTAPLVDTPKSVTVIPQELIHNSGAATLTEALRTVPGITFGAGEGGNPLGDRPFIRGYDTQGSMFVDGMRDTGATTREIFNTERIEITKGSDGAYGGRGGAGGSINLVTKAPHLGTTAEASAGLGTDRYRRFTADGNWQFADHAAFRLNLMSHNNDVAGRDAVNNERWGVAPSIAFGLGTPTRVSASYYHLSTDDLPDGGIPYFYTTANKPANVDTIYPANVDRHNFYGLVNRDFRKTTSDIGTLRIEHDITPSLTVRNTTRYTESTQDYIWTQPDDSQGNVVNGRVWRRNNNRNSAINSIANQTELFGEFRTGPFKHSFTTGIELSREWGKRDSYSVATGTGKICQQGIGAASGYNCTSLGSPNPNDPWAGSITRNNDYAHARTVTKSLYGFDTVELSKHWQVNAGVRVDDYSTRFTDTRANGGKTYTRDDTLVNWQLGLVFKPARNGSVYASYATSSTPAGALLGEGSETQSLTPGRGGVGANADQLAPEKNRSIELGTKWNVLDDKLSLTAALFQIDTTNARVTLPNNQYAMVGNKRVQGLELGFAGQLTRAWQVFGGYTYMKSELRDNGRNTADNGHQFPNTPKHSFTLWTNYDVTPKFTVGGGAFYMSKVFGDTANLRAVPSYWRFDAMAQYRINKKLDLQLNVNNLFNRTYFDQAYPAHYASIAPGRSAFVTLNARY

>WP_038750526.1 SCO family protein [Burkholderia pseudomallei]

MSADEIRTRRRKLLAAALSAALAPALPALAATQRAQPLAGQSGYHGGLITPPVPVPDMPLRTADGRATRLRALLAGRVTALQLFYTGCSSTCPIQGAVFHRVQTLLGPHPKPDIQLLSLSISPLEDTPQRMHAWLARFGARPGWIAAAPELKDVDALQAFFGGGRTGLDNHGTQVQMIDRRGALVWRTYELPSPETIAALLGHA

>WP_038760383.1 fimbrial protein [Burkholderia pseudomallei]

MKKKFSRLLAALVLLAATVDALAAGCNMLTAENIAWLTEQGKIARAHSSFPISFSSGMVDVDPNLEIGGLIAEAKSIPSEELHFIWCSAPSGNVHFALNSSPLPSELGNSIYETGVPGVGFRITQVRQSGSIGAIPRDTPWIEEKPGQDSSLNFGAGTVFRIELIKTSEALPSESTISLGNLSRVYGDDNKTVVDFNAGSVKLRVLPICHVDQQEKNVDFGQFGPKDVSFDSGPTKDVKFDVQCSGPTPPVSITATLAATPDSHDQSLIANAGDAMNLAIRLRDASTQQVLRPNDPSSEIKVEPGGAMEHGFALEATVLRVGTAPPTAGTIDATSIITLTIL

>WP_038761248.1 membrane protein [Burkholderia pseudomallei]

MKRSLIAFALTGAGIALSSTAHAVNVDVNIGTPAPVVVAPAPAPVIVAPAPAIVVGWHGDRYWDGHRYWERREWEEHHRHRGFCPPGHAKKGEC

>WP_038762696.1 hypothetical protein [Burkholderia pseudomallei]

MTTIKAGDASGAGCAAAARARMTTRIMRRALAVLGGAAFAGWFGAAFAASSGTTVPPLDTTPWLASVQAPKTPFFTRASDAQGVSFHFANSGHDVERGREVVVSETADGDMWQGGMVHATYIDAAGVETSGWLVRSHLRRVKKPVPPPPTWDGRWRSSAGARRLIVHGERISYSFAGGGGTGGAQPRVEMLLRMRPVSDDEAVLSRMQAPDGGMCDLAVRRLGDYLIVSARDCFIPGVSPEGILRKQR

>WP_038765499.1 type 1 fimbrial protein [Burkholderia pseudomallei]

MKKKVVSFAVATLFGLVAAQSAFAQSIDQGQVKFTGELTANTCTIDAGSKDQIVPLPKVSTSALAAPGDVAGSTAFEIKVSKCAADVKKVAAHFEMENMDPNTRTLKNEATGADAATNVTIQLVNSDGTELPVGSTGSYFDVTGTDDARGATMIYGGQYYALDTTTAGKVESHTLFTLAYE

>WP_038774138.1 MmgE/PrpD family protein [Burkholderia pseudomallei]

MNRRHFLAAAVAAGLPLAATLAPRGMRAQPNATQAAAPTLARQLAEYAAGLRYEDLDSATIDIVKSHLIDALGCALAALDEPPVRIARDAARSAGGGGPSTIIGTAERTSPDLATFATGTALRYFDFNDAYAGREIGHPSDNIAACVAVAEAQHASGRELILSIALAYEIACRLMDAAAISPRGWDHTCYSLPAAALAAGKLMRMPVEALTQAVNLSLNSHLALNQTRVQQLSNWKALADADAARNAVFSTQLARAGLTGPSPIFEGEAGFFRQVSGPFELETSRFGGRGEPFRIARCFVKYYPAQGFTQTAIPAALDVASQAGDLSRIRRIDVHTTRVGYVTAGSEPEKWKPSTRETADHSLPYVVARAMLDGDIRTTSFSDAALRDPVLHALIAKIRVEEDPALTAGYPARAANRVTAHCRDGAVYAKQVDDLPGSPTRPMRREDFEAKFVKNGGARLSEQRMRAALDRLWRLDELQDVAALPPLFVAG

>WP_038775093.1 triacylglycerol lipase [Burkholderia pseudomallei]

MKSRFASAAAAVRGARKTLAKVWAAVLVAAMCTFAAPAAHADAATGALDDYAATRYPIILVHGLTGTDDYFGVVPYWYGMRPDLQQHGATVFVADLSGFQSDIGPNGRGEQLLAYVQQVLAVTGAQKVNLIGHSQGGLTSRYVAAVAPELVASVTTIATPHRGSPFADFVLGALSLDPTGLSTPIFGALLNVFGILTSHTHNTNQDAIAALNALSTPYATQFDAQFPSAGLGAPGACASGAPSETVGGNVHLLYSWSGSAYQPITLLGLTTGALDKSTIPLVDPANVLDPSTLVFLTAGNIMALKQAGLNDGFTSTCSSLYGTVISTDYKWNHFDEINQLLGIRGAYAADPVAVLRTHANRLKLQGV

>WP_038778478.1 membrane protein [Burkholderia pseudomallei]

MKKYALALWLMLVSSMSAMAGAIIPSGATLVAGQYAISNNGNFAFGVDAASGKLYFHYRFPTQAWGASAMSQMYGTQPEGALYVGKGDRLVMQTDGRLVFYSGNDVVWSNWYYGPAPIPGSYAVVEDFGVVAIYPPQGGYPTFTWPFASVTPNSGALATVVGYPFGVNFPVLNGRQWLQIFNNVTYYLWNKSFAADSVAMQTDGNLVVYNKGQPVWSTGTSGNPGAYMLVTPVGFVLGKQYGGILANVPYPVSVDQSSRASGPNDPKPTPAAPPPPSLNLPINASWKCYYVKDWLACYAPG

>WP_038781359.1 ExeM/NucH family extracellular endonuclease [Burkholderia pseudomallei]

MRTPTPLLAALSLLSTVAAAPAFAAAASPVSTNCGGSATPIAEIQGAGAPSPLAGQNVSIEAVVTADFGGADGFGGFFVQQADPQRRNQPGVSEGLFVYSPKARARAGELVHVTGKVEEKYGQTQLTLSGAVAVCASGQSVTPATLALPVDSPNAFAAYEGMLVRLPQTLTVTEVYELGRYGSVLLSNGRLRTPTSVVPPAQAKAVAEANARNRLILDDGSNKQNPATVPYPAPALSAANTLRAGYTAADVEGVLELRYGAWRLQPVPGARTPTFGASANPRTAAPARDPRANLRVASFNVLNYFNGDGAGGGFDDPSNRGAKNYDEFVRQDAKIVSALKALDADVIGLMEIENDGYGPLSAVRQLAAKLGENWRVVDPGSARLGGDAIAVALIYDSRKVKPIGNAATLAIDDKNRQPLAQTFRPLGGSRAVTVAVNHLKSKNCPDATGDDLDQGDGQGCWNATRSRAAAKIADWLARNPTGAPSEGVLLIGDLNSYTYEDPVRTLESRGYVNLVSSKIGSGAYSYVYNGEAGYLDHALATNALAWRVKAVHDWHINADEPIALQYTLAYKSAEQQRTYYAPDAYRSSDHDPVLIDIALADEYAAAASRRGAEMAAAHSRRPWH

>WP_038783934.1 hypothetical protein [Burkholderia pseudomallei]

MGDRGLRGGRRRTIAHDTAAGVALPSLAALPPGVLPADLAAASSGALLSVGGGPSQQIDDHAFTSRNYTALVCSSGLRQEFTTPHCSR

>WP_038790363.1 sugar ABC transporter substrate-binding protein [Burkholderia pseudomallei]

MTWINRGRALAGCVALAAALAAAPAGADTGKVGLDLPLLTSPFWQSYNRYLLHYAKDMQIDALAPVNSNGDPAQQITDMNTLLNLGAKGIVVGPLDSAAIGRALDAAAARNVPVVAVDVAPTQGKVAMVVRADNRAYGEKACQYLGEHVRRGKVVQIMGDLASVNGRDRSEAFRACMKGYPNLQVLEIPAAWKGDVAATALDSLLSANPDVKGIYLQAGGVYLSPTLQTLRRKQMLYPAGDAKHVAIVSNDGIPQEYEAIRRGDIDATVSQPADLYARYGLFYIKAALAGRTFKPGPTDHGSVIVQRAPGVLEDQLPAPLVTKANVDDKGLWGNTIK

>WP_038797268.1 PepSY domain-containing protein [Burkholderia pseudomallei]

MKTSRLIKAVALASLVLGGALGIRAAMADDGDDCRAPLADWKPRDAVRALAQQKGWRVDKLKADDGCYEIKGHDADGKRFKAKLDPVTLDVVRMKREGERKRDHDDDDDHGRAPDARAPAGGPPAGAPPGGVLKPGSKPDVQIR

>WP_041189005.1 c-type cytochrome [Burkholderia pseudomallei]

MESRVSSRRLFRPLLAVVLMSAAGLLSTAHAQTKPTEPAAAKAPLKAPDTMAERVRGCTACHGTHGQGTDNDYFPRLAGKPAEYLYNQLVNFRDGRRKYPPMNYLLTYLSDDYLREIAQHFSDERPPYPAPTKPTVPAAVVERGKQLALHGDPARKLPACVACHGNGLTGMQPAIPGLVGLHSDYLSAQIGAWRSGTRHAKAPDCMHDVASKLSDEDVTAVTAWLAAQPAPANPVPAPARSMKTPLACGSEPQ

>WP_041190807.1 TlpA family protein disulfide reductase [Burkholderia pseudomallei]

MKRVAFALAAFAVLANAAPGAAQEGRLPQPLRATDVASLYASGSGAPLVVEVWSLDCGYCRENAAHLVEWQRRHPQVRVALVALDSLDEHGQELAGALAQMKLPAAVRQYANAEPMPERLRAALDPRWRGELPRTLWIGEDGTRRAKSGLLAPAVLDAWLQRREP

>WP_043294325.1 hypothetical protein [Burkholderia pseudomallei]

MLKRTALFLALAGSIVAFSVAQANGDASLKPQQEIQLTKNAWGCLSKDNLDSVLNHERDGKAQAKQQYFDDYRCLSVPEGQRFRVVSVDKGDVQFVSAENSDQQGLWTDARFIKQ

>WP_043299325.1 hypothetical protein [Burkholderia pseudomallei]

MKYLPLIALIAATSASAADTGVQNVGQSQKSAQDVSACIAKTWADKSQQQVVTQNVLANGLAADVYVPGQQPPNGAAAVVRPAFSGNAKTWVGFRAGSSGAADGAAAGDINSCL

>WP_043304483.1 hypothetical protein [Burkholderia pseudomallei]

MTMHGEPNARRASLMGLALKLAFGFAFALAWPGHAARADDSAARGRALFRGDAPLQGRLPMHPGDLPAPLVRCANCHAAGAGAAVPNSIAPRLTRAWLGDLQSRRGGPPSRYDRNAFCALLRTGLDPTYVLINVEMPRYRIGERDCTALWRFLNEAPHERR

>KIX45952.1 hypothetical protein SZ28_00940 [Burkholderia pseudomallei]

MRRALPIPILFAVACVAARAAGLPMPVLSRLPPGYEVMAARQGPDVDAGRISYLIVLHRPADSASEPSPRPLVIVEQQADGTFRLAARNDEVVLRANEGGQCDPFDPQDADENGLAVKGRFFTVQNFVACGQHWSDYVTFRHDARTGRWLFANEIRTESFPLEGKPDRVRAIRADPRKPVALDAWRRGD

>WP_045588731.1 hypothetical protein [Burkholderia pseudomallei]

MSGITGSIPVAQAGAMSRGPVPLDVKRIDGKPAACIPTSDEGQAEMRLGFIGVSRATGPVSPDVIYWQVEVSDNVPPVYLKRGDCIVYGQSIPGATVLTPPKRFDIGKWYNFAVMPGGKAEGPIYRGTFCISGKQGDSGRVVLRSENKDACSSGR

>WP_045597613.1 amidase [Burkholderia pseudomallei]

MKRRQFLHTLGALGPTHALFASAPASAGQTALDERAGASAPASRAVRESLERIARIDRDGPRLNAIIELNPDAEAIAHALDAERAAGVARGPLHGVTVALKDNIATGDRMSTTAGSLALDGVRATRDAHLVARLRRAGAVIVAKANLSEWANFRSTRSTSGWSARGGLSRNPYALDRTTSGSSSGSAAAVAAGLVAVSVGTETDGSIVSPAAINGCVGLKPTLGRVSRDGIVPVSHTQDTAGPIARTVRDAARLLGALAGGDARDSATASAPAPADYVAALDANALRGARLGIARAYFTGHDEVDVQIERAIAEMKRLGAVVIDPVDLPKADYEEDEKVVLLHEFKHGLPSWLRTFAPHARVRTLADVIAFNARQHAREMPYFGQELLLHAQEAGGLDAAAYRDALARCGRRARDEGLARVLREHRLDALVAPTEGTAWLIDLINGDSGGDGFSTPAAVAGFPHLTVPAGLVRGLPVGVSFVGAPWSEARLLALGYAFEQATQWRREPRFVERSNVPAAR

>WP_045889382.1 hypothetical protein [Burkholderia pseudomallei]

MRANRNNGLTAGALPPNSAAPDDAAKAAPPVAQSDRGAGPLAGLTALKADRAQCMSARPSIRSTTKAPAVMSHARADTSRELTPVEARIAQTFVRPEHLRAIAAAARAGSFAVSFRASGQLSLKRLANGAPAKGHDILEKTIKPGSVQEVYGADSERRLAQIERADIDGYVGHWGRDKLLGLYMGPAANERDTPMLFAALRSTPSGHRYYPVDHDDLEGSLALLKRTQGPSGTAGSFWRSLPYTGDYDTHDMINMAGHRAPVPSGSRDEHRIMRSLNVAIEAIDPIRKASSGSHRLIQHGPQHNYVAHMRTNEQGAVIDEAVARASFPLAMCDRGTWSVIETPEALAAFYRQHGVVLKEKWREKRANTFTA

>CFK48476.1 oxidoreductase [Burkholderia pseudomallei]

MSERTTHDAARRTGASVGRGCGGGAPAGISPRMIRAHPFGYPKHLDRRRVAQAGCPKRRKAVRRLHPPLAQPATALCRVPGYGALVQLWRRVRATLPGVVCFEAMRPAFYRCVAAHAPARRRRSTRTAASRR

>CFL10512.1 Pectinacetylesterase [Burkholderia pseudomallei]

MLVRECLECLRRLCVAGLLSAAAADGHAAVAAAXXXXXXXACAPRTTDPIEMEESMPVRECLECLRRLCVAGLLSAAAADGHAAVAAAQDPSIPYYSWYEVTLPESSGASCGNGTPMRFYINRAQSDNLLYMMEPGGACWDHGTCTETATGAQAGLGAFNPDGIPHNYVNGTVQQSLKTSFLSPLMTRMDLAHILVGEPKVETQEWTQVFVPYCTGDIHMGSAVRSYASPSGDWRIQHYSGLKNVRVIAQWLVDHGFGKPSRLLVYGTSAGGYGTLGNYATLRGTLQPQSHSSLLDDAGTVFNTRFGADPAAYPSVGLYDKVRDEWGMTAPDGMITVNSRLTRYFDPGNMGSAYAALSATYPHDRFGYTTYRRDRIIAAYHYRPFVPAVIAAPDDATKDALSLAMFDRELSDLKQVLNPLPNFGYFIPWARDDFMGNHQVTAVSFTGSGIHENGIDADVGTFVADLLNQQDPADVPVMKAYRTEQWSDFTFSTFLAWLDSIFNLTGEAGPISGHRS

>CFK95952.1 extracellular ligand-binding receptor [Burkholderia pseudomallei]

MNIKMQKLLPISAAAMLLAAAATNAAADQVVKIGHVAPLTGGIAHLGKDNENGARLAVXXXXXXNRLLPISAAAMLLAAAATNAAADQVVKIGHVAPLTGGIAHLGKDNENGARLAVEEINAKGLTIGGQKVTLQLDAQDDAADPRTATQVAQKLVDDKVVAVVGHLNSGTSIPASKIYSDAGIVQISPSATNPAYTQQGFKTTYRVVATDAQQGPALANYARSKGVKSVAVVDDSTAYGQGLANEFEKKAKALGLKVVSHDATNDKAVDFRAILTKIKGENPDAVMYGGMDATGGPFAKQAKQLGLRAKIFAGDGVCTEKLADLAGDATDNVVCSEAGASLEKMPGGAAFKAKYEKRFGQPIQIYAPFTYDAVYIIVDAMKRANSTDPAKIVAAMPKTNYTGVIGTTTFDSKGDLQHGVISLYDYKGGKKTFLDEVKM

>WP_050040327.1 LysR family transcriptional regulator [Burkholderia pseudomallei]

MDRFKQIETFAAVVAKGSLSAAAHAEGVAPAIIGRRLDALEERLGVKLLVRTTRKLTLTFEGSAFLEDCQRIINDMQNAEASVSAGGVKASGHLRVSAPAGFGRRHVAPLVPPFTGAHPDVSVTLDLSDRMVDLVNEGFDCAVRLGELPDSSLVSLKLGENRRVCVASPAYLARAGMPGTLDDLARHNCLALAANANQQRGWSFVDGGKVVSIRVSGTMECSDGAVLHEWCLEGHGLAWRSWWEVGADIAAGRLVSVLDAFAAPPVGIHAVFPQRRHLPLRVRLFLDFLKHTYERPGYWG

>WP_053292838.1 M20/M25/M40 family metallo-hydrolase [Burkholderia pseudomallei]

MSKLQHLAAAVCGALCVSAARAAPVWITLSEPALRELRALDPAVTSRYSAALATGDAKRTETIHVAQVDDSLLESLSQAIRRTRGHGPGFFVHATFDEARASLQPSAAKQAAAIDYPITYSQQVRNWISQLQASNIVSTIVSLSGFTNRYYTTTHGVAASDWIAQQWKQLAGSRTDVTVEQFTHAGWPQKSVILTIKGSDPAAGVVVIGGHLDSTVGRMSENTRAPGADDDASGIASLTEALRVLLANRYQPKRTLKFIGYAAEEAGLLGSQAIAKQFRAQNVNVVGAFQLDMTNYKGDPKDIYLIGDYTNATQNTYLANLAKAYLPELAVGTSQCGYACSDHASWNAQGYPASFPFEADQNDNPYIHSAYDTLERSDSQGNHALKFSKLALAYAAELGGGLSASAKR

>WP_053293009.1 glycoside hydrolase family 2 protein [Burkholderia pseudomallei]

MKSAPDRVARGAAQWTLIATPAGAIARPSELGEAGWCAASVPGTVAQALAAARRFDPAHPYPLGDSDYWYRTTLHGAGPRIVRLNGLATIAEVWLDDTLLLCSDNMYVAHDLPVTLGGAHRLALCFRSLDRHLAEHPPRGRARWRTRLVDTPALRGVRATFLGRMPGWFPAIEPVGPWRPIDIVNPAGAPTIVHDTLRATLDGRDGVLDATLEFAAPLPRTARAQLVCGEHAAPLEATGPRTARATLRIANVTPWWPHTHGEPALYDVGVAIGGATIALAKTGFRTLAVERGADGRGFALSVNGTPLFARGACWTSADPVGLHADAPAYRRALALARDAGCNMIRVGGTMIYEADAFYALCDELGLLVWQDFMLANFDYPSNDPRFAESLKREAEQFLGRHMARPSIAVLCGGSEIAQQAAMVGLAPDERRVPATEQWLAELCAAHRPDAAYVSDSPHGGVLPFAPREGVTHYYGVGAYLRPPEDARRAGVRFASECLAFANVPCDATLASIGSPAAHEPAWKRAVPRDPGAPWDFDDVRDHYLRTLYGVEPARLRSIDPARYLTLSRAVVADLVGETLAEWRRVGSSCAGALVWQFQDVMPGAGWGLVDAHGRPKSAWHALRRVSQPRQILLTDEGLNGLDVHVLNDAPAPLEARIELVALRDGKTPIARAARTVHVAAHAGQCVNSADLLGRFFDFTYAYRFGPREHDVVIASLYASDGALLSQAFHFPERTAPTVFERGDIGLEASAAYRDGRWCVQVQTRTFARYVHVCAPGLLPDIDWFHLAPGAAARIEFAADPHSPAPDHRPPEADAAHCAPPAIEVRALNSNKTIRPRIEN

>WP_057050932.1 CoA transferase [Burkholderia pseudomallei]

MSGNVSKRLRASSGSEIRRAAAGASEAAGAAGTAGAFADAAHPLPPAGGLCMSSEGGVPPGTAIADATATASDARTAFAANTKDAKDAKDAKDAKDAKDAKDAKDAKDAKDAKDTKDTKDTKDTTSRPSLAAANHATGPVRPGALAGLKIIDLSRVLGGPYCTQALADHGARVIKIEPPAGDETRGWGPPFLDDDAWYFTGVNRNKEGIVLDLSRDEGRAILWRLLEDADVLVENFKPGTLARWGMDYERDLRPRFARLVHCAITGFGADGPLGGLPGYDAVIQAMAGLMSVNGDAGGDATRIGLPIVDMVTGLNALAGILLALAERERSGVGQSIDIALYDCGVSLLHPHLPNWFGSGRTPARSGNAHPNIAPYDSYRTASAPIFLAIGNDRQFARLCAHLGVPALASDPRFIDNRSRCAHRAELKASLETRLAGRDGETLARELMAEGVPCGPVRTIDALACDPHTLHRGLVVEMGRYRGAASPIKLSRTPATYRTAPPTLGRDTRAVLDALGIDAATQARLADAGVLKEAKPMAT

>WP_060547731.1 hypothetical protein [Burkholderia pseudomallei]

MRTANVACALAVGFASAAALADAPAMPSGGMLVAANGMTLYTFDKDAPNAGKSLCNGPCAANWPPYKASATDRPAGGYTIIKRDDGTLQWAYQGKPLYFFAKDAAKGDKKGDGFKDVWHAVKE

>WP_063597677.1 type 1 fimbrial protein [Burkholderia pseudomallei]

MKAKGMQYDLTAVLLSMLSGGAAYSQTATTGTINFTGSITDVPCEIDTAATSSNVTMAKVFANDFSGVGSTTGTTAFKIVLKNCGASTSGATVRFMGTTDSANPAALQTTAGGAGGVALQLVDDTGTPISIGSSSKAYTIAEGDNTFNFAARYIATSATVTGGAANATAVFALTYK

>WP_065793661.1 sulfatase-like hydrolase/transferase [Burkholderia pseudomallei]

MKRNSKQFEIGAWLGACALAFASAASAAAVQDRDHDSRPVDAKRVLLVSIDGLHEQDLARCIGANTCPNLALLAKSGVTYTNARTPGLSDSFPGLAALVTGGSPKSAGLFYDVSYDRTLYAPSDATCSGKQGWNVVFDETTGIDAMNGGALTHLDGGGAFNPQAIPHARVNGQCVSVYPHDYVKTNTVFEVVKEHLRGSHTAWADKHAWGYDWVNGPSGKGVDDLARTEINSIDPATGTPYTDIYTHTEKFDDYHVQAIVNQIDGKNSTGTAAAPVPTLFGTNFQTLSVAQKATVASGGGYLDASFTPGPEVANAIAYVDGALGRIVAELRQRGLYDSTVVIVTAKHGQSPTDHTKLVKHGDTLTALLEANGFVDPNGNFGQNNTASGNPNDGTGLVGTGFVQTDDVGLVWLRDPRQLSAAVATLKANLGCNAPGICADGPQAYILYGPSVAERFGNPALGRTPDIVVQPNPGVIYTSSKKKDEEHGGNAPDDSHLGLLVSYAGLRQGRTIDAPVLTTQVAPTILRSLGLEPRLLHAVALEGTRVLPGLGLER

>OMR39540.1 sorbosone dehydrogenase [Burkholderia pseudomallei]

MRSLVALFHHVSSGPPALPRAASAACLVLATIVTVATAPAASAALPIDELRVPPGFRVQVLADDVPTAREMAWSPRGILYVGSMNGRVHALVVRDGHVREHHVIASGLEMPVGVAYRSGALFVSAVSRILRLDRIDERLAAPPKPVVVTNALPTDRHHGWKFIAFGPDGKLYVPTGAPCNICVADRDRYAMIGRMNADGSGYEVYARGVRNTVGFAWHPATRELWFTDNGRDLMGDDRPDDKLNRAPRAGLDFGYPFCHGGDVLDPQFGRGHTCSSYAPPVLKLGAHVAALGMRFYTGGMFPPEYRDNIFIAEHGSWNRSRKVGYRVVRVIASPDGRAAREETFVHGWLRPDESVWGRPADVLPLPDGSLLVSDDYAGAIYRITYDATH

>OMW33686.1 hypothetical protein AQ807_07070, partial [Burkholderia pseudomallei]

NSNSNSNSNSNSNSNSNSNSNSNSNSNGNGNSSRHRYHPRLRRTPYERSSNYPLEVPQGGSMSTNMKRLMIAALGAALAFGALSARAASFDCAHAANAAERAICGTPALGELDVRMAAYYEMLQNARPADEGMAYREFRDALRDEQQRWRQRTRDACGARIDCLTNAYTARIAALRGVAAERLVLRMTGGSAASAGAADATYAIEGESITLANGESVRPAAPGSAMKRVTTLVARSAVATIAGRPVEAVLLSDDPGGSGRFLYVATAQPGGGAPAVLLGDRVKPVSVSIERAATGGAVVVVEYLDRPEGAPFAQAPTIKIVRRFALEQGRLVEQRG

>ONC96520.1 UDP-N-acetylmuramate:L-alanyl-gamma-D-glutamyl-meso-diaminopimelate ligase [Burkholderia pseudomallei]

MRGGASAALSMFSGNRRDVRPAADFNYAHAARSSMHIHILGICGTFMGGLAVLARAAGHTVTGCDAGVYPPMSTQLEAQGIQLIEGYGAEQIDLKPDLFVIGNVVSRGNPLMEAILDRGLPYVSGPQWLGEHVLAGKWVLAVAGTHGKTTTSSMLAWVLEDAGLNPGFLIGGVPLNFGVSARLTDSSFFVIEADEYDTAFFDKRSKFVHYRPRTAILNNLEFDHADIFPDLAAIETQFHHLVRTVPGVGRLVTNGRDDALERVLSRGCWSDVERFGVDGGWQALPVENGVPVDGRFAVYWRSERVGAVDWQVQGEHNRMNALAAIAAARHVGVPPAQAAAALAAFRNVKRRMEVRGSVDGVTVYDDFAHHPTAIETTIAGLRTRIGRENTRILAVLEPRSNTMKLGVMKAQLPASLADADLVFGYGAPSGRDALGWSLPDALAPLGGKARAFDDLHALVKAVTASARPGDHVLVMSNGGFGGVHQKLLDALSARGDAAPARSGA

>WP_076802983.1 MULTISPECIES: chitinase [pseudomallei group]

MERTMNFSMLSRIVPRALAAGCLFAAAGASQAAGVYAPYVDVTLYPTPLVDQIGVQQGIQQFMLAFVVSGGNQCTPSWGGVQPIGNGATGDLLDKIATSVTAYRAKGGDVAVSFGGAAGQPLMQACSSVAALKGAYQTVIDTYSLTHVDFDIEGASQQDSAAVARNFQAVAQLQADYAAKGKPLHVTLTLPAMPTGLVQDGLNVLNAALANNVTLDAVNIMTMDYGPSGIDMGAAAISAAQGLYSQLDTAYKSAGKPQTDAQLKQLVGVTPMIGVNDVAGEIFTLANAQSVQTTAANNNYGFVGIWSITRDKACDGSSQYASPICSGVAQQPYAFSSVFKQLGGHWGAGVTQDPNYGGGSDGGGKPQPGAPWSATQVYTAGATVTYQGTTYQAQWWTQGDIPGQASVWKPVGGNVPAWSSTTAYPGGACVTYQGAKYCAKWWTQGDVPSAGGPWTRA

>WP_076819717.1 NAD(P)H-dependent oxidoreductase, partial [Burkholderia pseudomallei]

STERALGFALRGAQAAGARTRLFDGPFLHTLPHYAPERRALTDAQRELIDAVRAADALVIATPGYHGGVSGLVKNALDTLEELRADERPYLDARAVGCIVTAYGWQAAGTVLTSLRAIVHALRGWPTPFGATVNTLETRFETAGSCSDSKVAAQLETVGAQAAEFALAFASHRAATRVAGGETLAPVLEAAAG

>WP_076848120.1 cytochrome P450 [Burkholderia pseudomallei]

MSKHRQAPGPRGGLVMGNLAAYKRNPITMLLRLHQQYGDVARNRLGPFVTHALAHPDHIQYVLQENHRNYVRGRFYDNFKMFFGDGLLTTDGEFWRRHRRVVQPLFHKKQVDAHTAAVGDAALALAHRWSALPPGKALDVVEEMMHLSLRMLGLMVFNTDVSSHAEAVGPAVRFGIEAMMPQGNLNDFIPRWAPTRFNRRIAHARRAIDTIVAKIIADHREARCEPSDVISLLLNARDPDTGAPMTQQEVHDEVMTVFLAGHETTGAGLAWALYALAQHPAVLRQLRDELDARLGGRAPTVQDFEQLPYLSQVVDEVLRVYPPIWGFTRDLVEDDEIGGYRIPARSSVFMSPYVTHRHPAFWRNPDAFDPENFASDAPARHRFVYFPFGGGMRKCIGFQTALLQMRVLVAVVAQHFDLNALPGHPIELGATISLRPVHGIRLIVKPRERQQSHLARVREHESARALRPLGDTATTAGAACPMTAAREPGASAAAAVAPAALAVPAASAASAASAARPAPLAQPAAPAIVDSQPQTRAQVYEAVKQMTALGRQLFFDPSLSGSGKLACASCHSPQHAFGPPNALPAQFGGDDLRQQGFRAVPTLKYLQKVPAFSEHYHESDDEGDESVDAGPTGGLTWDGRVDSGAEQARAPLTSPFEMNGTPEKVARAVRAAPYAPAFRAAFGARVLDDDRATFEAVLQALGTFEQAPDVFYPYTSKYDAYLAGRARLTRAELHGLQVFNDEKKGNCASCHVSRRGLDGSPPQFSDFGLIALGVPRNRALAVNRNPNFYDLGACGPERRDLKGRDEFCGLFRTPTLRNVALKKTFFHNGVYHSLDDVLRFYAERDTHPEKFYPVKRGVVQKFDDLPKRYWKNLNDEPPFERKRGDPPAMTDAEIRDVIAFLGTLTDGYDPRAKPAGGAR

>WP_076852667.1 depolymerase [Burkholderia pseudomallei]

MRHAAARAVLAAAFTSTLAAAPAGARAAPPLPALRADANRVSVSGLSSGAYMALQYQVAYSASVVGVGVIAGGPYYCAAGSVANTDLCRGLVPNMVPDSGRLVAAAQGFAASGQIDPLANLQRARIYLFSGTKDTLVRQSAVDATWSFFWLVGVPVTNIVYVADVPAGHAFVTPSAGNACDANAAPFISHCTVGQSGYDQAGALLDAIYGPLAPPAATPTGRAIAFDQREFAPASSGLAAQGYAYVPRTCDANAGCKVHVVFHGCLQSAAVVRDMTTYDNWADANGIVVLYPQVAKTSTPNDPQGCWDWFAYTGQNYAWKSGAQMRAVRAMIERVTSAP

>WP_076887541.1 peptidoglycan DD-metalloendopeptidase family protein [Burkholderia pseudomallei]

MVWRGASDSITRWLAAPLIAAGLTGAASAAPVDMPDLQHAVRQAFSARLGKQAPAAPAVRDDLIESVRSDPEAGWVLGTVTQVVPNDTPAYPVTKLFIARRASEGWAVGIEGTDAFYTLAAASPAKLLAGDERAHLNAGRAPTAPPRKAVPAQTGLALPWQQGTAWYWTGGAHGWSGDSRPFNSLDFSGGNGQVLAARDGTLYKSCERNGSAIVKVVHDNGYTSTYYHMVQLTQAGSGTRVRQGQYLGRVGNGLPCGGQTTGPHVHFALSQGGSDVPVNGKTIGGWQFFEGSNAYSGYAVRNQRRVSVQASLTNYGADDSGGPTEPSPPVKATVQSPGPVNLRSAPSLSASIVGTVANGAAVQLACYAYGDTVQGNWGATRLWYRLDSNRWVSDGFVYTGSNDPVVSACAN

>WP_076889967.1 hypothetical protein [Burkholderia pseudomallei]

MRNADGTAPRAPLPSRIAASVLACILAAALAGNARAAPASPRTGGDPYAFAVLSGVIASADDEPQARRLLDAIARDRSVSFIVYEGNLKGPKEACRDQLYEQRNVLFATSRVPLVLLPGQYDWAACGTRDAGAYDPVERLDFLRQNVFTEPASPGANPLPLTRESEVARFHPYRENVRWIRDDTVFIGLNAPAPNNRYLTAGGRNGEFEDRAIANAFWIDHAAEYAKRRKARALVVFLEGDPQFNRYERSERFGWLRFNRPRVRDGFREFKRALVKAAAVFRGSIVVVHPSGEPLADGFRIDRPLRDDKGELVGNLTRVAIAPHARLTHWVRIGVDAAKQPMFNVSMQTVPKYLPQPPALPVVPHDDTPLPDMPEIPAQPVLPDSGASGAAPPGYGGWHGAHEGSFDHRAPSGATPGDAPDIAVPGSMQGTR

>WP_076902853.1 hypothetical protein [Burkholderia pseudomallei]

MKKLLIATVIGALSATMLASAPSAFAQDSSATAKKATPKRPAPKRHLIPRSKKAQARAAAKTDPVPEGAVKWACKDGLSYELAGDMKRDQIVTVHWANKNYKLPRQQTTTGADVFYDPASGMKLVVIPTKGMLFSDKDDSRLADECQTPEMAAGNGLAPTQSNELKPSN

>WP_076903047.1 cytochrome c1 [Burkholderia pseudomallei]

MKKLLSTLAMFIVSACLLTSASVRAEEGEFPLDRAPDNTENLVSLQHGAQLFVNYCLNCHSANLMRYNRLTDLGISQKEIEKNLLFATDKVGNTMTVAMRPEDAKTWLGVAPPDLSVEARARSRDWLYTYLRTFYRDDTRPTGWNNAVFPNVGMPHVLWQLQGERIAKFEEKTDEETGEKTHKLVGFQQVTPGTLAPADYDAAVGDLVAYLNWMSEPAQQTRKRLGVWVLLFLGVLTFLAWRLNAAYWKDIK

>WP_080248664.1 c-type cytochrome [Burkholderia pseudomallei]

MSVKPAQRPERGFAKTRRRTAAYRPAATRAAGVARAAGAATPPLPAAPHAAAAHAQAHAAQPAALPLAYVFDSAGPAPRPVLILGWALLALCTSVCLVIAVLPALALFLGRPASVGLTERGGLGFVYVGSAISTALLLAALVYMLWVLAAVAKPPRPPAVTIAVTAYDWWWKADYGGGPPDGFTTANELHVPVGEPVLIELRSADVIHAFWAPQLAGKTQAIPGQINRQWMQADRPGVYRGQCTQFCGAQHAQMGFEIVAEPPDAYRRWYASQRRGAEAPRTADALRGQRIFADRCAGCHAVRGTGAAGTQAPDLTHVGARRLLAAGALANTPDELRRWIADAQQVKPQSLMPSIRLDPAQQRDLSAYLATLR

>WP_080248669.1 TonB-dependent siderophore receptor [Burkholderia pseudomallei]

MEQRKMEWATSTRVRAIAAGVAFYAAAAGHAQAQAAQPGTDARQPGGEAKADTAAGGTLPAISVSGAAERDASVGLVARRSMTGTKTDTPIIEIPQTINVVTAQQIEATGATDINQAFRYIPGFSSYGSDNRSDWYAALRGFTPTVFVDGLQVPNTINLSSWRVDPYMIDSIAVLRGPTSVLYGQGDPGAIVDVQSKLANGERIRELGVQVGNYARKQLMFDIGDTIGKDGTLSYRIVGVGRDGNAQTGPLADQRVSFAPSLKWQPNADTSLTLAATYLQDWGDTSSNFLPSRGTVLPNPNGTISDDLYTADANFDHYRKKQWSLGYQFEHKLNPVWTLRQNVRWMHLSLDDASVYGGGLDDADPTMATMTRYAGLFQFNYSRFDVDNQAQAKFTTGPLSHTLLFGFDYNRQTTTDSEWLAKGPSLNLYRPVYTPIPSDIFSGPNAYPRTDTKTTLNAFGLYVQDQIKWRRWVLTLGGRQDWTRTSQDDIANAASFRQNDHAFSGRVGLTYLGDYGLAPYLSYSTSFNPQIGLKLAGGGLATPTKGRQIEAGLRWQPPGKNLMLNAAVYQINQTNVAMSNPNDPTSSTFVQVGEVRSRGVELSAVGNLSRELSVIAAYVYQDVKNVRANDNTLNKWPVDVPRPRQIASLWADWTWRTGPLTGFGVGAGVRYMSAAAGAADNSLTVPSYTLFDAALHYELRNWRFALNATNLFNRRYVAGCQSDAVCMYGNQRTVIATAKYNW

>WP_080248725.1 patatin-like phospholipase domain-containing protein [Burkholderia pseudomallei]

MTVLASSARAGFRVPGFVAALLFVCAATGRPAPAAAADTATVASAATAAASAAGAPASTTLPPAATPTRVASSAVAASSPASSLASSSASAATPANAAAAWSWTAARRADAPDSSSVSNAVSNSGSNVAGTAAAHGPHAPHTAPDAGAGAPSAPTTPTAATPAAAGASASMAAAPTPAATDTLVCMPDGGGPHRPAIGLVLSGGGARGYAHLGVLKVLEANRIPVDCIAATSMGAVVGGLYATGMTAQDMQRRLSQVNLADIAFDVTERSDLPQKKREDERLYIDSLTIGFDSKGFKAPVGLVQGNRLQALLANWTAAVPTNQPFDRLPIPFRAIATDLQTGQKVVLDHGSLPLAIRASMALPGLFSPAEIDGRALVDGGLVGNLPVDAARAMGADVVIAVDIGSPLRPLDALASPADVMQQMIGILIRQNVAEQRKQLTANDILLQPDLGKQTFTDFQNANQAIAAGEAAAVAALPRLARYALSPEQYEAYRAAHARPQQPIRITSIEIRTNGASVPKQIVRNALRVKPGDVYDPQAVSADLLSLTTSGNFENVTQQIINEGDEHRLVIDAQEKYWGPNFLLFGLGMSSSSTDEGGFRLHLGYRRPWLTPSGLEFRADTTLGSDMQSVHVELRQPLSNKTGYYVAPYADYKRRFTNLYDSDTDIKITQYRFQTSRVGLDLGLPLARLGDFRIGLAYTHLTASPTYNVPLNWFLPDDAPSPGTLFPSAYGHQISARARLVIDQLDDPTFPRKGYFVEARVERSLSKSNDTFFDSDASFTDVYGRLMVAQRFGRHSVNISVEAGKSFGGTNFGNPLGYTLGGFQHLSAYAADQLSGNALLYGQVTYMNQLATFNASPIKALYVGASAEVGNVWTPDTRIGSGSLKQSYTFFTSLTTAFGPVYFGVALAPGGRRNIYFQLGRTY

>WP_080248756.1 PhoPQ-regulated protein [Burkholderia pseudomallei]

MRNKVAGAIALSMLSLAYPRAEAAPIAFPCPIDARRDLTDTLACYRQTVENQPLNYTMTGIVQLPGIEQRTYRLVSQSWSPDQLVRPDTWVHEVALYIPQDALPRRALVIANDGTRHPGEGETPRTPNDFLPDTLADIARSTRTAVISVSDVPNQLLSYADNGKPKAEDDSVARSWTLFMQSPHTRGAMPLHVPMAASIWRAMSLAERELTSLGIHRFVVSGISKRAWTTWLALIGDQRVDAIAPFAIDLLSTRAALENMYRSYGGNWPLAFYPYYAEGIDRTLDSPAFGLLMRIEDPLSYLGTRHGSRLAVPKYIVNASGDDFFVPDNSQRYFDKLPGAKALRMVPNSSHSDIRRATLDSLVPFVKRIQHGKALPQVDAAPIETNGQTILRLRTSERPRQLLLWQATNPLARDFRYACGIRYTSTPIAMNGARPQQLVLVPPTNGWRAYFVEATFDDGFVATSQTYILGDGYPSTPPPAVGGSCRTLPGRDTHS

>WP_080300428.1 glycoside hydrolase 68 family protein [Burkholderia pseudomallei]

MTYSKQAVSRRHKRLALSAAALTAAACMSAHAQSDGAGPAPTPHTQQAYDPESHFTMRWTRADMRQLVKQSHTAGADKNSLPPALTMPDIAQNFPLVDSNVWVWDTWPLADMRANQLSYKGWEVIFSLSADPHAGYTFDDRHVHARIGFFYRRAGIPASQRPANGGWTWGGHLFPDGASAKVFGTAPMTNNAEWSGSARLTHGENVSLYYTALSFNRSAPGGADITPPIAIITRADGHIHADDKHVWFSGFDDHKALLQPEGKMYQTGQQNTYYSFRDPFVFTDPAHPGNTYMVFEGNTGGPRGARTCTEADLGYAPNDPYREDLNAVMNLGAVYQKANVGLAIATNPQLTEWKFLPPLLSANCVDDQTERPQIYLKDGKYYLFTISHRTTMAAGIDGPDGVYGFVGNGIRSDFLPLNGGSGLVLGNPTDFSAPAGAPYAQDPNQNPRAFQSYSHYVMPGGRVESFIDAIGARRGGTLAPTVKIDIHGDSTTVDRAYGAGGLGGYGDIPANLPAVGAGHHD

>WP_080310460.1 DUF3761 domain-containing protein [Burkholderia pseudomallei]

MHSLPLGRRYATEFLQRFAAVKMPSGRRARRQRRTMRETMPISLPRAARRIAVALAIAALPPAAAFAYSPTAPTAPTAHGEADLDRHDTYRNRDGDTVHAPAHSKSGRAPEGATARCRDGAYSFSRHRRGTCSGHGGVAAWL

>ARK97515.1 galactose oxidase [Burkholderia pseudomallei]

MTEWIRVRSPAFKRKTQFKRFQWEKARDRSPFRYMEINMKKREEKSLSVSHSRRAFLVWVPASLLVTACGGDNGSSAPASIKKAVAALTGPGGATWTGMGKGGILIQSPYSGAGNFELLAGDEYGMLHCLRRTNDNGAQWMVAVGDGMGSKNSVSGYCHTYSTNYTNIEAAVVDDGAVKTYFRSNSTPWSAHKTIPIGDAQGFPAYLQSNRNGAQQLELVVGMRRGGMVHYWRDDKDGFTWKQSSIFGSGQVKGVSVMQGNYIGPDNNANLELVAWVDDRLESWCCEGRAWRKICNIADGGVGGAPALLQSNYGTKGNFEVLVPLATGGIAHYWRNNDAEGIPWSFTGTIGTGAYVAAGLLQSQNGVKGDFEAIGLRASGKADVFRRGDTLVWENSATVSPFSEGAAADVGVSSHIVDVNVTGINSVLLKNGSVLMFGYYKGGSTNKTIPACIWNPVNDQITAIPSFRNNFCAGQTAMPDGKVLIVGGHIGDTLKDVVVFDPDNHTATLVATMTKGRWYPSTATLPDGQVFIISGTETAGWNTSVNDTWQTYANNALTAPEDVISPFSPYYPKSQTQIDLYPFLFVLPDGKLLVHARNTTRFFDIGTRSWSATLYKTVSDNSRTYPFMGGTAVLPLRPSENYRVKVVVAGGADKSAQIAIGDDSQYDNSVPGMTSCEMLDLGDAAPAWKAIAPLNEGRVMCDLVTLPDGKLFIVGGNKTGKADYGRGPTYRPELYDPQTNTWTLLASTRIARGYHATALLLPDGRIAITGKDGDYQGSGLQYAETRVEIFSPPYLFKGPRPAIQSAPASINHGGSFTLGLSSGTSPEDIGSIVIVACGSATHQINFSHRIVELVFAVSGGTLTVNAPPNANIAPPGYYMMFVLSKLGVPSVSSIVHVAAGSAQAASATPEALTRQPRVATVSIEQAGDAPRCAPLTVAEKQSINGMG

>ARL94721.1 DNA protecting protein DprA [Burkholderia pseudomallei]

MIGQSPWARACGIRSISDDENFITALRSPQAALFEEFIMSPRALTRAELSGWLRLAGASGVPAAACAALLGAFASLEALFAASHAELAALVGDAAAQSVLAPPAADFEQRVDAALAWLDEPGNALVTRHDPAYPGPLAELYDPPPLLYIKGRVALLHARAVAIVGSRGATPQGLADATRFARELSDAGLAIVSGLARGIDGAAHRGGLDGASGTVAVIGTGADLVYPACHHALAHEIAERGALVSEWPLGTPARSAHFPQRNRLIAALSGGVLIVEAALRSGSLITARLANEMGRDVFAIPGSIHAPLSQGCHALIRDGAKLVETAADVLDEFGLDPARPVNSKRGAPAAASADADLDDDTRRLLDAIGYGPVPLELLAQRTSLPSGTLHRLLLQLELAGRVAALPGGRYTRIDGAQAEPAQARGTSAVLHSGA

>WP_085538790.1 shikimate dehydrogenase [Burkholderia pseudomallei]

MSERSFLIGLIGAGIGGSLSPAMHEAEGCGQGLNYVYRRIDLDALGLTADALPELLAAAERMGFDGLNITHPCKQRVIEHLDELSADAAALGAVNTVCFAHGRRIGHNTDWSGFAKAFARGLPGASLERVVQLGAGGAGAAVAHAALTMGAAQLALFDVDPVRARSLADQLQARFPHARLSAGGTLADALANATGLIHATPTGMLGHPGLPLPAHLLRSGLWVADIVYFPLETELIRAARALGCRTLPGGGMAVYQAVDAFELFTGRAPDAERMFEHFRALVAR

>WP_085546935.1 Flp pilus assembly protein CpaB [Burkholderia pseudomallei]

MKNNRAFTMLAIAILAGLAAVAFASRWLVQTSSSAVTPVAVAATDVSLGEPLGPNQLRIVNWPTASVPPGAFTDLKPLEGRVVRTSLARGEPVLGTKLAPVGTKGGLSAVIAPGHRAITVRVNDVVGVAGFALPGNYVDVIVNTQEQSKADPQQSISKIVLEKILVLAVAQQVSRDDTAPKVVNAVTLEVSPEQAEKLDLARSVGTLSLVLRNQIDKDALSTGGATKNTLLGQPAAAPAPIAAPAAQPRPVRTRVVAHARAAAGRDCVGVLSGVTGSVECF

>VBI96880.1 adenylylsulfate kinase [Burkholderia pseudomallei]

MTSMSLLAAVYSGRREVSIDGCHEVQNIAVAAALDGGQAAPSAHGAVQFTDRKLMDKLHAERNVEWQTTSVSRRDRESRQAHGALAVWLTGLPGAGKSTLAIEAERCLHQAGKRTYVLDGDNLRHGLCRDLGFSADDRAENIRRAGEVARLFVDAGVIVLSAFISPFRFDRAKVRALFDAGDFIEVYCDCDPRICEIRDVKGLYRRARAGLISEFTGISSPYEPPRHPDVYVNTGKESLDACVDRIVRRVLHSLR

>VCQ54129.1 phosphoserine phosphatase [Burkholderia pseudomallei]

MESLSGFVVFVQVAETRSFVAAARALGVSASAVGKRIARLEARLNVRLFHRSTRSIALTAEGARFLERCRRVLAEIEEAEQELSRSAHAPSGRLRVSLPALSAPVLPVLADFMAAYPDIQLDLDFTDRLVDVIDEGFDAVVRGGEPRDSRLSARRLGTFAQVVVGSPDYFARRGTPRTPADLAHHTCLLYRFPTTGKLERWPLRPAPGDADVEPPQSMICNNVETRVCFAIRGRGLACVPDFAVRDELASARLRTALDAYIERPQTFHVLWPSGRHASPKLRAFVDFIVARSTWPPASRGRGAPMRTGAPPGMRPRRGAPRAATAHRTSSHLIASPRDTPRRCPCRRGPAAPPAARESVVKSAFESVRRGKTARAARVGRISTSGCPFSTGAPFYGQRCWAAPASAGGSAGGTRPARPRRANPPRRNSMKTGRRHFVRSVASASAALAAAAWSPARAAIDAPASPATALSLTPGRWSPNNVARLRAVLAGHGASSPRYRPEHRPYAVFDWDNTSIMNDCEEALLMHQIDGLHYRLTPEQFSAILRQGVPDGPFDAKLGYTSVDGKPVRMEDIAADVDADYRWLHANYRGLAGDKPLDEIHRSEQFRDFRAKLYFMYDAICDTYPVEIGYKWIMYWYAGMTRDELQAMAFDSNVANLGDALRKVTYESSRALPGKAGVIAATHFHGIRIHEEIRAVMDTLRSNGIDVYVSTASLDDVVRVFAGHPAFGYGVPAENVIGMRLTMADGKYMNEYLPNWHFNYGPGKTVGIRRELEAKKGYGPLLVFGDSDGDAWMLRDFADTAVGVIVNRMKKGEIGIDSRKAAEQIGAKDARLVLQGRDENTGLMVADERSIKYGKRDPKLLA

>VBC72697.1 Uncharacterised protein [Burkholderia pseudomallei]

MKHDAAHHDERAISMNHSAITWAAVLALCAGFAVTNAHARDSAECRRLSDATGGADDNFRPPLTATVTGAGRAYFHSAPASACIARRVFVVPGDTVTVYKPYRHWYQVTYVNGRTGEDVEGWIEAGRLRLGGHLGGGQ

>VCG67913.1 3-demethylubiquinone-9 3-methyltransferase [Burkholderia pseudomallei]

MRPASPAAAFLATSPACLYTRGLAGAPPPSFPHPLADMTNADPHELQKFSDLAHKWWDPNAEFKPLHDLNPVRLSWIDAHAHLPGKRVVDIGCGGGILSESMASLGAQVKGIDLATEALGVADLHSLESGVSVDYEAIAAEALAAREPGAYDVVTCMEMLEHVPSPANIVAACATLVKPGGWVFFSTLNRNLKSYLLAVIGAEYIAQMLPKGTHDYARFIRPSELARFVREAGLQMVEIKGIAYHPLAKRFALSNDTDVNYLVACRRGA

>WP_122634654.1 mechanosensitive ion channel family protein [Burkholderia pseudomallei]

MIRLATFARRVALIGLLHLLCAALPAAAADLASGASVPPAPAISRDEALAELKRVQAALDRIKQQASAATTYKQLDALDESTQALSADVDKLTAALVPTRAQLQAQLDVLGPPPAPGAAPETPAVARQRADLNARKTQLDAALKQAADEKESLANLTQQYSKLRRSLLRDQLVLRSSSILGPRFWAPLFRPSDDDRQELGAFSAQIGQVLAAAWEPGRRLGTALLLAAALAVWTLGRRLAERALAWFSLTKLPETRVRRSALATSIALSSVVTTGIAVQFVYLALTRDYALSSALQDFADELAKLALTCALIAGLGRALLCTHHPSWRLPALADEVALAMRPFPAILAALLLVAGALEELNRTADTSLQVTLFGRGIVSLVVVVTIGASLLRANRARTALAAAGEPPEARSTLAGLIHAGVTLTVVAALAALLIGYISVARFLTYELVWFEIVIGSVYLLTRLTRDVYASAFAANLSSGRLIKHLFGLNDSHLDQARTVLTGISTSVLLLVAVIALLTGGFGTTPNDLLVSVISMFGGDKLQRLNIVPARILNAALALAIGIYLLRSVRRWLDNELLPAIGLDQGMRASLVTLFSNLGYVALVLLALSLLGVKWDNLAWIVSALSVGIGFGLQEIVKNFVSGLILLTERPVKVGDMISISGVEGDIRRINVRATEIQLSDRSTVIVPNSQLISQNLRNVTMGNSTQGVATLMLTFPLNTDPEQVRDLLLDAYREHASVLEKPAPSVTFSQLTPDGITLSVTGYVGSPRIAAATKSDLLFEILKRLRAAGIALSNPQTLMVQNLPPPSSE

>WP_122651768.1 metallophosphoesterase family protein [Burkholderia pseudomallei]

MSNKPAPDAPPAEQPCAVSRRGFLKFVGVSGLASAARGLAATRAAAAAPDGTPEQIHLTWGDVDANEVVVSWASLAAATNPRVRFAGPNEAWRTVHGVQRTYTDGLNGEVVFTYHARLRGLKPGAVYRYEVTADNDANAAQPFAARFETAPRGRAPFRWTSYGDLATPNTGWVLSSPQSRFAVQAVERFQPLFHLLNGDLCYANLNPAHQPAVWRDFGNNNQTSAANRPWMPCPGNHEIEFHNGAQGLDSYLARYTLPENGTRFAGRWYSFRVGAVLFVSLDADDVVYQDAAAFVAGPNPLVPAASTGNEAIAPGTSLYVRGYSRGEQTRWLEQTLRRASRDRDIDWIVVQMHQDALSSSKTGNGSDKGIREAWLPLFDRYGVDLVLCGHDHDYERSFPVRGCNHRAGVDAATGEVVDTLQPRPVVTTDPADGRFDTSHGTIHMILGGGGTSAPLDVYGENPATGLPQARVFTKPNRPVPGAAPNTFVRHGADAVEDAIWSARRDTGTGYGIAVFDYEPGEHGGRSTITVNYYHAPGADQHPTAEYELFETIVLSKPRRA

>WP_122821554.1 hypothetical protein [Burkholderia pseudomallei]

MPTLAKSLLLTLMACFALSGEAGAAQKTFCYFHHNCPADGKLPKGKPPEQYTKRQCKNIGGKSWGDGPTSCENVNK

>WP_122826861.1 lytic transglycosylase domain-containing protein [Burkholderia pseudomallei]

MEKRFVIVAMFAAGAWSASAPARADCYDEAAKYQKVNPLVLRAIAWQESHNRPEALNKNANGSTDYGLMQINSIHLPTLSRYGITKDTLMEPCKSVYIAAWHLRRKMDKYGNTWQAIGAYHSETPSLRDKYARQIADILARWKLLPASAASATQDTQSAQR

>WP_122827599.1 cytochrome-c oxidase [Burkholderia pseudomallei]

MRRLRRRWLFAASGAALAALAGVDASRVRAAAPRVIKVHARRFVFTPDRIALAPHESVVFELTAQDTVMGFSIPQYGVRADVPPGAVVRLAAQAGGPGTVQFLCDIFCGSGHETMNGVLVVG

>WP_122830792.1 peroxiredoxin [Burkholderia pseudomallei]

MNRKLASGAAAALFALHAVAAHAVLKVGDAAPDFSAQASLGGKTYTYSLADALKKGPVVLYFYPAAFTTGCTIEAHAFAEAIDAYKRYGATVIGVSADDIGTLTKFSVSECRSKFPVAADPDAKIIREYDAKLPAISKANRVSYVISPDGKVLYEYTSLSPDKHVENTLNAVKAWAASHRQP

>WP_122831074.1 NAD(P)/FAD-dependent oxidoreductase [Burkholderia pseudomallei]

MEKFDIAVIGAGAAGMMTAAAAGQLGRRVVLLDHSPRLAEKIRISGGGRCNFTNLYAGPDNYLSANPHFCRSALARYTPRDFLGLLKNHRVTWHEKHKGQLFCDHSSDAIIDVLKSECDAGGVAWRRPVTVDAVRHAQADGFALDTSAGAIGAQALVVATGGLSIPKIGATDFAYRIAKQFGHKLVDTRPALVPLTFSADAWAPFAALSGVSLEARVSTGAKRGGGEFVEDILLTHRGLSGPGILQISSYWRSGEPIHVDLAPQIDAANELIRAKAGSRRQIGTLLAEWVPARLAHAWLEAERVHADARLADLPDKTLRRIGETLSRWTLTPSGTEGYRKAEVTKGGVDTRELSSATMMSERVPGLFFVGEALDVTGWLGGYNFQWAWASGVAAGRAAADGTRRLTG

>WP_139900217.1 c-type cytochrome [Burkholderia pseudomallei]

MKRKSLFALSAVAIVAASALVPVLWPGNDTLHGNAAVAATPADQAALIKKGEYLARVGDCIACHTVRGGKPFAGGLPMATPFGTMYTPNITPDDQAGIGKWTSDDFYRAMHTGRSKDGSLLYPGFPFASYTKVTRADSDAIYAYLRSVAPVSTPSRPHELRFPFNNRNLLIGWRTLFFKEGEYKPDPTKSVEWNRGAYLVEGLGHCSMCHTSINMMGGPVSSAAFAGGLIPLQNWYAPSLTNDKELGLGDWHVQELSDLLQAGVSHKGAVFGPMADVVHNSLQYMTDEDTRAMSTYLKSIPQKAEAPKNMQYEPSKQFGTALLEQGKKIYADNCATCHGPQGEGKPTAYPPLAQNRSIMMESAVNPIRMVLNGGYPPSTFKNPRPYGMPPFAQSLSNQEVAAVVTYIRMSWGNNGSPVSPQQVSDLRSAPLD

>WP_139910463.1 hypothetical protein [Burkholderia pseudomallei]

MNNLIEVVSIAAAAPAASADSQALDAALDCSSTGHAFVAPLVASGAVRSQPMHVEANSMNAFRTNRSLSAYGFSVYVVLGYQANDPLFAHGDGEPIGDWAYGVVVRGSKQAVEAKVRAAGSQAVVKDAFPFLTAIVCTSP

>WP_139910601.1 phospholipase D family protein [Burkholderia pseudomallei]

MSKPPRRALVLCAALALSGCATHPPATTLERTVSHALPPDASTPLADALAVQARAHPGESGFVVLPRGDEALQMRIAVARAATKTLDIQYYIAAEDTTGKLLLGAALYAADRGVRVRMLVDALNFKDIDKLMAALDAHANLEVRVFNPFGAPRLGMFARTANVFTRIDNFTRRMHNKAMISDNQIAIVGGRNLGDEYFNASPTLQFRDLDVLAAGPVTRAVSASFDAYWSSALTYPLPALNRRRYDAKDLDAARDALRAHWRANATPYNAKPLNATPLAAQIARNELGLVWASAEFTADSPEKIAAPDDSYKSPPMQRLFALTRDAQREFLVLSPYFVPHDAGVNALGRLTARGVRVAILTNSLAATDAIAVQAGYAPYRVPMLERGVELYEYKPDPGRSRIGMLGSRSRASLHAKAYVIDRKILVIGSMNLDPRSAHLNTELALVIHSPRLANEIANLFDEVTKPTISYRVTLAPDTPGAAQTTGAGAPAWPLVWTEIADGQVRTYSVDPNAGFYRNLLTGLCLLLPIDDQL

>WP_151269450.1 molecular chaperone [Burkholderia pseudomallei]

MPVRARARRAAAALFIALAGAAHAAIVPDRTRVILNEGEQAAIVTLSNKSDAYPYLVQSWLEDERGNRITTPLMVVPPLQRVEPKERNVLRIARMPGATLPADRESVFYLNIREVPPKTDTPNALQIALHTQMKLFYRPAGVQPARDEDPTLPMTLRVDAAARKLVFDNPTPYHVTVVALAAGAHRAALPLDPVMVNPKSAASVPFSASAAGGLFVTHVDDYGGQVTVEYTCDGLACRSVKR

>WP_151271354.1 DUF1223 domain-containing protein [Burkholderia pseudomallei]

MSTDMSISFSVSLRAVAAAFSLAAAGAAQAAADGACMARSPDGRQALVELYTSEGCSSCPPADDWLARLAARRAALRVVPLALHVDYWDGLGWADRFAQHRFTERQHALAARGGGRFVYTPEVAVDGRELRDWRDADAFGRRVVATAAEPARVGIALAAKRRAGALDVALSVTPRVGAPRALDAYLALYENGVESQVRAGENRGATLRHERVVRQWIGPLAATADAHASLDVRRALPLPANLRAADAARYGVAAFVEDPATGDVLQALDLPLCG

>WP_151277731.1 cytochrome c [Burkholderia pseudomallei]

MNSAKHVVVAAALAALAAGAQAAGVVGNPKDGASKAAMCIGCHGIDGYRVAYPEVYRVPLLGGQNQVYLENALKAYRKKDRHFPSMNAIAESLTDQDIADLASYYAAQKPDSKNNPYK

>WP_155027764.1 ABC transporter substrate-binding protein [Burkholderia pseudomallei]

MKPLSLNMKRALASACIGAAGFLAQQAALAQSCGLANGKPATGAPIPIGAVVGKTGPDDFSSSARAAAAYFKCVNANGGINGRPVQYLVEDDQWNPETASQVASKLVRDRKVLALAGNASFVECGANAKFYEQENVIAIAGVGVPRECYFARNYAPLNMGPRLSMTEAALYAKQQYKATRMVCIAPNIPSLGAWSCEGPALWGKRNGVSVDTIVMDPDSADPTSVVLQAASKNPQAILLGLPKGLMVPILSAAEQQNLGRRIHFVSAASGYDLGVPKAIGPYWKGNFDVNLEFQPLDAQTPDNRNWLAVMDKYGDRKDPRDTFSQAGYLAARLVTDTLLKLPANQLDRAHVTAALREVKDFRSDILCGPFYVGAGERHNANNAGRMAQSTGTGWKTVSTCQAVDDPQLADIRAAEKKMH

>WP_161788821.1 ABC transporter substrate-binding protein [Burkholderia pseudomallei]

MSIHAESDWASEPIDSFEAARQSKIVTFSRARVDMNVRMRCLSLLAAVAFDAHAQSSVVRIGVAMPLTGPVAHLGKDVQNGAQLAVDELNRAPPTIDGKPVKFALVVEDDQGDPRQAVQVAQRLVDARVAGVVGDLNSGPTIVAAKVYAAAGIAQIAPAATHPAYTQLGYKTAFRLMATDNQQGASLAGLAAKLAKGRPIALIDDRGAYGQGLIDQTEKTLRASGVTRIIRDYTTDTAVNFASILTRVKGAHAAVIVYGGADAQAGPMVRQMKALGIDAAFVGSDGVCTGQWTALSSGANEGQFCTQAGDPRARMAGYAAFERRFEARYGKVIVFAPYGYDAVMLLADAMRRANSTEPAALLGALATTRYDGVIGRIRFSPQGDNLNGAVTVYRVQRGALVPVSD

>WP_162492307.1 hypothetical protein [Burkholderia pseudomallei]

MSSRVVACRRVSSRVVACRRVSSRVVACRRVSSRVVACRRVSSRSSRSSRSSRSSRVVAGRRGFAFGARAARRRRTRSSGAPTVEAIVFQKDSMKKILAVIAFLAVVGWLAATTTVLHAPSAQPCTDAWFDAIDKQFDITDNAGHGPDPGSGEWLGVVERKAKLPESGQLTEQQRCEAIQRELSQRTYLVNRRLGLKLAL
